# Machine Learning Accelerates Crystallization for Structure Determination

**DOI:** 10.1002/anie.1218503

**Published:** 2026-05-04

**Authors:** Cui‐Zhou Luan, Xue‐Zhi Wang, Jian‐Guo Song, Yu Gu, Jing Wu, Ye‐Ting Wang, Jin‐Feng Liang, Jia‐Le Rao, Mo Xie, Jonathan R. Nitschke, Dan Li

**Affiliations:** ^1^ State Key Laboratory of Bioactive Molecules and Druggability Assessment College of Chemistry and Materials Science Guangdong Provincial Key Laboratory of Supramolecular Coordination Chemistry Jinan University Guangzhou P. R. China; ^2^ State Key Laboratory of Bioactive Molecules and Druggability Assessment Jinan University Guangzhou P. R. China; ^3^ College of Physics and Optoelectronic Engineering Jinan University Guangzhou P. R. China; ^4^ Yusuf Hamied Department of Chemistry University of Cambridge Cambridge UK; ^5^ Department of Ultrasound Institute of Ultrasound in Musculoskeletal Sports Medicine The Affiliated Guangdong Second Provincial General Hospital Jinan University Guangzhou P. R. China

**Keywords:** co‐crystallization, cyclic trinuclear complexes, machine learning, single‐crystal X‐ray diffraction, structure determination

## Abstract

Single‐crystal X‐ray diffraction (SCXRD) is a powerful tool for structural elucidation, but requires high‐quality crystals that are often difficult to obtain. The crystalline mate strategy helps overcome this limitation by facilitating the co‐crystallization of volatile or complex molecules, with few restrictions on size or purity. However, defining its scope of applicability remains challenging, until now requiring experimental trial‐and‐error screening. Here, we demonstrate a machine learning (ML)‐accelerated workflow that rapidly identifies suitable candidates for co‐crystallization. Through feature engineering and workflow optimization, we trained the **MCC** model, achieving over 95% prediction accuracy. Experimental validation confirmed 114 successful co‐crystals among 120 predicted compounds. The wide structural and functional diversity exhibited highlights the robustness and broad applicability of our strategy, enabling efficient discovery of new structures by SCXRD under standard laboratory conditions.

## Graphical abstract

Single‐crystal X‐ray diffraction (SCXRD) is often constrained by the difficulty of obtaining suitable crystals. Here, a machine learning‐accelerated co‐crystal discovery workflow is established for a crystalline mate strategy that achieves over 95% prediction accuracy and experimentally delivers 114 co‐crystals from 120 candidates. Rapid experimental validation enables efficient discovery of diverse co‐crystals, significantly expanding the practical scope of SCXRD under standard laboratory conditions.

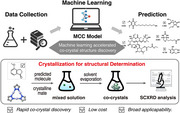

## 1 Introduction

Single‐crystal X‐ray diffraction (SCXRD) is a powerful and widely used technique for unambiguous molecular structure determination [1]. However, it requires high‐quality, well‐ordered single crystals, which can be challenging to prepare for molecules with high conformational flexibility, which tend to crystallize poorly and exhibit intrinsic disorder. In recent years, assisted crystallization strategies have emerged to address these limitations [2, 3, 4, 5, 6]. Foundational work by the Fujita group introduced the crystalline sponge (CS) method, enabling x‐ray diffraction (XRD) analysis for small molecules that are difficult to crystallize [7]. This approach relies on trapping guest molecules within the cavities of porous or cage‐like host materials via multiple weak host‐guest interactions, and has proven effective for determining the structures of diverse non‐crystalline compounds [8]. Subsequently, an alignment strategy utilizing metal–organic frameworks (MOFs) was reported, facilitating XRD structural studies on molecules capable of binding within MOFs [9]. Wu et al. built upon this concept by incorporating pillar[5]arene rings into MOF architectures, enabling rapid structural elucidation of alkyl‐chain‐containing molecules [10]. A limitation of these methods, however, is their dependence on the confinement effects and intermolecular interactions provided by the cavity‐based structure. Strict requirements are thus imposed on the sizes and shapes of guest molecules, thereby restricting broader applicability. Tetraaryladamantanes have been proposed as molecular chaperones, in which a rigid, three‐dimensionally symmetric core promotes crystallization and peripheral aryl groups bind small molecules to form co‐crystals [11]. However, their applicability remains limited to a narrow range of guest polarity and molecular weight. In 2024, we reported a new co‐crystallization strategy, called “crystalline mate,” based on cyclic trinuclear complexes (CTCs), specifically the Ag^I^ pyrazolate Ag_3_Pz_3_ [12]. This electron‐deficient metallacycle acts as a crystalline mate, engaging in multiple non‐covalent interactions with Lewis‐basic sites on substrates to promote co‐crystal formation. Unlike porous frameworks, this crystalline mate adapts to guests, and thus does not depend on size or shape complementarity [13]. It has enabled the determination of the structures of both pure compounds and complex mixtures, without requiring tedious separation steps [14]. While this flexibility broadens the scope of co‐crystallization applications, it also raises a critical question: how can we quickly assess whether a given compound is compatible with such a crystalline mate?

Machine learning (ML) has emerged as a powerful data‐driven approach for applying artificial intelligence to complex problems in chemistry and materials science [15, 16, 17]. ML techniques are capable of capturing non‐obvious structure‐property relationships and enabling the prediction of properties for unexplored materials. ML has thus accelerated the discovery of new molecules and materials [18], and it shows promise in predicting co‐crystallization behavior [19, 20, 21]. However, the complexity of co‐crystal formation and the imbalance in existing datasets, which overwhelmingly contain positive “hits” hinder model generalizability. Consequently, models trained on small organic molecule datasets exhibit limited effectiveness in predicting co‐crystallization with CTCs.

**FIGURE 1:**
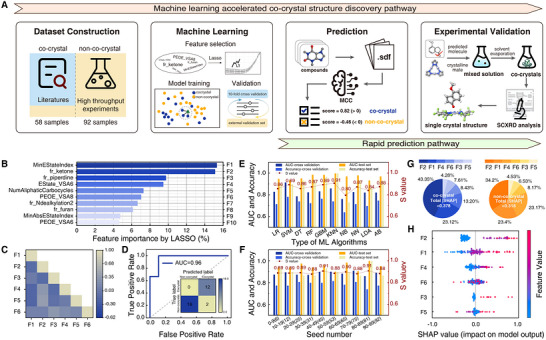
ML model for co‐crystal structure discovery based on an Ag_3_Pz_3_ crystalline mate. (A) Schematic workflow of the entire pathway and the rapid prediction pathway. (B) Relative importance ranking of the top 10 features with nonzero importance selected by the LASSO algorithm. (C) Pearson correlation heatmap of the top six‐ranked features. (D) Receiver operating characteristic (ROC) curve and confusion matrix of the KNN model trained with random seed 45 on the 30 external validation samples. (E) Performance evaluation of 10 different machine learning algorithms based on the weighted scoring function *S* using the optimal random seed. (F) Optimal random seed search for the KNN model trained with the top six ranked features. (G) The proportion of SHAP values for each feature in the “co‐crystal” and “non‐co‐crystal” categories, along with the total SHAP absolute values. (H) SHAP summary plot for the ML model.

Here, we present an ML‐accelerated workflow for the discovery of co‐crystals using the Ag_3_Pz_3_ crystalline mate, which enabled rapid prediction and experimental validation of over 100 co‐crystal structures. A high‐quality dataset was constructed from literature‐reported co‐crystals and compounds that were confirmed through high‐throughput experiments not to form co‐crystals. The ML algorithm and overall pipeline were optimized through feature engineering and benchmarking (Figure 1A). Multi‐dimensional validation confirmed that the model achieved over 95% prediction accuracy. When applied to a chemically diverse test set, the trained model identified 120 organic compounds with potential for co‐crystallizing with Ag_3_Pz_3_. Subsequent experimental single‐crystal growth and SCXRD validation yielded 114 successful co‐crystals. These compounds exhibited structural diversity—including liquid molecules, chain molecules, and cyclic bacteriocin macromolecules—highlighting both the strong generalizability of the ML model and the adaptability of the crystalline mate strategy.

Notably, the direct application of the model (rapid prediction pathway, Figure 1A) enabled efficient and straightforward discovery of co‐crystals, with predictions delivered within seconds. Subsequently, co‐crystallization can be attempted via solvent evaporation—typically requiring three or fewer solvent conditions—within 72 h. The simple structure of the Ag_3_Pz_3_ allows SCXRD data collection for the resulting co‐crystals to be completed within three hours. Our ML‐accelerated co‐crystal discovery process, crystalline mate method, thus complements and builds upon the crystalline sponge and porous materials methods, by being potentially faster, easier to implement, and more efficient.

## 2 Results and Discussion

### 2.1 ML Model Training

We constructed a high‐quality dataset derived from experimental results of Ag_3_Pz_3_ co‐crystal structures, comprising 48 successful co‐crystal structures obtained from the literature, along with 10 successful and 92 unsuccessful co‐crystal structures from high‐throughput experiments (Figures S1 and S2 for detailed structures). The 58 successful co‐crystal structures were used as positive samples in the ML model; all of these meet established crystallographic standards without any A‐level alerts. All 92 negative samples were selected based on stringent experimental criteria and underwent multiple rounds of rigorous verification to ensure data reliability and representativeness (Table S1).

A total of 208 molecular descriptors were calculated using the RDKit toolkit [22], covering properties such as molecular weight, topological polar surface area (TPSA), octanol‐water partition coefficient (LogP), counts of hydrogen bond donors and acceptors, number of rotatable bonds, and number of aromatic rings. Feature selection was performed using the least absolute shrinkage and selection operator (LASSO) regression [23]. LASSO is a linear regression method based on L1 regularization, which introduces an L1‐norm penalty term into the loss function to drive the coefficients of less relevant features to zero, thereby achieving feature selection [24]. In this study, LASSO was chosen because the RDKit‐derived descriptor space was high‐dimensional and potentially redundant. It enables efficient feature screening while retaining the physicochemical meaning of the original descriptors, thereby supporting both model generalization and interpretability. This process identified 20 features with nonzero coefficients, which were ranked by importance (Table S2 and Figure S3). The top 10 key features and their relative importance are presented in Figure 1B. Pearson correlation analysis revealed that all correlations among the selected features were below 0.7, supporting the robustness and scientific validity of the feature set (Figures 1C and S4). Since features F7 to F10 exhibited importance below 7.0%, and to avoid the curse of dimensionality associated with excessive features [25], we initially focused upon the top six features for algorithm evaluation and classification. This approach is consistent with previous studies indicating that six features often suffice for optimal model performance. These six selected features capture essential molecular characteristics, including functional groups, surface charge distribution, hydrophobicity, and van der Waals interaction range of organic molecules [26, 27, 28, 29]. Detailed descriptions of these features are provided in Table S2.

Using this dataset and the selected six features, we first evaluated the prediction accuracy of several common ML classification algorithms. The data were divided into an 80% training set and a 20% external validation set. To mitigate the impact of outliers and enhance robustness given the limited dataset size, model performance was evaluated by combining results from 10‐fold cross‐validation and external validation set using a weighted scoring function, S, defined as:

$$\begin{array}{cc} & S=w_{1}\times Acc_{-}cv+w_{2}\times AUC_{-}cv+w_{3}\times Acc_{-}test+w_{4}\\ & \;\times AUC_{-}test. \end{array}$$

Here, Acc_cv, AUC_cv refer to accuracy and area under the ROC curve from cross‐validation, and Acc_test and AUC_test refer to those from the external validation set. As AUC is generally more robust in assessing model quality, we assigned weights of *w*_1_ = 0.2, *w*_2_ = 0.3, *w*_3_ = 0.2, and *w*_4_ = 0.3. As shown in Figure 1E, among the classification algorithms tested (see Supporting Information and Tables S3 and S4 for details), including *k*‐nearest neighbors (KNN) [30], random forests (RF) [31], gradient boosting machine (GBM) [32], and so forth, the KNN algorithm [33] achieved the highest overall *S* value of *S* = 0.91. To mitigate the risk of data imbalance during the division of the external validation set, an optimized data partitioning strategy and seed selection were implemented. Specifically, 100 random seeds (0–99) were divided into 10 groups and evaluated sequentially using the KNN algorithm. For each group, the seed that produced the highest *S* value was identified, ultimately leading to the selection of the best‐performing seed as the optimal partition (Figure 1F). The model trained with random seed 45 achieved the highest *S* value (*S* = 0.91), corresponding to an AUC of 96% on the external validation set of 30 samples, with only two misclassifying samples (see Figure 1D). It also demonstrated strong generalization capability, with an average AUC of 90% and an average accuracy of 80% in 10‐fold cross‐validation (Table S5). Although random seed 81 also attained the highest *S* value in one group, seed 45 yielded more stable and balanced performance across metrics (see Table S5). Therefore, random seed 45 was selected for all subsequent model training and prediction.

Employing the KNN algorithm with random seed 45, we re‐evaluated the number of features. As shown in Figure S5, the *S* value reaches a maximum of 0.91 when using six features. Beyond this point, incorporating additional features during training did not lead to significant performance improvements, suggesting a dimensionality limit for this particular dataset. Based on the above benchmark results, the ML model was trained using the KNN algorithm with random seed 45 and six input features. The resulting model is designated as **MCC** (Model for Co‐Crystals).

### 2.2 ML Model Interpretability

SHAP (Shapley additive explanations) analysis [34, 35] was employed to interpret the **MCC** model and assess the contribution of individual features to predictions. Features F2 and F1 were identified as the most influential, with mean SHAP values of 0.33 and 0.19, respectively (Figure S6). The remaining features exhibited lower yet meaningful contributions, with SHAP values ranging from 0.05 to 0.12. To further investigate how these features influence co‐crystal formation between organic compounds and Ag_3_Pz_3_, we examined the SHAP summary plot (Figure 1H and Figures S7–S9), which offers a global interpretation of feature effects. The results indicate a positive correlation between the values of F2 and F3 and their corresponding SHAP values, suggesting that higher values of these features are associated with an increased probability of co‐crystal formation. Notably, F2 and F1 made the most substantial contribution to both predicted classes, underscoring their critical role in the model's decision‐making. We also compared the overall impact of features on both categories using total absolute SHAP values (|SHAP|). As shown in Figure 1G, the total |SHAP| value for “co‐crystal” samples (0.378) slightly exceeds that for “non‐co‐crystal” samples (0.318), indicating that feature influence is more pronounced in predicting co‐crystal formation. The SHAP force plot for selected molecules, detailed in Figures S10 and S11, illustrates how different features affect the co‐crystal and non‐co‐crystal feasibility of individual samples.

### 2.3 Model Prediction

We then applied **MCC** to predict the co‐crystal formation potential of previously untested organic molecules. A total of 5406 compounds were collected from the MedChemExpress database (Monmouth Junction, NJ, USA). The end‐to‐end prediction workflow using **MCC** was highly efficient, completing within seconds and generating output in a tabular format that included molecular structure images, SMILES strings, and corresponding prediction scores (see Figure S12 for an example and supplementary code for the complete document). Since the KNN model outputs binary classification scores of 0∼1, we applied a linear transformation to rescale the values to a more interpretable range from −1 to 1. Within this transformation scale, a prediction score > 0 indicates the potential for co‐crystal formation with Ag_3_Pz_3_, while a score < 0 suggests a low propensity for co‐crystallization. All predictions were completed within several seconds. From these predictions, we identified 1,206 compounds with high co‐crystal formation potential. To experimentally validate the practical applicability of the ML model, we selected 120 compounds from the positive predictions and 20 compounds from the negative range, covering different score intervals and diverse structural motifs, for subsequent co‐crystal experiments (Figure S13).

### 2.4 Experimental Validation

**FIGURE 2:**
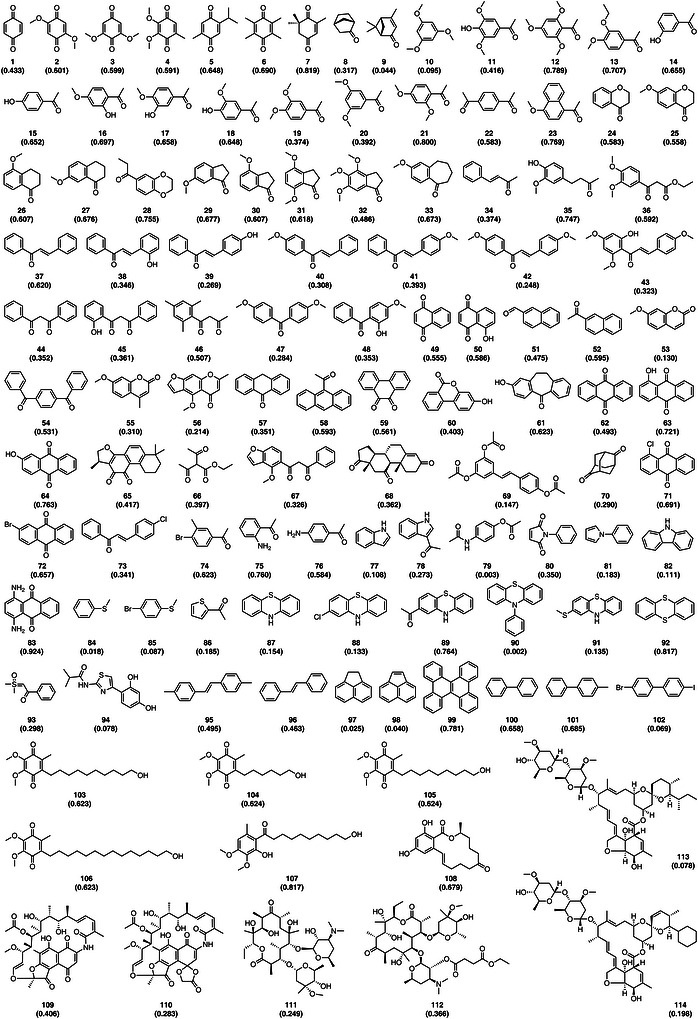
Chemical structures of organic compounds successfully co‐crystallized with Ag_3_Pz_3_ throughout **MCC** prediction and experimental validation. Each structure is labelled with its index number and corresponding prediction score.

To evaluate the real‐world predictive performance of the **MCC** and the general applicability of the crystalline mate strategy, we experimentally screened 120 candidate organic compounds from its prediction set. The selected molecules were chosen to cover the full range of predicted score intervals and, as far as possible, to include diverse heteroatoms, functional groups, conjugation patterns, and a broad molecular‐weight distribution (Figures S14−S16). These compounds spanned a range of structural complexity and were subjected to co‐crystallization with Ag_3_Pz_3_, which was synthesized through the reaction of Ag_2_O with 3,5‐bis(trifluoromethyl)pyrazole following an optimized procedure (see Supplementary Information for details). The co‐crystallization conditions are provided in Table S6. Crystallization was carried out using a conventional solvent evaporation method with common solvents including dichloromethane, methanol, acetonitrile, and hexane. Crystals typically formed within 72 h. As shown in Figure 2, 114 compounds successfully formed co‐crystals with Ag_3_Pz_3_, consistent with their **MCC** prediction scores. The six failures are listed in Figure S17 and Table S7 with an analysis of the possible reasons for the failures. Based on these experimental outcomes, the **MCC** achieved 95% accuracy in predicting co‐crystal formation, confirming the robustness and practical utility of this data‐driven approach for rapid identification of viable co‐crystal candidates. It should be noted that these failure cases are assessed only on the results under comparable crystallization conditions and do not rule out successful crystallization after optimizing conditions or applying alternative crystallization methods. Additionally, 20 organic compounds bringing different functional groups or heteratoms with a prediction score < 0 were selected for co‐crystallization trials. Under ambient conditions across three different kinds of solvents, no co‐crystals were obtained via solvent evaporation (Figure S18 and Table S8). This result demonstrates that **MCC** also achieves high accuracy in predicting compounds unlikely to form co‐crystals with Ag_3_Pz_3_.

Compounds **1–7**, benzoquinone derivatives with varied substituents, showed predicted scores of **0.433**–**0.819**, and all formed co‐crystals with Ag_3_Pz_3_. SCXRD analysis (Figures S19–S39 and Tables S9–S15) revealed that the carbonyl oxygen atoms on the benzoquinone scaffold serve as bridging sites, connecting adjacent Ag_3_Pz_3_ units to form the structural motif of the co‐crystals. Despite the common scaffold, variations in their prediction scores highlight the **MCC**’s sensitivity to subtle structural differences that correlate with co‐crystallization propensity. Notably, compound **7**, a volatile liquid natural product abundant in tea and aromatic plants, is difficult to crystallize. Yet its high prediction score (**0.819**) prompted us to obtain the first solid‐state crystal structure of the **Ag_3_Pz_3_·7** adduct (Figure 3A).

**FIGURE 3:**
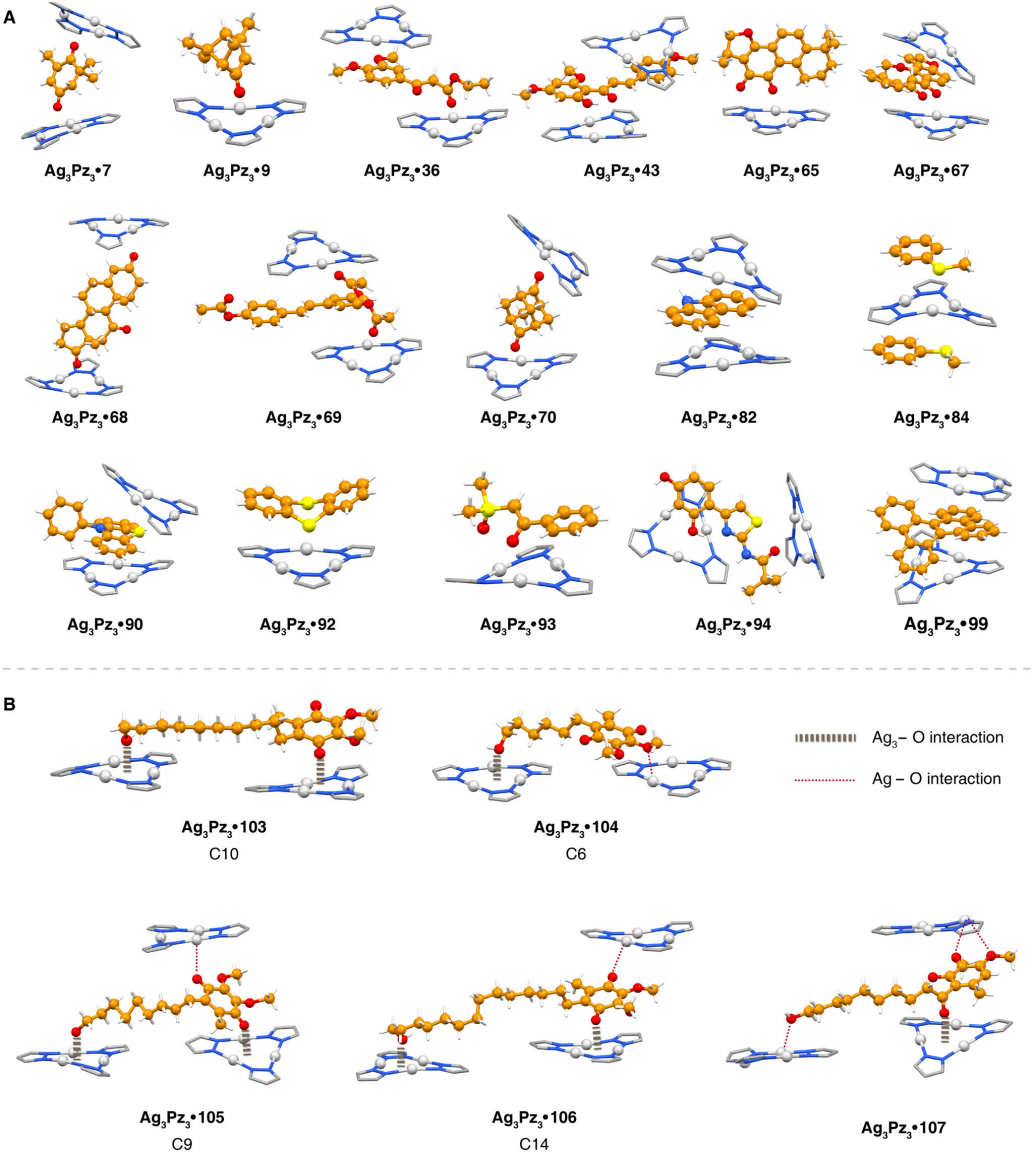
Representative co‐crystal structures of **Ag_3_Pz_3_·X**. (A) X = O/N/S containing organic compounds. (B) X = long‐chain‐containing Idebenone analogues. Ag_3_─O interactions and Ag─O weak coordination are shown as brown extended dashes and red dashes, respectively. Trifluoromethyl groups and H atoms in Ag_3_Pz_3_ are omitted for clarity. C, N, and Ag atoms in Ag_3_Pz_3_ are depicted in dark gray, light blue, and light gray, respectively; in organic molecules, C, O, N, S, and H atoms are shown in orange, red, light blue, yellow, and white, respectively. All crystal structures are presented in ball‐and‐stick style, thermal displacement parameters and structural disorder were provided in the supplementary materials.

We next explored oxygen‐containing organic molecules, including acetophenones, naphthoquinones, anthraquinones, indanones, benzophenones, chalcones, and flavonoid‐related derivatives (compounds **8–70**). Closely resembling the training set and aligning with F2 feature values, these compounds were predicted to crystallize readily, and all formed stable co‐crystals with Ag_3_Pz_3_ (Figures S40–S228 and Tables S16–S78). As shown in Figure 3A, compound **36** exhibits a unique combination of sandwich‐like binding and single‐site binding modes. Flavokawain A (**43**), a volatile anticancer natural product, and cryptotanshinone (**65**), a diterpene quinone from Salvia miltiorrhiza Bge, both yielded well‐defined co‐crystals with Ag_3_Pz_3_. Pongamol (**67**), a natural flavonoid from Pongamia pinnata with antioxidant and anti‐inflammatory activity, and adrenosterone (**68**), an adrenal steroid, also co‐crystallized with Ag_3_Pz_3_, the latter allowing unambiguous assignment of absolute configuration. Resveratrol, a natural polyphenol widely applied in blood glucose regulation and skin repair, initially failed to cocrystallize with Ag_3_Pz_3_ in high‐throughput screening experiments and was thus labeled as a negative sample in the dataset. However, its acetylated derivative (**69**) received a positive prediction score (0.147) from **MCC**. Experiments confirmed its ability to form a co‐crystal with Ag_3_Pz_3_, exhibiting a combined single‐point and sandwich binding mode. Notably, this result is in line with the importance of ML feature F2 in the prediction model, supporting the relevance of acetylation‐related structural changes in co‐crystal formation. It is also consistent with our recently reported acetylation‐tagging‐assisted co‐crystallization strategy [36, 37].

Compound **70**, with a bulky adamantane core and large steric hindrance, co‐crystallized with Ag_3_Pz_3_ successfully, underscoring the structural adaptability and molecular recognition capability of Ag_3_Pz_3_ crystalline mate.

Encouraged by **MCC** predictions, we further explored halogen‐, nitrogen‐, and sulfur‐containing molecules (compounds **71–94**, Figures S229–S300 and Tables S79–S102). Nitrogen‐ or sulfur‐heterocycles (e.g., **82**, **84**, **90**, and **92**, see Figure 3A) typically adopted a sandwich‐like or S‐site binding mode with Ag_3_Pz_3_, which correlated to their aromaticity and the electronegativity of sulfur. Of particular interest is compound **84**, a volatile liquid benzyl sulfide, previously resistant to crystallization, which yielded a high‐quality co‐crystal with Ag_3_Pz_3_. Compounds bearing multiple heteroatoms (e.g., **93** and **94**) exhibit greater conformational flexibility and offer several potential interaction sites. Both prediction and experimental results confirm their ability to co‐crystallize with Ag_3_Pz_3_.

Conjugated systems (compounds **95–102**) lacking electronegative heteroatoms were likewise predicted and experimentally confirmed to form co‐crystals with Ag_3_Pz_3_ (Figures S301–S324 and Tables S103–S110). Dibenzo[g,p]chrysene (**99**) assembled into a sandwich‐like supramolecular architecture, stabilized by strong π–π stacking interactions with two opposing Ag_3_Pz_3_ units (Figure 3A). Whereas trans‐stilbene (**96**) adopted a distinct co‐crystal structure involving its central C═C bond and the planar Ag coordination motif (Figures S304 and S305). These diverse binding modes underscore the remarkable structural adaptability of Ag_3_Pz_3_, which can fine‐tune molecular orientation and coordination angles to accommodate varied interaction sites and shapes [38].

Idebenone (**103**), a synthetic coenzyme Q10 analogue used in neurodegenerative and mitochondrial disorders [39], carries a benzoquinone core with a flexible C10 alkyl chain terminating in a hydroxyl group. This structural flexibility usually leads to poor crystallinity. **MCC**, however, assigned idebenone a high predicted score of 0.62. High‐quality co‐crystals of **Ag_3_Pz_3_•103** were successfully obtained and resolved by SCXRD (Figure 3B, Figures S325–S327, and Table S111). The structure revealed a dual‐binding mode, with both the benzoquinone core and the terminal hydroxyl group engaging distinct Ag_3_Pz_3_ units. This “end‐to‐end anchoring” restricts chain flexibility and promotes crystallization.

To probe chain‐length effects, we synthesized idebenone analogues with C6, C9, and C14 side chain (**104**–**106**, Supporting Information and Figures S328–S329, S333–S334, and S338–S339). All three analogues received positive prediction scores and successfully formed co‐crystals with Ag_3_Pz_3_ (Figures 3B, S330–S332, S335–S337, and S340–S342 and Tables S112–S114). In each co‐crystal structure, the terminal hydroxyl group anchored to the Ag_3_ center, while the benzoquinone core binding mode varied with chain length: **104** bound a single Ag atom via one methoxy oxygen; **105** engaged one Ag_3_ unit through a carbonyl–methoxy pair; and **106** coordinated two distinct Ag_3_ units through multiple carbonyl and methoxy groups, forming an extended anchoring network (Figure 3B). These differences reflect increased conformational freedom in long chains, requiring stronger π‐acidic stabilization. Extending this strategy, a predicted long‐chain analogue (**107**) with a distinct core also formed co‐crystals with Ag_3_Pz_3_, and its structure **Ag_3_Pz_3_·107** was confirmed by SCXRD (Figures 3B, S343–S345, and Table S115).

**FIGURE 4:**
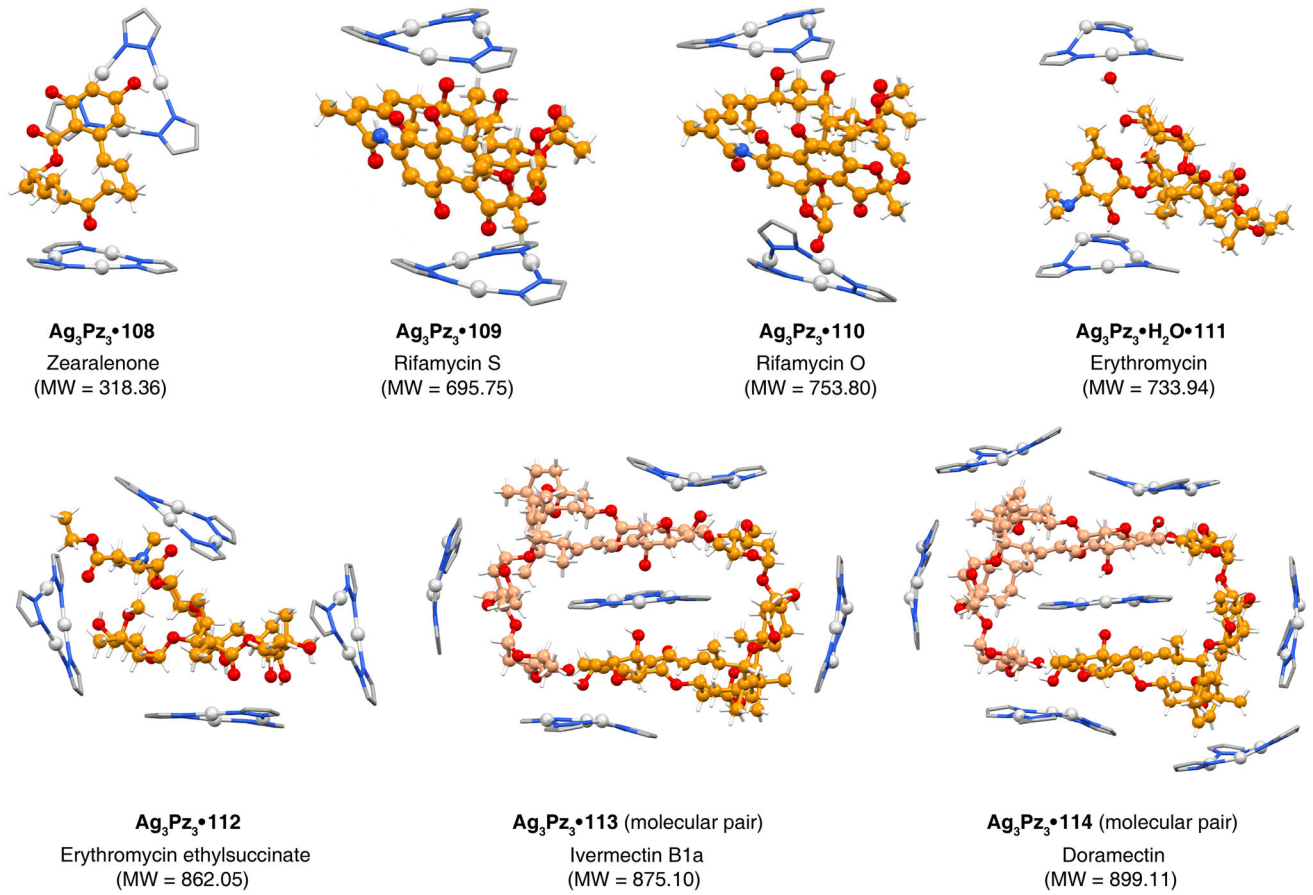
Co‐crystal structures of Ag_3_Pz_3_ with macrolide compounds. Trifluoromethyl groups and H atoms in Ag_3_Pz_3_ are omitted for clarity. C, N, and Ag atoms in Ag_3_Pz_3_ are shown in dark grey, light blue, and light grey, respectively; C, O, N, and H atoms in the organic molecules are depicted in orange, red, light blue, and white, respectively. In **Ag_3_Pz_3_·113** and **Ag_3_Pz_3_·114**, the C atoms from distinct organic molecules are colored in orange and pink, respectively. Common names and molecular weights (MW) of macrolide compounds are provided.

Macrolides, molecules commonly found in “mycin” natural products [40], are widely used in medicine [41, 42] but present persistent challenges in single‐crystal cultivation and structural elucidation due to their structural complexity and conformational flexibility. Inspired by the successful co‐crystallization of compound **33**, an oxygenated cycloalkane with a prediction score of **0.673**, we extended our investigation to macrolide compounds. Zearalenone (**108**, MW = **318.36**, score = **0.679**), an estrogenic mycotoxin found in contaminated grains, contains a hydroquinone ring and a carbonyl‐functionalized side chain. We successfully obtained the co‐crystal **Ag_3_Pz_3_·108** (Figures 4, S346–S348, and Table S116), whose SCXRD structure revealed a dual anchoring mechanism: a macrocyclic carbonyl coordinated one Ag_3_Pz_3_ unit while the aromatic core engaged another via π–π interactions, reducing torsional motion. Rifamycin S (**109**, MW = **695.75**, score = **0.406**) and Rifamycin O (**110**, MW = 753.80, score = 0.283), polyketide natural products synthesized by streptomyces and key intermediates in rifampicin synthesis [43], also co‐crystallized with Ag_3_Pz_3_ (Figures 4, S349–S354, and Tables S117 and S118). As illustrated in Figure 4, both **109** and **110** interact with a single Ag_3_Pz_3_ unit (the upper unit) through two adjacent hydroxyl groups on their shared backbone. However, their carbonyl binding mode differed: In **109**, two adjacent carbonyl groups engage with one Ag_3_Pz_3_ unit, whereas in **110**, only a single carbonyl group from the additional five‐membered ring serves as the binding site. This subtle variation accounts for both the divergence in prediction scores and the distinct binding behaviors observed.

Clinically important erythromycin [44, 45] (**111**; MW = **733.94**, score = **0.249**) and its prodrug erythromycin ethylsuccinate [46, 47] (**112**; MW = **862.05**, score = **0.366**) both formed co‐crystals with Ag_3_Pz_3_ (Figures 4, S355–S360, and Tables S119 and S120). Erythromycin interacted with one Ag_3_Pz_3_ unit via synergistic engagement of a hydroxyl group on one of its glycosidic moieties and an adjacent nitrogen atom. In addition, another hydroxyl group participates in an indirect interaction with a second Ag_3_Pz_3_ unit through hydrogen bonding mediated by an interstitial water molecule. More flexible ethylsuccinate derivative adopted a multi‐site binding mode via its long‐chain ester, glycosidic units, and macrolide core, yielding the first reported single crystal structure of erythromycin ethylsuccinate.

The Avermectin family, produced by Streptomyces avermitilis [48], comprises 16 structurally related macrolide natural products known for their broad‐spectrum and potent antiparasitic activity. Two representative members: the semi‐synthetic derivative ivermectin B1a (**113**; MW = **875.10**, score = **0.078**) and the synthetically optimized doramectin (**114**; MW = **899.11**, score = **0.198**), co‐crystallized with Ag_3_Pz_3_ successfully (Figures 4, S361–S366, and Tables S121 and S122). Structural analysis revealed that both adopt a bent conformation that dimerizes head‐to‐tail, encapsulating a central Ag_3_Pz_3_ trinuclear complex within a well‐defined cavity. Several peripheral Ag_3_Pz_3_ units reinforced this architecture through weaker secondary interactions with the organic backbone. These multivalent contacts further restrict the conformational flexibility and torsional motion of the large macrocycles. Unlike porous‐framework methods that trap molecules into an ordered arrangement [7, 8, 9, 10], our strategy generates a “clamped” supramolecular architecture through cooperative molecular reorganization and multi‐site Ag coordination, enabling high‐quality crystals or otherwise intractable macrolides.

The effectiveness of the ML–accelerated crystalline mate strategy was further experimentally validated under competitive crystallization conditions. Compounds **87** and **103** received predicted scores of 0.154 and 0.623, respectively. Despite their distinct structure, they were combined with Ag_3_Pz_3_ and produced three separate single crystals within a unified crystalline system (see supporting information and Figure S367 for details). As shown in Figure S368, three single crystal morphologies were visually distinguishable and identified as **Ag_3_Pz_3_·87’**, **Ag_3_Pz_3_·103**, and the ternary co‐crystal **Ag_3_Pz_3_·87·103** (Figures S369–S372 and Table S123). Notably, **Ag_3_Pz_3_·87’** is a new co‐crystal structure distinct from **Ag_3_Pz_3_·87**, featuring an asymmetric unit in which the stoichiometric ratio of Ag_3_Pz_3_ to compound **87** is 2:1 (Figures S373–S375 and Table S124). These results demonstrate that even in competitive crystallization conditions, Ag_3_Pz_3_ can interact with different target molecules, enabling the formation of higher‐order co‐crystal architectures.

## 3 Conclusion

In summary, we present an ML‐assisted strategy for structure determination via co‐crystallization using Ag_3_Pz_3_ as a universal crystalline mate. The **MCC** model, trained on a curated dataset and validated through multidimensional assessment, achieved high predictive accuracy (weighted average ***S* = 0.91**) and successfully identified 1206 organic compounds with high co‐crystallization potential. Experimental validation confirmed that 114 of 120 predicted compounds successfully formed co‐crystals with Ag_3_Pz_3_, corresponding to a 95% success rate and demonstrating strong model robustness. This approach enabled successful structural elucidation of diverse and challenging molecular classes—including stereochemically complex, long‐chain, and macrocyclic natural products. Many of these compounds exceed the structural complexity of the training set, underscoring the model's extrapolative capability. Notably, the workflow offers rapid prediction (within seconds), simplicity (using common solvents and solvent evaporation), and broad applicability (routinely producing high‐quality crystals suitable for SCXRD analysis within hours, without constraint of guest molecule size and shape) in most laboratory settings. Currently, the strategy is limited to Ag_3_‐based crystalline mate systems due to the scarcity of alternative crystalline mates and reported co‐crystal structures involving different crystalline mates. However, we anticipate that our approach may be expanded through the discovery of new crystalline mates and corresponding co‐crystal structures, potentially offering a generalizable and efficient solution for the structural analysis of challenging molecules.

## Author Contributions

**Cui‐Zhou Luan**: methodology, investigation, writing – original draft, validation, visualization, formal analysis, data curation. **Xue‐Zhi Wang**: validation, formal analysis, data curation. **Jian‐Guo Song**: formal analysis, data curation. **Yu Gu**: investigation, methodology, validation, formal analysis. **Jing Wu**: validation, formal analysis, data curation. **Ye‐Ting Wang**: investigation, validation, formal analysis, data curation. **Jin‐Feng Liang**: investigation, validation, data curation. **Jia‐Le Rao**: investigation, validation, data curation. **Mo Xie**: conceptualization, funding acquisition, writing – original draft, methodology, visualization, writing – review and editing, supervision. **Jonathan R. Nitschke**: writing – review and editing, resources. **Dan Li**: conceptualization, funding acquisition, methodology, writing ‐ review and editing, software, project administration, resources.

## Conflicts of Interest

The authors declare no conflicts of interest.

## Supporting information




**Supporting File 1**: Full experimental materials and procedures, characterization data, crystal data, and some supporting figures and tables are included in the Supporting Information. The code and training dataset used in this study are provided in a separate supporting material named supplementary_materials_MCC_code.zip and are also publicly accessible on GitHub (https://github.com/Cuizhou‐Luan/MCC/tree/main). The cif files of CCDC deposition numbers 2501752–2501821 and 2501843–2501888 containing the crystallographic data for this paper are packaged in supplementary_materials_crystallographic data.zip.

**Supporting File 2**: anie72462‐sup‐0002‐DataFile.zip.

## Data Availability

The data that supports the findings of this study are available in the supplementary material of this article.
