# Supplementary material for: Machine Learning Accelerates Crystallization for Structure Determination
Source: Angew Chem Int Ed Engl. 2026 May 4;65(25):e1218503. doi: 10.1002/anie.1218503 (PMC13266958; doi:10.1002/anie.1218503)
Supplement: Supplementary file 1 — Supporting File 1: Full experimental materials and procedures, characterization data, crystal data, and some supporting figures and tables are included in the Supporting Information. The code and training dataset used in this study are provided in a separate supporting material named supplementary_materials_MCC_code.zip and are also publicly accessible on GitHub (https://github.com/Cuizhou‐Luan/MCC/tree/main). The cif files of CCDC deposition numbers 2501752–2501821 and 2501843–2501888 containing the crystallographic data for this paper are packaged in supplementary_materials_crystallographic data.zip. [file ANIE-65-e1218503-s001.pdf]

Supplementary Information

Machine Learning Accelerates Crystallization for Structure Determination

Cui-Zhou Luan,[a] Xue-Zhi Wang,[a][e] Jian-Guo Song,[b] Yu Gu,[c] Jing Wu,[a] Ye-Ting Wang,[a] Jin-Feng Liang,[a] Jia-Le Rao,[a] Mo Xie,*[a] Jonathan R. Nitschke,*[d] and Dan Li*[a]

[a] C.-Z. Luan, Dr. X.-Z. Wang, J. Wu, Y.-T. Wang, J.-F. Liang, J.-L. Rao, Dr. M. Xie, Prof. D. Li
State Key Laboratory of Bioactive Molecules and Druggability Assessment, College of Chemistry and Materials Science, Guangdong Provincial Key Laboratory of Supramolecular Coordination Chemistry
Jinan University
Guangzhou 510632, P. R. China
E-mail: xiemo@jnu.edu.cn, danli@jnu.edu.cn

[b] Dr. J.-G. Song
State Key Laboratory of Bioactive Molecules and Druggability Assessment
Jinan University
Guangzhou 510632, P. R. China

[c] Dr. Y. Gu
College of Physics and Optoelectronic Engineering
Jinan University
Guangzhou 510632, P. R. China

[d] Prof. J. R. Nitschke
Yusuf Hamied Department of Chemistry
University of Cambridge
Cambridge, UK
E-mail: jrn34@cam.ac.uk

[e] Dr. X.-Z. Wang
Department of Ultrasound, Institute of Ultrasound in Musculoskeletal Sports Medicine
The Affiliated Guangdong Second Provincial General Hospital, Jinan University
Guangzhou 510317, P. R. China

Table of Contents (445 pages)

1. **Materials and methods** 3
2. **Machine learning techniques** 7
3. **Supplementary figures and tables** 12
4. **Co-crystallization experiments** 40
5. **Supplementary references** 445

1. Materials and methods

Materials and chemicals

All starting materials, including dichloromethane (DCM), methanol (MeOH), acetonitrile (MeCN), n-hexane (n-Hex), anhydrous ether, cyclohexane (c-Hex), ethyl acetate (EA), acetone (Me_2CO), toluene (PhMe), N,N-dimethylformamide (DMF), dimethyl sulfoxide (DMSO), silver(I) oxide (Ag_2O), 3,5-bis(trifluoromethyl)-1H-pyrazole ([3,5-$(CF_3)_2$Pz]H), potassium hydroxide (KOH), silver nitrate ($AgNO_3$), ammonium persulfate ($(NH_4)_2S_2O_8$), sodium bicarbonate ($NaHCO_3$), sodium chloride (NaCl), anhydrous sodium sulfate (Na_2SO_4), 7-hydroxyheptanoic acid, 10-hydroxydecanoic acid, 15-hydroxypentadecanoic acid, and the target compounds, were purchased from commercial suppliers and used without further purification unless otherwise specified. The target compounds (**104-106**) were synthesized by following previously reported procedures for structurally related idebenone derivatives.[1,2] The trinuclear silver complex with 3,5-bis(trifluoromethyl)-1H-pyrazole ligands (Ag_3[3,5-$(CF_3)_2$Pz]$_3$, Ag_3Pz_3) was prepared according to reported methods[3].

Instrumentation

Column chromatography was carried out on silica gel (100-200 mesh, Qingdao Marine Chemical Plant, Qingdao, China). Proton nuclear magnetic resonance (^{1}H NMR) spectra were recorded on a Bruker Avance III HD 400 MHz spectrometer. Chemical shifts were reported in parts per million (ppm), referenced to 0.0 ppm for tetramethylsilane (TMS). Gas chromatography-mass spectrometry (GC-MS) analyses were performed on an Agilent 7890B-5977B gas chromatograph-mass spectrometer, with acetonitrile used as the solvent. Optical photos of single crystals were recorded using a CNOPTEC SZ680 continuous zoom stereomicroscope coupled to a digital camera and a PC (video monitor).

Crystallography study

Single-crystal X-ray diffraction (SCXRD) data of **Ag_3Pz_3·1-114**, **Ag_3Pz_3·87·103**, and **Ag_3Pz_3·87'** were collected using an Oxford Diffraction XtaLAB (Rigaku, Japan, Cu Kα, λ = 1.54178 Å) equipped with a monochromator and CCD plate detector (CrysAlisPro CCD, Oxford Diffraction

Ltd) at 100 K. The structures were determined by intrinsic phasing (SHELXT 2018/2)[4] and refined by full-matrix least-squares on $\boldsymbol{F}^2$ (SHELXL-2018/2)[5] using the Olex2[6] software package. The disordered non-coordinated solvents were removed using the SQUEEZE program of PLATON[7]. All non-hydrogen atoms were refined anisotropically. Hydrogen atom positions were calculated geometrically and refined using the riding model.

General responses on CheckCIF alerts

All CheckCIF alerts were carefully examined and adequately responded to in the CheckCIF report. The corresponding responses have also been incorporated into the CIF files and presented under each SCXRD data table.

Synthesis of 6-(6-Hydroxyhexyl)-2,3-dimethoxy-5-methyl-1,4-benzoquinone (104)

KOH (0.31 g, 0.0055 mol) was dissolved in H_2O (7.5 mL), followed by the addition of 7-hydroxyheptanoic acid (0.804 g, 0.0055 mol). The resulting mixture was stirred in a water bath at 70 °C for 30 min. An aqueous solution of $AgNO_3$ (0.90 g, 0.00525 mol in 5 mL H_2O) was added dropwise to the above solution, and stirring was continued for an additional 20 min. Separately, 2,3-dimethoxy-5-methyl-1,4-benzoquinone (0.91 g, 0.005 mol) was dissolved in MeCN (16 mL) and added dropwise to the reaction mixture. Stirring was maintained at 70 °C for another 30 min. A solution of $(NH_4)_2S_2O_8$ (2.40 g, 0.0105 mol in 3.5 mL H_2O) was added in three portions at 15 min intervals, and the reaction was continued at 70 °C for a total of 1 h 15 min. Upon completion, the reaction mixture was extracted with EA (3 × 20 mL). The combined organic layers were washed twice with H_2O, twice with saturated $NaHCO_3$ solution, and once with saturated NaCl solution. The organic phase was dried over anhydrous Na_2SO_4, filtered, and concentrated under reduced pressure to afford a crude residue. The product was purified by column chromatography and characterized by ^{1}H NMR spectroscopy and GC-MS analysis (1.18 g, yield 76%). The ^{1}H NMR spectrum and GC-MS data for compound **104** are presented in the following sections. ^{1}H NMR (400 MHz, DMSO-d_6) δ (ppm): 4.32 (t, J = 5.2 Hz, 1H), 3.87 (s, 6H), 3.39 – 3.34 (m, 2H), 2.37 (t, J = 7.2 Hz, 2H), 1.93 (s, 3H), 1.42 – 1.27 (m, 8H).

Synthesis of 6-(9-Hydroxynonyl)-2,3-dimethoxy-5-methyl-1,4-benzoquinone (105)

KOH (0.31 g, 0.0055 mol) was dissolved in H_2O (7.5 mL), followed by the addition of 10-hydroxydecanoic acid (1.035 g, 0.0055 mol). The resulting mixture was stirred in a water bath at 70 °C for 30 min. An aqueous solution of $AgNO_3$ (0.90 g, 0.00525 mol in 5 mL H_2O) was added dropwise to the above solution, and stirring was continued for an additional 20 min. Separately, 2,3-dimethoxy-5-methyl-1,4-benzoquinone (0.91 g, 0.005 mol) was dissolved in MeCN (16 mL) and added dropwise to the reaction mixture. Stirring was maintained at 70 °C for another 30 min. A solution of $(NH_4)_2S_2O_8$ (2.40 g, 0.0105 mol in 3.5 mL H_2O) was added in three portions at 15 min intervals, and the reaction was continued at 70 °C for a total of 1 h 15 min. Upon completion, the reaction mixture was extracted with EA (3 × 20 mL). The combined organic layers were washed twice with H_2O, twice with saturated $NaHCO_3$ solution, and once with saturated NaCl solution. The organic phase was dried over anhydrous Na_2SO_4, filtered, and concentrated under reduced pressure to afford a crude residue. The product was purified by column chromatography and characterized by ^{1}H NMR spectroscopy and GC-MS analysis (1.52 g, yield 85%). The ^{1}H NMR spectrum and GC-MS data for compound **105** are presented in the following sections. ^{1}H NMR (400 MHz, DMSO-d_6) δ (ppm): 4.31 (t, J = 5.1 Hz, 1H), 3.87 (s, 6H), 3.40 – 3.34 (m, 2H), 2.37 (t, J = 7.1 Hz, 2H), 1.92 (s, 3H), 1.39 (t, J = 6.7 Hz, 2H), 1.27 (d, J = 16.5 Hz, 12H).

Synthesis of 6-(14-Hydroxytetradecyl)-2,3-dimethoxy-5-methyl-1,4-benzoquinone (106)

KOH (0.31 g, 0.0055 mol) was dissolved in H_2O (7.5 mL), followed by the addition of 15-hydroxypentadecanoic acid (1.421 g, 0.0055 mol). The resulting mixture was stirred in a water bath at 70 °C for 30 min. An aqueous solution of $AgNO_3$ (0.90 g, 0.00525 mol in 5 mL H_2O) was added dropwise to the above solution, and stirring was continued for an additional 20 min. Separately, 2,3-dimethoxy-5-methyl-1,4-benzoquinone (0.91 g, 0.005 mol) was dissolved in MeCN (16 mL) and added dropwise to the reaction mixture. Stirring was maintained at 70 °C for another 30 min. A solution of $(NH_4)_2S_2O_8$ (2.40 g, 0.0105 mol in 3.5 mL H_2O) was added in three portions at 15 min intervals, and the reaction was continued at 70 °C for a total of 1 h 15 min. Upon completion, the reaction mixture was extracted with EA (3 × 20 mL). The combined organic layers were washed twice with H_2O, twice with saturated $NaHCO_3$ solution, and once with saturated NaCl solution. The organic phase was dried over anhydrous Na_2SO_4, filtered, and

concentrated under reduced pressure to afford a crude residue. The product was purified by column chromatography and characterized by ^{1}H NMR spectroscopy and GC-MS analysis (1.56 g, yield 72%). The ^{1}H NMR spectrum and GC-MS data for compound **106** are presented in the following sections. ^{1}H NMR (400 MHz, DMSO-d_6) δ (ppm): 4.30 (t, J = 5.2 Hz, 1H), 3.87 (s, 6H), 3.39 – 3.34 (m, 2H), 2.37 (t, J = 7.2 Hz, 2H), 1.92 (s, 3H), 1.41 – 1.36 (m, 2H), 1.23 (s, 22H).

2. Machine learning techniques

Model training details

All machine learning models in this study followed a unified training, validation, and evaluation pipeline to ensure fairness and comparability across different algorithms under identical experimental conditions. Considering the limited size of the molecular dataset and the potential sensitivity of model performance to specific data splits, the model training procedure was designed with an emphasis on generalization assessment and robustness analysis. Specifically, based on prior feature importance analysis, the top six most relevant molecular descriptors were selected from the candidate feature pool as model inputs. This fixed number of features was consistently applied across all models and random seed experiments to control model complexity and reduce the risk of overfitting under small-sample conditions. Subsequently, the dataset was divided into a training set (80%) and a test set (20%) using stratified random sampling to preserve class proportions across different splits. Prior to model training, all input features were standardized. The scaling parameters were fitted exclusively on the training set and then applied to the corresponding test set to prevent information leakage. All models were trained and validated on the training set using 10-fold cross-validation, and their generalization performance was evaluated on an independent test set.

Hyperparameter optimization strategy

The hyperparameters of all machine learning models were systematically optimized using grid search in combination with cross-validation. For most models, 10-fold cross-validation was employed, whereas for neural network (NN) and linear discriminant analysis (LDA) models, 5-fold cross-validation was used to balance computational efficiency and training stability under the limited sample size. For each model, only the core hyperparameters that critically influence model complexity or learning behavior were included in the search space, while the remaining parameters were kept at their default values to avoid introducing unnecessary search noise under small-sample conditions. The hyperparameter optimization process was independently performed for each random seed, with the area under the receiver operating characteristic curve (ROC-AUC) adopted as the primary optimization metric. This metric provides a robust assessment of model

discriminative ability, particularly in the presence of class imbalance, and was used solely for model selection without altering the original class distribution of the dataset. Specifically, the hyperparameter search for each model was conducted as follows:

Logistic Regression (LR): For LR, hyperparameter optimization was performed using a two-part grid search corresponding to the two types of regularization penalties. For L1 regularization, the hyperparameter 'C' (inverse of regularization strength) was varied over {0.01, 0.1, 1, 10, 100}, and the 'solver' was selected from {liblinear, saga}, with 'max_iter' fixed at 1000; for L2 regularization, 'C' was similarly varied over {0.01, 0.1, 1, 10, 100}, and 'solver' was chosen from {newton-cg, lbfgs, sag}, again with 'max_iter' fixed at 1000. The search was conducted using 10-fold cross-validation, with ROC-AUC as the scoring metric.

Support Vector Machine (SVM): For SVM, hyperparameter optimization was performed using a grid search over key parameters. The regularization parameter 'C' was varied over {0.1, 1, 10, 100}, the 'kernel' was selected from {linear, rbf}, and the kernel coefficient 'gamma' was explored over {scale, auto, 0.1, 1}. Probability estimation was enabled ('probability' = True) to allow calculation of ROC-AUC. The search was conducted using 10-fold cross-validation, with ROC-AUC as the scoring metric.

Decision Tree (DT): For DT, hyperparameter optimization was conducted using a grid search over key tree parameters. The splitting criterion 'criterion' was selected from {gini, entropy}, the maximum tree depth 'max_depth' was varied over {None, 3, 5, 7, 10}, the minimum number of samples required to split a node 'min_samples_split' was explored over {2, 5, 10}, and the minimum number of samples required at a leaf node 'min_samples_leaf' was varied over {1, 2, 4}. The number of features considered for each split 'max_features' was selected from {sqrt, None}. The search was performed using 10-fold cross-validation, with ROC-AUC as the scoring metric.

Random Forest (RF): For RF, hyperparameter optimization was performed using a grid search over key ensemble parameters. The number of trees 'n_estimators' was varied over {50, 100, 200}, the maximum tree depth 'max_depth' over {None, 10, 20, 30}, the minimum number of samples

required to split a node 'min_samples_split' over {2, 5}, and the minimum number of samples required at a leaf node 'min_samples_leaf' over {1, 2}. The number of features considered for each split 'max_features' was selected from {sqrt, None}, and bootstrap sampling 'bootstrap' was explored as {True, False}. The search was conducted using 10-fold cross-validation, with ROC-AUC as the scoring metric.

Gradient Boosting Machine (GBM): For GBM, hyperparameter optimization was performed using a grid search over key boosting parameters. The number of boosting stages 'n_estimators' was varied over {50, 100, 200}, the learning rate 'learning_rate' over {0.01, 0.1, 0.2}, the maximum tree depth 'max_depth' over {3, 5, 7}, the minimum number of samples required to split a node 'min_samples_split' over {2, 5}, and the minimum number of samples required at a leaf node 'min_samples_leaf' over {1, 2}. The number of features considered for each split 'max_features' was selected from {sqrt, None}, and the subsample fraction 'subsample' over {0.8, 1.0}. The search was conducted using 10-fold cross-validation, with ROC-AUC as the scoring metric.

K-Nearest Neighbors (KNN): For KNN, hyperparameter optimization was performed using a grid search over key neighbor parameters. The number of neighbors 'n_neighbors' was varied over {3, 5, 7, 9, 11, 13, 15} (odd numbers were chosen to avoid tie votes), the weight function 'weights' was selected from {uniform, distance}, and the distance metric 'p' was explored over {1, 2}, corresponding to Manhattan and Euclidean distances, respectively. The search was conducted using 10-fold cross-validation, with ROC-AUC as the scoring metric.

Naive Bayes (NB): For NB, hyperparameter optimization was performed using a grid search over the variance smoothing parameter 'var_smoothing', which was varied logarithmically from 1 (10^0) to 10^{-9} over 100 points. This parameter helps to improve numerical stability and avoid zero variance issues. The search was conducted using 10-fold cross-validation, with ROC-AUC as the scoring metric.

Neural Network (NN): For NN, hyperparameter optimization was performed using a grid search over key multi-layer perceptron (MLP) parameters. The hidden layer architectures

'mlp__hidden_layer_sizes' were varied among {(50,), (100,), (50, 50), (100, 50)}, the activation function 'mlp__activation' was selected from {relu, tanh}, the L2 regularization term 'mlp__alpha' was varied over {0.0001, 0.001, 0.01}, the initial learning rate 'mlp__learning_rate_init' was explored over {0.001, 0.01}, and the batch size 'mlp__batch_size' over {32, 64}. The search was conducted using 5-fold cross-validation, with ROC-AUC as the scoring metric.

Linear Discriminant Analysis (LDA): For LDA, hyperparameter optimization was performed using a grid search over key discriminant analysis parameters. The solver 'lda__solver' was selected from {svd, lsqr, eigen}, the shrinkage parameter 'lda__shrinkage' from {None, auto, 0.1, 0.5, 0.9}, and the tolerance for stopping criteria 'lda__tol' over {1e-4, 1e-3, 1e-2}. The search was conducted using 5-fold cross-validation, with ROC-AUC as the scoring metric, balancing computational efficiency and training stability given the limited dataset size.

AdaBoost (AB): For AdaBoost, hyperparameter optimization was performed using a grid search over key boosting parameters. The number of boosting stages 'n_estimators' was varied over {50, 100, 200}, the learning rate 'learning_rate' over {0.01, 0.1, 1.0}, and the maximum depth of the base decision tree estimator 'base_estimator__max_depth' over {1, 2, 3} to control base learner complexity. A decision tree stump was used as the base estimator. The search was conducted using 10-fold cross-validation, with ROC-AUC as the scoring metric.

Model evaluation and scoring strategy

Given the limited dataset size, reliance on a single evaluation metric or a single data split may not adequately reflect the true performance of a model. Therefore, model performance was comprehensively assessed by integrating cross-validation results with external test set evaluations. For each model, classification accuracy (Acc_cv) and ROC-AUC (AUC_cv) obtained from ten-fold cross-validation were calculated, along with classification accuracy (Acc_test) and ROC-AUC (AUC_test) on the external test set. Based on these metrics, a weighted composite scoring function S was defined as:

$$S = w_1 \times Acc\_cv + w_2 \times AUC\_cv + w_3 \times Acc\_test + w_4 \times AUC\_test,$$

$$\text{where } w_1 = 0.2, w_2 = 0.3, w_3 = 0.2, w_4 = 0.3$$

This scoring function was used solely for model performance evaluation and comparison and was not involved in model training or hyperparameter optimization.

Repeated random seed strategy

Given that model performance on small datasets may be sensitive to the specific training–test split and that class imbalance may introduce incidental bias under a single data partition, the complete workflow of model training, hyperparameter optimization, and performance evaluation was repeated under 100 different random seed conditions. Different random seeds affected only the assignment of samples to the training and test sets and the model initialization paths, without altering the overall class distribution of the dataset. By repeating experiments across multiple random seeds, the evaluation bias jointly introduced by random data partitioning and class imbalance could be partially averaged out. This strategy enables a more robust characterization of the overall performance distribution of each model, reduces the likelihood of drawing incidental conclusions from a single split, and enhances the reliability and reproducibility of the reported results.

3. Supplementary figures and tables

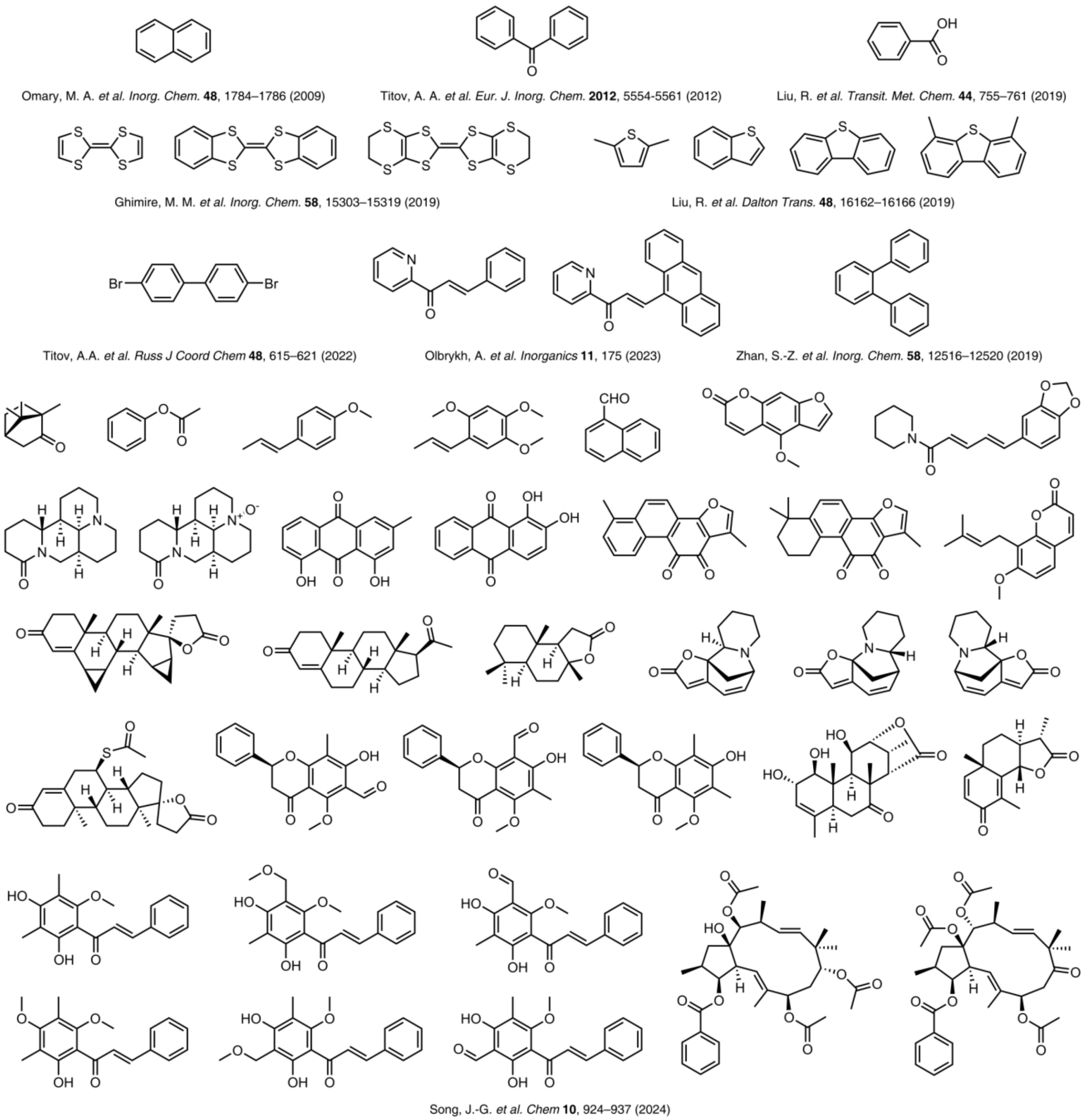

Figure S1. A dataset of organic compounds capable of forming co-crystals with Ag_3Pz_3, collected from publicly available literature sources for machine learning training (as of May 2024).[8-16]

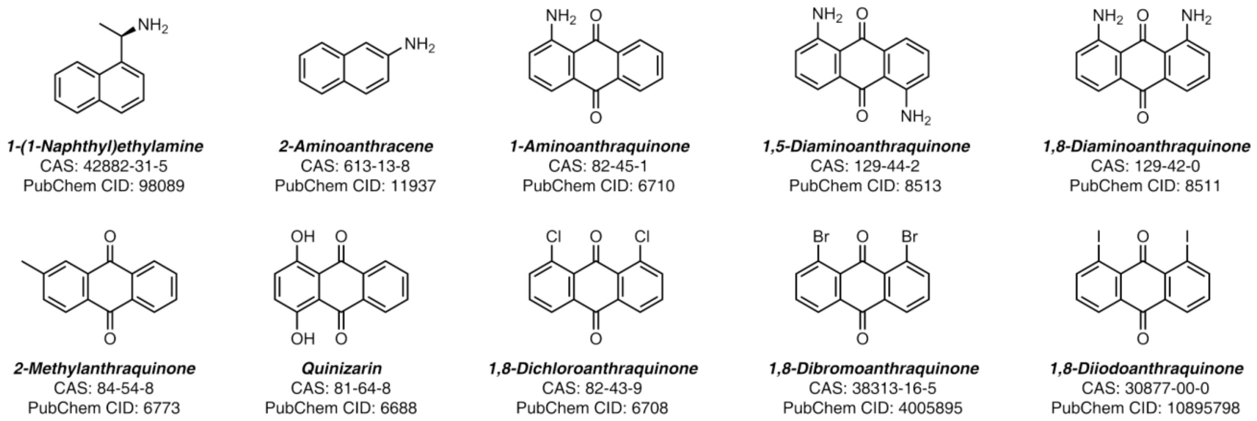

Figure S2. Structures of organic compounds that successfully formed co-crystals with Ag_3Pz_3 in high-throughput experiments. The italicized and bolded compound name, CAS number, and PubChem CID are provided below each structure.

Table S1. Summary of compounds that failed to form co-crystals with Ag_3Pz_3 in high-throughput experiments, including compound name, structural formula, molecular weight, CAS number, and PubChem CID.

No.	Compound Name	Structural Formula	Molecular Weight	CAS Number	PubChem CID
1	*β*-D-Fructofuranose	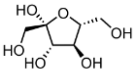	180.16	470-23-5	439709
2	*α*-L-Arabinopyranose	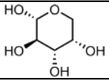	150.13	7296-55-1	439731
3	*β*-D-Glucopyranose	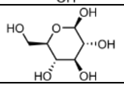	180.16	28905-12-6	64689
4	(2S,3S,4R,5R)-2,3,4,5,6-Pentahydroxyhexanal	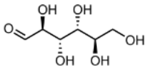	180.16	3458-28-4	161658
5	*β*-D-Galactose	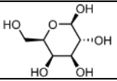	180.16	7296-64-2	439353
6	*β*-D-Lactose	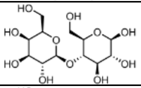	342.30	5965-66-2	6134
7	D-(+)-Cellobiose	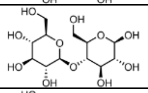	342.30	528-50-7	10712
8	*β*-Maltose	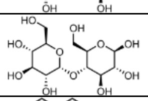	342.30	133-99-3	6255
9	[1,1'-Binaphthalene]-2,2'-Diamine	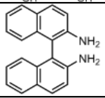	284.40	4488-22-6	20571

10	(R)-7-Bromo-2,2'-Dihydroxy-1,1'-Binaphthyl	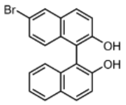	365.20	N.A.	10808802
11	9-Bromodinaphtho[2,1-d:1',2'-f][1,3]dioxepine	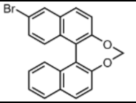	377.20	1346653-17-5	89507383
12	(R)-(+)-1-Phenylpropylamine	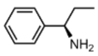	135.21	3082-64-2	5324978
13	(R)-(+)-α-Methylbenzylamine	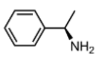	121.18	3886-69-9	643189
14	(R)-4-Bromo-α-Methylbenzylamine	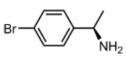	200.08	45791-36-4	853000
15	(R)-(+)-1-(p-tolyl)ethylamine	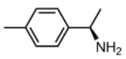	135.21	4187-38-6	7015756
16	1-Naphthylamine	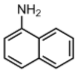	143.18	134-32-7	8640
17	Palmatine	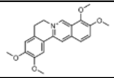	352.40	3486-67-7	19009
18	Eugenol	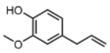	164.20	97-53-0	3314
19	Camptothecin	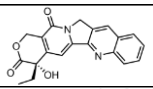	348.40	7689-03-4	24360
20	Coptisine	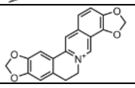	320.30	3486-66-6	72322
21	Cinnamaldehyde	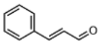	132.16	14371-10-9	637511
22	L(-)-Carvone	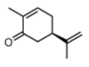	150.22	6485-40-1	439570
23	(R)-1-Phenylethanol	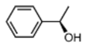	122.16	1517-69-7	637516
24	Podofilox	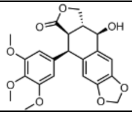	414.40	518-28-5	10607
25	Cytisinicline	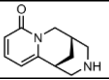	190.24	485-35-8	10235
26	Resveratrol	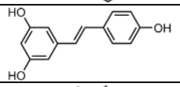	228.24	501-36-0	445154
27	Andrographolide	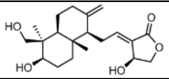	350.40	5508-58-7	5318517
28	Xanthone	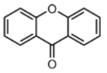	196.20	90-47-1	7020
29	Usnic Acid	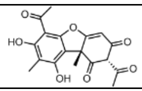	344.30	125-46-2	5646

30	Colchicine	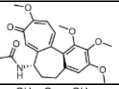	399.40	64-86-8	6167
31	Hypericin	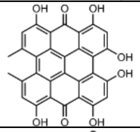	504.40	548-04-9	3663
32	Norcantharidin	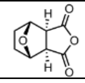	168.15	29745-04-8	9877482
33	Atractylenolide II	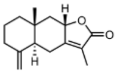	232.32	73069-14-4	14448070
34	Vanillin	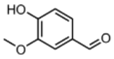	152.15	121-33-5	1183
35	Arecoline	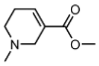	155.19	63-75-2	2230
36	Triptonide	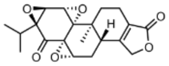	358.40	38647-11-9	65411
37	Pristimerin	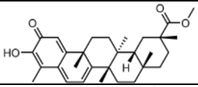	464.60	1258-84-0	159516
38	Celastrol	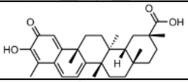	450.60	34157-83-0	122724
39	Indigo	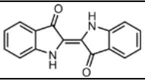	262.26	482-89-3	10215
40	Ginkgolide A	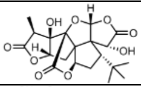	408.40	15291-75-5	9909368
41	Shikonin	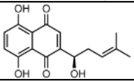	288.29	517-89-5	479503
42	Curcumin	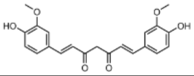	368.40	458-37-7	969516
43	Methyl Phenylacetate	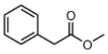	150.17	101-41-7	7559
44	2-Aminostrychnidin-10-one	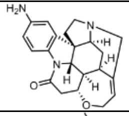	349.40	N.A.	102382
45	Cajanine	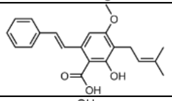	338.40	87402-84-4	9819225
46	Methyl Salicylate	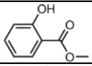	152.15	119-36-8	4133
47	Triphenylphosphine Oxide	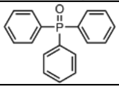	278.30	791-28-6	13097
48	Diisopropylaniline	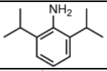	177.29	24544-04-5	32484
49	L-Menthyl Acetate	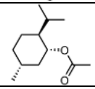	198.30	2623-23-6	220674
50	Phenyl Methanesulfonate	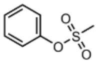	172.20	16156-59-5	316170

51	Acetic Thioanhydride	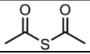	118.16	3232-39-1	76708
52	Dipropyl Sulfite	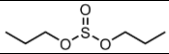	166.24	623-98-3	136434
53	Allicin	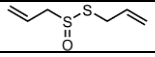	162.30	539-86-6	65036
54	Diallyl Trisulfide	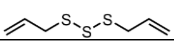	178.30	2050-87-5	16315
55	Sulforaphane	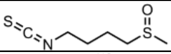	177.30	4478-93-7	5350
56	Griffonin	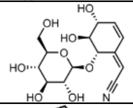	329.30	63492-69-3	10065132
57	Epigoitrin	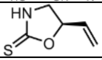	129.18	1072-93-1	3032313
58	2-Naphthyl Isocyanide	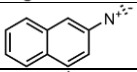	153.18	10124-78-4	16217475
59	2,6-Dimethylphenyl Isocyanide	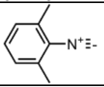	131.17	2769-71-3	76009
60	Ginkgolide B	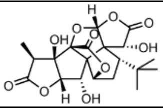	424.40	15291-77-7	11973122
61	Ginkgolide C	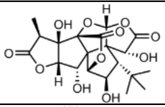	440.40	15291-76-6	9867869
62	Amoxicillin	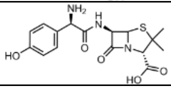	365.40	26787-78-0	33613
63	Diphenylphosphinic Chloride	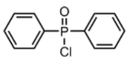	236.63	1499-21-4	73910
64	2,6-Di(thiophen-2-yl)dithieno[3,2-b:2',3'-d]thiophene	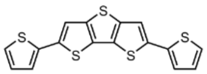	360.60	910788-24-8	37819117
65	Goitrin	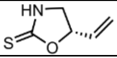	129.18	500-12-9	7568320
66	2,5-Di(thiophen-2-yl)thieno[3,2-b]thiophene	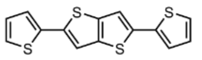	304.50	21210-90-2	11738270
67	2-Naphthalenethiol	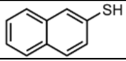	160.24	91-60-1	7058
68	Erythorbic Acid	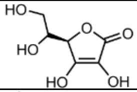	176.12	89-65-6	54675810
69	Diethyl Squarate	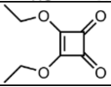	170.16	5231-87-8	123228
70	β-D-glucose Pentaacetate	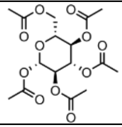	390.34	604-69-3	2724702
71	Melibiose	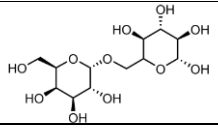	342.30	585-99-9	440658

72	(2R,3R,4R,5R,6R)-3-acetamido-6-(acetoxymethyl)tetrahydro-2H-pyran-2,4,5-triyl triacetate	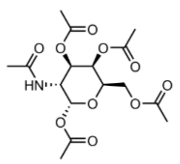	389.35	10385-50-9	11047389
73	Phlorizin	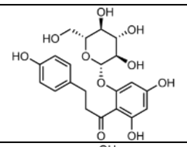	436.40	60-81-1	6072
74	Aloin B	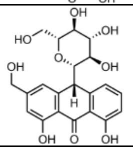	418.40	28371-16-6	14989
75	(E)-1-[3-[(2Z)-3,7-dimethylocta-2,6-dienyl]-2,4-dihydroxyphenyl]-3-(4-hydroxyphenyl)prop-2-en-1-one	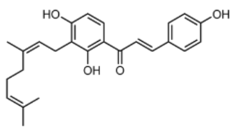	392.50	N.A.	44588243
76	Stichoneurine B	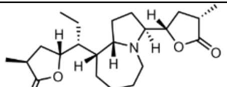	377.50	N.A.	162870991
77	Parvistemonine	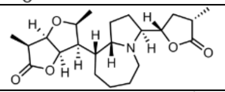	391.50	N.A.	101160221
78	Stemona-amine F	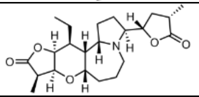	391.50	N.A.	132497115
79	Stichoneurine F	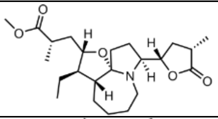	407.50	N.A.	90655427
80	Sessilifoliamide B	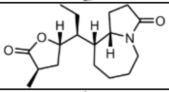	293.40	N.A.	12116760
81	Sessilifoliamide D	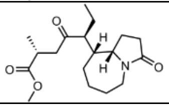	323.40	N.A.	12116762
82	Tuberostemonine	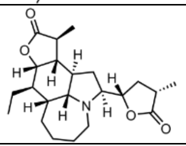	375.50	6879-01-2	100781
83	Sessilifoline A	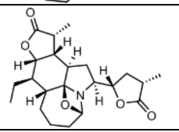	389.50	929637-35-4	91885238
84	Tuberostemoninol	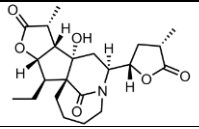	405.50	N.A.	388031
85	(+)-Stemofoline	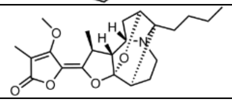	387.50	29881-57-0	70697717

86	Protostemonine	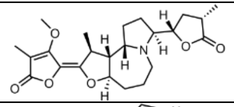	417.50	27495-40-5	25256772
87	Oxystemokerrine	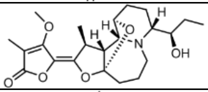	405.50	N.A.	70698070
88	Stemonamine	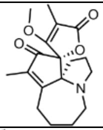	317.40	N.A.	25256774
89	Stemaphylline	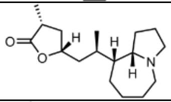	279.40	1151666-69-1	44139893
90	Croomine	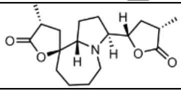	321.40	71239-66-2	3085457
91	10-Hydroxycroomine	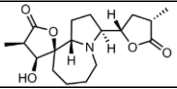	337.40	N.A.	25136348
92	Stemospironine	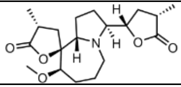	351.40	66267-46-7	11221878

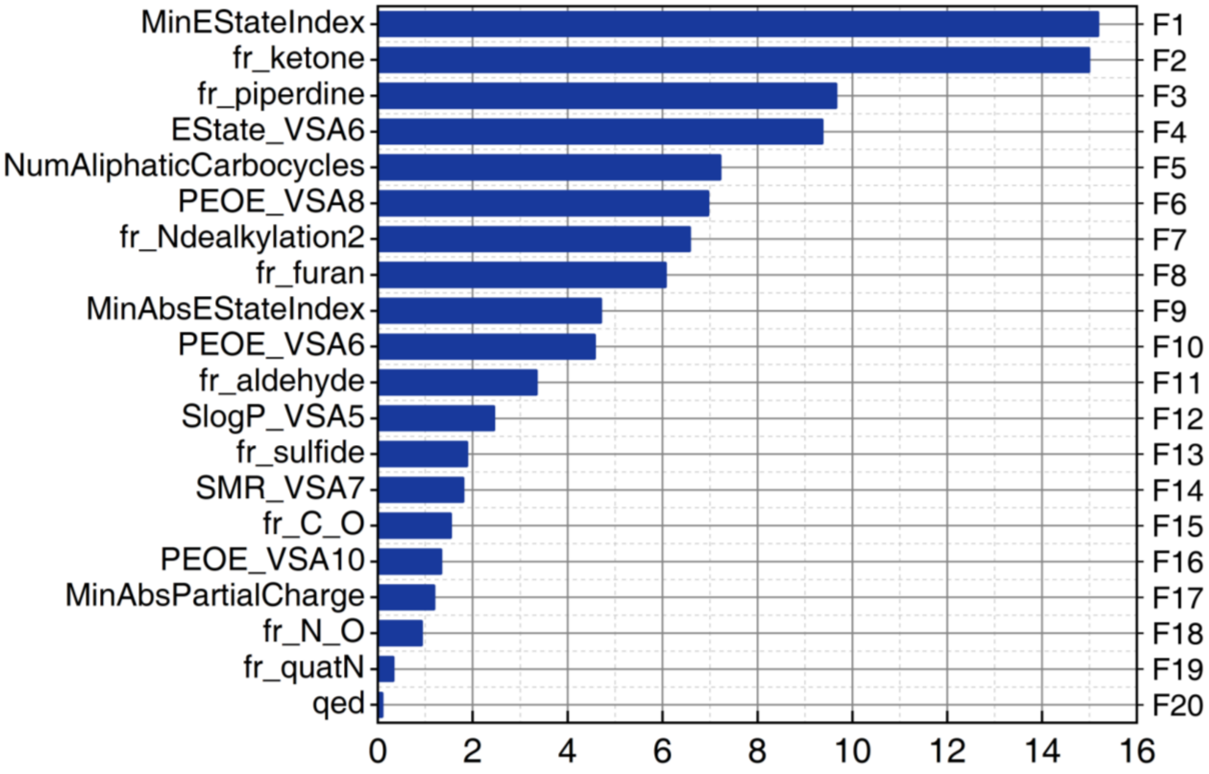

Figure S3. Relative importance ranking of the 20 nonzero importance features selected by the LASSO algorithm.

Table S2. The 20 features with non-zero importance identified by LASSO, together with their corresponding molecular descriptor definitions from RDKit toolkit.

Feature name	Description	Abbreviation
MinEStateIndex	MinEStateIndex(mol, force = 1)	F1
fr_ketone	Number of ketones	F2
fr_piperdine	Number of piperdine rings	F3
EState_VSA6	EState VSA Descriptor 6 (1.54 <= x < 1.81)	F4
NumAliphaticCarbocycles	Number of aliphatic (containing at least one non-aromatic bond) carbocycles	F5
PEOE_VSA8	MOE Charge VSA Descriptor 8 (0.00 <= x < 0.05)	F6
fr_Ndealkylation2	Number of tert-alicyclic amines (no heteroatoms, not quinine-like bridged N)	F7
fr_furan	Number of furan rings	F8
MinAbsEStateIndex	MinAbsEStateIndex(mol, force = 1)	F9
PEOE_VSA6	MOE Charge VSA Descriptor 6 (-0.10 <= x < -0.05)	F10
fr_aldehyde	Number of aldehydes	F11
SlogP_VSA5	MOE logP VSA Descriptor 5 (0.10 <= x < 0.15)	F12
fr_sulfide	Number of thioether	F13
SMR_VSA7	MOE MR VSA Descriptor 7 (3.05 <= x < 3.63)	F14
fr_C_O	Number of carbonyl O	F15
PEOE_VSA10	MOE Charge VSA Descriptor 10 (0.10 <= x < 0.15)	F16
MinAbsPartialCharge	MinAbsPartialCharge(mol, force = False)	F17
fr_N_O	Number of hydroxylamine groups	F18
fr_quatN	Number of quaternary nitrogens	F19
qed	Calculate the weighted sum of ADS mapped properties	F20

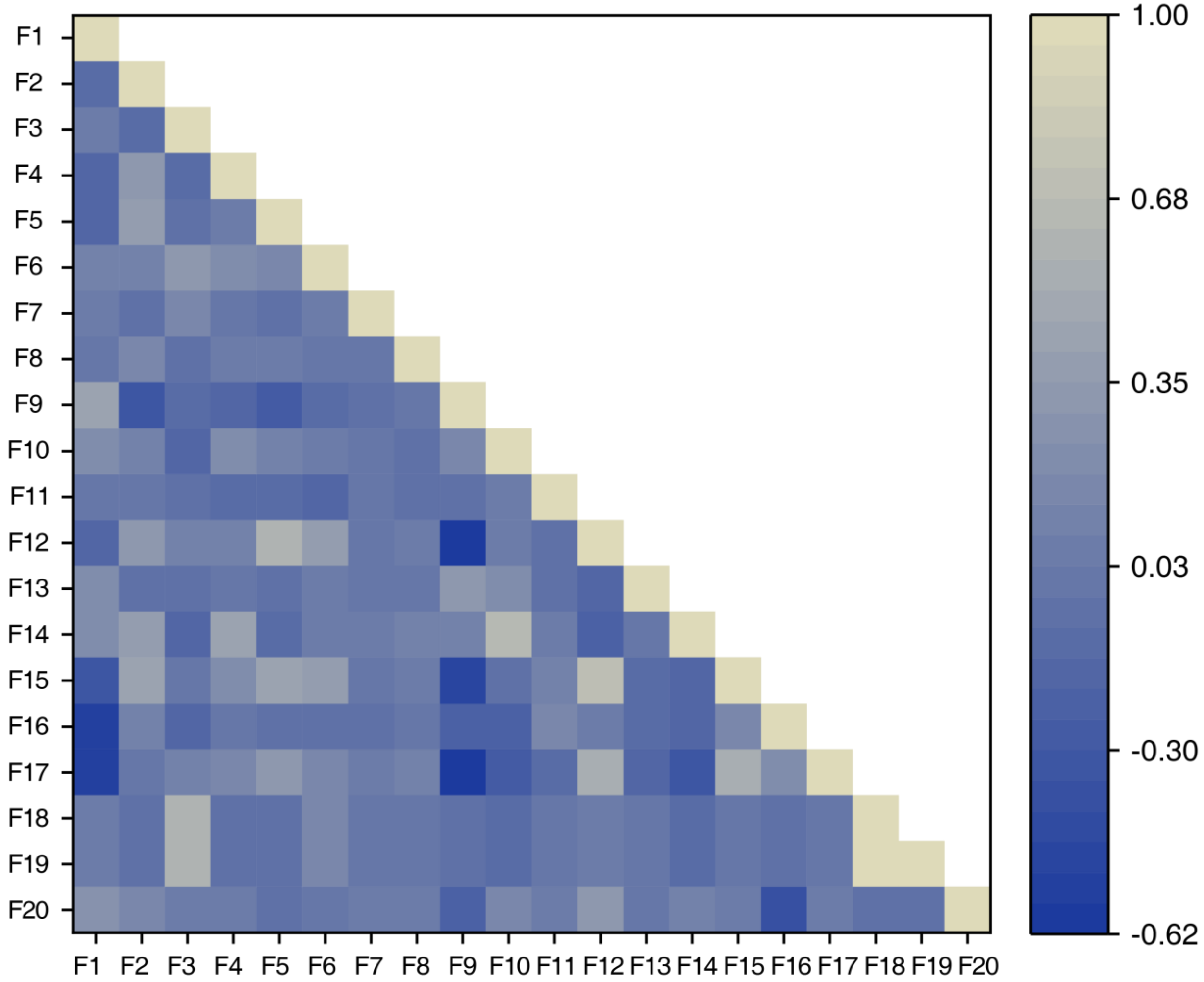

Figure S4. Pearson correlation heatmap of the 20 nonzero importance features.

Table S3. Hyperparameter search spaces and grid search settings used for training all machine learning models.

**Algorithm**	**Hyperparameter**	**Search Space**	**Optimization Method**	**CV Folds**	**Scoring Metric**
LR	Penalty, C, solver	penalty ∈ {L1, L2}; C ∈ {0.01, 0.1, 1, 10, 100}; solver = liblinear or saga (for L1); solver = newton-cg, lbfgs, or sag (for L2)	Grid Search	10-fold	ROC-AUC
SVM	C, kernel, gamma	C ∈ {0.1, 1, 10, 100}; kernel ∈ {linear, RBF}; gamma ∈ {scale, auto, 0.1, 1} (for RBF kernel)	Grid Search	10-fold	ROC-AUC
DT	riterion, max_depth, min_samples_split, min_samples_leaf, max_features	criterion ∈ {gini, entropy}; max_depth ∈ {None, 3, 5, 7, 10}; min_samples_split ∈ {2, 5, 10}; min_samples_leaf ∈ {1, 2, 4}; max_features ∈ {sqrt, None}	Grid Search	10-fold	ROC-AUC
RF	n_estimators, max_depth, min_samples_split, min_samples_leaf, max_features, bootstrap	n_estimators ∈ {50, 100, 200}; max_depth ∈ {None, 10, 20, 30}; min_samples_split ∈ {2, 5}; min_samples_leaf ∈ {1, 2}; max_features ∈ {sqrt, None}; bootstrap ∈ {True, False}	Grid Search	10-fold	ROC-AUC
GBM	n_estimators, learning_rate, max_depth, min_samples_split,	n_estimators ∈ {50, 100, 200}; learning_rate ∈ {0.01, 0.1, 0.2};	Grid Search	10-fold	ROC-AUC

	min_samples_leaf, max_features, subsample	max_depth ∈ {3, 5, 7}; min_samples_split ∈ {2, 5}; min_samples_leaf ∈ {1, 2}; max_features ∈ {sqrt, None}; subsample ∈ {0.8, 1.0}			
KNN	n_neighbors, weights, p	n_neighbors ∈ {3, 5, 7, 9, 11, 13, 15}; weights ∈ {uniform, distance}; p ∈ {1, 2}	Grid Search	10-fold	ROC-AUC
NB	var_smoothing	var_smoothing ∈ logspace(10^0, 10^{-9}) (100 values)	Grid Search	10-fold	ROC-AUC
NN	hidden_layer_sizes, activation, alpha, learning_rate_init, batch_size	hidden_layer_sizes ∈ {(50), (100), (50, 50), (100, 50)}; activation ∈ {relu, tanh}; alpha ∈ {0.0001, 0.001, 0.01}; learning_rate_init ∈ {0.001, 0.01}; batch_size ∈ {32, 64}	Grid Search	5-fold	ROC-AUC
LDA	solver, shrinkage, tol	solver ∈ {svd, lsqr, eigen}; shrinkage ∈ {None, auto, 0.1, 0.5, 0.9}; tol ∈ {1e-4, 1e-3, 1e-2}	Grid Search	5-fold	ROC-AUC
AB	n_estimators, learning_rate, base_estimator__max_depth	n_estimators ∈ {50, 100, 200}; learning_rate ∈ {0.01, 0.1, 1.0}; base_estimator__max_depth ∈ {1, 2, 3}	Grid Search	10-fold	ROC-AUC

Table S4. Best hyperparameter settings of the final models obtained for each ML algorithm.

Model	Hyperparameter	Best Value	Notes / Comments
LR	penalty	L1	L1 regularization selected; solver must support L1 (liblinear used)
	C	1	Inverse of regularization strength; balances underfitting and overfitting
	solver	liblinear	Chosen for compatibility with L1 penalty and small-sample stability
SVM	C	10	Regularization parameter; higher C reduces margin width, allowing fewer misclassifications on training data
	kernel	RBF	Radial basis function kernel for capturing non-linear relationships
	gamma	scale	Kernel coefficient automatically scaled based on feature variance
DT	criterion	gini	Splitting criterion; 'gini' measures node impurity for decision splits
	max_depth	3	Maximum depth of the tree; limits model complexity to prevent overfitting
	min_samples_split	2	Minimum number of samples required to split an internal node
	min_samples_leaf	4	Minimum number of samples required to be at a leaf node; helps smooth predictions
	max_features	sqrt	Number of features considered when looking for the best split; 'sqrt' reduces correlation among trees and improves generalization
RF	n_estimators	200	Number of trees in the forest; larger ensemble improves stability and reduces variance
	max_depth	None	No explicit depth limit, allowing trees to grow fully for capturing complex patterns
	min_samples_split	2	Minimum number of samples required to split an internal node
	min_samples_leaf	1	Minimum number of samples at a leaf node
	max_features	sqrt	Square-root of total features considered at each split to reduce feature correlation
	bootstrap	True	Bootstrap sampling enabled to enhance ensemble diversity
GBM	n_estimators	200	Number of boosting stages; larger ensemble improves predictive performance
	learning_rate	0.1	Shrinks the contribution of each tree to control the learning pace
	max_depth	5	Maximum depth of individual trees; balances model complexity and generalization
	min_samples_split	2	Minimum number of samples required to split an internal node

	min_samples_leaf	1	Minimum number of samples at a leaf node
	max_features	sqrt	Number of features considered at each split to reduce overfitting
	subsample	0.8	Fraction of samples used for fitting each base learner, introducing randomness to improve generalization
KNN	n_neighbors	15	Number of nearest neighbors considered; larger value improves robustness against noise
	weights	distance	Distance-weighted voting gives closer neighbors greater influence
	p	2	Power parameter for Minkowski distance; p = 2 corresponds to Euclidean distance
NB	var_smoothing	0.811130831	Variance smoothing parameter added to feature variances to improve numerical stability and prevent zero variance issues
NN	hidden_layer_sizes	(100, 50)	Two-layer neural network architecture with decreasing layer size
	activation	relu	Rectified linear unit activation function for efficient gradient propagation
	alpha	0.0001	L2 regularization term to reduce overfitting
	learning_rate_init	0.01	Initial learning rate controlling the optimization step size
	batch_size	32	Mini-batch size used during training to balance convergence stability and computational efficiency
LDA	lda__solver	lsqr	Least-squares solver supporting shrinkage estimation
	lda__shrinkage	0.1	Shrinkage parameter applied to covariance estimation to improve stability under small-sample conditions
	lda__tol	0.0001	Convergence tolerance for the optimization algorithm
AB	n_estimators	100	Number of boosting iterations used to build the ensemble
	learning_rate	1	Controls the contribution of each base estimator to the final model
	base_estimator__max_depth	3	Maximum depth of the base decision tree estimator, controlling base learner complexity

Table S5. Performance summary (random seeds 40-49, 80-89): Acc_cv, AUC_cv refer to accuracy and area under the ROC curve from cross-validation, and Acc_test and AUC_test refer to those from external validation set.

S value is defined as:

$S = w_1 \times Acc\_cv + w_2 \times AUC\_cv + w_3 \times Acc\_test + w_4 \times AUC\_test$, in which $w_1 = 0.2$, $w_2 = 0.3$, $w_3 = 0.2$, and $w_4 = 0.3$.

seed	Acc_cv	AUC_cv	Acc_test	AUC_test	S value
40	0.80	0.87	0.83	0.92	0.86
41	0.80	0.90	0.90	0.94	0.89
42	0.83	0.89	0.83	0.86	0.86
43	0.81	0.89	0.77	0.92	0.86
44	0.79	0.90	0.80	0.88	0.85
**45**	**0.80**	**0.90**	**0.93**	**0.96**	**0.91**
46	0.81	0.87	0.73	0.89	0.84
47	0.83	0.89	0.70	0.82	0.82
48	0.80	0.88	0.80	0.87	0.84
49	0.77	0.89	0.83	0.88	0.85
80	0.86	0.90	0.80	0.91	0.88
**81**	**0.74**	**0.86**	**1.00**	**1.00**	**0.91**
82	0.81	0.85	0.80	0.91	0.85
83	0.82	0.89	0.80	0.86	0.85
84	0.77	0.88	0.87	0.96	0.88
85	0.79	0.88	0.87	0.85	0.85
86	0.82	0.92	0.77	0.82	0.84
87	0.78	0.86	0.90	0.98	0.89
88	0.80	0.91	0.80	0.91	0.87
89	0.82	0.85	0.90	0.95	0.88

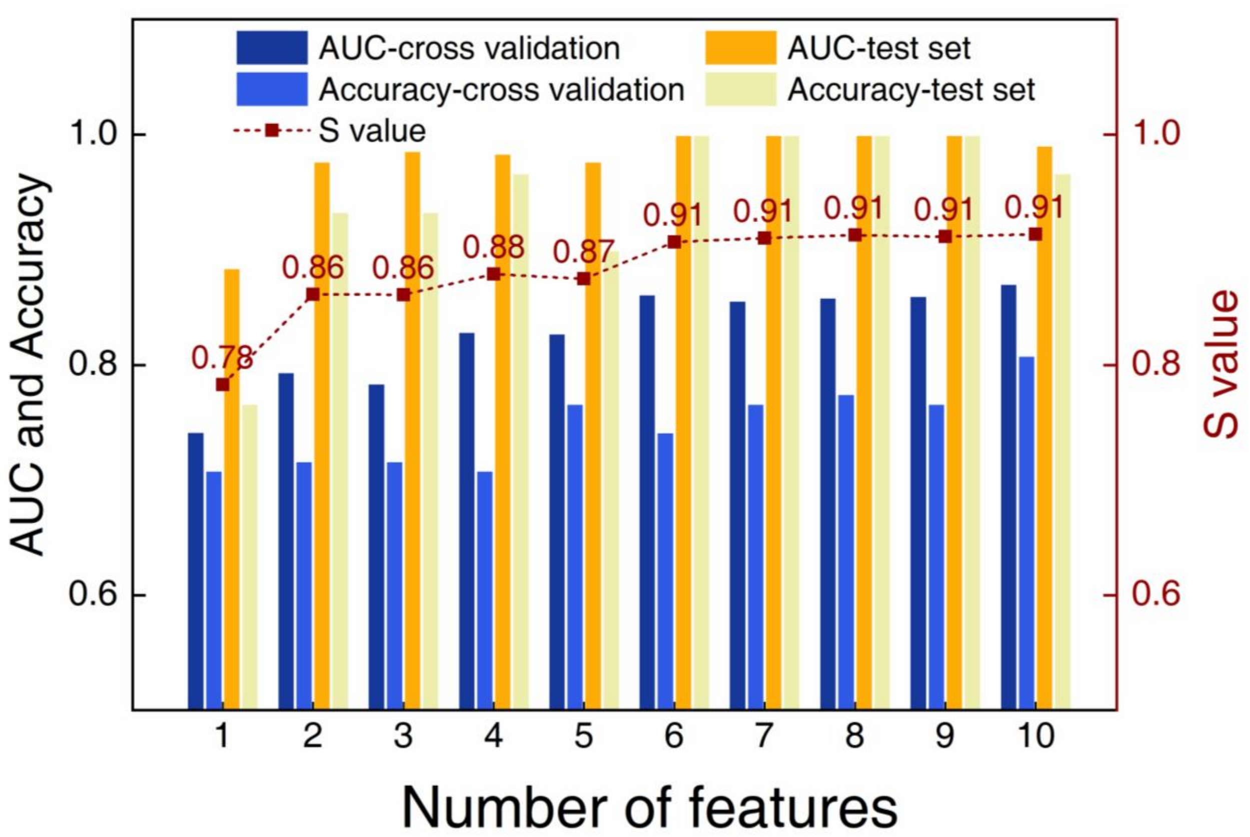

Figure S5. Performance evaluation of the KNN model under different feature numbers based on the weighted scoring function S with the optimal random seed.

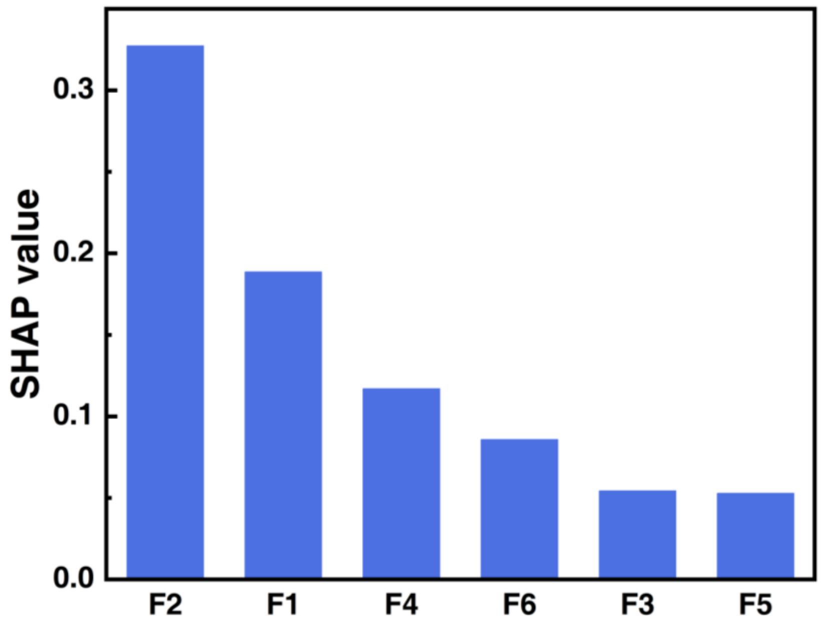

Figure S6. The sorted mean SHAP values of the six selected features in the **MCC** model.

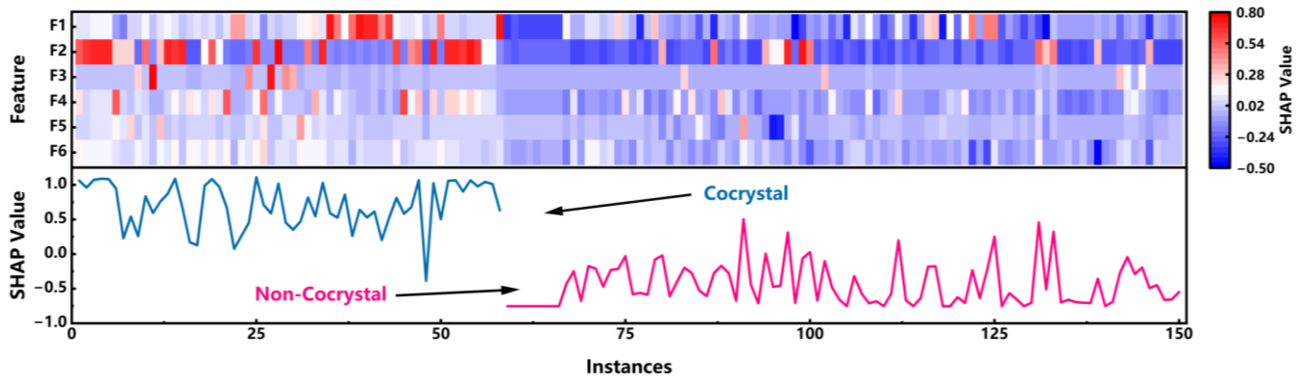

Figure S7. The SHAP values for six features across 150 samples, together with the curve of their total SHAP values, which reflects the marginal contributions of each feature to the classification of whether a compound can form a co-crystal with Ag_3Pz_3.

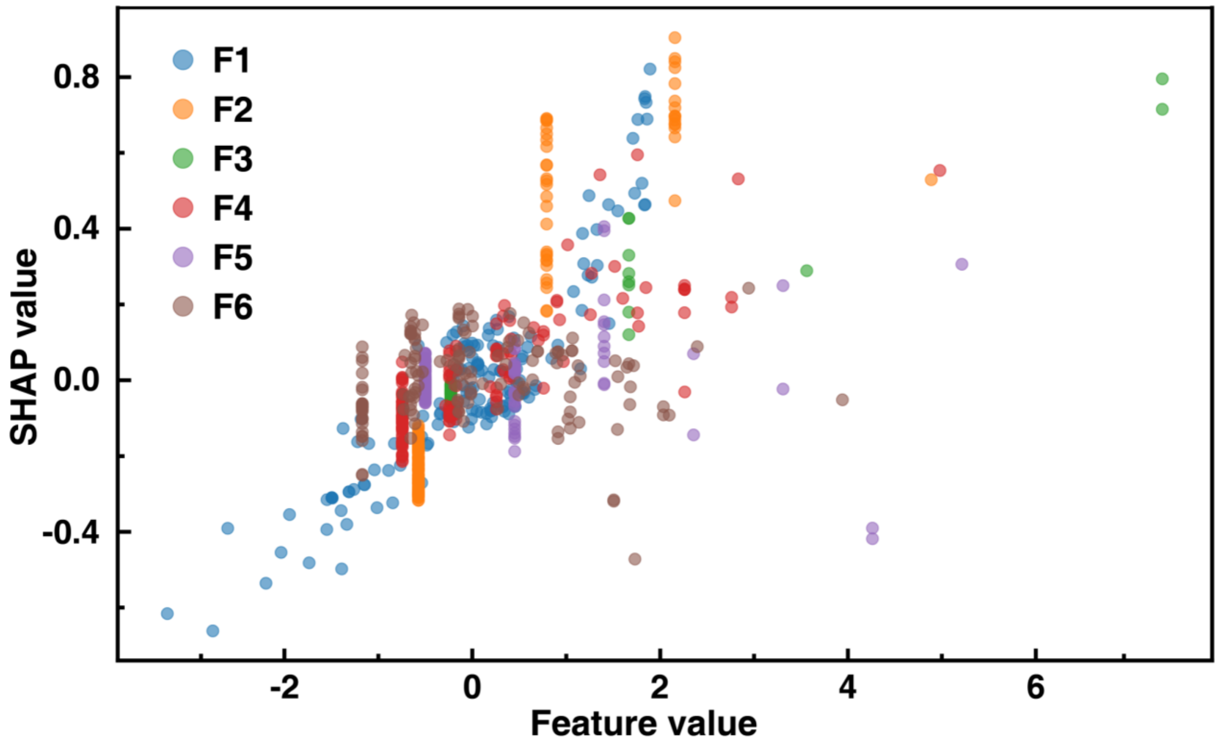

Figure S8. SHAP dependence plots for six features in the **MCC** models. Each color represents a distinct feature. Positive SHAP values indicate that a given feature drives the model prediction toward higher co-crystallization feasibility, whereas negative values indicate reduced feasibility. Feature F2 exhibits a clear monotonic relationship with model output: higher F2 values strongly promote co-crystal formation, whereas lower values favor the "non-co-crystal" category. Feature F1 shows a similar but less pronounced trend.

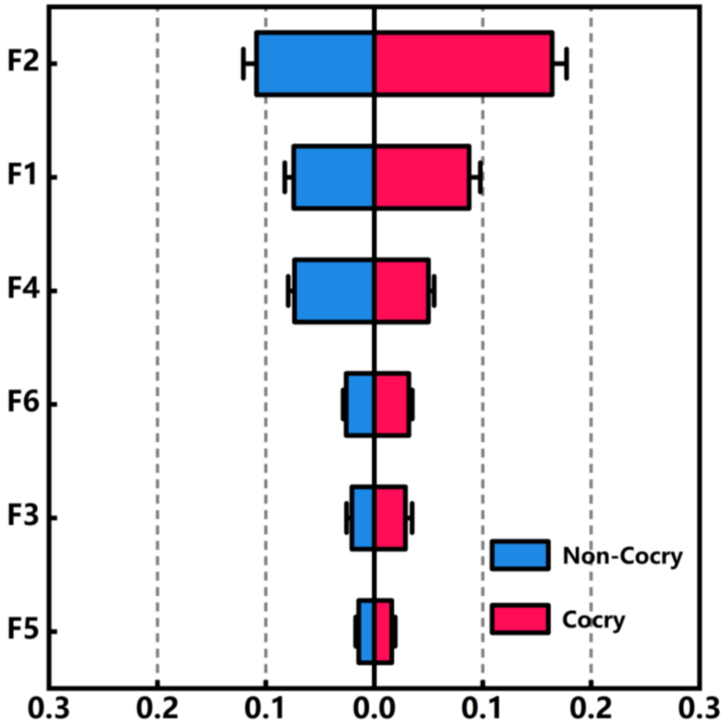

Figure S9. Bidirectional bar chart of average SHAP values for feature contributions to the "co-crystal" and " non-co-crystal " categories. Red and blue bars represent positive and negative contributions, respectively. Among all features, F2 and F1 exhibit the most significant contributions in both categories, while the average SHAP values of five features (F2, F1, F6, F3, and F5) contribute more strongly to "co-crystal". In contrast, F4 shows a dominant negative impact, favoring the " non-co-crystal " category.

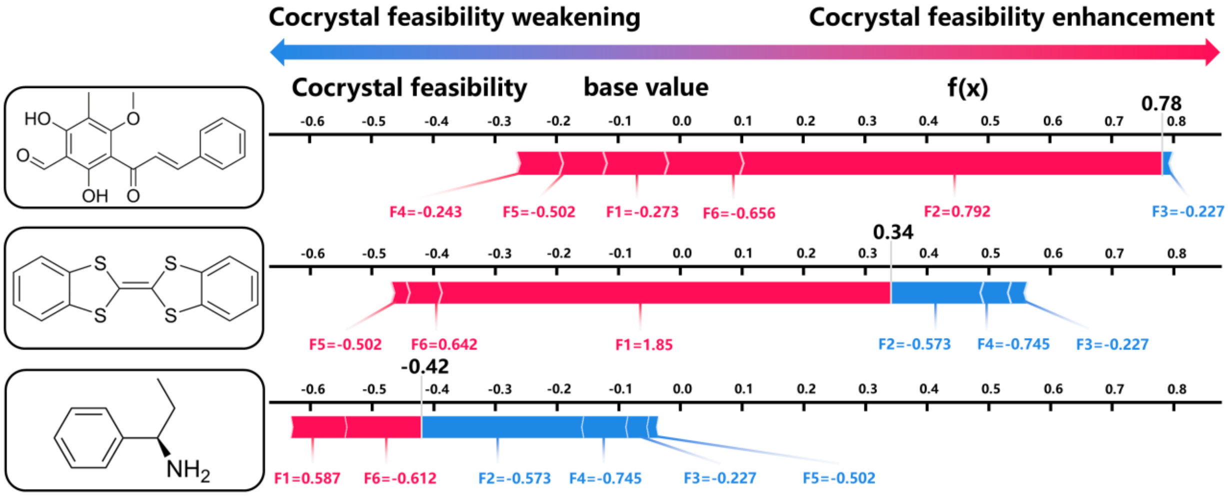

Figure S10. SHAP force plots for three representative molecules in the training set. Red and blue arrows indicate positive and negative feature contributions, respectively, with arrow length reflecting SHAP magnitude. The baseline co-crystal feasibility is -0.248, predicted values for each organic compound are 0.78, 0.34, and -0.42 (highlighted in bold black), corresponding to two co-crystal cases and one non-co-crystal case. For dibenzotetrathiafulvalene (the second line), feature F1 plays a critical positive role in enhancing co-crystal feasibility, and its positive effect on the first organic compound is also pronounced. In contrast, (R)-1-phenylpropylamine (the third line) is predicted as "non-co-crystal" due to a strong negative contribution from F2, associated with the absence of a ketone group. Additional negative impacts from F4, F3, and F5 further reinforce the non-co-crystal prediction.

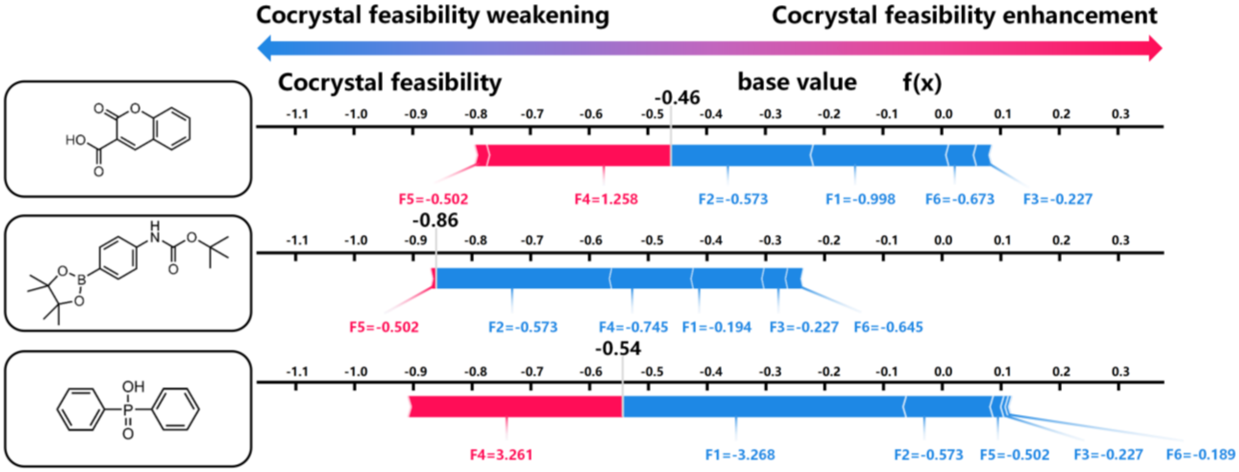

Figure S11. SHAP force plots for three representative organic molecules that were determined as no-co-crystal by **MCC**. Red and blue arrows indicate positive and negative SHAP feature contributions, respectively, with arrow length proportional to the SHAP magnitude. The baseline co-crystal feasibility (base value), derived from the training set, is -0.248. The predicted values for the three molecules, highlighted in bold black, are -0.46, -0.86, and -0.54, all indicating non–co-crystal cases consistent with experimental validation. For coumarin-3-carboxylic acid (the first line), features F1-F3 contribute negatively, while only a few (e.g., F4) show positive effects; the cumulative negative influence shifts the prediction toward the unfavorable region. The tert-butyl N-[4-(4,4,5,5-tetramethy1-1,3,2-dioxaborolan-2-yl)phenyl]carbamate (the second line) exhibits stronger inhibitory effects: F1, F2, F3, and F6 collectively drive a large negative deviation, making this the most confidently rejected case. For diphenylphosphinic acid (the third line), F4 provides a positive contribution, but F1, F2, F3, and F5 impose competing negative effects; the net result remains below baseline. Overall, these feature-level SHAP interpretations clarify why the model rejects co-crystal formation for these molecules and verify the predictive rationality of the developed approach.

Sample Name	Molecule Image	SMILES	Decision Function Value
test0001	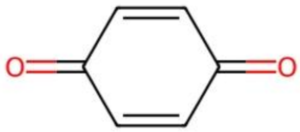	O=C1C=CC(=O)C=C1	0.432799942
test0002	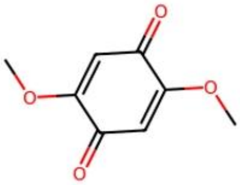	COC1=CC(=O)C(OC)=CC1=O	0.600544761
test0003	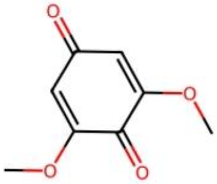	COC1=CC(=O)C=C(OC)C1=O	0.598733587
test0004	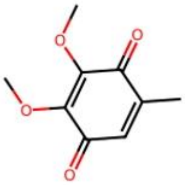	COC1=C(OC)C(=O)C(C)=CC1=O	0.591182996
test0005	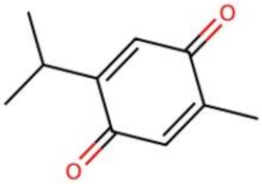	CC1=CC(=O)C(C(C)C)=CC1=O	0.647746768

Figure S12. Example output of **MCC** prediction results. Shown are the screenshot of the output file including molecular structure images, SMILES strings, and corresponding prediction scores. This complete output file is provided in the code package within the supplementy materials.

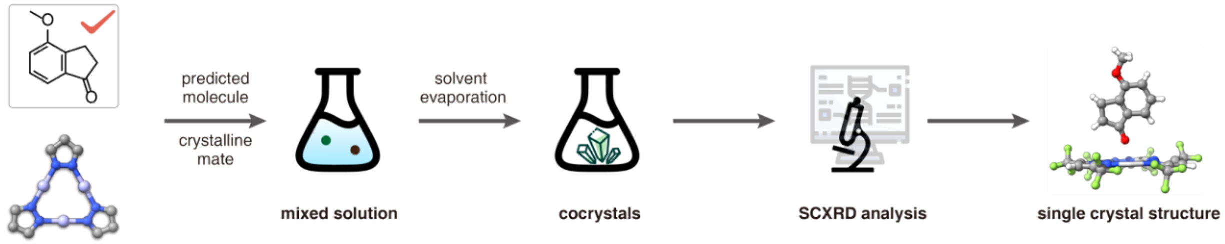

Figure S13. Workflow for the co-crystallization of Ag_3Pz_3 with organic compounds predicted and selected by **MCC**.

Table S6. Compilation of the experimental conditions under which successful co-crystallization was achieved in the formal experiments.

Compound Amount	Co-crystallization Method	Solvent condition
0.0107 mmol $\mathbf{Ag_3Pz_3}$ + 0.0107 mmol **Organic compound**	Solvent evaporation	3 mL DCM
		3 mL DCM: MeOH (1:1, v/v)
		3 mL MeOH: MeCN (1:1, v/v)
		3 mL n-Hex
		3 mL c-Hex
		3 mL DCM: n-Hex (1:1, v/v)
		3 mL DCM: c-Hex (1:1, v/v)
		3 mL n-Hex: c-Hex (1:1, v/v)
		3 mL Me_2CO: c-Hex (1:1, v/v)

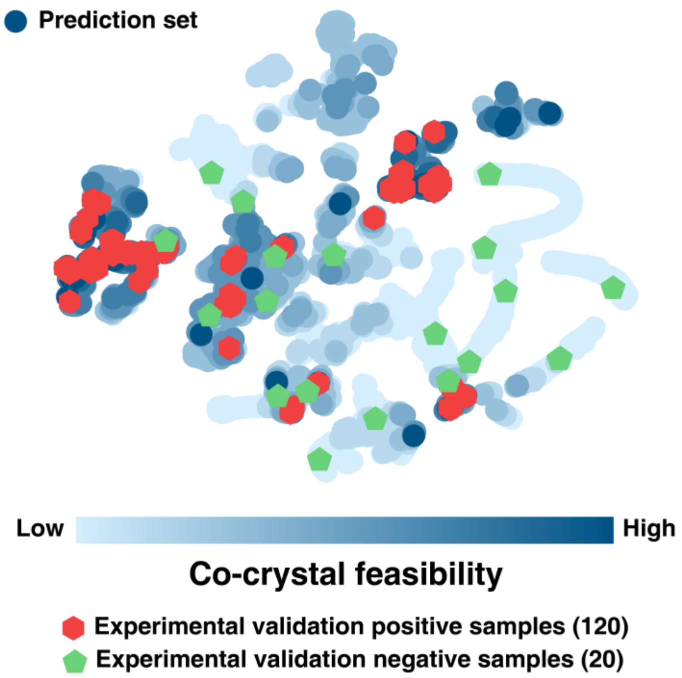

Figure S14. Predicted co-crystal feasibility of prediction set visualized by t-SNE method. The positive and negative samples chosen for experimental validation are indicated by red and green symbols, respectively.

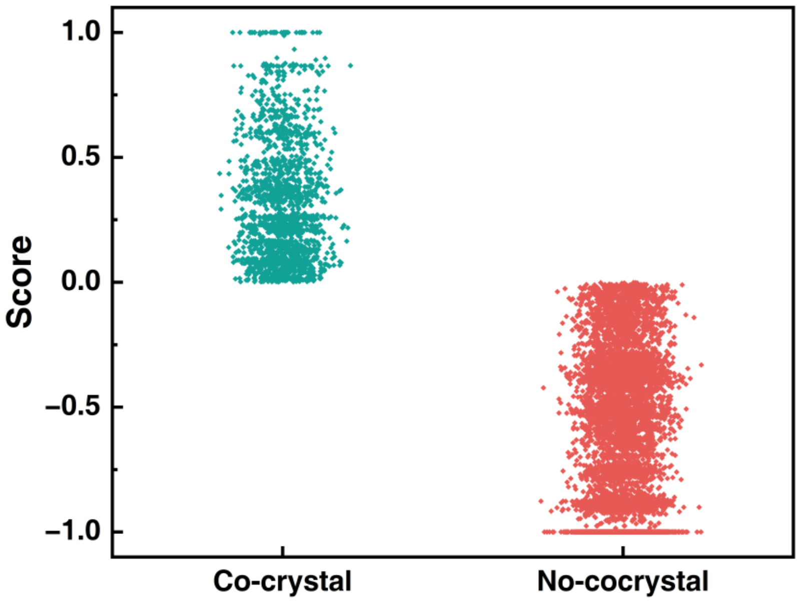

Figure S15 Distribution of predicted scores for 5,406 compounds collected from the MedChemExpress database, grouped by their co-crystal and non-co-crystal outcomes.

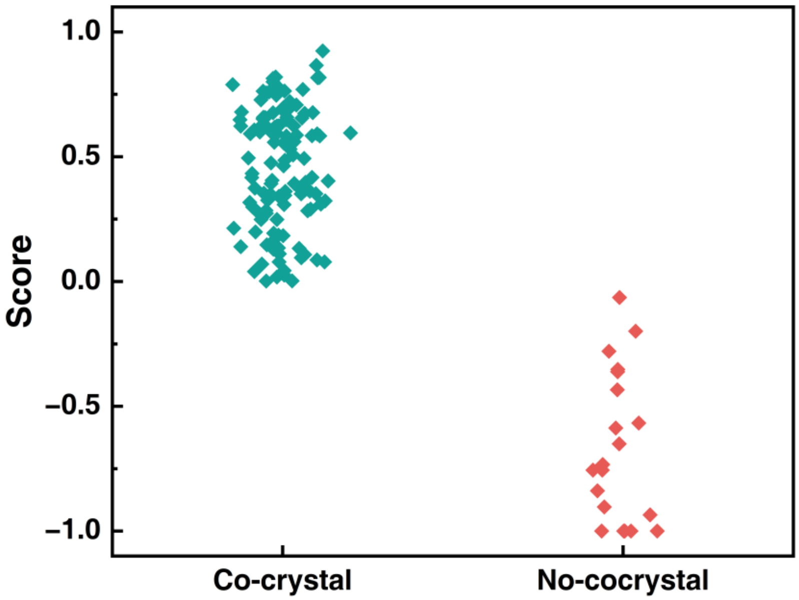

Figure S16. Distribution of predicted scores for 140 compounds selected for experimental validation, grouped by their co-crystal and non-co-crystal outcomes.

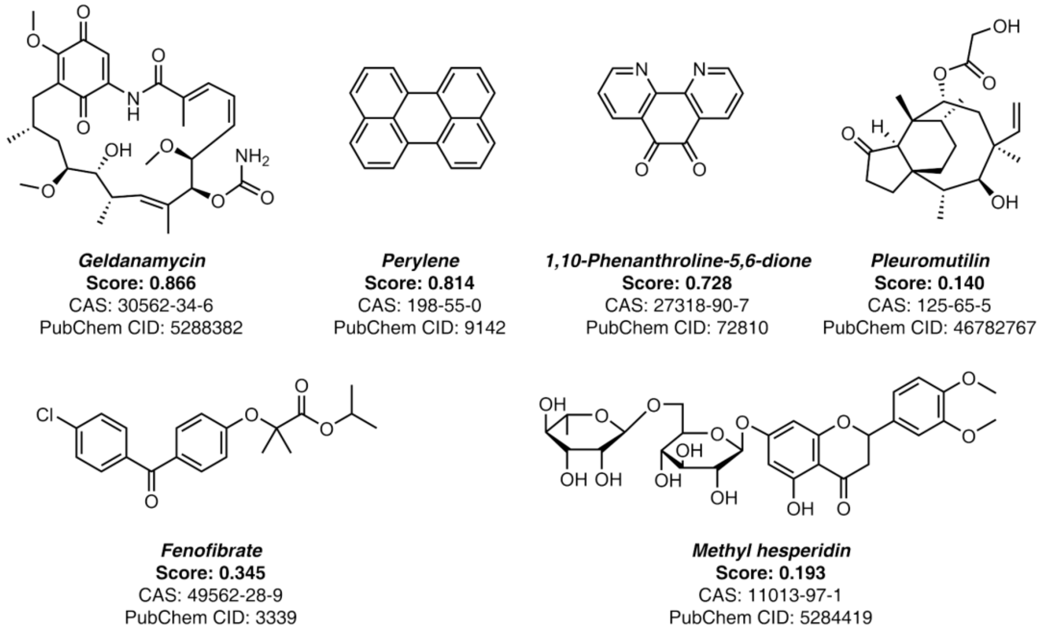

Figure S17. Organic compounds that received positive prediction scores from the **MCC** model but failed to form co-crystals with Ag_3Pz_3 in experimental validation. Each structure is annotated with the compound name, model prediction score, CAS number, and PubChem CID.

Table S7. Co-crystallization conditions and outcomes for organic compounds predicted as positive by the **MCC** model but experimentally failed to form co-crystals with Ag_3Pz_3. The table provides the compound name, experimental conditions, and observed results. "Ag_3Pz_3" in the Results column indicates that only Ag_3Pz_3 crystals were obtained, while "N.A." denotes that no crystalline material was formed.

Number	Compound Name	Co-crystallization condition	Result
1	Geldanamycin	3 mL DCM: MeOH (1:1, v/v)	Ag_3Pz_3
		3 mL DCM: n-Hex (1:1, v/v)	Ag_3Pz_3
		3 mL DCM: c-Hex (1:1, v/v)	Ag_3Pz_3
2	Perylene	3 mL DCM	N.A.
		3 mL DCM: MeOH (1:1, v/v)	N.A.
		3 mL MeOH: MeCN (1:1, v/v)	N.A.
3	1,10-Phenanthroline-5,6-dione	3 mL DCM: MeOH (1:1, v/v)	N.A.
		3 mL DCM: n-Hex (1:1, v/v)	N.A.
		3 mL DCM: c-Hex (1:1, v/v)	N.A.
4	Pleuromutilin	3 mL DCM: MeOH (1:1, v/v)	Ag_3Pz_3
		3 mL DCM: n-Hex (1:1, v/v)	N.A.
		3 mL DCM: c-Hex (1:1, v/v)	N.A.
5	Fenofibrate	3 mL DCM: MeOH (1:1, v/v)	Ag_3Pz_3
		3 mL DCM: n-Hex (1:1, v/v)	Ag_3Pz_3
		3 mL DCM: c-Hex (1:1, v/v)	Ag_3Pz_3
6	Methyl hesperidin	3 mL DCM: MeOH (1:1, v/v)	Ag_3Pz_3
		3 mL MeOH: MeCN (1:1, v/v)	N.A.

Possible reasons for failure include a) differences in solubility of the Ag_3Pz_3 in the chosen solvent systems (Nos. 1-6), b) coordination activity that may trigger structural rearrangement of the Ag_3Pz_3 (Nos. 2-3), c) redox behavior that can compromise solution stability (No. 6), and d) insufficient purity of commercially available samples or imprecise control of partner loading (Nos. 1-6).

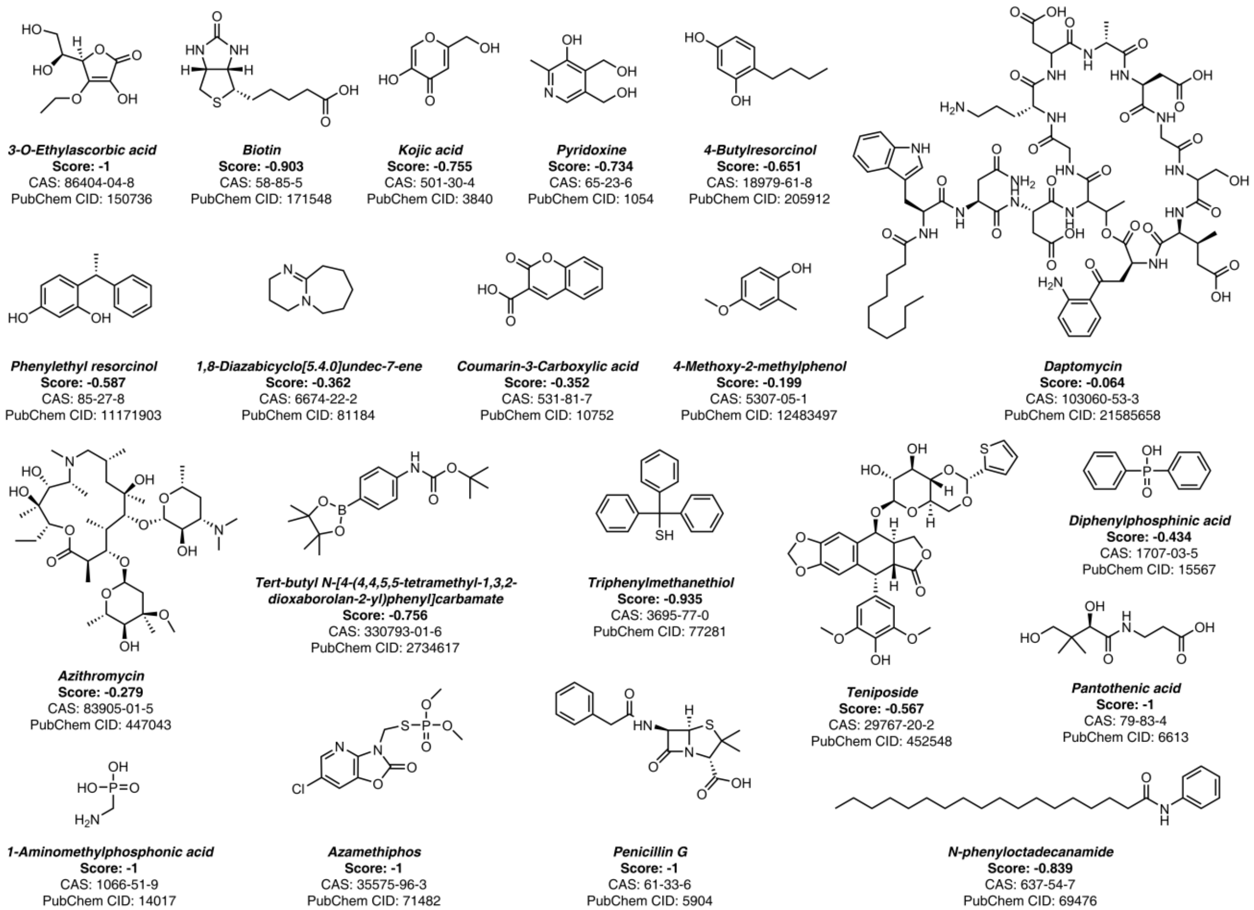

Figure S18. Organic compounds predicted as negative by the **MCC** model and experimentally failed to form co-crystals with Ag_3Pz_3. Each molecular structure is annotated with the compound name, model prediction score, CAS number, and PubChem CID.

Table S8. Co-crystallization conditions and outcomes for organic compounds that were predicted as <u>negative</u> by the MCC model and experimentally <u>failed</u> to form co-crystals with Ag_3Pz_3. The table summarizes each compound, the applied experimental conditions, and the observed results. "Ag_3Pz_3" in the Results column indicates that only Ag_3Pz_3 crystals were obtained, whereas "Compound" denotes that crystals of the organic compound itself were formed. "N.A." represents that no crystalline material was obtained.

Number	Compound Name	Co-crystallization condition	Result
1	3-O-Ethylascorbic acid	3 mL DCM: MeOH (1:1, v/v)	Ag_3Pz_3
		3 mL DCM: MeOH (4:1, v/v)	Ag_3Pz_3
		3 mL MeOH: MeCN (1:1, v/v)	N.A.
2	Biotin	3 mL DCM: MeOH (1:1, v/v)	Ag_3Pz_3
		3 mL MeOH: MeCN (1:1, v/v)	N.A.
		3 mL MeOH: Me_2CO (1:1, v/v)	N.A.
3	Kojic acid	3 mL MeOH: PhMe (2:1, v/v)	N.A.
		3 mL MeOH: MeCN (1:1, v/v)	N.A.
		3 mL n-Hex: Me_2CO (1:1, v/v)	N.A.
4	Pyridoxine	3 mL DCM: MeOH (1:1, v/v)	Ag_3Pz_3
		3 mL MeOH: MeCN (1:1, v/v)	N.A.
		3 mL MeOH: Me_2CO (1:1, v/v)	N.A.
5	4-Butylresorcinol	3 mL DCM: MeOH (1:1, v/v)	Ag_3Pz_3
		3 mL DCM: n-Hex (1:1, v/v)	N.A.
		3 mL DCM: c-Hex (1:1, v/v)	N.A.
6	Phenylethyl resorcinol	3 mL MeOH: PhMe (2:1, v/v)	N.A.
		3 mL MeCN: PhMe (2:1, v/v)	N.A.
		3 mL Me_2CO: c-Hex (1:1, v/v)	Ag_3Pz_3
7	1,8-Diazabicyclo[5.4.0]undec-7-ene	3 mL DCM: MeOH (1:1, v/v)	Ag_3Pz_3
		3 mL DCM: n-Hex (1:1, v/v)	N.A.
		3 mL DCM: c-Hex (1:1, v/v)	N.A.
8	Coumarin-3-Carboxylic Acid	3 mL DCM: MeOH (1:1, v/v)	N.A.
		3 mL DCM: n-Hex (1:1, v/v)	N.A.
		3 mL DCM: c-Hex (1:1, v/v)	N.A.
9	4-Methoxy-2-methylphenol	3 mL DCM: MeOH (1:1, v/v)	N.A.
		3 mL DCM: n-Hex (1:1, v/v)	Ag_3Pz_3
		3 mL DCM: c-Hex (1:1, v/v)	Ag_3Pz_3
10	Daptomycin	3 mL MeOH: DMF (1:1, v/v)	N.A.
		3 mL MeOH: DMSO (1:1, v/v)	N.A.
		3 mL MeOH: PhMe (1:1, v/v)	N.A.
11	Azithromycin	3 mL DCM: MeOH (1:1, v/v)	Ag_3Pz_3
		3 mL DCM: n-Hex (1:1, v/v)	Ag_3Pz_3

		3 mL DCM: c-Hex (1:1, v/v)	Ag_3Pz_3
12	Tert-butyl N-[4-(4,4,5,5-tetramethyl-1,3,2-dioxaborolan-2-yl)phenyl]carbamate	3 mL n-Hex	Compound
		3 mL c-Hex	Ag_3Pz_3
		3 mL DCM: n-Hex (1:1, v/v)	N.A.
13	Triphenylmethanethiol	3 mL DCM: MeOH (1:1, v/v)	N.A.
		3 mL DCM: n-Hex (1:1, v/v)	N.A.
		3 mL DCM: c-Hex (1:1, v/v)	N.A.
14	Teniposide	3 mL DCM: MeOH (1:1, v/v)	Ag_3Pz_3
		3 mL DCM: n-Hex (1:1, v/v)	Ag_3Pz_3
		3 mL DCM: c-Hex (1:1, v/v)	N.A.
15	Diphenylphosphinic acid	3 mL DCM: MeOH (1:1, v/v)	N.A.
		3 mL DCM: n-Hex (1:1, v/v)	N.A.
		3 mL DCM: c-Hex (1:1, v/v)	N.A.
16	Pantothenic acid	3 mL DCM: MeOH (1:1, v/v)	Ag_3Pz_3
		3 mL MeOH: MeCN (1:1, v/v)	N.A.
		3 mL DCM: c-Hex (1:1, v/v)	N.A.
17	1-Aminomethylphosphonic acid	3 mL DCM: MeOH (1:1, v/v)	Ag_3Pz_3
		3 mL DCM: n-Hex (1:1, v/v)	N.A.
		3 mL DCM: c-Hex (1:1, v/v)	Ag_3Pz_3
18	Azamethiphos	3 mL DCM: MeOH (1:1, v/v)	N.A.
		3 mL DCM: n-Hex (1:1, v/v)	Ag_3Pz_3
		3 mL DCM: c-Hex (1:1, v/v)	N.A.
19	Penicillin G	3 mL DCM: MeOH (1:1, v/v)	N.A.
		3 mL DCM: n-Hex (1:1, v/v)	Ag_3Pz_3
		3 mL DCM: c-Hex (1:1, v/v)	Ag_3Pz_3
20	N-phenyloctadecanamide	3 mL DCM: MeOH (1:1, v/v)	Ag_3Pz_3
		3 mL DCM: n-Hex (1:1, v/v)	N.A.
		3 mL DCM: c-Hex (1:1, v/v)	N.A.

4. Co-crystallization experiments

A comprehensive literature survey revealed that most studies on the co-crystallization of Ag_3Pz_3 with organic compounds employ the solvent evaporation method, typically using solvents include dichloromethane, methanol, acetonitrile, and n-hexane. To ensure consistency and comparability of experimental conditions while improving experimental efficiency, we adopted the solvent evaporation method in this work and designed eight solvent systems: dichloromethane, dichloromethane/methanol (1:1, v/v), methanol/acetonitrile (1:1, v/v), n-hexane, cyclohexane, dichloromethane/n-hexane (1:1, v/v), dichloromethane/cyclohexane (1:1, v/v), and n-hexane/cyclohexane (1:1, v/v). These systems were selected to accommodate the varying solubilities of different organic compounds while aligning closely with reported conditions. All samples were prepared under identical weighing, handling, crystal growth procedures. Some target organic compounds formed co-crystals with Ag_3Pz_3 in more than one solvent systems. To rapidly evaluate the predictive accuracy of the previously established ML model, all crystalline samples were subjected to crystal parameter testing, and their unit cell data were recorded. For cases where significant differences in unit cell parameters were observed, only the highest quality crystal was selected for single-crystal X-ray diffraction analysis, with detailed documentation of its corresponding growth conditions.

Preparation of Ag_3Pz_3·1. 1.16 mg (0.0107 mmol) of 1,4-benzoquinone (**1**) was dissolved in 3 mL of a binary solvent system of DCM and n-Hex (1:1, v/v), followed by the addition of equimolar amounts of Ag_3Pz_3 (10.00 mg, 0.0107 mmol). The resulting mixed solution was filtered and then transferred to a 20 mL screw-capped sample vial. The cap of the sample vial was loosely closed to allow the solvent to slowly evaporate at room temperature. The entire co-crystal incubation process was protected from light using aluminum foil. After the designated evaporation period, typically 1-3 days, high-quality yellow plate-shaped crystals suitable for single-crystal X-ray diffraction analysis formed at the bottom of the vial.

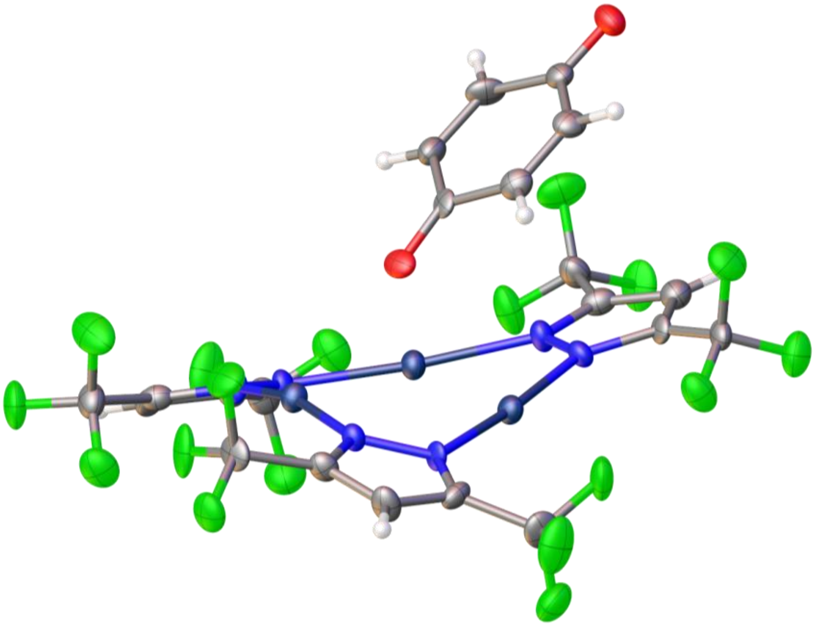

Figure S19. Asymmetric unit of **Ag_3Pz_3·1** (thermal displacement parameters at the 50% probability level).

Table S9. Crystal data and structure refinement for **Ag_3Pz_3·1**

Empirical formula	$C_{21}H_7Ag_3F_{18}N_6O_2$
Formula weight	1040.94
Temperature/K	100.01(19)
Crystal system	orthorhombic
Space group	$P2_12_12_1$
a/Å	8.50290(10)
b/Å	16.2171(2)
c/Å	21.0602(2)
α/°	90
β/°	90
γ/°	90
Volume/Å^3	2904.04(6)
Z	4
ρ_{calc}g/cm^3	2.381
μ/mm^{-1}	17.507
F(000)	1976.0
Crystal size/mm^3	0.16 × 0.14 × 0.1
Radiation	Cu Kα (λ = 1.54184)
2θ range for data collection/°	6.88 to 156.88
Index ranges	$-10 \le h \le 10$, $-19 \le k \le 10$, $-25 \le l \le 26$
Reflections collected	13226
Independent reflections	5791 [R_{int} = 0.0430, R_{sigma} = 0.0467]
Data/restraints/parameters	5791/12/452
Goodness-of-fit on F^2	1.106
Final R indexes [I>=2σ (I)]	R_1 = 0.0538, wR_2 = 0.1503
Final R indexes [all data]	R_1 = 0.0576, wR_2 = 0.1527
Largest diff. peak/hole / e Å^{-3}	1.80/-0.86
Flack parameter	0.34(2)
CCDC-number	2501752

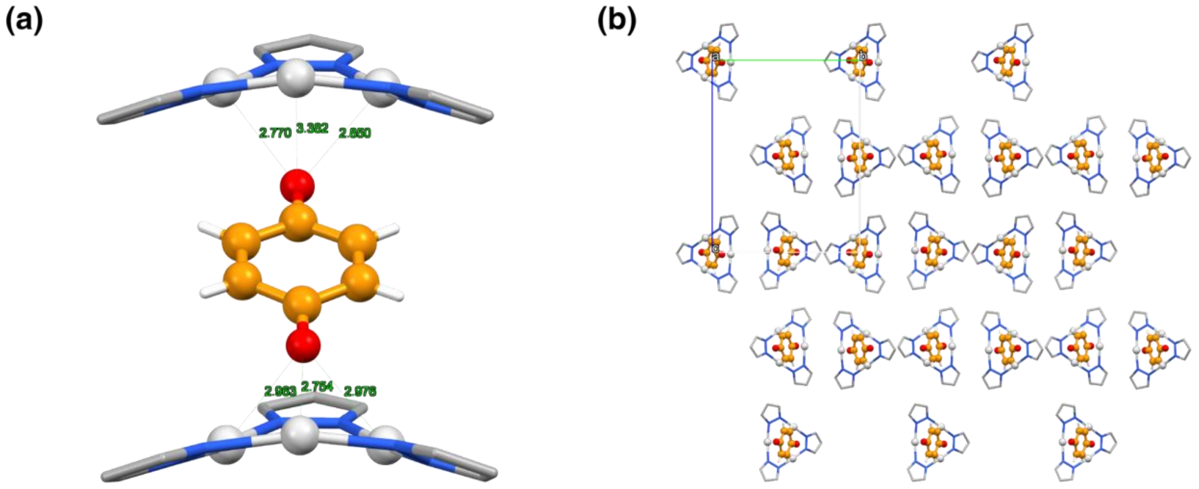

Figure S20. (a) A schematic diagram of the co-crystal structure in the $\mathbf{Ag_3Pz_3{\cdot}1}$ single crystal, formed by the guest organic molecule and the surrounding Ag_3Pz_3 units that exhibit significant interactions with it. (b) A 1 × 3 × 2 packing mode in the single crystal structure of $\mathbf{Ag_3Pz_3{\cdot}1}$ along the *a* axis. Trifluoromethyl groups and H atoms in Ag_3Pz_3 are omitted for clarity. Ag···O interactions are indicated with green dotted lines with distances in Å. C, N, and Ag atoms in Ag_3Pz_3 are depicted in dark gray, light blue, and light gray, respectively; C, O, and H atoms in **1** are depicted in orange, red, and white, respectively.

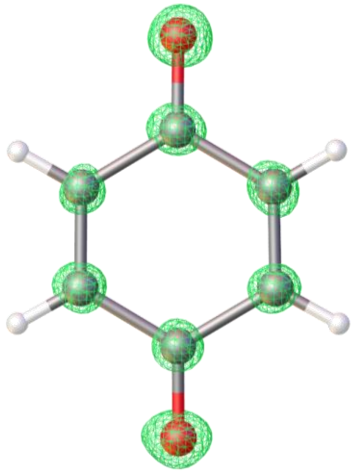

Figure S21. F_{obs} (contour: 1.25) electron density map superimposed on the structure of **1** in the single crystal structure of $\mathbf{Ag_3Pz_3{\cdot}1}$.

Preparation of Ag_3Pz_3·2. 1.80 mg (0.0107 mmol) of 2,5-dimethoxy-1,4-benzoquinone (**2**) was dissolved in 3 mL of a binary solvent system of DCM and n-Hex (1:1, v/v), followed by the addition of equimolar amounts of Ag_3Pz_3 (10.00 mg, 0.0107 mmol). The resulting mixed solution was filtered and then transferred to a 20 mL screw-capped sample vial. The cap of the sample vial was loosely closed to allow the solvent to slowly evaporate at room temperature. The entire co-crystal incubation process was protected from light using aluminum foil. After the designated evaporation period, typically 1-3 days, high-quality yellow plate-shaped crystals suitable for single-crystal X-ray diffraction analysis formed at the bottom of the vial.

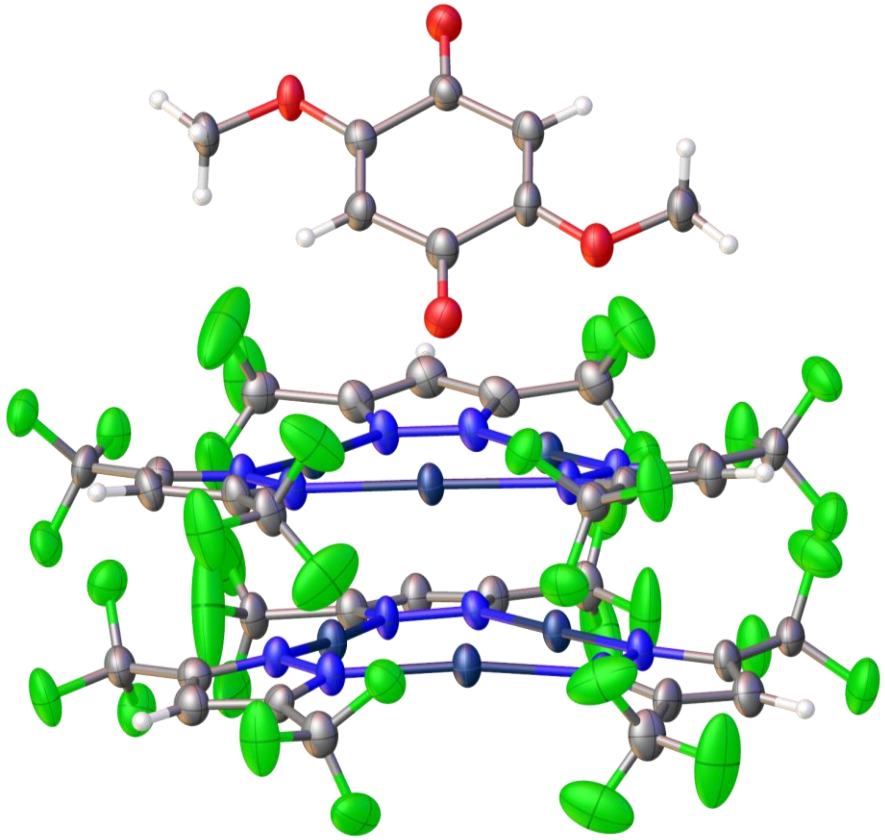

Figure S22. Asymmetric unit of **Ag_3Pz_3·2** (thermal displacement parameters at the 50% probability level).

Table S10. Crystal data and structure refinement for **Ag_3Pz_3·2**

Empirical formula	$C_{38}H_{14}Ag_6F_{36}N_{12}O_4$
Formula weight	2033.83
Temperature/K	100.01(16)
Crystal system	monoclinic
Space group	$P2_1/c$
a/Å	11.6037(2)
b/Å	13.31850(10)
c/Å	35.1044(3)
α/°	90
β/°	91.6730(10)
γ/°	90
Volume/Å^3	5422.86(11)
Z	4
ρ_{calc}g/cm^3	2.491
μ/mm^{-1}	18.724
F(000)	3856.0
Crystal size/mm^3	0.25 × 0.23 × 0.13
Radiation	Cu Kα (λ = 1.54184)
2θ range for data collection/°	5.036 to 157.072
Index ranges	$-7 \leq h \leq 14$, $-16 \leq k \leq 12$, $-44 \leq l \leq 43$
Reflections collected	31888
Independent reflections	11193 [R_{int} = 0.0410, R_{sigma} = 0.0503]
Data/restraints/parameters	11193/0/867
Goodness-of-fit on F^2	1.099
Final R indexes [I>=2σ (I)]	R_1 = 0.0504, wR_2 = 0.1366
Final R indexes [all data]	R_1 = 0.0640, wR_2 = 0.1435
Largest diff. peak/hole / e Å^{-3}	1.50/-1.12
CCDC-number	2501763

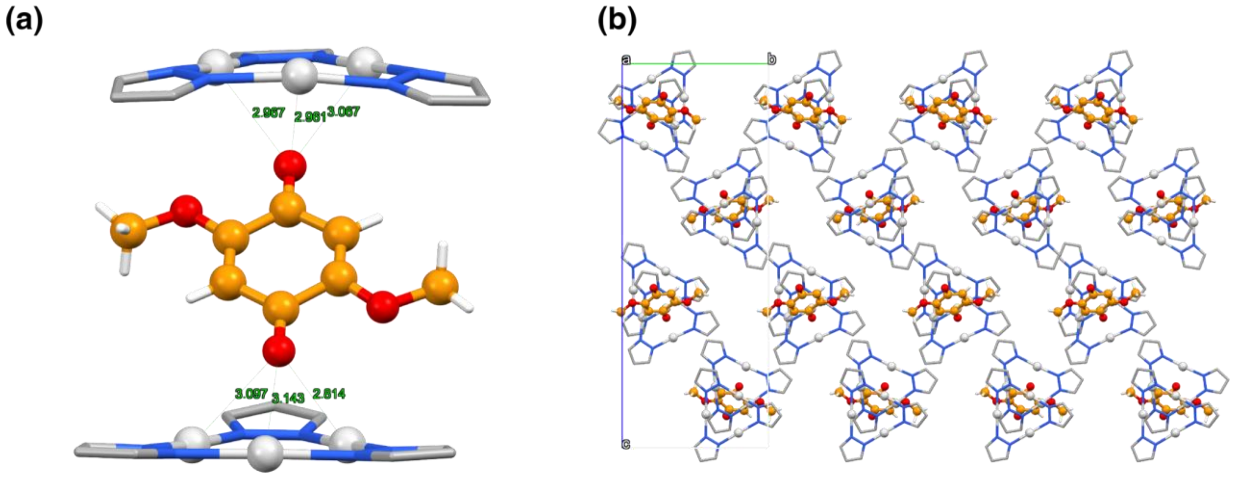

Figure S23. (a) A schematic diagram of the co-crystal structure in the **Ag_3Pz_3·2** single crystal, formed by the guest organic molecule and the surrounding Ag_3Pz_3 units that exhibit significant interactions with it. (b) A 1 × 4 × 1 packing mode in the single crystal structure of **Ag_3Pz_3·2** along the *a* axis. Trifluoromethyl groups and H atoms in Ag_3Pz_3 are omitted for clarity. Ag···O interactions are indicated with green dotted lines with distances in Å. C, N, and Ag atoms in Ag_3Pz_3 are depicted in dark gray, light blue, and light gray, respectively; C, O, and H atoms in **2** are depicted in orange, red, and white, respectively.

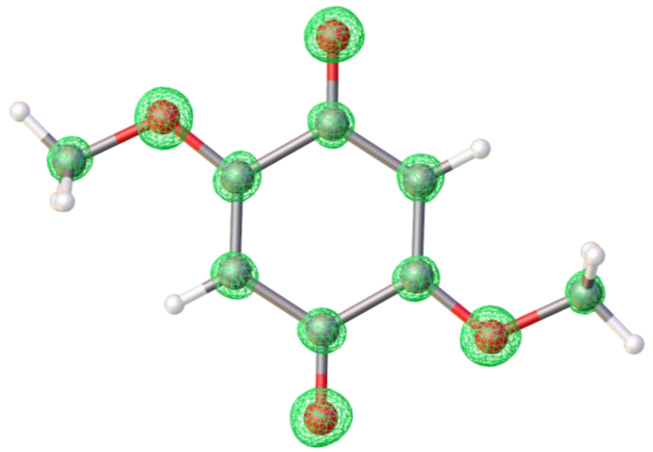

Figure S24. F_{obs} (contour: 0.30) electron density map superimposed on the structure of **2** in the single crystal structure of **Ag_3Pz_3·2**.

Preparation of Ag_3Pz_3·3. 1.80 mg (0.0107 mmol) of 2,6-dimethoxy-1,4-benzoquinone (**3**) was dissolved in 3 mL of a binary solvent system of DCM and n-Hex (1:1, v/v), followed by the addition of equimolar amounts of Ag_3Pz_3 (10.00 mg, 0.0107 mmol). The resulting mixed solution was filtered and then transferred to a 20 mL screw-capped sample vial. The cap of the sample vial was loosely closed to allow the solvent to slowly evaporate at room temperature. The entire co-crystal incubation process was protected from light using aluminum foil. After the designated evaporation period, typically 1-3 days, high-quality yellow block-shaped crystals suitable for single-crystal X-ray diffraction analysis formed at the bottom of the vial.

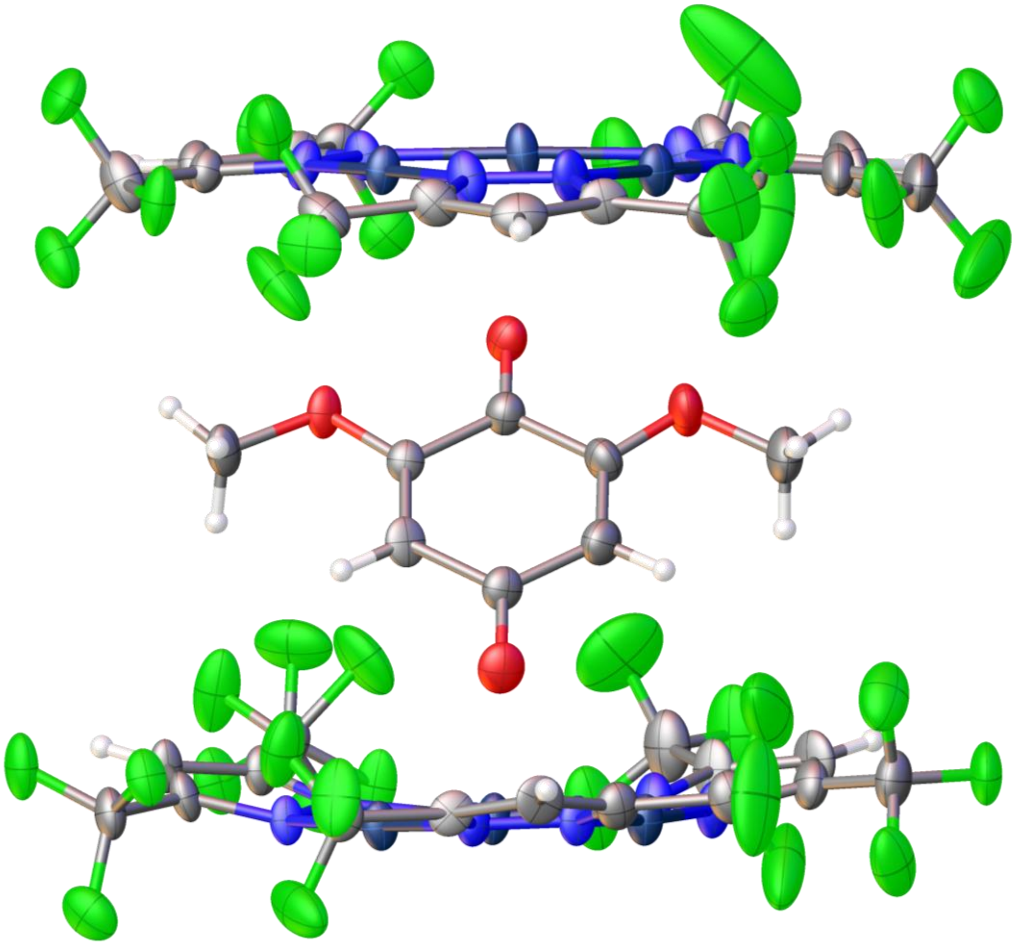

Figure S25. Asymmetric unit of **Ag_3Pz_3·3** (thermal displacement parameters at the 50% probability level).

Table S11. Crystal data and structure refinement for **Ag_3Pz_3·3**

Empirical formula	$C_{38}H_{14}Ag_6F_{36}N_{12}O_4$
Formula weight	2033.83
Temperature/K	100.03(18)
Crystal system	triclinic
Space group	$P\bar{1}$
a/Å	11.5217(3)
b/Å	12.1025(2)
c/Å	22.8839(4)
α/°	91.076(2)
β/°	92.848(2)
γ/°	117.691(2)
Volume/Å^3	2818.84(11)
Z	2
ρ_{calc}g/cm^3	2.396
μ/mm^{-1}	18.011
F(000)	1928.0
Crystal size/mm^3	0.156 × 0.146 × 0.14
Radiation	Cu Kα (λ = 1.54184)
2θ range for data collection/°	7.744 to 156.114
Index ranges	$-14 \le h \le 14$, $-15 \le k \le 12$, $-28 \le l \le 28$
Reflections collected	27265
Independent reflections	11446 [R_{int} = 0.0480, R_{sigma} = 0.0552]
Data/restraints/parameters	11446/198/882
Goodness-of-fit on F^2	1.046
Final R indexes [I>=2σ (I)]	R_1 = 0.0728, wR_2 = 0.1831
Final R indexes [all data]	R_1 = 0.0886, wR_2 = 0.1901
Largest diff. peak/hole / e Å^{-3}	1.80/-1.47
CCDC-number	2501774

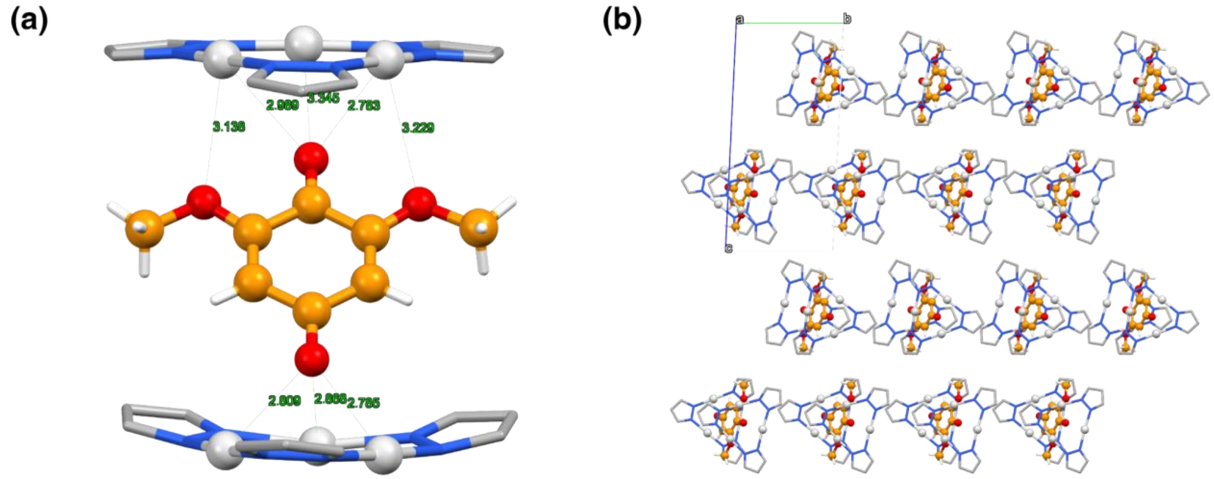

Figure S26. (a) A schematic diagram of the co-crystal structure in the **Ag_3Pz_3·3** single crystal, formed by the guest organic molecule and the surrounding Ag_3Pz_3 units that exhibit significant interactions with it. (b) A 1 × 4 × 2 packing mode in the single crystal structure of **Ag_3Pz_3·3** along the *a* axis. Trifluoromethyl groups and H atoms in Ag_3Pz_3 are omitted for clarity. Ag···O interactions are indicated with green dotted lines with distances in Å. C, N, and Ag atoms in Ag_3Pz_3 are depicted in dark gray, light blue, and light gray, respectively; C, O, and H atoms in **3** are depicted in orange, red, and white, respectively.

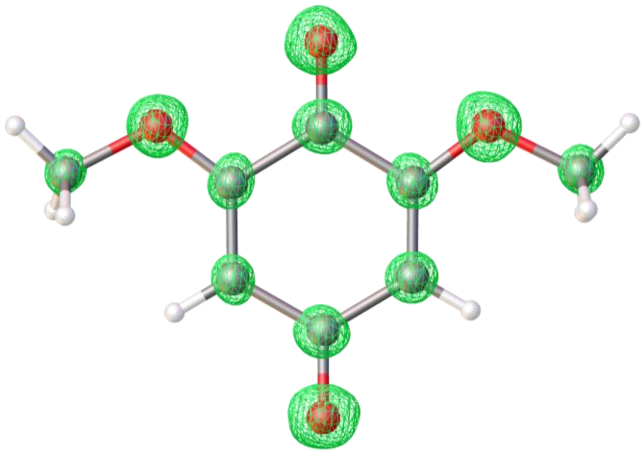

Figure S27. F_{obs} (contour: 0.80) electron density map superimposed on the structure of **3** in the single crystal structure of **Ag_3Pz_3·3**.

Preparation of Ag_3Pz_3·4. 1.95 mg (0.0107 mmol) of 2,3-dimethoxy-5-methyl-1,4-benzoquinone (**4**) was dissolved in 3 mL of c-Hex, followed by the addition of equimolar amounts of Ag_3Pz_3 (10.00 mg, 0.0107 mmol). The resulting mixed solution was filtered and then transferred to a 20 mL screw-capped sample vial. The cap of the sample vial was loosely closed to allow the solvent to slowly evaporate at room temperature. The entire co-crystal incubation process was protected from light using aluminum foil. After the designated evaporation period, typically 1-3 days, high-quality orange block-shaped crystals suitable for single-crystal X-ray diffraction analysis formed at the bottom of the vial.

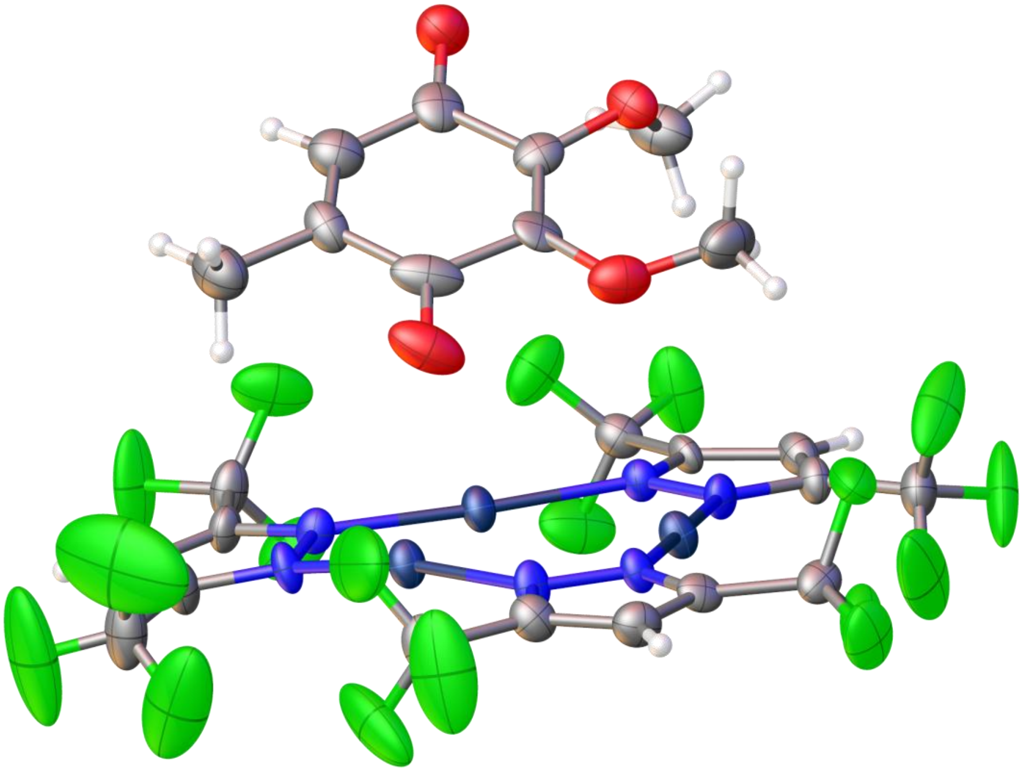

Figure S28. Asymmetric unit of **Ag_3Pz_3·4** (thermal displacement parameters at the 50% probability level).

Table S12. Crystal data and structure refinement for **Ag_3Pz_3·4**

Empirical formula	$C_{24}H_{13}Ag_3F_{18}N_6O_4$
Formula weight	1115.01
Temperature/K	99.99(10)
Crystal system	monoclinic
Space group	$P2_1/n$
a/Å	8.6774(2)
b/Å	9.1450(2)
c/Å	41.2028(6)
α/°	90
β/°	93.794(2)
γ/°	90
Volume/Å^3	3262.48(11)
Z	4
ρ_{calc}g/cm^3	2.270
μ/mm^{-1}	15.692
F(000)	2136.0
Crystal size/mm^3	0.2 × 0.1 × 0.1
Radiation	Cu Kα (λ = 1.54184)
2θ range for data collection/°	9.908 to 155.46
Index ranges	$-10 \le h \le 10$, $-11 \le k \le 7$, $-48 \le l \le 52$
Reflections collected	18795
Independent reflections	6640 [R_{int} = 0.0480, R_{sigma} = 0.0515]
Data/restraints/parameters	6640/18/499
Goodness-of-fit on F^2	1.092
Final R indexes [I>=2σ (I)]	R_1 = 0.0715, wR_2 = 0.1695
Final R indexes [all data]	R_1 = 0.0856, wR_2 = 0.1746
Largest diff. peak/hole / e Å^{-3}	2.04/-1.48
CCDC-number	2501785

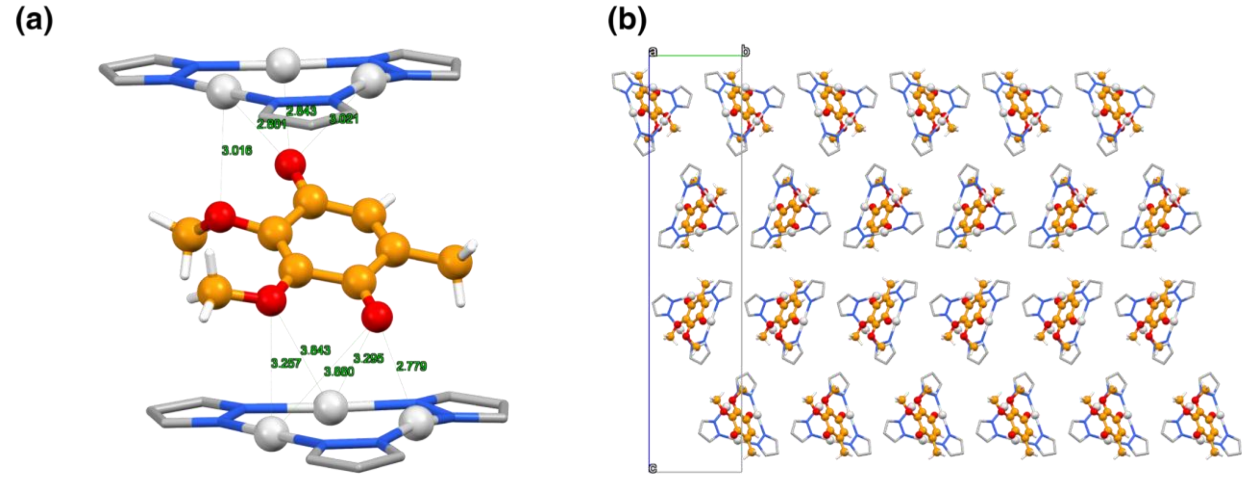

Figure S29. (a) A schematic diagram of the co-crystal structure in the **Ag_3Pz_3·4** single crystal, formed by the guest organic molecule and the surrounding Ag_3Pz_3 units that exhibit significant interactions with it. (b) A 1 × 6 × 1 packing mode in the single crystal structure of **Ag_3Pz_3·4** along the *a* axis. Trifluoromethyl groups and H atoms in Ag_3Pz_3 are omitted for clarity. Ag···O interactions are indicated with green dotted lines with distances in Å. C, N, and Ag atoms in Ag_3Pz_3 are depicted in dark gray, light blue, and light gray, respectively; C, O, and H atoms in **4** are depicted in orange, red, and white, respectively.

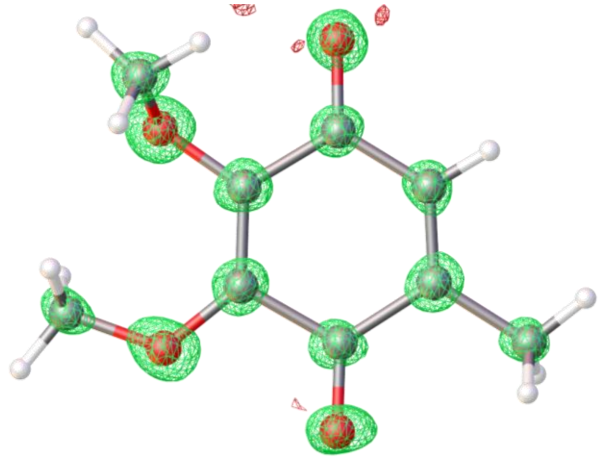

Figure S30. F_{obs} (contour: 0.80) electron density map superimposed on the structure of **4** in the single crystal structure of **Ag_3Pz_3·4**.

Preparation of Ag_3Pz_3·5. 1.76 mg (0.0107 mmol) of thymoquinone (**5**) was dissolved in 3 mL of a binary solvent system of n-Hex and c-Hex (1:1, v/v), followed by the addition of equimolar amounts of Ag_3Pz_3 (10.00 mg, 0.0107 mmol). The resulting mixed solution was filtered and then transferred to a 20 mL screw-capped sample vial. The cap of the sample vial was loosely closed to allow the solvent to slowly evaporate at room temperature. The entire co-crystal incubation process was protected from light using aluminum foil. After the designated evaporation period, typically 1-3 days, high-quality yellow plate-shaped crystals suitable for single-crystal X-ray diffraction analysis formed at the bottom of the vial.

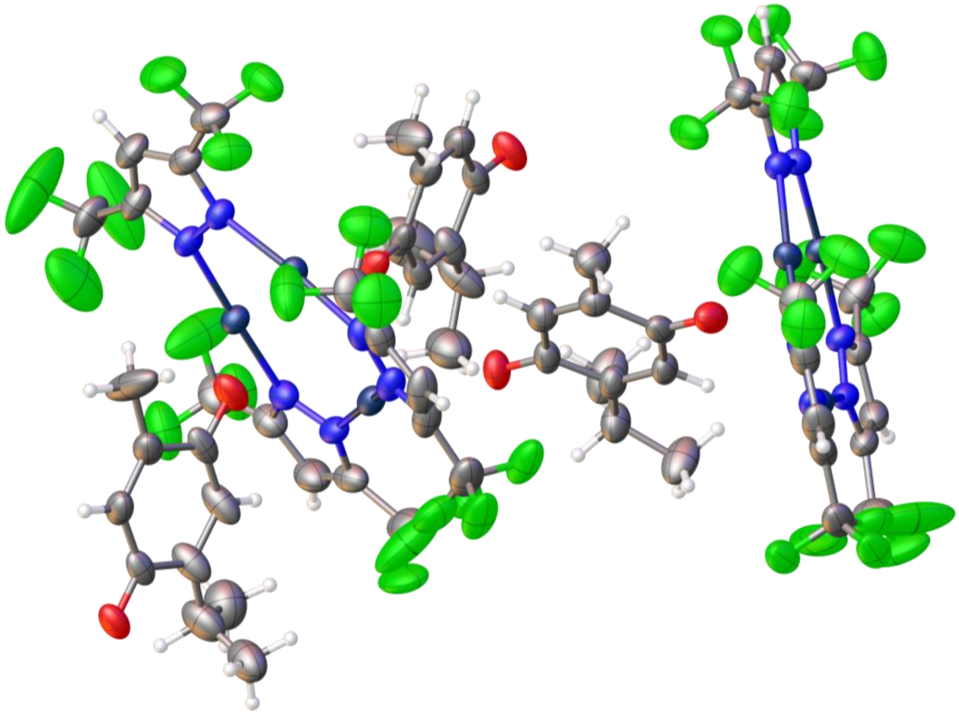

Figure S31. Asymmetric unit of **Ag_3Pz_3·5** (thermal displacement parameters at the 50% probability level).

Table S13. Crystal data and structure refinement for $\mathbf{Ag_3Pz_3{\cdot}5}$

Empirical formula	$C_{60}H_{42}Ag_6F_{36}N_{12}O_6$
Formula weight	2358.27
Temperature/K	100.0(3)
Crystal system	monoclinic
Space group	$P2_1/c$
a/Å	10.02080(10)
b/Å	34.2509(3)
c/Å	22.8960(2)
α/°	90
β/°	98.5440(10)
γ/°	90
Volume/Å^3	7771.19(13)
Z	4
ρ_{calc}g/cm^3	2.016
μ/mm^{-1}	13.200
F(000)	4560.0
Crystal size/mm^3	0.43 × 0.25 × 0.25
Radiation	Cu Kα (λ = 1.54184)
2θ range for data collection/°	4.678 to 155.926
Index ranges	$-12 \leq h \leq 8$, $-37 \leq k \leq 43$, $-29 \leq l \leq 28$
Reflections collected	43420
Independent reflections	15848 [R_{int} = 0.0414, R_{sigma} = 0.0478]
Data/restraints/parameters	15848/6/1090
Goodness-of-fit on F^2	1.065
Final R indexes [I>=2σ (I)]	R_1 = 0.0504, wR_2 = 0.1369
Final R indexes [all data]	R_1 = 0.0619, wR_2 = 0.1441
Largest diff. peak/hole / e Å^{-3}	1.84/-0.74
CCDC-number	2501796

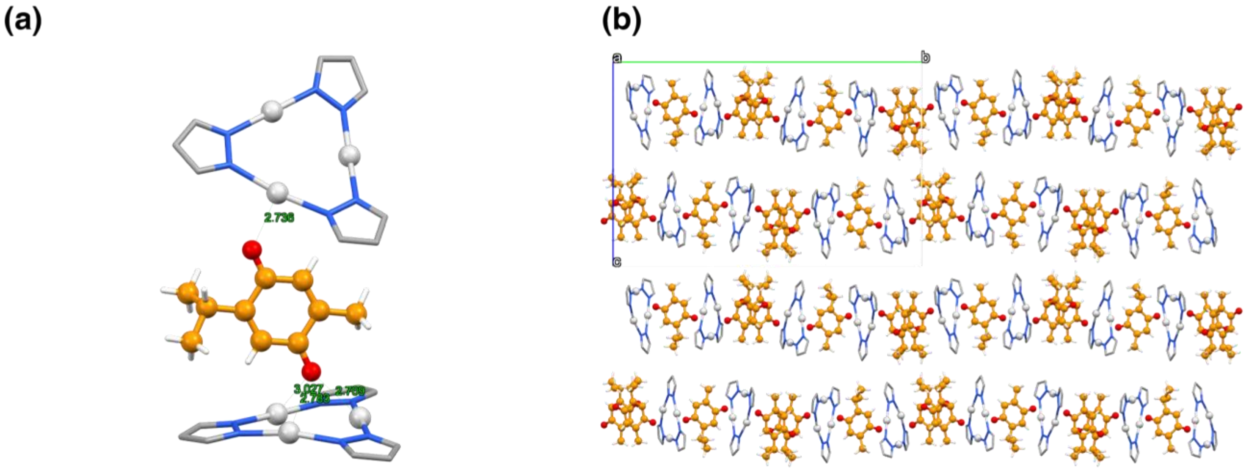

Figure S32. (a) A schematic diagram of the co-crystal structure in the **Ag_3Pz_3·5** single crystal, formed by the guest organic molecule and the surrounding Ag_3Pz_3 units that exhibit significant interactions with it. (b) A 1 × 2 × 2 packing mode in the single crystal structure of **Ag_3Pz_3·5** along the *a* axis. Trifluoromethyl groups and H atoms in Ag_3Pz_3 are omitted for clarity. Ag···O interactions are indicated with green dotted lines with distances in Å. C, N, and Ag atoms in Ag_3Pz_3 are depicted in dark gray, light blue, and light gray, respectively; C, O, and H atoms in **5** are depicted in orange, red, and white, respectively.

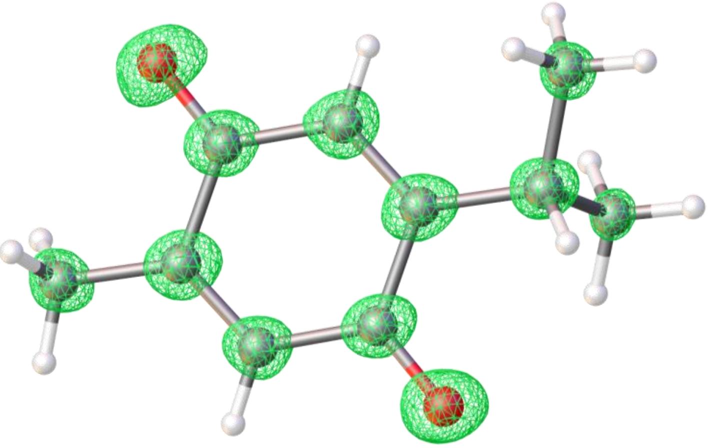

Figure S33. F_{obs} (contour: 0.40) electron density map superimposed on the structure of **5** in the single crystal structure of **Ag_3Pz_3·5**.

Preparation of Ag_3Pz_3·6. 1.76 mg (0.0107 mmol) of duroquinone (**6**) was dissolved in 3 mL of a binary solvent system of DCM and n-Hex (1:1, v/v), followed by the addition of equimolar amounts of Ag_3Pz_3 (10.00 mg, 0.0107 mmol). The resulting mixed solution was filtered and then transferred to a 20 mL screw-capped sample vial. The cap of the sample vial was loosely closed to allow the solvent to slowly evaporate at room temperature. The entire co-crystal incubation process was protected from light using aluminum foil. After the designated evaporation period, typically 1-3 days, high-quality yellow plate-shaped crystals suitable for single-crystal X-ray diffraction analysis formed at the bottom of the vial.

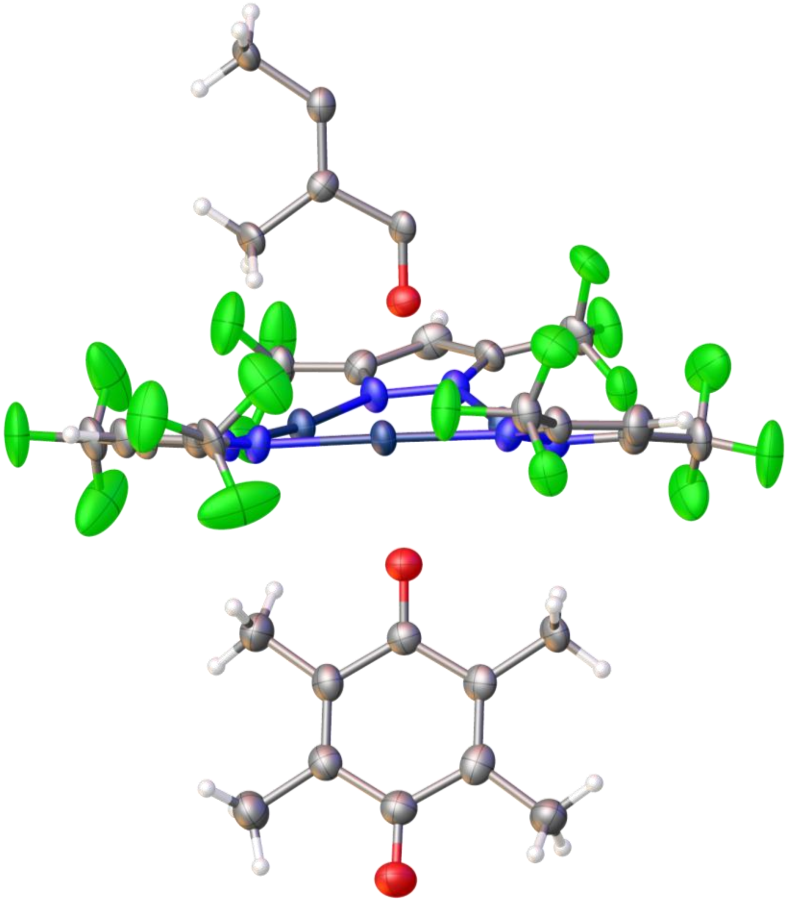

Figure S34. Asymmetric unit of **Ag_3Pz_3·6** (thermal displacement parameters at the 50% probability level).

Table S14. Crystal data and structure refinement for **$Ag_3Pz_3·6$**

Empirical formula	$C_{30}H_{21}Ag_3F_{18}N_6O_3$
Formula weight	1179.14
Temperature/K	1 00.1(4)
Crystal system	triclinic
Space group	$P\bar{1}$
a/Å	9.1547(3)
b/Å	11.6982(3)
c/Å	18.6879(3)
α/°	102.603(2)
β/°	98.938(2)
γ/°	100.501(2)
Volume/Å^3	1880.29(9)
Z	2
ρ_{calc}g/cm^3	2.083
μ/mm^{-1}	13.639
F(000)	1140.0
Crystal size/mm^3	0.3 × 0.2 × 0.1
Radiation	Cu Kα (λ = 1.54184)
2θ range for data collection/°	4.948 to 133.198
Index ranges	$-10 \leq h \leq 7$, $-10 \leq k \leq 13$, $-21 \leq l \leq 22$
Reflections collected	14701
Independent reflections	6558 [R_{int} = 0.0361, R_{sigma} = 0.0433]
Data/restraints/parameters	6558/0/547
Goodness-of-fit on F^2	1.090
Final R indexes [I>=2σ (I)]	R_1 = 0.0600, wR_2 = 0.1652
Final R indexes [all data]	R_1 = 0.0686, wR_2 = 0.1734
Largest diff. peak/hole / e Å^{-3}	2.57/-0.89
CCDC-number	2501807

Responses to CheckCIF alerts for Ag_3Pz_3·6 crystal structure:

(There is no A-level alert)

B-level alerts:

"Check Calcd Resid. Dens. 1.01Ang From Ag01 2.65 eA-3"

This Alert is due to presence of residual density in the presence of heavy metal atom (Ag).

"Check Calcd Resid. Dens. 1.05Ang From Ag03 2.53 eA-3"

This Alert is due to presence of residual density in the presence of heavy metal atom (Ag).

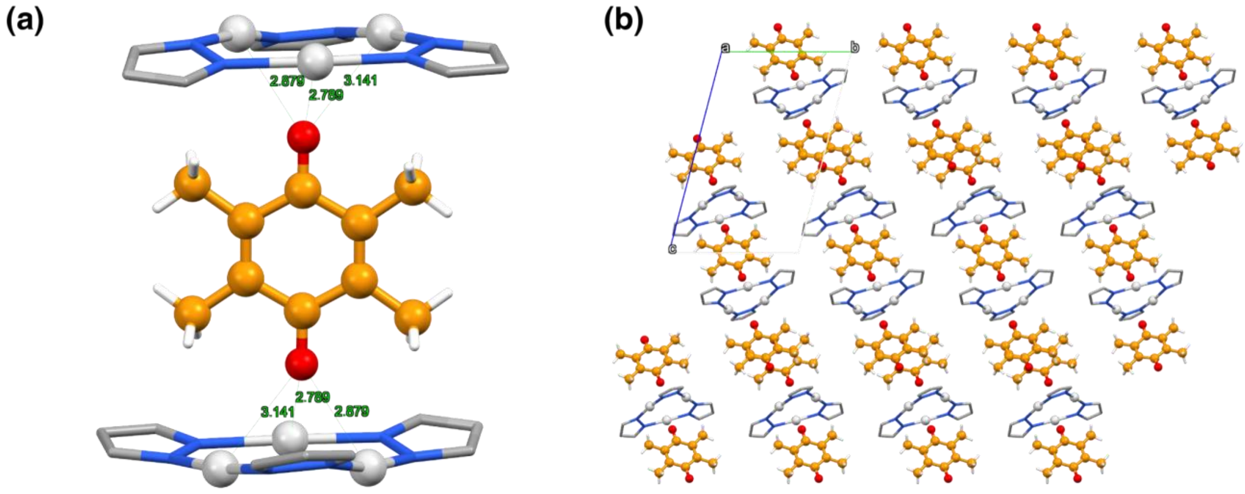

Figure S35. (a) A schematic diagram of the co-crystal structure in the $\mathbf{Ag_3Pz_3{\cdot}6}$ single crystal, formed by the guest organic molecule and the surrounding Ag_3Pz_3 units that exhibit significant interactions with it. (b) A 1 × 4 × 2 packing mode in the single crystal structure of $\mathbf{Ag_3Pz_3{\cdot}6}$ along the *a* axis. Trifluoromethyl groups and H atoms in Ag_3Pz_3 are omitted for clarity. Ag···O interactions are indicated with green dotted lines with distances in Å. C, N, and Ag atoms in Ag_3Pz_3 are depicted in dark gray, light blue, and light gray, respectively; C, O, and H atoms in **6** are depicted in orange, red, and white, respectively.

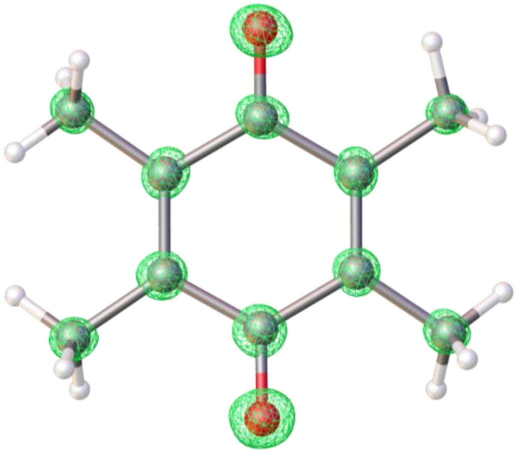

Figure S36. F_{obs} (contour: 1.10) electron density map superimposed on the structure of **6** in the single crystal structure of $\mathbf{Ag_3Pz_3{\cdot}6}$.

Preparation of Ag_3Pz_3·7. 1.63 mg (0.0107 mmol) of 4-oxoisophorone (**7**) was dissolved in 3 mL of a binary solvent system of n-Hex and c-Hex (1:1, v/v), followed by the addition of equimolar amounts of Ag_3Pz_3 (10.00 mg, 0.0107 mmol). The resulting mixed solution was filtered and then transferred to a 20 mL screw-capped sample vial. The cap of the sample vial was loosely closed to allow the solvent to slowly evaporate at room temperature. The entire co-crystal incubation process was protected from light using aluminum foil. After the designated evaporation period, typically 1-3 days, high-quality colorless block-shaped crystals suitable for single-crystal X-ray diffraction analysis formed at the bottom of the vial.

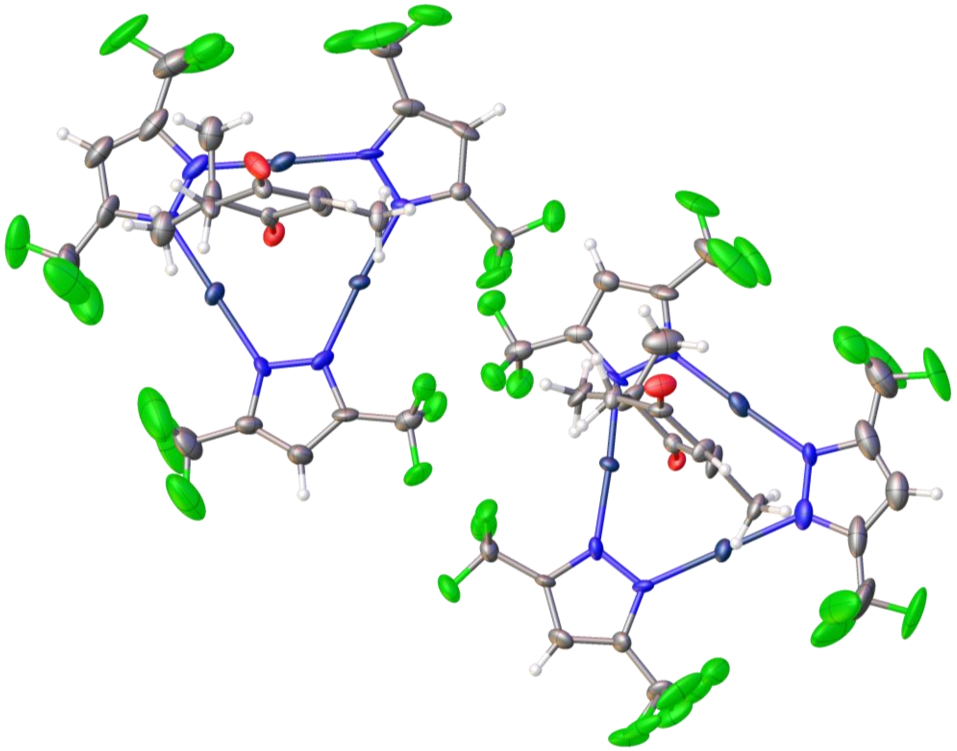

Figure S37. Asymmetric unit of **Ag_3Pz_3·7** (thermal displacement parameters at the 50% probability level).

Table S15. Crystal data and structure refinement for **Ag_3Pz_3·7**

Empirical formula	$C_{24}H_{15}Ag_3F_{18}N_6O_2$
Formula weight	1085.03
Temperature/K	99.99(14)
Crystal system	orthorhombic
Space group	$Pna2_1$
a/Å	18.24293(11)
b/Å	9.33640(5)
c/Å	39.6212(2)
α/°	90
β/°	90
γ/°	90
Volume/Å^3	6748.42(6)
Z	8
ρ_{calc}g/cm^3	2.136
μ/mm^{-1}	15.100
F(000)	4160.0
Crystal size/mm^3	0.15 × 0.14 × 0.12
Radiation	Cu Kα (λ = 1.54184)
2θ range for data collection/°	9.696 to 156.718
Index ranges	$-22 \leq h \leq 12$, $-9 \leq k \leq 11$, $-50 \leq l \leq 50$
Reflections collected	28744
Independent reflections	12664 [R_{int} = 0.0278, R_{sigma} = 0.0313]
Data/restraints/parameters	12664/422/910
Goodness-of-fit on F^2	1.084
Final R indexes [I>=2σ (I)]	R_1 = 0.0765, wR_2 = 0.2039
Final R indexes [all data]	R_1 = 0.0770, wR_2 = 0.2045
Largest diff. peak/hole / e Å^{-3}	1.68/-1.44
Flack parameter	0.037(17)
CCDC-number	2501818

Responses to CheckCIF alert for Ag_3Pz_3·7 crystal structure:

(There is no A-level alert)

B-level alert:

"Coordinates do not Form a Properly Connected Set   Please Do !"

The alert is due to a large number of co-crystallized molecules in the which sometimes do not show as a connected set. This is acceptable from a crystallographic point of view.

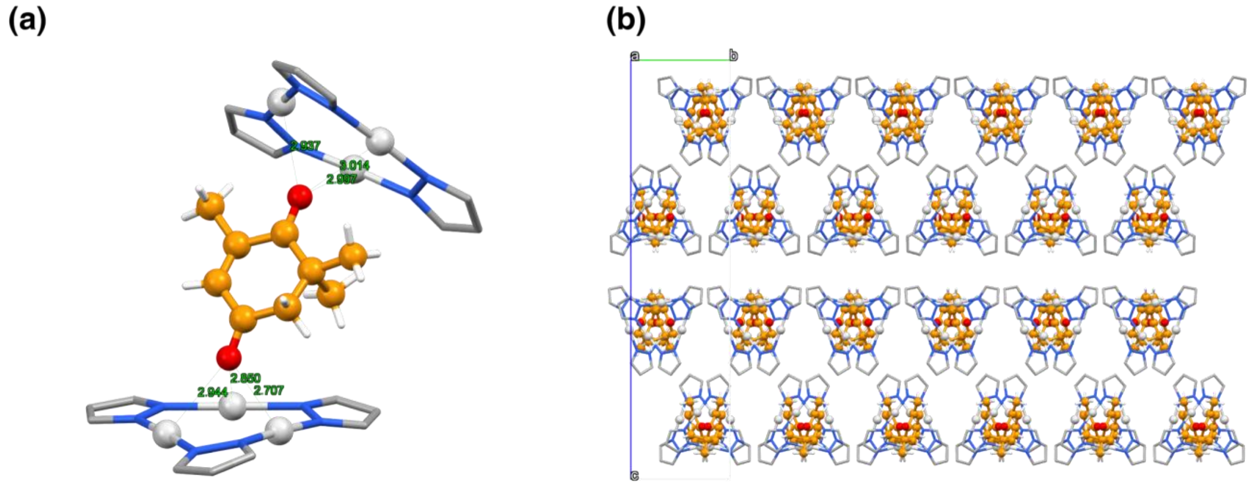

Figure S38. (a) A schematic diagram of the co-crystal structure in the $\mathbf{Ag_3Pz_3{\cdot}7}$ single crystal, formed by the guest organic molecule and the surrounding Ag_3Pz_3 units that exhibit significant interactions with it. (b) A 1 × 6 × 1 packing mode in the single crystal structure of $\mathbf{Ag_3Pz_3{\cdot}7}$ along the *a* axis. Trifluoromethyl groups and H atoms in Ag_3Pz_3 are omitted for clarity. Ag···O interactions are indicated with green dotted lines with distances in Å. C, N, and Ag atoms in Ag_3Pz_3 are depicted in dark gray, light blue, and light gray, respectively; C, O, and H atoms in **7** are depicted in orange, red, and white, respectively.

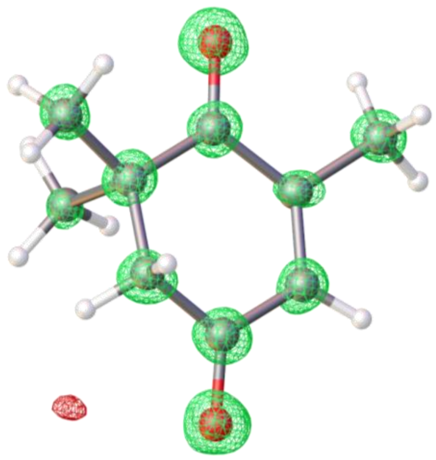

Figure S39. F_{obs} (contour: 0.60) electron density map superimposed on the structure of **7** in the single crystal structure of $\mathbf{Ag_3Pz_3{\cdot}7}$.

Preparation of Ag_3Pz_3·8. 1.18 mg (0.0107 mmol) of norcamphor (**8**) was dissolved in 3 mL of a binary solvent system of DCM and c-Hex (1:1, v/v), followed by the addition of equimolar amounts of Ag_3Pz_3 (10.00 mg, 0.0107 mmol). The resulting mixed solution was filtered and then transferred to a 20 mL screw-capped sample vial. The cap of the sample vial was loosely closed to allow the solvent to slowly evaporate at room temperature. The entire co-crystal incubation process was protected from light using aluminum foil. After the designated evaporation period, typically 1-3 days, high-quality colorless block-shaped crystals suitable for single-crystal X-ray diffraction analysis formed at the bottom of the vial.

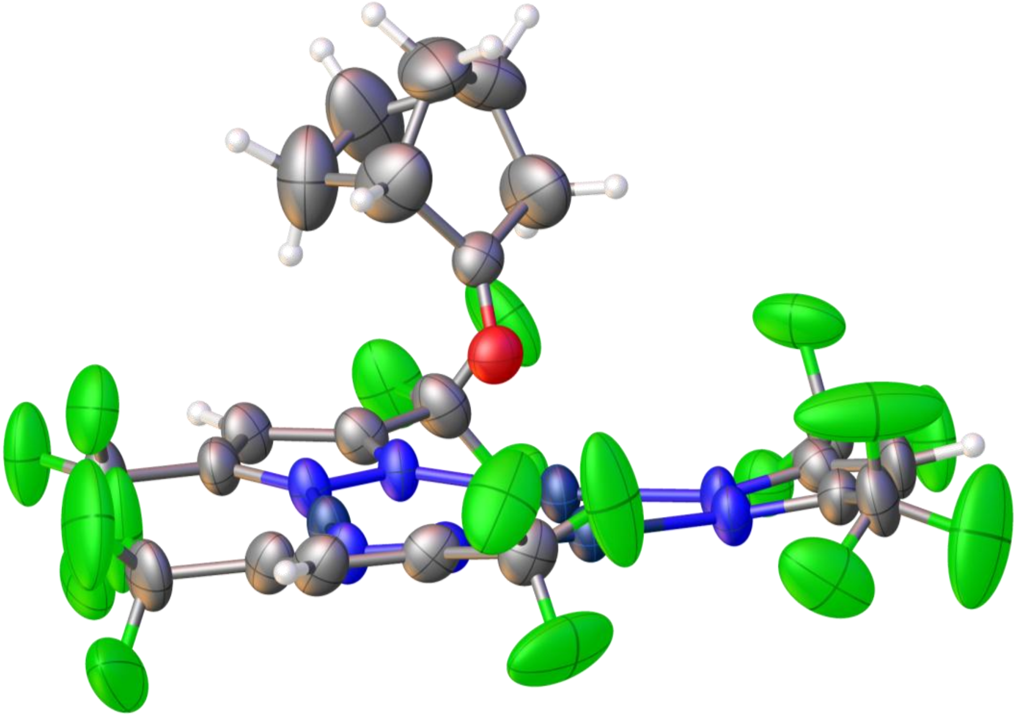

Figure S40. Asymmetric unit of **Ag_3Pz_3·8** (thermal displacement parameters at the 50% probability level).

Table S16. Crystal data and structure refinement for **Ag_3Pz_3·8**

Empirical formula	$C_{22}H_{13}Ag_3F_{18}N_6O$
Formula weight	1042.99
Temperature/K	100.00(10)
Crystal system	triclinic
Space group	$P\bar{1}$
a/Å	10.1664(3)
b/Å	12.0268(4)
c/Å	13.4008(4)
α/°	72.917(3)
β/°	75.561(3)
γ/°	78.910(2)
Volume/Å^3	1504.14(9)
Z	2
ρ_{calc}g/cm^3	2.303
μ/mm^{-1}	16.872
F(000)	996.0
Crystal size/mm^3	0.23 × 0.21 × 0.16
Radiation	Cu Kα (λ = 1.54184)
2θ range for data collection/°	7.052 to 156.26
Index ranges	$-12 \leq h \leq 8$, $-15 \leq k \leq 14$, $-16 \leq l \leq 16$
Reflections collected	13601
Independent reflections	6095 [R_{int} = 0.0504, R_{sigma} = 0.0602]
Data/restraints/parameters	6095/23/451
Goodness-of-fit on F^2	1.230
Final R indexes [I>=2σ (I)]	R_1 = 0.0788, wR_2 = 0.1820
Final R indexes [all data]	R_1 = 0.1002, wR_2 = 0.1883
Largest diff. peak/hole / e Å^{-3}	1.46/-1.12
CCDC-number	2501820

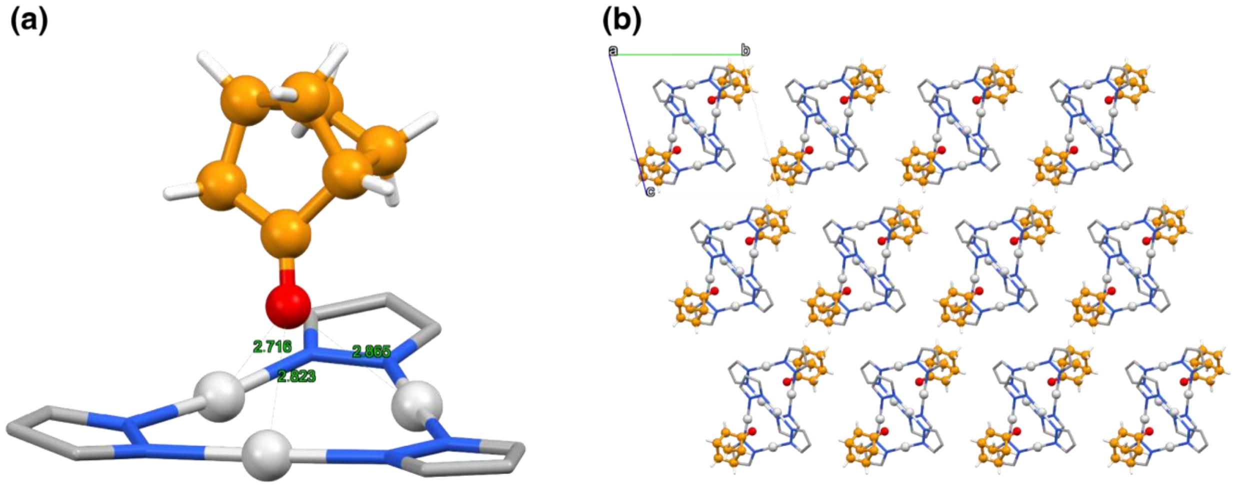

Figure S41. (a) A schematic diagram of the co-crystal structure in the $\mathbf{Ag_3Pz_3\cdot 8}$ single crystal, formed by the guest organic molecule and the surrounding Ag_3Pz_3 units that exhibit significant interactions with it. (b) A 1 × 4 × 3 packing mode in the single crystal structure of $\mathbf{Ag_3Pz_3\cdot 8}$ along the *a* axis. Trifluoromethyl groups and H atoms in Ag_3Pz_3 are omitted for clarity. Ag···O interactions are indicated with green dotted lines with distances in Å. C, N, and Ag atoms in Ag_3Pz_3 are depicted in dark gray, light blue, and light gray, respectively; C, O, and H atoms in **8** are depicted in orange, red, and white, respectively.

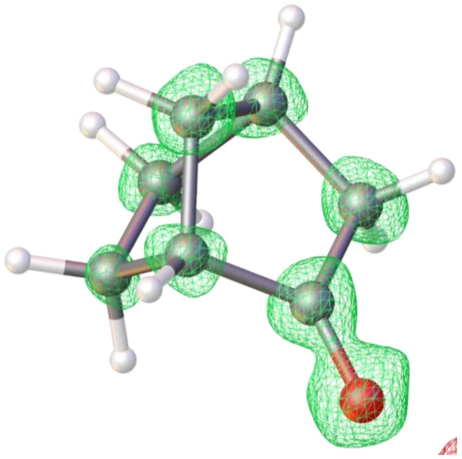

Figure S42. F_{obs} (contour: 0.90) electron density map superimposed on the structure of **8** in the single crystal structure of $\mathbf{Ag_3Pz_3\cdot 8}$.

Preparation of Ag_3Pz_3·9. 1.61 mg (0.0107 mmol) of (-)-verbenone (**9**) was dissolved in 3 mL of n-Hex, followed by the addition of equimolar amounts of Ag_3Pz_3 (10.00 mg, 0.0107 mmol). The resulting mixed solution was filtered and then transferred to a 20 mL screw-capped sample vial. The cap of the sample vial was loosely closed to allow the solvent to slowly evaporate at room temperature. The entire co-crystal incubation process was protected from light using aluminum foil. After the designated evaporation period, typically 1-3 days, high-quality colorless block-shaped crystals suitable for single-crystal X-ray diffraction analysis formed at the bottom of the vial.

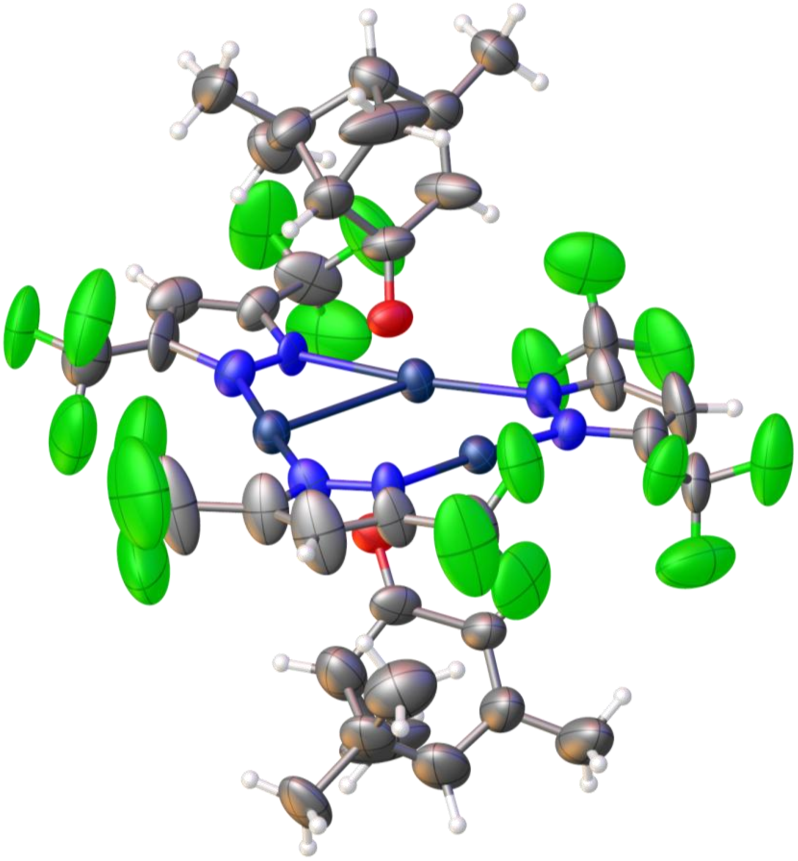

Figure S43. Asymmetric unit of **Ag_3Pz_3·9** (thermal displacement parameters at the 50% probability level).

Table S17. Crystal data and structure refinement for **$Ag_3Pz_3 \cdot 9$**

Empirical formula	$C_{35}H_{31}Ag_3F_{18}N_6O_2$
Formula weight	1233.27
Temperature/K	100.00(10)
Crystal system	monoclinic
Space group	$P2_1$
a/Å	9.57530(10)
b/Å	14.1910(2)
c/Å	16.1289(2)
α/°	90
β/°	98.2100(10)
γ/°	90
Volume/Å^3	2169.18(5)
Z	2
ρ_{calc}g/cm^3	1.888
μ/mm^{-1}	11.836
F(000)	1204.0
Crystal size/mm^3	0.19 × 0.16 × 0.13
Radiation	Cu Kα (λ = 1.54184)
2θ range for data collection/°	5.536 to 155.376
Index ranges	$-12 \leq h \leq 12$, $-9 \leq k \leq 17$, $-20 \leq l \leq 20$
Reflections collected	13600
Independent reflections	6581 [R_{int} = 0.0431, R_{sigma} = 0.0543]
Data/restraints/parameters	6581/267/577
Goodness-of-fit on F^2	1.060
Final R indexes [I>=2σ (I)]	R_1 = 0.0572, wR_2 = 0.1502
Final R indexes [all data]	R_1 = 0.0661, wR_2 = 0.1557
Largest diff. peak/hole / e Å^{-3}	1.16/-0.67
Flack parameter	-0.007(8)
CCDC-number	2501821

Responses to CheckCIF alerts for Ag_3Pz_3·9 crystal structure:

(There is no A-level alert)

B-level alerts:

“Low Bond Precision on C-C Bonds .... 0.0225 Ang.”

Disordered structure.

“No Flack x Check Done: Low Friedel Pair Coverage 45 %”

Due to insufficient data. The compound crystallizes in the chiral space group, but no chiral ligand is used, and the complex is no longer chiral.

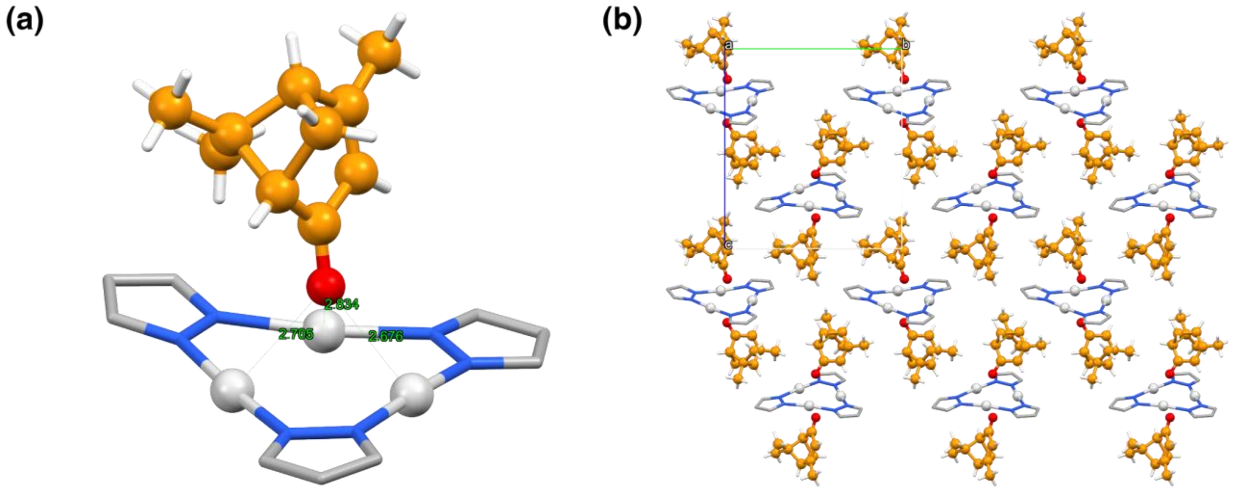

Figure S44. (a) A schematic diagram of the co-crystal structure in the $\mathbf{Ag_3Pz_3{\cdot}9}$ single crystal, formed by the guest organic molecule and the surrounding Ag_3Pz_3 units that exhibit significant interactions with it. (b) A 1 × 3 × 2 packing mode in the single crystal structure of $\mathbf{Ag_3Pz_3{\cdot}9}$ along the *a* axis. Trifluoromethyl groups and H atoms in Ag_3Pz_3 are omitted for clarity. Ag···O interactions are indicated with green dotted lines with distances in Å. C, N, and Ag atoms in Ag_3Pz_3 are depicted in dark gray, light blue, and light gray, respectively; C, O, and H atoms in **9** are depicted in orange, red, and white, respectively.

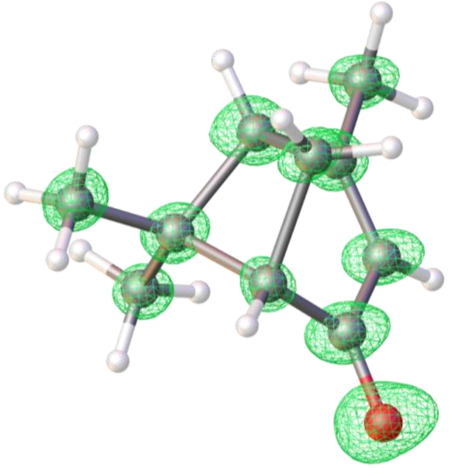

Figure S45. F_{obs} (contour: 0.80) electron density map superimposed on the structure of **9** in the single crystal structure of $\mathbf{Ag_3Pz_3{\cdot}9}$.

Preparation of Ag_3Pz_3·10. 1.80 mg (0.0107 mmol) of 1,3,5-trimethoxybenzene (**10**) was dissolved in 3 mL of a binary solvent system of DCM and n-Hex (1:1, v/v), followed by the addition of equimolar amounts of Ag_3Pz_3 (10.00 mg, 0.0107 mmol). The resulting mixed solution was filtered and then transferred to a 20 mL screw-capped sample vial. The cap of the sample vial was loosely closed to allow the solvent to slowly evaporate at room temperature. The entire co-crystal incubation process was protected from light using aluminum foil. After the designated evaporation period, typically 1-3 days, high-quality colorless needle-shaped crystals suitable for single-crystal X-ray diffraction analysis formed at the bottom of the vial.

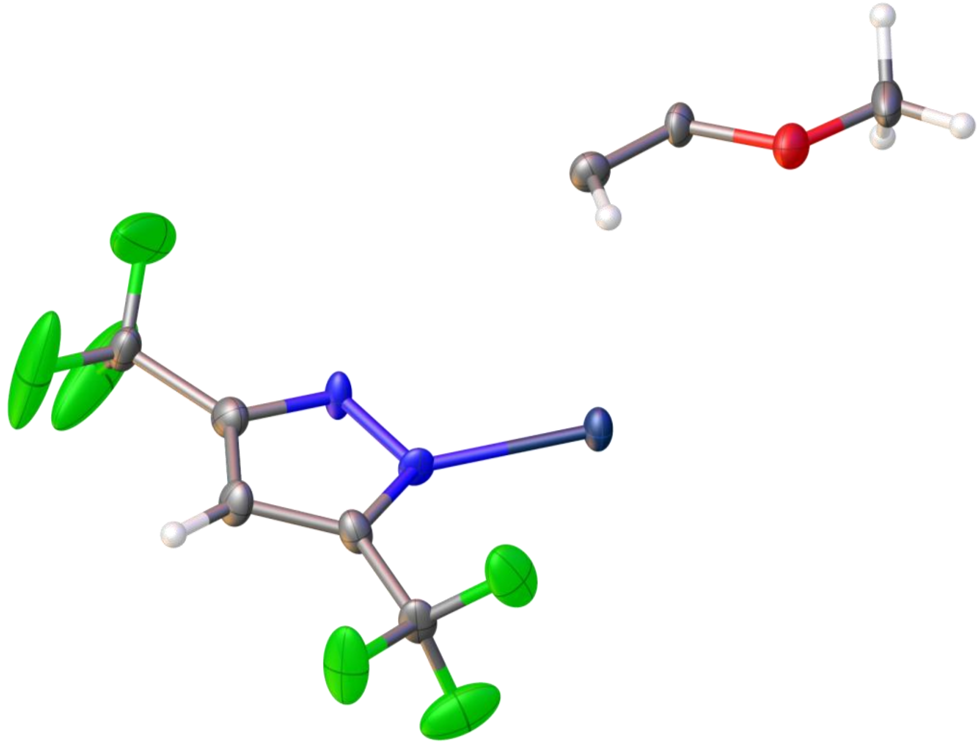

Figure S46. Asymmetric unit of **Ag_3Pz_3·10** (thermal displacement parameters at the 50% probability level).

Table S18. Crystal data and structure refinement for **Ag_3Pz_3·10**

Empirical formula	$C_8H_5AgF_6N_2O$
Formula weight	367.01
Temperature/K	100.0(2)
Crystal system	trigonal
Space group	*R*3*c*
a/Å	21.1388(2)
b/Å	21.1388(2)
c/Å	12.62900(10)
α/°	90
β/°	90
γ/°	120
Volume/$Å^3$	4887.20(10)
Z	18
$\rho_{calc}g/cm^3$	2.245
μ/mm^{-1}	15.675
F(000)	3168.0
Crystal size/mm^3	0.23 × 0.15 × 0.14
Radiation	Cu K*α* (*λ* = 1.54184)
2*θ* range for data collection/°	14.516 to 157.23
Index ranges	$-21 \leq h \leq 23$, $-13 \leq k \leq 23$, $-16 \leq l \leq 14$
Reflections collected	5315
Independent reflections	1908 [R_{int} = 0.0227, R_{sigma} = 0.0234]
Data/restraints/parameters	1908/1/164
Goodness-of-fit on F^2	1.087
Final *R* indexes [I>=2σ (I)]	R_1 = 0.0344, wR_2 = 0.0938
Final *R* indexes [all data]	R_1 = 0.0344, wR_2 = 0.0939
Largest diff. peak/hole / e $Å^{-3}$	0.88/-0.92
Flack parameter	-0.017(12)
CCDC-number	2501753

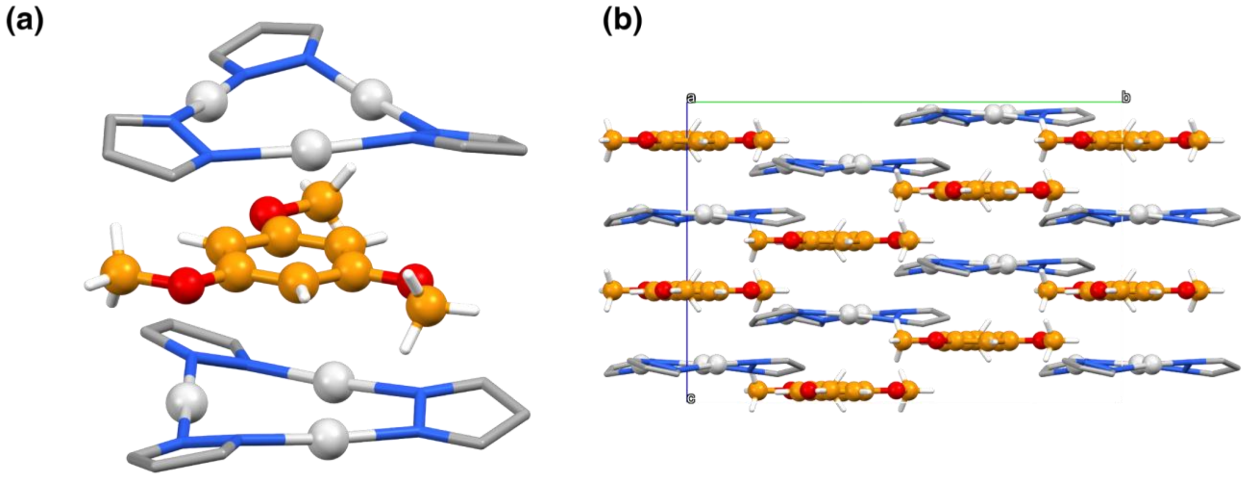

Figure S47. (a) A schematic diagram of the co-crystal structure in the $\mathbf{Ag_3Pz_3{\cdot}10}$ single crystal, formed by the guest organic molecule and the surrounding Ag_3Pz_3 units that exhibit significant interactions with it. (b) A 1 × 1 × 1 packing mode in the single crystal structure of $\mathbf{Ag_3Pz_3{\cdot}10}$ along the *a* axis. Trifluoromethyl groups and H atoms in Ag_3Pz_3 are omitted for clarity. C, N, and Ag atoms in Ag_3Pz_3 are depicted in dark gray, light blue, and light gray, respectively; C, O, and H atoms in **10** molecules are depicted in orange, red, and white, respectively.

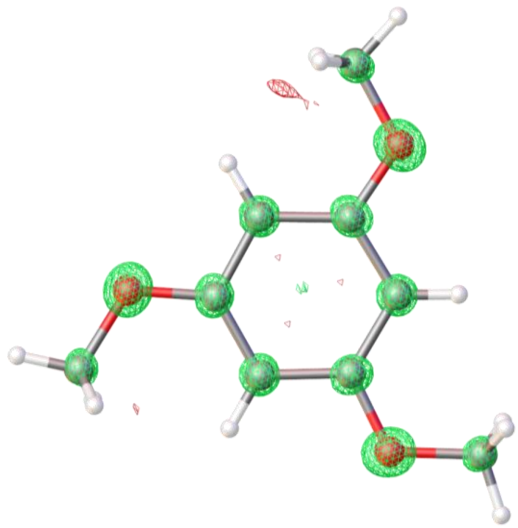

Figure S48. F_{obs} (contour: 0.48) electron density map superimposed on the structure of **10** in the single crystal structure of $\mathbf{Ag_3Pz_3{\cdot}10}$.

Preparation of Ag_3Pz_3·11. 2.10 mg (0.0107 mmol) of acetosyringone (**11**) was dissolved in 3 mL of a binary solvent system of DCM and n-Hex (1:1, v/v), followed by the addition of equimolar amounts of Ag_3Pz_3 (10.00 mg, 0.0107 mmol). The resulting mixed solution was filtered and then transferred to a 20 mL screw-capped sample vial. The cap of the sample vial was loosely closed to allow the solvent to slowly evaporate at room temperature. The entire co-crystal incubation process was protected from light using aluminum foil. After the designated evaporation period, typically 1-3 days, high-quality colorless needle-shaped crystals suitable for single-crystal X-ray diffraction analysis formed at the bottom of the vial.

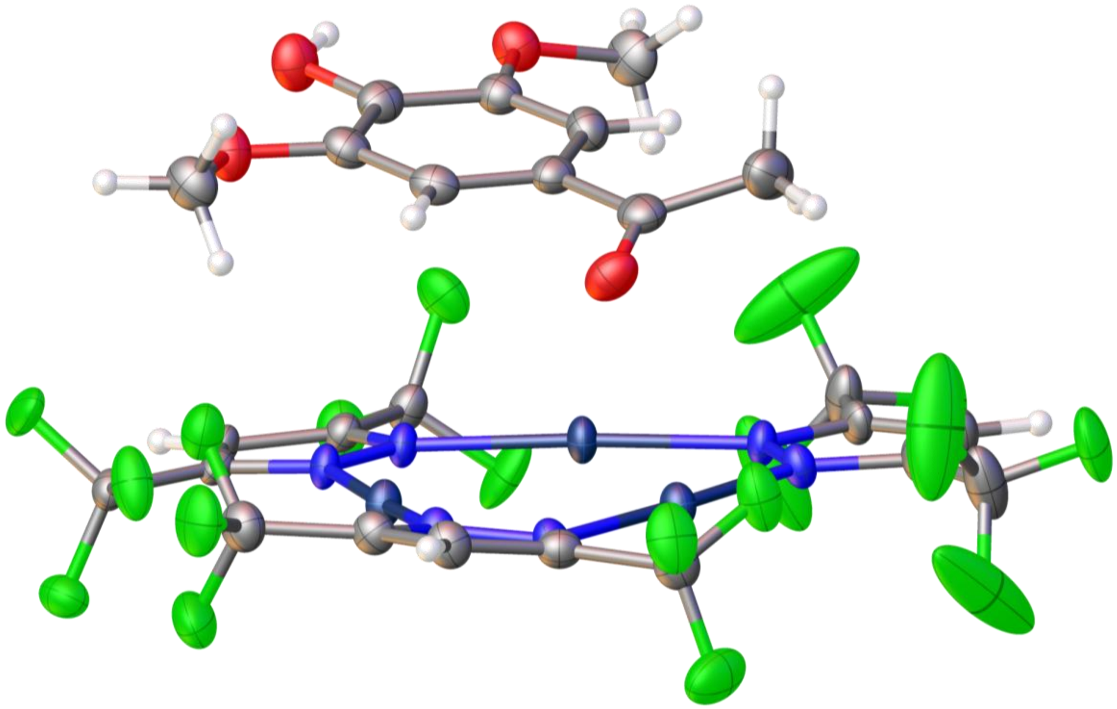

Figure S49. Asymmetric unit of **Ag_3Pz_3·11** (thermal displacement parameters at the 50% probability level).

Table S19. Crystal data and structure refinement for $\mathbf{Ag_3Pz_3{\cdot}11}$

Empirical formula	$C_{25}H_{15}Ag_3F_{18}N_6O_4$
Formula weight	1129.04
Temperature/K	100.0(2)
Crystal system	monoclinic
Space group	$P2_1/n$
a/Å	8.21540(10)
b/Å	16.71790(10)
c/Å	24.4730(2)
α/°	90
β/°	97.0010(10)
γ/°	90
Volume/Å^3	3336.16(5)
Z	4
ρ_{calc}g/cm^3	2.248
μ/mm^{-1}	15.356
F(000)	2168.0
Crystal size/mm^3	0.17 × 0.16 × 0.15
Radiation	Cu Kα (λ = 1.54184)
2θ range for data collection/°	6.418 to 156.866
Index ranges	$-10 \leq h \leq 10$, $-14 \leq k \leq 21$, $-27 \leq l \leq 30$
Reflections collected	21654
Independent reflections	6887 [R_{int} = 0.0286, R_{sigma} = 0.0278]
Data/restraints/parameters	6887/0/509
Goodness-of-fit on F^2	1.043
Final R indexes [I>=2σ (I)]	R_1 = 0.0362, wR_2 = 0.0885
Final R indexes [all data]	R_1 = 0.0395, wR_2 = 0.0898
Largest diff. peak/hole / e Å^{-3}	1.35/-1.12
CCDC-number	2501754

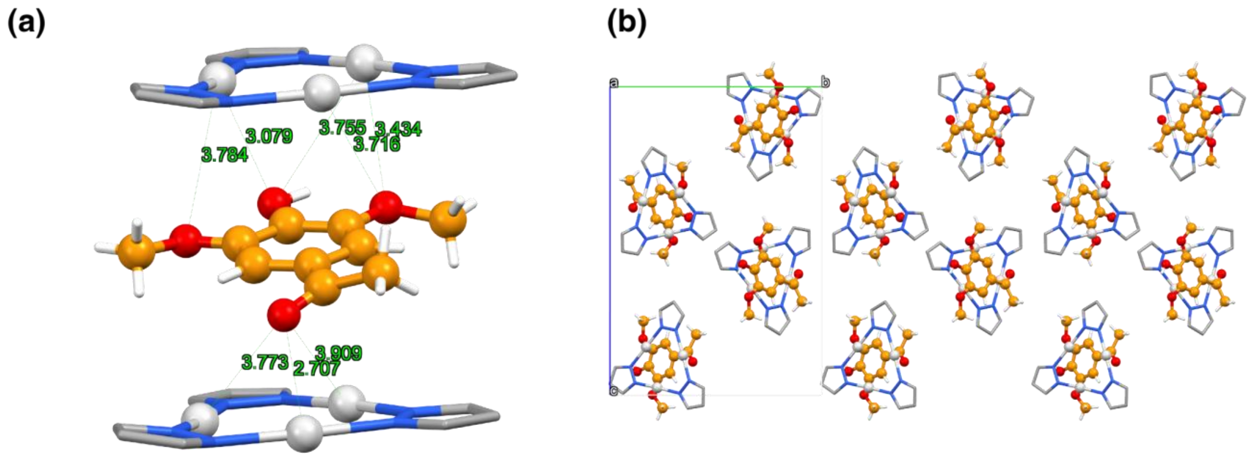

Figure S50. (a) A schematic diagram of the co-crystal structure in the **Ag_3Pz_3·11** single crystal, formed by the guest organic molecule and the surrounding Ag_3Pz_3 units that exhibit significant interactions with it. (b) A 1 × 3 × 1 packing mode in the single crystal structure of **Ag_3Pz_3·11** along the *a* axis. Trifluoromethyl groups and H atoms in Ag_3Pz_3 are omitted for clarity. Ag···O interactions are indicated with green dotted lines with distances in Å. C, N, and Ag atoms in Ag_3Pz_3 are depicted in dark gray, light blue, and light gray, respectively; C, O, and H atoms in **11** are depicted in orange, red, and white, respectively.

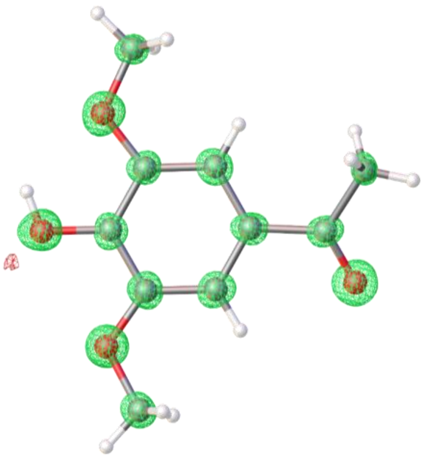

Figure S51. F_{obs} (contour: 0.95) electron density map superimposed on the structure of **11** in the single crystal structure of **Ag_3Pz_3·11**.

Preparation of Ag_3Pz_3·12. 2.25 mg (0.0107 mmol) of 2',4',6'-trimethoxyacetophenone (**12**) was dissolved in 3 mL of a binary solvent system of DCM and c-Hex (1:1, v/v), followed by the addition of equimolar amounts of Ag_3Pz_3 (10.00 mg, 0.0107 mmol). The resulting mixed solution was filtered and then transferred to a 20 mL screw-capped sample vial. The cap of the sample vial was loosely closed to allow the solvent to slowly evaporate at room temperature. The entire co-crystal incubation process was protected from light using aluminum foil. After the designated evaporation period, typically 1-3 days, high-quality colorless needle-shaped crystals suitable for single-crystal X-ray diffraction analysis formed at the bottom of the vial.

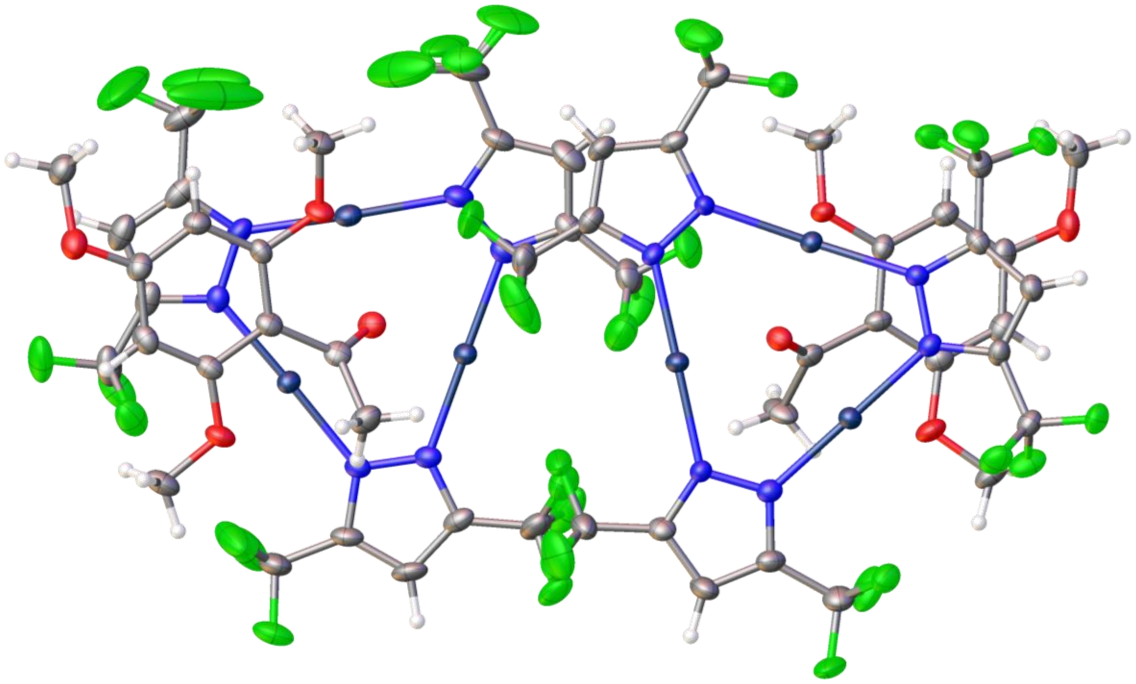

Figure S52. Asymmetric unit of **Ag_3Pz_3·12** (thermal displacement parameters at the 50% probability level).

Table S20. Crystal data and structure refinement for $\mathbf{Ag_3Pz_3 \cdot 12}$

Empirical formula	$C_{52}H_{34}Ag_6F_{36}N_{12}O_8$
Formula weight	2286.13
Temperature/K	100.00(10)
Crystal system	monoclinic
Space group	$P2_1/n$
a/Å	23.5373(2)
b/Å	8.78930(10)
c/Å	35.3778(3)
α/°	90
β/°	106.6760(10)
γ/°	90
Volume/Å^3	7011.02(12)
Z	4
ρ_{calc}g/cm^3	2.166
μ/mm^{-1}	14.625
F(000)	4400.0
Crystal size/mm^3	0.14 × 0.12 × 0.1
Radiation	Cu Kα (λ = 1.54184)
2θ range for data collection/°	5.216 to 133.198
Index ranges	$-28 \le h \le 27$, $-7 \le k \le 10$, $-41 \le l \le 42$
Reflections collected	36932
Independent reflections	12302 [R_{int} = 0.0528, R_{sigma} = 0.0625]
Data/restraints/parameters	12302/15/1063
Goodness-of-fit on F^2	1.057
Final R indexes [I>=2σ (I)]	R_1 = 0.0451, wR_2 = 0.1112
Final R indexes [all data]	R_1 = 0.0708, wR_2 = 0.1176
Largest diff. peak/hole / e Å^{-3}	1.58/-1.02
CCDC-number	2501755

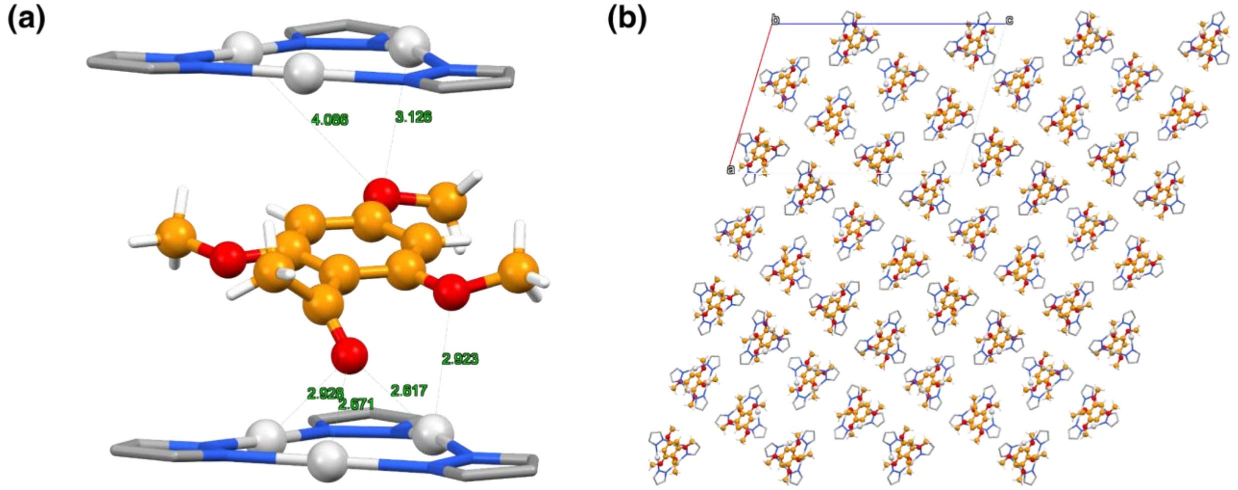

Figure S53. (a) A schematic diagram of the co-crystal structure in the $\mathbf{Ag_3Pz_3{\cdot}12}$ single crystal, formed by the guest organic molecule and the surrounding Ag_3Pz_3 units that exhibit significant interactions with it. (b) A 3 × 1 × 2 packing mode in the single crystal structure of $\mathbf{Ag_3Pz_3{\cdot}12}$ along the *b* axis. Trifluoromethyl groups and H atoms in Ag_3Pz_3 are omitted for clarity. Ag···O interactions are indicated with green dotted lines with distances in Å. C, N, and Ag atoms in Ag_3Pz_3 are depicted in dark gray, light blue, and light gray, respectively; C, O, and H atoms in **12** are depicted in orange, red, and white, respectively.

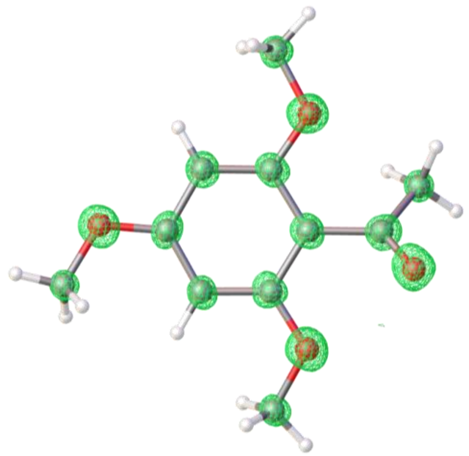

Figure S54. F_{obs} (contour: 0.45) electron density map superimposed on the structure of **12** in the single crystal structure of $\mathbf{Ag_3Pz_3{\cdot}12}$.

Preparation of Ag_3Pz_3·13. 2.08 mg (0.0107 mmol) of 1-(3-Ethoxy-4-methoxyphenyl)ethanone (**13**) was dissolved in 3 mL of a binary solvent system of DCM and n-Hex (1:1, v/v), followed by the addition of equimolar amounts of Ag_3Pz_3 (10.00 mg, 0.0107 mmol). The resulting mixed solution was filtered and then transferred to a 20 mL screw-capped sample vial. The cap of the sample vial was loosely closed to allow the solvent to slowly evaporate at room temperature. The entire co-crystal incubation process was protected from light using aluminum foil. After the designated evaporation period, typically 1-3 days, high-quality colorless block-shaped crystals suitable for single-crystal X-ray diffraction analysis formed at the bottom of the vial.

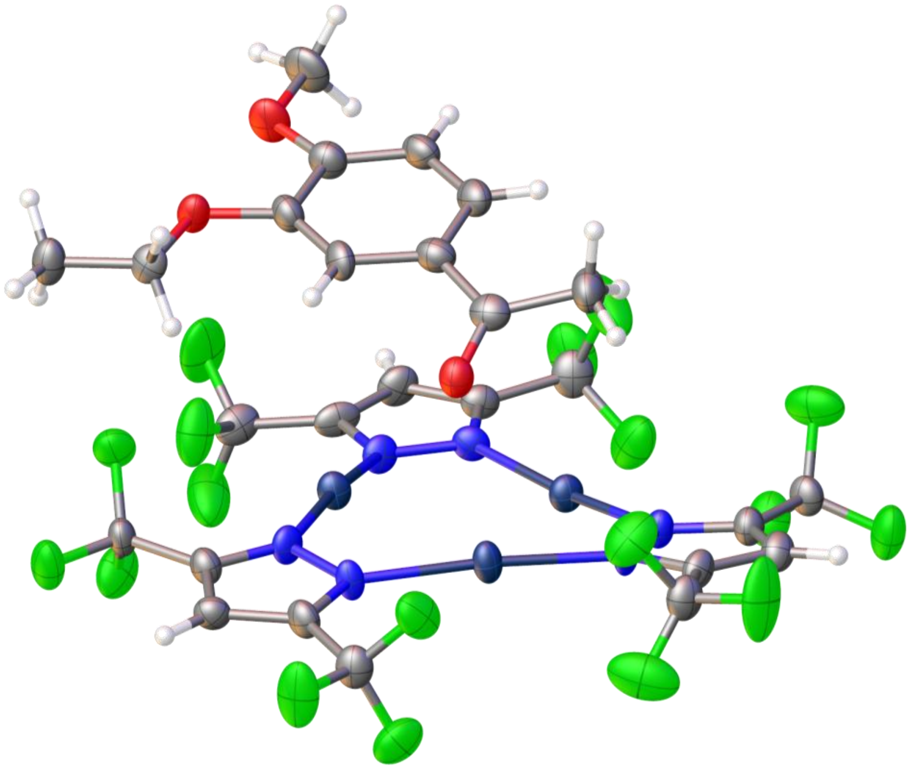

Figure S55. Asymmetric unit of **Ag_3Pz_3·13** (thermal displacement parameters at the 50% probability level).

Table S21. Crystal data and structure refinement for **Ag_3Pz_3·13**

Empirical formula	$C_{26}H_{17}Ag_3F_{18}N_6O_3$
Formula weight	1127.06
Temperature/K	100.01(16)
Crystal system	monoclinic
Space group	$P2_1/c$
a/Å	14.1818(5)
b/Å	13.6089(3)
c/Å	18.2517(6)
α/°	90
β/°	104.205(3)
γ/°	90
Volume/Å^3	3414.85(19)
Z	4
ρ_{calc}g/cm^3	2.192
μ/mm^{-1}	14.977
F(000)	2168.0
Crystal size/mm^3	0.2 × 0.2 × 0.1
Radiation	Cu Kα (λ = 1.54184)
2θ range for data collection/°	6.43 to 133.11
Index ranges	$-16 \le h \le 16$, $-16 \le k \le 16$, $-21 \le l \le 21$
Reflections collected	15943
Independent reflections	6008 [R_{int} = 0.0490, R_{sigma} = 0.0501]
Data/restraints/parameters	6008/12/508
Goodness-of-fit on F^2	1.016
Final R indexes [I>=2σ (I)]	R_1 = 0.0673, wR_2 = 0.1728
Final R indexes [all data]	R_1 = 0.0757, wR_2 = 0.1804
Largest diff. peak/hole / e Å^{-3}	4.68/-1.75
CCDC-number	2501756

Responses to CheckCIF alerts for Ag_3Pz_3·13 crystal structure:

A-level alerts:

"Check Calcd Resid. Dens. 1.40Ang From O00E 4.94 eA-3"

This Alert is due to presence of residual density in the presence of heavy metal atom (Ag).

"Check Calcd Resid. Dens. 1.18Ang From C014 4.37 eA-3"

This Alert is due to presence of residual density in the presence of heavy metal atom (Ag).

"Check Calcd Resid. Dens. 1.78Ang From C014 3.75 eA-3"

This Alert is due to presence of residual density in the presence of heavy metal atom (Ag).

B-level alert:

"Check Calcd Positive Resid. Density on Ag02 1.94 eA-3"

This Alert is due to presence of residual density in the presence of heavy metal atom (Ag).

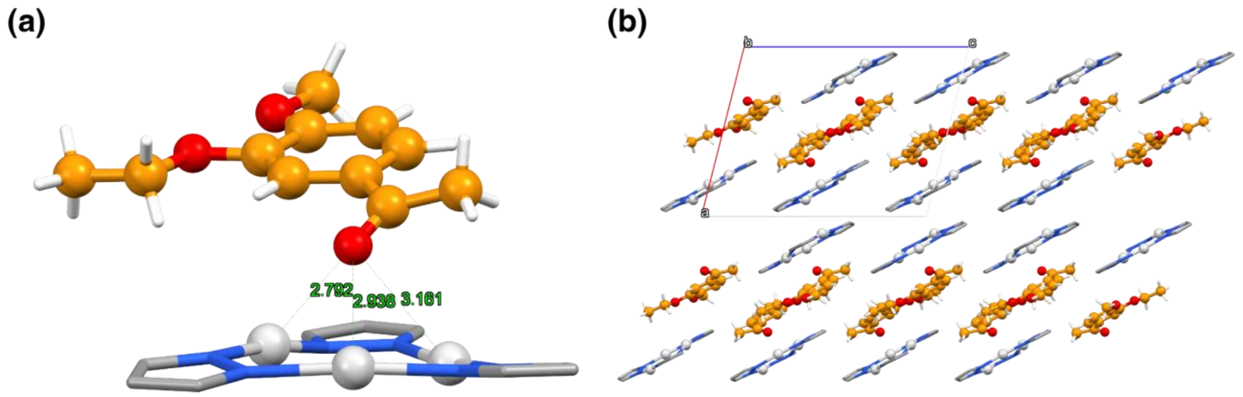

Figure S56. (a) A schematic diagram of the co-crystal structure in the $\mathbf{Ag_3Pz_3 \cdot 13}$ single crystal, formed by the guest organic molecule and the surrounding Ag_3Pz_3 units that exhibit significant interactions with it. (b) A 2 × 1 × 2 packing mode in the single crystal structure of $\mathbf{Ag_3Pz_3 \cdot 13}$ along the *b* axis. Trifluoromethyl groups and H atoms in Ag_3Pz_3 are omitted for clarity. Ag···O interactions are indicated with green dotted lines with distances in Å. C, N, and Ag atoms in Ag_3Pz_3 are depicted in dark gray, light blue, and light gray, respectively; C, O, and H atoms in **13** are depicted in orange, red, and white, respectively.

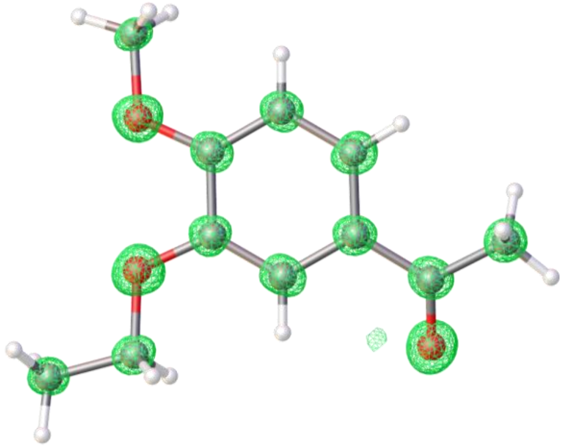

Figure S57. F_{obs} (contour: 0.45) electron density map superimposed on the structure of **13** in the single crystal structure of $\mathbf{Ag_3Pz_3 \cdot 13}$.

Preparation of Ag_3Pz_3·14. 1.46 mg (0.0107 mmol) of 3'-hydroxyacetophenone (**14**) was dissolved in 3 mL of a binary solvent system of DCM and n-Hex (1:1, v/v), followed by the addition of equimolar amounts of Ag_3Pz_3 (10.00 mg, 0.0107 mmol). The resulting mixed solution was filtered and then transferred to a 20 mL screw-capped sample vial. The cap of the sample vial was loosely closed to allow the solvent to slowly evaporate at room temperature. The entire co-crystal incubation process was protected from light using aluminum foil. After the designated evaporation period, typically 1-3 days, high-quality colorless needle-shaped crystals suitable for single-crystal X-ray diffraction analysis formed at the bottom of the vial.

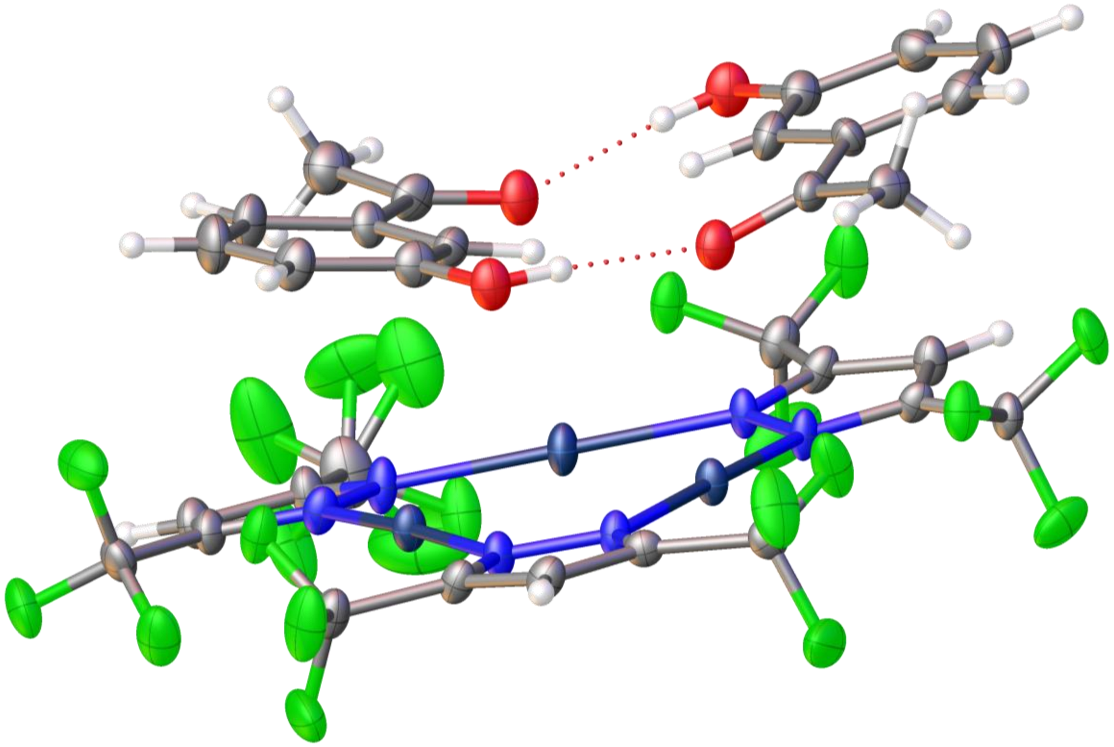

Figure S58. Asymmetric unit of **Ag_3Pz_3·14** (thermal displacement parameters at the 50% probability level).

Table S22. Crystal data and structure refinement for **Ag_3Pz_3·14**

Empirical formula	$C_{31}H_{19}Ag_3F_{18}N_6O_4$
Formula weight	1205.13
Temperature/K	99.98(13)
Crystal system	monoclinic
Space group	$P2_1/c$
a/Å	8.48770(10)
b/Å	40.1219(4)
c/Å	11.04370(10)
α/°	90
β/°	96.5430(10)
γ/°	90
Volume/Å^3	3736.35(7)
Z	4
ρ_{calc}g/cm^3	2.142
μ/mm^{-1}	13.769
F(000)	2328.0
Crystal size/mm^3	0.2 × 0.1 × 0.1
Radiation	Cu Kα (λ = 1.54184)
2θ range for data collection/°	8.354 to 156.802
Index ranges	$-9 \le h \le 10$, $-48 \le k \le 51$, $-12 \le l \le 13$
Reflections collected	19744
Independent reflections	7710 [R_{int} = 0.0408, R_{sigma} = 0.0437]
Data/restraints/parameters	7710/63/591
Goodness-of-fit on F^2	1.070
Final R indexes [I>=2σ (I)]	R_1 = 0.0514, wR_2 = 0.1358
Final R indexes [all data]	R_1 = 0.0613, wR_2 = 0.1403
Largest diff. peak/hole / e Å^{-3}	1.41/-1.12
CCDC-number	2501757

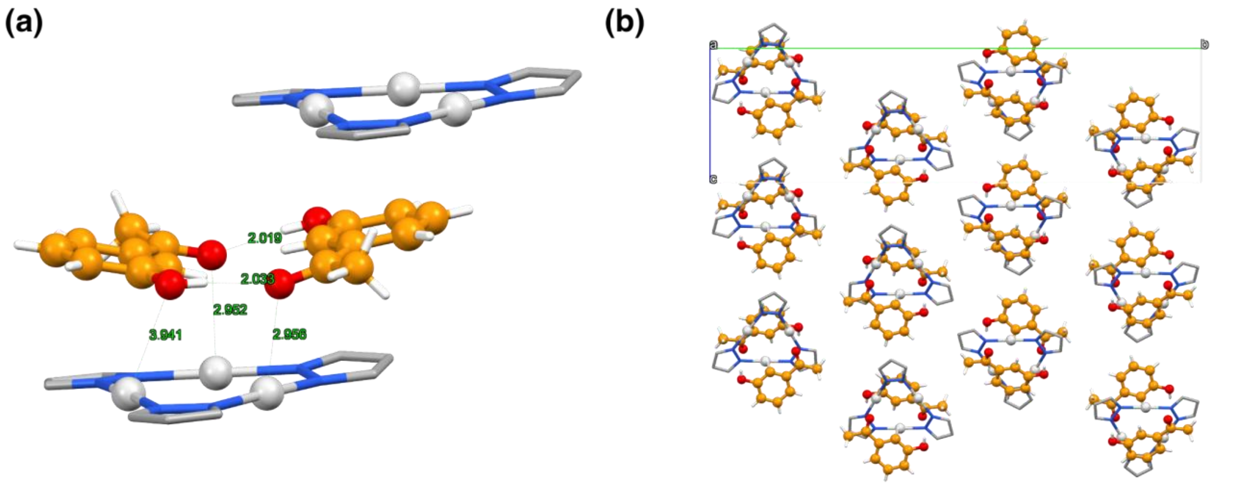

Figure S59. (a) A schematic diagram of the co-crystal structure in the **Ag_3Pz_3·14** single crystal, formed by the guest organic molecule and the surrounding Ag_3Pz_3 units that exhibit significant interactions with it. (b) A 1 × 1 × 3 packing mode in the single crystal structure of **Ag_3Pz_3·14** along the *a* axis. Trifluoromethyl groups and H atoms in Ag_3Pz_3 are omitted for clarity. Ag···O and O-H···O interactions are indicated with green dotted lines with distances in Å. C, N, and Ag atoms in Ag_3Pz_3 are depicted in dark gray, light blue, and light gray, respectively; C, O, and H atoms in **14** are depicted in orange, red, and white, respectively.

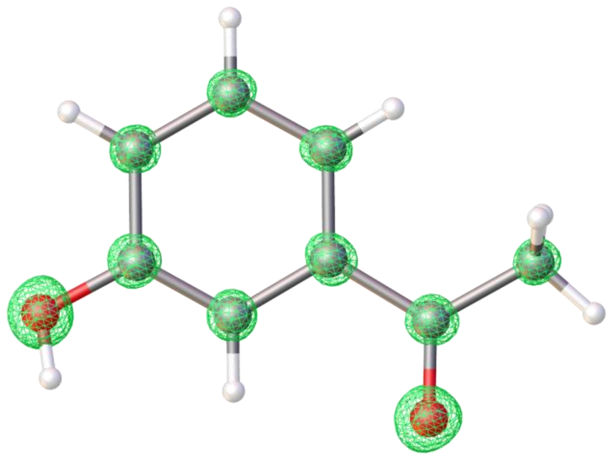

Figure S60. F_{obs} (contour: 1.00) electron density map superimposed on the structure of **14** in the single crystal structure of **Ag_3Pz_3·14**.

Preparation of Ag_3Pz_3·15. 1.46 mg (0.0107 mmol) of 4'-hydroxyacetophenone (**15**) was dissolved in 3 mL of a binary solvent system of DCM and n-Hex (1:1, v/v), followed by the addition of equimolar amounts of Ag_3Pz_3 (10.00 mg, 0.0107 mmol). The resulting mixed solution was filtered and then transferred to a 20 mL screw-capped sample vial. The cap of the sample vial was loosely closed to allow the solvent to slowly evaporate at room temperature. The entire co-crystal incubation process was protected from light using aluminum foil. After the designated evaporation period, typically 1-3 days, high-quality colorless plate-shaped crystals suitable for single-crystal X-ray diffraction analysis formed at the bottom of the vial.

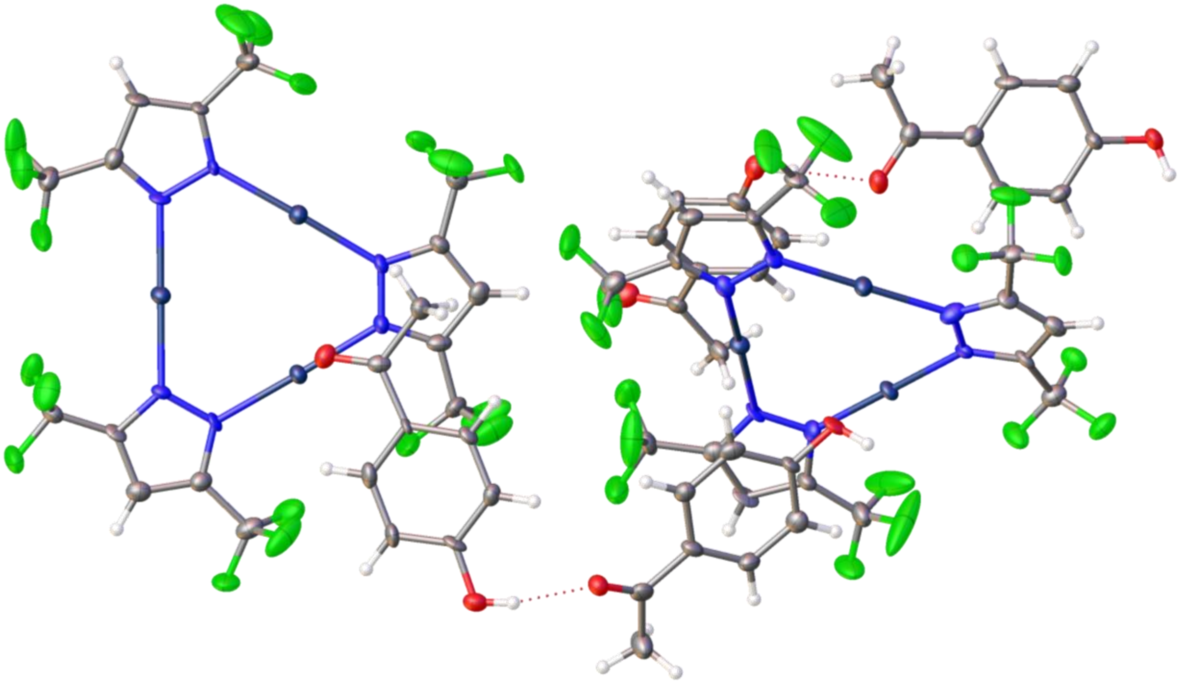

Figure S61. Asymmetric unit of **Ag_3Pz_3·15** (thermal displacement parameters at the 50% probability level).

Table S23. Crystal data and structure refinement for **Ag_3Pz_3·15**

Empirical formula	$C_{62}H_{38}Ag_6F_{36}N_{12}O_8$
Formula weight	2410.26
Temperature/K	100.01(18)
Crystal system	triclinic
Space group	$P\bar{1}$
a/Å	10.2262(2)
b/Å	17.2521(4)
c/Å	21.9165(5)
α/°	83.212(2)
β/°	84.130(2)
γ/°	87.082(2)
Volume/Å^3	3816.40(15)
Z	2
ρ_{calc}g/cm^3	2.097
μ/mm^{-1}	13.480
F(000)	2328.0
Crystal size/mm^3	0.15 × 0.14 × 0.12
Radiation	Cu Kα (λ = 1.54184)
2θ range for data collection/°	6.206 to 157.246
Index ranges	$-12 \leq h \leq 12$, $-20 \leq k \leq 21$, $-23 \leq l \leq 27$
Reflections collected	45701
Independent reflections	15759 [R_{int} = 0.1049, R_{sigma} = 0.1054]
Data/restraints/parameters	15759/94/1125
Goodness-of-fit on F^2	1.049
Final R indexes [I>=2σ (I)]	R_1 = 0.0919, wR_2 = 0.2062
Final R indexes [all data]	R_1 = 0.1241, wR_2 = 0.2175
Largest diff. peak/hole / e Å^{-3}	1.90/-1.54
CCDC-number	2501758

Responses to CheckCIF alert for Ag_3Pz_3·15 crystal structure:

(There is no A-level alert)

B-level alert:

"Coordinates do not Form a Properly Connected Set Please Do !"

The alert is due to a large number of co-crystallized molecules in the which sometimes do not show as a connected set. This is acceptable from a crystallographic point of view.

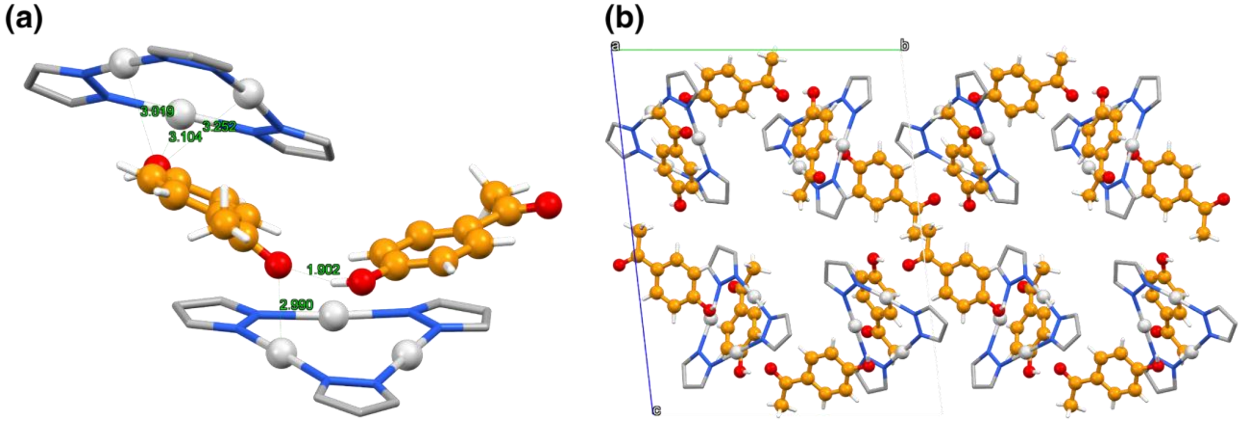

Figure S62. (a) A schematic diagram of the co-crystal structure in the $\mathbf{Ag_3Pz_3{\cdot}15}$ single crystal, formed by the guest organic molecule and the surrounding Ag_3Pz_3 units that exhibit significant interactions with it. (b) A 1 × 2 × 1 packing mode in the single crystal structure of $\mathbf{Ag_3Pz_3{\cdot}15}$ along the *a* axis. Trifluoromethyl groups and H atoms in Ag_3Pz_3 are omitted for clarity. Ag···O and O-H···O interactions are indicated with green dotted lines with distances in Å. C, N, and Ag atoms in Ag_3Pz_3 are depicted in dark gray, light blue, and light gray, respectively; C, O, and H atoms in **15** are depicted in orange, red, and white, respectively.

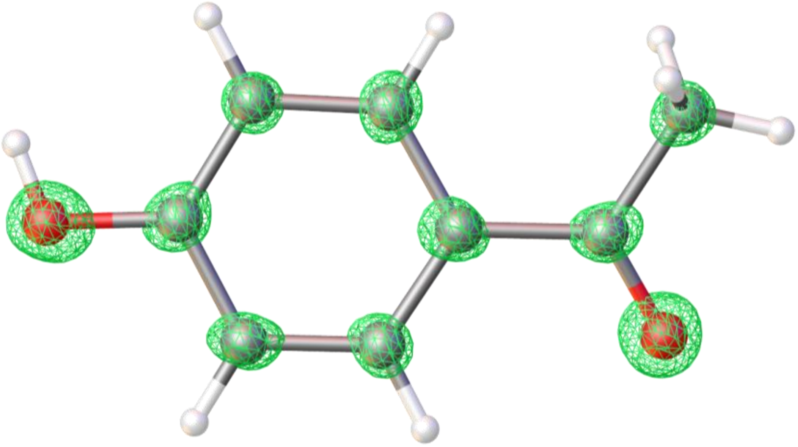

Figure S63. F_{obs} (contour: 1.00) electron density map superimposed on the structure of **15** in the single crystal structure of $\mathbf{Ag_3Pz_3{\cdot}15}$.

Preparation of Ag_3Pz_3·16. 1.78 mg (0.0107 mmol) of paeonol (**16**) was dissolved in 3 mL of c-Hex, followed by the addition of equimolar amounts of Ag_3Pz_3 (10.00 mg, 0.0107 mmol). The resulting mixed solution was filtered and then transferred to a 20 mL screw-capped sample vial. The cap of the sample vial was loosely closed to allow the solvent to slowly evaporate at room temperature. The entire co-crystal incubation process was protected from light using aluminum foil. After the designated evaporation period, typically 1-3 days, high-quality colorless needle-shaped crystals suitable for single-crystal X-ray diffraction analysis formed at the bottom of the vial.

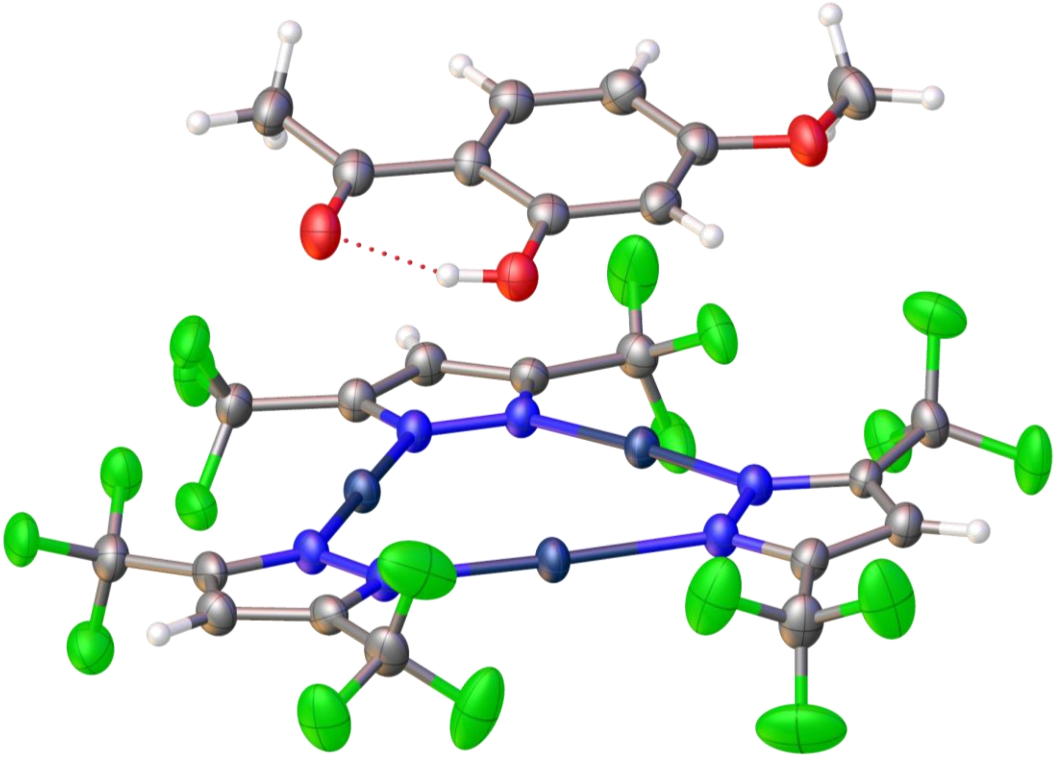

Figure S64. Asymmetric unit of **Ag_3Pz_3·16** (thermal displacement parameters at the 50% probability level).

Table S24. Crystal data and structure refinement for **Ag_3Pz_3·16**

Empirical formula	$C_{24}H_{13}Ag_3F_{18}N_6O_3$
Formula weight	1099.01
Temperature/K	99.99(18)
Crystal system	monoclinic
Space group	$P2_1/n$
a/Å	12.73460(10)
b/Å	11.28520(10)
c/Å	22.53920(10)
α/°	90
β/°	95.5450(10)
γ/°	90
Volume/Å^3	3224.01(4)
Z	4
ρ_{calc}g/cm^3	2.264
μ/mm^{-1}	15.841
F(000)	2104.0
Crystal size/mm^3	0.2 × 0.1 × 0.1
Radiation	Cu Kα (λ = 1.54184)
2θ range for data collection/°	8.338 to 155.952
Index ranges	$-15 \le h \le 16, -14 \le k \le 14, -27 \le l \le 28$
Reflections collected	20958
Independent reflections	6658 [R_{int} = 0.0295, R_{sigma} = 0.0295]
Data/restraints/parameters	6658/0/490
Goodness-of-fit on F^2	1.068
Final R indexes [I>=2σ (I)]	R_1 = 0.0364, wR_2 = 0.1011
Final R indexes [all data]	R_1 = 0.0381, wR_2 = 0.1024
Largest diff. peak/hole / e Å^{-3}	1.37/-0.90
CCDC-number	2501759

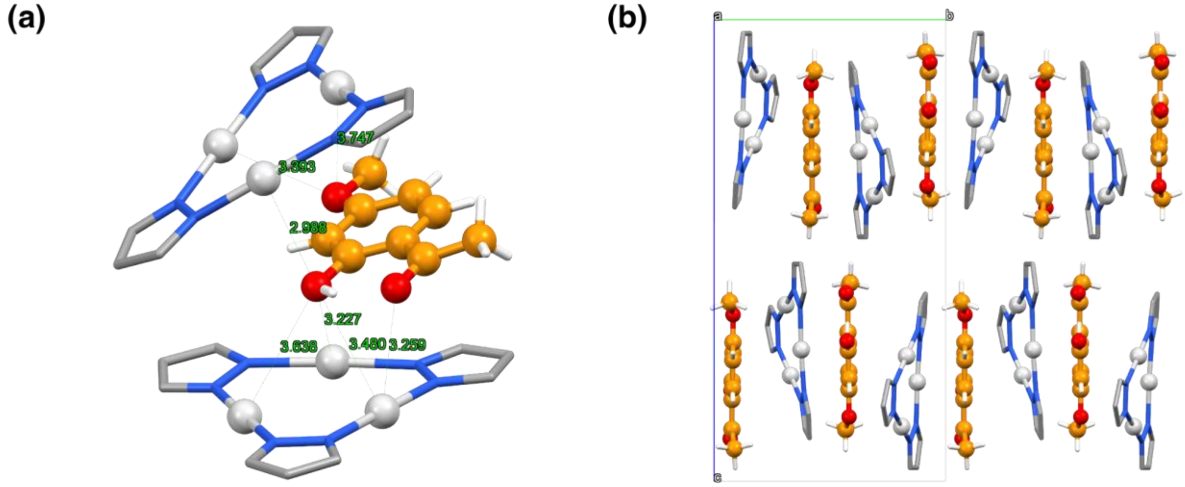

Figure S65. (a) A schematic diagram of the co-crystal structure in the **Ag_3Pz_3·16** single crystal, formed by the guest organic molecule and the surrounding Ag_3Pz_3 units that exhibit significant interactions with it. (b) A 1 × 2 × 1 packing mode in the single crystal structure of **Ag_3Pz_3·16** along the *a* axis. Trifluoromethyl groups and H atoms in Ag_3Pz_3 are omitted for clarity. Ag···O interactions are indicated with green dotted lines with distances in Å. C, N, and Ag atoms in Ag_3Pz_3 are depicted in dark gray, light blue, and light gray, respectively; C, O, and H atoms in **16** are depicted in orange, red, and white, respectively.

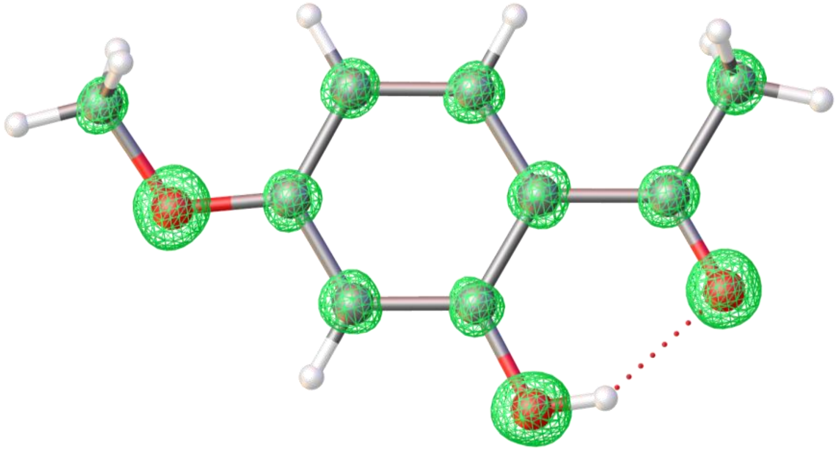

Figure S66. F_{obs} (contour: 0.75) electron density map superimposed on the structure of **16** in the single crystal structure of **Ag_3Pz_3·16**.

Preparation of Ag_3Pz_3·17. 1.78 mg (0.0107 mmol) of 3-hydroxy-4-methoxyacetophenone (**17**) was dissolved in 3 mL of a binary solvent system of DCM and n-Hex (1:1, v/v), followed by the addition of equimolar amounts of Ag_3Pz_3 (10.00 mg, 0.0107 mmol). The resulting mixed solution was filtered and then transferred to a 20 mL screw-capped sample vial. The cap of the sample vial was loosely closed to allow the solvent to slowly evaporate at room temperature. The entire co-crystal incubation process was protected from light using aluminum foil. After the designated evaporation period, typically 1-3 days, high-quality colorless needle-shaped crystals suitable for single-crystal X-ray diffraction analysis formed at the bottom of the vial.

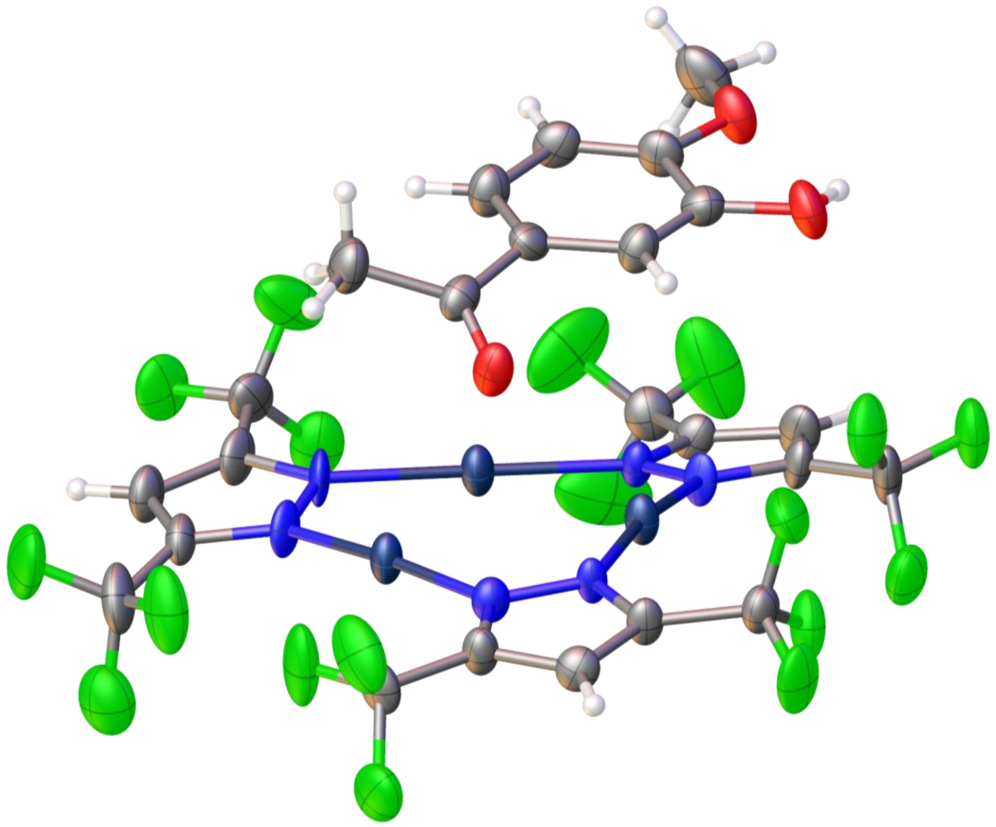

Figure S67. Asymmetric unit of **Ag_3Pz_3·17** (thermal displacement parameters at the 50% probability level).

Table S25. Crystal data and structure refinement for **Ag_3Pz_3·17**

Empirical formula	$C_{24}H_{13}Ag_3F_{18}N_6O_3$
Formula weight	1099.01
Temperature/K	99.98(13)
Crystal system	trigonal
Space group	$P3_2$
a/Å	13.2788(3)
b/Å	13.2788(3)
c/Å	15.8408(8)
α/°	90
β/°	90
γ/°	120
Volume/Å^3	2418.94(16)
Z	3
ρ_{calc}g/cm^3	2.263
μ/mm^{-1}	15.835
F(000)	1578.0
Crystal size/mm^3	0.16 × 0.15 × 0.14
Radiation	Cu Kα (λ = 1.54184)
2θ range for data collection/°	7.688 to 156.268
Index ranges	$-16 \le h \le 16$, $-16 \le k \le 16$, $-11 \le l \le 19$
Reflections collected	9522
Independent reflections	4596 [R_{int} = 0.0481, R_{sigma} = 0.0551]
Data/restraints/parameters	4596/33/478
Goodness-of-fit on F^2	1.100
Final R indexes [I>=2σ (I)]	R_1 = 0.0518, wR_2 = 0.1333
Final R indexes [all data]	R_1 = 0.0574, wR_2 = 0.1361
Largest diff. peak/hole / e Å^{-3}	1.10/-0.95
Flack parameter	-0.012(17)
CCDC-number	2501760

Responses to CheckCIF alert for Ag_3Pz_3·17 crystal structure:

(There is no A-level alert)

B-level alert:

"No Flack x Check Done: Low Friedel Pair Coverage 37 %"

Due to insufficient data. The compound crystallizes in the chiral space group, but no chiral ligand is used, and the complex is no longer chiral.

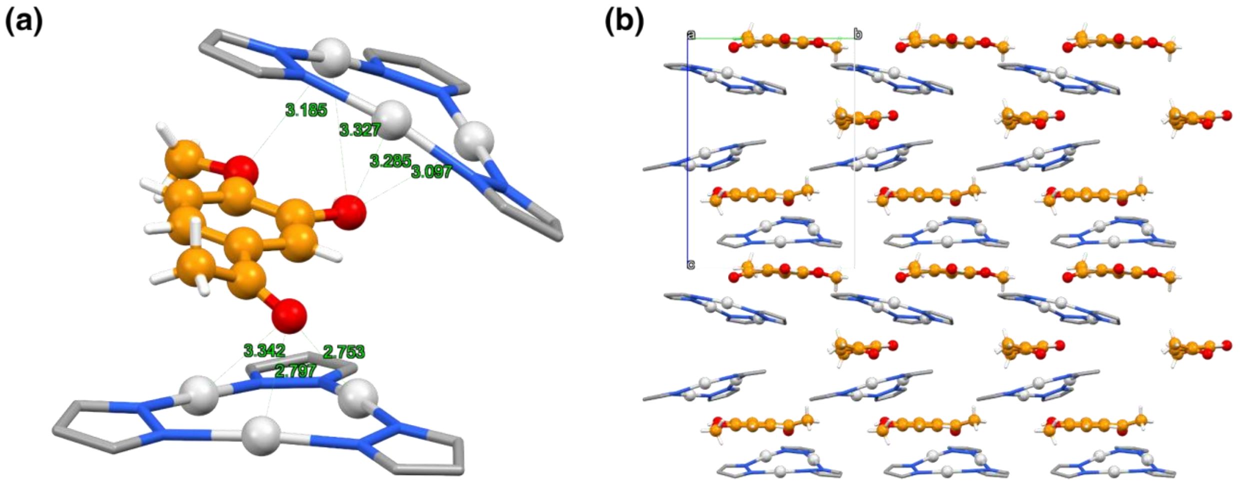

Figure S68. (a) A schematic diagram of the co-crystal structure in the **Ag_3Pz_3·17** single crystal, formed by the guest organic molecule and the surrounding Ag_3Pz_3 units that exhibit significant interactions with it. (b) A 1 × 3 × 2 packing mode in the single crystal structure of **Ag_3Pz_3·17** along the *a* axis. Trifluoromethyl groups and H atoms in Ag_3Pz_3 are omitted for clarity. Ag···O interactions are indicated with green dotted lines with distances in Å. C, N, and Ag atoms in Ag_3Pz_3 are depicted in dark gray, light blue, and light gray, respectively; C, O, and H atoms in **17** are depicted in orange, red, and white, respectively.

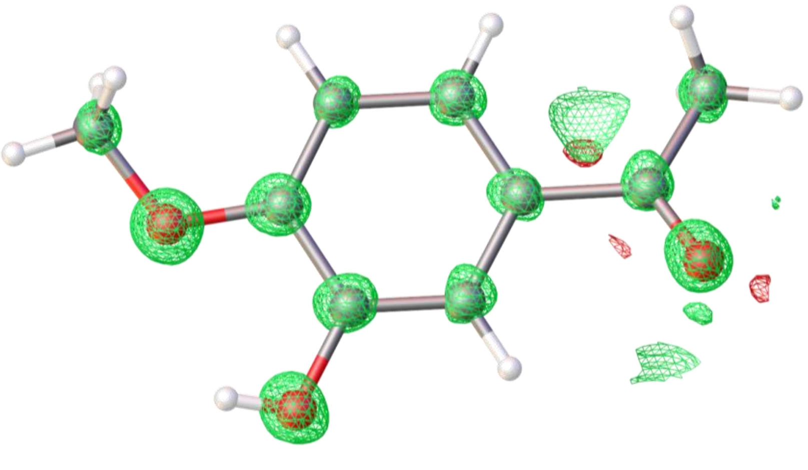

Figure S69. F_{obs} (contour: 0.95) electron density map superimposed on the structure of **17** in the single crystal structure of **Ag_3Pz_3·17**. We believe that the unassigned electron density is attributable to residual solvent molecules and the Ag_3Pz_3 units.

Preparation of Ag_3Pz_3·18. 1.78 mg (0.0107 mmol) of acetovanillone (**18**) was dissolved in 3 mL of a binary solvent system of DCM and c-Hex (1:1, v/v), followed by the addition of equimolar amounts of Ag_3Pz_3 (10.00 mg, 0.0107 mmol). The resulting mixed solution was filtered and then transferred to a 20 mL screw-capped sample vial. The cap of the sample vial was loosely closed to allow the solvent to slowly evaporate at room temperature. The entire co-crystal incubation process was protected from light using aluminum foil. After the designated evaporation period, typically 1-3 days, high-quality colorless block-shaped crystals suitable for single-crystal X-ray diffraction analysis formed at the bottom of the vial.

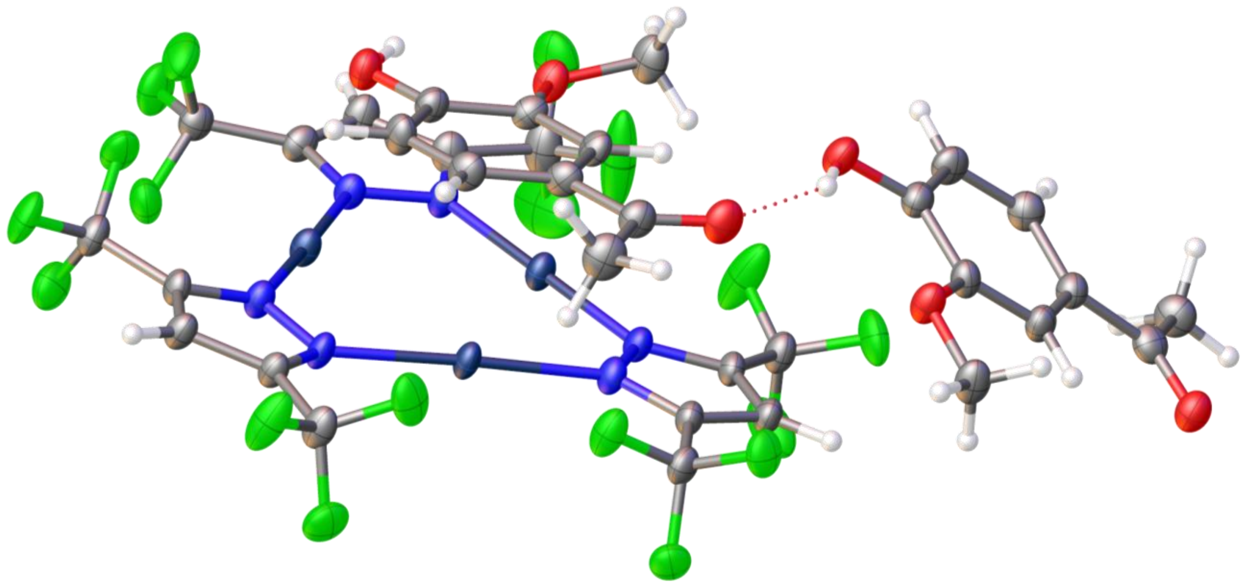

Figure S70. Asymmetric unit of **Ag_3Pz_3·18** (thermal displacement parameters at the 50% probability level).

Table S26. Crystal data and structure refinement for **Ag_3Pz_3·18**

Empirical formula	$C_{33}H_{23}Ag_3F_{18}N_6O_6$
Formula weight	1265.18
Temperature/K	101(1)
Crystal system	monoclinic
Space group	$P2_1/n$
a/Å	12.80340(10)
b/Å	14.59220(10)
c/Å	22.2955(2)
α/°	90
β/°	106.2490(10)
γ/°	90
Volume/Å^3	3999.07(6)
Z	4
ρ_{calc}g/cm^3	2.101
μ/mm^{-1}	12.944
F(000)	2456.0
Crystal size/mm^3	0.26 × 0.16 × 0.15
Radiation	Cu Kα (λ = 1.54184)
2θ range for data collection/°	7.222 to 156.756
Index ranges	$-13 \leq h \leq 16$, $-18 \leq k \leq 17$, $-28 \leq l \leq 28$
Reflections collected	20125
Independent reflections	8166 [R_{int} = 0.0251, R_{sigma} = 0.0262]
Data/restraints/parameters	8166/0/601
Goodness-of-fit on F^2	1.125
Final R indexes [I>=2σ (I)]	R_1 = 0.0427, wR_2 = 0.1255
Final R indexes [all data]	R_1 = 0.0445, wR_2 = 0.1266
Largest diff. peak/hole / e Å^{-3}	1.10/-1.73
CCDC-number	2501761

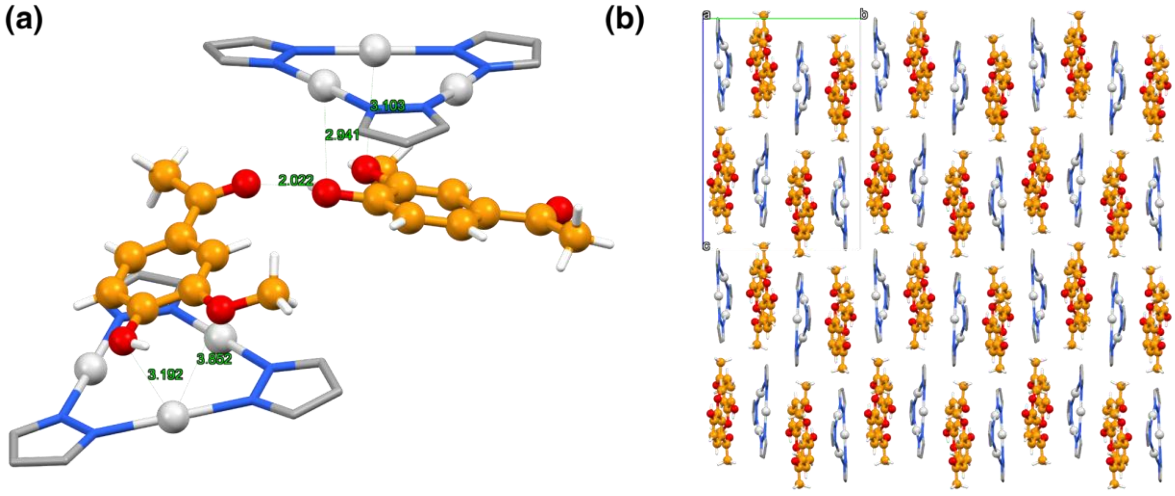

Figure S71. (a) A schematic diagram of the co-crystal structure in the $\mathbf{Ag_3Pz_3{\cdot}18}$ single crystal, formed by the guest organic molecule and the surrounding Ag_3Pz_3 units that exhibit significant interactions with it. (b) A $1 \times 3 \times 2$ packing mode in the single crystal structure of $\mathbf{Ag_3Pz_3{\cdot}18}$ along the *a* axis. Trifluoromethyl groups and H atoms in Ag_3Pz_3 are omitted for clarity. Ag···O and O-H···O interactions are indicated with green dotted lines with distances in Å. C, N, and Ag atoms in Ag_3Pz_3 are depicted in dark gray, light blue, and light gray, respectively; C, O, and H atoms in **18** are depicted in orange, red, and white, respectively.

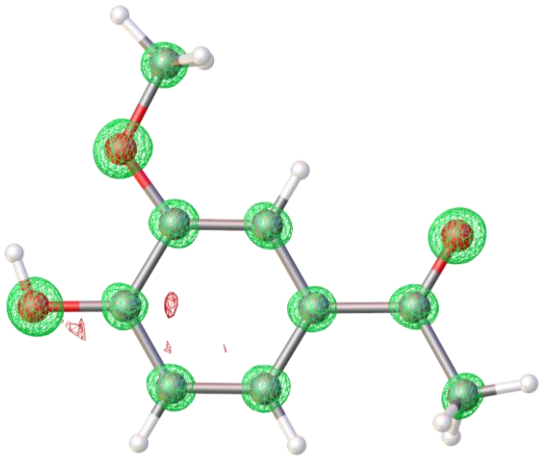

Figure S72. F_{obs} (contour: 1.20) electron density map superimposed on the structure of **18** in the single crystal structure of $\mathbf{Ag_3Pz_3{\cdot}18}$.

Preparation of Ag_3Pz_3·19. 1.93 mg (0.0107 mmol) of 3,4-dimethoxyacetophenone (**19**) was dissolved in 3 mL of c-Hex, followed by the addition of equimolar amounts of Ag_3Pz_3 (10.00 mg, 0.0107 mmol). The resulting mixed solution was filtered and then transferred to a 20 mL screw-capped sample vial. The cap of the sample vial was loosely closed to allow the solvent to slowly evaporate at room temperature. The entire co-crystal incubation process was protected from light using aluminum foil. After the designated evaporation period, typically 1-3 days, high-quality colorless needle-shaped crystals suitable for single-crystal X-ray diffraction analysis formed at the bottom of the vial.

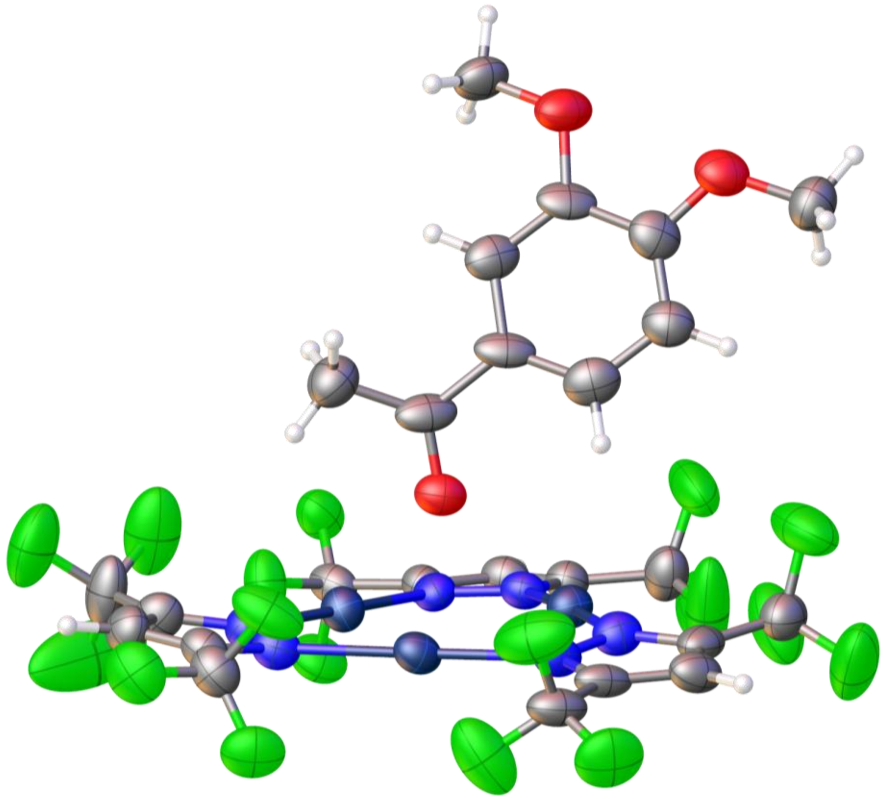

Figure S73. Asymmetric unit of **Ag_3Pz_3·19** (thermal displacement parameters at the 50% probability level).

Table S27. Crystal data and structure refinement for $\mathbf{Ag_3Pz_3 \cdot 19}$

Empirical formula	$C_{25}H_{15}Ag_3F_{18}N_6O_3$
Formula weight	1113.04
Temperature/K	100.00(15)
Crystal system	orthorhombic
Space group	*Pbca*
a/Å	16.7884(3)
b/Å	16.1378(3)
c/Å	25.0667(4)
α/°	90
β/°	90
γ/°	90
Volume/Å^3	6791.3(2)
Z	8
ρ_{calc}g/cm^3	2.177
μ/mm^{-1}	15.051
F(000)	4272.0
Crystal size/mm^3	0.2 × 0.1 × 0.1
Radiation	Cu Kα (λ = 1.54184)
2θ range for data collection/°	7.052 to 156.986
Index ranges	$-21 \leq h \leq 19$, $-10 \leq k \leq 19$, $-31 \leq l \leq 31$
Reflections collected	27068
Independent reflections	7032 [R_{int} = 0.0479, R_{sigma} = 0.0340]
Data/restraints/parameters	7032/0/499
Goodness-of-fit on F^2	1.151
Final R indexes [I>=2σ (I)]	R_1 = 0.0756, wR_2 = 0.1861
Final R indexes [all data]	R_1 = 0.0920, wR_2 = 0.1956
Largest diff. peak/hole / e Å^{-3}	2.37/-1.02
CCDC-number	2501762

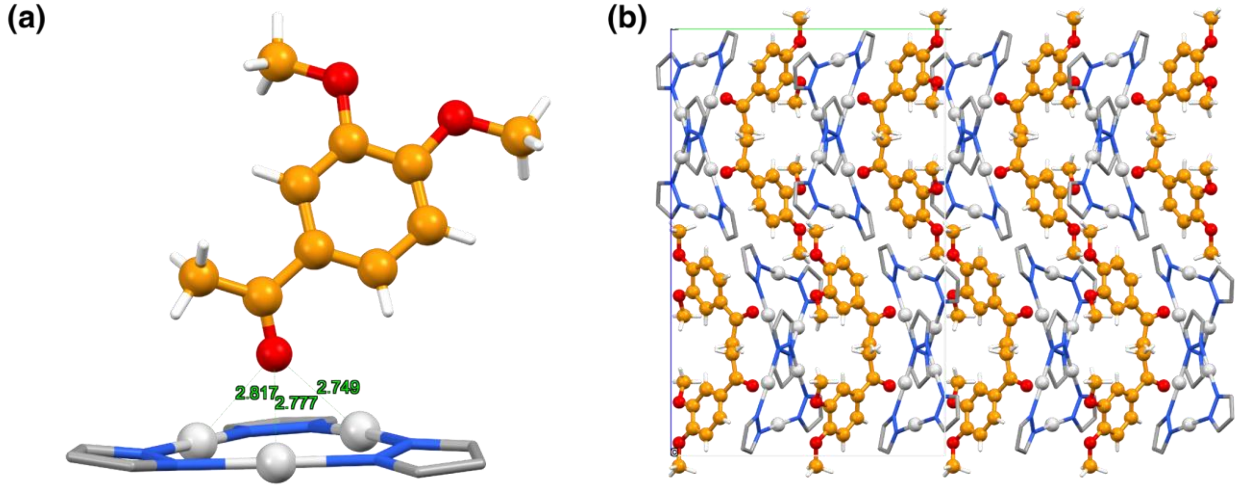

Figure S74. (a) A schematic diagram of the co-crystal structure in the **Ag_3Pz_3·19** single crystal, formed by the guest organic molecule and the surrounding Ag_3Pz_3 units that exhibit significant interactions with it. (b) A 1 × 2 × 1 packing mode in the single crystal structure of **Ag_3Pz_3·19** along the *a* axis. Trifluoromethyl groups and H atoms in Ag_3Pz_3 are omitted for clarity. Ag···O interactions are indicated with green dotted lines with distances in Å. C, N, and Ag atoms in Ag_3Pz_3 are depicted in dark gray, light blue, and light gray, respectively; C, O, and H atoms in **19** are depicted in orange, red, and white, respectively.

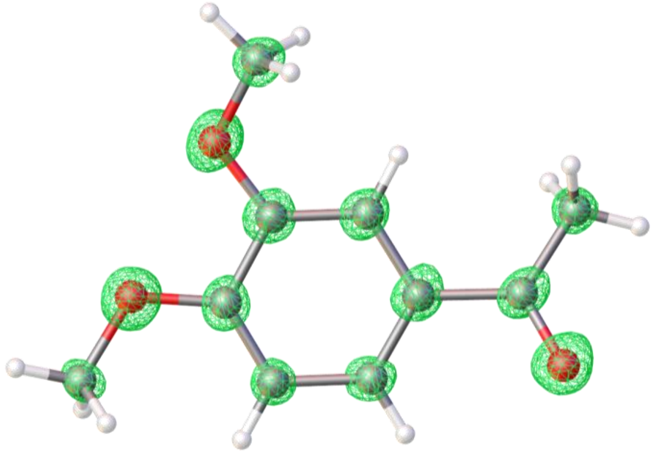

Figure S75. F_{obs} (contour: 0.45) electron density map superimposed on the structure of **19** in the single crystal structure of **Ag_3Pz_3·19**.

Preparation of Ag_3Pz_3·20. 1.93 mg (0.0107 mmol) of 3,5-dimethoxyacetophenone (**20**) was dissolved in 3 mL of n-Hex, followed by the addition of equimolar amounts of Ag_3Pz_3 (10.00 mg, 0.0107 mmol). The resulting mixed solution was filtered and then transferred to a 20 mL screw-capped sample vial. The cap of the sample vial was loosely closed to allow the solvent to slowly evaporate at room temperature. The entire co-crystal incubation process was protected from light using aluminum foil. After the designated evaporation period, typically 1-3 days, high-quality colorless needle-shaped crystals suitable for single-crystal X-ray diffraction analysis formed at the bottom of the vial.

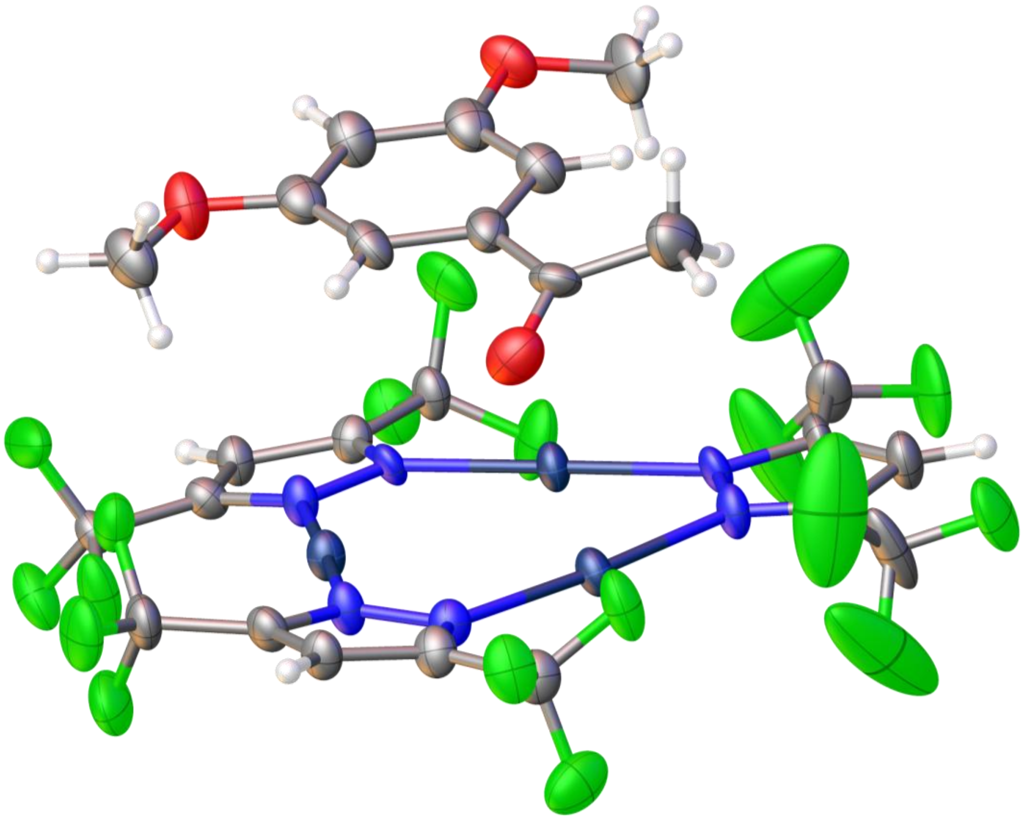

Figure S76. Asymmetric unit of **Ag_3Pz_3·20** (thermal displacement parameters at the 50% probability level).

Table S28. Crystal data and structure refinement for **Ag_3Pz_3·20**

Empirical formula	$C_{25}H_{15}Ag_3F_{18}N_6O_3$
Formula weight	1113.04
Temperature/K	99.98(16)
Crystal system	monoclinic
Space group	$P2_1/n$
a/Å	8.08780(10)
b/Å	16.6965(2)
c/Å	24.5862(3)
α/°	90
β/°	97.7990(10)
γ/°	90
Volume/Å^3	3289.36(7)
Z	4
ρ_{calc}g/cm^3	2.248
μ/mm^{-1}	15.538
F(000)	2136.0
Crystal size/mm^3	0.2 × 0.2 × 0.2
Radiation	Cu Kα (λ = 1.54184)
2θ range for data collection/°	6.418 to 155.78
Index ranges	$-8 \le h \le 9$, $-20 \le k \le 17$, $-29 \le l \le 31$
Reflections collected	16793
Independent reflections	6724 [R_{int} = 0.0648, R_{sigma} = 0.0633]
Data/restraints/parameters	6724/24/499
Goodness-of-fit on F^2	1.055
Final R indexes [I>=2σ (I)]	R_1 = 0.0684, wR_2 = 0.1785
Final R indexes [all data]	R_1 = 0.0794, wR_2 = 0.1853
Largest diff. peak/hole / e Å^{-3}	2.07/-1.34
CCDC-number	2501764

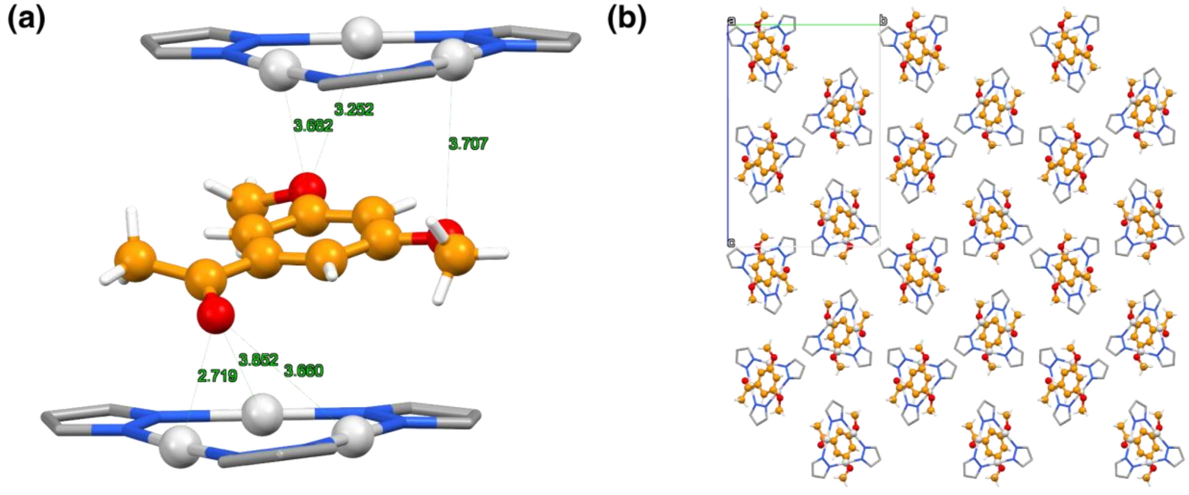

Figure S77. (a) A schematic diagram of the co-crystal structure in the **Ag_3Pz_3·20** single crystal, formed by the guest organic molecule and the surrounding Ag_3Pz_3 units that exhibit significant interactions with it. (b) A 1 × 3 × 2 packing mode in the single crystal structure of **Ag_3Pz_3·20** along the *a* axis. Trifluoromethyl groups and H atoms in Ag_3Pz_3 are omitted for clarity. Ag···O interactions are indicated with green dotted lines with distances in Å. C, N, and Ag atoms in Ag_3Pz_3 are depicted in dark gray, light blue, and light gray, respectively; C, O, and H atoms in **20** are depicted in orange, red, and white, respectively.

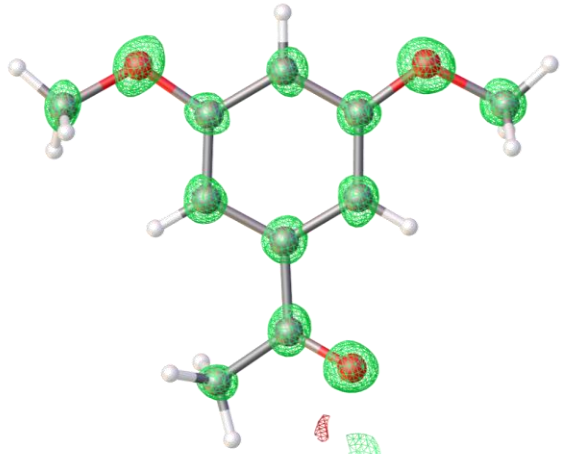

Figure S78. F_{obs} (contour: 0.75) electron density map superimposed on the structure of **20** in the single crystal structure of **Ag_3Pz_3·20**.

Preparation of Ag_3Pz_3·21. 1.93 mg (0.0107 mmol) of 2,4-dimethoxyacetophenone (**21**) was dissolved in 3 mL of n-Hex, followed by the addition of equimolar amounts of Ag_3Pz_3 (10.00 mg, 0.0107 mmol). The resulting mixed solution was filtered and then transferred to a 20 mL screw-capped sample vial. The cap of the sample vial was loosely closed to allow the solvent to slowly evaporate at room temperature. The entire co-crystal incubation process was protected from light using aluminum foil. After the designated evaporation period, typically 1-3 days, high-quality colorless needle-shaped crystals suitable for single-crystal X-ray diffraction analysis formed at the bottom of the vial.

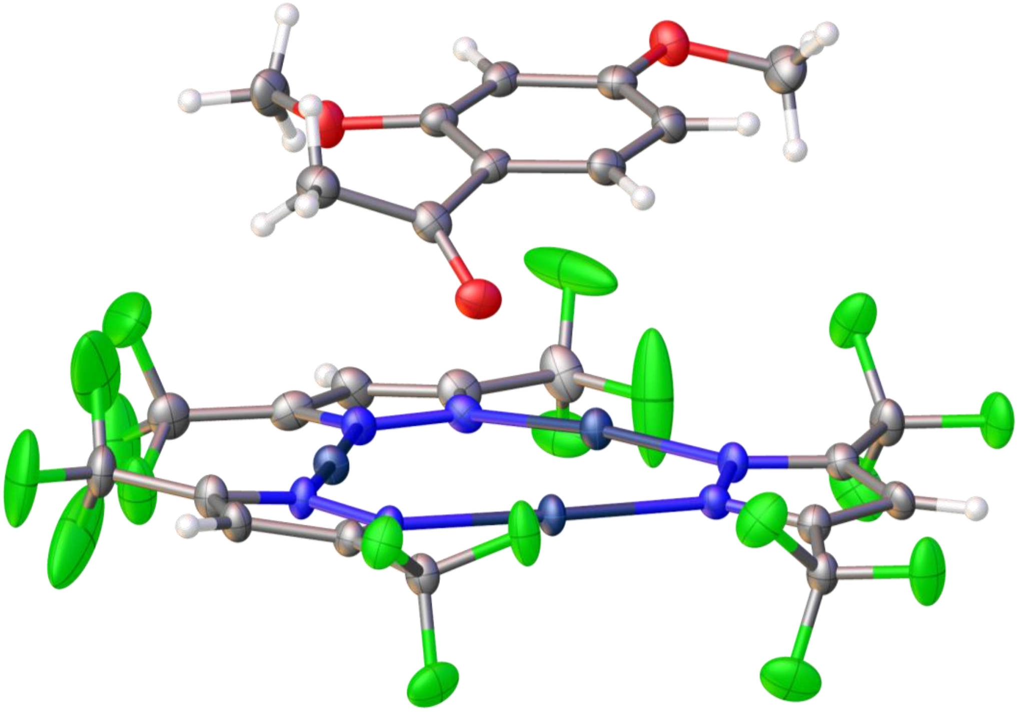

Figure S79. Asymmetric unit of **Ag_3Pz_3·21** (thermal displacement parameters at the 50% probability level).

Table S29. Crystal data and structure refinement for **Ag_3Pz_3·21**

Empirical formula	$C_{25}H_{15}Ag_3F_{18}N_6O_3$
Formula weight	1113.04
Temperature/K	100.03(15)
Crystal system	triclinic
Space group	$P\bar{1}$
a/Å	8.4178(2)
b/Å	12.5046(2)
c/Å	16.8597(3)
α/°	102.357(2)
β/°	101.656(2)
γ/°	102.699(2)
Volume/Å^3	1632.36(6)
Z	2
ρ_{calc}g/cm^3	2.265
μ/mm^{-1}	15.655
F(000)	1068.0
Crystal size/mm^3	0.3 × 0.2 × 0.1
Radiation	Cu Kα (λ = 1.54184)
2θ range for data collection/°	5.56 to 156.668
Index ranges	$-5 \le h \le 10$, $-15 \le k \le 15$, $-19 \le l \le 21$
Reflections collected	13356
Independent reflections	6608 [R_{int} = 0.0257, R_{sigma} = 0.0268]
Data/restraints/parameters	6608/24/499
Goodness-of-fit on F^2	1.068
Final R indexes [I>=2σ (I)]	R_1 = 0.0410, wR_2 = 0.1145
Final R indexes [all data]	R_1 = 0.0428, wR_2 = 0.1159
Largest diff. peak/hole / e Å^{-3}	1.41/-1.34
CCDC-number	2501765

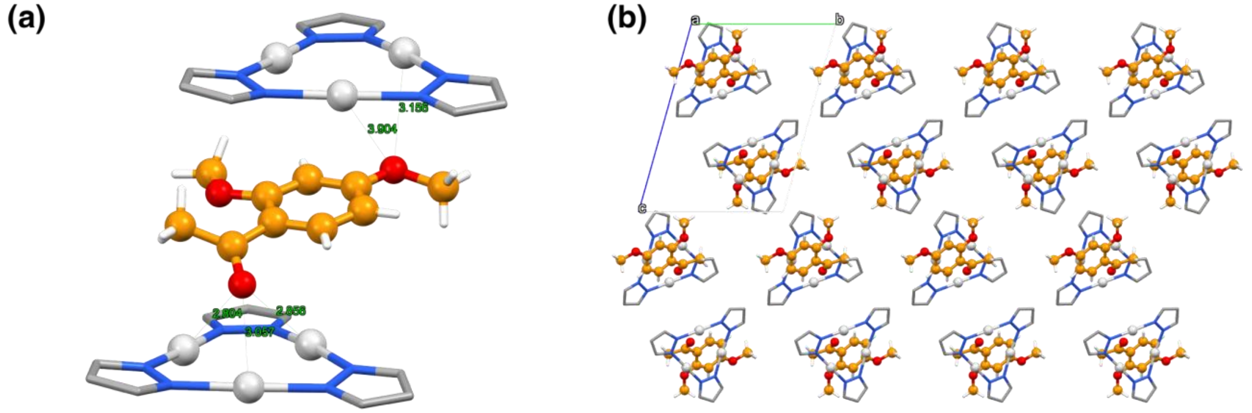

Figure S80. (a) A schematic diagram of the co-crystal structure in the **Ag_3Pz_3·21** single crystal, formed by the guest organic molecule and the surrounding Ag_3Pz_3 units that exhibit significant interactions with it. (b) A 1 × 4 × 2 packing mode in the single crystal structure of **Ag_3Pz_3·21** along the *a* axis. Trifluoromethyl groups and H atoms in Ag_3Pz_3 are omitted for clarity. Ag···O interactions are indicated with green dotted lines with distances in Å. C, N, and Ag atoms in Ag_3Pz_3 are depicted in dark gray, light blue, and light gray, respectively; C, O, and H atoms in **21** are depicted in orange, red, and white, respectively.

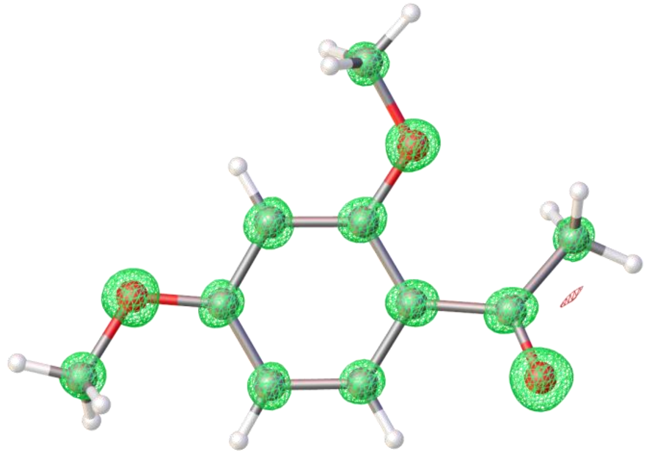

Figure S81. F_{obs} (contour: 1.90) electron density map superimposed on the structure of **21** in the single crystal structure of **Ag_3Pz_3·21**.

Preparation of Ag_3Pz_3·22. 1.74 mg (0.0107 mmol) of 1,4-diacetylbenzene (**22**) was dissolved in 3 mL of a binary solvent system of DCM and n-Hex (1:1, v/v), followed by the addition of equimolar amounts of Ag_3Pz_3 (10.00 mg, 0.0107 mmol). The resulting mixed solution was filtered and then transferred to a 20 mL screw-capped sample vial. The cap of the sample vial was loosely closed to allow the solvent to slowly evaporate at room temperature. The entire co-crystal incubation process was protected from light using aluminum foil. After the designated evaporation period, typically 1-3 days, high-quality colorless needle-shaped crystals suitable for single-crystal X-ray diffraction analysis formed at the bottom of the vial.

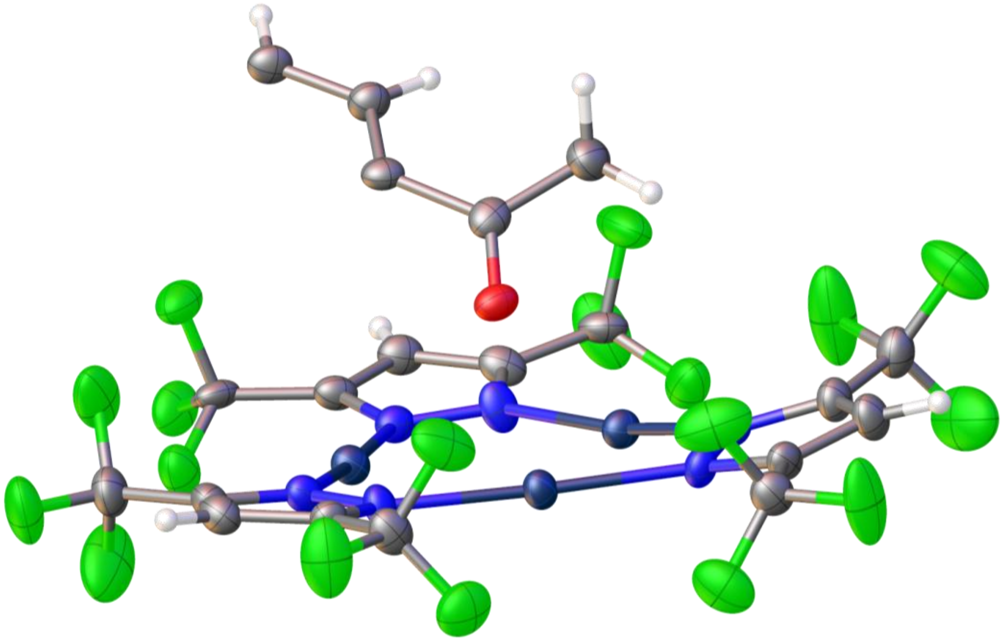

Figure S82. Asymmetric unit of **Ag_3Pz_3·22** (thermal displacement parameters at the 50% probability level).

Table S30. Crystal data and structure refinement for $\mathbf{Ag_3Pz_3 \cdot 22}$

Empirical formula	$C_{20}H_8Ag_3F_{18}N_6O$
Formula weight	1013.93
Temperature/K	100.01(17)
Crystal system	triclinic
Space group	$P\bar{1}$
a/Å	11.6764(3)
b/Å	12.0088(2)
c/Å	12.0565(3)
α/°	115.741(2)
β/°	103.986(2)
γ/°	101.171(2)
Volume/Å^3	1388.69(6)
Z	2
ρ_{calc}g/cm^3	2.425
μ/mm^{-1}	18.249
F(000)	962.0
Crystal size/mm^3	0.3 × 0.1 × 0.1
Radiation	Cu Kα (λ = 1.54184)
2θ range for data collection/°	8.304 to 156.84
Index ranges	$-14 \leq h \leq 14$, $-15 \leq k \leq 11$, $-14 \leq l \leq 15$
Reflections collected	11888
Independent reflections	5640 [R_{int} = 0.0488, R_{sigma} = 0.0531]
Data/restraints/parameters	5640/0/434
Goodness-of-fit on F^2	1.062
Final R indexes [I>=2σ (I)]	R_1 = 0.0575, wR_2 = 0.1568
Final R indexes [all data]	R_1 = 0.0666, wR_2 = 0.1611
Largest diff. peak/hole / e Å^{-3}	1.96/-1.42
CCDC-number	2501766

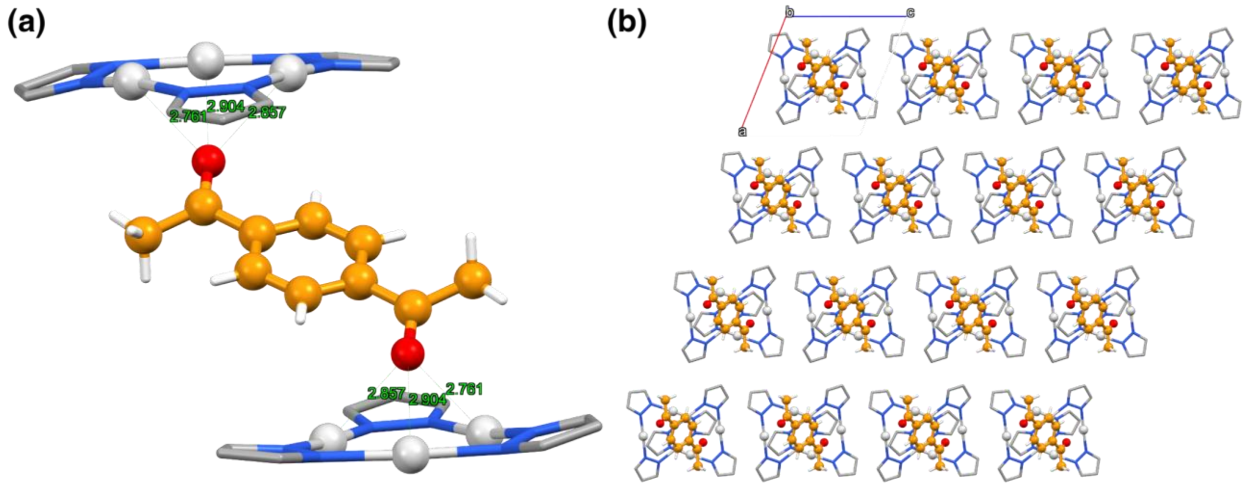

Figure S83. (a) A schematic diagram of the co-crystal structure in the $\mathbf{Ag_3Pz_3{\cdot}22}$ single crystal, formed by the guest organic molecule and the surrounding Ag_3Pz_3 units that exhibit significant interactions with it. (b) A 4 × 1 × 4 packing mode in the single crystal structure of $\mathbf{Ag_3Pz_3{\cdot}22}$ along the *b* axis. Trifluoromethyl groups and H atoms in Ag_3Pz_3 are omitted for clarity. Ag···O interactions are indicated with green dotted lines with distances in Å. C, N, and Ag atoms in Ag_3Pz_3 are depicted in dark gray, light blue, and light gray, respectively; C, O, and H atoms in **22** are depicted in orange, red, and white, respectively.

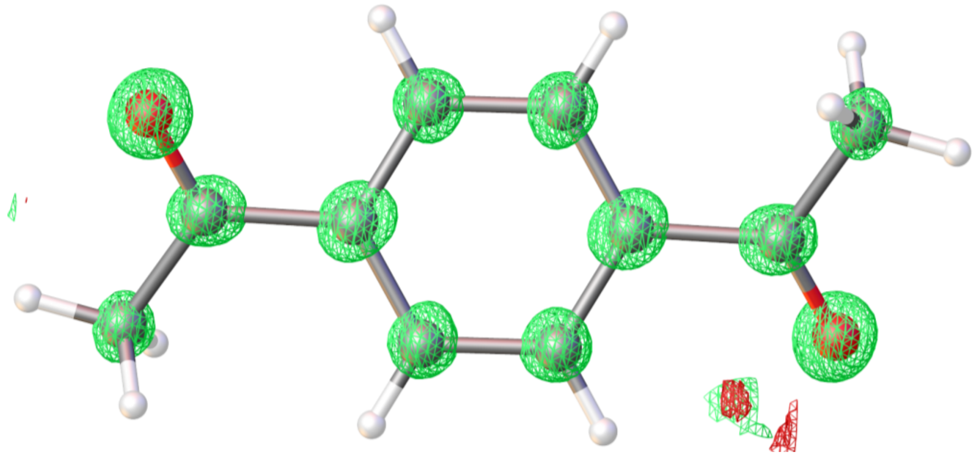

Figure S84. F_{obs} (contour: 1.25) electron density map superimposed on the structure of **22** in the single crystal structure of $\mathbf{Ag_3Pz_3{\cdot}22}$.

Preparation of Ag_3Pz_3·23. 2.14 mg (0.0107 mmol) of 1-(4-methoxy-1-naphthyl)ethanone (**23**) was dissolved in 3 mL of a binary solvent system of DCM and MeOH (1:1, v/v), followed by the addition of equimolar amounts of Ag_3Pz_3 (10.00 mg, 0.0107 mmol). The resulting mixed solution was filtered and then transferred to a 20 mL screw-capped sample vial. The cap of the sample vial was loosely closed to allow the solvent to slowly evaporate at room temperature. The entire co-crystal incubation process was protected from light using aluminum foil. After the designated evaporation period, typically 1-3 days, high-quality colorless needle-shaped crystals suitable for single-crystal X-ray diffraction analysis formed at the bottom of the vial.

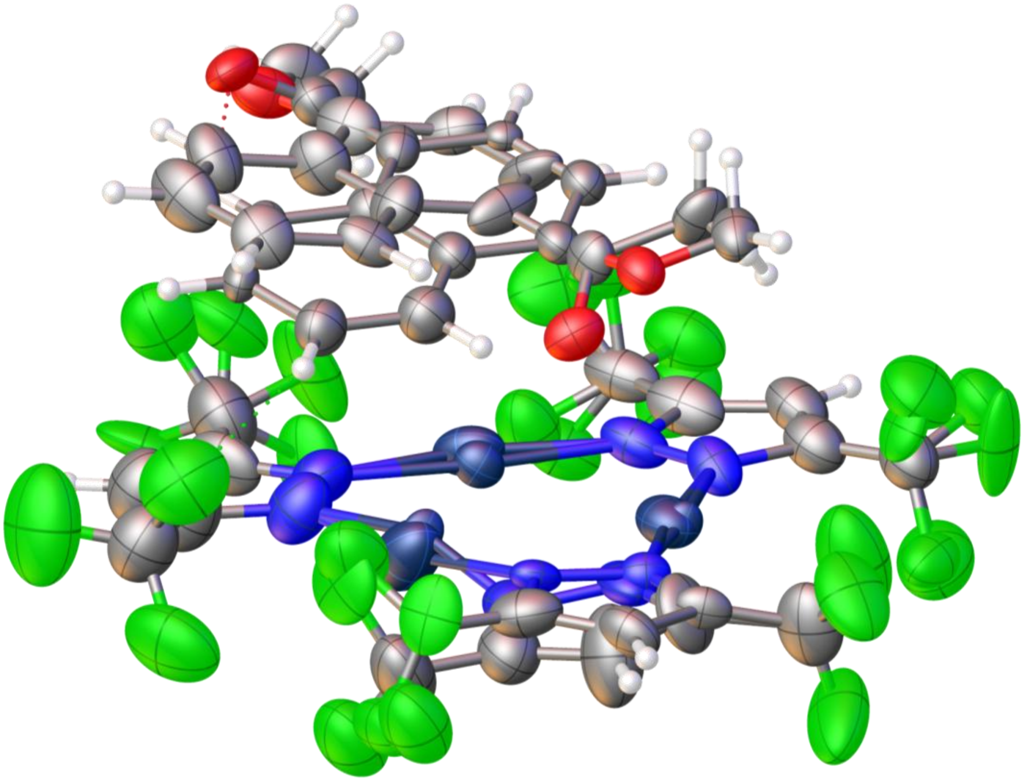

Figure S85. Asymmetric unit of **Ag_3Pz_3·23** (thermal displacement parameters at the 50% probability level).

Table S31. Crystal data and structure refinement for $\mathbf{Ag_3Pz_3{\cdot}23}$

Empirical formula	$C_{28}H_{15}Ag_3F_{18}N_6O_2$
Formula weight	1133.07
Temperature/K	100.15
Crystal system	monoclinic
Space group	$P2_1/n$
a/Å	8.3525(5)
b/Å	22.6623(11)
c/Å	17.9457(11)
α/°	90
β/°	96.182(5)
γ/°	90
Volume/Å^3	3377.1(3)
Z	4
ρ_{calc}g/cm^3	2.229
μ/mm^{-1}	15.130
F(000)	2176.0
Crystal size/mm^3	0.21 × 0.16 × 0.12
Radiation	Cu Kα (λ = 1.54184)
2θ range for data collection/°	7.802 to 145.66
Index ranges	$-10 \leq h \leq 9$, $-27 \leq k \leq 22$, $-21 \leq l \leq 21$
Reflections collected	24936
Independent reflections	6506 [R_{int} = 0.0568, R_{sigma} = 0.0522]
Data/restraints/parameters	6506/508/761
Goodness-of-fit on F^2	1.123
Final R indexes [I>=2σ (I)]	R_1 = 0.1054, wR_2 = 0.2715
Final R indexes [all data]	R_1 = 0.1400, wR_2 = 0.2927
Largest diff. peak/hole / e Å^{-3}	1.97/-1.17
CCDC-number	2501767

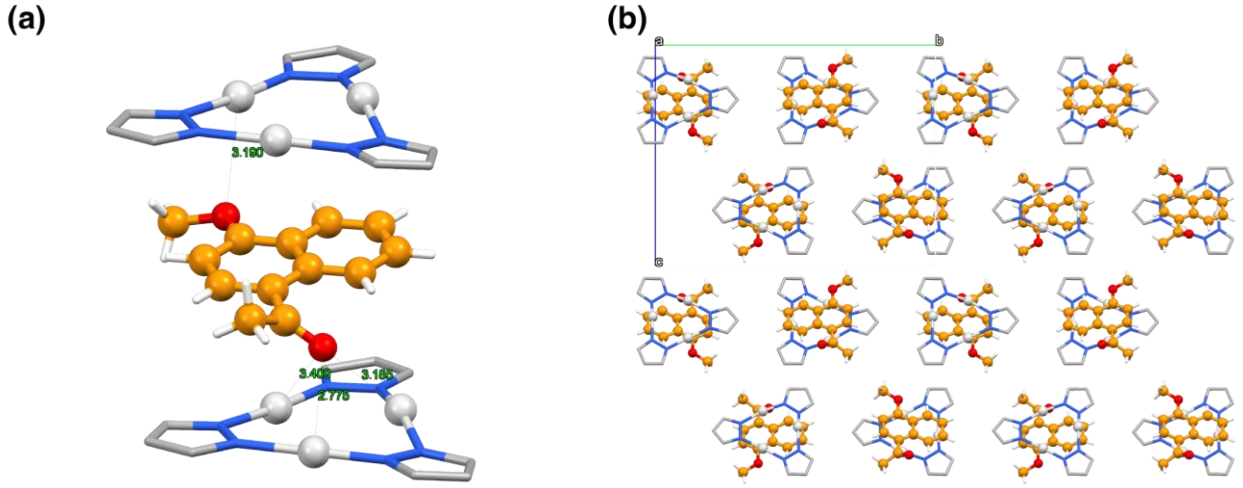

Figure S86. (a) A schematic diagram of the co-crystal structure in the **Ag_3Pz_3·23** single crystal, formed by the guest organic molecule and the surrounding Ag_3Pz_3 units that exhibit significant interactions with it. (b) A 1 × 2 × 2 packing mode in the single crystal structure of **Ag_3Pz_3·23** along the *a* axis. Trifluoromethyl groups and H atoms in Ag_3Pz_3 are omitted for clarity. Ag···O interactions are indicated with green dotted lines with distances in Å. C, N, and Ag atoms in Ag_3Pz_3 are depicted in dark gray, light blue, and light gray, respectively; C, O, and H atoms in **23** are depicted in orange, red, and white, respectively.

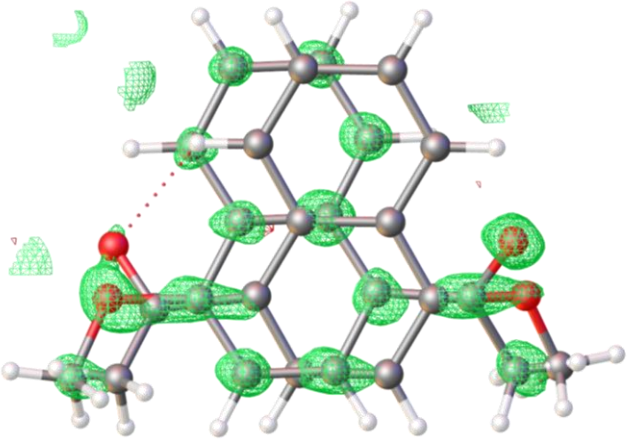

Figure S87. F_{obs} (contour: 0.25) electron density map superimposed on the structure of **23** in the single crystal structure of **Ag_3Pz_3·23**. Please note that there is a twofold disorder in the molecule **23**, and the unassigned electron density is attributable to the Ag_3Pz_3 units.

Preparation of Ag_3Pz_3·24. 1.59 mg (0.0107 mmol) of 4-chromanone (**24**) was dissolved in 3 mL of a binary solvent system of DCM and n-Hex (1:1, v/v), followed by the addition of equimolar amounts of Ag_3Pz_3 (10.00 mg, 0.0107 mmol). The resulting mixed solution was filtered and then transferred to a 20 mL screw-capped sample vial. The cap of the sample vial was loosely closed to allow the solvent to slowly evaporate at room temperature. The entire co-crystal incubation process was protected from light using aluminum foil. After the designated evaporation period, typically 1-3 days, high-quality colorless block-shaped crystals suitable for single-crystal X-ray diffraction analysis formed at the bottom of the vial.

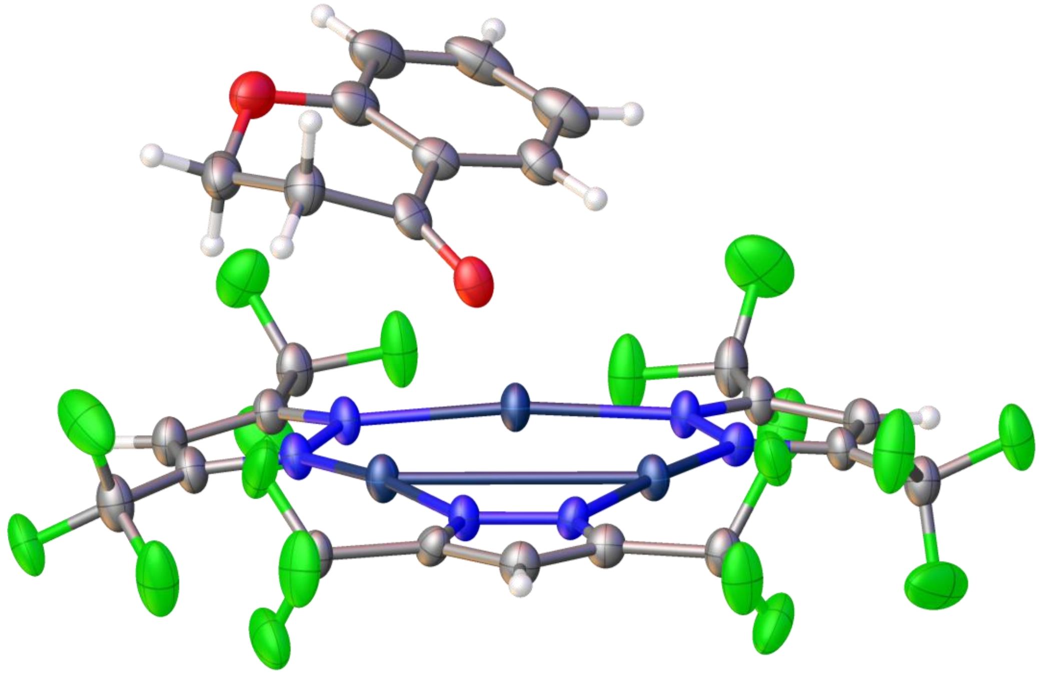

Figure S88. Asymmetric unit of **Ag_3Pz_3·24** (thermal displacement parameters at the 50% probability level).

Table S32. Crystal data and structure refinement for **Ag_3Pz_3·24**

Empirical formula	$C_{24}H_{11}Ag_3F_{18}N_6O_2$
Formula weight	1081.00
Temperature/K	100.01(19)
Crystal system	monoclinic
Space group	$P2_1/c$
a/Å	13.4249(2)
b/Å	13.20530(10)
c/Å	17.2600(2)
α/°	90
β/°	90.3530(10)
γ/°	90
Volume/Å^3	3059.79(6)
Z	4
ρ_{calc}g/cm^3	2.347
μ/mm^{-1}	16.651
F(000)	2064.0
Crystal size/mm^3	0.17 × 0.16 × 0.14
Radiation	Cu Kα (λ = 1.54184)
2θ range for data collection/°	6.584 to 156.102
Index ranges	$-16 \le h \le 16$, $-16 \le k \le 16$, $-21 \le l \le 21$
Reflections collected	18246
Independent reflections	6141 [R_{int} = 0.0389, R_{sigma} = 0.0383]
Data/restraints/parameters	6141/0/478
Goodness-of-fit on F^2	1.073
Final R indexes [I>=2σ (I)]	R_1 = 0.0423, wR_2 = 0.1145
Final R indexes [all data]	R_1 = 0.0470, wR_2 = 0.1172
Largest diff. peak/hole / e Å^{-3}	1.24/-1.26
CCDC-number	2501768

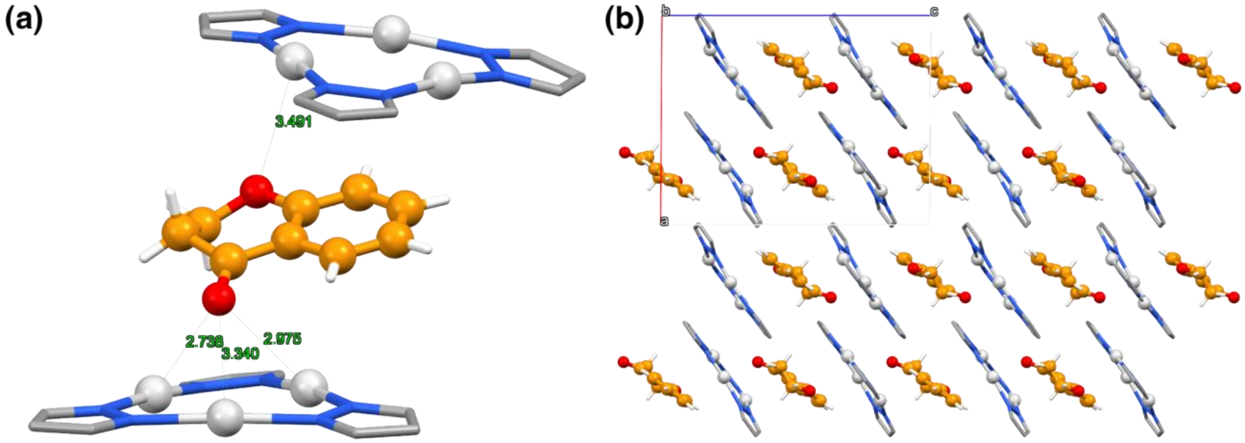

Figure S89. (a) A schematic diagram of the co-crystal structure in the **Ag_3Pz_3·24** single crystal, formed by the guest organic molecule and the surrounding Ag_3Pz_3 units that exhibit significant interactions with it. (b) A 2 × 1 × 2 packing mode in the single crystal structure of **Ag_3Pz_3·24** along the *b* axis. Trifluoromethyl groups and H atoms in Ag_3Pz_3 are omitted for clarity. Ag···O interactions are indicated with green dotted lines with distances in Å. C, N, and Ag atoms in Ag_3Pz_3 are depicted in dark gray, light blue, and light gray, respectively; C, O, and H atoms in **24** are depicted in orange, red, and white, respectively.

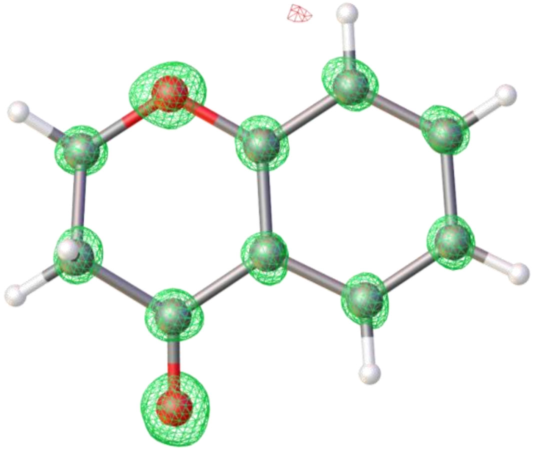

Figure S90. F_{obs} (contour: 0.89) electron density map superimposed on the structure of **24** in the single crystal structure of **Ag_3Pz_3·24**.

Preparation of Ag_3Pz_3·25. 1.91 mg (0.0107 mmol) of 7-methoxy-4-chromanone (**25**) was dissolved in 3 mL of a binary solvent system of DCM and n-Hex (1:1, v/v), followed by the addition of equimolar amounts of Ag_3Pz_3 (10.00 mg, 0.0107 mmol). The resulting mixed solution was filtered and then transferred to a 20 mL screw-capped sample vial. The cap of the sample vial was loosely closed to allow the solvent to slowly evaporate at room temperature. The entire co-crystal incubation process was protected from light using aluminum foil. After the designated evaporation period, typically 1-3 days, high-quality colorless needle-shaped crystals suitable for single-crystal X-ray diffraction analysis formed at the bottom of the vial.

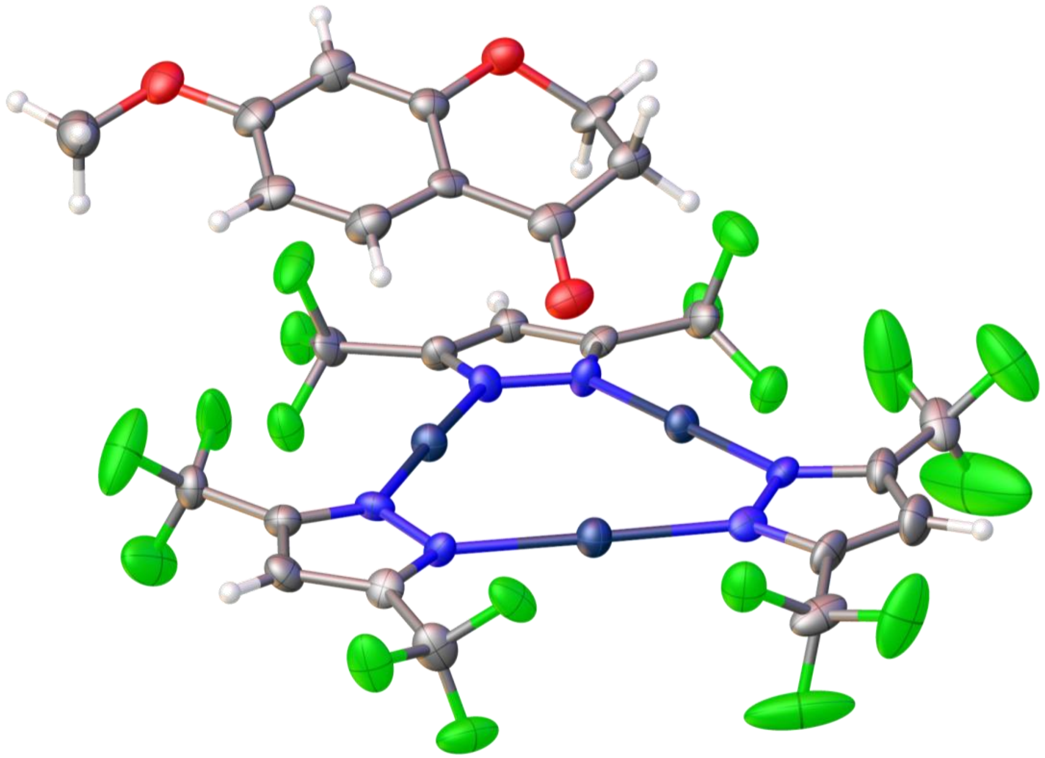

Figure S91. Asymmetric unit of **Ag_3Pz_3·25** (thermal displacement parameters at the 50% probability level).

Table S33. Crystal data and structure refinement for **Ag_3Pz_3·25**

Empirical formula	$C_{25}H_{13}Ag_3F_{18}N_6O_3$
Formula weight	1111.02
Temperature/K	100.0(2)
Crystal system	monoclinic
Space group	*Cc*
a/Å	12.54040(10)
b/Å	12.04020(10)
c/Å	21.4876(2)
α/°	90
β/°	94.2070(10)
γ/°	90
Volume/Å^3	3235.65(5)
Z	4
ρ_{calc}g/cm^3	2.281
μ/mm^{-1}	15.795
F(000)	2128.0
Crystal size/mm^3	0.23 × 0.16 × 0.14
Radiation	Cu Kα (λ = 1.54184)
2θ range for data collection/°	10.198 to 156.962
Index ranges	$-13 \leq h \leq 15$, $-11 \leq k \leq 15$, $-27 \leq l \leq 26$
Reflections collected	9609
Independent reflections	4851 [R_{int} = 0.0321, R_{sigma} = 0.0383]
Data/restraints/parameters	4851/14/498
Goodness-of-fit on F^2	1.093
Final R indexes [I>=2σ (I)]	R_1 = 0.0466, wR_2 = 0.1193
Final R indexes [all data]	R_1 = 0.0498, wR_2 = 0.1213
Largest diff. peak/hole / e Å^{-3}	1.24/-0.98
Flack parameter	-0.016(16)
CCDC-number	2501769

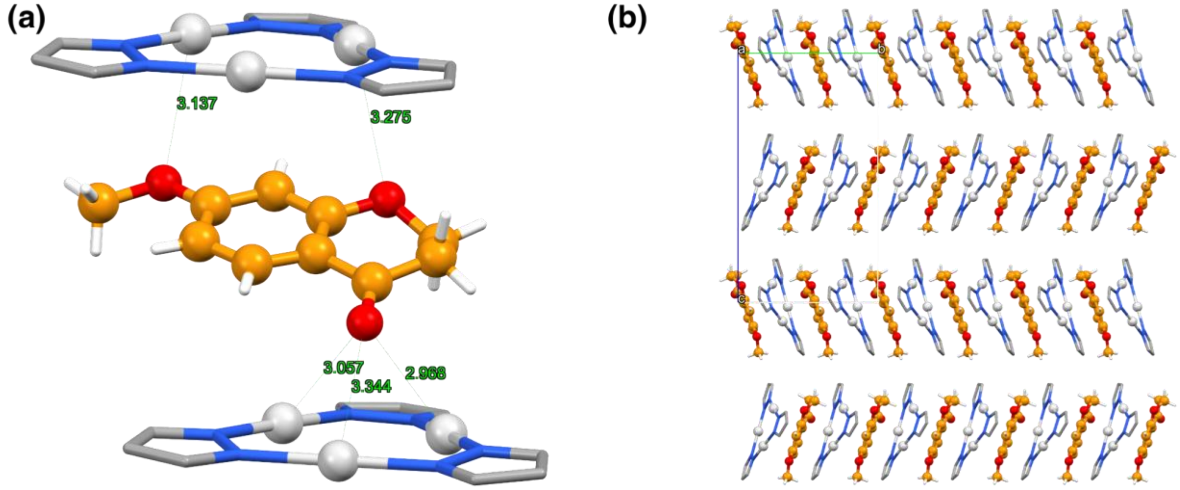

Figure S92. (a) A schematic diagram of the co-crystal structure in the **Ag_3Pz_3·25** single crystal, formed by the guest organic molecule and the surrounding Ag_3Pz_3 units that exhibit significant interactions with it. (b) A 1 × 3 × 2 packing mode in the single crystal structure of **Ag_3Pz_3·25** along the *a* axis. Trifluoromethyl groups and H atoms in Ag_3Pz_3 are omitted for clarity. Ag···O interactions are indicated with green dotted lines with distances in Å. C, N, and Ag atoms in Ag_3Pz_3 are depicted in dark gray, light blue, and light gray, respectively; C, O, and H atoms in **25** are depicted in orange, red, and white, respectively.

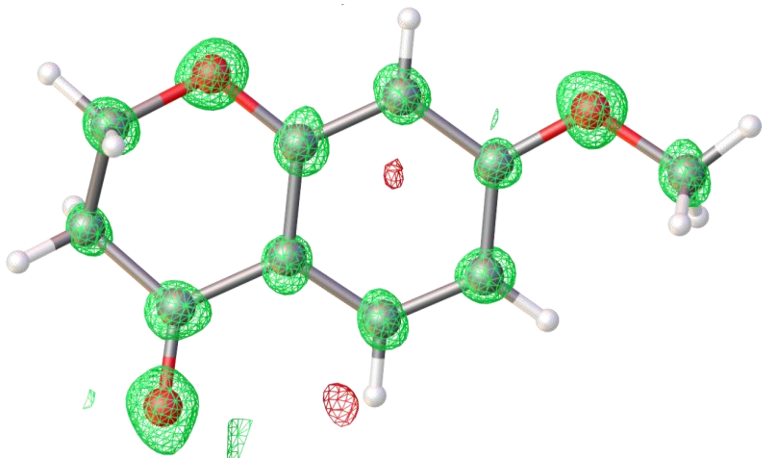

Figure S93. F_{obs} (contour: 0.72) electron density map superimposed on the structure of **25** in the single crystal structure of **Ag_3Pz_3·25**. We believe that the unassigned electron density is attributable to residual solvent molecules and the Ag_3Pz_3 units.

Preparation of Ag_3Pz_3·26. 1.89 mg (0.0107 mmol) of 5-methoxy-1-tetralone (**26**) was dissolved in 3 mL of DCM, followed by the addition of equimolar amounts of Ag_3Pz_3 (10.00 mg, 0.0107 mmol). The resulting mixed solution was filtered and then transferred to a 20 mL screw-capped sample vial. The cap of the sample vial was loosely closed to allow the solvent to slowly evaporate at room temperature. The entire co-crystal incubation process was protected from light using aluminum foil. After the designated evaporation period, typically 1-3 days, high-quality colorless needle-shaped crystals suitable for single-crystal X-ray diffraction analysis formed at the bottom of the vial.

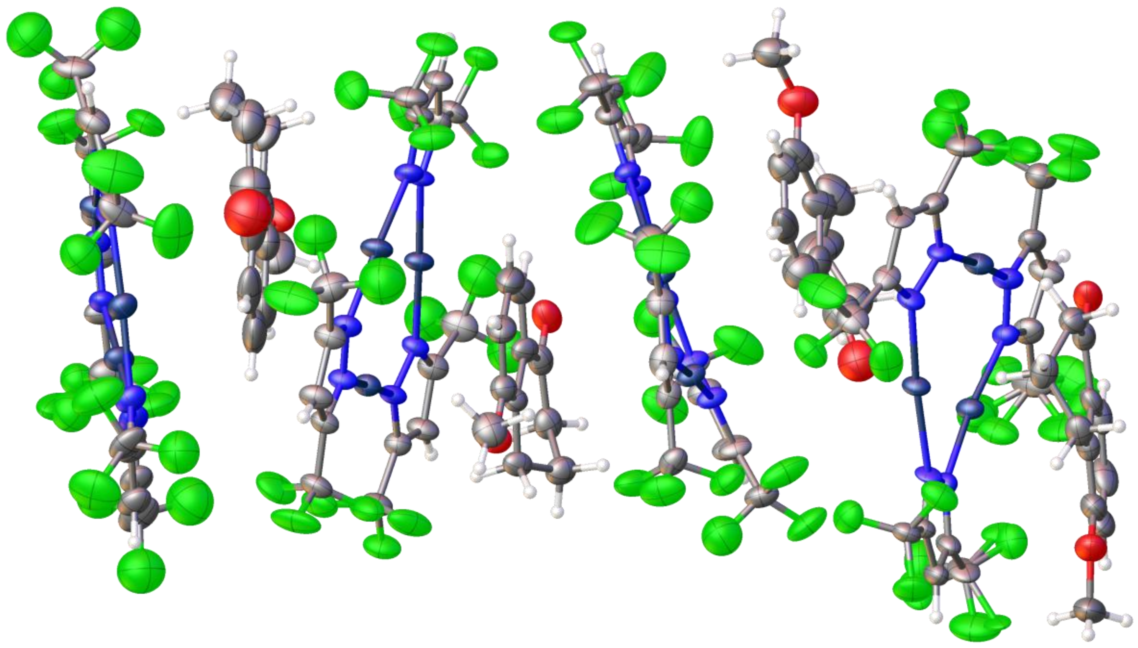

Figure S94. Asymmetric unit of **Ag_3Pz_3·26** (thermal displacement parameters at the 50% probability level).

Table S34. Crystal data and structure refinement for **Ag_3Pz_3·26**

Empirical formula	$C_{26}H_{15}Ag_3F_{18}N_6O_2$
Formula weight	1109.05
Temperature/K	100.15
Crystal system	monoclinic
Space group	$P2_1/c$
a/Å	24.8797(2)
b/Å	22.34730(10)
c/Å	25.4100(2)
α/°	90
β/°	110.2610(10)
γ/°	90
Volume/Å^3	13253.65(18)
Z	16
ρ_{calc}g/cm^3	2.223
μ/mm^{-1}	15.399
F(000)	8512.0
Crystal size/mm^3	0.16 × 0.15 × 0.12
Radiation	Cu Kα (λ = 1.54184)
2θ range for data collection/°	5.832 to 157.09
Index ranges	$-31 \leq h \leq 30$, $-28 \leq k \leq 27$, $-25 \leq l \leq 31$
Reflections collected	72002
Independent reflections	27284 [R_{int} = 0.0470, R_{sigma} = 0.0529]
Data/restraints/parameters	27284/261/2069
Goodness-of-fit on F^2	1.067
Final R indexes [I>=2σ (I)]	R_1 = 0.0766, wR_2 = 0.1986
Final R indexes [all data]	R_1 = 0.0938, wR_2 = 0.2061
Largest diff. peak/hole / e Å^{-3}	2.27/-1.52
CCDC-number	2501770

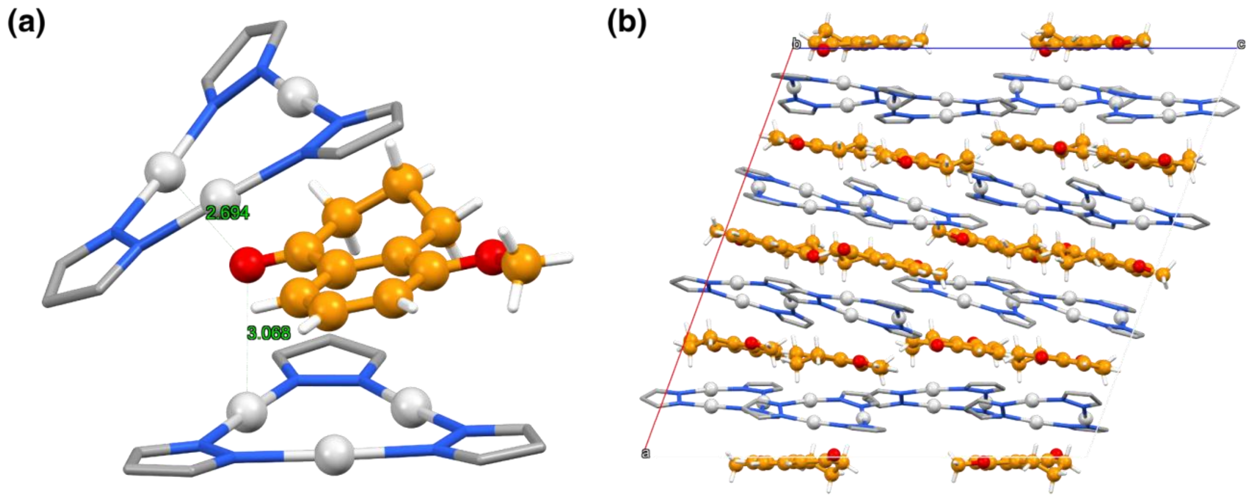

Figure S95. (a) A schematic diagram of the co-crystal structure in the **Ag_3Pz_3·26** single crystal, formed by the guest organic molecule and the surrounding Ag_3Pz_3 units that exhibit significant interactions with it. (b) A 1 × 1 × 1 packing mode in the single crystal structure of **Ag_3Pz_3·26** along the *b* axis. Trifluoromethyl groups and H atoms in Ag_3Pz_3 are omitted for clarity. Ag···O interactions are indicated with green dotted lines with distances in Å. C, N, and Ag atoms in Ag_3Pz_3 are depicted in dark gray, light blue, and light gray, respectively; C, O, and H atoms in **26** are depicted in orange, red, and white, respectively.

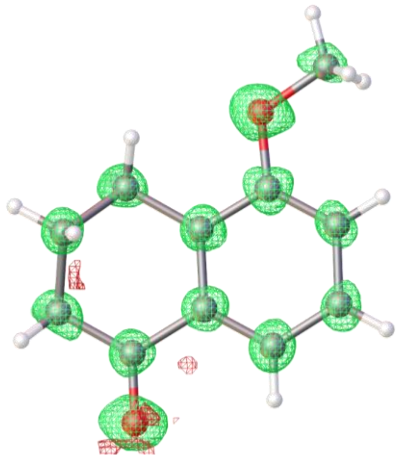

Figure S96. F_{obs} (contour: 0.20) electron density map superimposed on the structure of **26** in the single crystal structure of **Ag_3Pz_3·26**. We believe that the unassigned electron density is attributable to the Ag_3Pz_3 units.

Preparation of Ag_3Pz_3·27. 1.89 mg (0.0107 mmol) of 7-methoxy-1-tetralone (**27**) was dissolved in 3 mL of a binary solvent system of n-Hex and c-Hex (1:1, v/v), followed by the addition of equimolar amounts of Ag_3Pz_3 (10.00 mg, 0.0107 mmol). The resulting mixed solution was filtered and then transferred to a 20 mL screw-capped sample vial. The cap of the sample vial was loosely closed to allow the solvent to slowly evaporate at room temperature. The entire co-crystal incubation process was protected from light using aluminum foil. After the designated evaporation period, typically 1-3 days, high-quality colorless block-shaped crystals suitable for single-crystal X-ray diffraction analysis formed at the bottom of the vial.

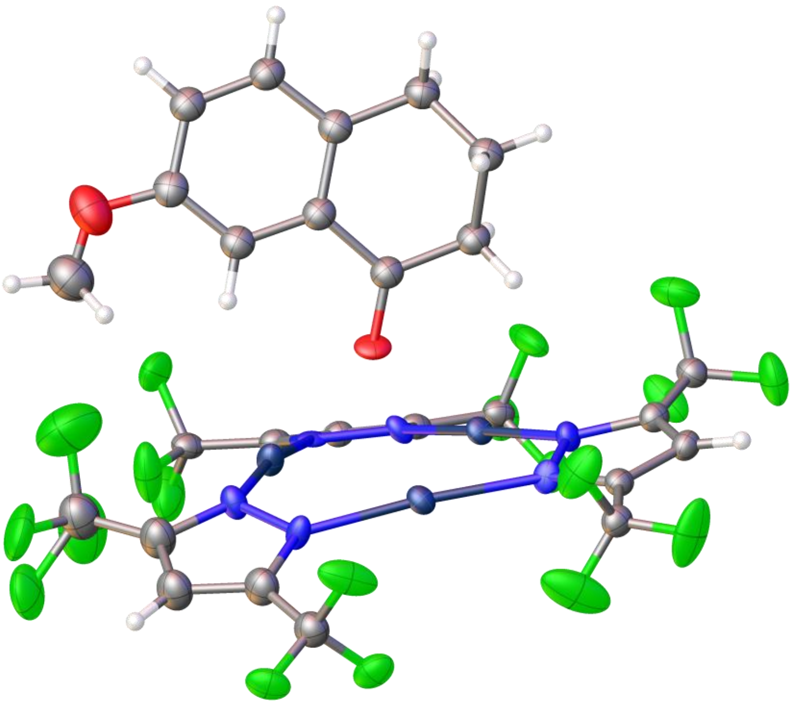

Figure S97. Asymmetric unit of **Ag_3Pz_3·27** (thermal displacement parameters at the 50% probability level).

Table S35. Crystal data and structure refinement for **Ag_3Pz_3·27**

Empirical formula	$C_{26}H_{15}Ag_3F_{18}N_6O_2$
Formula weight	1109.05
Temperature/K	100.15
Crystal system	monoclinic
Space group	$P2_1/c$
a/Å	23.3011(4)
b/Å	9.4996(2)
c/Å	15.8191(3)
α/°	90
β/°	108.666(2)
γ/°	90
Volume/Å^3	3317.39(12)
Z	4
ρ_{calc}g/cm^3	2.221
μ/mm^{-1}	15.380
F(000)	2128.0
Crystal size/mm^3	0.15 × 0.14 × 0.12
Radiation	Cu Kα (λ = 1.54184)
2θ range for data collection/°	8.01 to 149.202
Index ranges	$-28 \leq h \leq 29$, $-11 \leq k \leq 11$, $-14 \leq l \leq 19$
Reflections collected	18614
Independent reflections	6408 [R_{int} = 0.0857, R_{sigma} = 0.0505]
Data/restraints/parameters	6408/583/479
Goodness-of-fit on F^2	1.242
Final R indexes [I>=2σ (I)]	R_1 = 0.1154, wR_2 = 0.2894
Final R indexes [all data]	R_1 = 0.1303, wR_2 = 0.2963
Largest diff. peak/hole / e Å^{-3}	4.60/-2.25
CCDC-number	2501771

Responses to CheckCIF alerts for Ag_3Pz_3·27 crystal structure:

A-level alerts:

"Check Calcd Resid. Dens. 0.83Ang From Ag02 5.51 eA-3"

This Alert is due to presence of residual density in the presence of heavy metal atom (Ag).

"Check Calcd Resid. Dens. 0.85Ang From Ag02 4.91 eA-3"

This Alert is due to presence of residual density in the presence of heavy metal atom (Ag).

"Check Calcd Resid. Dens. 0.89Ang From Ag01 4.53 eA-3"

This Alert is due to presence of residual density in the presence of heavy metal atom (Ag).

"Check Calcd Resid. Dens. 0.93Ang From Ag01 4.51 eA-3"

This Alert is due to presence of residual density in the presence of heavy metal atom (Ag).

B-level alerts:

"Low Bond Precision on C-C Bonds .... 0.02474 Ang."

This Alert is due to presence of residual density in the presence of heavy metal atom (Ag).

"Check Calcd Resid. Dens. 1.11Ang From O00X 3.12 eA-3"

This Alert is due to presence of residual density in the presence of heavy metal atom (Ag).

"Check Calcd Resid. Dens. 0.87Ang From C01I 2.74 eA-3"

This Alert is due to presence of residual density in the presence of heavy metal atom (Ag).

"Check Calcd Resid. Dens. 0.88Ang From F5 2.74 eA-3"

This Alert is due to presence of residual density in the presence of heavy metal atom (Ag).

"Check Calcd Resid. Dens. 0.89Ang From Ag02 -2.62 eA-3"

This Alert is due to presence of residual density in the presence of heavy metal atom (Ag).

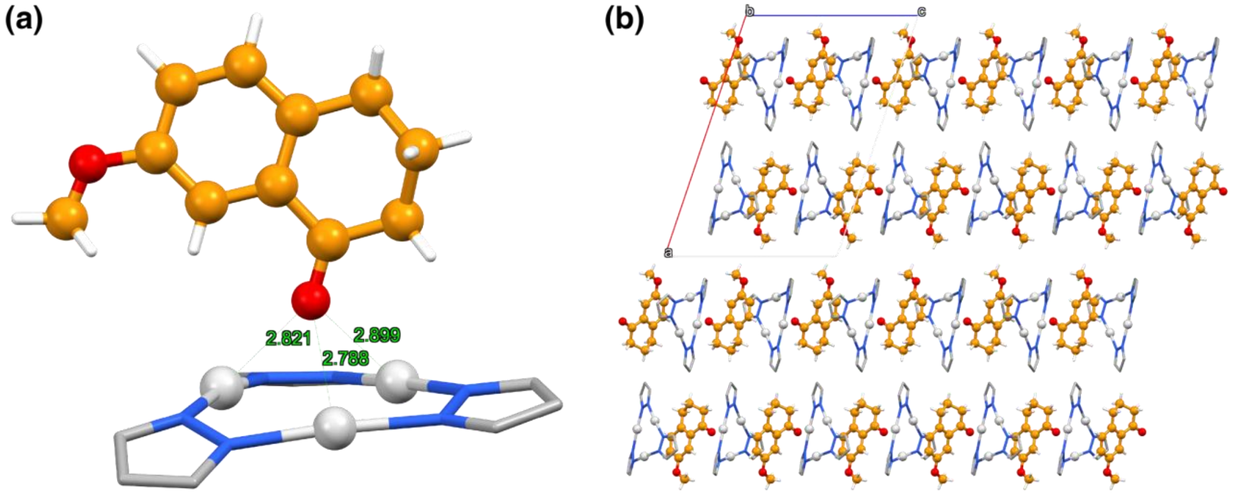

Figure S98. (a) A schematic diagram of the co-crystal structure in the **Ag_3Pz_3·27** single crystal, formed by the guest organic molecule and the surrounding Ag_3Pz_3 units that exhibit significant interactions with it. (b) A 2 × 1 × 3 packing mode in the single crystal structure of **Ag_3Pz_3·27** along the *b* axis. Trifluoromethyl groups and H atoms in Ag_3Pz_3 are omitted for clarity. Ag···O interactions are indicated with green dotted lines with distances in Å. C, N, and Ag atoms in Ag_3Pz_3 are depicted in dark gray, light blue, and light gray, respectively; C, O, and H atoms in **27** are depicted in orange, red, and white, respectively.

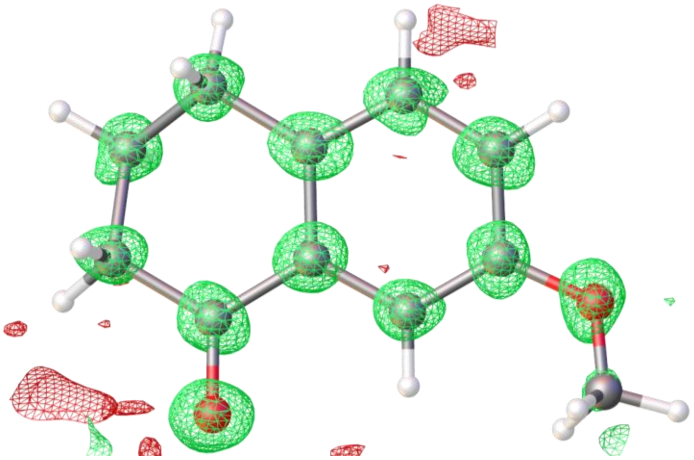

Figure S99. F_{obs} (contour: 0.70) electron density map superimposed on the structure of **27** in the single crystal structure of **Ag_3Pz_3·27**. We believe that the unassigned electron density is attributable to residual solvent molecules and the Ag_3Pz_3 units.

Preparation of Ag_3Pz_3·28. 2.06 mg (0.0107 mmol) of 1-(2,3-dihydrobenzo[b][1,4]dioxin-6-yl)propan-1-one (**28**) was dissolved in 3 mL of a binary solvent system of DCM and n-Hex (1:1, v/v), followed by the addition of equimolar amounts of Ag_3Pz_3 (10.00 mg, 0.0107 mmol). The resulting mixed solution was filtered and then transferred to a 20 mL screw-capped sample vial. The cap of the sample vial was loosely closed to allow the solvent to slowly evaporate at room temperature. The entire co-crystal incubation process was protected from light using aluminum foil. After the designated evaporation period, typically 1-3 days, high-quality colorless block-shaped crystals suitable for single-crystal X-ray diffraction analysis formed at the bottom of the vial.

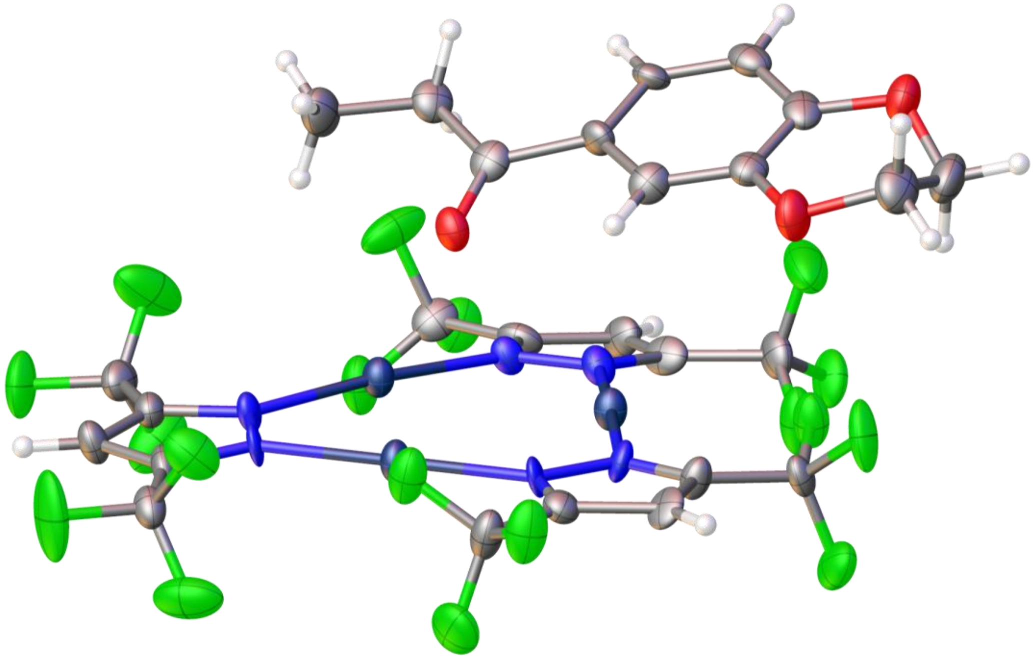

Figure S100. Asymmetric unit of **Ag_3Pz_3·28** (thermal displacement parameters at the 50% probability level).

Table S36. Crystal data and structure refinement for **Ag_3Pz_3·28**

Empirical formula	$C_{26}H_{15}Ag_3F_{18}N_6O_3$
Formula weight	1125.05
Temperature/K	100.01(19)
Crystal system	triclinic
Space group	*P*1
a/Å	8.4700(3)
b/Å	8.5735(3)
c/Å	12.4840(5)
α/°	74.978(3)
β/°	70.595(3)
γ/°	87.087(3)
Volume/Å^3	825.22(6)
Z	1
ρ_{calc}g/cm^3	2.264
μ/mm^{-1}	15.494
F(000)	540.0
Crystal size/mm^3	0.3 × 0.2 × 0.2
Radiation	Cu Kα (λ = 1.54184)
2θ range for data collection/°	7.77 to 155.81
Index ranges	$-8 \le h \le 10$, $-10 \le k \le 9$, $-15 \le l \le 15$
Reflections collected	6770
Independent reflections	3965 [R_{int} = 0.0436, R_{sigma} = 0.0352]
Data/restraints/parameters	3965/34/506
Goodness-of-fit on F^2	1.049
Final *R* indexes [I>=2σ (I)]	R_1 = 0.0517, wR_2 = 0.1384
Final *R* indexes [all data]	R_1 = 0.0523, wR_2 = 0.1388
Largest diff. peak/hole / e Å^{-3}	2.36/-1.28
Flack parameter	0.008(13)
CCDC-number	2501772

Responses to CheckCIF alert for Ag_3Pz_3·28 crystal structure:

(There is no A-level alert)

B-level alert:

"No Flack x Check Done: Low Friedel Pair Coverage 18 %"

Due to insufficient data. The compound crystallizes in the chiral space group, but no chiral ligand is used, and the complex is no longer chiral.

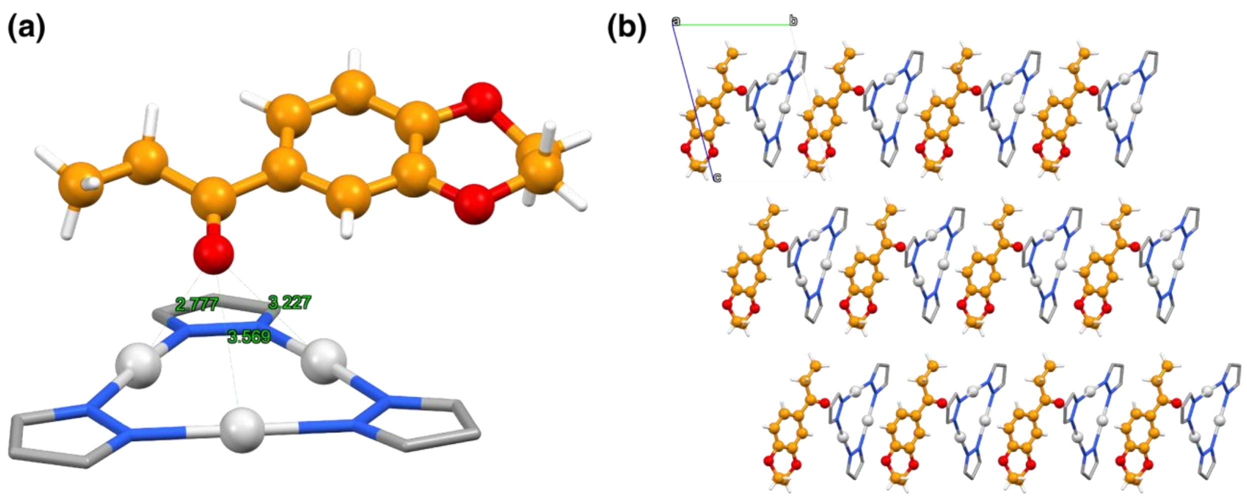

Figure S101. (a) A schematic diagram of the co-crystal structure in the **Ag_3Pz_3·28** single crystal, formed by the guest organic molecule and the surrounding Ag_3Pz_3 units that exhibit significant interactions with it. (b) A 1 × 4 × 3 packing mode in the single crystal structure of **Ag_3Pz_3·28** along the *a* axis. Trifluoromethyl groups and H atoms in Ag_3Pz_3 are omitted for clarity. Ag···O interactions are indicated with green dotted lines with distances in Å. C, N, and Ag atoms in Ag_3Pz_3 are depicted in dark gray, light blue, and light gray, respectively; C, O, and H atoms in **28** are depicted in orange, red, and white, respectively.

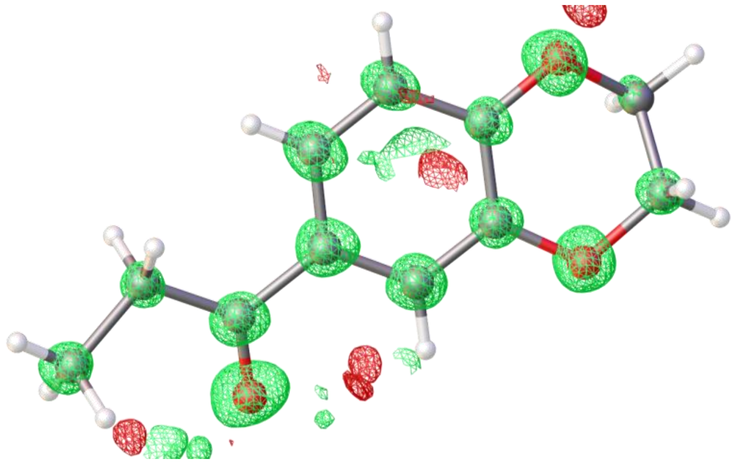

Figure S102. F_{obs} (contour: 1.00) electron density map superimposed on the structure of **28** in the single crystal structure of **Ag_3Pz_3·28**. We believe that the unassigned electron density is attributable to residual solvent molecules and the Ag_3Pz_3 units.

Preparation of Ag_3Pz_3·29. 1.74 mg (0.0107 mmol) of 6-methoxy-1-indanone (**29**) was dissolved in 3 mL of n-Hex, followed by the addition of equimolar amounts of Ag_3Pz_3 (10.00 mg, 0.0107 mmol). The resulting mixed solution was filtered and then transferred to a 20 mL screw-capped sample vial. The cap of the sample vial was loosely closed to allow the solvent to slowly evaporate at room temperature. The entire co-crystal incubation process was protected from light using aluminum foil. After the designated evaporation period, typically 1-3 days, high-quality colorless block-shaped crystals suitable for single-crystal X-ray diffraction analysis formed at the bottom of the vial.

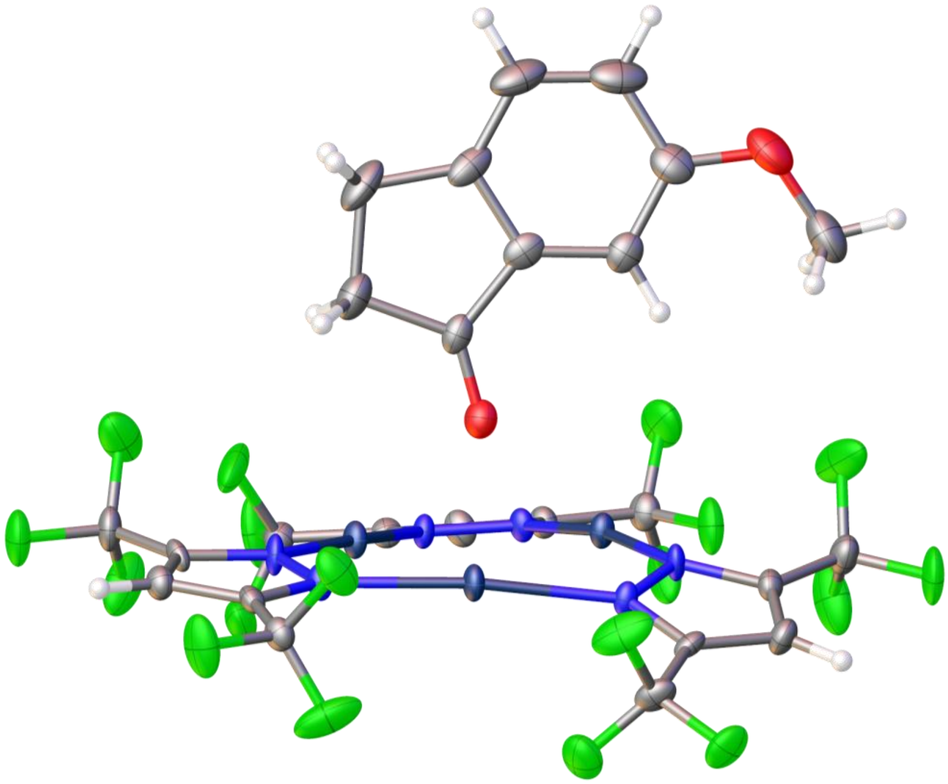

Figure S103. Asymmetric unit of **Ag_3Pz_3·29** (thermal displacement parameters at the 50% probability level).

Table S37. Crystal data and structure refinement for **Ag_3Pz_3·29**

Empirical formula	$C_{25}H_{13}Ag_3F_{18}N_6O_2$
Formula weight	1095.02
Temperature/K	99.97(15)
Crystal system	monoclinic
Space group	$P2_1/c$
a/Å	23.0450(3)
b/Å	9.46500(10)
c/Å	15.9513(2)
α/°	90
β/°	109.3400(10)
γ/°	90
Volume/Å^3	3282.97(7)
Z	4
ρ_{calc}g/cm^3	2.215
μ/mm^{-1}	15.531
F(000)	2096.0
Crystal size/mm^3	0.3 × 0.2 × 0.2
Radiation	Cu Kα (λ = 1.54184)
2θ range for data collection/°	8.132 to 156.754
Index ranges	$-29 \le h \le 29$, $-11 \le k \le 11$, $-19 \le l \le 16$
Reflections collected	34844
Independent reflections	6907 [R_{int} = 0.0914, R_{sigma} = 0.0412]
Data/restraints/parameters	6907/0/488
Goodness-of-fit on F^2	1.043
Final R indexes [I>=2σ (I)]	R_1 = 0.0608, wR_2 = 0.1641
Final R indexes [all data]	R_1 = 0.0630, wR_2 = 0.1663
Largest diff. peak/hole / e Å^{-3}	1.72/-2.30
CCDC-number	2501773

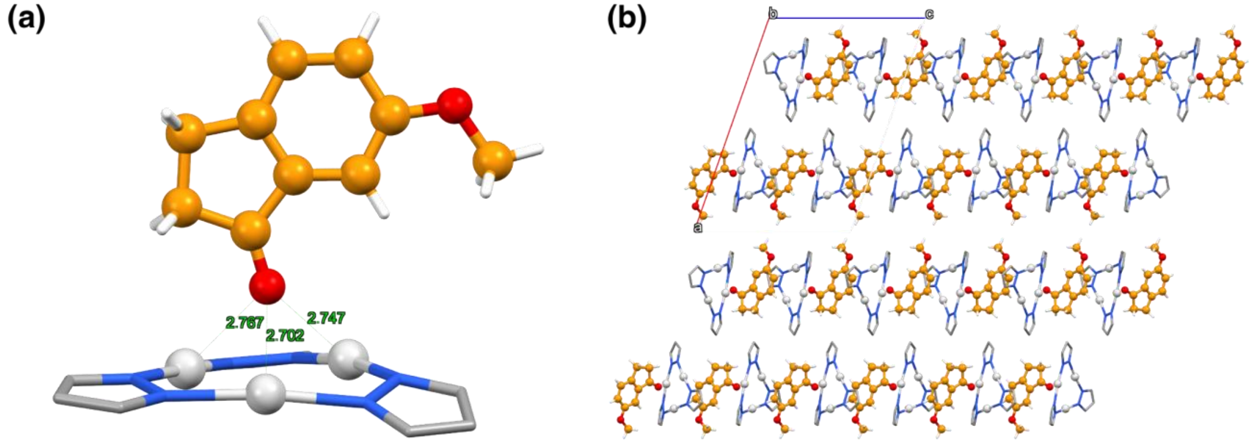

Figure S104. (a) A schematic diagram of the co-crystal structure in the $\mathbf{Ag_3Pz_3{\cdot}29}$ single crystal, formed by the guest organic molecule and the surrounding Ag_3Pz_3 units that exhibit significant interactions with it. (b) A 2 × 1 × 3 packing mode in the single crystal structure of $\mathbf{Ag_3Pz_3{\cdot}29}$ along the *b* axis. Trifluoromethyl groups and H atoms in Ag_3Pz_3 are omitted for clarity. Ag···O interactions are indicated with green dotted lines with distances in Å. C, N, and Ag atoms in Ag_3Pz_3 are depicted in dark gray, light blue, and light gray, respectively; C, O, and H atoms in **29** are depicted in orange, red, and white, respectively.

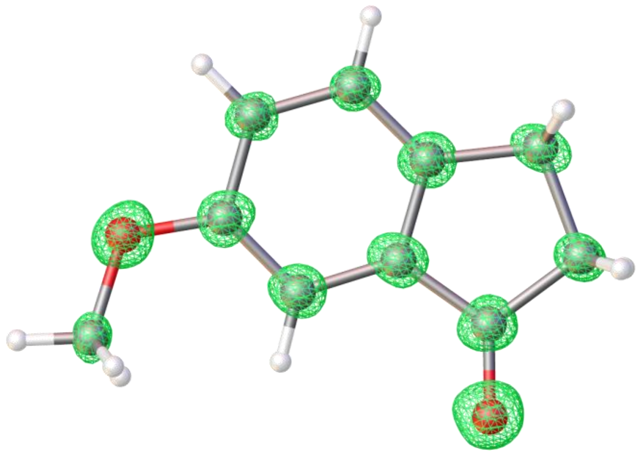

Figure S105. F_{obs} (contour: 1.00) electron density map superimposed on the structure of **29** in the single crystal structure of $\mathbf{Ag_3Pz_3{\cdot}29}$.

Preparation of Ag_3Pz_3·30. 1.74 mg (0.0107 mmol) of 4-methoxy-1-indanone (**30**) was dissolved in 3 mL of a binary solvent system of DCM and n-Hex (1:1, v/v), followed by the addition of equimolar amounts of Ag_3Pz_3 (10.00 mg, 0.0107 mmol). The resulting mixed solution was filtered and then transferred to a 20 mL screw-capped sample vial. The cap of the sample vial was loosely closed to allow the solvent to slowly evaporate at room temperature. The entire co-crystal incubation process was protected from light using aluminum foil. After the designated evaporation period, typically 1-3 days, high-quality colorless block-shaped crystals suitable for single-crystal X-ray diffraction analysis formed at the bottom of the vial.

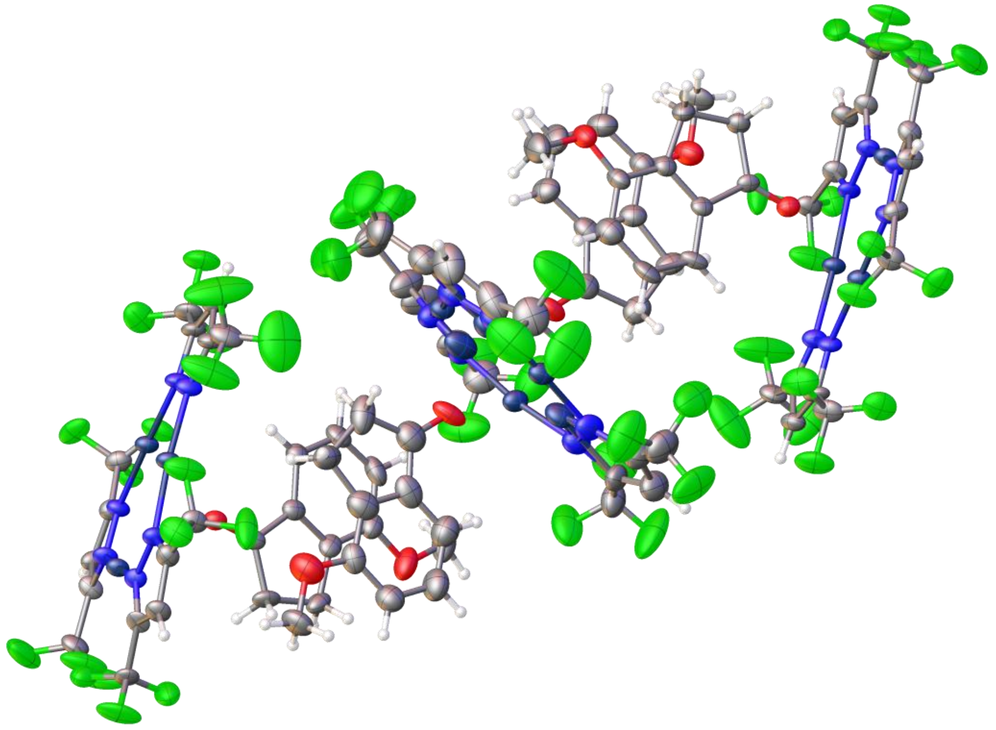

Figure S106. Asymmetric unit of **Ag_3Pz_3·30** (thermal displacement parameters at the 50% probability level).

Table S38. Crystal data and structure refinement for $\mathbf{Ag_3Pz_3 \cdot 30}$

Empirical formula	$C_{28.33}H_{16.33}Ag_3F_{18}N_6O_{2.67}$
Formula weight	1149.08
Temperature/K	99.97(18)
Crystal system	triclinic
Space group	$P\bar{1}$
a/Å	13.33030(10)
b/Å	21.6652(3)
c/Å	21.8805(3)
α/°	106.9040(10)
β/°	105.9880(10)
γ/°	98.3020(10)
Volume/Å^3	5636.51(12)
Z	6
ρ_{calc}g/cm^3	2.031
μ/mm^{-1}	13.622
F(000)	3316.0
Crystal size/mm^3	0.19 × 0.16 × 0.14
Radiation	Cu Kα (λ = 1.54184)
2θ range for data collection/°	5.07 to 156.698
Index ranges	$-13 \leq h \leq 16$, $-26 \leq k \leq 27$, $-26 \leq l \leq 27$
Reflections collected	61134
Independent reflections	23072 [R_{int} = 0.0453, R_{sigma} = 0.0534]
Data/restraints/parameters	23072/369/1562
Goodness-of-fit on F^2	1.096
Final R indexes [I>=2σ (I)]	R_1 = 0.0714, wR_2 = 0.1805
Final R indexes [all data]	R_1 = 0.0877, wR_2 = 0.1871
Largest diff. peak/hole / e Å^{-3}	2.72/-1.36
CCDC-number	2501775

Responses to CheckCIF alert for Ag_3Pz_3·30 crystal structure:

(There is no A-level alert)

B-level alert:

"Check Calcd Resid. Dens. 1.08Ang From C111 2.99 eA-3"

The residual density peak could not be modelled as any chemically sens.

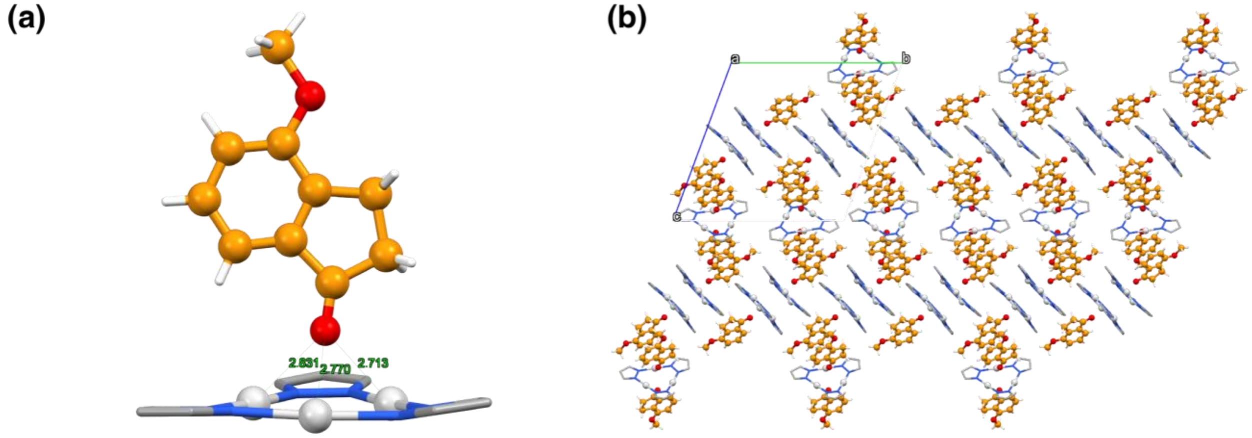

Figure S107. (a) A schematic diagram of the co-crystal structure in the **Ag_3Pz_3·30** single crystal, formed by the guest organic molecule and the surrounding Ag_3Pz_3 units that exhibit significant interactions with it. (b) A 1 × 3 × 2 packing mode in the single crystal structure of **Ag_3Pz_3·30** along the *a* axis. Trifluoromethyl groups and H atoms in Ag_3Pz_3 are omitted for clarity. Ag···O interactions are indicated with green dotted lines with distances in Å. C, N, and Ag atoms in Ag_3Pz_3 are depicted in dark gray, light blue, and light gray, respectively; C, O, and H atoms in 30 are depicted in orange, red, and white, respectively.

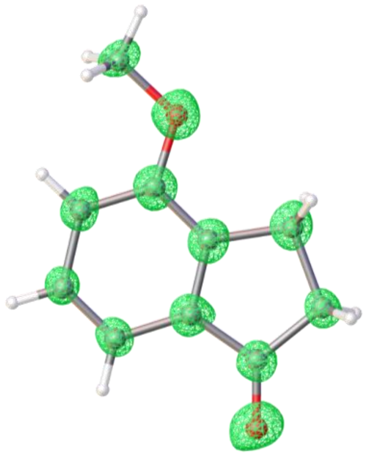

Figure S108. F_{obs} (contour: 0.55) electron density map superimposed on the structure of **30** in the single crystal structure of **Ag_3Pz_3·30**.

Preparation of Ag_3Pz_3·31. 2.06 mg (0.0107 mmol) of 4,7-dimethoxy-1-indanone (**31**) was dissolved in 3 mL of a binary solvent system of DCM and n-Hex (1:1, v/v), followed by the addition of equimolar amounts of Ag_3Pz_3 (10.00 mg, 0.0107 mmol). The resulting mixed solution was filtered and then transferred to a 20 mL screw-capped sample vial. The cap of the sample vial was loosely closed to allow the solvent to slowly evaporate at room temperature. The entire co-crystal incubation process was protected from light using aluminum foil. After the designated evaporation period, typically 1-3 days, high-quality colorless block-shaped crystals suitable for single-crystal X-ray diffraction analysis formed at the bottom of the vial.

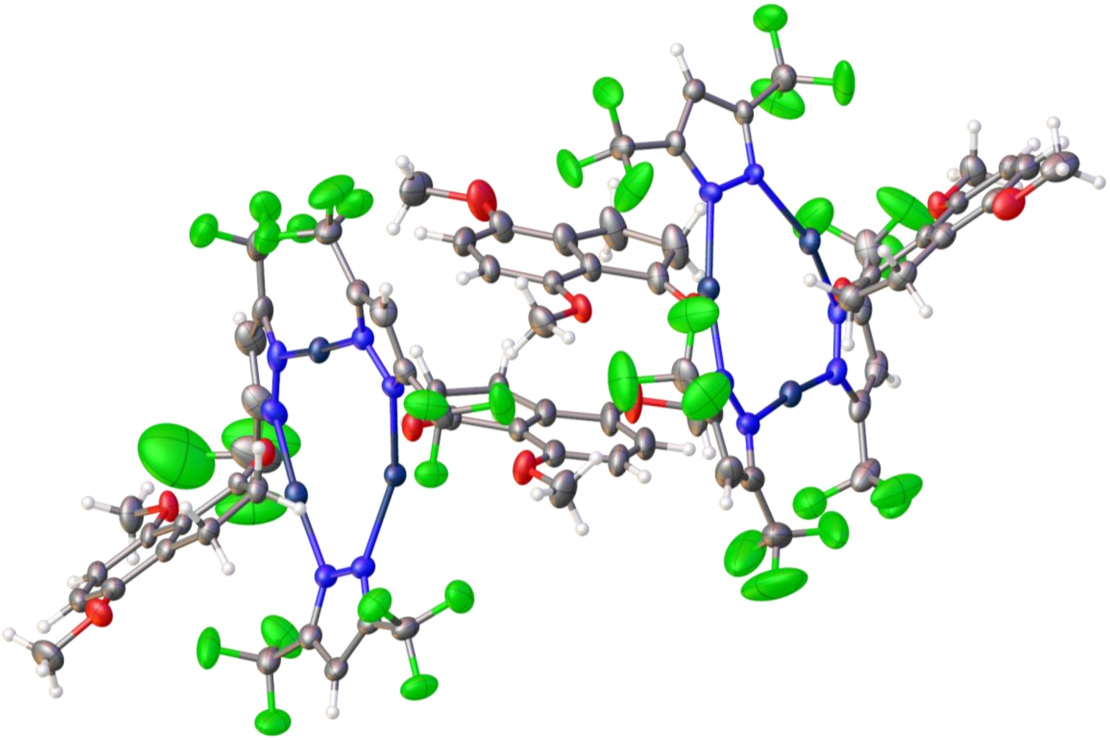

Figure S109. Asymmetric unit of **Ag_3Pz_3·31** (thermal displacement parameters at the 50% probability level).

Table S39. Crystal data and structure refinement for $\mathbf{Ag_3Pz_3 \cdot 31}$

Empirical formula	$C_{37}H_{27}Ag_3F_{18}N_6O_6$
Formula weight	1317.25
Temperature/K	100.0(3)
Crystal system	triclinic
Space group	$P\bar{1}$
a/Å	13.6963(2)
b/Å	17.6966(2)
c/Å	19.9309(4)
α/°	91.0360(10)
β/°	93.3600(10)
γ/°	106.9100(10)
Volume/Å^3	4610.87(13)
Z	4
ρ_{calc}g/cm^3	1.898
μ/mm^{-1}	11.258
F(000)	2568.0
Crystal size/mm^3	0.15 × 0.14 × 0.12
Radiation	Cu Kα (λ = 1.54184)
2θ range for data collection/°	6.734 to 157.388
Index ranges	$-17 \leq h \leq 15$, $-17 \leq k \leq 22$, $-25 \leq l \leq 25$
Reflections collected	46660
Independent reflections	18889 [R_{int} = 0.0441, R_{sigma} = 0.0521]
Data/restraints/parameters	18889/45/1269
Goodness-of-fit on F^2	1.076
Final R indexes [I>=2σ (I)]	R_1 = 0.0589, wR_2 = 0.1402
Final R indexes [all data]	R_1 = 0.0721, wR_2 = 0.1464
Largest diff. peak/hole / e Å^{-3}	2.41/-1.33
CCDC-number	2501776

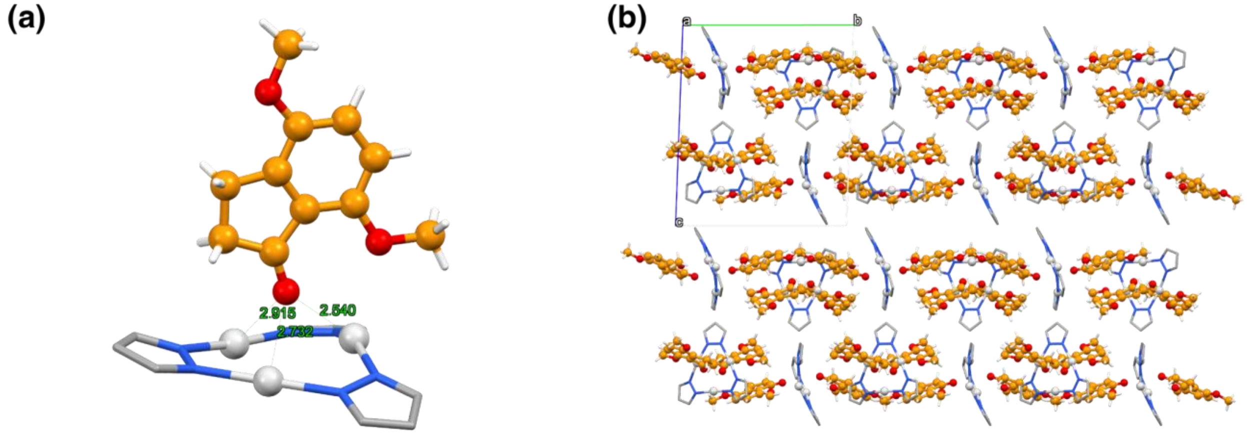

Figure S110. (a) A schematic diagram of the co-crystal structure in the $\mathbf{Ag_3Pz_3{\cdot}31}$ single crystal, formed by the guest organic molecule and the surrounding Ag_3Pz_3 units that exhibit significant interactions with it. (b) A 1 × 3 × 2 packing mode in the single crystal structure of $\mathbf{Ag_3Pz_3{\cdot}31}$ along the *a* axis. Trifluoromethyl groups and H atoms in Ag_3Pz_3 are omitted for clarity. Ag···O interactions are indicated with green dotted lines with distances in Å. C, N, and Ag atoms in Ag_3Pz_3 are depicted in dark gray, light blue, and light gray, respectively; C, O, and H atoms in **31** are depicted in orange, red, and white, respectively.

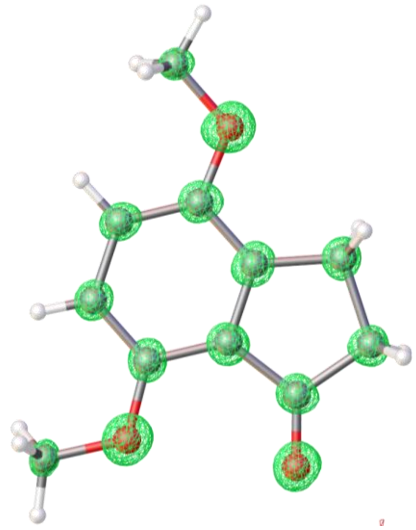

Figure S111. F_{obs} (contour: 0.84) electron density map superimposed on the structure of **31** in the single crystal structure of $\mathbf{Ag_3Pz_3{\cdot}31}$.

Preparation of Ag_3Pz_3·32. 2.38 mg (0.0107 mmol) of 4,5,6-trimethoxy-1-indanone (**32**) was dissolved in 3 mL of a binary solvent system of DCM and MeOH (1:1, v/v), followed by the addition of equimolar amounts of Ag_3Pz_3 (10.00 mg, 0.0107 mmol). The resulting mixed solution was filtered and then transferred to a 20 mL screw-capped sample vial. The cap of the sample vial was loosely closed to allow the solvent to slowly evaporate at room temperature. The entire co-crystal incubation process was protected from light using aluminum foil. After the designated evaporation period, typically 1-3 days, high-quality colorless needle-shaped crystals suitable for single-crystal X-ray diffraction analysis formed at the bottom of the vial.

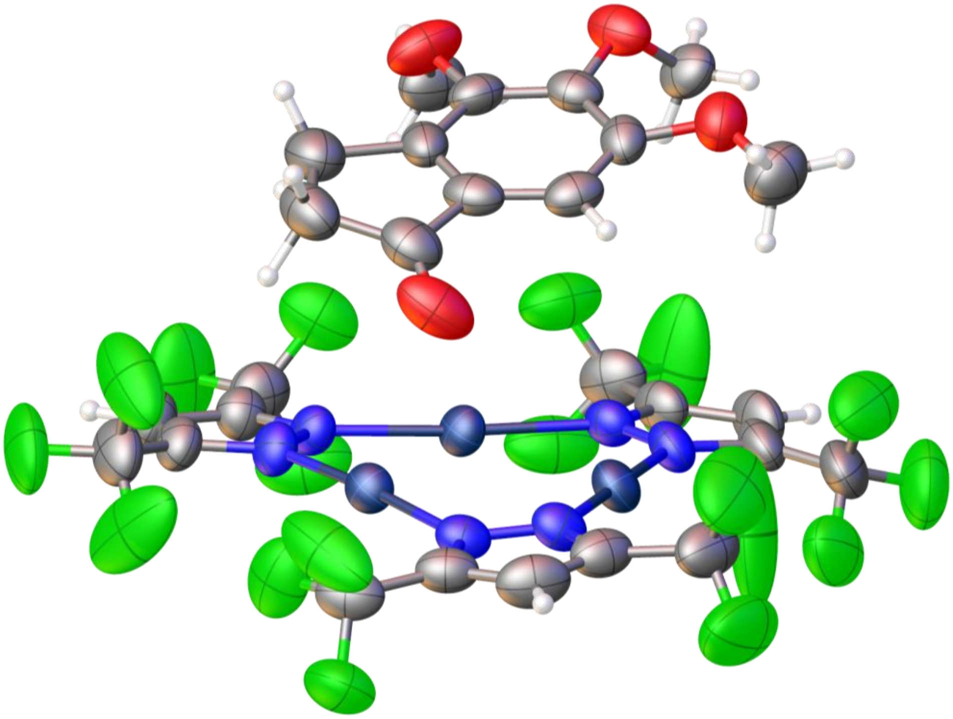

Figure S112. Asymmetric unit of **Ag_3Pz_3·32** (thermal displacement parameters at the 50% probability level).

Table S40. Crystal data and structure refinement for **Ag_3Pz_3·32**

Empirical formula	$C_{27}H_{15}Ag_3F_{18}N_6O_4$
Formula weight	1153.06
Temperature/K	100.00(10)
Crystal system	monoclinic
Space group	$P2_1/c$
a/Å	8.7328(2)
b/Å	18.1114(3)
c/Å	22.8152(3)
α/°	90
β/°	100.574(2)
γ/°	90
Volume/Å^3	3547.25(11)
Z	4
ρ_{calc}g/cm^3	2.159
μ/mm^{-1}	14.462
F(000)	2216.0
Crystal size/mm^3	0.24 × 0.23 × 0.14
Radiation	Cu Kα (λ = 1.54184)
2θ range for data collection/°	6.272 to 149.38
Index ranges	$-10 \leq h \leq 10$, $-18 \leq k \leq 22$, $-28 \leq l \leq 28$
Reflections collected	21886
Independent reflections	6985 [R_{int} = 0.0346, R_{sigma} = 0.0286]
Data/restraints/parameters	6985/1/534
Goodness-of-fit on F^2	1.035
Final R indexes [I>=2σ (I)]	R_1 = 0.0530, wR_2 = 0.1448
Final R indexes [all data]	R_1 = 0.0593, wR_2 = 0.1506
Largest diff. peak/hole / e Å^{-3}	1.85/-0.90
CCDC-number	2501777

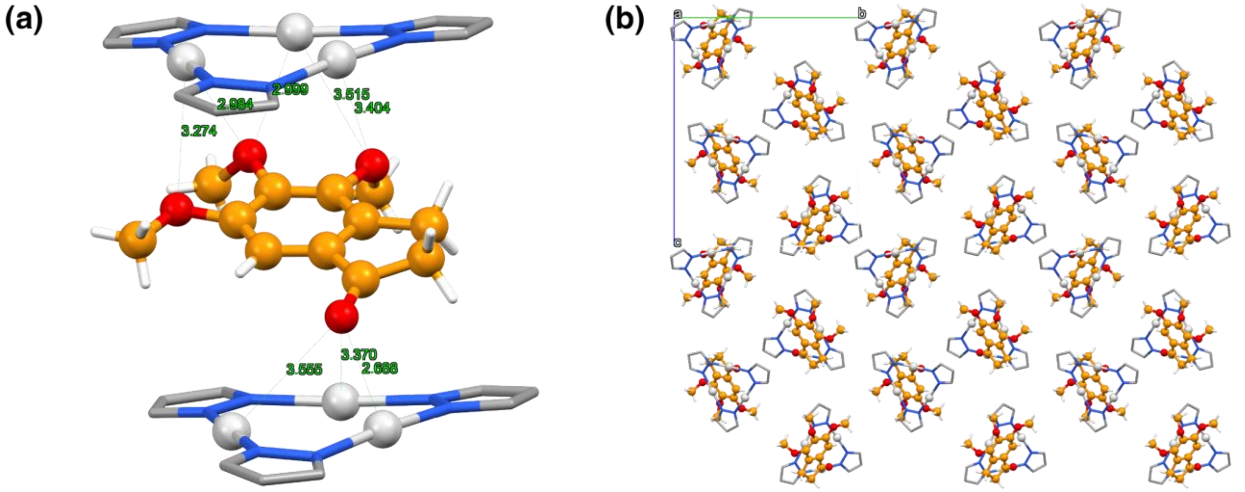

Figure S113. (a) A schematic diagram of the co-crystal structure in the **Ag_3Pz_3·32** single crystal, formed by the guest organic molecule and the surrounding Ag_3Pz_3 units that exhibit significant interactions with it. (b) A 1 × 3 × 2 packing mode in the single crystal structure of **Ag_3Pz_3·32** along the *a* axis. Trifluoromethyl groups and H atoms in Ag_3Pz_3 are omitted for clarity. Ag···O interactions are indicated with green dotted lines with distances in Å. C, N, and Ag atoms in Ag_3Pz_3 are depicted in dark gray, light blue, and light gray, respectively; C, O, and H atoms in **32** are depicted in orange, red, and white, respectively.

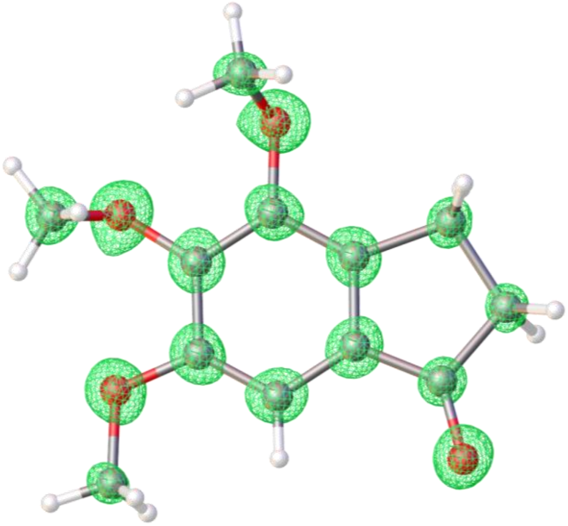

Figure S114. F_{obs} (contour: 0.50) electron density map superimposed on the structure of **32** in the single crystal structure of **Ag_3Pz_3·32**.

Preparation of Ag_3Pz_3·33. 2.04 mg (0.0107 mmol) of 2-methoxy-6,7,8,9-tetrahydrobenzocyclohepten-5-one (**33**) was dissolved in 3 mL of n-Hex, followed by the addition of equimolar amounts of Ag_3Pz_3 (10.00 mg, 0.0107 mmol). The resulting mixed solution was filtered and then transferred to a 20 mL screw-capped sample vial. The cap of the sample vial was loosely closed to allow the solvent to slowly evaporate at room temperature. The entire co-crystal incubation process was protected from light using aluminum foil. After the designated evaporation period, typically 1-3 days, high-quality colorless block-shaped crystals suitable for single-crystal X-ray diffraction analysis formed at the bottom of the vial.

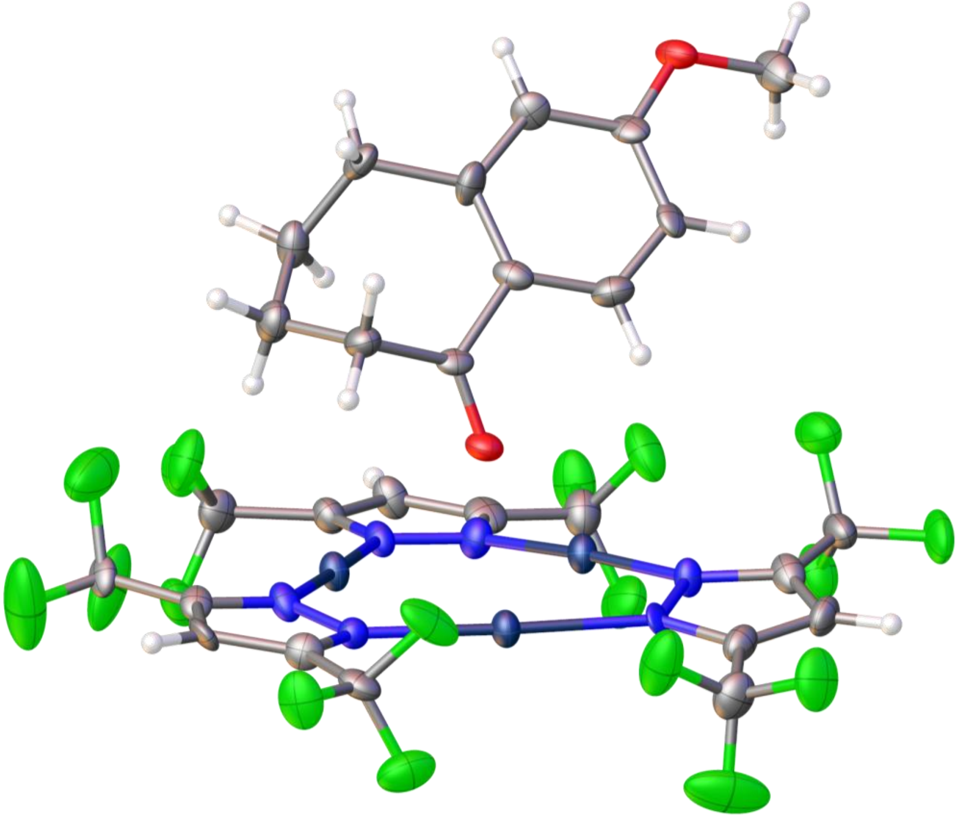

Figure S115. Asymmetric unit of **Ag_3Pz_3·33** (thermal displacement parameters at the 50% probability level).

Table S41. Crystal data and structure refinement for $\mathbf{Ag_3Pz_3 \cdot 33}$

Empirical formula	$C_{27}H_{17}Ag_3F_{18}N_6O_2$
Formula weight	1123.07
Temperature/K	100.00(10)
Crystal system	orthorhombic
Space group	*Pbca*
a/Å	11.1399(2)
b/Å	14.5492(3)
c/Å	42.2954(8)
α/°	90
β/°	90
γ/°	90
Volume/Å^3	6855.1(2)
Z	8
ρ_{calc}g/cm^3	2.176
μ/mm^{-1}	14.897
F(000)	4320.0
Crystal size/mm^3	0.16 × 0.15 × 0.1
Radiation	Cu Kα (λ = 1.54184)
2θ range for data collection/°	8.972 to 133.194
Index ranges	$-12 \leq h \leq 13$, $-14 \leq k \leq 17$, $-45 \leq l \leq 50$
Reflections collected	17116
Independent reflections	6004 [R_{int} = 0.0577, R_{sigma} = 0.0445]
Data/restraints/parameters	6004/42/506
Goodness-of-fit on F^2	1.646
Final R indexes [I>=2σ (I)]	R_1 = 0.1166, wR_2 = 0.3415
Final R indexes [all data]	R_1 = 0.1225, wR_2 = 0.3509
Largest diff. peak/hole / e Å^{-3}	6.46/-3.47
CCDC-number	2501778

Responses to CheckCIF alerts for Ag_3Pz_3·33 crystal structure:

A-level alerts:

"Check Calcd Resid. Dens. 1.09Ang From Ag02 6.38 eA-3"

This Alert is due to presence of residual density in the presence of heavy metal atom (Ag).

"Check Calcd Resid. Dens. 0.83Ang From Ag02 5.70 eA-3"

This Alert is due to presence of residual density in the presence of heavy metal atom (Ag).

"Check Calcd Resid. Dens. 0.82Ang From Ag03 5.64 eA-3"

This Alert is due to presence of residual density in the presence of heavy metal atom (Ag).

"Check Calcd Resid. Dens. 1.06Ang From Ag03 5.43 eA-3"

This Alert is due to presence of residual density in the presence of heavy metal atom (Ag).

"Check Calcd Resid. Dens. 1.02Ang From N00K 5.25 eA-3"

This Alert is due to presence of residual density in the presence of heavy metal atom (Ag).

"Check Calcd Resid. Dens. 1.16Ang From N00J 5.02 eA-3"

This Alert is due to presence of residual density in the presence of heavy metal atom (Ag).

"Calcd Positive Resid. Density on Ag03 3.23 eA-3"

This Alert is due to presence of residual density in the presence of heavy metal atom (Ag).

B-level alerts:

"Large Reported Max. (Positive) Residual Density 6.47 eA-3"

This Alert is due to presence of residual density in the presence of heavy metal atom (Ag).

"Check Calcd Resid. Dens. 0.95Ang From Ag02 2.69 eA-3"

This Alert is due to presence of residual density in the presence of heavy metal atom (Ag).

"Check Calcd Resid. Dens. 0.87Ang From Ag03 2.68 eA-3"

This Alert is due to presence of residual density in the presence of heavy metal atom (Ag).

"Check Calcd Resid. Dens. 0.93Ang From Ag03 2.55 eA-3"

This Alert is due to presence of residual density in the presence of heavy metal atom (Ag).

“Check Calcd Resid. Dens. 1.50Ang From F00P -3.50 eA-3”

This Alert is due to presence of residual density in the presence of heavy metal atom (Ag) and disordered structure.

“Check Calcd Resid. Dens. 0.97Ang From Ag03 -3.39 eA-3”

This Alert is due to presence of residual density in the presence of heavy metal atom (Ag) and disordered structure.

“Check Calcd Resid. Dens. 1.07Ang From Ag03 -3.14 eA-3”

This Alert is due to presence of residual density in the presence of heavy metal atom (Ag) and disordered structure.

“Check Calcd Resid. Dens. 1.49Ang From O00E -3.10 eA-3”

This Alert is due to presence of residual density in the presence of heavy metal atom (Ag) and disordered structure.

“Check Calcd Resid. Dens. 1.15Ang From Ag01 -3.04 eA-3”

This Alert is due to presence of residual density in the presence of heavy metal atom (Ag) and disordered structure.

“Check Calcd Resid. Dens. 1.59Ang From N00L -3.00 eA-3”

This Alert is due to presence of residual density in the presence of heavy metal atom (Ag) and disordered structure.

“Check Calcd Resid. Dens. 0.54Ang From Ag03 -2.98 eA-3”

This Alert is due to presence of residual density in the presence of heavy metal atom (Ag) and disordered structure.

“Check Calcd Resid. Dens. 1.63Ang From F007 -2.95 eA-3”

This Alert is due to presence of residual density in the presence of heavy metal atom (Ag) and disordered structure.

“Check Calcd Resid. Dens. 0.64Ang From N00K -2.94 eA-3”

This Alert is due to presence of residual density in the presence of heavy metal atom (Ag) and disordered structure.

“Check Calcd Resid. Dens. 1.03Ang From Ag02 -2.91 eA-3”

This Alert is due to presence of residual density in the presence of heavy metal atom (Ag) and disordered structure.

“Check Calcd Resid. Dens. 0.94Ang From Ag02 -2.89 eA-3”

This Alert is due to presence of residual density in the presence of heavy metal atom (Ag) and disordered structure.

“Check Calcd Resid. Dens. 1.20Ang From Ag02 -2.83 eA-3”

This Alert is due to presence of residual density in the presence of heavy metal atom (Ag) and disordered structure.

“Check Calcd Resid. Dens. 0.51Ang From Ag02 -2.82 eA-3”

This Alert is due to presence of residual density in the presence of heavy metal atom (Ag) and disordered structure.

“Check Calcd Resid. Dens. 1.09Ang From Ag03 -2.78 eA-3”

This Alert is due to presence of residual density in the presence of heavy metal atom (Ag) and disordered structure.

“Check Calcd Resid. Dens. 0.66Ang From N00J -2.68 eA-3”

This Alert is due to presence of residual density in the presence of heavy metal atom (Ag) and disordered structure.

“Check Calcd Resid. Dens. 0.88Ang From Ag01 -2.67 eA-3”

This Alert is due to presence of residual density in the presence of heavy metal atom (Ag) and disordered structure.

“Check Calcd Resid. Dens. 1.11Ang From Ag01 -2.66 eA-3”

This Alert is due to presence of residual density in the presence of heavy metal atom (Ag) and disordered structure.

“Check Calcd Resid. Dens. 1.08Ang From N00O -2.62 eA-3”

This Alert is due to presence of residual density in the presence of heavy metal atom (Ag) and disordered structure.

"Check Calcd Resid. Dens. 1.04Ang From Ag03 -2.60 eA-3"

This Alert is due to presence of residual density in the presence of heavy metal atom (Ag) and disordered structure.

"Check Calcd Resid. Dens. 1.02Ang From Ag03 -2.55 eA-3"

This Alert is due to presence of residual density in the presence of heavy metal atom (Ag) and disordered structure.

"Check Calcd Resid. Dens. 0.89Ang From Ag02 -2.55 eA-3"

This Alert is due to presence of residual density in the presence of heavy metal atom (Ag) and disordered structure.

"Check Calcd Resid. Dens. 1.23Ang From Ag02 -2.55 eA-3"

This Alert is due to presence of residual density in the presence of heavy metal atom (Ag) and disordered structure.

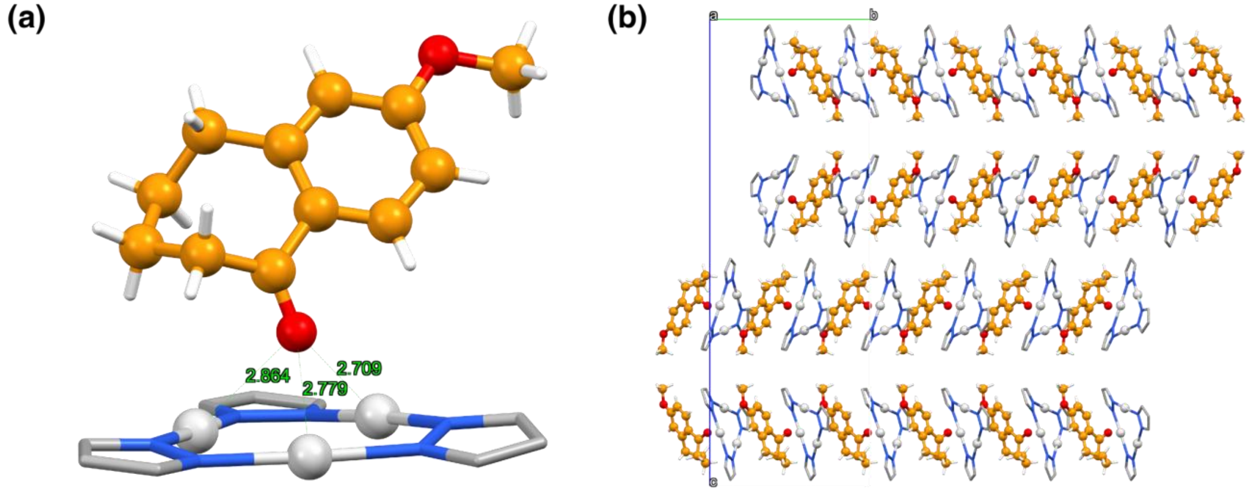

Figure S116. (a) A schematic diagram of the co-crystal structure in the **Ag_3Pz_3·33** single crystal, formed by the guest organic molecule and the surrounding Ag_3Pz_3 units that exhibit significant interactions with it. (b) A 1 × 3 × 1 packing mode in the single crystal structure of **Ag_3Pz_3·33** along the *a* axis. Trifluoromethyl groups and H atoms in Ag_3Pz_3 are omitted for clarity. Ag···O interactions are indicated with green dotted lines with distances in Å. C, N, and Ag atoms in Ag_3Pz_3 are depicted in dark gray, light blue, and light gray, respectively; C, O, and H atoms in **33** are depicted in orange, red, and white, respectively.

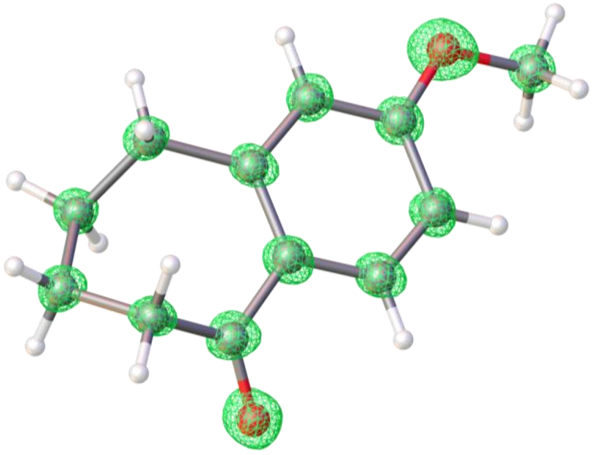

Figure S117. F_{obs} (contour: 0.56) electron density map superimposed on the structure of **33** in the single crystal structure of **Ag_3Pz_3·33**.

Preparation of Ag_3Pz_3·34. 1.56 mg (0.0107 mmol) of benzylideneacetone (**34**) was dissolved in 3 mL of n-Hex, followed by the addition of equimolar amounts of Ag_3Pz_3 (10.00 mg, 0.0107 mmol). The resulting mixed solution was filtered and then transferred to a 20 mL screw-capped sample vial. The cap of the sample vial was loosely closed to allow the solvent to slowly evaporate at room temperature. The entire co-crystal incubation process was protected from light using aluminum foil. After the designated evaporation period, typically 1-3 days, high-quality colorless needle-shaped crystals suitable for single-crystal X-ray diffraction analysis formed at the bottom of the vial.

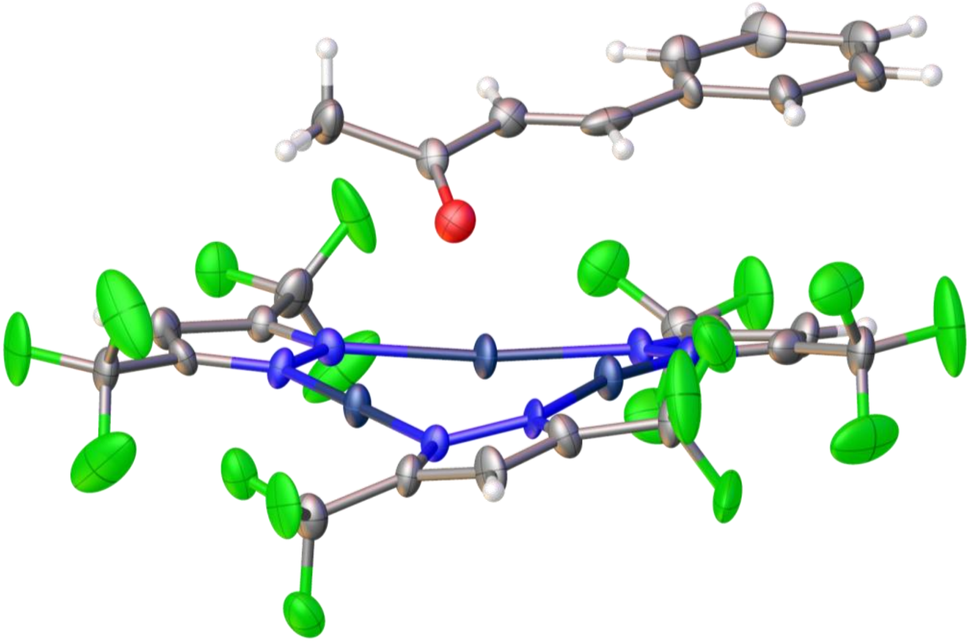

Figure S118. Asymmetric unit of **Ag_3Pz_3·34** (thermal displacement parameters at the 50% probability level).

Table S42. Crystal data and structure refinement for **Ag_3Pz_3·34**

Empirical formula	$C_{25}H_{13}Ag_3F_{18}N_6O$
Formula weight	1079.02
Temperature/K	99.99(17)
Crystal system	monoclinic
Space group	$P2_1/c$
a/Å	10.8243(2)
b/Å	17.4480(2)
c/Å	17.0198(3)
α/°	90
β/°	94.186(2)
γ/°	90
Volume/Å^3	3205.82(9)
Z	4
ρ_{calc}g/cm^3	2.236
μ/mm^{-1}	15.866
F(000)	2064.0
Crystal size/mm^3	0.26 × 0.15 × 0.14
Radiation	Cu Kα (λ = 1.54184)
2θ range for data collection/°	7.266 to 155.686
Index ranges	$-13 \leq h \leq 9$, $-21 \leq k \leq 15$, $-20 \leq l \leq 21$
Reflections collected	15820
Independent reflections	6542 [R_{int} = 0.0738, R_{sigma} = 0.0830]
Data/restraints/parameters	6542/130/461
Goodness-of-fit on F^2	1.065
Final R indexes [I>=2σ (I)]	R_1 = 0.0944, wR_2 = 0.2075
Final R indexes [all data]	R_1 = 0.1242, wR_2 = 0.2206
Largest diff. peak/hole / e Å^{-3}	1.58/-2.25
CCDC-number	2501779

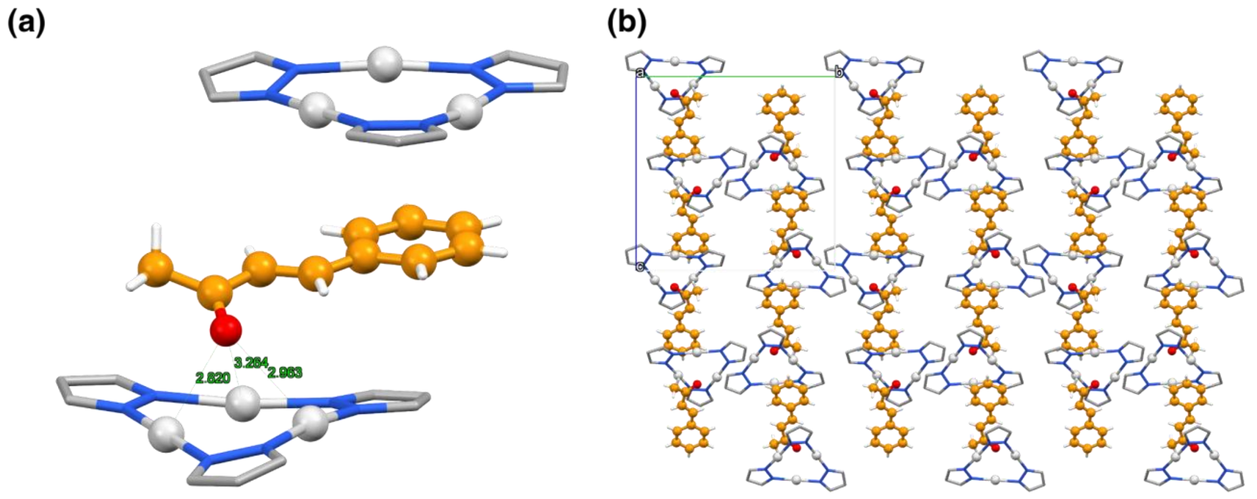

Figure S119. (a) A schematic diagram of the co-crystal structure in the $\mathbf{Ag_3Pz_3 \cdot 34}$ single crystal, formed by the guest organic molecule and the surrounding Ag_3Pz_3 units that exhibit significant interactions with it. (b) A 1 × 3 × 2 packing mode in the single crystal structure of $\mathbf{Ag_3Pz_3 \cdot 34}$ along the *a* axis. Trifluoromethyl groups and H atoms in Ag_3Pz_3 are omitted for clarity. Ag···O interactions are indicated with green dotted lines with distances in Å. C, N, and Ag atoms in Ag_3Pz_3 are depicted in dark gray, light blue, and light gray, respectively; C, O, and H atoms in **34** are depicted in orange, red, and white, respectively.

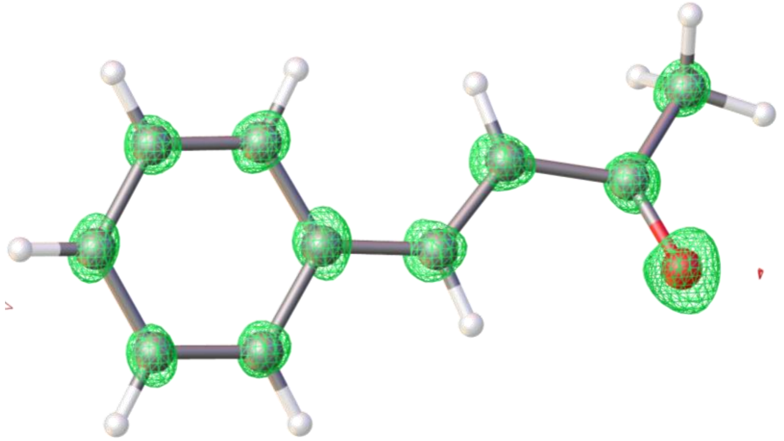

Figure S120. F_{obs} (contour: 0.55) electron density map superimposed on the structure of **34** in the single crystal structure of $\mathbf{Ag_3Pz_3 \cdot 34}$.

Preparation of Ag_3Pz_3·35. 2.08 mg (0.0107 mmol) of zingerone (**35**) was dissolved in 3 mL of a binary solvent system of DCM and c-Hex (1:1, v/v), followed by the addition of equimolar amounts of Ag_3Pz_3 (10.00 mg, 0.0107 mmol). The resulting mixed solution was filtered and then transferred to a 20 mL screw-capped sample vial. The cap of the sample vial was loosely closed to allow the solvent to slowly evaporate at room temperature. The entire co-crystal incubation process was protected from light using aluminum foil. After the designated evaporation period, typically 1-3 days, high-quality colorless block-shaped crystals suitable for single-crystal X-ray diffraction analysis formed at the bottom of the vial.

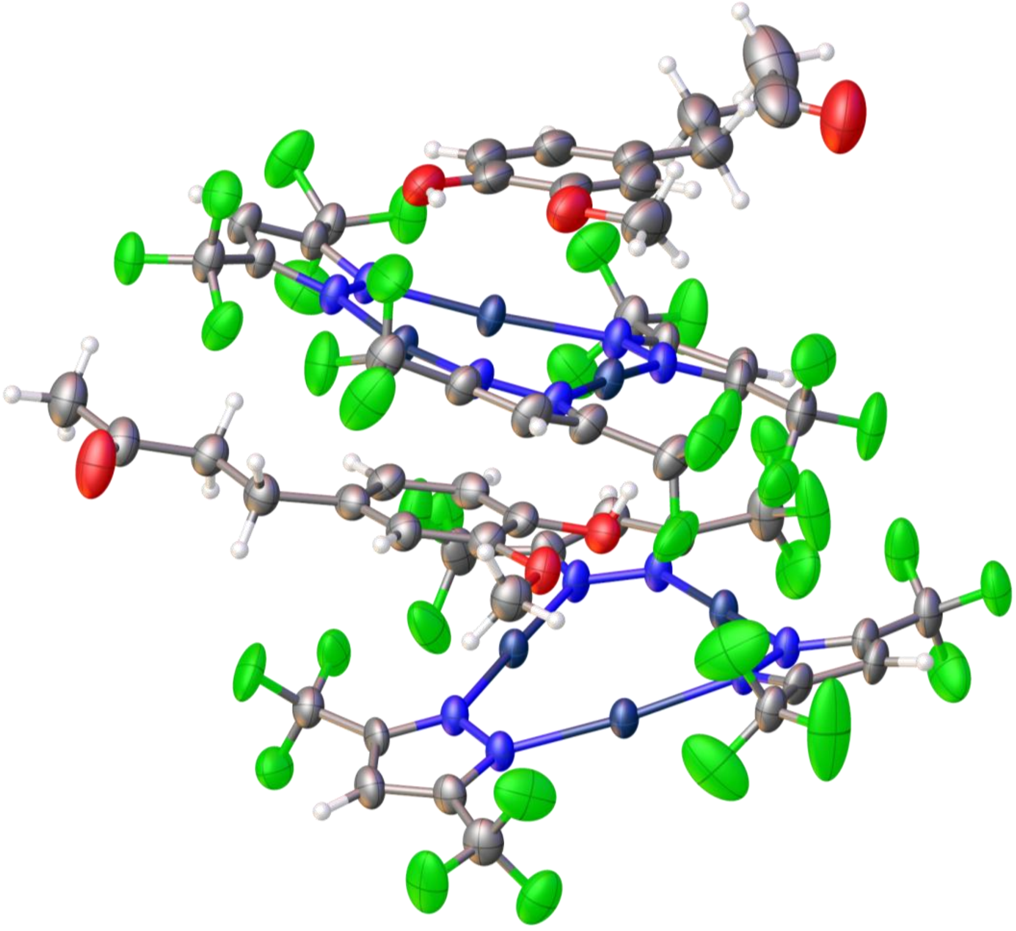

Figure S121. Asymmetric unit of **Ag_3Pz_3·35** (thermal displacement parameters at the 50% probability level).

Table S43. Crystal data and structure refinement for **Ag_3Pz_3·35**

Empirical formula	$C_{26}H_{17}Ag_3F_{18}N_6O_3$
Formula weight	1127.06
Temperature/K	100.00(10)
Crystal system	monoclinic
Space group	$P2_1/c$
a/Å	22.7183(2)
b/Å	13.04960(10)
c/Å	24.3237(4)
α/°	90
β/°	108.1520(10)
γ/°	90
Volume/Å^3	6852.24(14)
Z	8
ρ_{calc}g/cm^3	2.185
μ/mm^{-1}	14.928
F(000)	4336.0
Crystal size/mm^3	0.2 × 0.2 × 0.2
Radiation	Cu Kα (λ = 1.54184)
2θ range for data collection/°	7.468 to 157.064
Index ranges	$-28 \leq h \leq 28$, $-11 \leq k \leq 16$, $-29 \leq l \leq 26$
Reflections collected	38050
Independent reflections	14167 [R_{int} = 0.0382, R_{sigma} = 0.0463]
Data/restraints/parameters	14167/0/1015
Goodness-of-fit on F^2	1.116
Final R indexes [I>=2σ (I)]	R_1 = 0.0544, wR_2 = 0.1548
Final R indexes [all data]	R_1 = 0.0619, wR_2 = 0.1598
Largest diff. peak/hole / e Å^{-3}	2.54/-1.68
CCDC-number	2501780

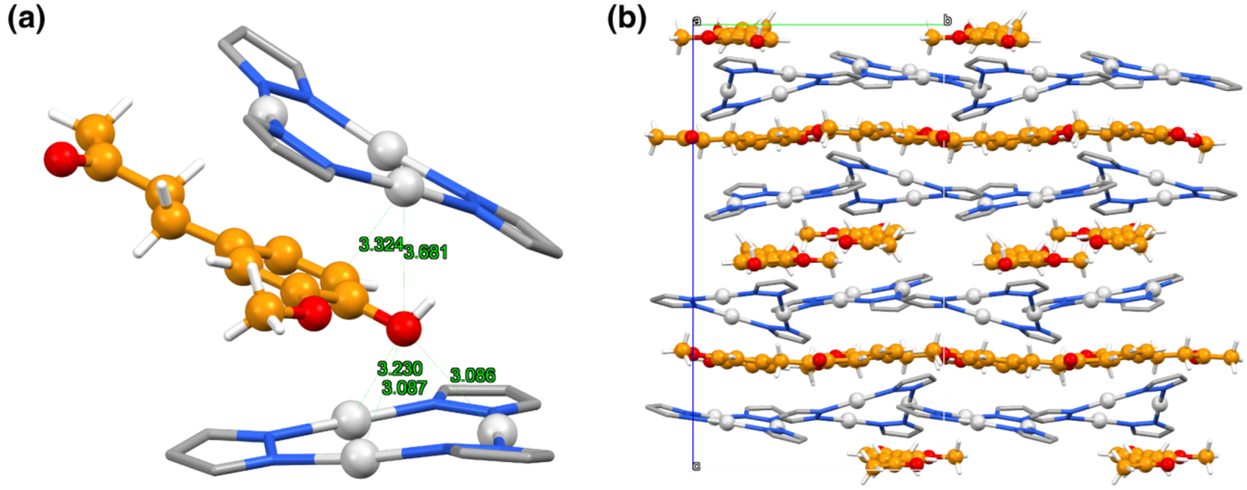

Figure S122. (a) A schematic diagram of the co-crystal structure in the **Ag_3Pz_3·35** single crystal, formed by the guest organic molecule and the surrounding Ag_3Pz_3 units that exhibit significant interactions with it. (b) A 1 × 2 × 1 packing mode in the single crystal structure of **Ag_3Pz_3·35** along the *a* axis. Trifluoromethyl groups and H atoms in Ag_3Pz_3 are omitted for clarity. Ag···O interactions are indicated with green dotted lines with distances in Å. C, N, and Ag atoms in Ag_3Pz_3 are depicted in dark gray, light blue, and light gray, respectively; C, O, and H atoms in **35** are depicted in orange, red, and white, respectively.

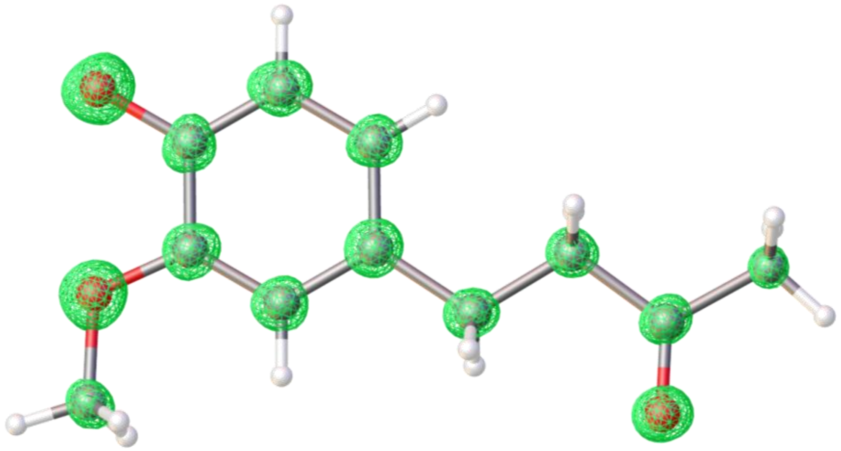

Figure S123. F_{obs} (contour: 0.40) electron density map superimposed on the structure of **35** in the single crystal structure of **Ag_3Pz_3·35**.

Preparation of Ag_3Pz_3·36. 2.70 mg (0.0107 mmol) of ethyl 3,4-dimethoxybenzoylacetate (**36**) was dissolved in 3 mL of a binary solvent system of DCM and c-Hex (1:1, v/v), followed by the addition of equimolar amounts of Ag_3Pz_3 (10.00 mg, 0.0107 mmol). The resulting mixed solution was filtered and then transferred to a 20 mL screw-capped sample vial. The cap of the sample vial was loosely closed to allow the solvent to slowly evaporate at room temperature. The entire co-crystal incubation process was protected from light using aluminum foil. After the designated evaporation period, typically 1-3 days, high-quality colorless block-shaped crystals suitable for single-crystal X-ray diffraction analysis formed at the bottom of the vial.

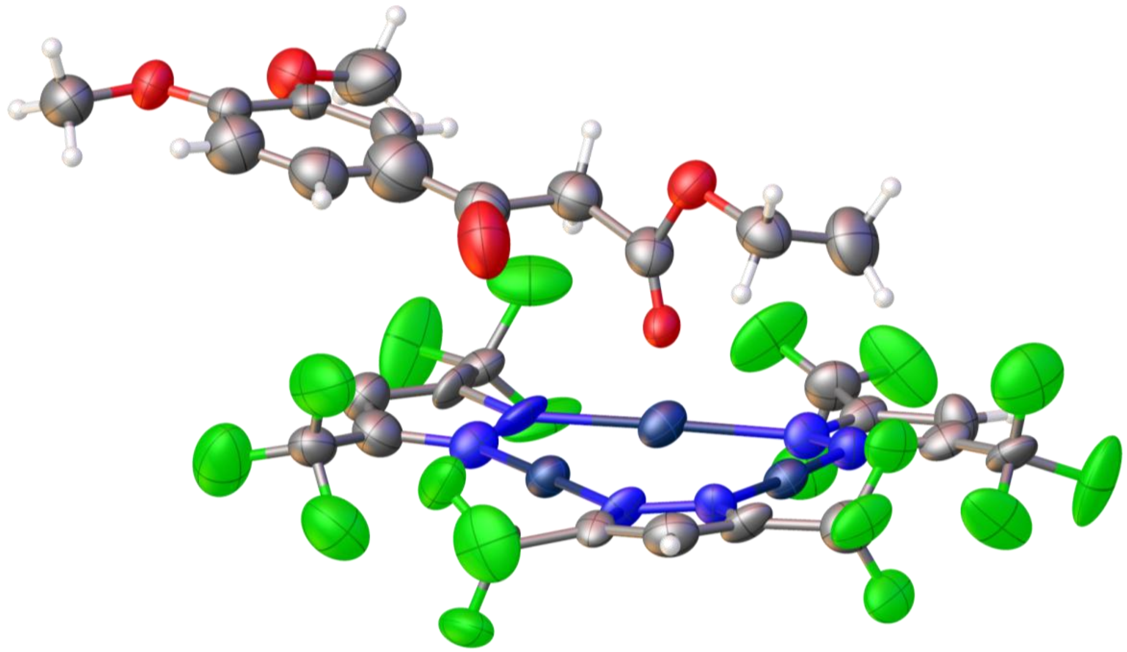

Figure S124. Asymmetric unit of **Ag_3Pz_3·36** (thermal displacement parameters at the 50% probability level).

Table S44. Crystal data and structure refinement for $\mathbf{Ag_3Pz_3{\cdot}36}$

Empirical formula	$C_{28}H_{19}Ag_3F_{18}N_6O_5$
Formula weight	1185.10
Temperature/K	100.00(10)
Crystal system	orthorhombic
Space group	$Pna2_1$
a/Å	14.7184(4)
b/Å	13.4029(4)
c/Å	18.7533(7)
α/°	90
β/°	90
γ/°	90
Volume/Å^3	3699.4(2)
Z	4
ρ_{calc}g/cm^3	2.128
μ/mm^{-1}	13.910
F(000)	2288.0
Crystal size/mm^3	0.19 × 0.16 × 0.15
Radiation	Cu Kα (λ = 1.54184)
2θ range for data collection/°	8.108 to 145.944
Index ranges	$-9 \le h \le 17$, $-16 \le k \le 16$, $-22 \le l \le 22$
Reflections collected	11767
Independent reflections	5868 [R_{int} = 0.0456, R_{sigma} = 0.0436]
Data/restraints/parameters	5868/293/524
Goodness-of-fit on F^2	1.776
Final R indexes [I>=2σ (I)]	R_1 = 0.1501, wR_2 = 0.3766
Final R indexes [all data]	R_1 = 0.1552, wR_2 = 0.3818
Largest diff. peak/hole / e Å^{-3}	4.82/-2.29
Flack parameter	0.18(4)
CCDC-number	2501781

Responses to CheckCIF alerts for Ag_3Pz_3·36 crystal structure:

A-level alerts:

"Check Calcd Resid. Dens. 0.76Ang From Ag03 4.73 eA-3"

This Alert is due to presence of residual density in the presence of heavy metal atom (Ag).

"Check Calcd Resid. Dens. 0.76Ang From Ag01 4.51 eA-3"

This Alert is due to presence of residual density in the presence of heavy metal atom (Ag).

"Check Calcd Resid. Dens. 0.38Ang From Ag01 4.14 eA-3"

This Alert is due to presence of residual density in the presence of heavy metal atom (Ag).

"Check Calcd Resid. Dens. 0.73Ang From Ag02 4.13 eA-3"

This Alert is due to presence of residual density in the presence of heavy metal atom (Ag).

"Check Calcd Resid. Dens. 0.97Ang From Ag02 4.12 eA-3"

This Alert is due to presence of residual density in the presence of heavy metal atom (Ag).

"Check Calcd Resid. Dens. 0.79Ang From Ag02 3.98 eA-3"

This Alert is due to presence of residual density in the presence of heavy metal atom (Ag).

"Check Calcd Resid. Dens. 0.96Ang From Ag01 3.98 eA-3"

This Alert is due to presence of residual density in the presence of heavy metal atom (Ag).

"Check Calcd Resid. Dens. 0.97Ang From Ag01 3.95 eA-3"

This Alert is due to presence of residual density in the presence of heavy metal atom (Ag).

"Check Calcd Positive Resid. Density on Ag03 3.77 eA-3"

This Alert is due to presence of residual density in the presence of heavy metal atom (Ag).

"Check Calcd Positive Resid. Density on Ag02 3.02 eA-3"

This Alert is due to presence of residual density in the presence of heavy metal atom (Ag).

B-level alerts:

"High wR2 Value (i.e. > 0.25) .... 0.38 Report"

Weakly diffracting crystal leading to relatively poor data of challenging sample.

“Large Reported Max. (Positive) Residual Density 4.82 eA-3”

This Alert is due to presence of residual density in the presence of heavy metal atom (Ag).

“Low Bond Precision on C-C Bonds .... 0.04773 Ang.”

Disordered structure.

“Check Calcd Resid. Dens. 0.77Ang From Ag03 3.37 eA-3”

This Alert is due to presence of residual density in the presence of heavy metal atom (Ag).

“Check Calcd Resid. Dens. 0.84Ang From Ag03 3.23 eA-3”

This Alert is due to presence of residual density in the presence of heavy metal atom (Ag).

“Check Calcd Resid. Dens. 0.95Ang From Ag03 3.14 eA-3”

This Alert is due to presence of residual density in the presence of heavy metal atom (Ag).

“Check Calcd Resid. Dens. 1.77Ang From Ag03 2.90 eA-3”

This Alert is due to presence of residual density in the presence of heavy metal atom (Ag).

“Check Calcd Resid. Dens. 0.75Ang From Ag02 2.90 eA-3”

This Alert is due to presence of residual density in the presence of heavy metal atom (Ag).

“Check Calcd Resid. Dens. 0.76Ang From Ag01 2.86 eA-3”

This Alert is due to presence of residual density in the presence of heavy metal atom (Ag).

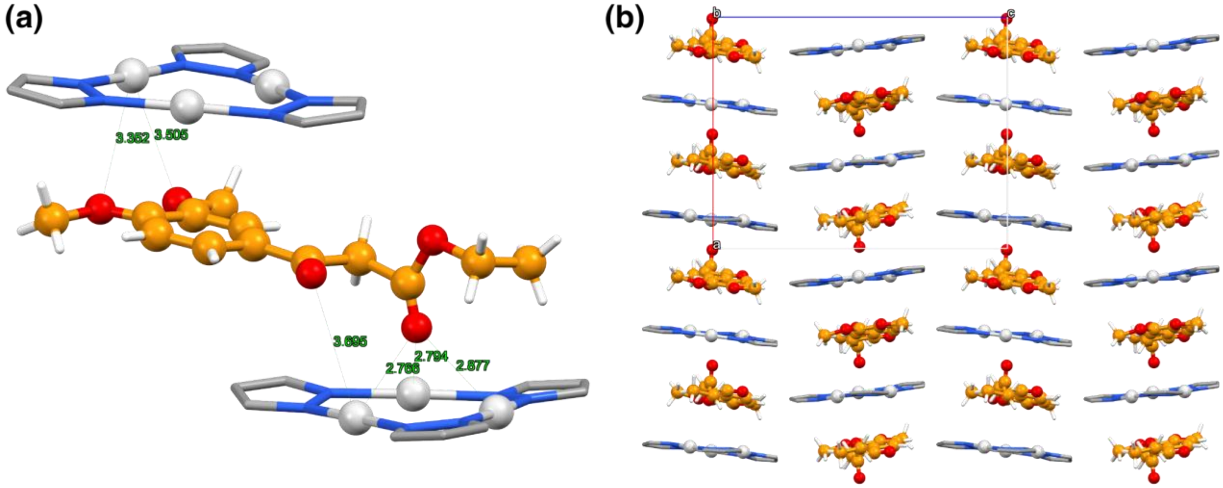

Figure S125. (a) A schematic diagram of the co-crystal structure in the **Ag_3Pz_3·36** single crystal, formed by the guest organic molecule and the surrounding Ag_3Pz_3 units that exhibit significant interactions with it. (b) A 2 × 1 × 2 packing mode in the single crystal structure of **Ag_3Pz_3·36** along the *b* axis. Trifluoromethyl groups and H atoms in Ag_3Pz_3 are omitted for clarity. Ag···O interactions are indicated with green dotted lines with distances in Å. C, N, and Ag atoms in Ag_3Pz_3 are depicted in dark gray, light blue, and light gray, respectively; C, O, and H atoms in **36** are depicted in orange, red, and white, respectively.

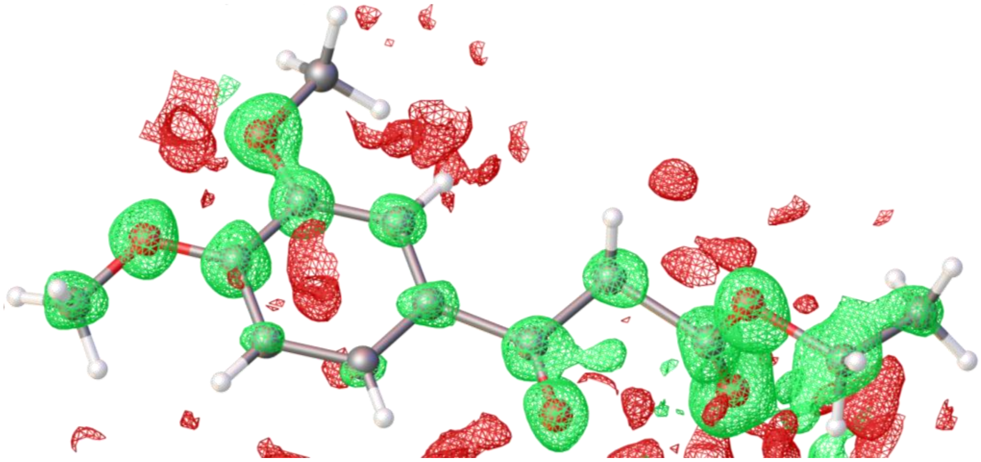

Figure S126. F_{obs} (contour: 0.30) electron density map superimposed on the structure of **36** in the single crystal structure of **Ag_3Pz_3·36**. We believe that the unassigned electron density is attributable to residual solvent molecules and the Ag_3Pz_3 units.

Preparation of Ag_3Pz_3·37. 2.23 mg (0.0107 mmol) of chalcone (**37**) was dissolved in 3 mL of n-Hex, followed by the addition of equimolar amounts of Ag_3Pz_3 (10.00 mg, 0.0107 mmol). The resulting mixed solution was filtered and then transferred to a 20 mL screw-capped sample vial. The cap of the sample vial was loosely closed to allow the solvent to slowly evaporate at room temperature. The entire co-crystal incubation process was protected from light using aluminum foil. After the designated evaporation period, typically 1-3 days, high-quality colorless needle-shaped crystals suitable for single-crystal X-ray diffraction analysis formed at the bottom of the vial.

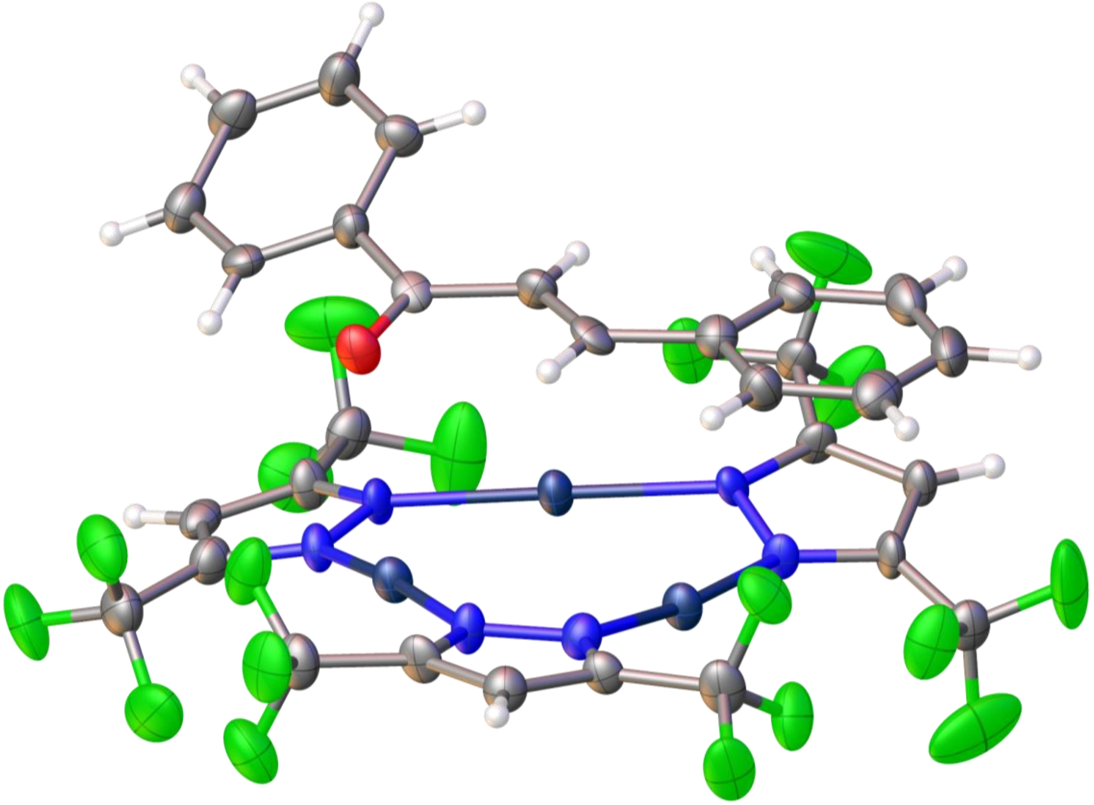

Figure S127. Asymmetric unit of **Ag_3Pz_3·37** (thermal displacement parameters at the 50% probability level).

Table S45. Crystal data and structure refinement for **Ag_3Pz_3·37**

Empirical formula	$C_{10}H_5AgF_6N_2O_{0.33}$
Formula weight	380.36
Temperature/K	100.00(10)
Crystal system	orthorhombic
Space group	$P2_12_12_1$
a/Å	12.1630(2)
b/Å	13.5555(2)
c/Å	21.4413(2)
α/°	90
β/°	90
γ/°	90
Volume/Å^3	3535.15(8)
Z	12
ρ_{calc}g/cm^3	2.144
μ/mm^{-1}	14.439
F(000)	2192.0
Crystal size/mm^3	0.1 × 0.1 × 0.1
Radiation	Cu Kα (λ = 1.54184)
2θ range for data collection/°	8.358 to 156.744
Index ranges	$-14 \leq h \leq 12$, $-15 \leq k \leq 17$, $-27 \leq l \leq 26$
Reflections collected	14474
Independent reflections	6950 [R_{int} = 0.0402, R_{sigma} = 0.0485]
Data/restraints/parameters	6950/0/523
Goodness-of-fit on F^2	1.059
Final R indexes [I>=2σ (I)]	R_1 = 0.0567, wR_2 = 0.1501
Final R indexes [all data]	R_1 = 0.0609, wR_2 = 0.1526
Largest diff. peak/hole / e Å^{-3}	1.78/-1.31
Flack parameter	-0.019(8)
CCDC-number	2501782

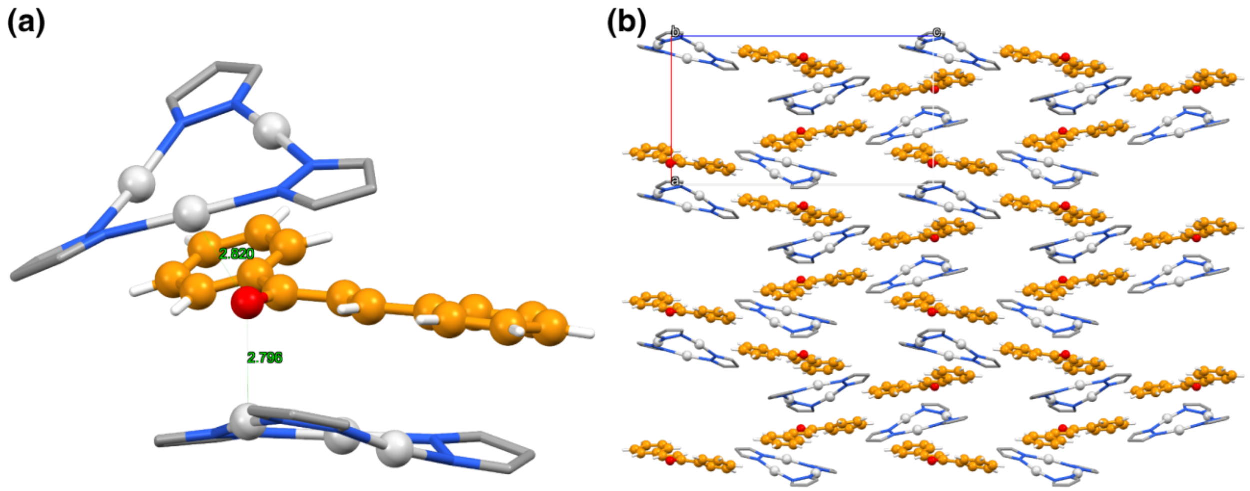

Figure S128. (a) A schematic diagram of the co-crystal structure in the **Ag_3Pz_3·37** single crystal, formed by the guest organic molecule and the surrounding Ag_3Pz_3 units that exhibit significant interactions with it. (b) A 3 × 1 × 2 packing mode in the single crystal structure of **Ag_3Pz_3·37** along the *b* axis. Trifluoromethyl groups and H atoms in Ag_3Pz_3 are omitted for clarity. Ag···O interactions are indicated with green dotted lines with distances in Å. C, N, and Ag atoms in Ag_3Pz_3 are depicted in dark gray, light blue, and light gray, respectively; C, O, and H atoms in **37** are depicted in orange, red, and white, respectively.

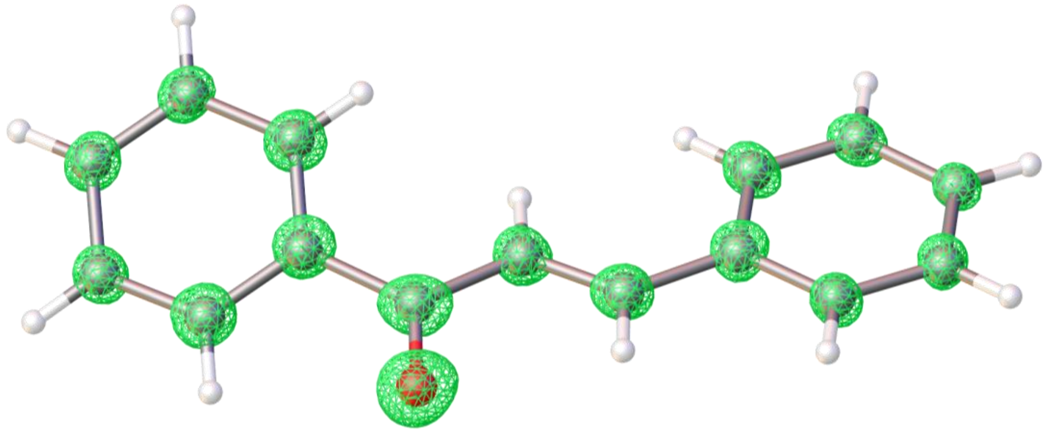

Figure S129. F_{obs} (contour: 1.30) electron density map superimposed on the structure of **37** in the single crystal structure of **Ag_3Pz_3·37**.

Preparation of Ag_3Pz_3·38. 2.40 mg (0.0107 mmol) of 2-hydroxychalcone (**38**) was dissolved in 3 mL of a binary solvent system of DCM and c-Hex (1:1, v/v), followed by the addition of equimolar amounts of Ag_3Pz_3 (10.00 mg, 0.0107 mmol). The resulting mixed solution was filtered and then transferred to a 20 mL screw-capped sample vial. The cap of the sample vial was loosely closed to allow the solvent to slowly evaporate at room temperature. The entire co-crystal incubation process was protected from light using aluminum foil. After the designated evaporation period, typically 1-3 days, high-quality colorless needle-shaped crystals suitable for single-crystal X-ray diffraction analysis formed at the bottom of the vial.

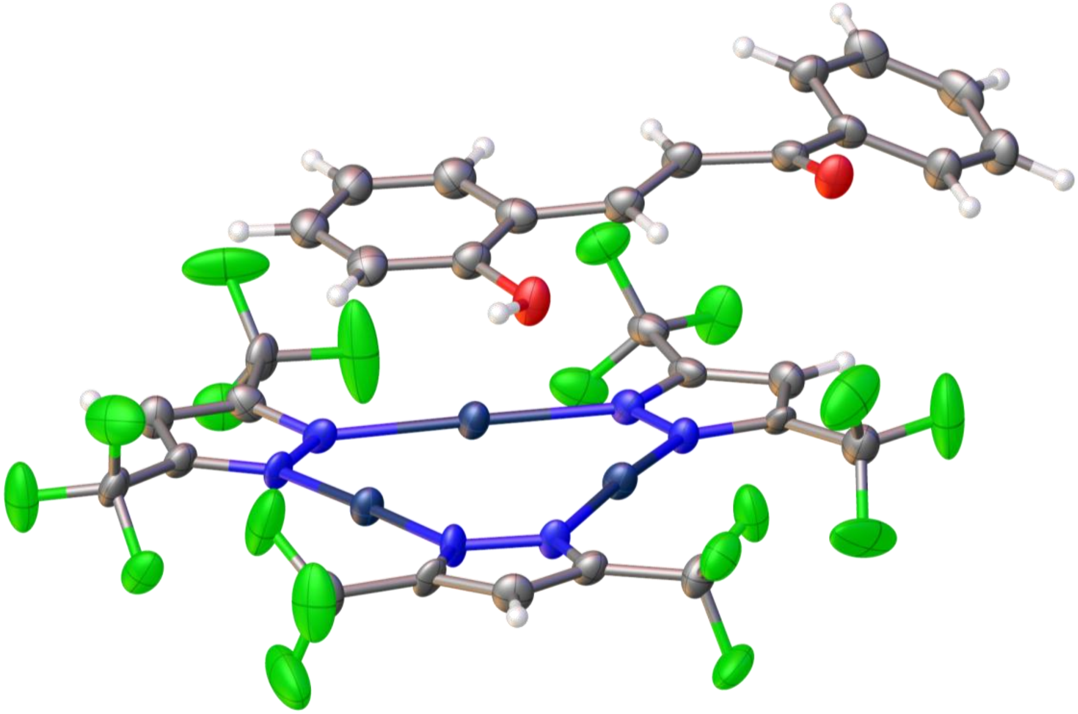

Figure S130. Asymmetric unit of **Ag_3Pz_3·38** (thermal displacement parameters at the 50% probability level).

Table S46. Crystal data and structure refinement for $\mathbf{Ag_3Pz_3 \cdot 38}$

Empirical formula	$C_{30}H_{15}Ag_3F_{18}N_6O_2$
Formula weight	1157.09
Temperature/K	100.01(11)
Crystal system	monoclinic
Space group	$P2_1/n$
a/Å	13.5121(2)
b/Å	12.8040(2)
c/Å	20.7098(3)
α/°	90
β/°	99.8610(10)
γ/°	90
Volume/Å^3	3530.05(9)
Z	4
ρ_{calc}g/cm^3	2.177
μ/mm^{-1}	14.495
F(000)	2224.0
Crystal size/mm^3	0.1 × 0.1 × 0.1
Radiation	Cu Kα (λ = 1.54184)
2θ range for data collection/°	7.28 to 157.736
Index ranges	$-12 \le h \le 17$, $-8 \le k \le 16$, $-25 \le l \le 26$
Reflections collected	19474
Independent reflections	7285 [R_{int} = 0.0524, R_{sigma} = 0.0576]
Data/restraints/parameters	7285/12/533
Goodness-of-fit on F^2	1.071
Final R indexes [I>=2σ (I)]	R_1 = 0.0587, wR_2 = 0.1440
Final R indexes [all data]	R_1 = 0.0791, wR_2 = 0.1512
Largest diff. peak/hole / e Å^{-3}	1.16/-1.10
CCDC-number	2501783

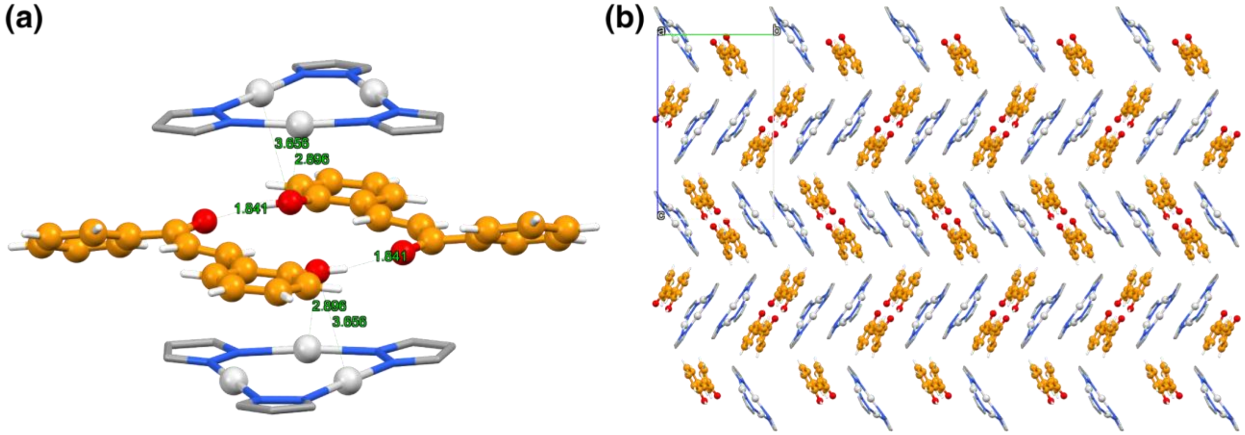

Figure S131. (a) A schematic diagram of the co-crystal structure in the **Ag_3Pz_3·38** single crystal, formed by the guest organic molecule and the surrounding Ag_3Pz_3 units that exhibit significant interactions with it. (b) A 1 × 5 × 2 packing mode in the single crystal structure of **Ag_3Pz_3·38** along the *a* axis. Trifluoromethyl groups and H atoms in Ag_3Pz_3 are omitted for clarity. Ag···O and O-H···O interactions are indicated with green dotted lines with distances in Å. C, N, and Ag atoms in Ag_3Pz_3 are depicted in dark gray, light blue, and light gray, respectively; C, O, and H atoms in **38** are depicted in orange, red, and white, respectively.

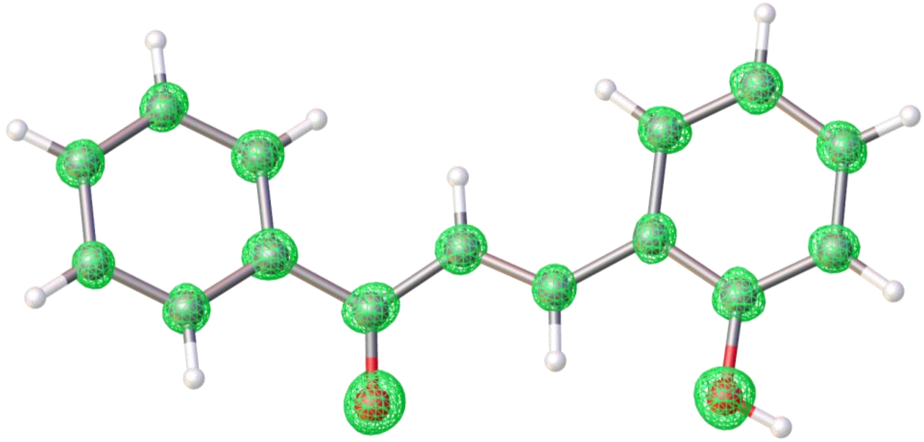

Figure S132. F_{obs} (contour: 0.90) electron density map superimposed on the structure of **38** in the single crystal structure of **Ag_3Pz_3·38**.

Preparation of Ag_3Pz_3·39. 2.40 mg (0.0107 mmol) of 4-hydroxychalcone (**39**) was dissolved in 3 mL of a binary solvent system of DCM and n-Hex (1:1, v/v), followed by the addition of equimolar amounts of Ag_3Pz_3 (10.00 mg, 0.0107 mmol). The resulting mixed solution was filtered and then transferred to a 20 mL screw-capped sample vial. The cap of the sample vial was loosely closed to allow the solvent to slowly evaporate at room temperature. The entire co-crystal incubation process was protected from light using aluminum foil. After the designated evaporation period, typically 1-3 days, high-quality colorless plate-shaped crystals suitable for single-crystal X-ray diffraction analysis formed at the bottom of the vial.

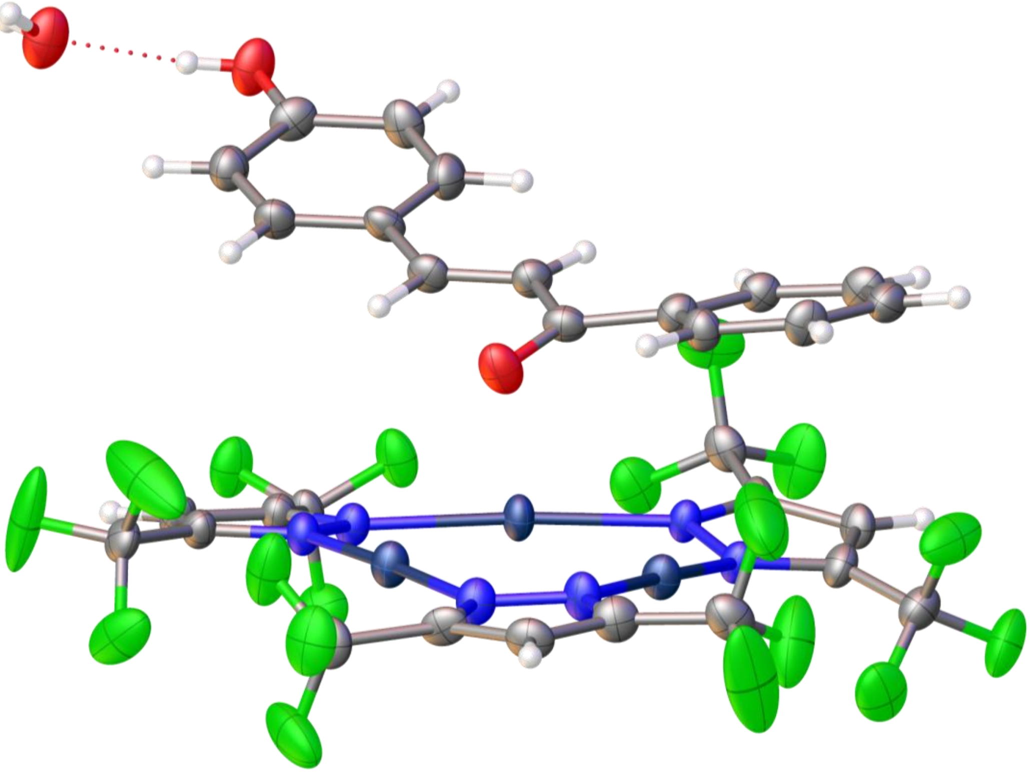

Figure S133. Asymmetric unit of **Ag_3Pz_3·39** (thermal displacement parameters at the 50% probability level).

Table S47. Crystal data and structure refinement for **Ag_3Pz_3·39**

Empirical formula	$C_{30}H_{17}Ag_3F_{18}N_6O_3$
Formula weight	1175.10
Temperature/K	100.0(3)
Crystal system	triclinic
Space group	$P\bar{1}$
a/Å	8.2215(2)
b/Å	13.1633(3)
c/Å	16.8689(4)
α/°	86.119(2)
β/°	83.075(2)
γ/°	88.010(2)
Volume/Å^3	1807.46(7)
Z	2
ρ_{calc}g/cm^3	2.159
μ/mm^{-1}	14.188
F(000)	1132.0
Crystal size/mm^3	0.21 × 0.16 × 0.15
Radiation	Cu Kα (λ = 1.54184)
2θ range for data collection/°	5.288 to 157.424
Index ranges	$-10 \leq h \leq 8$, $-14 \leq k \leq 16$, $-21 \leq l \leq 21$
Reflections collected	16631
Independent reflections	7378 [R_{int} = 0.0369, R_{sigma} = 0.0450]
Data/restraints/parameters	7378/0/545
Goodness-of-fit on F^2	1.070
Final R indexes [I>=2σ (I)]	R_1 = 0.0528, wR_2 = 0.1345
Final R indexes [all data]	R_1 = 0.0627, wR_2 = 0.1409
Largest diff. peak/hole / e Å^{-3}	1.74/-1.23
CCDC-number	2501784

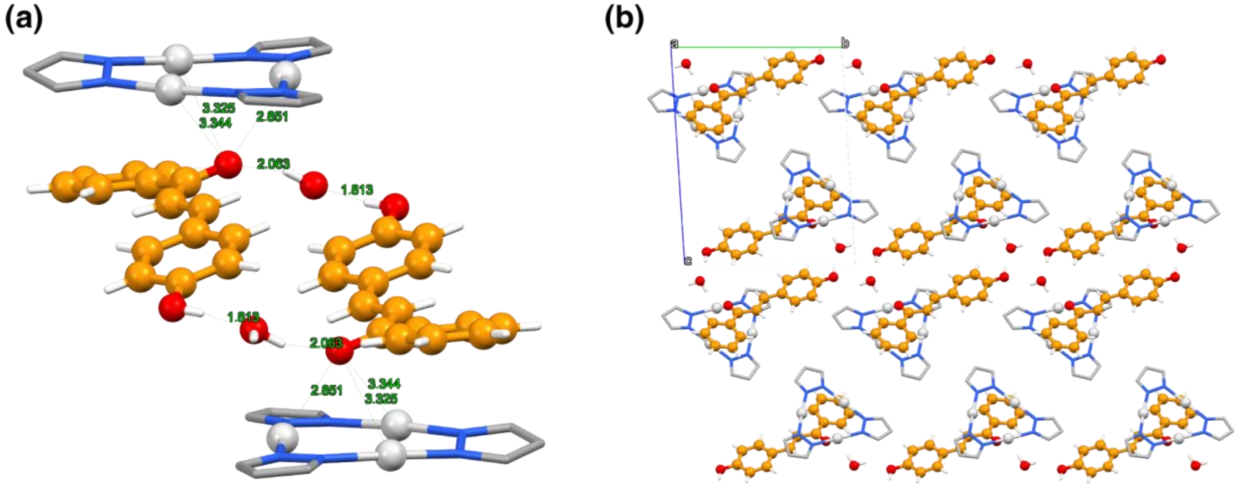

Figure S134. (a) A schematic diagram of the co-crystal structure in the **Ag_3Pz_3·39** single crystal, formed by the guest organic molecule and the surrounding Ag_3Pz_3 units that exhibit significant interactions with it. (b) A 1 × 3 × 2 packing mode in the single crystal structure of **Ag_3Pz_3·39** along the *a* axis. Trifluoromethyl groups and H atoms in Ag_3Pz_3 are omitted for clarity. Ag···O and O-H···O interactions are indicated with green dotted lines with distances in Å. C, N, and Ag atoms in Ag_3Pz_3 are depicted in dark gray, light blue, and light gray, respectively; C, O, and H atoms in **39** are depicted in orange, red, and white, respectively.

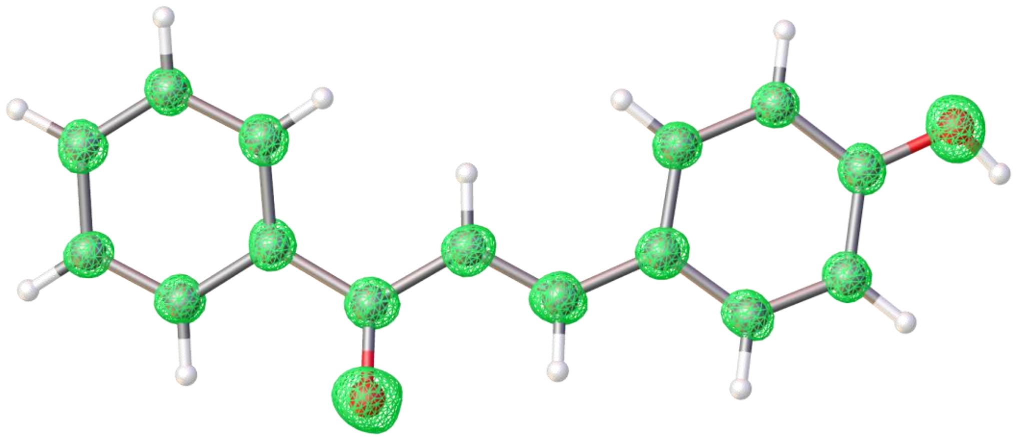

Figure S135. F_{obs} (contour: 1.70) electron density map superimposed on the structure of **39** in the single crystal structure of **Ag_3Pz_3·39**.

Preparation of Ag_3Pz_3·40. 2.55 mg (0.0107 mmol) of 4'-methoxychalcone (**40**) was dissolved in 3 mL of a binary solvent system of DCM and c-Hex (1:1, v/v), followed by the addition of equimolar amounts of Ag_3Pz_3 (10.00 mg, 0.0107 mmol). The resulting mixed solution was filtered and then transferred to a 20 mL screw-capped sample vial. The cap of the sample vial was loosely closed to allow the solvent to slowly evaporate at room temperature. The entire co-crystal incubation process was protected from light using aluminum foil. After the designated evaporation period, typically 1-3 days, high-quality colorless needle-shaped crystals suitable for single-crystal X-ray diffraction analysis formed at the bottom of the vial.

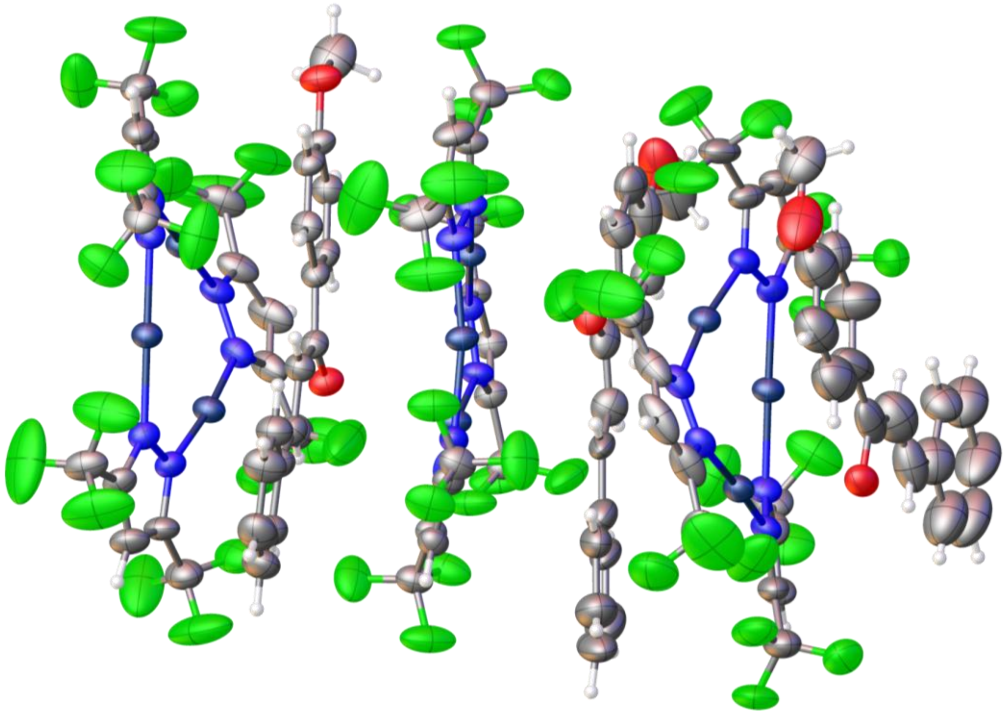

Figure S136. Asymmetric unit of **Ag_3Pz_3·40** (thermal displacement parameters at the 50% probability level).

Table S48. Crystal data and structure refinement for **Ag_3Pz_3·40**

Empirical formula	$C_{31}H_{17}Ag_3F_{18}N_6O_2$
Formula weight	1171.11
Temperature/K	100.15
Crystal system	triclinic
Space group	$P\bar{1}$
a/Å	15.2112(2)
b/Å	17.7741(3)
c/Å	22.4082(3)
α/°	90.0860(10)
β/°	91.1660(10)
γ/°	113.565(2)
Volume/Å^3	5551.71(16)
Z	6
ρ_{calc}g/cm^3	2.102
μ/mm^{-1}	13.834
F(000)	3384.0
Crystal size/mm^3	0.26 × 0.26 × 0.24
Radiation	Cu Kα (λ = 1.54184)
2θ range for data collection/°	5.424 to 133.198
Index ranges	$-18 \leq h \leq 16$, $-21 \leq k \leq 21$, $-26 \leq l \leq 26$
Reflections collected	65424
Independent reflections	19453 [R_{int} = 0.0589, R_{sigma} = 0.0463]
Data/restraints/parameters	19453/238/1594
Goodness-of-fit on F^2	1.029
Final R indexes [I>=2σ (I)]	R_1 = 0.0693, wR_2 = 0.1811
Final R indexes [all data]	R_1 = 0.0783, wR_2 = 0.1881
Largest diff. peak/hole / e Å^{-3}	3.84/-1.22
CCDC-number	2501786

Responses to CheckCIF alerts for Ag_3Pz_3·40 crystal structure:

A-level alerts:

"Check Calcd Resid. Dens. 0.99Ang From N00U 3.90 eA-3"

This Alert is due to presence of residual density in the presence of heavy metal atom (Ag).

"Check Calcd Resid. Dens. 1.21Ang From N012 3.88 eA-3"

This Alert is due to presence of residual density in the presence of heavy metal atom (Ag).

B-level alerts:

"Check Calcd Resid. Dens. 1.49Ang From C04P 2.93 eA-3"

This Alert is due to presence of residual density in the presence of heavy metal atom (Ag).

"Check Calcd Resid. Dens. 1.08Ang From C2 2.65 eA-3"

This Alert is due to presence of residual density in the presence of heavy metal atom (Ag).

"Check Calcd Resid. Dens. 1.06Ang From C04X 2.63 eA-3"

This Alert is due to presence of residual density in the presence of heavy metal atom (Ag).

"Check Calcd Resid. Dens. 1.10Ang From C04T 2.56 eA-3"

This Alert is due to presence of residual density in the presence of heavy metal atom (Ag).

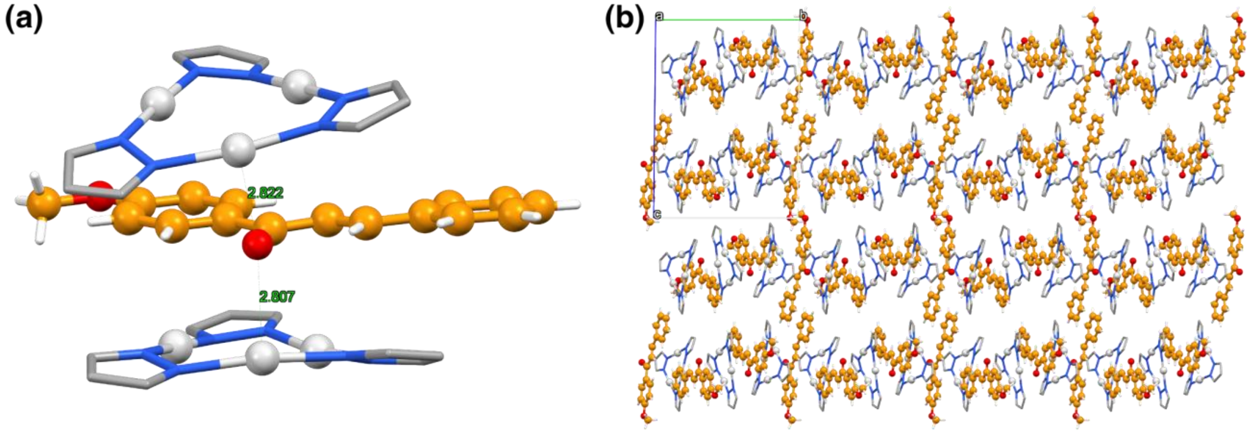

Figure S137. (a) A schematic diagram of the co-crystal structure in the **Ag_3Pz_3·40** single crystal, formed by the guest organic molecule and the surrounding Ag_3Pz_3 units that exhibit significant interactions with it. (b) A 1 × 4 × 2 packing mode in the single crystal structure of **Ag_3Pz_3·40** along the *a* axis. Trifluoromethyl groups and H atoms in Ag_3Pz_3 are omitted for clarity. Ag···O interactions are indicated with green dotted lines with distances in Å. C, N, and Ag atoms in Ag_3Pz_3 are depicted in dark gray, light blue, and light gray, respectively; C, O, and H atoms in **40** are depicted in orange, red, and white, respectively.

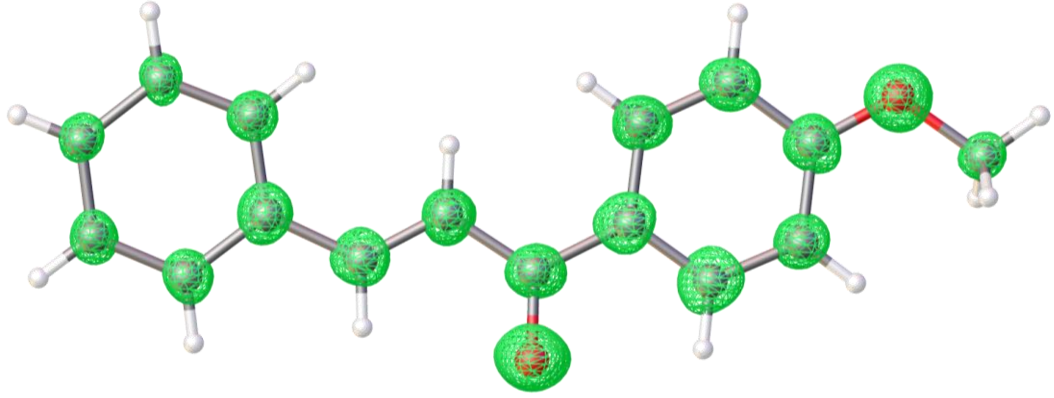

Figure S138. F_{obs} (contour: 0.30) electron density map superimposed on the structure of **40** in the single crystal structure of **Ag_3Pz_3·40**.

Preparation of Ag_3Pz_3·41. 2.55 mg (0.0107 mmol) of 4-methoxychalcone (**41**) was dissolved in 3 mL of c-Hex, followed by the addition of equimolar amounts of Ag_3Pz_3 (10.00 mg, 0.0107 mmol). The resulting mixed solution was filtered and then transferred to a 20 mL screw-capped sample vial. The cap of the sample vial was loosely closed to allow the solvent to slowly evaporate at room temperature. The entire co-crystal incubation process was protected from light using aluminum foil. After the designated evaporation period, typically 1-3 days, high-quality yellow block-shaped crystals suitable for single-crystal X-ray diffraction analysis formed at the bottom of the vial.

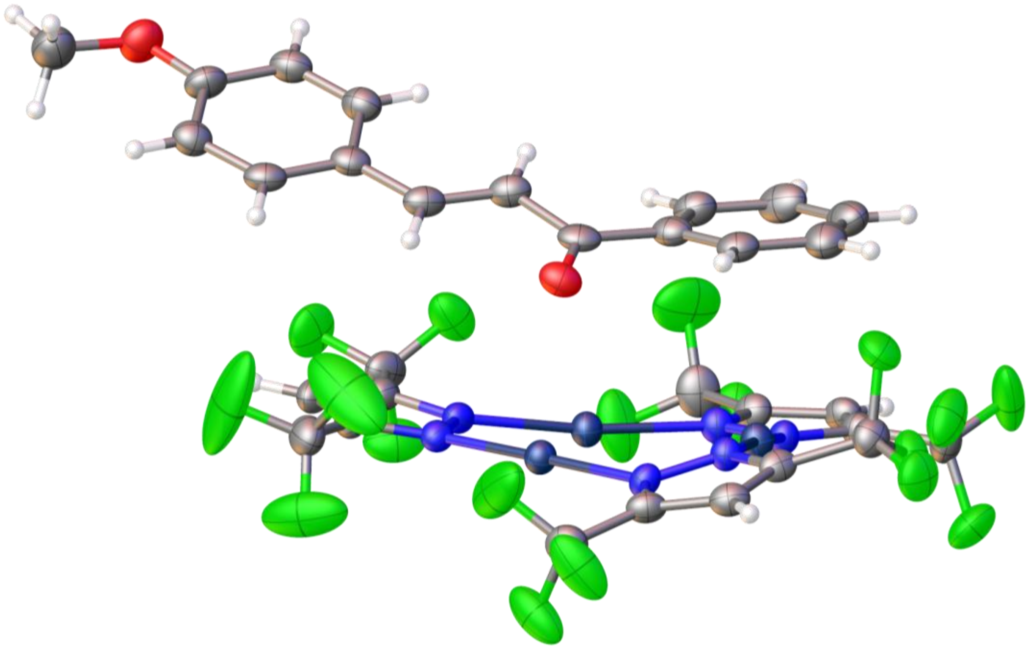

Figure S139. Asymmetric unit of **Ag_3Pz_3·41** (thermal displacement parameters at the 50% probability level).

Table S49. Crystal data and structure refinement for **Ag_3Pz_3·41**

Empirical formula	$C_{31}H_{17}Ag_3F_{18}N_6O_2$
Formula weight	1171.11
Temperature/K	100.00(16)
Crystal system	monoclinic
Space group	$P2_1/n$
a/Å	12.7396(2)
b/Å	12.06380(10)
c/Å	24.1304(2)
α/°	90
β/°	90.0570(10)
γ/°	90
Volume/Å^3	3708.55(7)
Z	4
ρ_{calc}g/cm^3	2.098
μ/mm^{-1}	13.807
F(000)	2256.0
Crystal size/mm^3	0.17 × 0.16 × 0.13
Radiation	Cu Kα (λ = 1.54184)
2θ range for data collection/°	7.326 to 157.012
Index ranges	$-8 \le h \le 15$, $-13 \le k \le 15$, $-30 \le l \le 30$
Reflections collected	28822
Independent reflections	7436 [R_{int} = 0.0376, R_{sigma} = 0.0383]
Data/restraints/parameters	7436/0/542
Goodness-of-fit on F^2	1.128
Final R indexes [I>=2σ (I)]	R_1 = 0.0415, wR_2 = 0.1112
Final R indexes [all data]	R_1 = 0.0490, wR_2 = 0.1147
Largest diff. peak/hole / e Å^{-3}	1.20/-0.93
CCDC-number	2501787

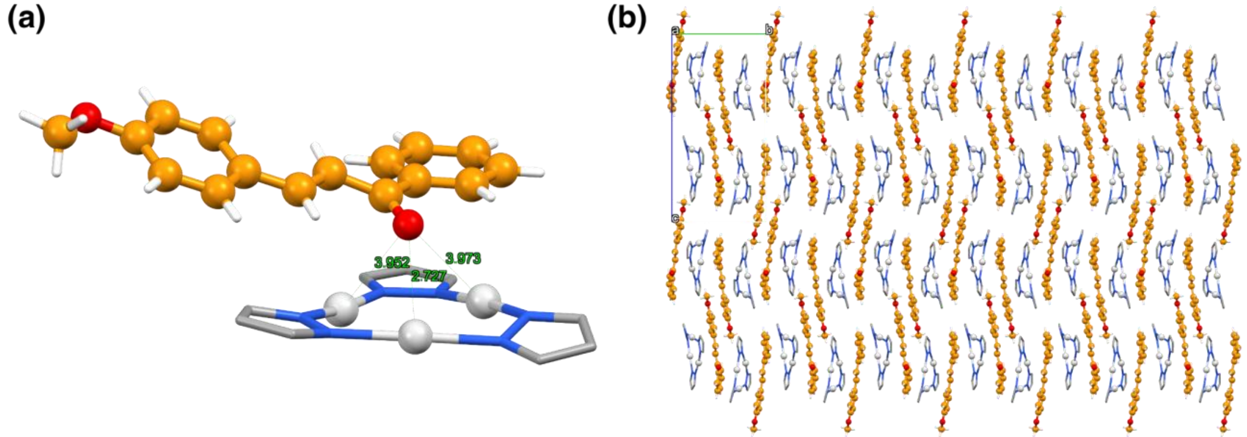

Figure S140. (a) A schematic diagram of the co-crystal structure in the $\mathbf{Ag_3Pz_3}$·**41** single crystal, formed by the guest organic molecule and the surrounding Ag_3Pz_3 units that exhibit significant interactions with it. (b) A 1 × 6 × 2 packing mode in the single crystal structure of $\mathbf{Ag_3Pz_3}$·**41** along the *a* axis. Trifluoromethyl groups and H atoms in Ag_3Pz_3 are omitted for clarity. Ag···O interactions are indicated with green dotted lines with distances in Å. C, N, and Ag atoms in Ag_3Pz_3 are depicted in dark gray, light blue, and light gray, respectively; C, O, and H atoms in **41** are depicted in orange, red, and white, respectively.

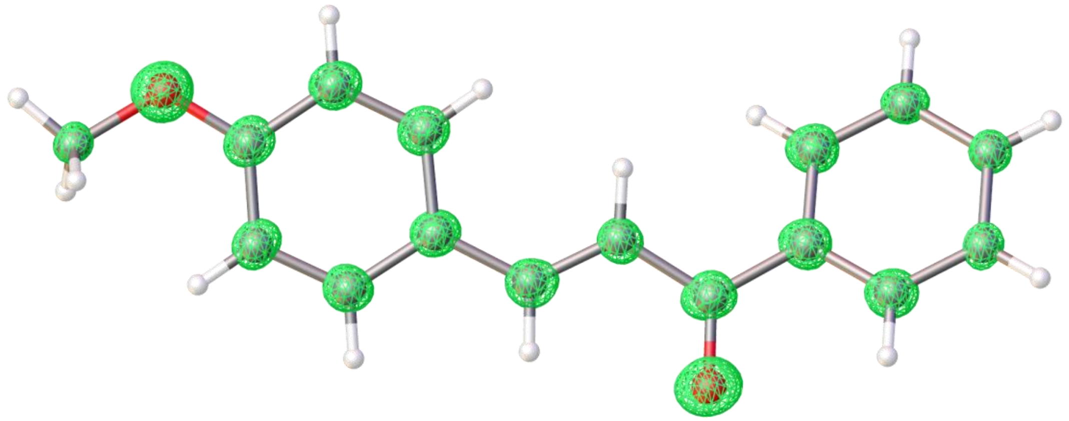

Figure S141. F_{obs} (contour: 0.90) electron density map superimposed on the structure of **41** in the single crystal structure of $\mathbf{Ag_3Pz_3}$·**41**.

Preparation of $\mathbf{Ag_3Pz_3 \cdot 42}$. 2.87 mg (0.0107 mmol) of 4,4'-dimethoxychalcone (**42**) was dissolved in 3 mL of a binary solvent system of DCM and c-Hex (1:1, v/v), followed by the addition of equimolar amounts of Ag_3Pz_3 (10.00 mg, 0.0107 mmol). The resulting mixed solution was filtered and then transferred to a 20 mL screw-capped sample vial. The cap of the sample vial was loosely closed to allow the solvent to slowly evaporate at room temperature. The entire co-crystal incubation process was protected from light using aluminum foil. After the designated evaporation period, typically 1-3 days, high-quality yellow block-shaped crystals suitable for single-crystal X-ray diffraction analysis formed at the bottom of the vial.

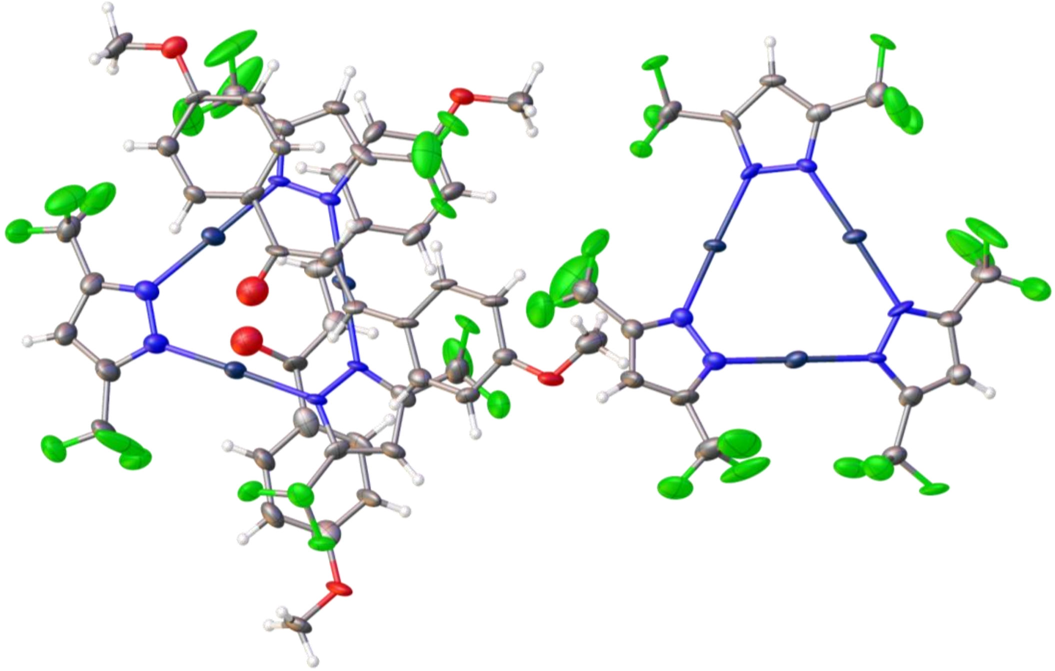

Figure S142. Asymmetric unit of $\mathbf{Ag_3Pz_3 \cdot 42}$ (thermal displacement parameters at the 50% probability level).

Table S50. Crystal data and structure refinement for **Ag_3Pz_3·42**

Empirical formula	$C_{32}H_{19}Ag_3F_{18}N_6O_3$
Formula weight	1201.14
Temperature/K	100.00(17)
Crystal system	monoclinic
Space group	$P2_1/n$
a/Å	14.27860(10)
b/Å	25.1937(3)
c/Å	21.4298(2)
α/°	90
β/°	91.6910(10)
γ/°	90
Volume/Å^3	7705.60(13)
Z	8
ρ_{calc}g/cm^3	2.071
μ/mm^{-1}	13.331
F(000)	4640.0
Crystal size/mm^3	0.28 × 0.26 × 0.126
Radiation	Cu Kα (λ = 1.54184)
2θ range for data collection/°	5.416 to 157.144
Index ranges	$-15 \le h \le 18$, $-31 \le k \le 25$, $-22 \le l \le 26$
Reflections collected	38011
Independent reflections	15699 [R_{int} = 0.0663, R_{sigma} = 0.0791]
Data/restraints/parameters	15699/265/1067
Goodness-of-fit on F^2	1.090
Final R indexes [I>=2σ (I)]	R_1 = 0.1057, wR_2 = 0.2372
Final R indexes [all data]	R_1 = 0.1354, wR_2 = 0.2495
Largest diff. peak/hole / e Å^{-3}	2.00/-1.79
CCDC-number	2501788

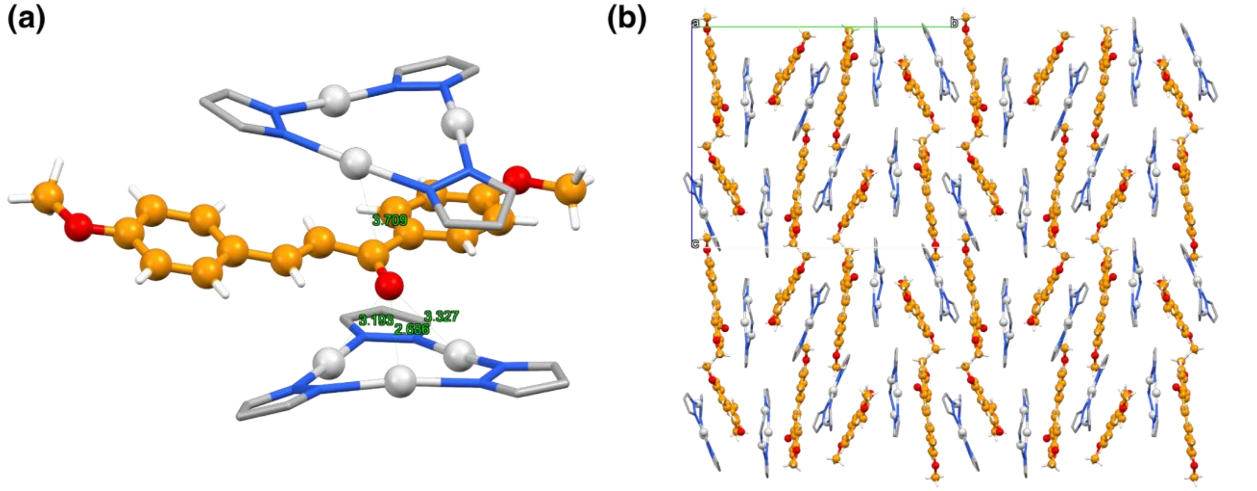

Figure S143. (a) A schematic diagram of the co-crystal structure in the **Ag_3Pz_3·42** single crystal, formed by the guest organic molecule and the surrounding Ag_3Pz_3 units that exhibit significant interactions with it. (b) A 1 × 2 × 2 packing mode in the single crystal structure of **Ag_3Pz_3·42** along the *a* axis. Trifluoromethyl groups and H atoms in Ag_3Pz_3 are omitted for clarity. Ag···O interactions are indicated with green dotted lines with distances in Å. C, N, and Ag atoms in Ag_3Pz_3 are depicted in dark gray, light blue, and light gray, respectively; C, O, and H atoms in **42** are depicted in orange, red, and white, respectively.

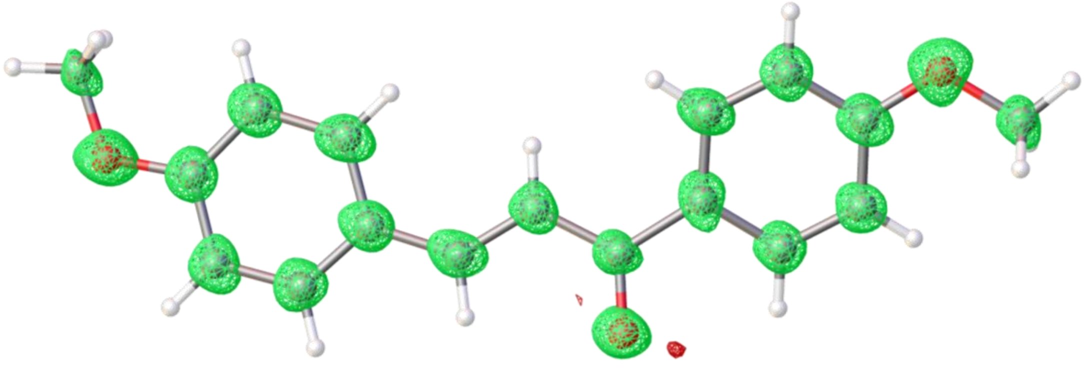

Figure S144. F_{obs} (contour: 0.35) electron density map superimposed on the structure of **42** in the single crystal structure of **Ag_3Pz_3·42**.

Preparation of Ag_3Pz_3·43. 3.36 mg (0.0107 mmol) of flavokawain A (**43**) was dissolved in 3 mL of a binary solvent system of DCM and MeOH (1:1, v/v), followed by the addition of equimolar amounts of Ag_3Pz_3 (10.00 mg, 0.0107 mmol). The resulting mixed solution was filtered and then transferred to a 20 mL screw-capped sample vial. The cap of the sample vial was loosely closed to allow the solvent to slowly evaporate at room temperature. The entire co-crystal incubation process was protected from light using aluminum foil. After the designated evaporation period, typically 1-3 days, high-quality yellow needle-shaped crystals suitable for single-crystal X-ray diffraction analysis formed at the bottom of the vial.

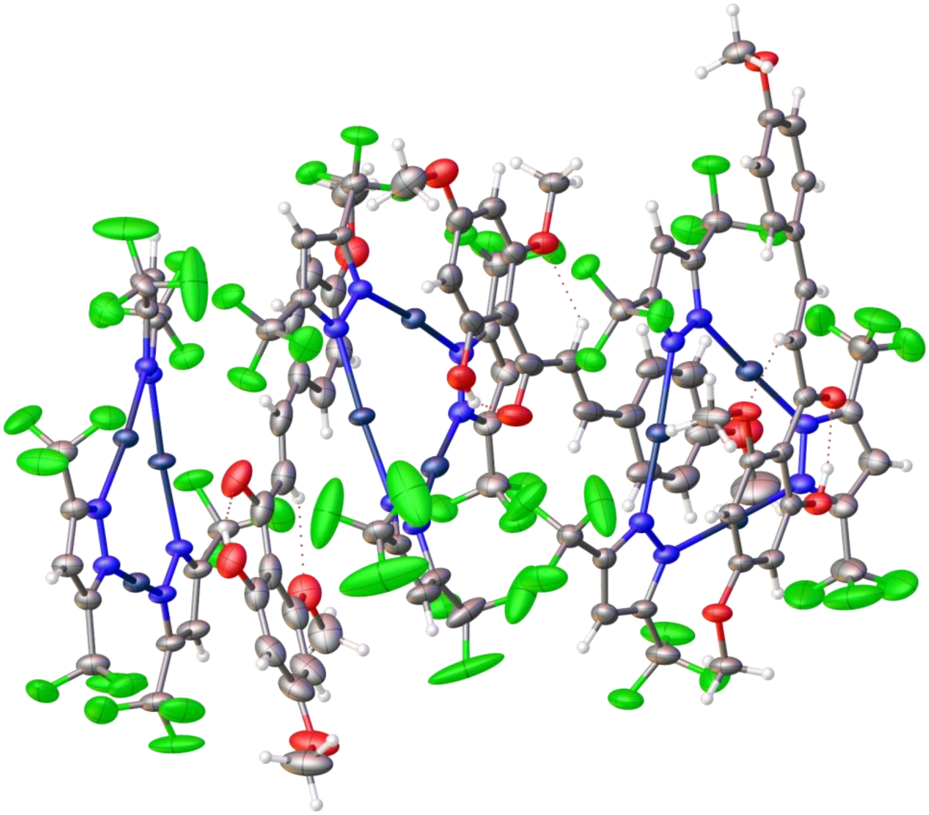

Figure S145. Asymmetric unit of **Ag_3Pz_3·43** (thermal displacement parameters at the 50% probability level).

Table S51. Crystal data and structure refinement for **Ag_3Pz_3·43**

Empirical formula	$C_{99}H_{63}Ag_9F_{54}N_{18}O_{15}$
Formula weight	3741.50
Temperature/K	100.03(11)
Crystal system	triclinic
Space group	$P\bar{1}$
a/Å	16.20520(10)
b/Å	17.7791(2)
c/Å	24.0541(2)
α/°	104.3770(10)
β/°	99.0600(10)
γ/°	111.7620(10)
Volume/Å^3	5990.22(10)
Z	2
ρ_{calc}g/cm^3	2.074
μ/mm^{-1}	12.931
F(000)	3624.0
Crystal size/mm^3	0.12 × 0.12 × 0.1
Radiation	Cu Kα (λ = 1.54184)
2θ range for data collection/°	7.34 to 146.322
Index ranges	$-20 \leq h \leq 19$, $-21 \leq k \leq 21$, $-28 \leq l \leq 29$
Reflections collected	103557
Independent reflections	22504 [R_{int} = 0.0420, R_{sigma} = 0.0227]
Data/restraints/parameters	22504/0/1768
Goodness-of-fit on F^2	1.034
Final R indexes [I>=2σ (I)]	R_1 = 0.0431, wR_2 = 0.1126
Final R indexes [all data]	R_1 = 0.0470, wR_2 = 0.1160
Largest diff. peak/hole / e Å^{-3}	1.40/-1.68
CCDC-number	2501789

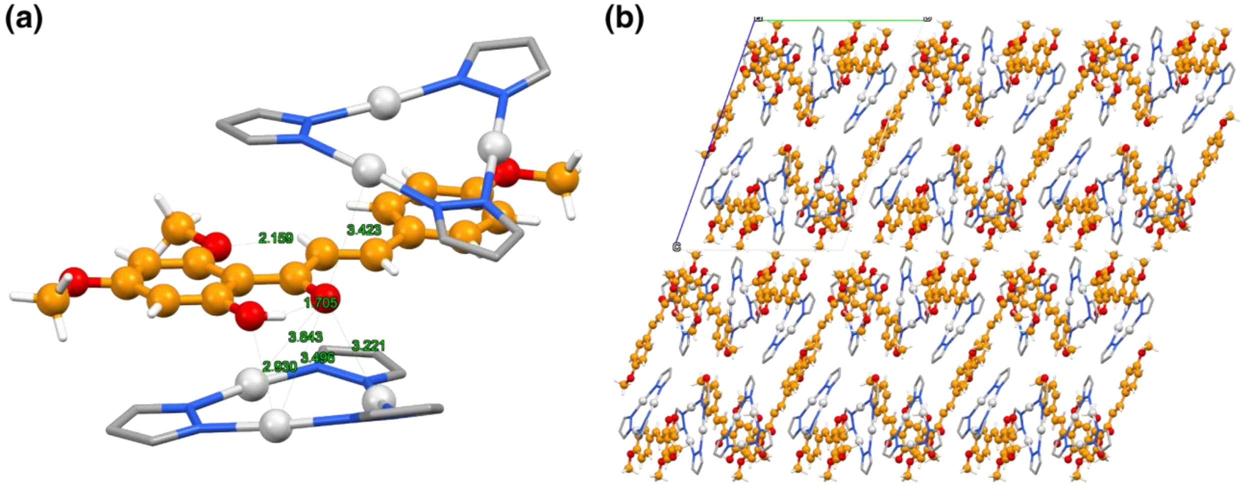

Figure S146. (a) A schematic diagram of the co-crystal structure in the $\mathbf{Ag_3Pz_3 \cdot 43}$ single crystal, formed by the guest organic molecule and the surrounding Ag_3Pz_3 units that exhibit significant interactions with it. (b) A 1 × 3 × 2 packing mode in the single crystal structure of $\mathbf{Ag_3Pz_3 \cdot 43}$ along the *a* axis. Trifluoromethyl groups and H atoms in Ag_3Pz_3 are omitted for clarity. Ag···O and O-H···O interactions are indicated with green dotted lines with distances in Å. C, N, and Ag atoms in Ag_3Pz_3 are depicted in dark gray, light blue, and light gray, respectively; C, O, and H atoms in **43** are depicted in orange, red, and white, respectively.

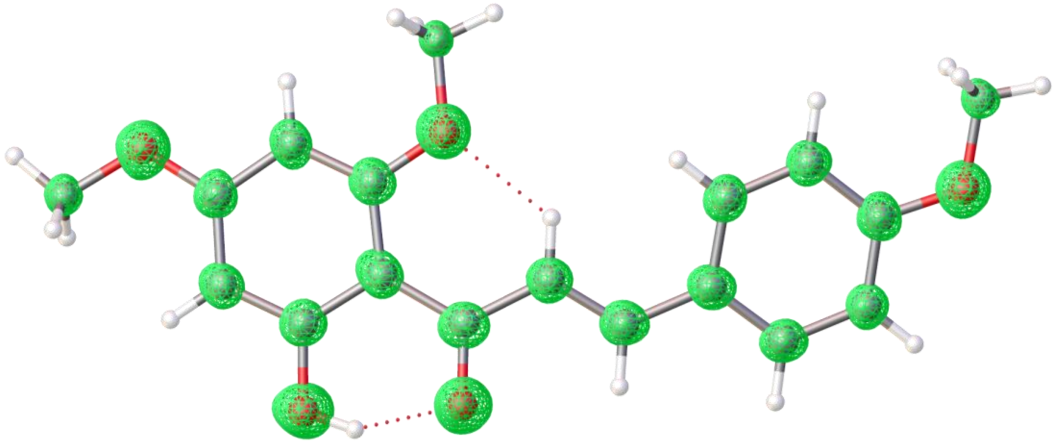

Figure S147. F_{obs} (contour: 0.55) electron density map superimposed on the structure of **43** in the single crystal structure of $\mathbf{Ag_3Pz_3 \cdot 43}$.

Preparation of Ag_3Pz_3·44. 2.40 mg (0.0107 mmol) of dibenzoylmethane (**44**) was dissolved in 3 mL of n-Hex, followed by the addition of equimolar amounts of Ag_3Pz_3 (10.00 mg, 0.0107 mmol). The resulting mixed solution was filtered and then transferred to a 20 mL screw-capped sample vial. The cap of the sample vial was loosely closed to allow the solvent to slowly evaporate at room temperature. The entire co-crystal incubation process was protected from light using aluminum foil. After the designated evaporation period, typically 1-3 days, high-quality colorless needle-shaped crystals suitable for single-crystal X-ray diffraction analysis formed at the bottom of the vial.

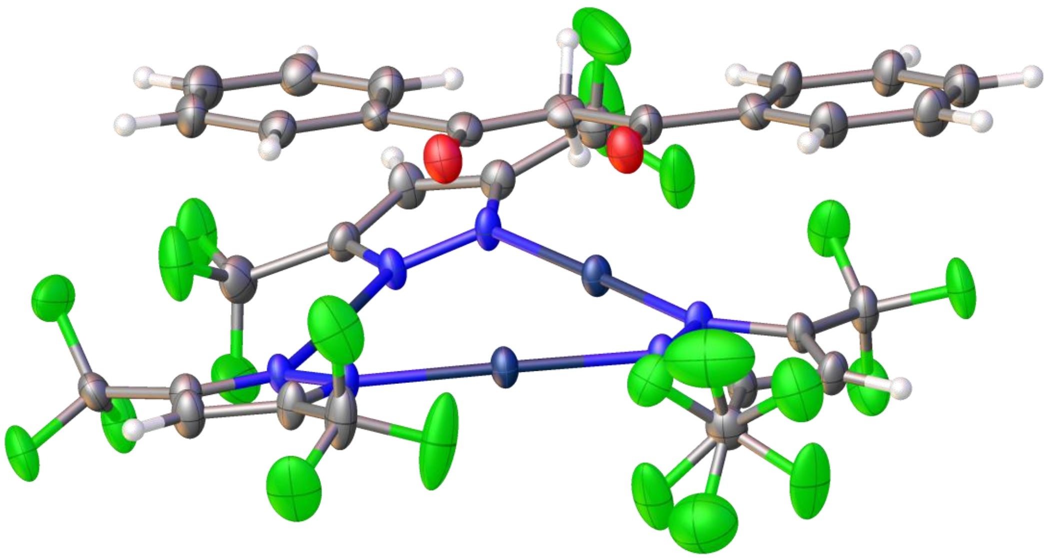

Figure S148. Asymmetric unit of **Ag_3Pz_3·44** (thermal displacement parameters at the 50% probability level).

Table S52. Crystal data and structure refinement for **Ag_3Pz_3·44**

Empirical formula	$C_{30}H_{15}Ag_3F_{18}N_6O_2$
Formula weight	1157.09
Temperature/K	100.00(10)
Crystal system	monoclinic
Space group	$P2_1/c$
a/Å	12.80990(10)
b/Å	22.8396(2)
c/Å	13.17360(10)
α/°	90
β/°	114.9480(10)
γ/°	90
Volume/Å^3	3494.60(6)
Z	4
ρ_{calc}g/cm^3	2.199
μ/mm^{-1}	14.642
F(000)	2224.0
Crystal size/mm^3	0.3 × 0.2 × 0.1
Radiation	Cu Kα (λ = 1.54184)
2θ range for data collection/°	7.612 to 156.912
Index ranges	$-16 \leq h \leq 12$, $-23 \leq k \leq 28$, $-12 \leq l \leq 16$
Reflections collected	17946
Independent reflections	7211 [R_{int} = 0.0377, R_{sigma} = 0.0440]
Data/restraints/parameters	7211/45/560
Goodness-of-fit on F^2	1.083
Final R indexes [I>=2σ (I)]	R_1 = 0.0519, wR_2 = 0.1346
Final R indexes [all data]	R_1 = 0.0643, wR_2 = 0.1386
Largest diff. peak/hole / e Å^{-3}	1.22/-0.88
CCDC-number	2501790

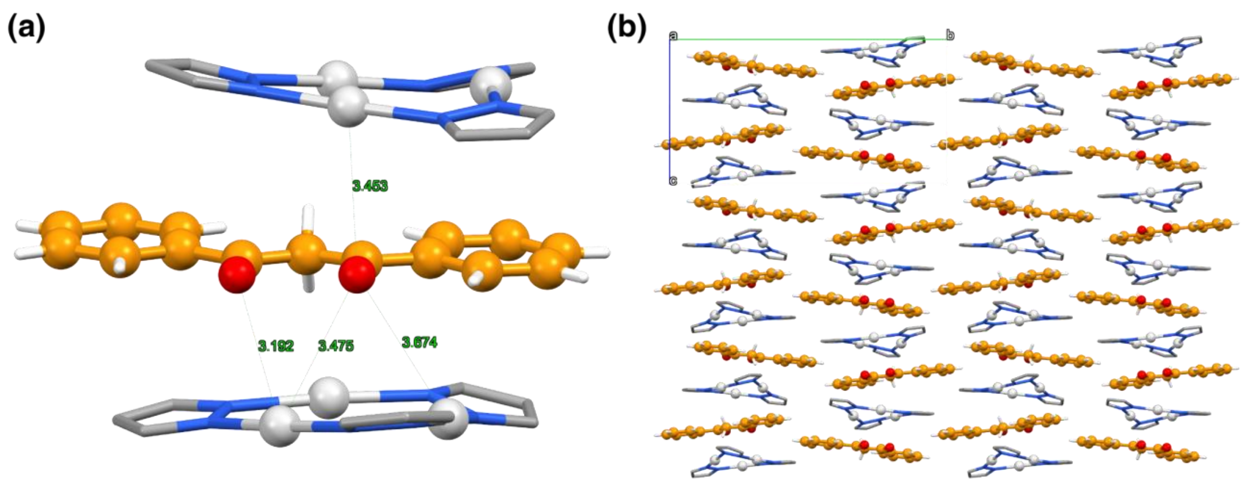

Figure S149. (a) A schematic diagram of the co-crystal structure in the **Ag_3Pz_3·44** single crystal, formed by the guest organic molecule and the surrounding Ag_3Pz_3 units that exhibit significant interactions with it. (b) A 1 × 2 × 3 packing mode in the single crystal structure of **Ag_3Pz_3·44** along the *a* axis. Trifluoromethyl groups and H atoms in Ag_3Pz_3 are omitted for clarity. Ag···O interactions are indicated with green dotted lines with distances in Å. C, N, and Ag atoms in Ag_3Pz_3 are depicted in dark gray, light blue, and light gray, respectively; C, O, and H atoms in **44** are depicted in orange, red, and white, respectively.

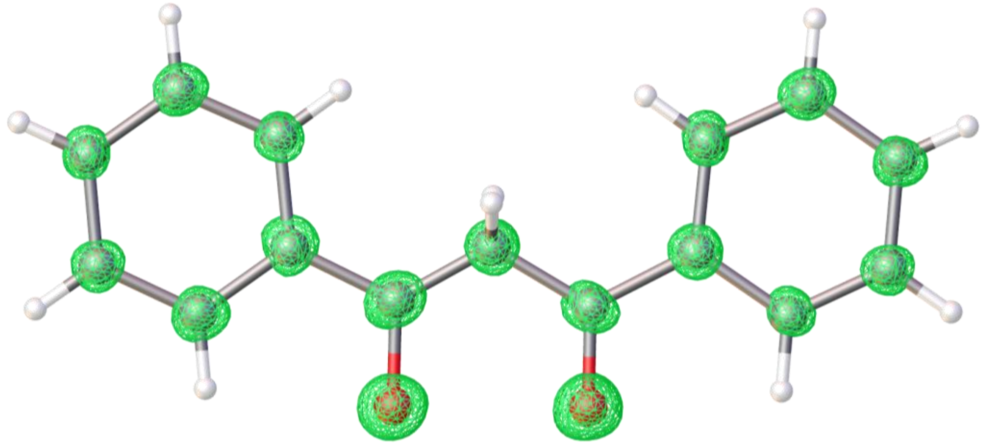

Figure S150. F_{obs} (contour: 0.50) electron density map superimposed on the structure of **44** in the single crystal structure of **Ag_3Pz_3·44**.

Preparation of Ag_3Pz_3·45. 2.57 mg (0.0107 mmol) of 1-(2-hydroxyphenyl)-3-phenyl-1,3-propanedione (**45**) was dissolved in 3 mL of a binary solvent system of DCM and n-Hex (1:1, v/v), followed by the addition of equimolar amounts of Ag_3Pz_3 (10.00 mg, 0.0107 mmol). The resulting mixed solution was filtered and then transferred to a 20 mL screw-capped sample vial. The cap of the sample vial was loosely closed to allow the solvent to slowly evaporate at room temperature. The entire co-crystal incubation process was protected from light using aluminum foil. After the designated evaporation period, typically 1-3 days, high-quality yellow needle-shaped crystals suitable for single-crystal X-ray diffraction analysis formed at the bottom of the vial.

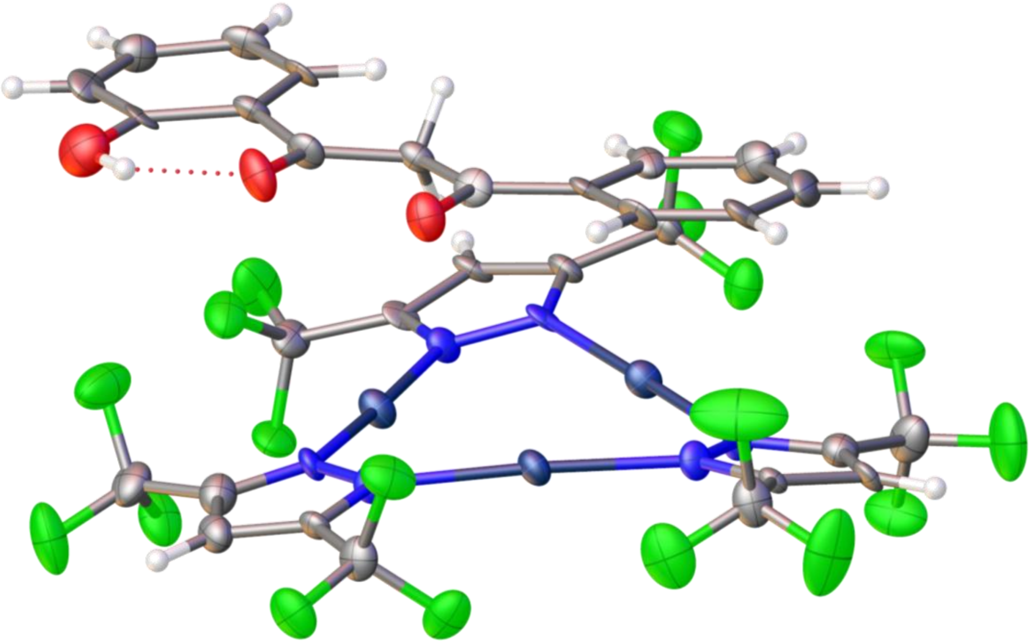

Figure S151. Asymmetric unit of **Ag_3Pz_3·45** (thermal displacement parameters at the 50% probability level).

Table S53. Crystal data and structure refinement for $\mathbf{Ag_3Pz_3{\cdot}45}$

Empirical formula	$C_{30}H_{15}Ag_3F_{18}N_6O_3$
Formula weight	1173.09
Temperature/K	100.01(17)
Crystal system	monoclinic
Space group	$P2_1/c$
a/Å	8.23110(10)
b/Å	20.4500(2)
c/Å	21.1854(2)
α/°	90
β/°	98.5390(10)
γ/°	90
Volume/Å^3	3526.52(6)
Z	4
ρ_{calc}g/cm^3	2.209
μ/mm^{-1}	14.544
F(000)	2256.0
Crystal size/mm^3	0.24 × 0.23 × 0.21
Radiation	Cu Kα (λ = 1.54184)
2θ range for data collection/°	6.04 to 155.86
Index ranges	$-10 \leq h \leq 9$, $-23 \leq k \leq 25$, $-26 \leq l \leq 20$
Reflections collected	17951
Independent reflections	7198 [R_{int} = 0.0934, R_{sigma} = 0.0935]
Data/restraints/parameters	7198/92/491
Goodness-of-fit on F^2	1.172
Final R indexes [I>=2σ (I)]	R_1 = 0.1155, wR_2 = 0.2953
Final R indexes [all data]	R_1 = 0.1415, wR_2 = 0.3048
Largest diff. peak/hole / e Å^{-3}	2.93/-1.72
CCDC-number	2501791

Responses to CheckCIF alerts for Ag_3Pz_3·45 crystal structure:

(There is no A-level alert)

B-level alerts:

"Check Calcd Resid. Dens. 0.88Ang From Ag03 3.13 eA-3"

This Alert is due to presence of residual density in the presence of heavy metal atom (Ag).

"Check Calcd Resid. Dens. 0.83Ang From Ag03 2.82 eA-3"

This Alert is due to presence of residual density in the presence of heavy metal atom (Ag).

"Check Calcd Resid. Dens. 0.96Ang From Ag01 2.67 eA-3"

This Alert is due to presence of residual density in the presence of heavy metal atom (Ag).

"Check Calcd Resid. Dens. 0.83Ang From Ag02 2.59 eA-3"

This Alert is due to presence of residual density in the presence of heavy metal atom (Ag).

"Check Calcd Resid. Dens. 0.95Ang From Ag01 2.52 eA-3"

This Alert is due to presence of residual density in the presence of heavy metal atom (Ag).

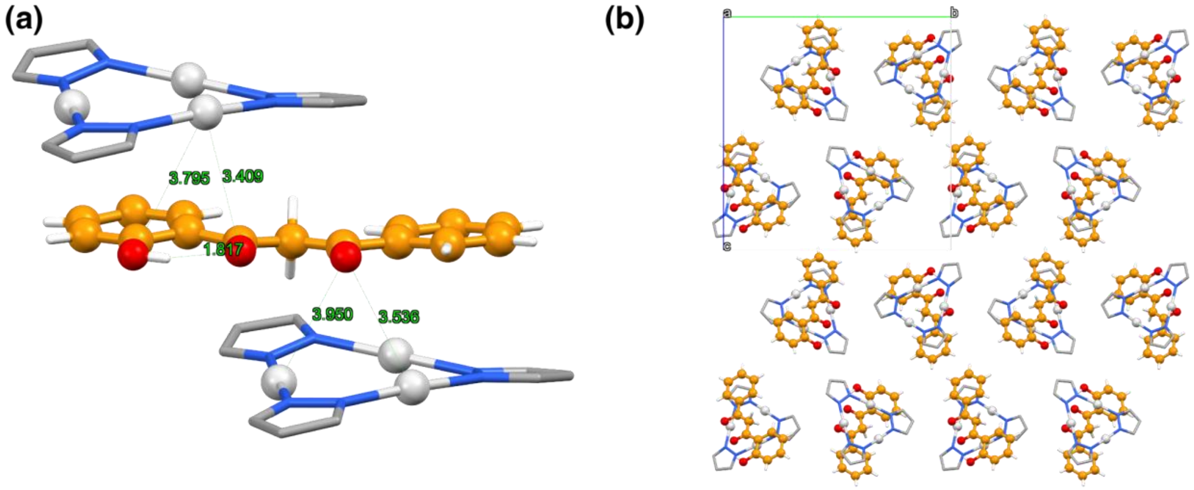

Figure S152. (a) A schematic diagram of the co-crystal structure in the **Ag_3Pz_3·45** single crystal, formed by the guest organic molecule and the surrounding Ag_3Pz_3 units that exhibit significant interactions with it. (b) A 1 × 2 × 2 packing mode in the single crystal structure of **Ag_3Pz_3·45** along the *a* axis. Trifluoromethyl groups and H atoms in Ag_3Pz_3 are omitted for clarity. Ag···O and O-H···O interactions are indicated with green dotted lines with distances in Å. C, N, and Ag atoms in Ag_3Pz_3 are depicted in dark gray, light blue, and light gray, respectively; C, O, and H atoms in **45** are depicted in orange, red, and white, respectively.

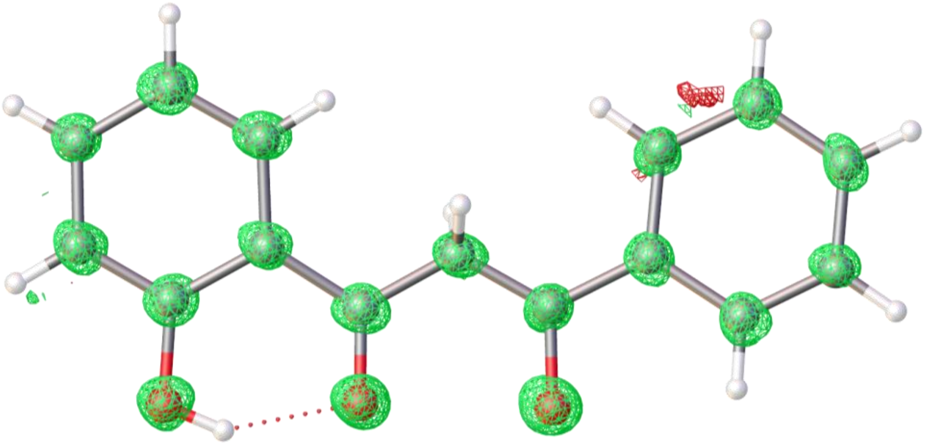

Figure S153. F_{obs} (contour: 1.10) electron density map superimposed on the structure of **45** in the single crystal structure of **Ag_3Pz_3·45**.

Preparation of Ag_3Pz_3·46. 2.19 mg (0.0107 mmol) of 1-(2-mesitylene)-1,3-butanedione (**46**) was dissolved in 3 mL of a binary solvent system of DCM and MeOH (1:1, v/v), followed by the addition of equimolar amounts of Ag_3Pz_3 (10.00 mg, 0.0107 mmol). The resulting mixed solution was filtered and then transferred to a 20 mL screw-capped sample vial. The cap of the sample vial was loosely closed to allow the solvent to slowly evaporate at room temperature. The entire co-crystal incubation process was protected from light using aluminum foil. After the designated evaporation period, typically 1-3 days, high-quality colorless block-shaped crystals suitable for single-crystal X-ray diffraction analysis formed at the bottom of the vial.

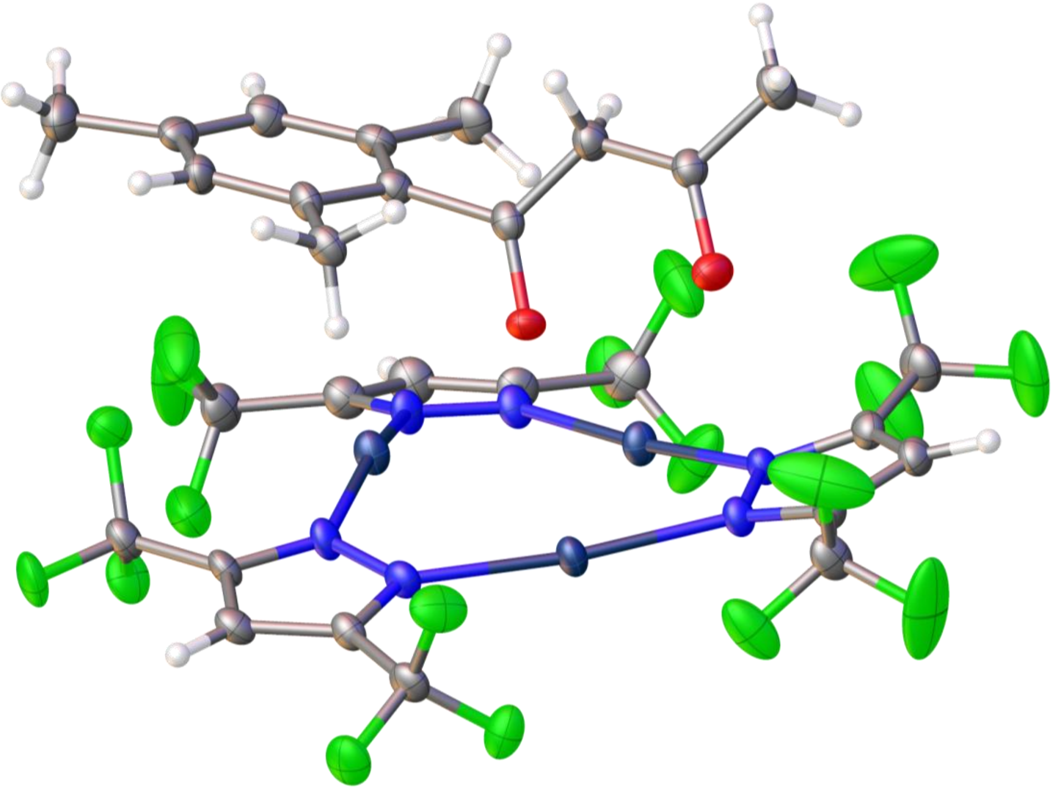

Figure S154. Asymmetric unit of **Ag_3Pz_3·46** (thermal displacement parameters at the 50% probability level).

Table S54. Crystal data and structure refinement for **Ag_3Pz_3·46**

Empirical formula	$C_{28}H_{19}Ag_3F_{18}N_6O_2$
Formula weight	1137.10
Temperature/K	100.2(5)
Crystal system	triclinic
Space group	$P\bar{1}$
a/Å	8.1897(2)
b/Å	12.9426(3)
c/Å	17.1971(4)
α/°	90.273(2)
β/°	97.670(2)
γ/°	103.305(2)
Volume/Å^3	1756.84(7)
Z	2
ρ_{calc}g/cm^3	2.150
μ/mm^{-1}	14.542
F(000)	1096.0
Crystal size/mm^3	0.23 × 0.16 × 0.13
Radiation	Cu Kα (λ = 1.54184)
2θ range for data collection/°	7.024 to 157.184
Index ranges	$-10 \le h \le 10$, $-15 \le k \le 16$, $-21 \le l \le 10$
Reflections collected	16998
Independent reflections	7198 [R_{int} = 0.0324, R_{sigma} = 0.0372]
Data/restraints/parameters	7198/0/518
Goodness-of-fit on F^2	1.056
Final R indexes [I>=2σ (I)]	R_1 = 0.0437, wR_2 = 0.1189
Final R indexes [all data]	R_1 = 0.0483, wR_2 = 0.1206
Largest diff. peak/hole / e Å^{-3}	1.14/-1.12
CCDC-number	2501792

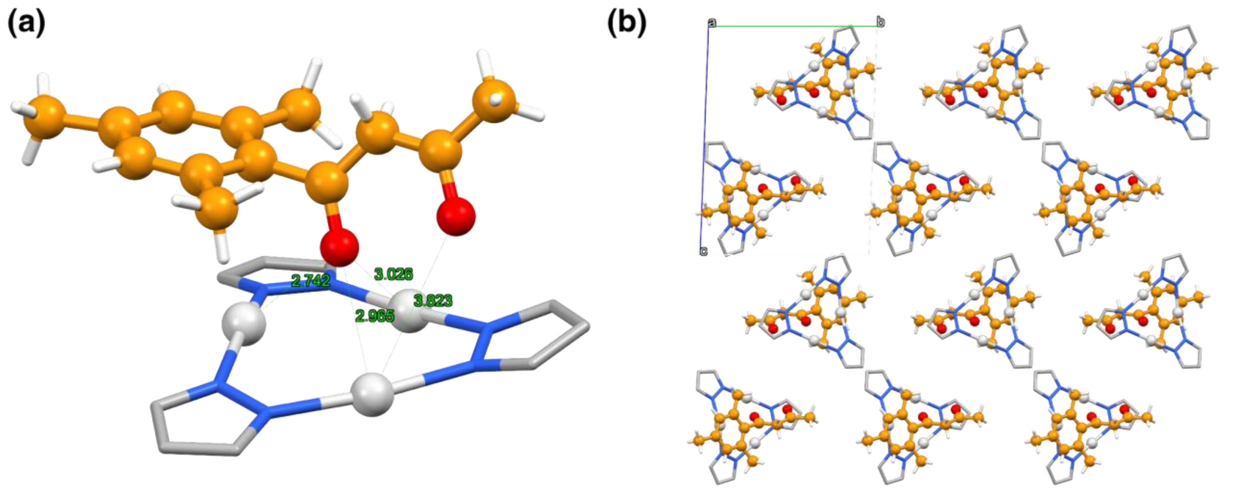

Figure S155. (a) A schematic diagram of the co-crystal structure in the $\mathbf{Ag_3Pz_3{\cdot}46}$ single crystal, formed by the guest organic molecule and the surrounding Ag_3Pz_3 units that exhibit significant interactions with it. (b) A 1 × 3 × 2 packing mode in the single crystal structure of $\mathbf{Ag_3Pz_3{\cdot}46}$ along the *a* axis. Trifluoromethyl groups and H atoms in Ag_3Pz_3 are omitted for clarity. Ag···O interactions are indicated with green dotted lines with distances in Å. C, N, and Ag atoms in Ag_3Pz_3 are depicted in dark gray, light blue, and light gray, respectively; C, O, and H atoms in **46** are depicted in orange, red, and white, respectively.

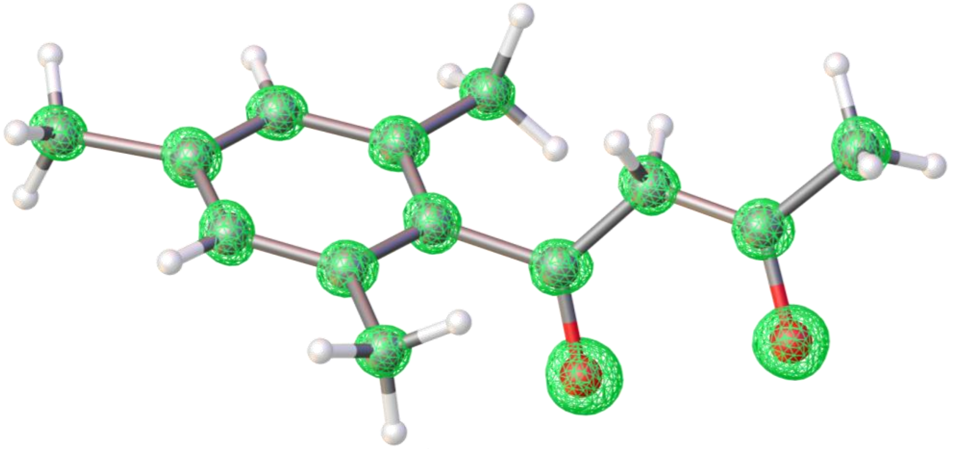

Figure S156. F_{obs} (contour: 2.00) electron density map superimposed on the structure of **46** in the single crystal structure of $\mathbf{Ag_3Pz_3{\cdot}46}$.

Preparation of Ag_3Pz_3·47. 2.59 mg (0.0107 mmol) of 4,4'-dimethoxybenzophenone (**47**) was dissolved in 3 mL of a binary solvent system of n-Hex and c-Hex (1:1, v/v), followed by the addition of equimolar amounts of Ag_3Pz_3 (10.00 mg, 0.0107 mmol). The resulting mixed solution was filtered and then transferred to a 20 mL screw-capped sample vial. The cap of the sample vial was loosely closed to allow the solvent to slowly evaporate at room temperature. The entire co-crystal incubation process was protected from light using aluminum foil. After the designated evaporation period, typically 1-3 days, high-quality colorless block-shaped crystals suitable for single-crystal X-ray diffraction analysis formed at the bottom of the vial.

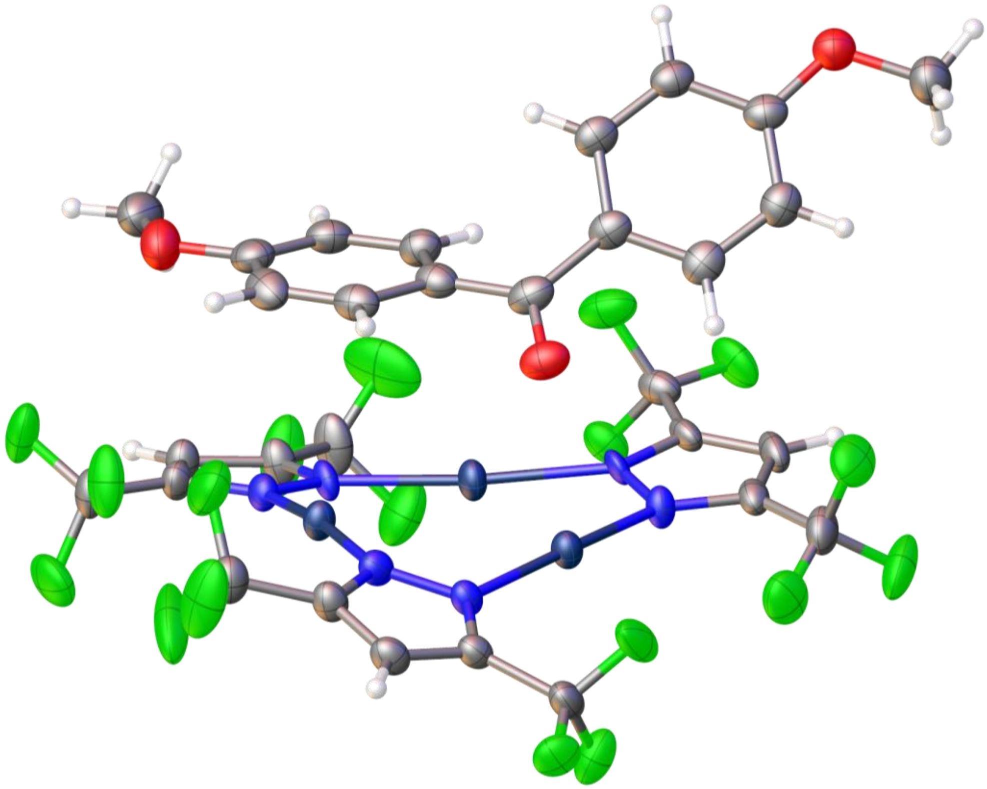

Figure S157. Asymmetric unit of **Ag_3Pz_3·47** (thermal displacement parameters at the 50% probability level).

Table S55. Crystal data and structure refinement for **Ag_3Pz_3·47**

Empirical formula	$C_{30}H_{17}Ag_3F_{18}N_6O_3$
Formula weight	1175.10
Temperature/K	100.00(10)
Crystal system	monoclinic
Space group	$P2_1/n$
a/Å	11.8873(3)
b/Å	12.7795(5)
c/Å	23.8506(6)
α/°	90
β/°	91.767(3)
γ/°	90
Volume/Å^3	3621.51(19)
Z	4
ρ_{calc}g/cm^3	2.155
μ/mm^{-1}	14.162
F(000)	2264.0
Crystal size/mm^3	0.2 × 0.2 × 0.2
Radiation	Cu Kα (λ = 1.54184)
2θ range for data collection/°	7.416 to 156.632
Index ranges	$-14 \leq h \leq 15$, $-15 \leq k \leq 9$, $-28 \leq l \leq 30$
Reflections collected	19989
Independent reflections	7395 [R_{int} = 0.0441, R_{sigma} = 0.0398]
Data/restraints/parameters	7395/0/543
Goodness-of-fit on F^2	1.089
Final R indexes [I>=2σ (I)]	R_1 = 0.0637, wR_2 = 0.1808
Final R indexes [all data]	R_1 = 0.0726, wR_2 = 0.1861
Largest diff. peak/hole / e Å^{-3}	1.81/-1.63
CCDC-number	2501793

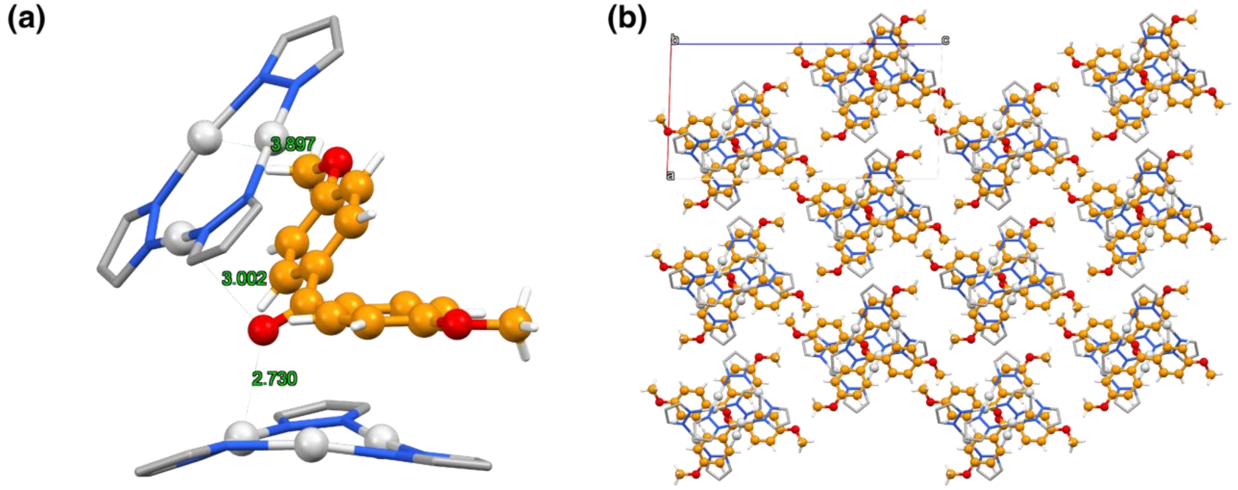

Figure S158. (a) A schematic diagram of the co-crystal structure in the **Ag_3Pz_3·47** single crystal, formed by the guest organic molecule and the surrounding Ag_3Pz_3 units that exhibit significant interactions with it. (b) A 3 × 1 × 2 packing mode in the single crystal structure of **Ag_3Pz_3·47** along the *b* axis. Trifluoromethyl groups and H atoms in Ag_3Pz_3 are omitted for clarity. Ag···O interactions are indicated with green dotted lines with distances in Å. C, N, and Ag atoms in Ag_3Pz_3 are depicted in dark gray, light blue, and light gray, respectively; C, O, and H atoms in **47** are depicted in orange, red, and white, respectively.

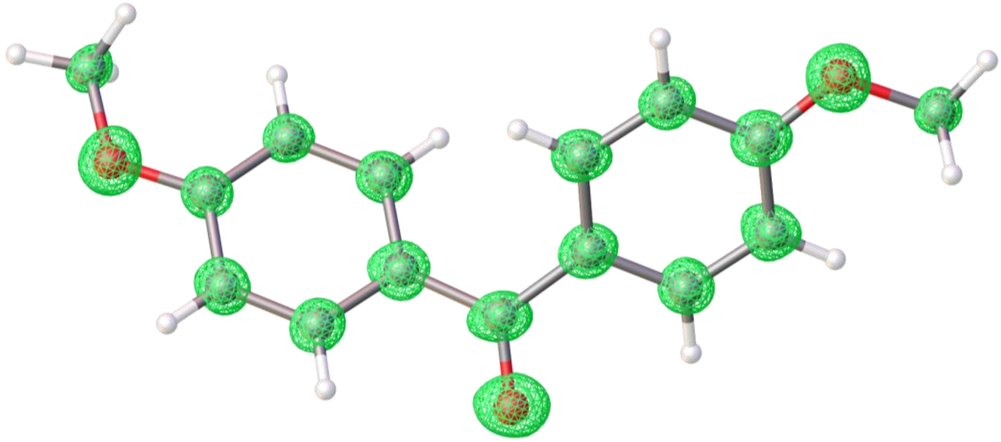

Figure S159. F_{obs} (contour: 0.85) electron density map superimposed on the structure of **47** in the single crystal structure of **Ag_3Pz_3·47**.

Preparation of Ag_3Pz_3·48. 2.44 mg (0.0107 mmol) of oxybenzone (**48**) was dissolved in 3 mL of a binary solvent system of n-Hex and c-Hex (1:1, v/v), followed by the addition of equimolar amounts of Ag_3Pz_3 (10.00 mg, 0.0107 mmol). The resulting mixed solution was filtered and then transferred to a 20 mL screw-capped sample vial. The cap of the sample vial was loosely closed to allow the solvent to slowly evaporate at room temperature. The entire co-crystal incubation process was protected from light using aluminum foil. After the designated evaporation period, typically 1-3 days, high-quality colorless block-shaped crystals suitable for single-crystal X-ray diffraction analysis formed at the bottom of the vial.

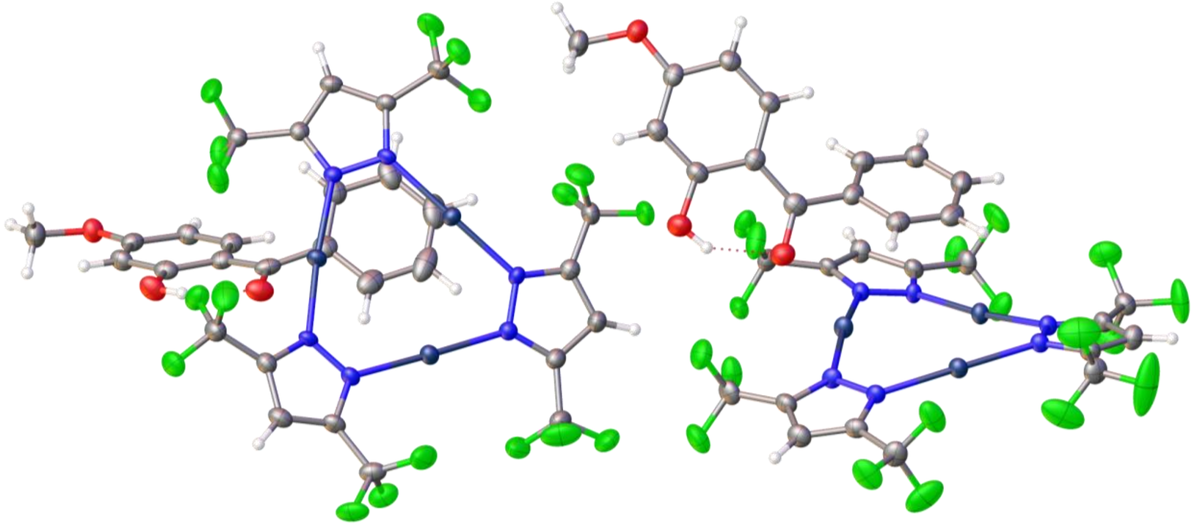

Figure S160. Asymmetric unit of **Ag_3Pz_3·48** (thermal displacement parameters at the 50% probability level).

Table S56. Crystal data and structure refinement for **Ag_3Pz_3·48**

Empirical formula	$C_{29}H_{15}Ag_3F_{18}N_6O_3$
Formula weight	1161.08
Temperature/K	99.99(13)
Crystal system	monoclinic
Space group	$P2_1/c$
a/Å	24.4595(4)
b/Å	12.9956(2)
c/Å	25.5356(4)
α/°	90
β/°	117.487(2)
γ/°	90
Volume/Å^3	7200.6(2)
Z	8
ρ_{calc}g/cm^3	2.142
μ/mm^{-1}	14.236
F(000)	4464.0
Crystal size/mm^3	0.3 × 0.2 × 0.1
Radiation	Cu Kα (λ = 1.54184)
2θ range for data collection/°	6.94 to 156.378
Index ranges	$-30 \leq h \leq 30$, $-15 \leq k \leq 10$, $-32 \leq l \leq 32$
Reflections collected	43186
Independent reflections	14788 [R_{int} = 0.0345, R_{sigma} = 0.0360]
Data/restraints/parameters	14788/0/1067
Goodness-of-fit on F^2	1.085
Final R indexes [I>=2σ (I)]	R_1 = 0.0414, wR_2 = 0.1156
Final R indexes [all data]	R_1 = 0.0474, wR$_2$ = 0.1193
Largest diff. peak/hole / e Å^{-3}	0.93/-1.18
CCDC-number	2501794

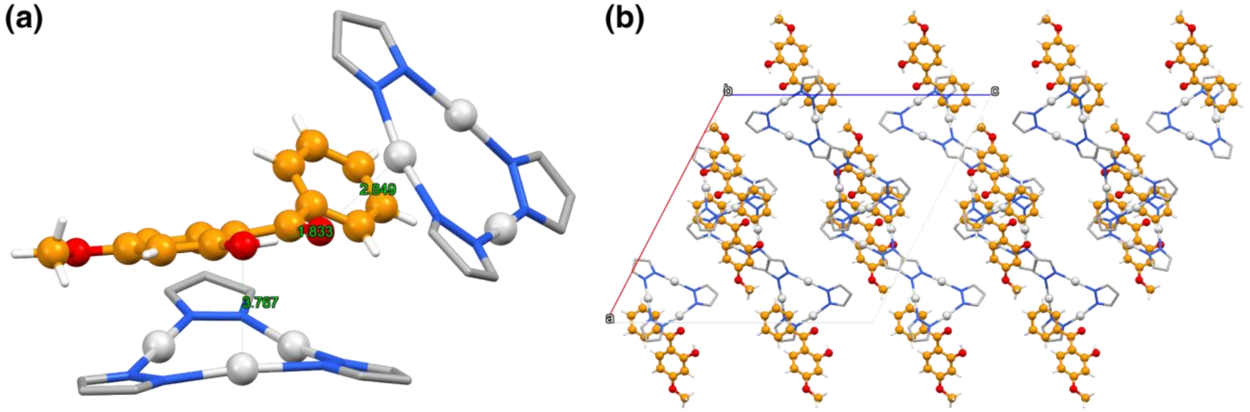

Figure S161. (a) A schematic diagram of the co-crystal structure in the $\mathbf{Ag_3Pz_3{\cdot}48}$ single crystal, formed by the guest organic molecule and the surrounding Ag_3Pz_3 units that exhibit significant interactions with it. (b) A 1 × 1 × 2 packing mode in the single crystal structure of $\mathbf{Ag_3Pz_3{\cdot}48}$ along the *b* axis. Trifluoromethyl groups and H atoms in Ag_3Pz_3 are omitted for clarity. Ag···O and O-H···O interactions are indicated with green dotted lines with distances in Å. C, N, and Ag atoms in Ag_3Pz_3 are depicted in dark gray, light blue, and light gray, respectively; C, O, and H atoms in **48** are depicted in orange, red, and white, respectively.

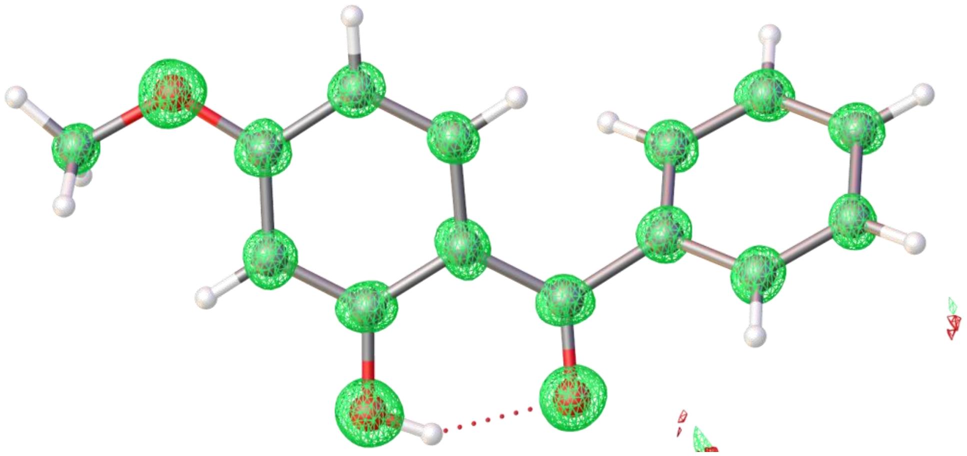

Figure S162. F_{obs} (contour: 0.50) electron density map superimposed on the structure of **48** in the single crystal structure of $\mathbf{Ag_3Pz_3{\cdot}48}$.

Preparation of Ag_3Pz_3·49. 1.69 mg (0.0107 mmol) of 1,4-naphthoquinone (**49**) was dissolved in 3 mL of n-Hex, followed by the addition of equimolar amounts of Ag_3Pz_3 (10.00 mg, 0.0107 mmol). The resulting mixed solution was filtered and then transferred to a 20 mL screw-capped sample vial. The cap of the sample vial was loosely closed to allow the solvent to slowly evaporate at room temperature. The entire co-crystal incubation process was protected from light using aluminum foil. After the designated evaporation period, typically 1-3 days, high-quality yellow needle-shaped crystals suitable for single-crystal X-ray diffraction analysis formed at the bottom of the vial.

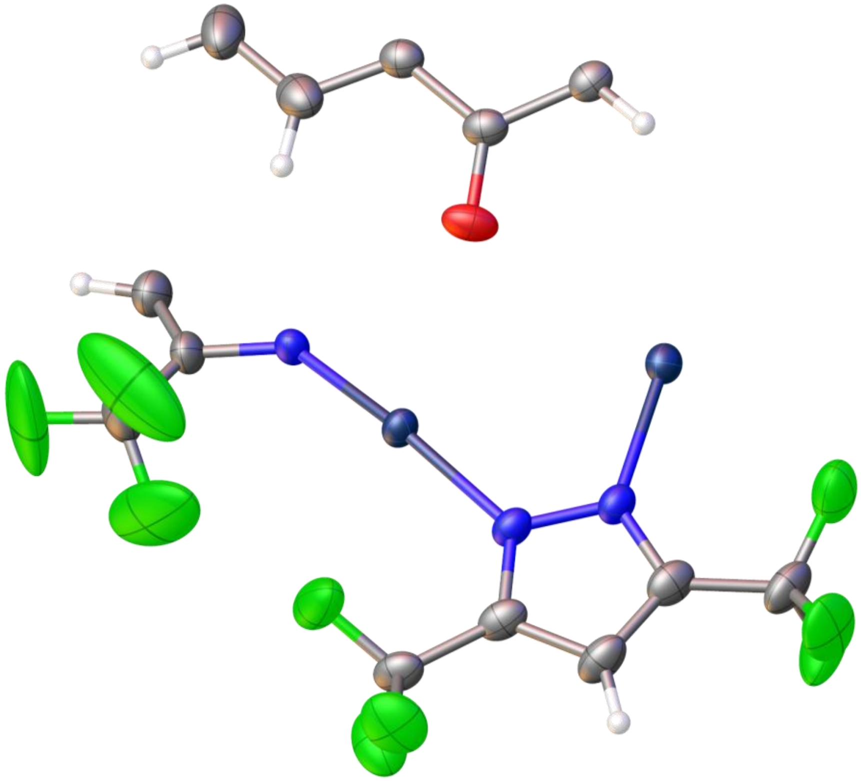

Figure S163. Asymmetric unit of **Ag_3Pz_3·49** (thermal displacement parameters at the 50% probability level).

Table S57. Crystal data and structure refinement for **Ag_3Pz_3·49**

Empirical formula	$C_{12.5}H_{4.5}Ag_{1.5}F_9N_3O$
Formula weight	545.50
Temperature/K	99.99(18)
Crystal system	monoclinic
Space group	*C*2/*c*
a/Å	8.56980(10)
b/Å	24.9122(3)
c/Å	14.9925(2)
α/°	90
β/°	101.8140(10)
γ/°	90
Volume/Å^3	3132.99(7)
Z	8
ρ_{calc}g/cm^3	2.313
μ/mm^{-1}	16.274
F(000)	2080.0
Crystal size/mm^3	0.16 × 0.14 × 0.13
Radiation	Cu Kα (λ = 1.54184)
2θ range for data collection/°	7.096 to 156.6
Index ranges	$-10 \leq h \leq 10$, $-19 \leq k \leq 30$, $-19 \leq l \leq 17$
Reflections collected	10103
Independent reflections	3240 [R_{int} = 0.0331, R_{sigma} = 0.0302]
Data/restraints/parameters	3240/18/245
Goodness-of-fit on F^2	1.107
Final *R* indexes [I>=2σ (I)]	R_1 = 0.0461, wR_2 = 0.1239
Final *R* indexes [all data]	R_1 = 0.0507, wR_2 = 0.1258
Largest diff. peak/hole / e Å^{-3}	1.24/-1.36
CCDC-number	2501795

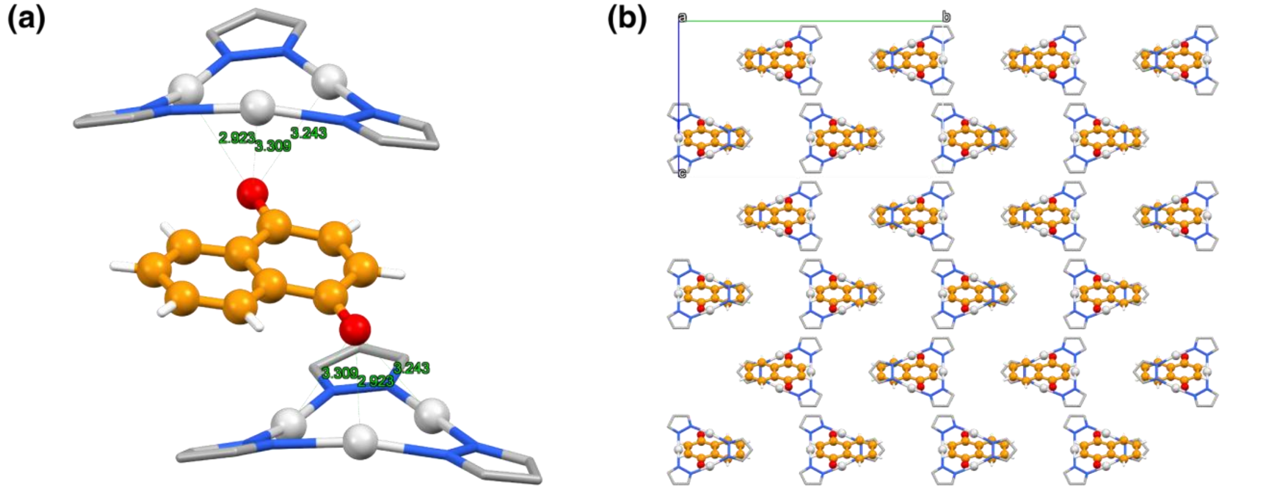

Figure S164. (a) A schematic diagram of the co-crystal structure in the **Ag_3Pz_3·49** single crystal, formed by the guest organic molecule and the surrounding Ag_3Pz_3 units that exhibit significant interactions with it. (b) A 1 × 2 × 3 packing mode in the single crystal structure of **Ag_3Pz_3·49** along the *a* axis. Trifluoromethyl groups and H atoms in Ag_3Pz_3 are omitted for clarity. Ag···O interactions are indicated with green dotted lines with distances in Å. C, N, and Ag atoms in Ag_3Pz_3 are depicted in dark gray, light blue, and light gray, respectively; C, O, and H atoms in **49** are depicted in orange, red, and white, respectively.

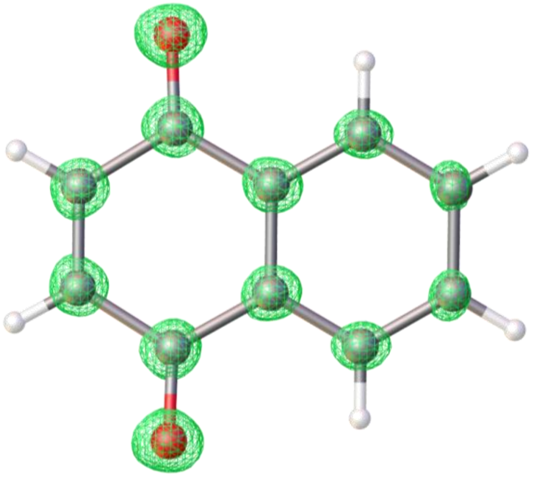

Figure S165. F_{obs} (contour: 0.55) electron density map superimposed on the structure of **49** in the single crystal structure of **Ag_3Pz_3·49**.

Preparation of Ag_3Pz_3·50. 1.86 mg (0.0107 mmol) of juglone (**50**) was dissolved in 3 mL of n-Hex, followed by the addition of equimolar amounts of Ag_3Pz_3 (10.00 mg, 0.0107 mmol). The resulting mixed solution was filtered and then transferred to a 20 mL screw-capped sample vial. The cap of the sample vial was loosely closed to allow the solvent to slowly evaporate at room temperature. The entire co-crystal incubation process was protected from light using aluminum foil. After the designated evaporation period, typically 1-3 days, high-quality yellow block-shaped crystals suitable for single-crystal X-ray diffraction analysis formed at the bottom of the vial.

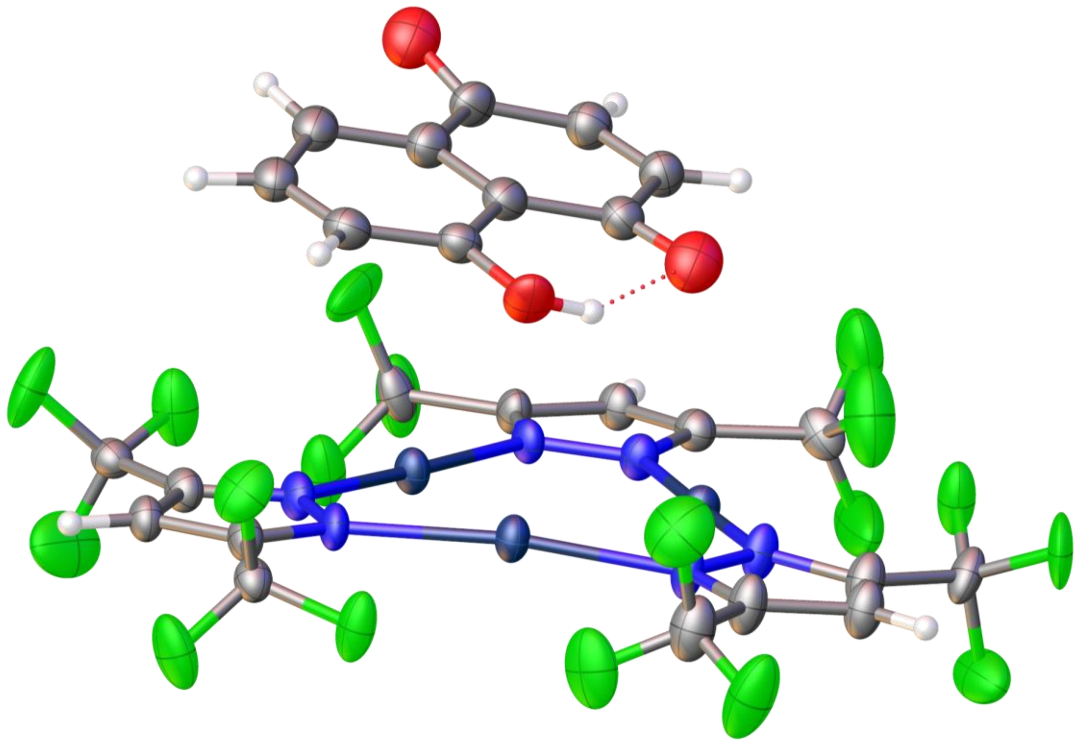

Figure S166. Asymmetric unit of **Ag_3Pz_3·50** (thermal displacement parameters at the 50% probability level).

Table S58. Crystal data and structure refinement for **Ag_3Pz_3·50**

Empirical formula	$C_{25}H_9Ag_3F_{18}N_6O_3$
Formula weight	1106.99
Temperature/K	100.02(18)
Crystal system	monoclinic
Space group	*Ia*
a/Å	16.5453(5)
b/Å	13.1583(4)
c/Å	14.3533(5)
α/°	90
β/°	92.658(3)
γ/°	90
Volume/Å^3	3121.47(17)
Z	4
ρ_{calc}g/cm^3	2.356
μ/mm^{-1}	16.373
F(000)	2112.0
Crystal size/mm^3	0.16 × 0.149 × 0.146
Radiation	Cu Kα (λ = 1.54184)
2θ range for data collection/°	8.59 to 149.956
Index ranges	$-19 \leq h \leq 20$, $-8 \leq k \leq 16$, $-17 \leq l \leq 13$
Reflections collected	7376
Independent reflections	3870 [R_{int} = 0.0619, R_{sigma} = 0.0578]
Data/restraints/parameters	3870/272/452
Goodness-of-fit on F^2	1.118
Final *R* indexes [I>=2σ (I)]	R_1 = 0.0649, wR_2 = 0.1704
Final *R* indexes [all data]	R_1 = 0.0735, wR_2 = 0.1742
Largest diff. peak/hole / e Å^{-3}	2.47/-1.67
Flack parameter	0.18(3)
CCDC-number	2501797

Responses to CheckCIF alerts for Ag_3Pz_3·50 crystal structure:

(There is no A-level alert)

B-level alerts:

"Low Bond Precision on C-C Bonds .... 0.02604 Ang."

Disordered structure.

"Check Calcd Resid. Dens. 0.94Ang From Ag03 2.57 eA-3"

This Alert is due to presence of residual density in the presence of heavy metal atom (Ag).

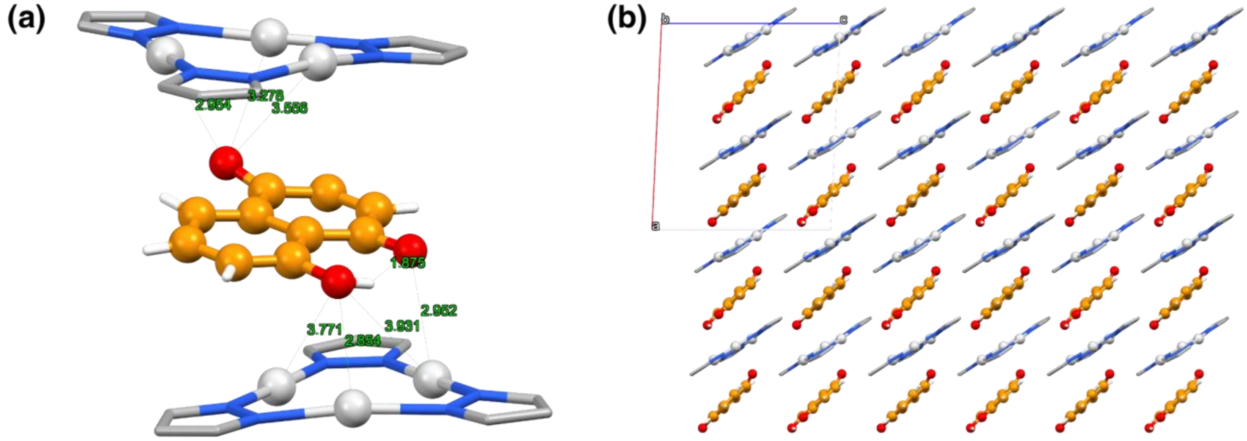

Figure S167. (a) A schematic diagram of the co-crystal structure in the **Ag_3Pz_3·50** single crystal, formed by the guest organic molecule and the surrounding Ag_3Pz_3 units that exhibit significant interactions with it. (b) A 2 × 1 × 3 packing mode in the single crystal structure of **Ag_3Pz_3·50** along the *b* axis. Trifluoromethyl groups and H atoms in Ag_3Pz_3 are omitted for clarity. Ag···O and O-H···O interactions are indicated with green dotted lines with distances in Å. C, N, and Ag atoms in Ag_3Pz_3 are depicted in dark gray, light blue, and light gray, respectively; C, O, and H atoms in **50** are depicted in orange, red, and white, respectively.

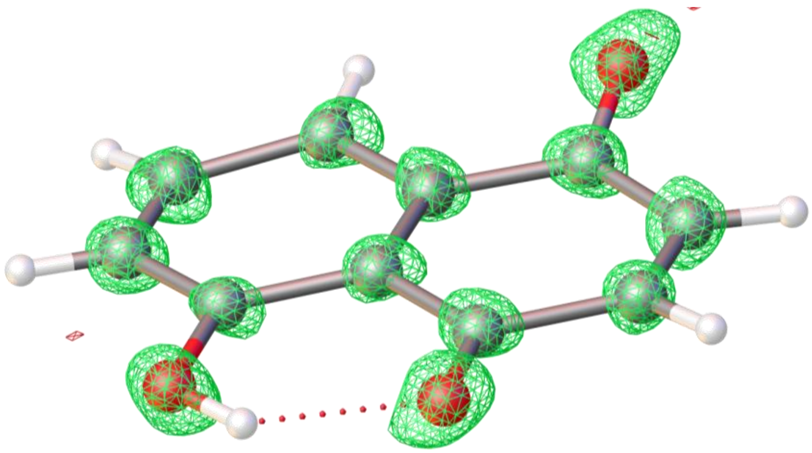

Figure S168. F_{obs} (contour: 0.34) electron density map superimposed on the structure of **50** in the single crystal structure of **Ag_3Pz_3·50**.

Preparation of Ag_3Pz_3·51. 1.67 mg (0.0107 mmol) of 2-naphthaldehyde (**51**) was dissolved in 3 mL of a binary solvent system of DCM and c-Hex (1:1, v/v), followed by the addition of equimolar amounts of Ag_3Pz_3 (10.00 mg, 0.0107 mmol). The resulting mixed solution was filtered and then transferred to a 20 mL screw-capped sample vial. The cap of the sample vial was loosely closed to allow the solvent to slowly evaporate at room temperature. The entire co-crystal incubation process was protected from light using aluminum foil. After the designated evaporation period, typically 1-3 days, high-quality colorless needle-shaped crystals suitable for single-crystal X-ray diffraction analysis formed at the bottom of the vial.

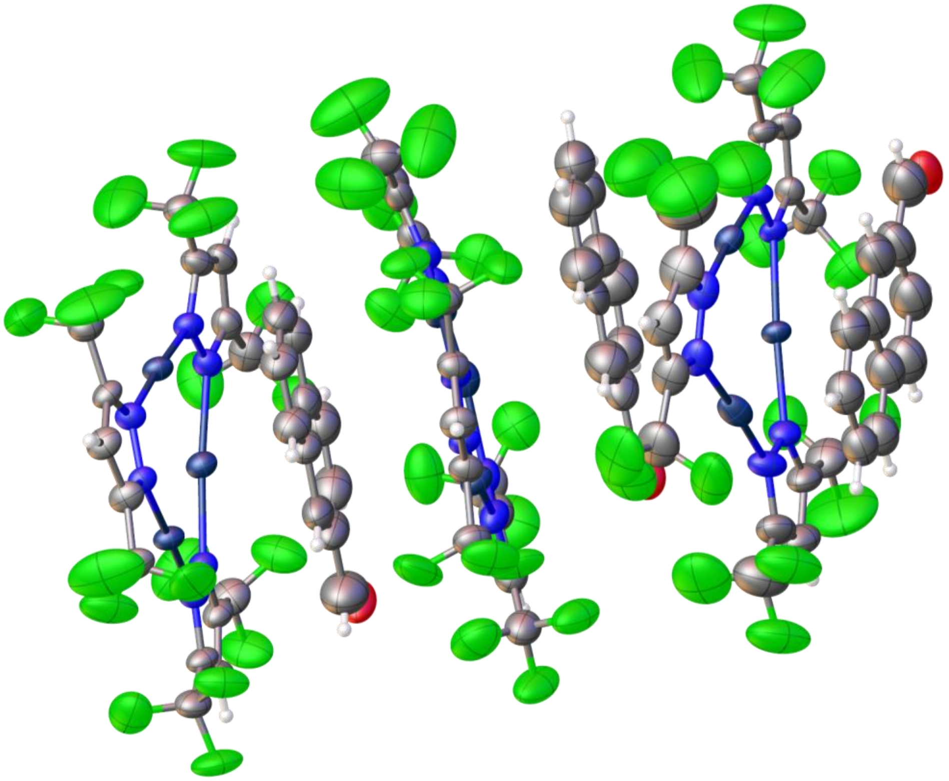

Figure S169. Asymmetric unit of **Ag_3Pz_3·51** (thermal displacement parameters at the 50% probability level).

Table S59. Crystal data and structure refinement for **Ag_3Pz_3·51**

Empirical formula	$C_{26}H_{11}Ag_3F_{18}N_6O$
Formula weight	1089.02
Temperature/K	100.15
Crystal system	triclinic
Space group	$P\bar{1}$
a/Å	14.9707(3)
b/Å	15.6255(4)
c/Å	23.4814(5)
α/°	101.863(2)
β/°	104.219(2)
γ/°	106.042(2)
Volume/Å^3	4891.8(2)
Z	6
ρ_{calc}g/cm^3	2.218
μ/mm^{-1}	15.608
F(000)	3120.0
Crystal size/mm^3	0.26 × 0.23 × 0.16
Radiation	Cu Kα (λ = 1.54184)
2θ range for data collection/°	6.156 to 133.198
Index ranges	$-17 \le h \le 17$, $-18 \le k \le 18$, $-27 \le l \le 27$
Reflections collected	45289
Independent reflections	17143 [R_{int} = 0.0753, R_{sigma} = 0.0791]
Data/restraints/parameters	17143/1412/1405
Goodness-of-fit on F^2	1.033
Final R indexes [I>=2σ (I)]	R_1 = 0.0984, wR_2 = 0.2197
Final R indexes [all data]	R_1 = 0.1293, wR_2 = 0.2338
Largest diff. peak/hole / e Å^{-3}	4.42/-2.10
CCDC-number	2501798

Responses to CheckCIF alerts for Ag_3Pz_3·51 crystal structure:

A-level alert:

"Check Calcd Resid. Dens. 1.05Ang From Ag09 4.16 eA-3"

This Alert is due to presence of residual density in the presence of heavy metal atom (Ag).

B-level alert:

"Low Bond Precision on C-C Bonds .... 0.02422 Ang."

Disordered structure.

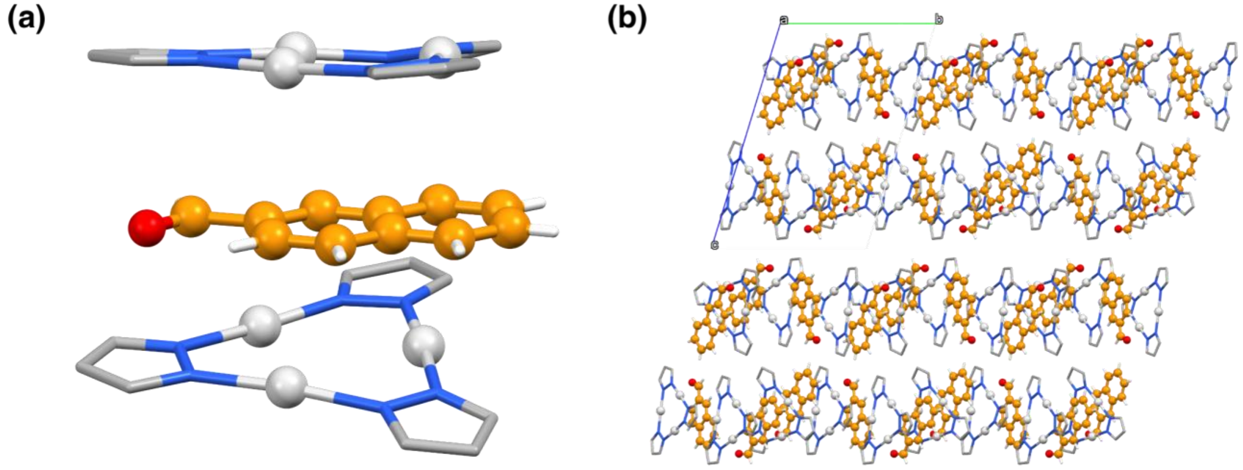

Figure S170. (a) A schematic diagram of the co-crystal structure in the **Ag_3Pz_3·51** single crystal, formed by the guest organic molecule and the surrounding Ag_3Pz_3 units that exhibit significant interactions with it. (b) A 1 × 3 × 2 packing mode in the single crystal structure of **Ag_3Pz_3·51** along the *a* axis. Trifluoromethyl groups and H atoms in Ag_3Pz_3 are omitted for clarity. C, N, and Ag atoms in Ag_3Pz_3 are depicted in dark gray, light blue, and light gray, respectively; C, O, and H atoms in **51** are depicted in orange, red, and white, respectively.

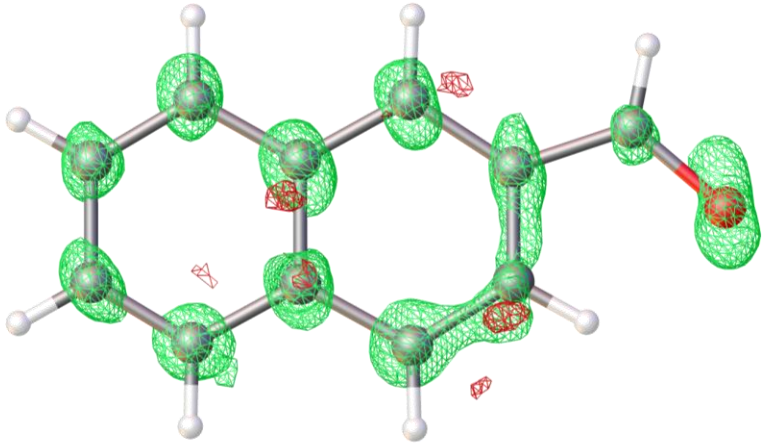

Figure S171. F_{obs} (contour: 0.30) electron density map superimposed on the structure of **51** in the single crystal structure of **Ag_3Pz_3·51**.

Preparation of Ag_3Pz_3·52. 1.82 mg (0.0107 mmol) of 2-acetylnaphthalene (**52**) was dissolved in 3 mL of n-Hex, followed by the addition of equimolar amounts of Ag_3Pz_3 (10.00 mg, 0.0107 mmol). The resulting mixed solution was filtered and then transferred to a 20 mL screw-capped sample vial. The cap of the sample vial was loosely closed to allow the solvent to slowly evaporate at room temperature. The entire co-crystal incubation process was protected from light using aluminum foil. After the designated evaporation period, typically 1-3 days, high-quality colorless plate-shaped crystals suitable for single-crystal X-ray diffraction analysis formed at the bottom of the vial.

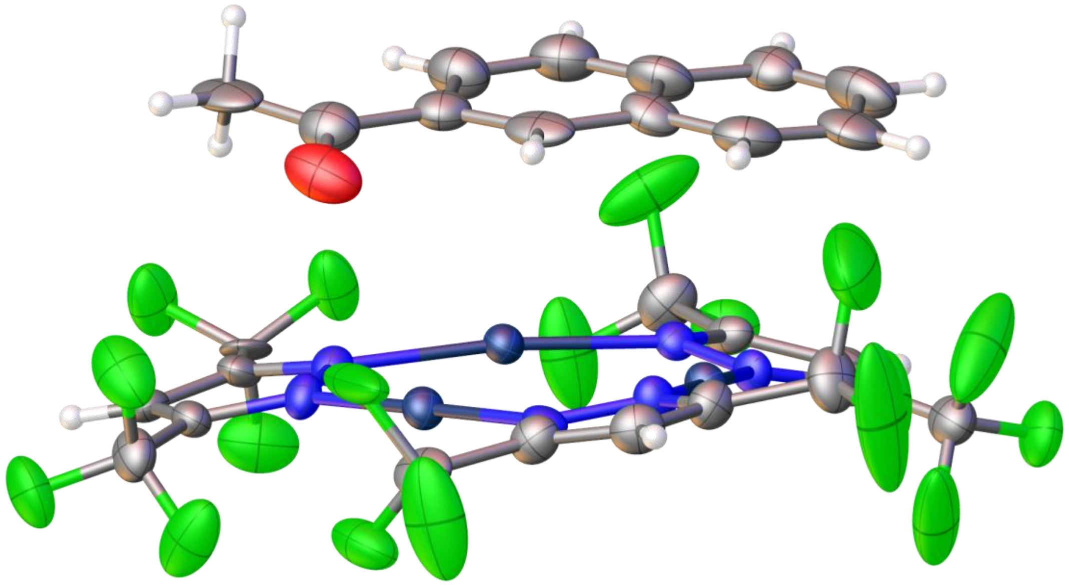

Figure S172. Asymmetric unit of **Ag_3Pz_3·52** (thermal displacement parameters at the 50% probability level).

Table S60. Crystal data and structure refinement for **Ag_3Pz_3·52**

Empirical formula	$C_{27}H_{13}Ag_3F_{18}N_6O$
Formula weight	1103.04
Temperature/K	99.95(19)
Crystal system	monoclinic
Space group	$P2_1/n$
a/Å	12.6142(3)
b/Å	22.0088(3)
c/Å	13.4981(3)
α/°	90
β/°	116.760(3)
γ/°	90
Volume/Å^3	3346.04(14)
Z	4
ρ_{calc}g/cm^3	2.190
μ/mm^{-1}	15.223
F(000)	2112.0
Crystal size/mm^3	0.46 × 0.14 × 0.13
Radiation	Cu Kα (λ = 1.54184)
2θ range for data collection/°	7.974 to 155.292
Index ranges	$-12 \le h \le 15$, $-20 \le k \le 27$, $-16 \le l \le 16$
Reflections collected	16609
Independent reflections	6767 [R_{int} = 0.0714, R_{sigma} = 0.0709]
Data/restraints/parameters	6767/24/447
Goodness-of-fit on F^2	1.121
Final R indexes [I>=2σ (I)]	R_1 = 0.0797, wR_2 = 0.1758
Final R indexes [all data]	R_1 = 0.1032, wR_2 = 0.1836
Largest diff. peak/hole / e Å^{-3}	1.83/-1.25
CCDC-number	2501799

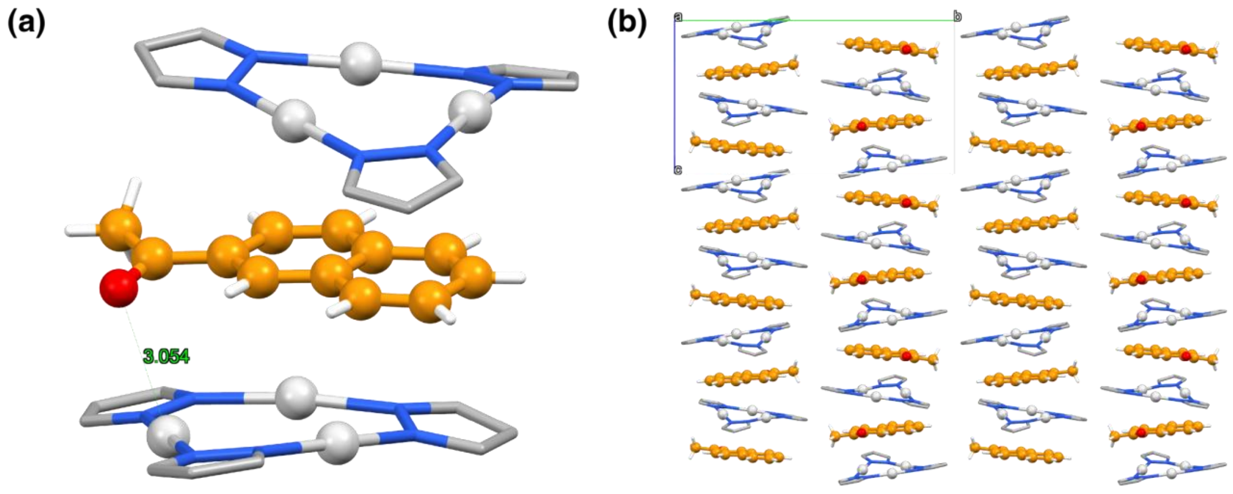

Figure S173. (a) A schematic diagram of the co-crystal structure in the **Ag_3Pz_3·52** single crystal, formed by the guest organic molecule and the surrounding Ag_3Pz_3 units that exhibit significant interactions with it. (b) A 1 × 2 × 3 packing mode in the single crystal structure of **Ag_3Pz_3·52** along the *a* axis. Trifluoromethyl groups and H atoms in Ag_3Pz_3 are omitted for clarity. Ag···O interactions are indicated with green dotted lines with distances in Å. C, N, and Ag atoms in Ag_3Pz_3 are depicted in dark gray, light blue, and light gray, respectively; C, O, and H atoms in **52** are depicted in orange, red, and white, respectively.

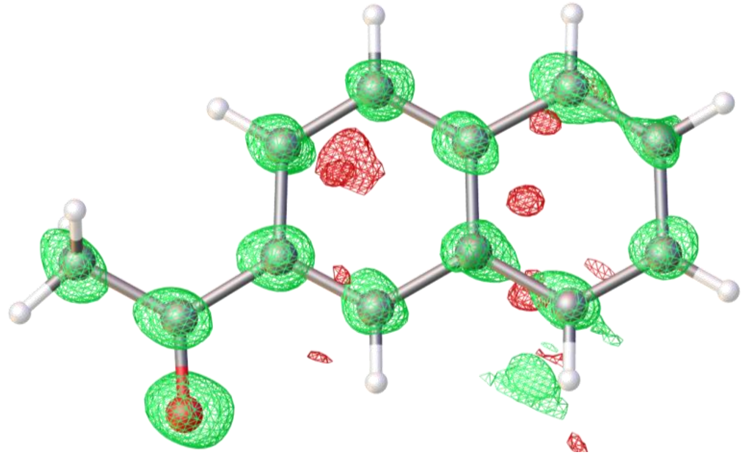

Figure S174. F_{obs} (contour: 0.65) electron density map superimposed on the structure of **52** in the single crystal structure of **Ag_3Pz_3·52**. We believe that the unassigned electron density is attributable to residual solvent molecules and the Ag_3Pz_3 units.

Preparation of Ag_3Pz_3·53. 1.89 mg (0.0107 mmol) of 7-methoxycoumarin (**53**) was dissolved in 3 mL of n-Hex, followed by the addition of equimolar amounts of Ag_3Pz_3 (10.00 mg, 0.0107 mmol). The resulting mixed solution was filtered and then transferred to a 20 mL screw-capped sample vial. The cap of the sample vial was loosely closed to allow the solvent to slowly evaporate at room temperature. The entire co-crystal incubation process was protected from light using aluminum foil. After the designated evaporation period, typically 1-3 days, high-quality colorless plate-shaped crystals suitable for single-crystal X-ray diffraction analysis formed at the bottom of the vial.

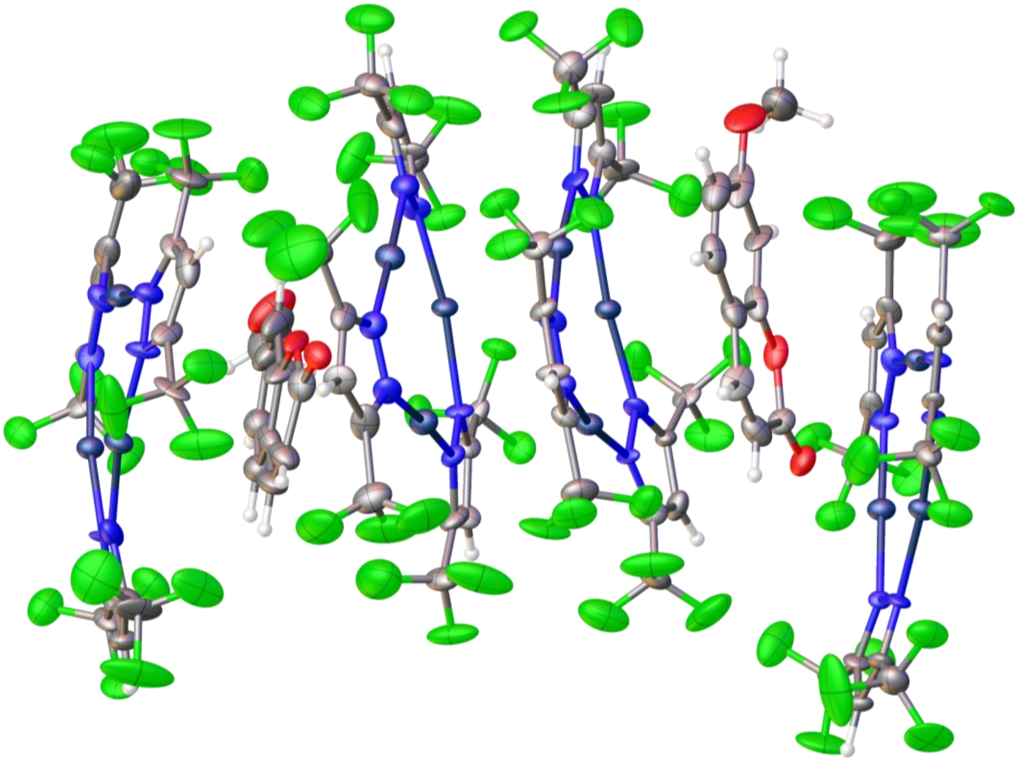

Figure S175. Asymmetric unit of **Ag_3Pz_3·53** (thermal displacement parameters at the 50% probability level).

Table S61. Crystal data and structure refinement for **Ag_3Pz_3·53**

Empirical formula	$C_{80}H_{28}Ag_{12}F_{72}N_{24}O_6$
Formula weight	4083.70
Temperature/K	100.01(15)
Crystal system	monoclinic
Space group	$P2_1$
a/Å	12.68750(10)
b/Å	22.7148(2)
c/Å	19.3275(2)
α/°	90
β/°	91.8290(10)
γ/°	90
Volume/Å^3	5567.23(9)
Z	2
ρ_{calc}g/cm^3	2.436
μ/mm^{-1}	18.230
F(000)	3872.0
Crystal size/mm^3	0.18 × 0.16 × 0.13
Radiation	Cu Kα (λ = 1.54184)
2θ range for data collection/°	6.006 to 157.002
Index ranges	$-16 \leq h \leq 16$, $-28 \leq k \leq 15$, $-23 \leq l \leq 24$
Reflections collected	34926
Independent reflections	17516 [R_{int} = 0.0379, R_{sigma} = 0.0488]
Data/restraints/parameters	17516/258/1750
Goodness-of-fit on F^2	1.074
Final R indexes [I>=2σ (I)]	R_1 = 0.0693, wR_2 = 0.1774
Final R indexes [all data]	R_1 = 0.0709, wR_2 = 0.1782
Largest diff. peak/hole / e Å^{-3}	3.72/-1.16
Flack parameter	0.026(16)
CCDC-number	2501800

Responses to CheckCIF alerts for Ag_3Pz_3·53 crystal structure:

A-level alerts:

"Check Calcd Resid. Dens. 1.17Ang From Ag01 3.67 eA-3"

This Alert is due to presence of residual density in the presence of heavy metal atom (Ag).

"Check Calcd Resid. Dens. 1.24Ang From Ag02 3.53 eA-3"

This Alert is due to presence of residual density in the presence of heavy metal atom (Ag).

"Check Calcd Resid. Dens. 1.26Ang From Ag03 3.52 eA-3"

This Alert is due to presence of residual density in the presence of heavy metal atom (Ag).

"Check Calcd Resid. Dens. 1.24Ang From Ag09 3.51 eA-3"

This Alert is due to presence of residual density in the presence of heavy metal atom (Ag).

B-level alerts:

"Low Bond Precision on C-C Bonds .... 0.03712 Ang."

Disordered structure.

"No Flack x Check Done: Low Friedel Pair Coverage 49 %"

Due to insufficient data. The compound crystallizes in the chiral space group, but no chiral ligand is used, and the complex is no longer chiral.

"Check Calcd Resid. Dens. 1.16Ang From N02N 3.47 eA-3"

This Alert is due to presence of residual density in the presence of heavy metal atom (Ag).

"Check Calcd Resid. Dens. 1.22Ang From N01J 3.25 eA-3"

This Alert is due to presence of residual density in the presence of heavy metal atom (Ag).

"Check Calcd Resid. Dens. 1.22Ang From Ag07 3.25 eA-3"

This Alert is due to presence of residual density in the presence of heavy metal atom (Ag).

"Check Calcd Resid. Dens. 1.23Ang From Ag05 3.20 eA-3"

This Alert is due to presence of residual density in the presence of heavy metal atom (Ag).

"Check Calcd Resid. Dens. 1.00Ang From N00S 3.18 eA-3"

This Alert is due to presence of residual density in the presence of heavy metal atom (Ag).

“Check Calcd Resid. Dens. 1.23Ang From N00H 3.17 eA-3”

This Alert is due to presence of residual density in the presence of heavy metal atom (Ag).

“Check Calcd Resid. Dens. 1.17Ang From Ag09 3.12 eA-3”

This Alert is due to presence of residual density in the presence of heavy metal atom (Ag).

“Check Calcd Resid. Dens. 1.25Ang From N03B 3.11 eA-3”

This Alert is due to presence of residual density in the presence of heavy metal atom (Ag).

“Check Calcd Resid. Dens. 1.26Ang From Ag05 3.01 eA-3”

This Alert is due to presence of residual density in the presence of heavy metal atom (Ag).

“Check Calcd Resid. Dens. 1.07Ang From Ag0A 2.91 eA-3”

This Alert is due to presence of residual density in the presence of heavy metal atom (Ag).

“Check Calcd Resid. Dens. 0.97Ang From N02D 2.90 eA-3”

This Alert is due to presence of residual density in the presence of heavy metal atom (Ag).

“Check Calcd Resid. Dens. 1.26Ang From Ag08 2.87 eA-3”

This Alert is due to presence of residual density in the presence of heavy metal atom (Ag).

“Check Calcd Resid. Dens. 1.11Ang From N00L 2.86 eA-3”

This Alert is due to presence of residual density in the presence of heavy metal atom (Ag).

“Check Calcd Resid. Dens. 1.21Ang From Ag0C 2.78 eA-3”

This Alert is due to presence of residual density in the presence of heavy metal atom (Ag).

“Check Calcd Resid. Dens. 1.16Ang From Ag08 2.73 eA-3”

This Alert is due to presence of residual density in the presence of heavy metal atom (Ag).

“Check Calcd Resid. Dens. 1.18Ang From Ag04 2.71 eA-3”

This Alert is due to presence of residual density in the presence of heavy metal atom (Ag).

“Check Calcd Resid. Dens. 1.14Ang From Ag02 2.66 eA-3”

This Alert is due to presence of residual density in the presence of heavy metal atom (Ag).

“Check Calcd Resid. Dens. 1.17Ang From Ag0C 2.63 eA-3”

This Alert is due to presence of residual density in the presence of heavy metal atom (Ag).

“Check Calcd Resid. Dens. 1.18Ang From Ag03   2.59 eA-3”

This Alert is due to presence of residual density in the presence of heavy metal atom (Ag).

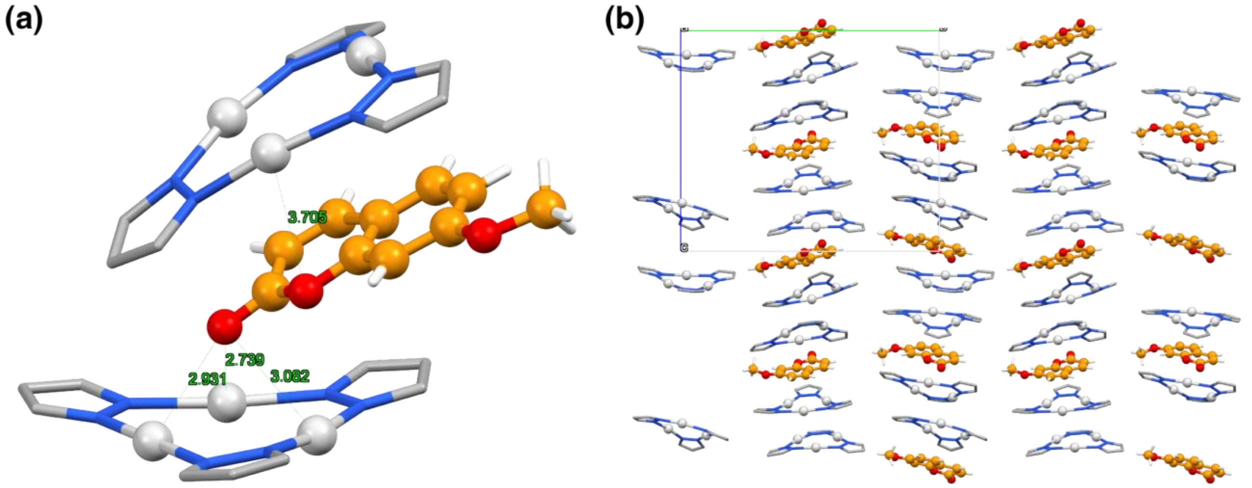

Figure S176. (a) A schematic diagram of the co-crystal structure in the **Ag_3Pz_3·53** single crystal, formed by the guest organic molecule and the surrounding Ag_3Pz_3 units that exhibit significant interactions with it. (b) A 1 × 2 × 2 packing mode in the single crystal structure of **Ag_3Pz_3·53** along the *a* axis. Trifluoromethyl groups and H atoms in Ag_3Pz_3 are omitted for clarity. Ag···O interactions are indicated with green dotted lines with distances in Å. C, N, and Ag atoms in Ag_3Pz_3 are depicted in dark gray, light blue, and light gray, respectively; C, O, and H atoms in **53** are depicted in orange, red, and white, respectively.

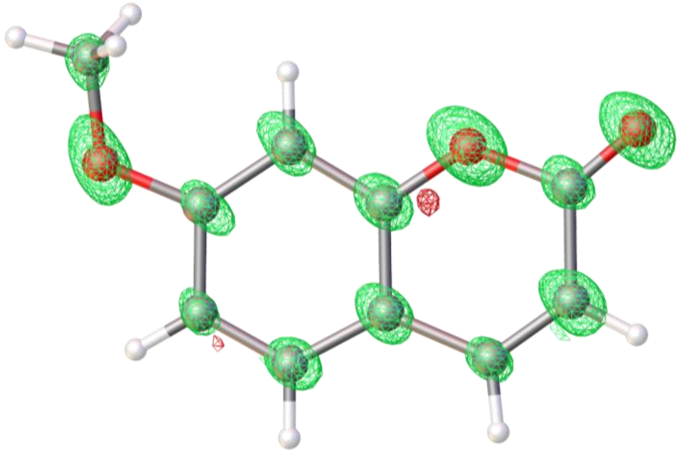

Figure S177. F_{obs} (contour: 0.50) electron density map superimposed on the structure of **53** in the single crystal structure of **Ag_3Pz_3·53**.

Preparation of Ag_3Pz_3·54. 3.06 mg (0.0107 mmol) of 1,4-dibenzoylbenzene (**54**) was dissolved in 3 mL of c-Hex, followed by the addition of equimolar amounts of Ag_3Pz_3 (10.00 mg, 0.0107 mmol). The resulting mixed solution was filtered and then transferred to a 20 mL screw-capped sample vial. The cap of the sample vial was loosely closed to allow the solvent to slowly evaporate at room temperature. The entire co-crystal incubation process was protected from light using aluminum foil. After the designated evaporation period, typically 1-3 days, high-quality colorless needle-shaped crystals suitable for single-crystal X-ray diffraction analysis formed at the bottom of the vial.

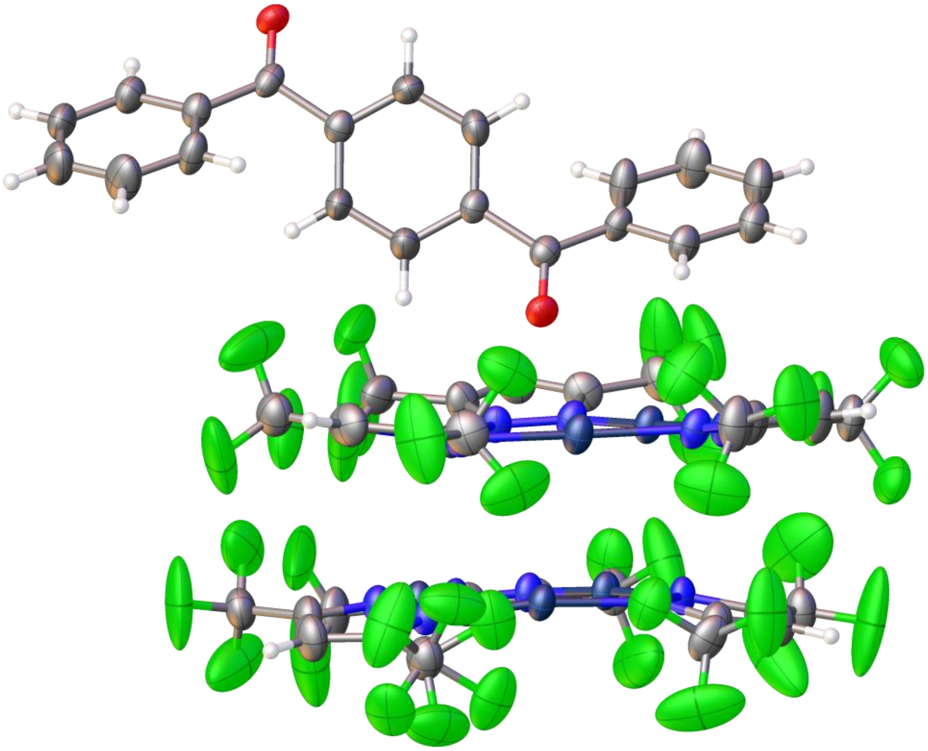

Figure S178. Asymmetric unit of **Ag_3Pz_3·54** (thermal displacement parameters at the 50% probability level).

Table S62. Crystal data and structure refinement for **Ag_3Pz_3·54**

Empirical formula	$C_{25}H_{10}Ag_3F_{18}N_6O$
Formula weight	1076.00
Temperature/K	150.00(12)
Crystal system	triclinic
Space group	$P\bar{1}$
a/Å	11.9194(3)
b/Å	12.6845(3)
c/Å	24.4397(6)
α/°	95.602(2)
β/°	92.165(2)
γ/°	115.154(2)
Volume/Å^3	3315.78(15)
Z	4
ρ_{calc}g/cm^3	2.155
μ/mm^{-1}	15.340
F(000)	2052.0
Crystal size/mm^3	0.2 × 0.1 × 0.1
Radiation	Cu Kα (λ = 1.54184)
2θ range for data collection/°	7.762 to 155.944
Index ranges	$-14 \le h \le 15$, $-12 \le k \le 16$, $-30 \le l \le 28$
Reflections collected	27969
Independent reflections	13319 [R_{int} = 0.0434, R_{sigma} = 0.0475]
Data/restraints/parameters	13319/66/977
Goodness-of-fit on F^2	1.055
Final R indexes [I>=2σ (I)]	R_1 = 0.0671, wR_2 = 0.1793
Final R indexes [all data]	R_1 = 0.0764, wR_2 = 0.1857
Largest diff. peak/hole / e Å^{-3}	2.06/-1.20
CCDC-number	2501801

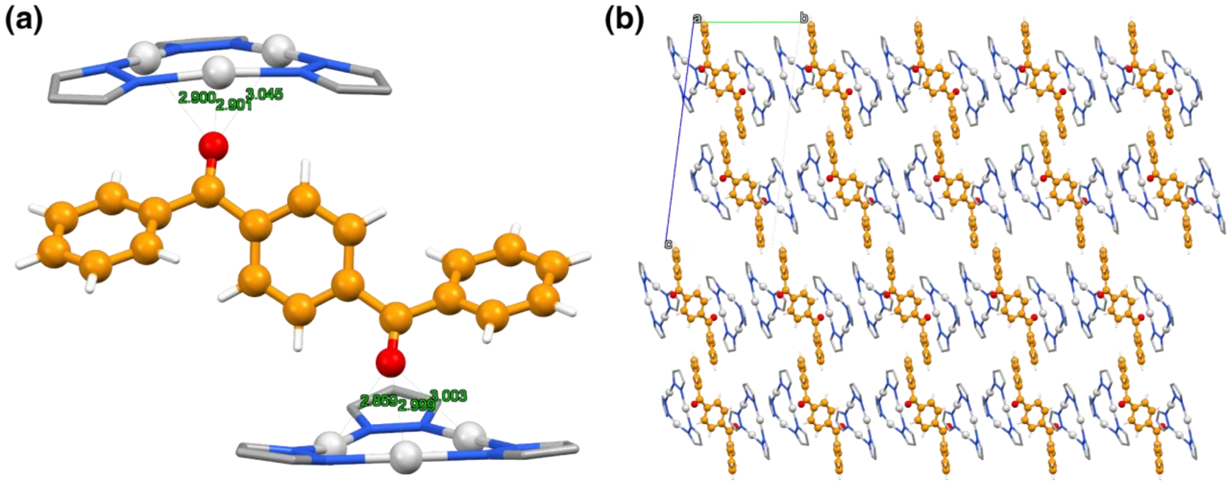

Figure S179. (a) A schematic diagram of the co-crystal structure in the **Ag_3Pz_3·54** single crystal, formed by the guest organic molecule and the surrounding Ag_3Pz_3 units that exhibit significant interactions with it. (b) A 1 × 5 × 2 packing mode in the single crystal structure of **Ag_3Pz_3·54** along the *a* axis. Trifluoromethyl groups and H atoms in Ag_3Pz_3 are omitted for clarity. Ag···O interactions are indicated with green dotted lines with distances in Å. C, N, and Ag atoms in Ag_3Pz_3 are depicted in dark gray, light blue, and light gray, respectively; C, O, and H atoms in **54** are depicted in orange, red, and white, respectively.

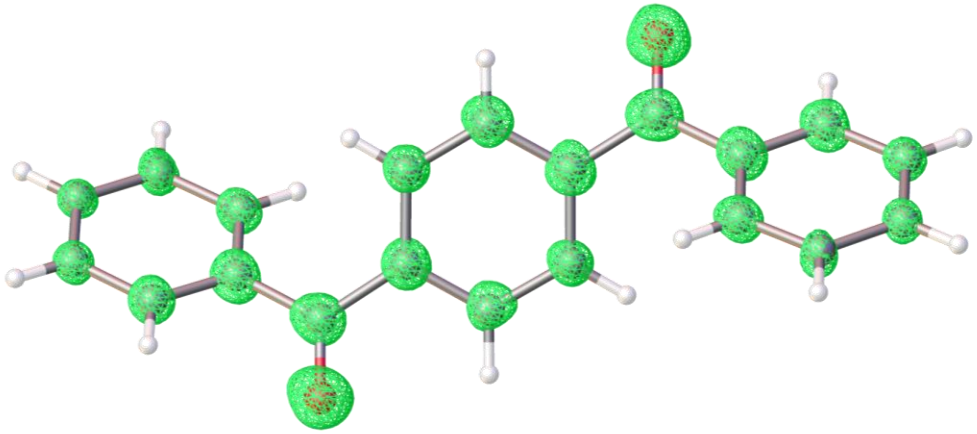

Figure S180. F_{obs} (contour: 0.70) electron density map superimposed on the structure of **54** in the single crystal structure of **Ag_3Pz_3·54**.

Preparation of Ag_3Pz_3·55. 2.04 mg (0.0107 mmol) of 7-methoxy-4-methylcoumarin (**55**) was dissolved in 3 mL of a binary solvent system of DCM and n-Hex (1:1, v/v), followed by the addition of equimolar amounts of Ag_3Pz_3 (10.00 mg, 0.0107 mmol). The resulting mixed solution was filtered and then transferred to a 20 mL screw-capped sample vial. The cap of the sample vial was loosely closed to allow the solvent to slowly evaporate at room temperature. The entire co-crystal incubation process was protected from light using aluminum foil. After the designated evaporation period, typically 1-3 days, high-quality colorless block-shaped crystals suitable for single-crystal X-ray diffraction analysis formed at the bottom of the vial.

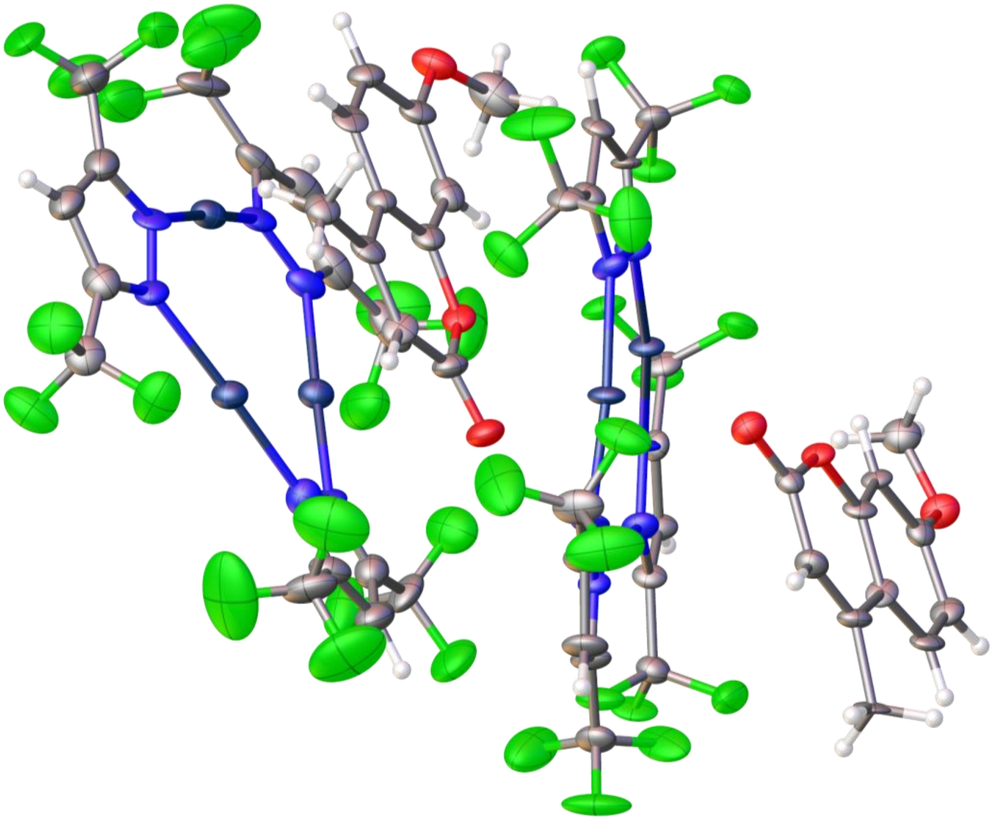

Figure S181. Asymmetric unit of **Ag_3Pz_3·55** (thermal displacement parameters at the 50% probability level).

Table S63. Crystal data and structure refinement for **Ag_3Pz_3·55**

Empirical formula	$C_{52}H_{26}Ag_6F_{36}N_{12}O_6$
Formula weight	2246.07
Temperature/K	100.2(4)
Crystal system	triclinic
Space group	$P\bar{1}$
a/Å	12.4662(3)
b/Å	13.1607(2)
c/Å	20.7819(2)
α/°	92.2890(10)
β/°	95.0270(10)
γ/°	91.1490(10)
Volume/Å^3	3392.78(10)
Z	2
ρ_{calc}g/cm^3	2.199
μ/mm^{-1}	15.074
F(000)	2152.0
Crystal size/mm^3	0.21 × 0.16 × 0.15
Radiation	Cu Kα (λ = 1.54184)
2θ range for data collection/°	6.724 to 149.998
Index ranges	$-15 \le h \le 15$, $-16 \le k \le 16$, $-26 \le l \le 20$
Reflections collected	36939
Independent reflections	13599 [R_{int} = 0.0666, R_{sigma} = 0.0891]
Data/restraints/parameters	13599/102/1007
Goodness-of-fit on F^2	1.056
Final R indexes [I>=2σ (I)]	R_1 = 0.0599, wR_2 = 0.1336
Final R indexes [all data]	R_1 = 0.0935, wR_2 = 0.1409
Largest diff. peak/hole / e Å^{-3}	1.34/-1.11
CCDC-number	2501802

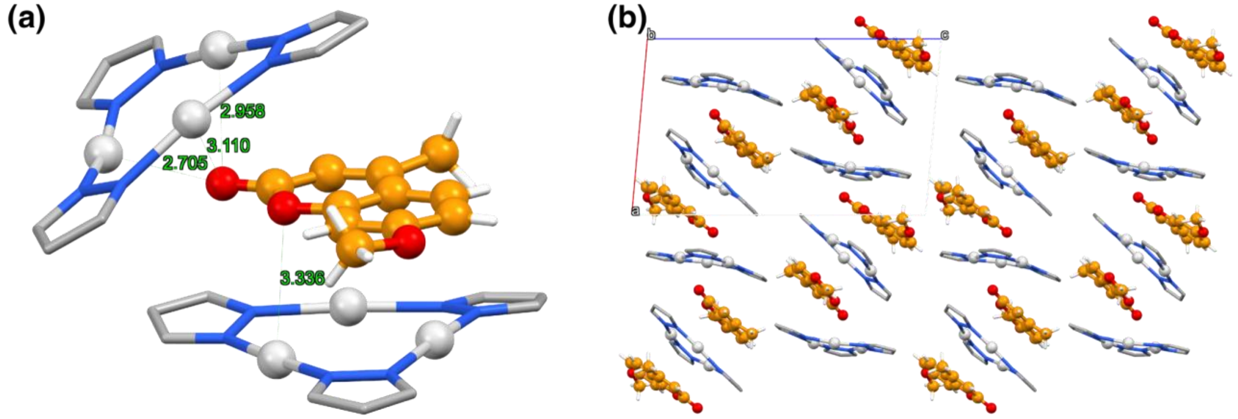

Figure S182. (a) A schematic diagram of the co-crystal structure in the **Ag_3Pz_3·55** single crystal, formed by the guest organic molecule and the surrounding Ag_3Pz_3 units that exhibit significant interactions with it. (b) A 2 × 1 × 2 packing mode in the single crystal structure of **Ag_3Pz_3·55** along the *b* axis. Trifluoromethyl groups and H atoms in Ag_3Pz_3 are omitted for clarity. Ag···O interactions are indicated with green dotted lines with distances in Å. C, N, and Ag atoms in Ag_3Pz_3 are depicted in dark gray, light blue, and light gray, respectively; C, O, and H atoms in **55** are depicted in orange, red, and white, respectively.

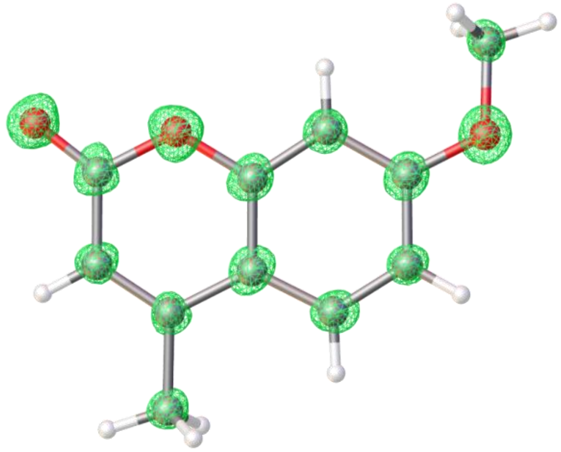

Figure S183. F_{obs} (contour: 0.85) electron density map superimposed on the structure of **55** in the single crystal structure of **Ag_3Pz_3·55**.

Preparation of Ag_3Pz_3·56. 2.46 mg (0.0107 mmol) of visnagin (**56**) was dissolved in 3 mL of a binary solvent system of DCM and n-Hex (1:1, v/v), followed by the addition of equimolar amounts of Ag_3Pz_3 (10.00 mg, 0.0107 mmol). The resulting mixed solution was filtered and then transferred to a 20 mL screw-capped sample vial. The cap of the sample vial was loosely closed to allow the solvent to slowly evaporate at room temperature. The entire co-crystal incubation process was protected from light using aluminum foil. After the designated evaporation period, typically 1-3 days, high-quality colorless needle-shaped crystals suitable for single-crystal X-ray diffraction analysis formed at the bottom of the vial.

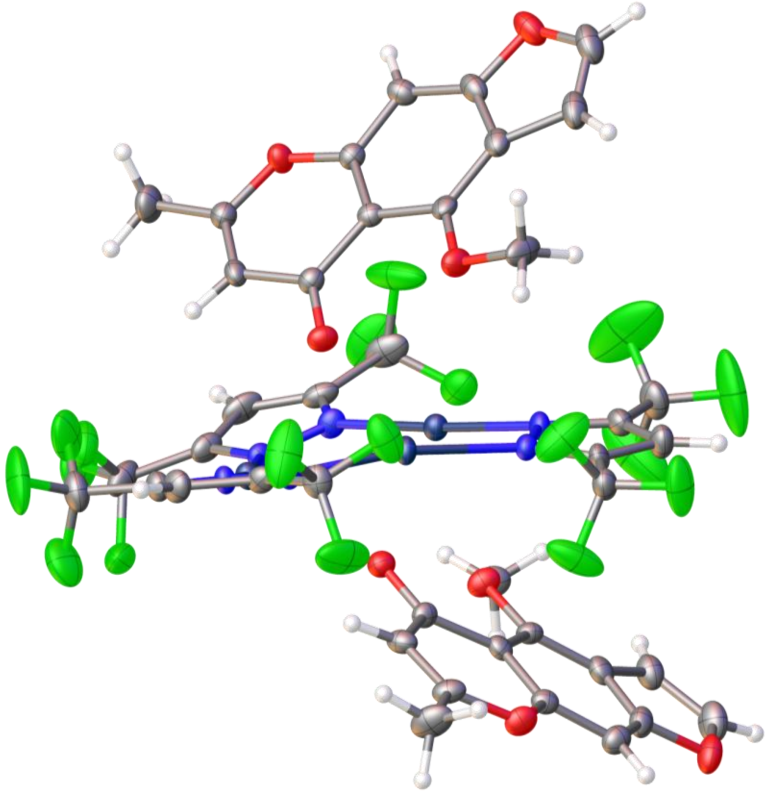

Figure S184. Asymmetric unit of **Ag_3Pz_3·56** (thermal displacement parameters at the 50% probability level).

Table S64. Crystal data and structure refinement for **Ag_3Pz_3·56**

Empirical formula	$C_{41}H_{23}Ag_3F_{18}N_6O_8$
Formula weight	1393.26
Temperature/K	99.95(18)
Crystal system	orthorhombic
Space group	$P2_12_12_1$
a/Å	12.22550(10)
b/Å	16.8622(2)
c/Å	22.5277(2)
α/°	90
β/°	90
γ/°	90
Volume/Å^3	4644.06(8)
Z	4
ρ_{calc}g/cm^3	1.993
μ/mm^{-1}	11.260
F(000)	2712.0
Crystal size/mm^3	0.16 × 0.14 × 0.13
Radiation	Cu Kα (λ = 1.54184)
2θ range for data collection/°	6.548 to 155.958
Index ranges	$-15 \le h \le 13$, $-17 \le k \le 21$, $-28 \le l \le 28$
Reflections collected	22817
Independent reflections	9225 [R_{int} = 0.0320, R_{sigma} = 0.0396]
Data/restraints/parameters	9225/0/689
Goodness-of-fit on F^2	1.100
Final R indexes [I>=2σ (I)]	R_1 = 0.0285, wR_2 = 0.0730
Final R indexes [all data]	R_1 = 0.0297, wR_2 = 0.0735
Largest diff. peak/hole / e Å^{-3}	0.68/-0.80
Flack parameter	-0.019(4)
CCDC-number	2501803

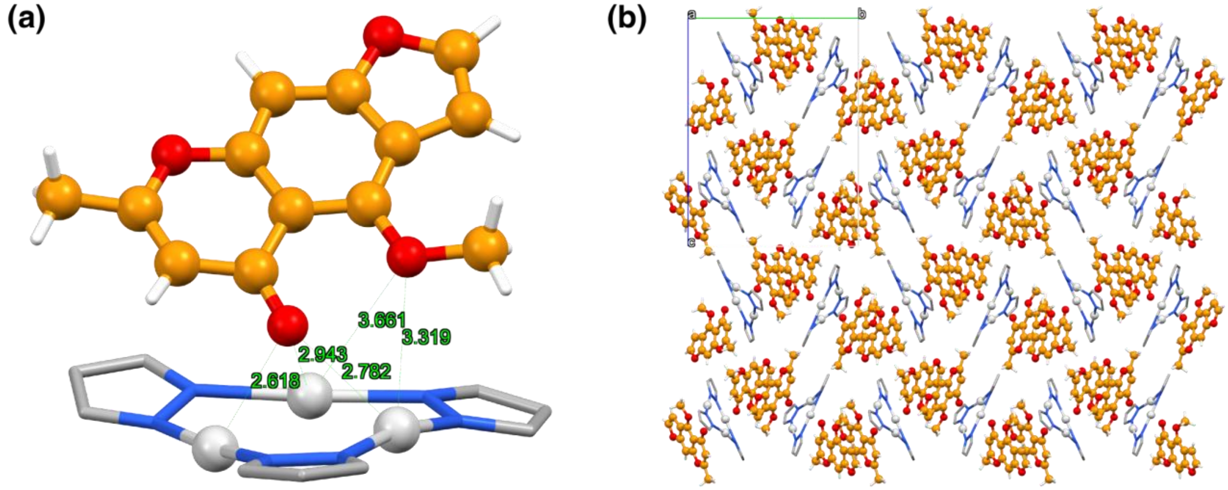

Figure S185. (a) A schematic diagram of the co-crystal structure in the $\mathbf{Ag_3Pz_3}$·**56** single crystal, formed by the guest organic molecule and the surrounding Ag_3Pz_3 units that exhibit significant interactions with it. (b) A 1 × 3 × 2 packing mode in the single crystal structure of $\mathbf{Ag_3Pz_3}$·**56** along the *a* axis. Trifluoromethyl groups and H atoms in Ag_3Pz_3 are omitted for clarity. Ag···O interactions are indicated with green dotted lines with distances in Å. C, N, and Ag atoms in Ag_3Pz_3 are depicted in dark gray, light blue, and light gray, respectively; C, O, and H atoms in **56** are depicted in orange, red, and white, respectively.

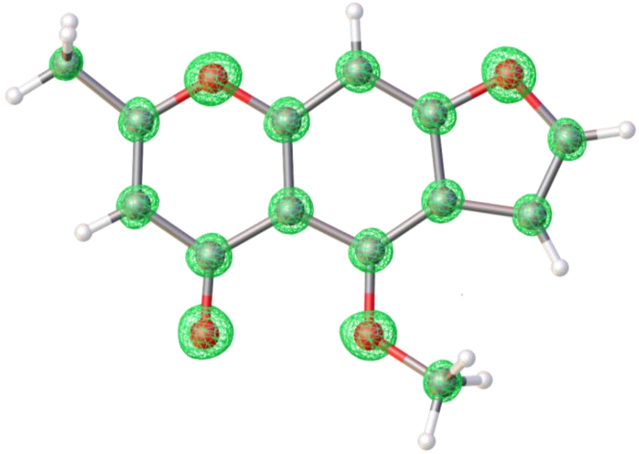

Figure S186. F_{obs} (contour: 1.20) electron density map superimposed on the structure of **56** in the single crystal structure of $\mathbf{Ag_3Pz_3}$·**56**.

Preparation of Ag_3Pz_3·57. 2.08 mg (0.0107 mmol) of anthrone (**57**) was dissolved in 3 mL of DCM, followed by the addition of equimolar amounts of Ag_3Pz_3 (10.00 mg, 0.0107 mmol). The resulting mixed solution was filtered and then transferred to a 20 mL screw-capped sample vial. The cap of the sample vial was loosely closed to allow the solvent to slowly evaporate at room temperature. The entire co-crystal incubation process was protected from light using aluminum foil. After the designated evaporation period, typically 1-3 days, high-quality colorless plate-shaped crystals suitable for single-crystal X-ray diffraction analysis formed at the bottom of the vial.

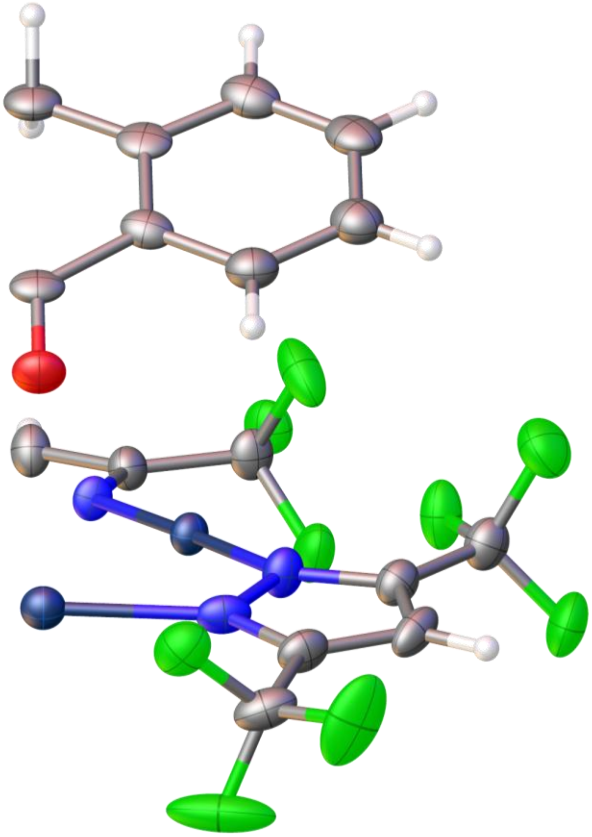

Figure S187. Asymmetric unit of **Ag_3Pz_3·57** (thermal displacement parameters at the 50% probability level).

Table S65. Crystal data and structure refinement for $\mathbf{Ag_3Pz_3 \cdot 57}$

Empirical formula	$C_{14.5}H_{6.5}Ag_{1.5}F_9N_3O_{0.5}$
Formula weight	563.53
Temperature/K	100.0(2)
Crystal system	orthorhombic
Space group	*Pnma*
a/Å	11.8389(3)
b/Å	22.7203(4)
c/Å	12.4354(3)
α/°	90
β/°	90
γ/°	90
Volume/Å^3	3344.92(13)
Z	8
ρ_{calc}g/cm^3	2.238
μ/mm^{-1}	15.250
F(000)	2160.0
Crystal size/mm^3	0.18 × 0.16 × 0.16
Radiation	Cu Kα (λ = 1.54184)
2θ range for data collection/°	8.106 to 157.654
Index ranges	$-14 \leq h \leq 9$, $-28 \leq k \leq 25$, $-15 \leq l \leq 15$
Reflections collected	12568
Independent reflections	3501 [R_{int} = 0.0455, R_{sigma} = 0.0355]
Data/restraints/parameters	3501/48/265
Goodness-of-fit on F^2	1.148
Final R indexes [I>=2σ (I)]	R_1 = 0.0712, wR_2 = 0.2011
Final R indexes [all data]	R_1 = 0.0795, wR_2 = 0.2060
Largest diff. peak/hole / e Å^{-3}	3.52/-1.33
CCDC-number	2501804

Responses to CheckCIF alerts for Ag_3Pz_3·57 crystal structure:

A-level alerts:

"Check Calcd Resid. Dens. 0.99Ang From Ag01 4.03 eA-3"

This Alert is due to presence of residual density in the presence of heavy metal atom (Ag).

"Check Calcd Resid. Dens. 1.00Ang From Ag02 3.84 eA-3"

This Alert is due to presence of residual density in the presence of heavy metal atom (Ag).

"Check Calcd Resid. Dens. 0.90Ang From Ag01 3.57 eA-3"

This Alert is due to presence of residual density in the presence of heavy metal atom (Ag).

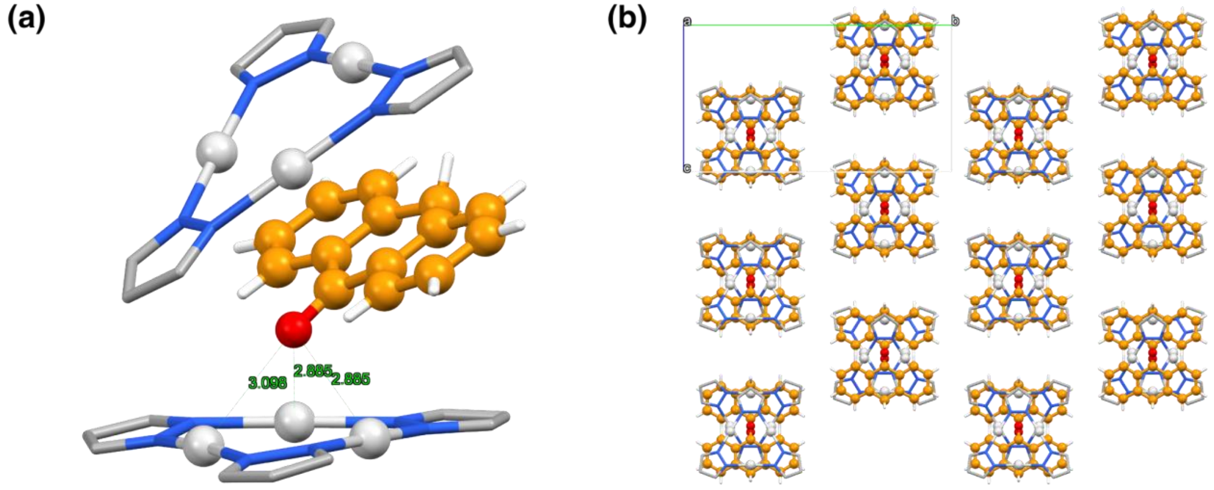

Figure S188. (a) A schematic diagram of the co-crystal structure in the $\mathbf{Ag_3Pz_3{\cdot}57}$ single crystal, formed by the guest organic molecule and the surrounding Ag_3Pz_3 units that exhibit significant interactions with it. (b) A 1 × 2 × 3 packing mode in the single crystal structure of $\mathbf{Ag_3Pz_3{\cdot}57}$ along the *a* axis. Trifluoromethyl groups and H atoms in Ag_3Pz_3 are omitted for clarity. Ag···O interactions are indicated with green dotted lines with distances in Å. C, N, and Ag atoms in Ag_3Pz_3 are depicted in dark gray, light blue, and light gray, respectively; C, O, and H atoms in **57** are depicted in orange, red, and white, respectively.

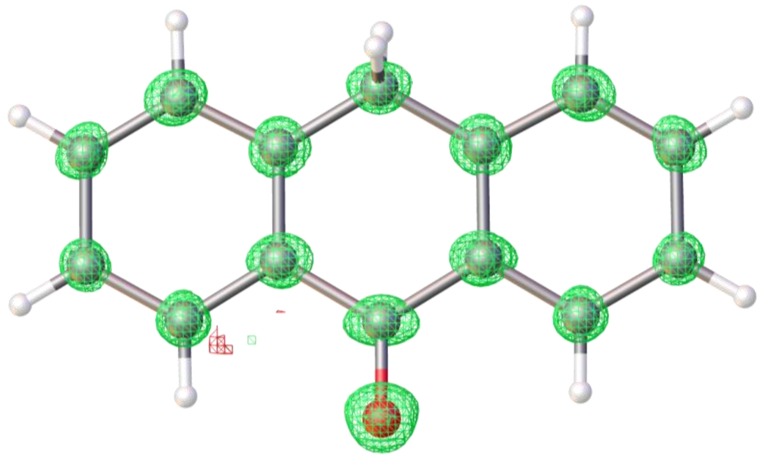

Figure S189. F_{obs} (contour: 0.75) electron density map superimposed on the structure of **57** in the single crystal structure of $\mathbf{Ag_3Pz_3{\cdot}57}$.

Preparation of Ag_3Pz_3·58. 2.36 mg (0.0107 mmol) of 9-acetylanthracene (**58**) was dissolved in 3 mL of c-Hex, followed by the addition of equimolar amounts of Ag_3Pz_3 (10.00 mg, 0.0107 mmol). The resulting mixed solution was filtered and then transferred to a 20 mL screw-capped sample vial. The cap of the sample vial was loosely closed to allow the solvent to slowly evaporate at room temperature. The entire co-crystal incubation process was protected from light using aluminum foil. After the designated evaporation period, typically 1-3 days, high-quality colorless block-shaped crystals suitable for single-crystal X-ray diffraction analysis formed at the bottom of the vial.

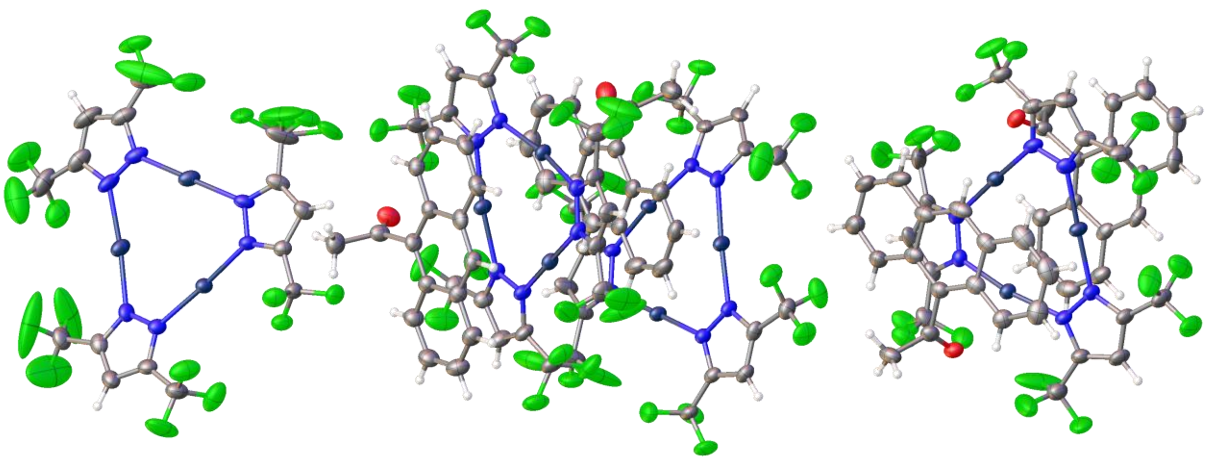

Figure S190. Asymmetric unit of **Ag_3Pz_3·58** (thermal displacement parameters at the 50% probability level).

Table S66. Crystal data and structure refinement for **Ag_3Pz_3·58**

Empirical formula	$C_{31}H_{15}Ag_3F_{18}N_6O$
Formula weight	1153.10
Temperature/K	100.00(10)
Crystal system	triclinic
Space group	$P\bar{1}$
a/Å	12.9913(2)
b/Å	22.0768(2)
c/Å	26.2307(2)
α/°	70.2030(10)
β/°	82.2560(10)
γ/°	87.7040(10)
Volume/Å^3	7013.82(14)
Z	8
ρ_{calc}g/cm^3	2.184
μ/mm^{-1}	14.566
F(000)	4432.0
Crystal size/mm^3	0.3 × 0.2 × 0.1
Radiation	Cu Kα (λ = 1.54184)
2θ range for data collection/°	7.042 to 146.626
Index ranges	$-16 \le h \le 15$, $-27 \le k \le 27$, $-21 \le l \le 30$
Reflections collected	119258
Independent reflections	26333 [R_{int} = 0.0354, R_{sigma} = 0.0252]
Data/restraints/parameters	26333/63/2157
Goodness-of-fit on F^2	1.065
Final R indexes [I>=2σ (I)]	R_1 = 0.0485, wR_2 = 0.1234
Final R indexes [all data]	R_1 = 0.0561, wR_2 = 0.1288
Largest diff. peak/hole / e Å^{-3}	2.09/-1.63
CCDC-number	2501805

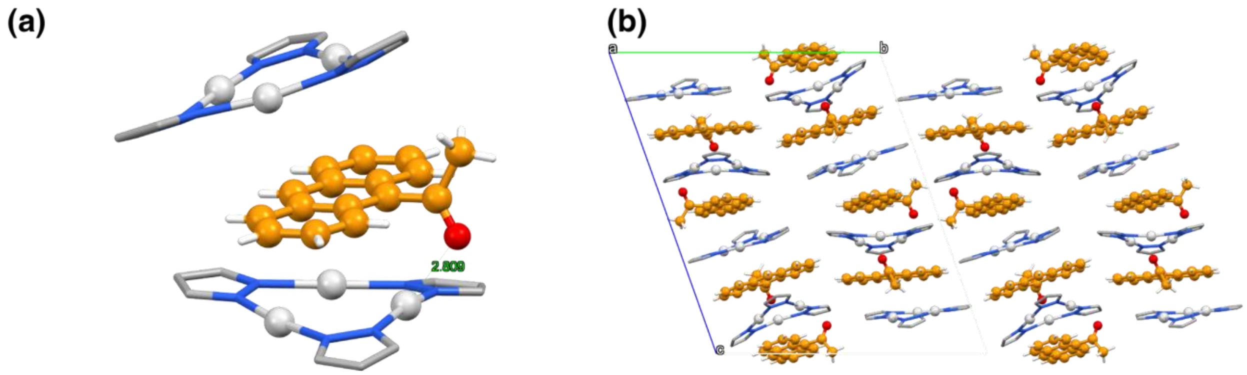

Figure S191. (a) A schematic diagram of the co-crystal structure in the **Ag_3Pz_3·58** single crystal, formed by the guest organic molecule and the surrounding Ag_3Pz_3 units that exhibit significant interactions with it. (b) A 1 × 2 × 1 packing mode in the single crystal structure of **Ag_3Pz_3·58** along the *a* axis. Trifluoromethyl groups and H atoms in Ag_3Pz_3 are omitted for clarity. Ag···O interactions are indicated with green dotted lines with distances in Å. C, N, and Ag atoms in Ag_3Pz_3 are depicted in dark gray, light blue, and light gray, respectively; C, O, and H atoms in **58** are depicted in orange, red, and white, respectively.

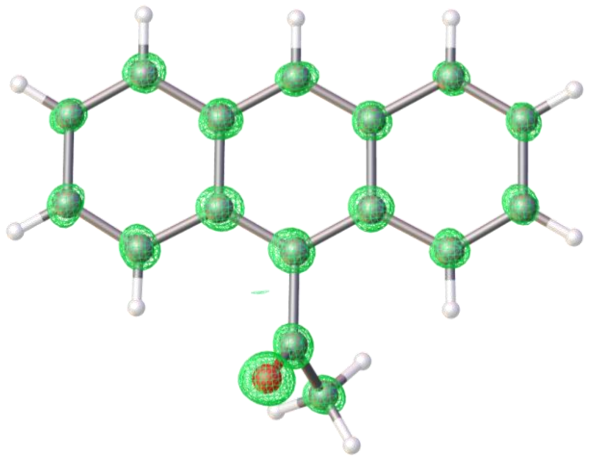

Figure S192. F_{obs} (contour: 0.40) electron density map superimposed on the structure of **58** in the single crystal structure of **Ag_3Pz_3·58**.

Preparation of $\mathbf{Ag_3Pz_3{\cdot}59}$. 2.23 mg (0.0107 mmol) of 9,10-phenanthrenedione (**59**) was dissolved in 3 mL of a binary solvent system of DCM and n-Hex (1:1, v/v), followed by the addition of equimolar amounts of Ag_3Pz_3 (10.00 mg, 0.0107 mmol). The resulting mixed solution was filtered and then transferred to a 20 mL screw-capped sample vial. The cap of the sample vial was loosely closed to allow the solvent to slowly evaporate at room temperature. The entire co-crystal incubation process was protected from light using aluminum foil. After the designated evaporation period, typically 1-3 days, high-quality brown needle-shaped crystals suitable for single-crystal X-ray diffraction analysis formed at the bottom of the vial.

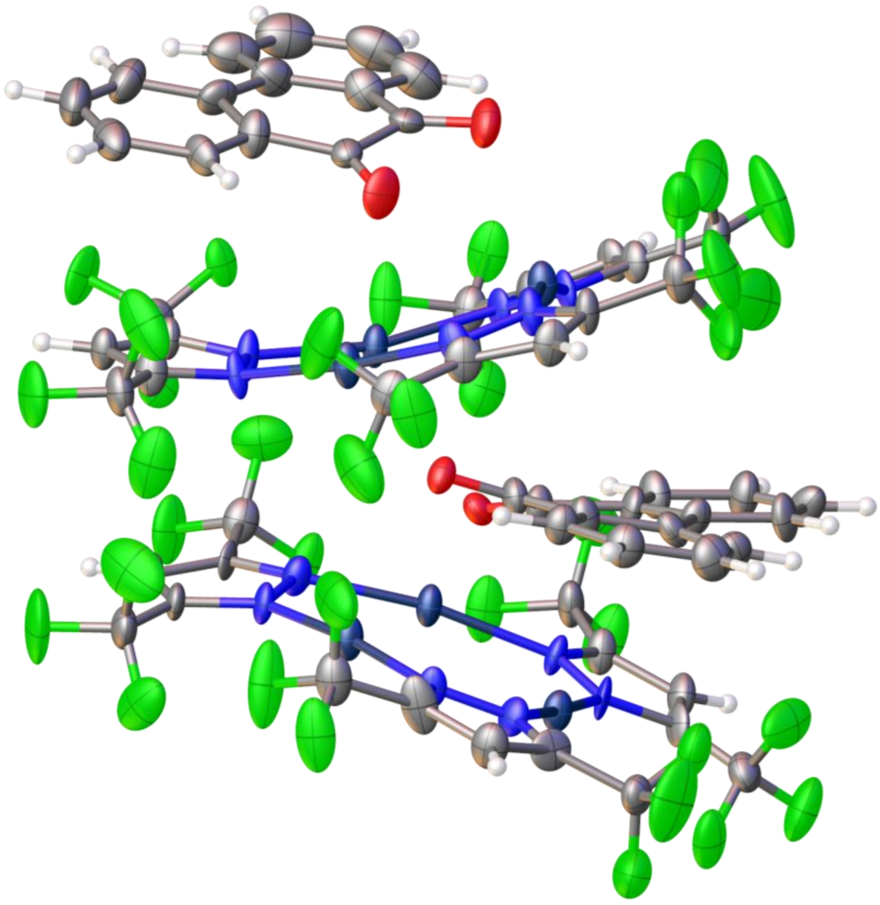

Figure S193. Asymmetric unit of $\mathbf{Ag_3Pz_3{\cdot}59}$ (thermal displacement parameters at the 50% probability level).

Table S67. Crystal data and structure refinement for **Ag_3Pz_3·59**

Empirical formula	$C_{58}H_{22}Ag_6F_{36}N_{12}O_4$
Formula weight	2282.09
Temperature/K	99.97(19)
Crystal system	hexagonal
Space group	$P6_5$
a/Å	13.16180(10)
b/Å	13.16180(10)
c/Å	68.0701(13)
α/°	90
β/°	90
γ/°	120
Volume/Å^3	10212.2(2)
Z	6
ρ_{calc}g/cm^3	2.226
μ/mm^{-1}	15.020
F(000)	6552.0
Crystal size/mm^3	0.2 × 0.2 × 0.1
Radiation	Cu Kα (λ = 1.54184)
2θ range for data collection/°	7.756 to 157.05
Index ranges	$-12 \le h \le 16$, $-16 \le k \le 11$, $-84 \le l \le 68$
Reflections collected	41193
Independent reflections	13110 [R_{int} = 0.0948, R_{sigma} = 0.0769]
Data/restraints/parameters	13110/232/1046
Goodness-of-fit on F^2	1.145
Final R indexes [I>=2σ (I)]	R_1 = 0.0802, wR_2 = 0.1566
Final R indexes [all data]	R_1 = 0.0915, wR_2 = 0.1600
Largest diff. peak/hole / e Å^{-3}	2.76/-1.38
Flack parameter	0.028(17)
CCDC-number	2501806

Responses to CheckCIF alerts for Ag_3Pz_3·59 crystal structure:

(There is no A-level alert)

B-level alerts:

"Low Bond Precision on C-C Bonds .... 0.03321 Ang."

Disordered structure.

"Check Calcd Resid. Dens. 1.12Ang From Ag02 2.89 eA-3"

This Alert is due to presence of residual density in the presence of heavy metal atom (Ag).

"Check Calcd Resid. Dens. 1.03Ang From Ag03 2.71 eA-3"

This Alert is due to presence of residual density in the presence of heavy metal atom (Ag).

"Check Calcd Resid. Dens. 1.08Ang From Ag03 2.62 eA-3"

This Alert is due to presence of residual density in the presence of heavy metal atom (Ag).

"Check Calcd Resid. Dens. 1.02Ang From Ag02 2.59 eA-3"

This Alert is due to presence of residual density in the presence of heavy metal atom (Ag).

"Check Calcd Resid. Dens. 1.03Ang From Ag06 2.51 eA-3"

This Alert is due to presence of residual density in the presence of heavy metal atom (Ag).

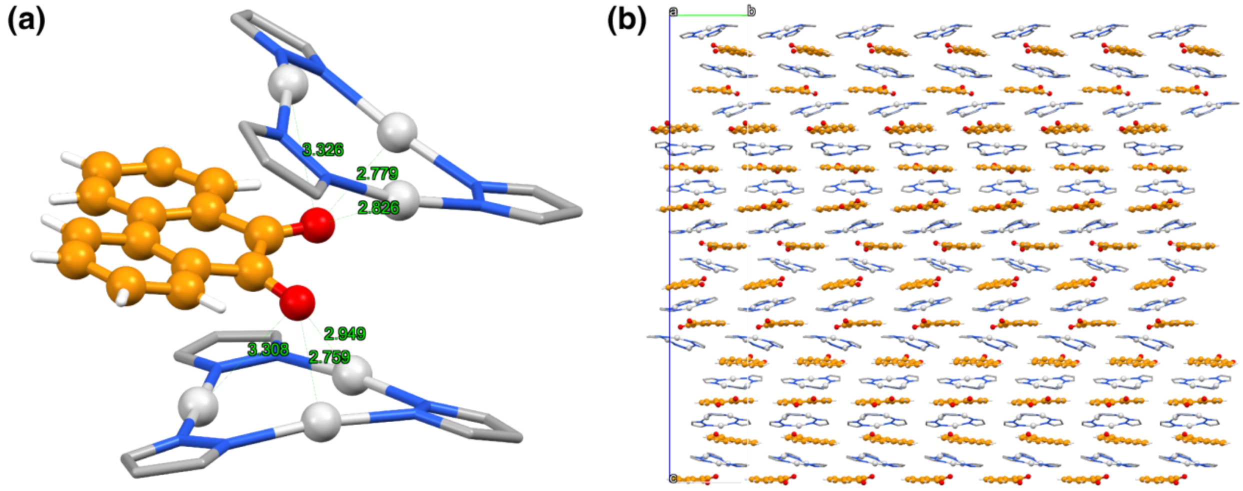

Figure S194. (a) A schematic diagram of the co-crystal structure in the **Ag_3Pz_3·59** single crystal, formed by the guest organic molecule and the surrounding Ag_3Pz_3 units that exhibit significant interactions with it. (b) A 1 × 7 × 1 packing mode in the single crystal structure of **Ag_3Pz_3·59** along the *a* axis. Trifluoromethyl groups and H atoms in Ag_3Pz_3 are omitted for clarity. Ag···O interactions are indicated with green dotted lines with distances in Å. C, N, and Ag atoms in Ag_3Pz_3 are depicted in dark gray, light blue, and light gray, respectively; C, O, and H atoms in **59** are depicted in orange, red, and white, respectively.

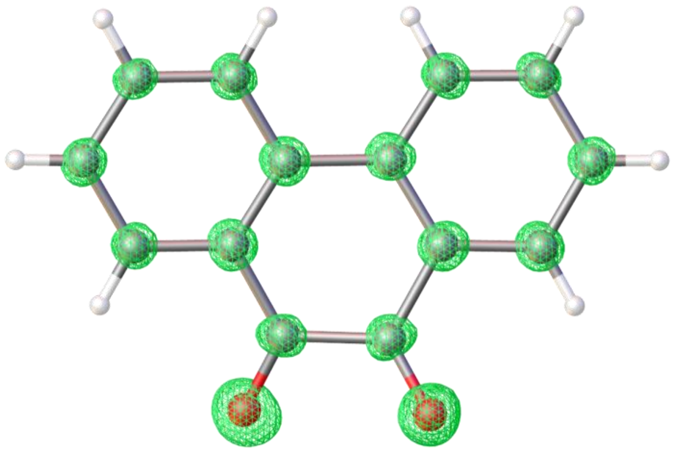

Figure S195. F_{obs} (contour: 0.30) electron density map superimposed on the structure of **59** in the single crystal structure of **Ag_3Pz_3·59**.

Preparation of Ag_3Pz_3·60. 2.27 mg (0.0107 mmol) of urolithin B (**60**) was dissolved in 3 mL of a binary solvent system of DCM and MeOH (1:1, v/v), followed by the addition of equimolar amounts of Ag_3Pz_3 (10.00 mg, 0.0107 mmol). The resulting mixed solution was filtered and then transferred to a 20 mL screw-capped sample vial. The cap of the sample vial was loosely closed to allow the solvent to slowly evaporate at room temperature. The entire co-crystal incubation process was protected from light using aluminum foil. After the designated evaporation period, typically 1-3 days, high-quality colorless needle-shaped crystals suitable for single-crystal X-ray diffraction analysis formed at the bottom of the vial.

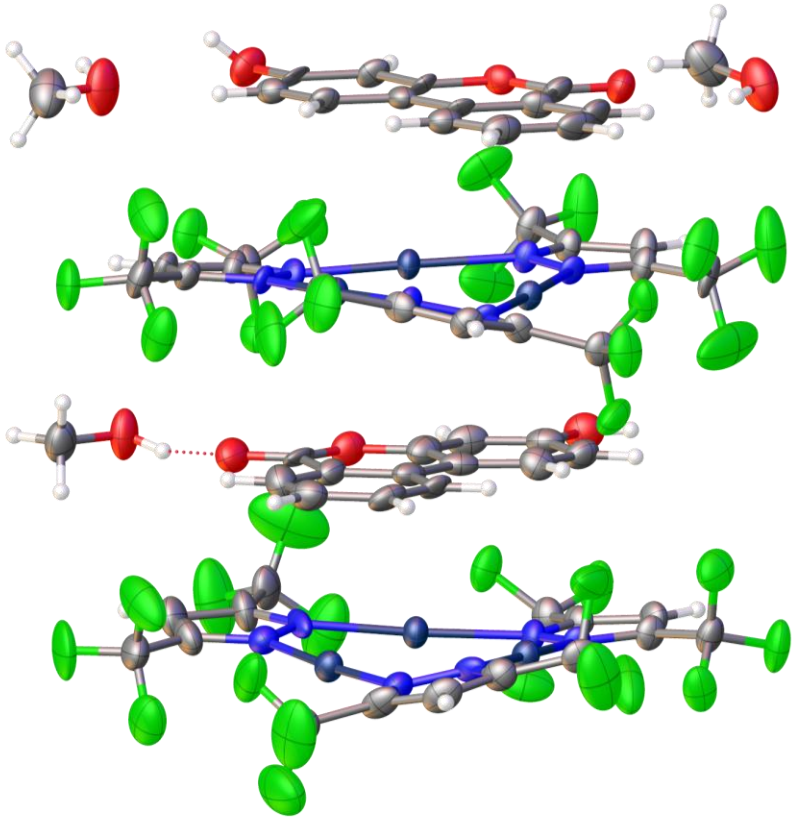

Figure S196. Asymmetric unit of **Ag_3Pz_3·60** (thermal displacement parameters at the 50% probability level).

Table S68. Crystal data and structure refinement for **Ag_3Pz_3·60**

Empirical formula	$C_{59}H_{34}Ag_6F_{36}N_{12}O_9$
Formula weight	2386.20
Temperature/K	100.0(3)
Crystal system	triclinic
Space group	$P\bar{1}$
a/Å	12.4289(2)
b/Å	13.0611(2)
c/Å	23.2463(3)
α/°	86.6960(10)
β/°	88.8370(10)
γ/°	76.5370(10)
Volume/Å^3	3663.80(10)
Z	2
ρ_{calc}g/cm^3	2.163
μ/mm^{-1}	14.044
F(000)	2300.0
Crystal size/mm^3	0.15 × 0.14 × 0.12
Radiation	Cu Kα (λ = 1.54184)
2θ range for data collection/°	6.97 to 155.666
Index ranges	$-15 \le h \le 15$, $-14 \le k \le 16$, $-28 \le l \le 29$
Reflections collected	35414
Independent reflections	14861 [R_{int} = 0.0665, R_{sigma} = 0.0734]
Data/restraints/parameters	14861/66/1107
Goodness-of-fit on F^2	1.043
Final R indexes [I>=2σ (I)]	R_1 = 0.0877, wR_2 = 0.1935
Final R indexes [all data]	R_1 = 0.1120, wR_2 = 0.2023
Largest diff. peak/hole / e Å^{-3}	3.00/-1.47
CCDC-number	2501808

Responses to CheckCIF alerts for Ag_3Pz_3·60 crystal structure:

(There is no A-level alert)

B-level alerts:

"Check Calcd Resid. Dens. 0.99Ang From N01C 3.07 eA-3"

This Alert is due to presence of residual density in the presence of heavy metal atom (Ag).

"Check Calcd Resid. Dens. 0.99Ang From N00Y 2.58 eA-3"

This Alert is due to presence of residual density in the presence of heavy metal atom (Ag).

"Check Calcd Resid. Dens. 1.02Ang From N00M 2.51 eA-3"

This Alert is due to presence of residual density in the presence of heavy metal atom (Ag).

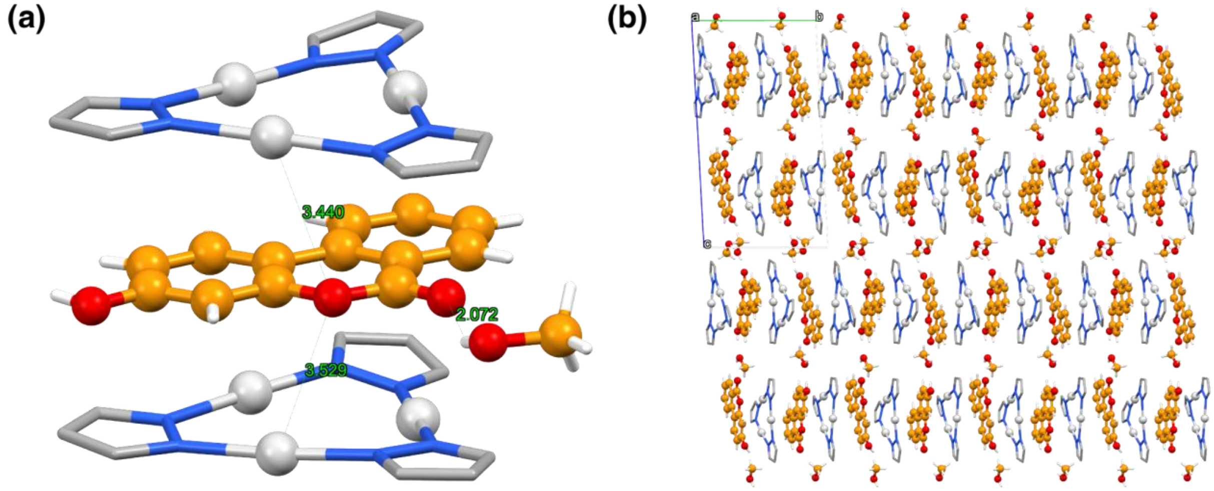

Figure S197. (a) A schematic diagram of the co-crystal structure in the **Ag_3Pz_3·60** single crystal, formed by the guest organic molecule and the surrounding Ag_3Pz_3 units that exhibit significant interactions with it. (b) A 1 × 4 × 2 packing mode in the single crystal structure of **Ag_3Pz_3·60** along the *a* axis. Trifluoromethyl groups and H atoms in Ag_3Pz_3 are omitted for clarity. Ag···O and O-H···O interactions are indicated with green dotted lines with distances in Å. C, N, and Ag atoms in Ag_3Pz_3 are depicted in dark gray, light blue, and light gray, respectively; C, O, and H atoms in **60** are depicted in orange, red, and white, respectively.

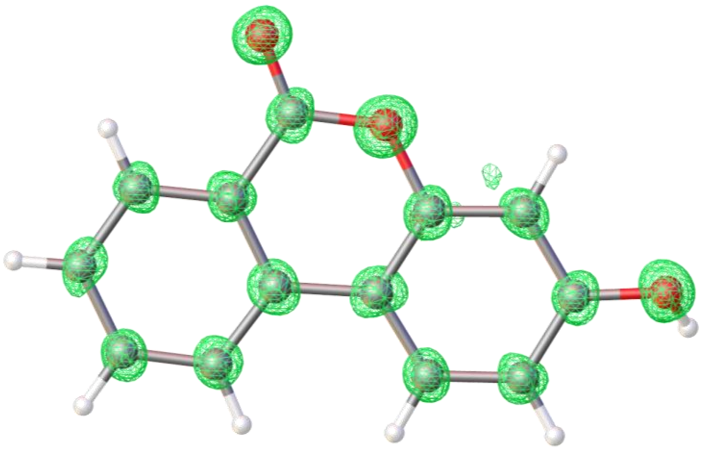

Figure S198. F_{obs} (contour: 0.70) electron density map superimposed on the structure of **60** in the single crystal structure of **Ag_3Pz_3·60**.

Preparation of Ag_3Pz_3·61. 2.40 mg (0.0107 mmol) of 2-hydroxy-5-dibenzosuberone (**61**) was dissolved in 3 mL of a binary solvent system of DCM and c-Hex (1:1, v/v), followed by the addition of equimolar amounts of Ag_3Pz_3 (10.00 mg, 0.0107 mmol). The resulting mixed solution was filtered and then transferred to a 20 mL screw-capped sample vial. The cap of the sample vial was loosely closed to allow the solvent to slowly evaporate at room temperature. The entire co-crystal incubation process was protected from light using aluminum foil. After the designated evaporation period, typically 1-3 days, high-quality colorless plate-shaped crystals suitable for single-crystal X-ray diffraction analysis formed at the bottom of the vial.

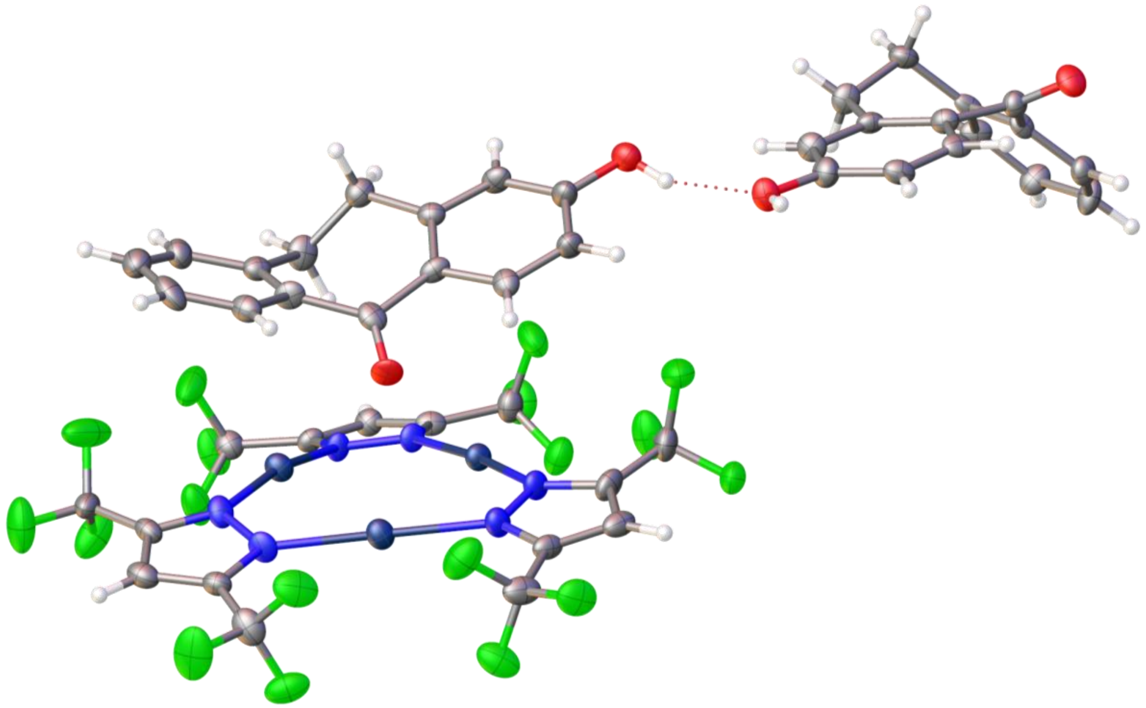

Figure S199. Asymmetric unit of **Ag_3Pz_3·61** (thermal displacement parameters at the 50% probability level).

Table S69. Crystal data and structure refinement for **Ag_3Pz_3·61**

Empirical formula	$C_{45}H_{27}Ag_3F_{18}N_6O_4$
Formula weight	1381.33
Temperature/K	99.98(16)
Crystal system	orthorhombic
Space group	$P2_12_12_1$
a/Å	8.55960(10)
b/Å	9.41130(10)
c/Å	56.4718(6)
α/°	90
β/°	90
γ/°	90
Volume/Å^3	4549.20(9)
Z	4
ρ_{calc}g/cm^3	2.017
μ/mm^{-1}	11.420
F(000)	2696.0
Crystal size/mm^3	0.25 × 0.16 × 0.13
Radiation	Cu Kα (λ = 1.54184)
2θ range for data collection/°	6.26 to 155.57
Index ranges	$-10 \leq h \leq 8$, $-11 \leq k \leq 11$, $-69 \leq l \leq 55$
Reflections collected	21051
Independent reflections	8879 [R_{int} = 0.0436, R_{sigma} = 0.0596]
Data/restraints/parameters	8879/0/688
Goodness-of-fit on F^2	1.068
Final R indexes [I>=2σ (I)]	R_1 = 0.0531, wR_2 = 0.1238
Final R indexes [all data]	R_1 = 0.0568, wR_2 = 0.1256
Largest diff. peak/hole / e Å^{-3}	2.45/-1.15
Flack parameter	0.175(13)
CCDC-number	2501809

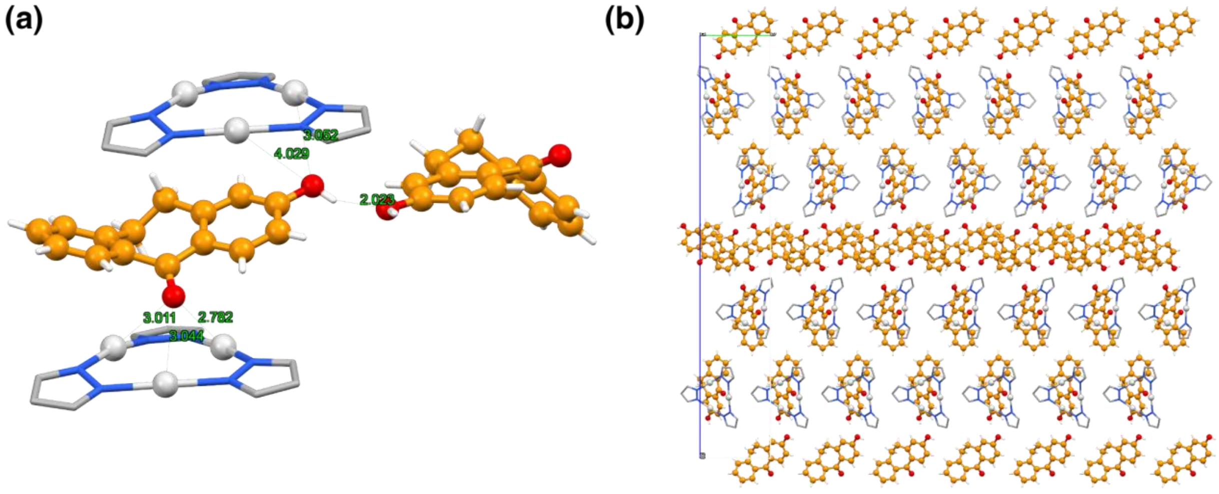

Figure S200. (a) A schematic diagram of the co-crystal structure in the **Ag_3Pz_3·61** single crystal, formed by the guest organic molecule and the surrounding Ag_3Pz_3 units that exhibit significant interactions with it. (b) A 1 × 7 × 1 packing mode in the single crystal structure of **Ag_3Pz_3·61** along the *a* axis. Trifluoromethyl groups and H atoms in Ag_3Pz_3 are omitted for clarity. Ag···O interactions are indicated with green dotted lines with distances in Å. C, N, and Ag atoms in Ag_3Pz_3 are depicted in dark gray, light blue, and light gray, respectively; C, O, and H atoms in **61** are depicted in orange, red, and white, respectively.

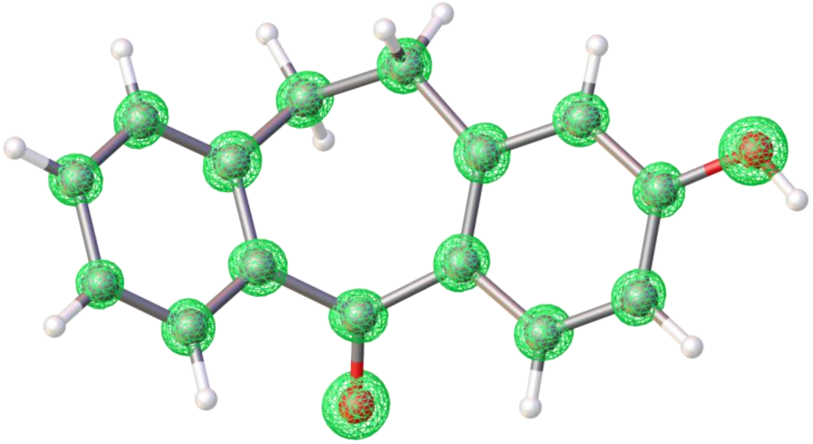

Figure S201. F_{obs} (contour: 1.15) electron density map superimposed on the structure of **61** in the single crystal structure of **Ag_3Pz_3·61**.

Preparation of Ag_3Pz_3·62. 2.23 mg (0.0107 mmol) of anthraquinone (**62**) was dissolved in 3 mL of a binary solvent system of DCM and n-Hex (1:1, v/v), followed by the addition of equimolar amounts of Ag_3Pz_3 (10.00 mg, 0.0107 mmol). The resulting mixed solution was filtered and then transferred to a 20 mL screw-capped sample vial. The cap of the sample vial was loosely closed to allow the solvent to slowly evaporate at room temperature. The entire co-crystal incubation process was protected from light using aluminum foil. After the designated evaporation period, typically 1-3 days, high-quality colorless block-shaped crystals suitable for single-crystal X-ray diffraction analysis formed at the bottom of the vial.

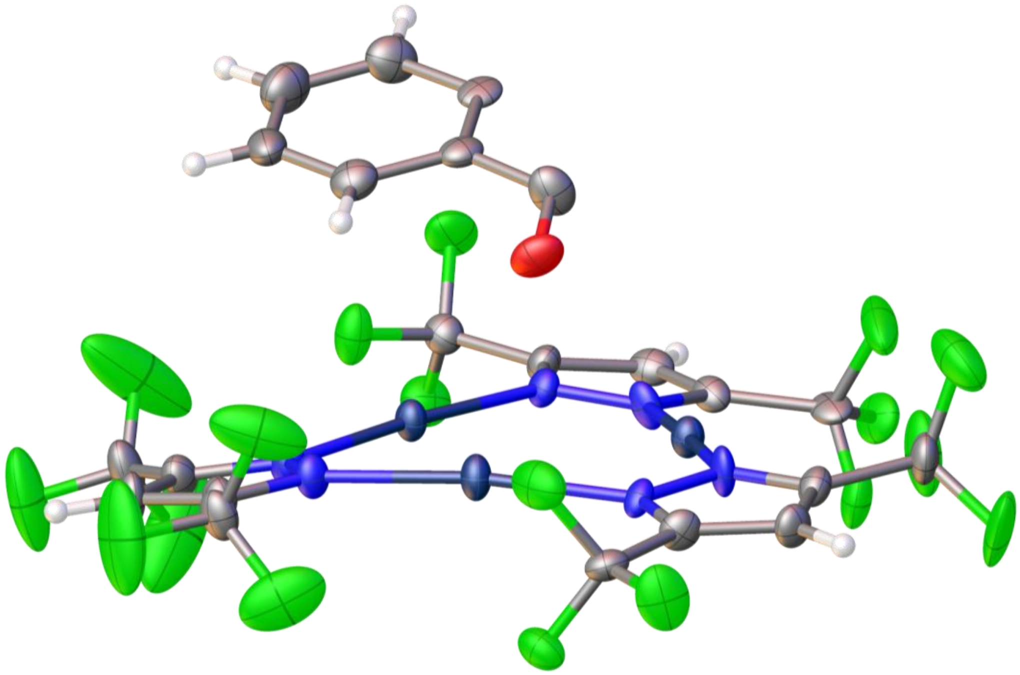

Figure S202. Asymmetric unit of **Ag_3Pz_3·62** (thermal displacement parameters at the 50% probability level).

Table S70. Crystal data and structure refinement for $\mathbf{Ag_3Pz_3 \cdot 62}$

Empirical formula	$C_{22}H_7Ag_3F_{18}N_6O$
Formula weight	1036.95
Temperature/K	100.03(17)
Crystal system	triclinic
Space group	$P\bar{1}$
a/Å	8.8270(3)
b/Å	12.9600(5)
c/Å	14.3646(6)
α/°	103.546(3)
β/°	104.763(3)
γ/°	107.510(3)
Volume/Å^3	1426.69(10)
Z	2
ρ_{calc}g/cm^3	2.414
μ/mm^{-1}	17.788
F(000)	984.0
Crystal size/mm^3	0.165 × 0.156 × 0.147
Radiation	Cu Kα (λ = 1.54184)
2θ range for data collection/°	6.76 to 156.534
Index ranges	$-11 \le h \le 10$, $-16 \le k \le 16$, $-17 \le l \le 15$
Reflections collected	13616
Independent reflections	5769 [R_{int} = 0.0540, R_{sigma} = 0.0651]
Data/restraints/parameters	5769/6/412
Goodness-of-fit on F^2	1.090
Final R indexes [I>=2σ (I)]	R_1 = 0.0724, wR_2 = 0.1770
Final R indexes [all data]	R_1 = 0.0934, wR_2 = 0.1843
Largest diff. peak/hole / e Å^{-3}	2.07/-1.53
CCDC-number	2501810

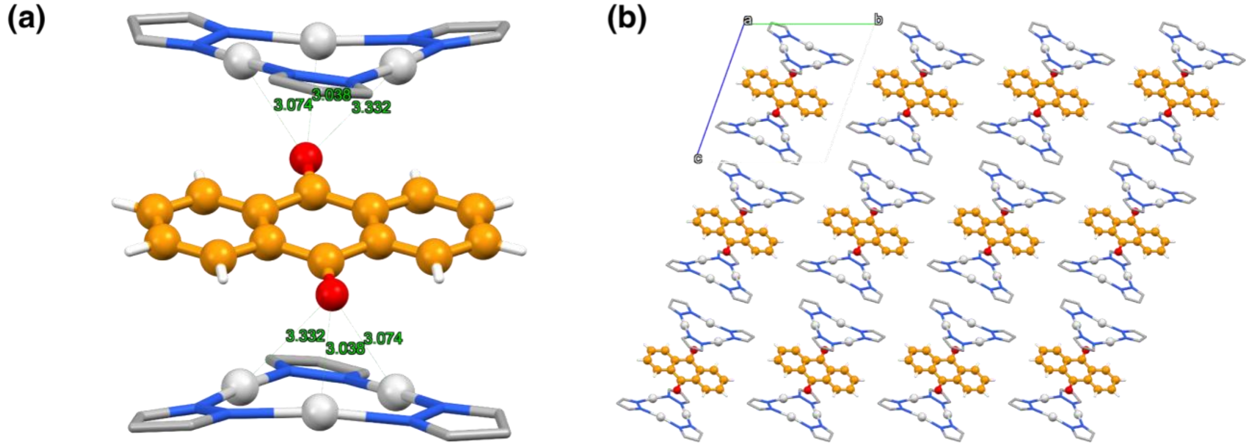

Figure S203. (a) A schematic diagram of the co-crystal structure in the **Ag_3Pz_3·62** single crystal, formed by the guest organic molecule and the surrounding Ag_3Pz_3 units that exhibit significant interactions with it. (b) A 1 × 4 × 3 packing mode in the single crystal structure of **Ag_3Pz_3·62** along the *a* axis. Trifluoromethyl groups and H atoms in Ag_3Pz_3 are omitted for clarity. Ag···O interactions are indicated with green dotted lines with distances in Å. C, N, and Ag atoms in Ag_3Pz_3 are depicted in dark gray, light blue, and light gray, respectively; C, O, and H atoms **62** are depicted in orange, red, and white, respectively.

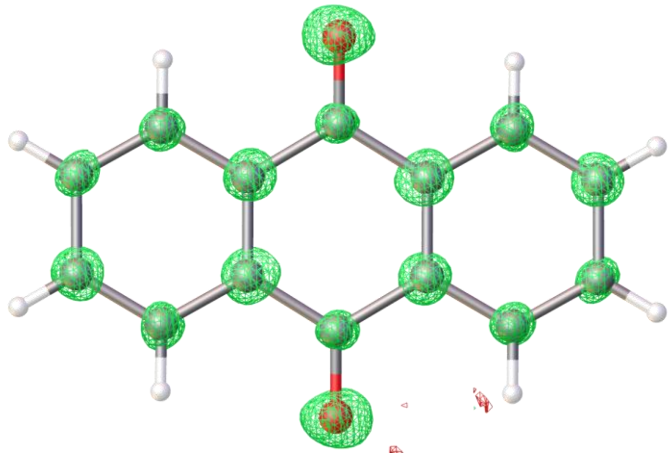

Figure S204. F_{obs} (contour: 1.20) electron density map superimposed on the structure of **62** in the single crystal structure of **Ag_3Pz_3·62**.

Preparation of Ag_3Pz_3·63. 2.40 mg (0.0107 mmol) of 1-hydroxyanthraquinone (**63**) was dissolved in 3 mL of a binary solvent system of DCM and c-Hex (1:1, v/v), followed by the addition of equimolar amounts of Ag_3Pz_3 (10.00 mg, 0.0107 mmol). The resulting mixed solution was filtered and then transferred to a 20 mL screw-capped sample vial. The cap of the sample vial was loosely closed to allow the solvent to slowly evaporate at room temperature. The entire co-crystal incubation process was protected from light using aluminum foil. After the designated evaporation period, typically 1-3 days, high-quality yellow block-shaped crystals suitable for single-crystal X-ray diffraction analysis formed at the bottom of the vial.

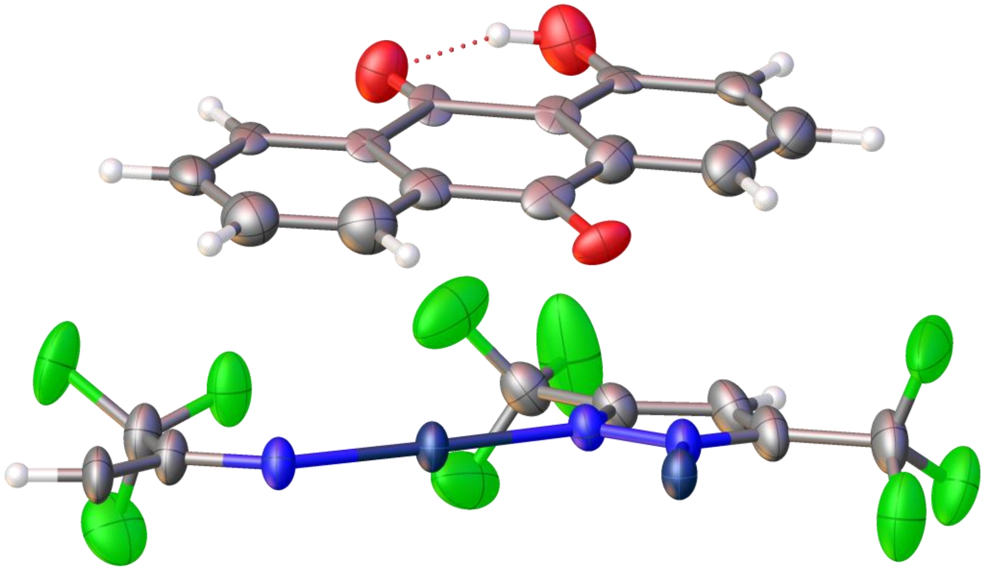

Figure S205. Asymmetric unit of **Ag_3Pz_3·63** (thermal displacement parameters at the 50% probability level).

Table S71. Crystal data and structure refinement for **Ag_3Pz_3·63**

Empirical formula	$C_{29}H_{11}Ag_3F_{18}N_6O_3$
Formula weight	1157.05
Temperature/K	100.15
Crystal system	monoclinic
Space group	*I*2/*a*
a/Å	17.1674(3)
b/Å	13.1306(2)
c/Å	14.9007(2)
α/°	90
β/°	93.6350(10)
γ/°	90
Volume/Å^3	3352.13(9)
Z	4
ρ_{calc}g/cm^3	2.293
μ/mm^{-1}	15.289
F(000)	2216.0
Crystal size/mm^3	0.26 × 0.14 × 0.13
Radiation	Cu Kα (λ = 1.54184)
2θ range for data collection/°	8.484 to 145.428
Index ranges	$-20 \leq h \leq 19$, $-16 \leq k \leq 15$, $-17 \leq l \leq 18$
Reflections collected	12157
Independent reflections	3239 [R_{int} = 0.0207, R_{sigma} = 0.0175]
Data/restraints/parameters	3239/280/320
Goodness-of-fit on F^2	1.063
Final *R* indexes [I>=2σ (I)]	R_1 = 0.0372, wR_2 = 0.1011
Final *R* indexes [all data]	R_1 = 0.0394, wR_2 = 0.1029
Largest diff. peak/hole / e Å^{-3}	1.75/-1.32
CCDC-number	2501811

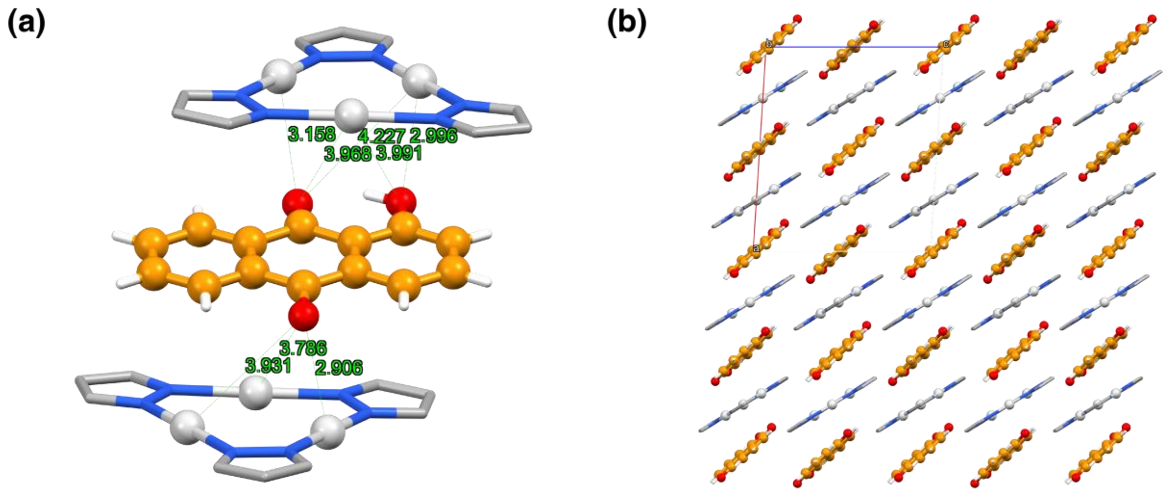

Figure S206. (a) A schematic diagram of the co-crystal structure in the **Ag_3Pz_3·63** single crystal, formed by the guest organic molecule and the surrounding Ag_3Pz_3 units that exhibit significant interactions with it. (b) A 2 × 1 × 2 packing mode in the single crystal structure of **Ag_3Pz_3·63** along the *b* axis. Trifluoromethyl groups and H atoms in Ag_3Pz_3 are omitted for clarity. Ag···O interactions are indicated with green dotted lines with distances in Å. C, N, and Ag atoms in Ag_3Pz_3 are depicted in dark gray, light blue, and light gray, respectively; C, O, and H atoms in **63** are depicted in orange, red, and white, respectively.

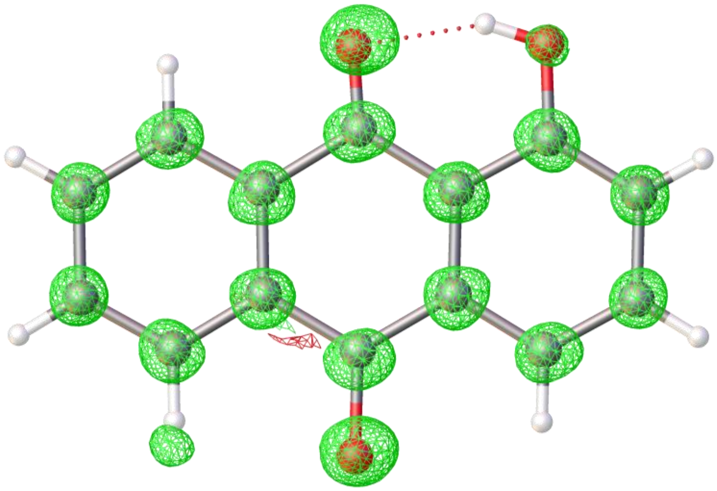

Figure S207. F_{obs} (contour: 0.35) electron density map superimposed on the structure of **63** in the single crystal structure of **Ag_3Pz_3·63**. We believe that the unassigned electron density is attributable to disordered structure.

Preparation of Ag$_3$Pz$_3$·64. 2.40 mg (0.0107 mmol) of 2-hydroxyanthraquinone (**64**) was dissolved in 3 mL of a binary solvent system of DCM and MeOH (1:1, v/v), followed by the addition of equimolar amounts of Ag$_3$Pz$_3$ (10.00 mg, 0.0107 mmol). The resulting mixed solution was filtered and then transferred to a 20 mL screw-capped sample vial. The cap of the sample vial was loosely closed to allow the solvent to slowly evaporate at room temperature. The entire co-crystal incubation process was protected from light using aluminum foil. After the designated evaporation period, typically 1-3 days, high-quality yellow needle-shaped crystals suitable for single-crystal X-ray diffraction analysis formed at the bottom of the vial.

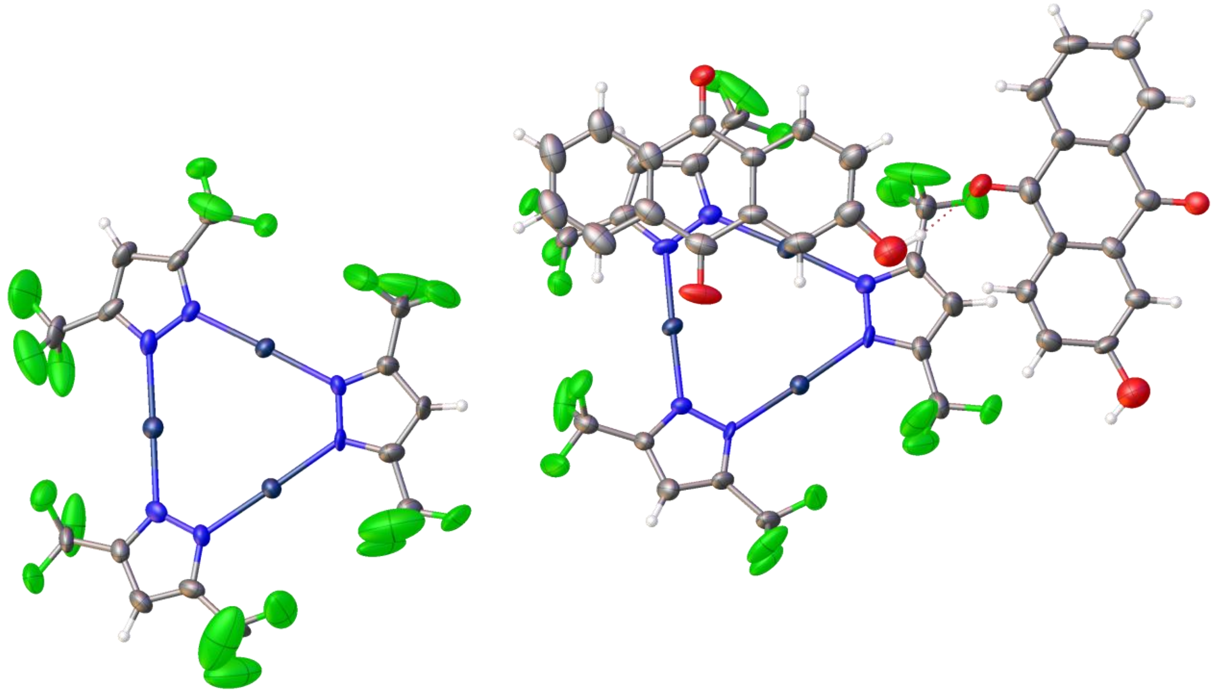

Figure S208. Asymmetric unit of **Ag$_3$Pz$_3$·64** (thermal displacement parameters at the 50% probability level).

Table S72. Crystal data and structure refinement for $\mathbf{Ag_3Pz_3 \cdot 64}$

Empirical formula	$C_{58}H_{22}Ag_6F_{36}N_{12}O_6$
Formula weight	2314.09
Temperature/K	99.98(15)
Crystal system	monoclinic
Space group	$P2_1$
a/Å	13.2452(2)
b/Å	11.4403(2)
c/Å	22.7510(3)
α/°	90
β/°	90.4980(10)
γ/°	90
Volume/Å^3	3447.31(9)
Z	2
ρ_{calc}g/cm^3	2.229
μ/mm^{-1}	14.867
F(000)	2216.0
Crystal size/mm^3	0.18 × 0.16 × 0.14
Radiation	Cu Kα (λ = 1.54184)
2θ range for data collection/°	6.674 to 155.862
Index ranges	$-13 \leq h \leq 16$, $-14 \leq k \leq 14$, $-28 \leq l \leq 27$
Reflections collected	27312
Independent reflections	12237 [R_{int} = 0.0360, R_{sigma} = 0.0480]
Data/restraints/parameters	12237/194/1060
Goodness-of-fit on F^2	1.027
Final R indexes [I>=2σ (I)]	R_1 = 0.0567, wR_2 = 0.1365
Final R indexes [all data]	R_1 = 0.0654, wR_2 = 0.1442
Largest diff. peak/hole / e Å^{-3}	1.75/-1.04
Flack parameter	0.470(15)
CCDC-number	2501812

Responses to CheckCIF alerts for Ag_3Pz_3·64 crystal structure:

(There is no A-level alert)

B-level alerts:

"Low Bond Precision on C-C Bonds .... 0.02321 Ang."

Disordered structure.

"Coordinates do not Form a Properly Connected Set Please Do !"

The alert is due to a large number of co-crystallized molecules in the which sometimes do not show as a connected set. This is acceptable from a crystallographic point of view.

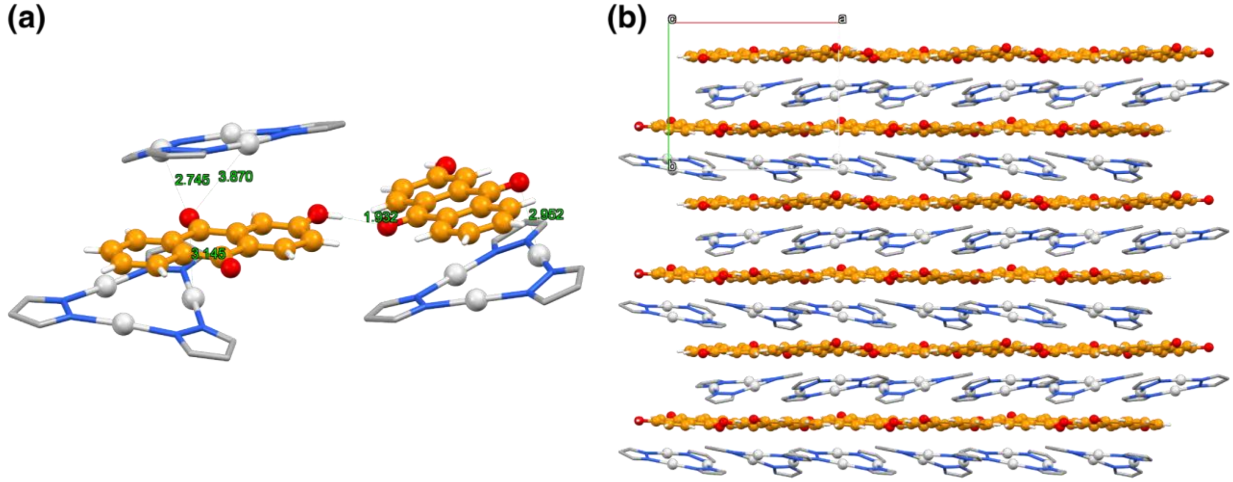

Figure S209. (a) A schematic diagram of the co-crystal structure in the **Ag_3Pz_3·64** single crystal, formed by the guest organic molecule and the surrounding Ag_3Pz_3 units that exhibit significant interactions with it. (b) A 3 × 3 × 1 packing mode in the single crystal structure of **Ag_3Pz_3·64** along the *c* axis. Trifluoromethyl groups and H atoms in Ag_3Pz_3 are omitted for clarity. Ag···O and O-H···O interactions are indicated with green dotted lines with distances in Å. C, N, and Ag atoms in Ag_3Pz_3 are depicted in dark gray, light blue, and light gray, respectively; C, O, and H atoms in **64** are depicted in orange, red, and white, respectively.

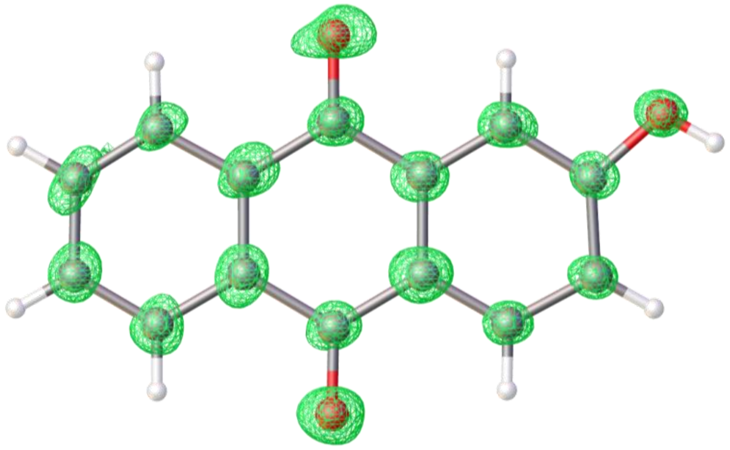

Figure S210. F_{obs} (contour: 0.55) electron density map superimposed on the structure of **64** in the single crystal structure of **Ag_3Pz_3·64**.

Preparation of Ag_3Pz_3·65. 3.17 mg (0.0107 mmol) of cryptotanshinone (**65**) was dissolved in 3 mL of n-Hex, followed by the addition of equimolar amounts of Ag_3Pz_3 (10.00 mg, 0.0107 mmol). The resulting mixed solution was filtered and then transferred to a 20 mL screw-capped sample vial. The cap of the sample vial was loosely closed to allow the solvent to slowly evaporate at room temperature. The entire co-crystal incubation process was protected from light using aluminum foil. After the designated evaporation period, typically 1-3 days, high-quality brown needle-shaped crystals suitable for single-crystal X-ray diffraction analysis formed at the bottom of the vial.

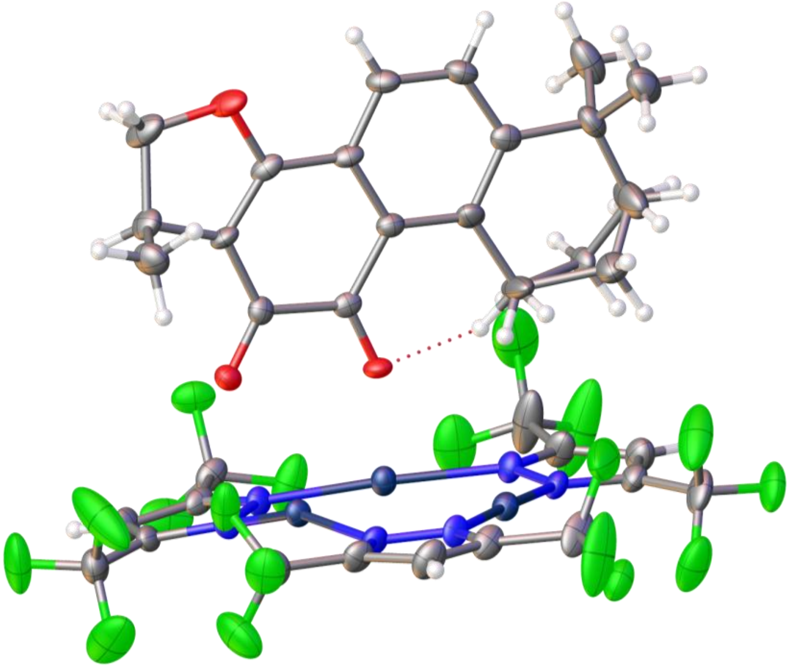

Figure S211. Asymmetric unit of **Ag_3Pz_3·65** (thermal displacement parameters at the 50% probability level).

Table S73. Crystal data and structure refinement for **Ag_3Pz_3·65**

Empirical formula	$C_{34}H_{23}Ag_3F_{18}N_6O_3$
Formula weight	1229.19
Temperature/K	100.15
Crystal system	orthorhombic
Space group	$P2_12_12_1$
a/Å	9.38490(10)
b/Å	12.78620(10)
c/Å	32.9001(3)
α/°	90
β/°	90
γ/°	90
Volume/Å^3	3947.92(6)
Z	4
ρ_{calc}g/cm^3	2.068
μ/mm^{-1}	13.028
F(000)	2384.0
Crystal size/mm^3	0.18 × 0.164 × 0.14
Radiation	Cu Kα (λ = 1.54184)
2θ range for data collection/°	7.418 to 155.61
Index ranges	$-6 \leq h \leq 11$, $-14 \leq k \leq 15$, $-41 \leq l \leq 39$
Reflections collected	18632
Independent reflections	7817 [R_{int} = 0.0296, R_{sigma} = 0.0408]
Data/restraints/parameters	7817/7/589
Goodness-of-fit on F^2	1.048
Final R indexes [I>=2σ (I)]	R_1 = 0.0324, wR_2 = 0.0682
Final R indexes [all data]	R_1 = 0.0363, wR_2 = 0.0694
Largest diff. peak/hole / e Å^{-3}	0.80/-0.60
Flack parameter	-0.020(5)
CCDC-number	2501813

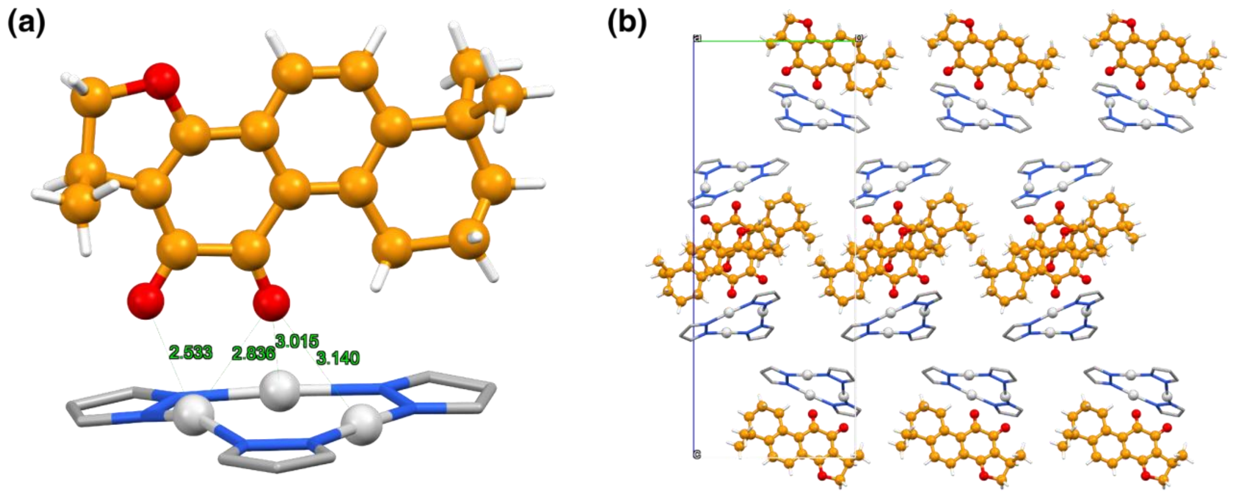

Figure S212. (a) A schematic diagram of the co-crystal structure in the **Ag_3Pz_3·65** single crystal, formed by the guest organic molecule and the surrounding Ag_3Pz_3 units that exhibit significant interactions with it. (b) A 1 × 3 × 1 packing mode in the single crystal structure of **Ag_3Pz_3·65** along the *a* axis. Trifluoromethyl groups and H atoms in Ag_3Pz_3 are omitted for clarity. Ag···O interactions are indicated with green dotted lines with distances in Å. C, N, and Ag atoms in Ag_3Pz_3 are depicted in dark gray, light blue, and light gray, respectively; C, O, and H atoms in **65** are depicted in orange, red, and white, respectively.

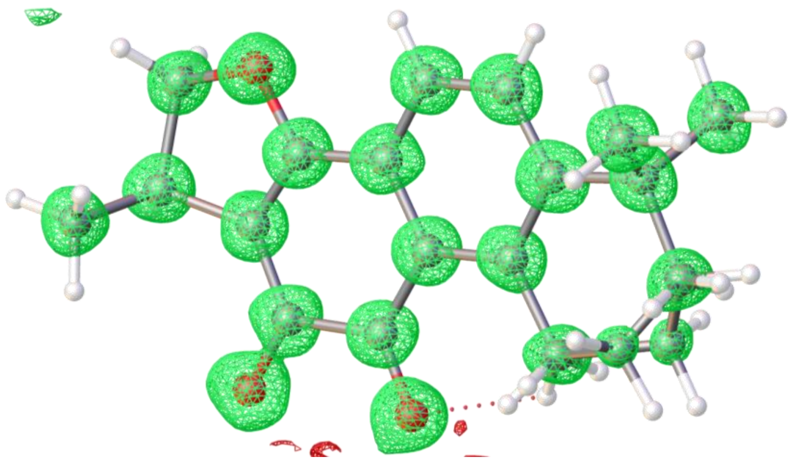

Figure S213. F_{obs} (contour: 0.60) electron density map superimposed on the structure of **65** in the single crystal structure of **Ag_3Pz_3·65**. Please note that a twofold positional disorder is present at certain atomic sites of molecule **65**, likely due to weak intra-/intermolecular interactions and local steric effects.

Preparation of Ag_3Pz_3·66. 1.84 mg (0.0107 mmol) of ethyl diacetoacetate (**66**) was dissolved in 3 mL of a binary solvent system of DCM and n-Hex (1:1, v/v), followed by the addition of equimolar amounts of Ag_3Pz_3 (10.00 mg, 0.0107 mmol). The resulting mixed solution was filtered and then transferred to a 20 mL screw-capped sample vial. The cap of the sample vial was loosely closed to allow the solvent to slowly evaporate at room temperature. The entire co-crystal incubation process was protected from light using aluminum foil. After the designated evaporation period, typically 1-3 days, high-quality colorless needle-shaped crystals suitable for single-crystal X-ray diffraction analysis formed at the bottom of the vial.

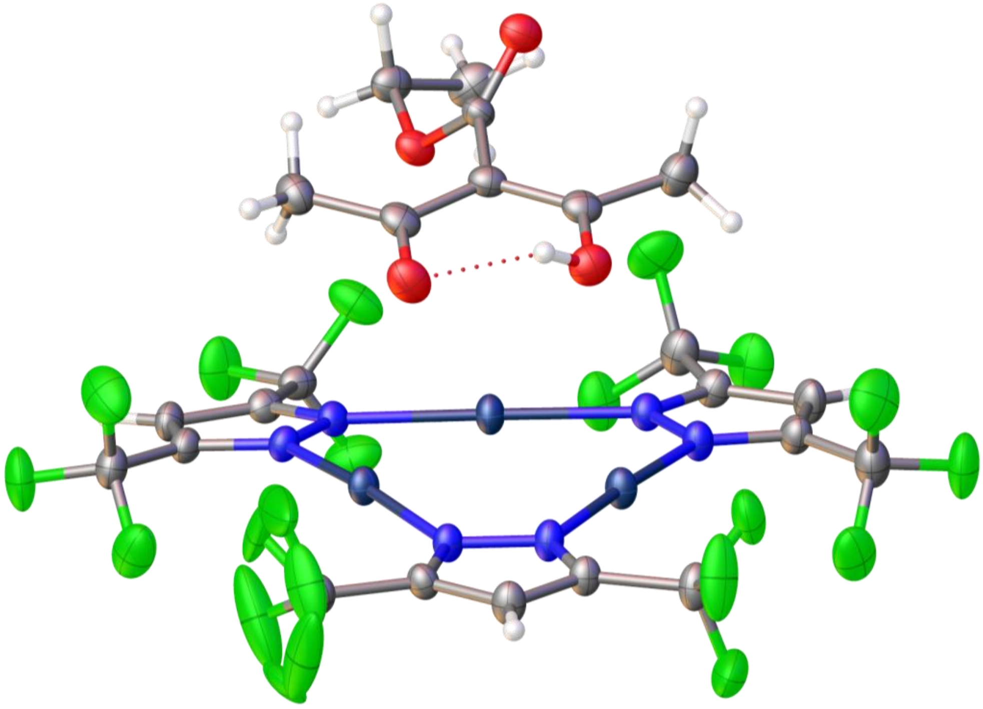

Figure S214. Asymmetric unit of **Ag_3Pz_3·66** (thermal displacement parameters at the 50% probability level).

Table S74. Crystal data and structure refinement for **Ag_3Pz_3·66**

Empirical formula	$C_{23}H_{15}Ag_3F_{18}N_6O_4$
Formula weight	1105.02
Temperature/K	100.15
Crystal system	monoclinic
Space group	$P2_1/n$
a/Å	7.88340(10)
b/Å	17.4573(3)
c/Å	23.7253(3)
α/°	90
β/°	91.9790(10)
γ/°	90
Volume/Å^3	3263.20(8)
Z	4
ρ_{calc}g/cm^3	2.249
μ/mm^{-1}	15.677
F(000)	2120.0
Crystal size/mm^3	0.15 × 0.14 × 0.12
Radiation	Cu Kα (λ = 1.54184)
2θ range for data collection/°	6.288 to 149
Index ranges	$-9 \leq h \leq 6$, $-20 \leq k \leq 21$, $-29 \leq l \leq 29$
Reflections collected	21092
Independent reflections	6377 [R_{int} = 0.0507, R_{sigma} = 0.0307]
Data/restraints/parameters	6377/54/517
Goodness-of-fit on F^2	1.027
Final R indexes [I>=2σ (I)]	R_1 = 0.0399, wR_2 = 0.0876
Final R indexes [all data]	R_1 = 0.0462, wR_2 = 0.0915
Largest diff. peak/hole / e Å^{-3}	0.77/-1.29
CCDC-number	2501814

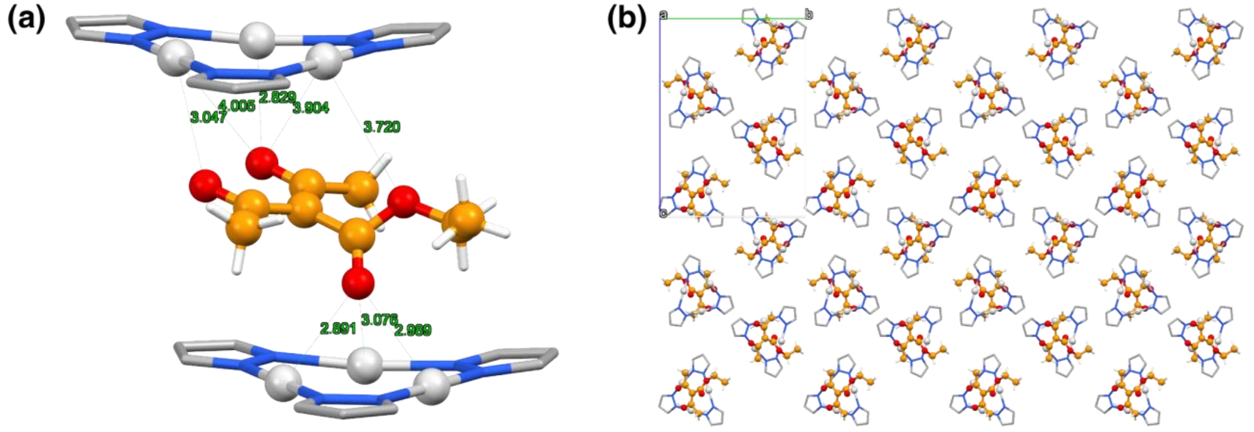

Figure S215. (a) A schematic diagram of the co-crystal structure in the **Ag_3Pz_3·66** single crystal, formed by the guest organic molecule and the surrounding Ag_3Pz_3 units that exhibit significant interactions with it. (b) A 1 × 4 × 2 packing mode in the single crystal structure of **Ag_3Pz_3·66** along the *a* axis. Trifluoromethyl groups and H atoms in Ag_3Pz_3 are omitted for clarity. Ag···O interactions are indicated with green dotted lines with distances in Å. C, N, and Ag atoms in Ag_3Pz_3 are depicted in dark gray, light blue, and light gray, respectively; C, O, and H atoms in **66** are depicted in orange, red, and white, respectively.

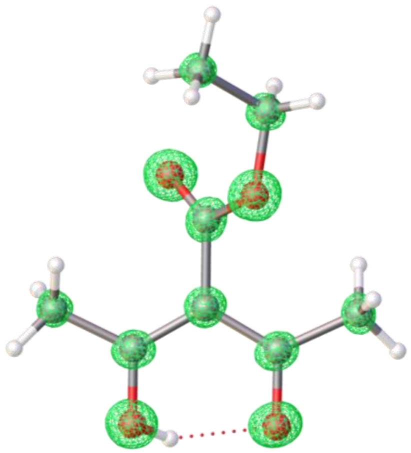

Figure S216. F_{obs} (contour: 0.90) electron density map superimposed on the structure of **66** in the single crystal structure of **Ag_3Pz_3·66**.

Preparation of Ag_3Pz_3·67. 3.15 mg (0.0107 mmol) of pongamol (**67**) was dissolved in 3 mL of a binary solvent system of DCM and n-Hex (1:1, v/v), followed by the addition of equimolar amounts of Ag_3Pz_3 (10.00 mg, 0.0107 mmol). The resulting mixed solution was filtered and then transferred to a 20 mL screw-capped sample vial. The cap of the sample vial was loosely closed to allow the solvent to slowly evaporate at room temperature. The entire co-crystal incubation process was protected from light using aluminum foil. After the designated evaporation period, typically 1-3 days, high-quality colorless block-shaped crystals suitable for single-crystal X-ray diffraction analysis formed at the bottom of the vial.

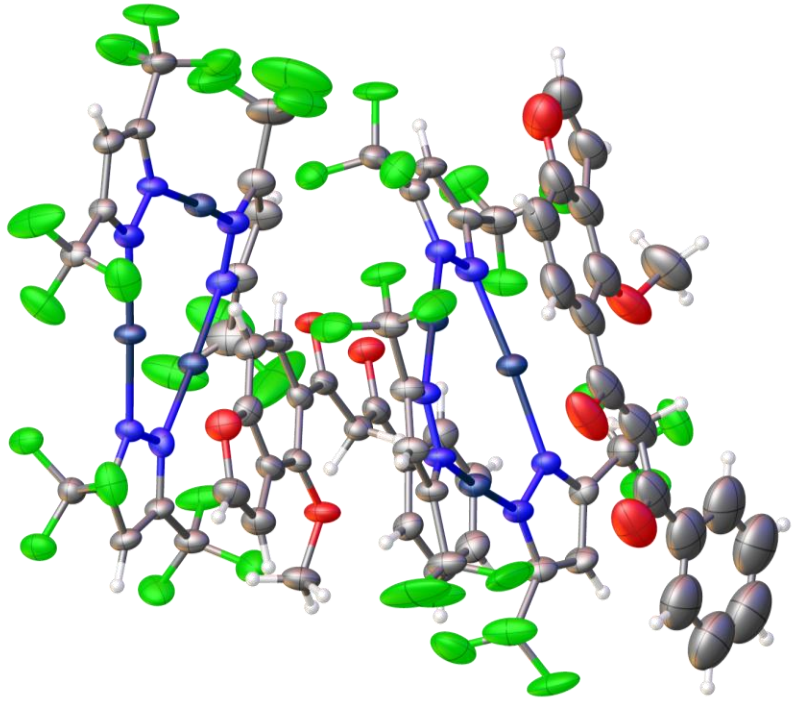

Figure S217. Asymmetric unit of **Ag_3Pz_3·67** (thermal displacement parameters at the 50% probability level).

Table S75. Crystal data and structure refinement for **Ag_3Pz_3·67**

Empirical formula	$C_{33}H_{17}Ag_3F_{18}N_6O_4$
Formula weight	1227.14
Temperature/K	100.00(10)
Crystal system	triclinic
Space group	$P\bar{1}$
a/Å	12.9257(2)
b/Å	13.2495(2)
c/Å	29.9273(5)
α/°	79.9820(10)
β/°	79.4990(10)
γ/°	60.911(2)
Volume/Å^3	4382.05(14)
Z	4
ρ_{calc}g/cm^3	1.860
μ/mm^{-1}	11.757
F(000)	2368.0
Crystal size/mm^3	0.23 × 0.17 × 0.16
Radiation	Cu Kα (λ = 1.54184)
2θ range for data collection/°	7.674 to 147.108
Index ranges	$-15 \le h \le 15$, $-16 \le k \le 16$, $-28 \le l \le 36$
Reflections collected	82236
Independent reflections	16439 [R_{int} = 0.0428, R_{sigma} = 0.0356]
Data/restraints/parameters	16439/128/1155
Goodness-of-fit on F^2	1.051
Final R indexes [I>=2σ (I)]	R_1 = 0.0559, wR_2 = 0.1399
Final R indexes [all data]	R_1 = 0.0653, wR_2 = 0.1450
Largest diff. peak/hole / e Å^{-3}	2.30/-1.12
CCDC-number	2501815

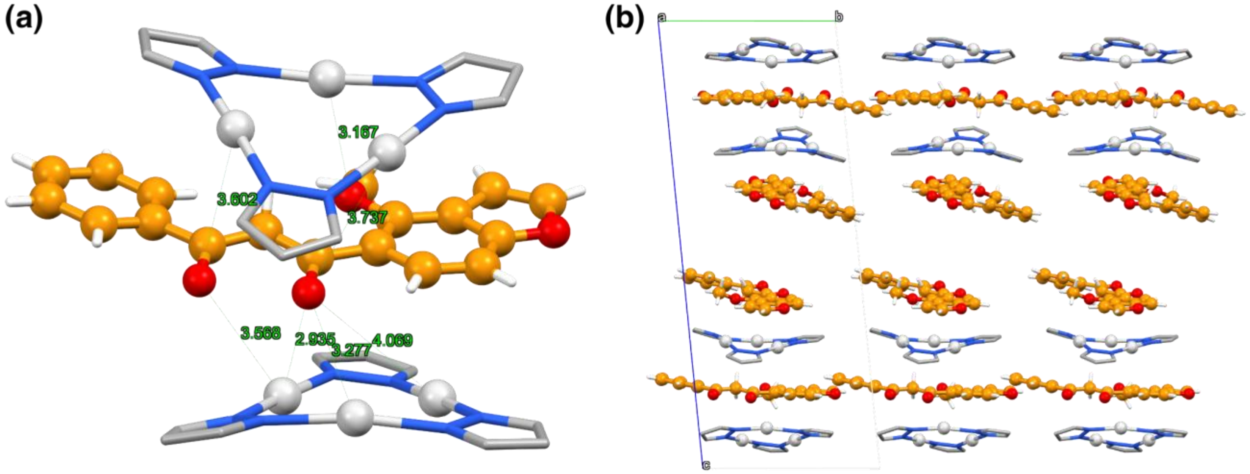

Figure S218. (a) A schematic diagram of the co-crystal structure in the **Ag_3Pz_3·67** single crystal, formed by the guest organic molecule and the surrounding Ag_3Pz_3 units that exhibit significant interactions with it. (b) A 1 × 3 × 1 packing mode in the single crystal structure of **Ag_3Pz_3·67** along the *a* axis. Trifluoromethyl groups and H atoms in Ag_3Pz_3 are omitted for clarity. Ag···O interactions are indicated with green dotted lines with distances in Å. C, N, and Ag atoms in Ag_3Pz_3 are depicted in dark gray, light blue, and light gray, respectively; C, O, and H atoms in **67** are depicted in orange, red, and white, respectively.

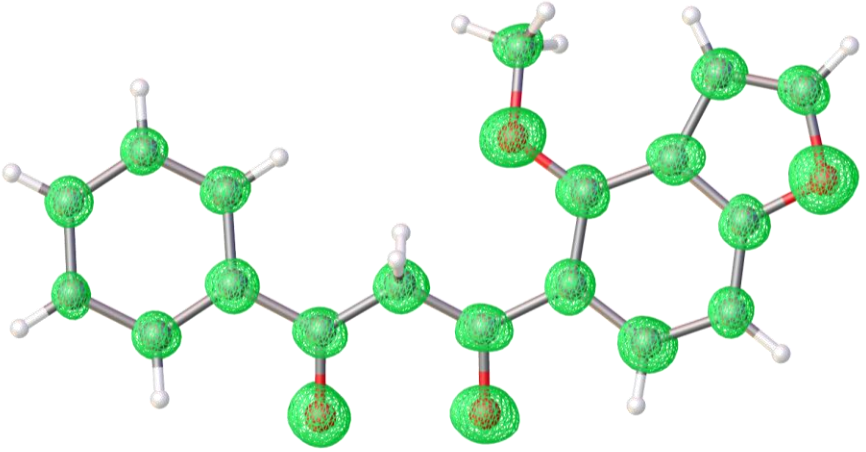

Figure S219. F_{obs} (contour: 0.55) electron density map superimposed on the structure of **67** in the single crystal structure of **Ag_3Pz_3·67**.

Preparation of Ag_3Pz_3·68. 3.21 mg (0.0107 mmol) of adrenosterone (**68**) was dissolved in 3 mL of a binary solvent system of DCM and n-Hex (1:1, v/v), followed by the addition of equimolar amounts of Ag_3Pz_3 (10.00 mg, 0.0107 mmol). The resulting mixed solution was filtered and then transferred to a 20 mL screw-capped sample vial. The cap of the sample vial was loosely closed to allow the solvent to slowly evaporate at room temperature. The entire co-crystal incubation process was protected from light using aluminum foil. After the designated evaporation period, typically 1-3 days, high-quality colorless block-shaped crystals suitable for single-crystal X-ray diffraction analysis formed at the bottom of the vial.

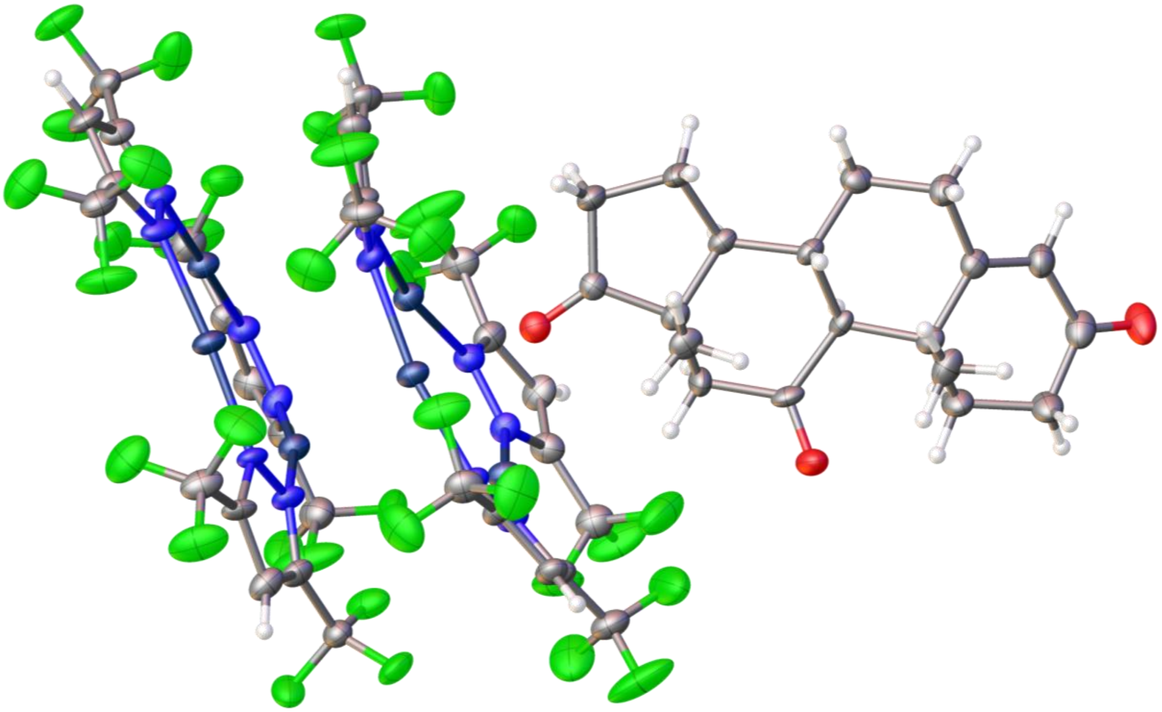

Figure S220. Asymmetric unit of **Ag_3Pz_3·68** (thermal displacement parameters at the 50% probability level).

Table S76. Crystal data and structure refinement for **Ag_3Pz_3·68**

Empirical formula	$C_{49}H_{30}Ag_6F_{36}N_{12}O_3$
Formula weight	2166.07
Temperature/K	100.0(2)
Crystal system	orthorhombic
Space group	$P2_12_12_1$
a/Å	13.36410(10)
b/Å	14.75060(10)
c/Å	32.2331(3)
α/°	90
β/°	90
γ/°	90
Volume/Å^3	6354.06(9)
Z	4
ρ_{calc}g/cm^3	2.264
μ/mm^{-1}	16.024
F(000)	4152.0
Crystal size/mm^3	0.21 × 0.14 × 0.13
Radiation	Cu Kα (λ = 1.54184)
2θ range for data collection/°	5.484 to 157.464
Index ranges	$-15 \leq h \leq 16$, $-16 \leq k \leq 18$, $-40 \leq l \leq 39$
Reflections collected	35108
Independent reflections	12673 [R_{int} = 0.0308, R_{sigma} = 0.0353]
Data/restraints/parameters	12673/0/958
Goodness-of-fit on F^2	1.130
Final R indexes [I>=2σ (I)]	R_1 = 0.0347, wR_2 = 0.0911
Final R indexes [all data]	R_1 = 0.0370, wR_2 = 0.0923
Largest diff. peak/hole / e Å^{-3}	0.86/-1.00
Flack parameter	-0.011(9)
CCDC-number	2501816

Responses to CheckCIF alert for Ag_3Pz_3·68 crystal structure:

(There is no A-level alert)

B-level alert:

"Coordinates do not Form a Properly Connected Set   Please Do !"

The alert is due to a large number of co-crystallized molecules in the which sometimes do not show as a connected set. This is acceptable from a crystallographic point of view.

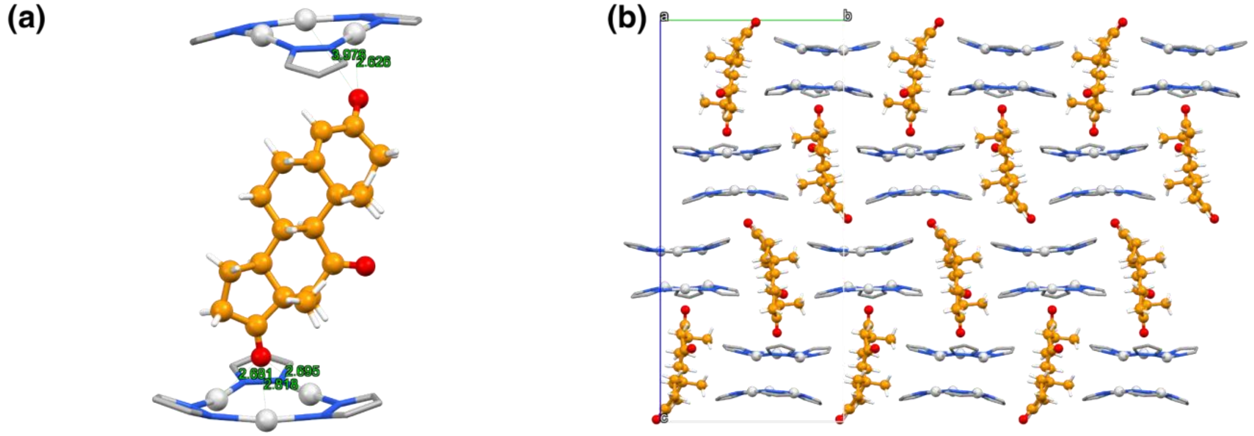

Figure S221. (a) A schematic diagram of the co-crystal structure in the **Ag_3Pz_3·68** single crystal, formed by the guest organic molecule and the surrounding Ag_3Pz_3 units that exhibit significant interactions with it. (b) A 1 × 3 × 1 packing mode in the single crystal structure of **Ag_3Pz_3·68** along the *a* axis. Trifluoromethyl groups and H atoms in Ag_3Pz_3 are omitted for clarity. Ag···O interactions are indicated with green dotted lines with distances in Å. C, N, and Ag atoms in Ag_3Pz_3 are depicted in dark gray, light blue, and light gray, respectively; C, O, and H atoms in **68** are depicted in orange, red, and white, respectively.

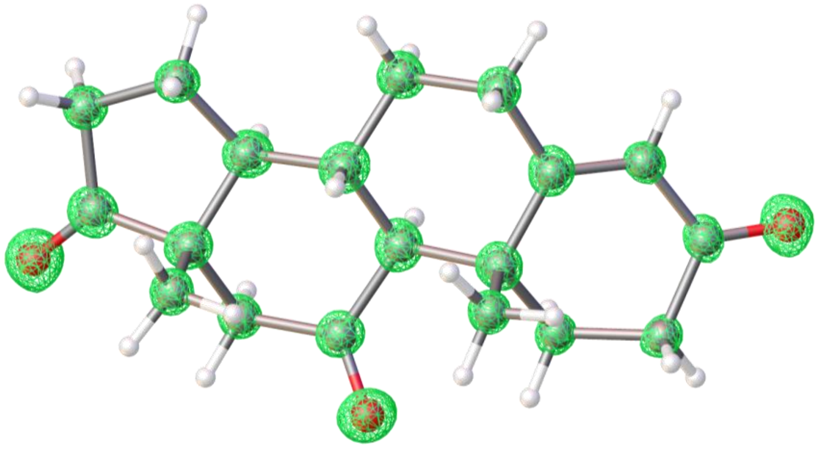

Figure S222. F_{obs} (contour: 0.35) electron density map superimposed on the structure of **68** in the single crystal structure of **Ag_3Pz_3·68**.

Preparation of Ag_3Pz_3·69. 3.79 mg (0.0107 mmol) of triacetylresveratrol (**69**) was dissolved in 3 mL of a binary solvent system of DCM and n-Hex (1:1, v/v), followed by the addition of equimolar amounts of Ag_3Pz_3 (10.00 mg, 0.0107 mmol). The resulting mixed solution was filtered and then transferred to a 20 mL screw-capped sample vial. The cap of the sample vial was loosely closed to allow the solvent to slowly evaporate at room temperature. The entire co-crystal incubation process was protected from light using aluminum foil. After the designated evaporation period, typically 1-3 days, high-quality colorless needle-shaped crystals suitable for single-crystal X-ray diffraction analysis formed at the bottom of the vial.

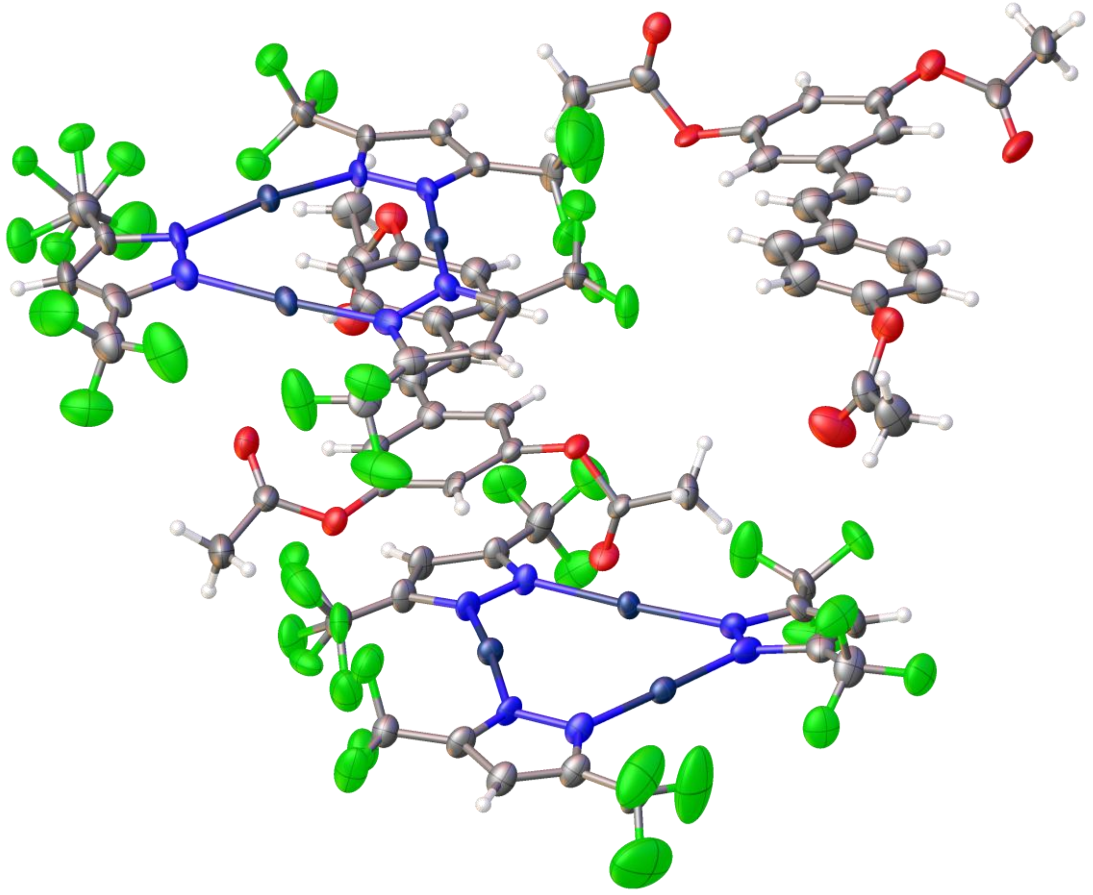

Figure S223. Asymmetric unit of **Ag_3Pz_3·69** (thermal displacement parameters at the 50% probability level).

Table S77. Crystal data and structure refinement for **Ag_3Pz_3·69**

Empirical formula	$C_{70}H_{42}Ag_6F_{36}N_{12}O_{12}$
Formula weight	2574.37
Temperature/K	100.0(3)
Crystal system	monoclinic
Space group	$P2_1/n$
a/Å	18.2200(6)
b/Å	16.9582(5)
c/Å	27.3909(7)
α/°	90
β/°	96.637(3)
γ/°	90
Volume/Å^3	8406.5(4)
Z	4
ρ_{calc}g/cm^3	2.034
μ/mm^{-1}	12.332
F(000)	4992.0
Crystal size/mm^3	0.16 × 0.14 × 0.13
Radiation	Cu Kα (λ = 1.54184)
2θ range for data collection/°	6.17 to 149.984
Index ranges	$-22 \le h \le 13$, $-20 \le k \le 15$, $-33 \le l \le 34$
Reflections collected	38944
Independent reflections	16714 [R_{int} = 0.0945, R_{sigma} = 0.0990]
Data/restraints/parameters	16714/673/1171
Goodness-of-fit on F^2	1.144
Final R indexes [I>=2σ (I)]	R_1 = 0.1304, wR_2 = 0.2980
Final R indexes [all data]	R_1 = 0.1769, wR_2 = 0.3152
Largest diff. peak/hole / e Å^{-3}	2.06/-1.66
CCDC-number	2501817

Responses to CheckCIF alerts for Ag_3Pz_3·69 crystal structure:

(There is no A-level alert)

B-level alerts:

"Low Bond Precision on C-C Bonds .... 0.02098 Ang."

Disordered structure.

"Coordinates do not Form a Properly Connected Set Please Do !"

The alert is due to a large number of co-crystallized molecules in the which sometimes do not show as a connected set. This is acceptable from a crystallographic point of view.

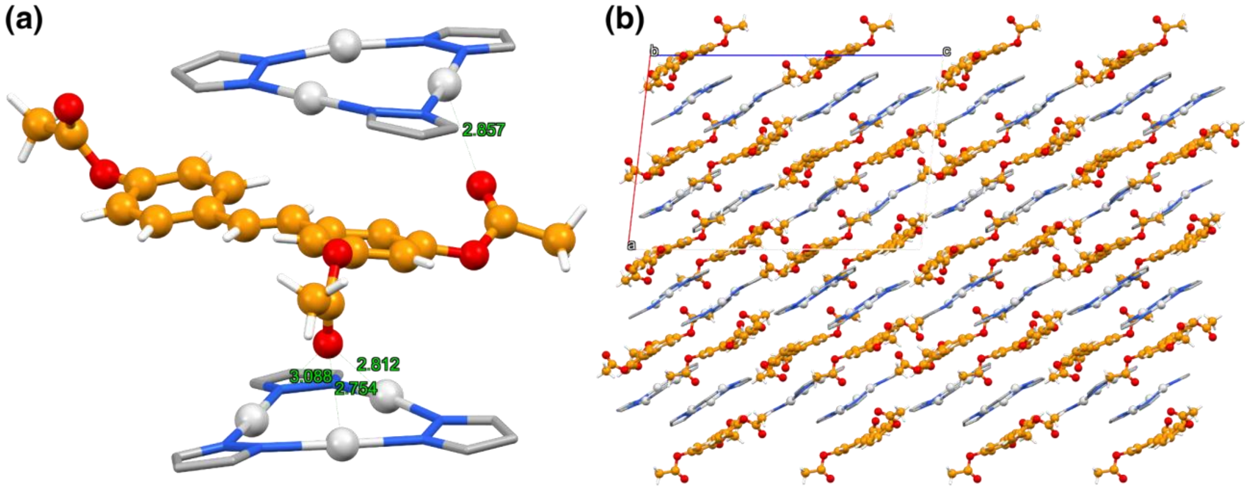

Figure S224. (a) A schematic diagram of the co-crystal structure in the **Ag_3Pz_3·69** single crystal, formed by the guest organic molecule and the surrounding Ag_3Pz_3 units that exhibit significant interactions with it. (b) A 2 × 1 × 2 packing mode in the single crystal structure of **Ag_3Pz_3·69** along the *b* axis. Trifluoromethyl groups and H atoms in Ag_3Pz_3 are omitted for clarity. Ag···O interactions are indicated with green dotted lines with distances in Å. C, N, and Ag atoms in Ag_3Pz_3 are depicted in dark gray, light blue, and light gray, respectively; C, O, and H atoms in **69** are depicted in orange, red, and white, respectively.

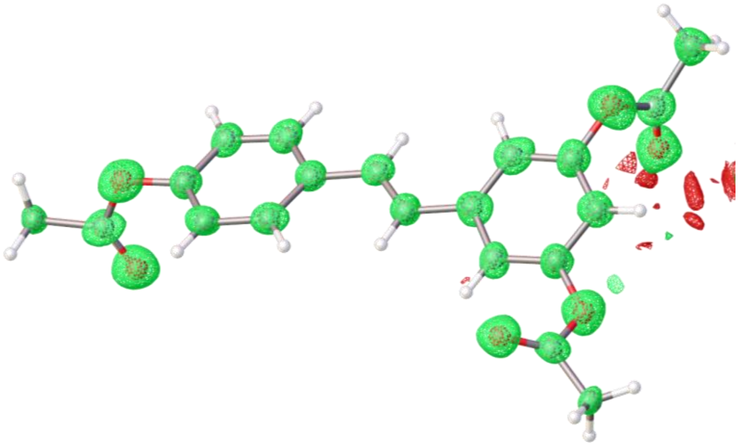

Figure S225. F_{obs} (contour: 0.44) electron density map superimposed on the structure of **69** in the single crystal structure of **Ag_3Pz_3·69**. We believe that the unassigned electron density is attributable to residual solvent molecules and the Ag_3Pz_3 units.

Preparation of Ag_3Pz_3·70. 1.76 mg (0.0107 mmol) of 2,6-adamantanedione (**70**) was dissolved in 3 mL of a binary solvent system of DCM and c-Hex (1:1, v/v), followed by the addition of equimolar amounts of Ag_3Pz_3 (10.00 mg, 0.0107 mmol). The resulting mixed solution was filtered and then transferred to a 20 mL screw-capped sample vial. The cap of the sample vial was loosely closed to allow the solvent to slowly evaporate at room temperature. The entire co-crystal incubation process was protected from light using aluminum foil. After the designated evaporation period, typically 1-3 days, high-quality colorless block-shaped crystals suitable for single-crystal X-ray diffraction analysis formed at the bottom of the vial.

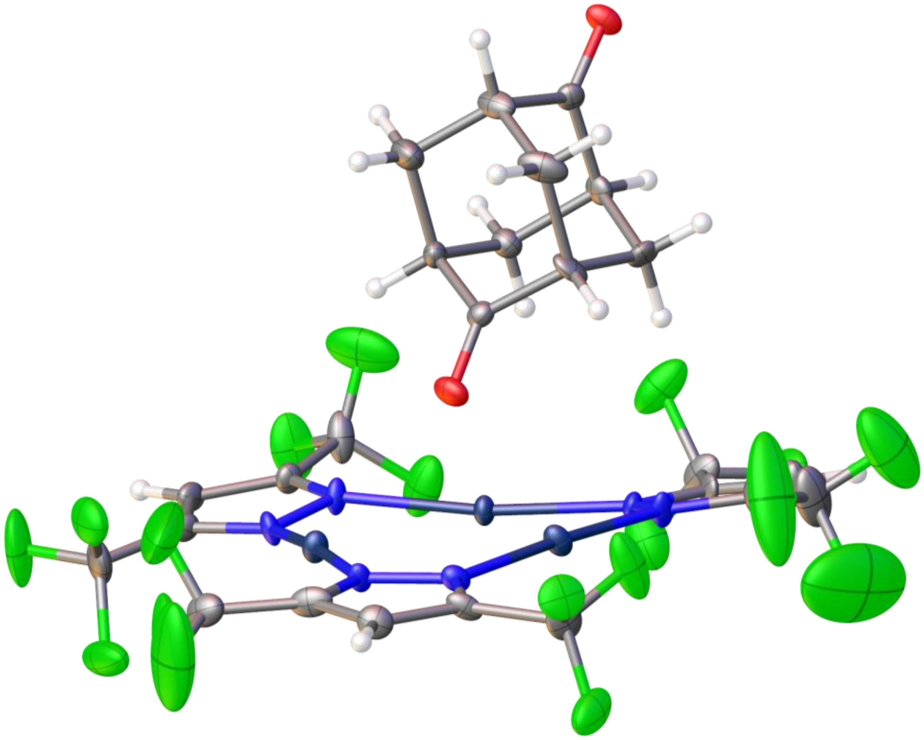

Figure S226. Asymmetric unit of **Ag_3Pz_3·70** (thermal displacement parameters at the 50% probability level).

Table S78. Crystal data and structure refinement for **Ag_3Pz_3·70**

Empirical formula	$C_{25}H_{15}Ag_3F_{18}N_6O_2$
Formula weight	1097.04
Temperature/K	100.00(10)
Crystal system	monoclinic
Space group	*Pc*
a/Å	9.48700(10)
b/Å	9.35520(10)
c/Å	18.48030(10)
α/°	90
β/°	95.1310(10)
γ/°	90
Volume/Å^3	1633.61(3)
Z	2
ρ_{calc}g/cm^3	2.230
μ/mm^{-1}	15.606
F(000)	1052.0
Crystal size/mm^3	0.23 × 0.21 × 0.16
Radiation	Cu Kα (λ = 1.54184)
2θ range for data collection/°	10.608 to 156.496
Index ranges	$-12 \le h \le 11$, $-9 \le k \le 11$, $-12 \le l \le 22$
Reflections collected	8558
Independent reflections	4527 [R_{int} = 0.0224, R_{sigma} = 0.0237]
Data/restraints/parameters	4527/56/488
Goodness-of-fit on F^2	1.070
Final R indexes [I>=2σ (I)]	R_1 = 0.0376, wR_2 = 0.1018
Final R indexes [all data]	R_1 = 0.0384, wR_2 = 0.1066
Largest diff. peak/hole / e Å^{-3}	1.92/-1.09
Flack parameter	0.126(15)
CCDC-number	2501819

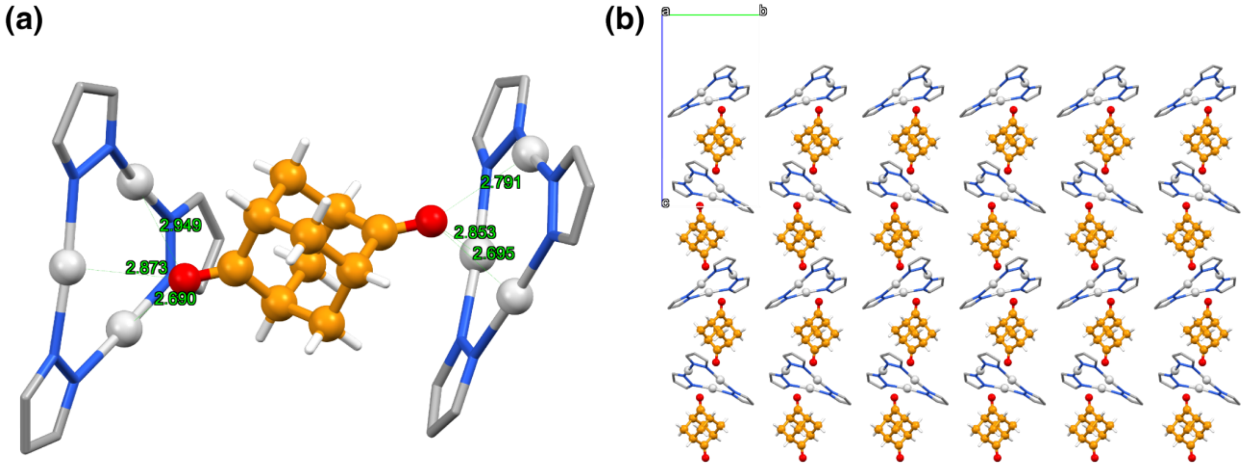

Figure S227. (a) A schematic diagram of the co-crystal structure in the **Ag_3Pz_3·70** single crystal, formed by the guest organic molecule and the surrounding Ag_3Pz_3 units that exhibit significant interactions with it. (b) A 1 × 6 × 2 packing mode in the single crystal structure of **Ag_3Pz_3·70** along the *a* axis. Trifluoromethyl groups and H atoms in Ag_3Pz_3 are omitted for clarity. Ag···O interactions are indicated with green dotted lines with distances in Å. C, N, and Ag atoms in Ag_3Pz_3 are depicted in dark gray, light blue, and light gray, respectively; C, O, and H atoms in **70** are depicted in orange, red, and white, respectively.

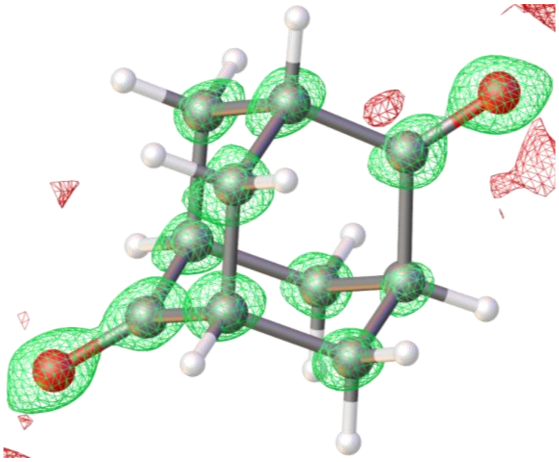

Figure S228. F_{obs} (contour: 1.60) electron density map superimposed on the structure of **70** in the single crystal structure of **Ag_3Pz_3·70**. We believe that the unassigned electron density is attributable to residual solvent molecules and the Ag_3Pz_3 units.

Preparation of Ag_3Pz_3·71. 2.60 mg (0.0107 mmol) of 1-chloroanthraquinone (**71**) was dissolved in 3 mL of n-Hex, followed by the addition of equimolar amounts of Ag_3Pz_3 (10.00 mg, 0.0107 mmol). The resulting mixed solution was filtered and then transferred to a 20 mL screw-capped sample vial. The cap of the sample vial was loosely closed to allow the solvent to slowly evaporate at room temperature. The entire co-crystal incubation process was protected from light using aluminum foil. After the designated evaporation period, typically 1-3 days, high-quality green block-shaped crystals suitable for single-crystal X-ray diffraction analysis formed at the bottom of the vial.

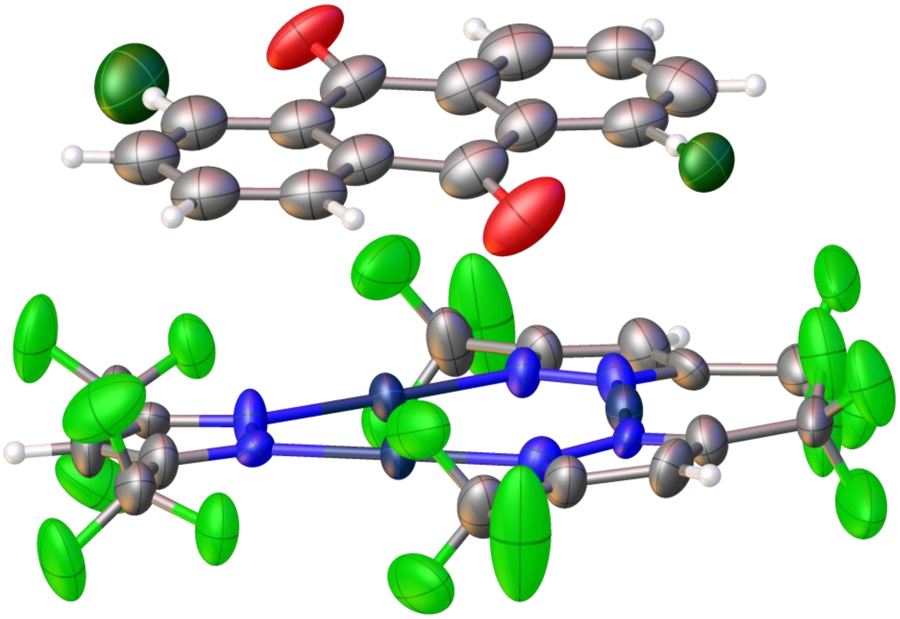

Figure S229. Asymmetric unit of **Ag_3Pz_3·71** (thermal displacement parameters at the 50% probability level).

Table S79. Crystal data and structure refinement for $\mathbf{Ag_3Pz_3{\cdot}71}$

Empirical formula	$C_{29}H_{10}Ag_3ClF_{18}N_6O_2$
Formula weight	1175.49
Temperature/K	100.15
Crystal system	monoclinic
Space group	$P2_1/c$
a/Å	15.5671(2)
b/Å	13.1301(2)
c/Å	16.7111(3)
α/°	90
β/°	91.4580(10)
γ/°	90
Volume/Å^3	3414.60(9)
Z	4
ρ_{calc}g/cm^3	2.287
μ/mm^{-1}	15.705
F(000)	2248.0
Crystal size/mm^3	0.16 × 0.15 × 0.14
Radiation	Cu Kα (λ = 1.54184)
2θ range for data collection/°	5.68 to 156.806
Index ranges	$-17 \leq h \leq 19$, $-16 \leq k \leq 16$, $-19 \leq l \leq 20$
Reflections collected	15799
Independent reflections	6975 [R_{int} = 0.0377, R_{sigma} = 0.0452]
Data/restraints/parameters	6975/228/529
Goodness-of-fit on F^2	1.115
Final R indexes [I>=2σ (I)]	R_1 = 0.0891, wR_2 = 0.2102
Final R indexes [all data]	R_1 = 0.1248, wR_2 = 0.2243
Largest diff. peak/hole / e Å^{-3}	2.15/-1.81
CCDC-number	2501858

Responses to CheckCIF alerts for Ag_3Pz_3·71 crystal structure:

(There is no A-level alert)

B-level alerts:

"ADDSYM Detects Potential Lattice Translation ... ? Check"

The host has higher symmetry, but the co-crystallized molecules prevent the possible additional symmetry. The space group was checked with Platon which indicated that there was pseudo-translation present but that the correct $P2_1/c$.

"ADDSYM Detects New (Pseudo) Symm. Elem I 100 %Fit"

The host has higher symmetry, but the co-crystallized molecules prevent the possible additional symmetry. The space group was checked with Platon which indicated that there was pseudo-translation present but that the correct $P2_1/c$.

"ADDSYM Suggests Possible Pseudo/New Space-group I2/a Check

WARNING: Disordered Atoms Excluded from Analysis

Check Model Parameter Symmetry for Reflection Data Support"

The host has higher symmetry, but the co-crystallized molecules prevent the possible additional symmetry. The space group was checked with Platon which indicated that there was pseudo-translation present but that the correct $P2_1/c$.

"Low Bond Precision on C-C Bonds .... 0.02011 Ang."

Disordered structure.

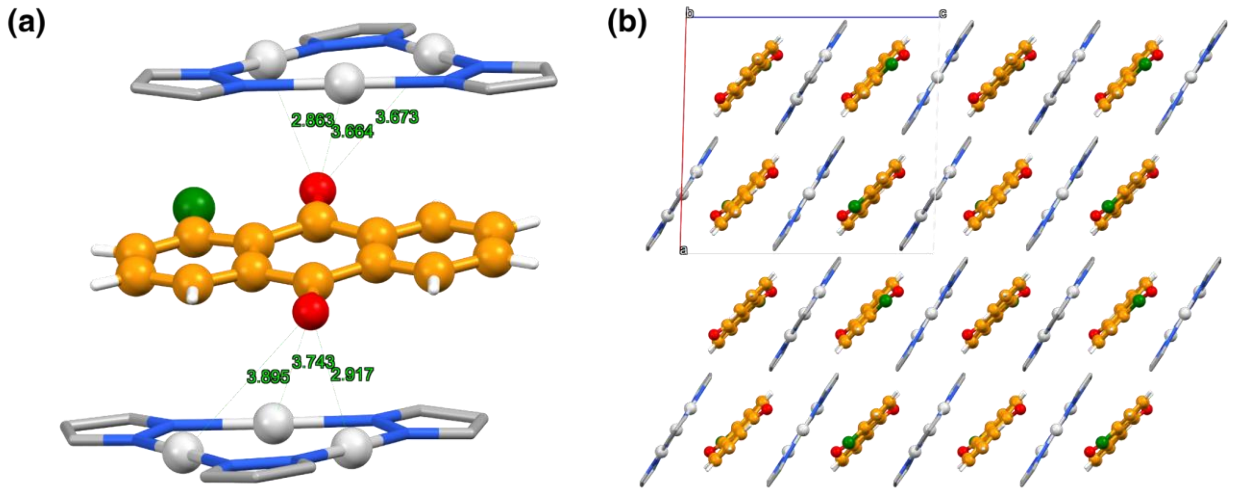

Figure S230. (a) A schematic diagram of the co-crystal structure in the **Ag_3Pz_3·71** single crystal, formed by the guest organic molecule and the surrounding Ag_3Pz_3 units that exhibit significant interactions with it. (b) A 2 × 1 × 2 packing mode in the single crystal structure of **Ag_3Pz_3·71** along the *b* axis. Trifluoromethyl groups and H atoms in Ag_3Pz_3 are omitted for clarity. Ag···O interactions are indicated with green dotted lines with distances in Å. C, N, and Ag atoms in Ag_3Pz_3 are depicted in dark gray, light blue, and light gray, respectively; C, O, Cl, and H atoms in **71** are depicted in orange, red, green, and white, respectively.

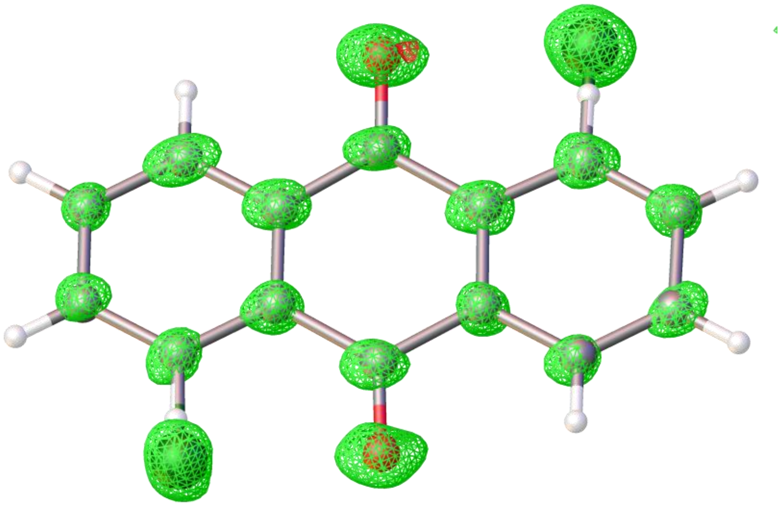

Figure S231. F_{obs} (contour: 0.55) electron density map superimposed on the structure of **71** in the single crystal structure of **Ag_3Pz_3·71**. Please note that there is a twofold disorder in the molecule **71** which is due to molecular vibrations.

Preparation of Ag_3Pz_3·72. 3.07 mg (0.0107 mmol) of 2-bromoanthraquinone (**72**) was dissolved in 3 mL of a binary solvent system of DCM and n-Hex (1:1, v/v), followed by the addition of equimolar amounts of Ag_3Pz_3 (10.00 mg, 0.0107 mmol). The resulting mixed solution was filtered and then transferred to a 20 mL screw-capped sample vial. The cap of the sample vial was loosely closed to allow the solvent to slowly evaporate at room temperature. The entire co-crystal incubation process was protected from light using aluminum foil. After the designated evaporation period, typically 1-3 days, high-quality colorless block-shaped crystals suitable for single-crystal X-ray diffraction analysis formed at the bottom of the vial.

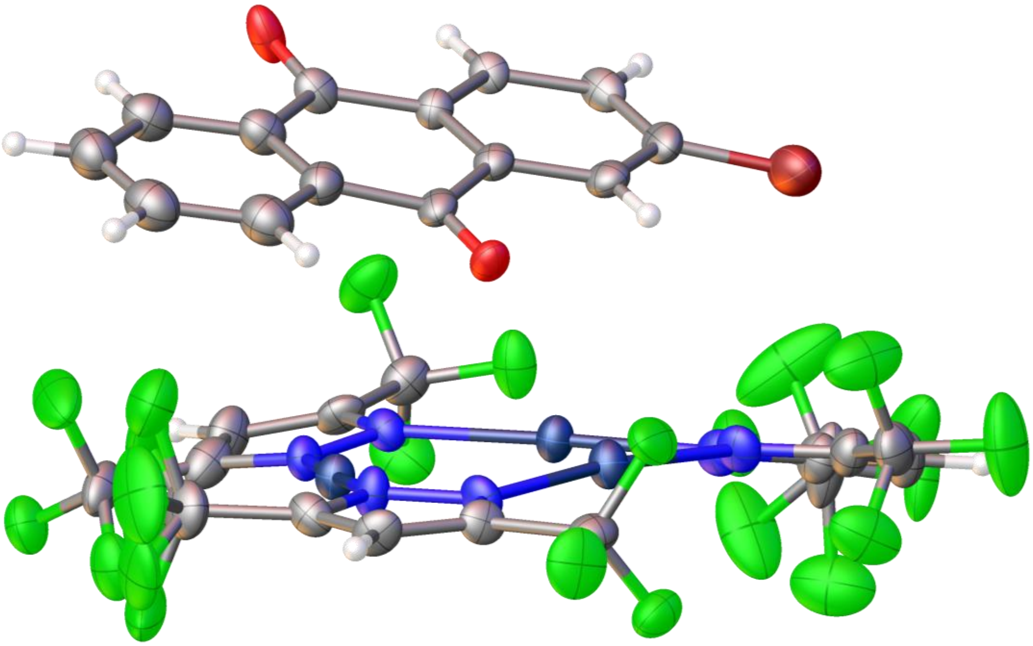

Figure S232. Asymmetric unit of **Ag_3Pz_3·72** (thermal displacement parameters at the 50% probability level).

Table S80. Crystal data and structure refinement for **Ag_3Pz_3·72**

Empirical formula	$C_{22}H_{6.5}Ag_3Br_{0.5}F_{18}N_6O$
Formula weight	1076.4
Temperature/K	100.15
Crystal system	triclinic
Space group	$P\bar{1}$
a/Å	8.79390(10)
b/Å	12.7822(3)
c/Å	14.5413(2)
α/°	103.013(2)
β/°	104.1290(10)
γ/°	106.063(2)
Volume/Å^3	1446.09(5)
Z	2
ρ_{calc}g/cm^3	2.472
μ/mm^{-1}	18.365
F(000)	1018.0
Crystal size/mm^3	0.26 × 0.26 × 0.15
Radiation	Cu Kα (λ = 1.54184)
2θ range for data collection/°	7.58 to 145.744
Index ranges	$-9 \le h \le 10$, $-15 \le k \le 15$, $-18 \le l \le 15$
Reflections collected	18717
Independent reflections	5407 [R_{int} = 0.0653, R_{sigma} = 0.0345]
Data/restraints/parameters	5407/204/564
Goodness-of-fit on F^2	1.051
Final R indexes [I>=2σ (I)]	R_1 = 0.0745, wR_2 = 0.2083
Final R indexes [all data]	R_1 = 0.0758, wR_2 = 0.2104
Largest diff. peak/hole / e Å^{-3}	3.88/-2.93
CCDC-number	2501859

Responses to CheckCIF alerts for Ag_3Pz_3·72 crystal structure:

A-level alerts:

"Check Calcd Resid. Dens. 1.01Ang From Ag01 3.90 eA-3"

This Alert is due to presence of residual density in the presence of heavy metal atom (Ag).

"Check Calcd Resid. Dens. 1.04Ang From Ag02 3.69 eA-3"

This Alert is due to presence of residual density in the presence of heavy metal atom (Ag).

"Check Calcd Resid. Dens. 1.04Ang From Ag03 3.54 eA-3"

This Alert is due to presence of residual density in the presence of heavy metal atom (Ag).

"Check Calcd Resid. Dens. 1.03Ang From Ag02 3.52 eA-3"

This Alert is due to presence of residual density in the presence of heavy metal atom (Ag).

B-level alerts:

"Check Calcd Resid. Dens. 1.06Ang From Ag01 3.49 eA-3"

This Alert is due to presence of residual density in the presence of heavy metal atom (Ag).

"Check Calcd Resid. Dens. 1.02Ang From Ag03 3.30 eA-3"

This Alert is due to presence of residual density in the presence of heavy metal atom (Ag).

"Check Calcd Resid. Dens. 0.88Ang From Ag01 -2.93 eA-3"

This Alert is due to presence of residual density in the presence of heavy metal atom (Ag) and disordered structure.

"Check Calcd Resid. Dens. 0.81Ang From Ag03 -2.76 eA-3"

This Alert is due to presence of residual density in the presence of heavy metal atom (Ag) and disordered structure.

"Check Calcd Resid. Dens. 0.88Ang From Ag02 -2.71 eA-3"

This Alert is due to presence of residual density in the presence of heavy metal atom (Ag) and disordered structure.

“Check Calcd Resid. Dens. 0.92Ang From Ag02 -2.68 eA-3”

This Alert is due to presence of residual density in the presence of heavy metal atom (Ag) and disordered structure.

“Check Calcd Resid. Dens. 1.01Ang From Ag01 -2.68 eA-3”

This Alert is due to presence of residual density in the presence of heavy metal atom (Ag) and disordered structure.

“Check Calcd Resid. Dens. 0.88Ang From Ag03 -2.62 eA-3”

This Alert is due to presence of residual density in the presence of heavy metal atom (Ag) and disordered structure.

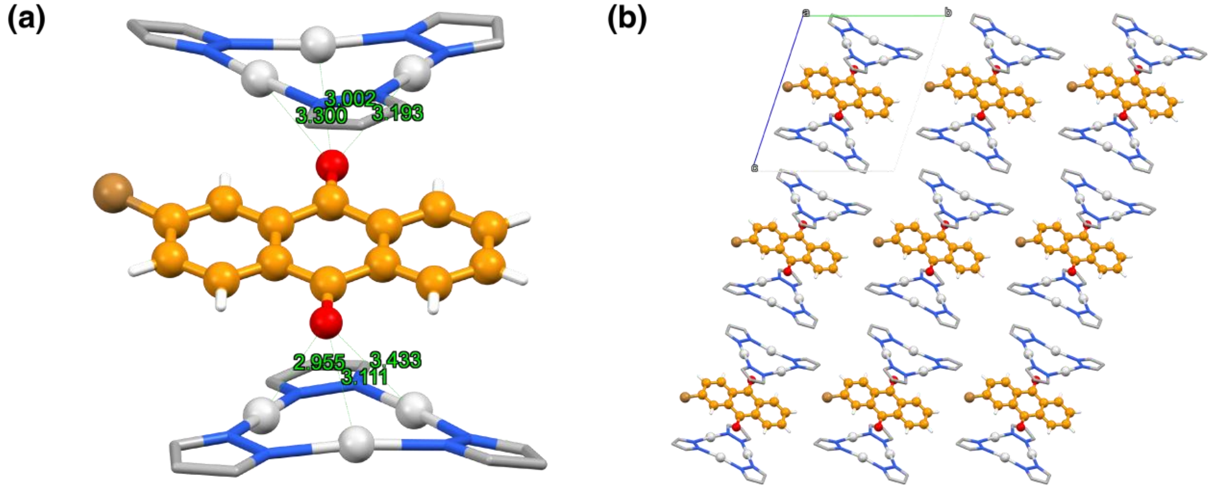

Figure S233. (a) A schematic diagram of the co-crystal structure in the $\mathbf{Ag_3Pz_3{\cdot}72}$ single crystal, formed by the guest organic molecule and the surrounding Ag_3Pz_3 units that exhibit significant interactions with it. (b) A 1 × 3 × 3 packing mode in the single crystal structure of $\mathbf{Ag_3Pz_3{\cdot}72}$ along the *a* axis. Trifluoromethyl groups and H atoms in Ag_3Pz_3 are omitted for clarity. Ag···O interactions are indicated with green dotted lines with distances in Å. C, N, and Ag atoms in Ag_3Pz_3 are depicted in dark gray, light blue, and light gray, respectively; C, O, Br, and H atoms in **72** are depicted in orange, red, brown, and white, respectively.

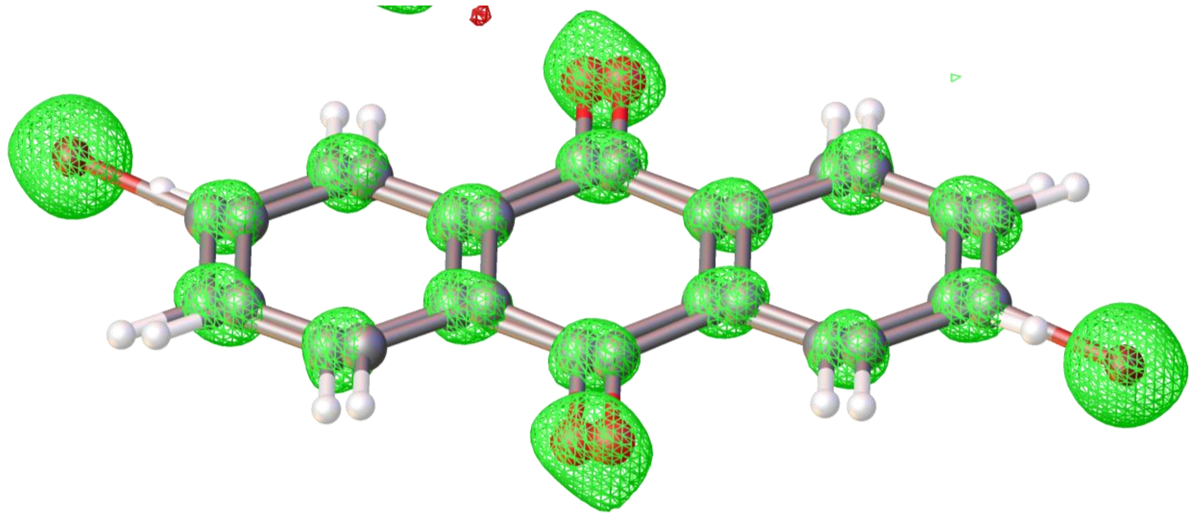

Figure S234. F_{obs} (contour: 1.05) electron density map superimposed on the structure of **72** in the single crystal structure of $\mathbf{Ag_3Pz_3{\cdot}72}$. Please note that there is a twofold disorder in the molecule **72**, likely due to molecular symmetry.

Preparation of Ag_3Pz_3·73. 2.60 mg (0.0107 mmol) of 4-chlorochalcone (**73**) was dissolved in 3 mL of n-Hex, followed by the addition of equimolar amounts of Ag_3Pz_3 (10.00 mg, 0.0107 mmol). The resulting mixed solution was filtered and then transferred to a 20 mL screw-capped sample vial. The cap of the sample vial was loosely closed to allow the solvent to slowly evaporate at room temperature. The entire co-crystal incubation process was protected from light using aluminum foil. After the designated evaporation period, typically 1-3 days, high-quality colorless needle-shaped crystals suitable for single-crystal X-ray diffraction analysis formed at the bottom of the vial.

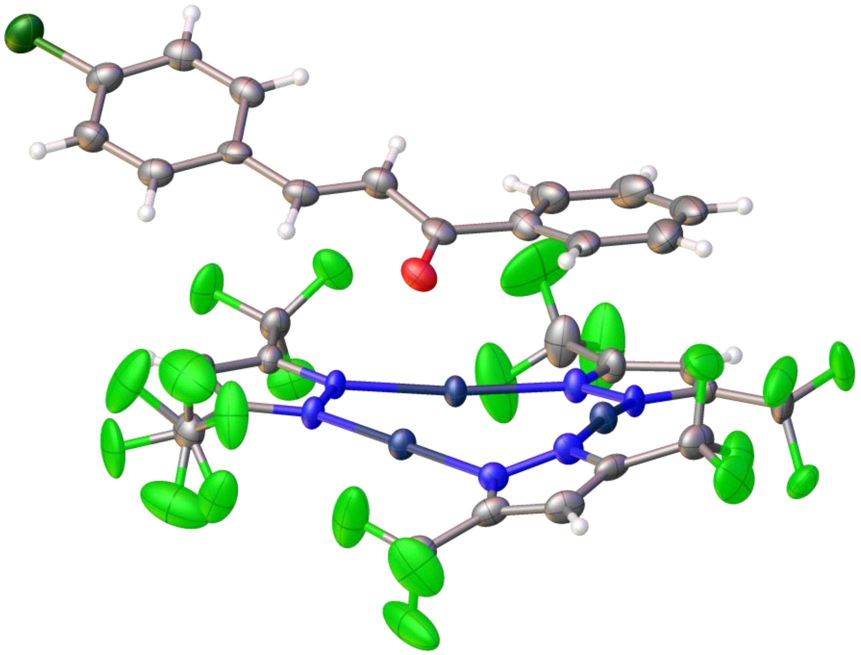

Figure S235. Asymmetric unit of **Ag_3Pz_3·73** (thermal displacement parameters at the 50% probability level).

Table S81. Crystal data and structure refinement for **Ag_3Pz_3·73**

Empirical formula	$C_{30}H_{14}Ag_3ClF_{18}N_6O$
Formula weight	1175.53
Temperature/K	100.00(12)
Crystal system	monoclinic
Space group	$P2_1/n$
a/Å	12.86710(10)
b/Å	12.0958(2)
c/Å	23.4124(3)
α/°	90
β/°	90.7790(10)
γ/°	90
Volume/Å^3	3643.52(8)
Z	4
ρ_{calc}g/cm^3	2.143
μ/mm^{-1}	14.695
F(000)	2256.0
Crystal size/mm^3	0.17 × 0.16 × 0.14
Radiation	Cu Kα (λ = 1.54184)
2θ range for data collection/°	8.228 to 156.504
Index ranges	$-15 \le h \le 16$, $-11 \le k \le 14$, $-28 \le l \le 29$
Reflections collected	23611
Independent reflections	7501 [R_{int} = 0.0403, R_{sigma} = 0.0445]
Data/restraints/parameters	7501/39/560
Goodness-of-fit on F^2	1.087
Final R indexes [I>=2σ (I)]	R_1 = 0.0558, wR_2 = 0.1315
Final R indexes [all data]	R_1 = 0.0678, wR_2 = 0.1359
Largest diff. peak/hole / e Å^{-3}	1.31/-1.20
CCDC-number	2501860

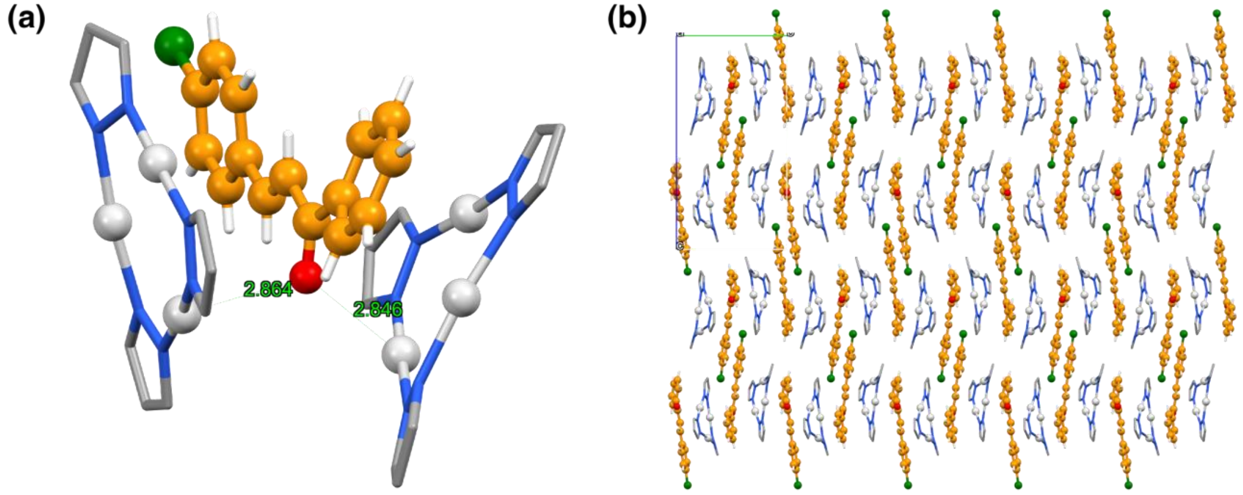

Figure S236. (a) A schematic diagram of the co-crystal structure in the **Ag_3Pz_3·73** single crystal, formed by the guest organic molecule and the surrounding Ag_3Pz_3 units that exhibit significant interactions with it. (b) A 1 × 5 × 1 packing mode in the single crystal structure of **Ag_3Pz_3·73** along the *a* axis. Trifluoromethyl groups and H atoms in Ag_3Pz_3 are omitted for clarity. Ag···O interactions are indicated with green dotted lines with distances in Å. C, N, and Ag atoms in Ag_3Pz_3 are depicted in dark gray, light blue, and light gray, respectively; C, O, Cl, and H atoms in **73** are depicted in orange, red, green, and white, respectively.

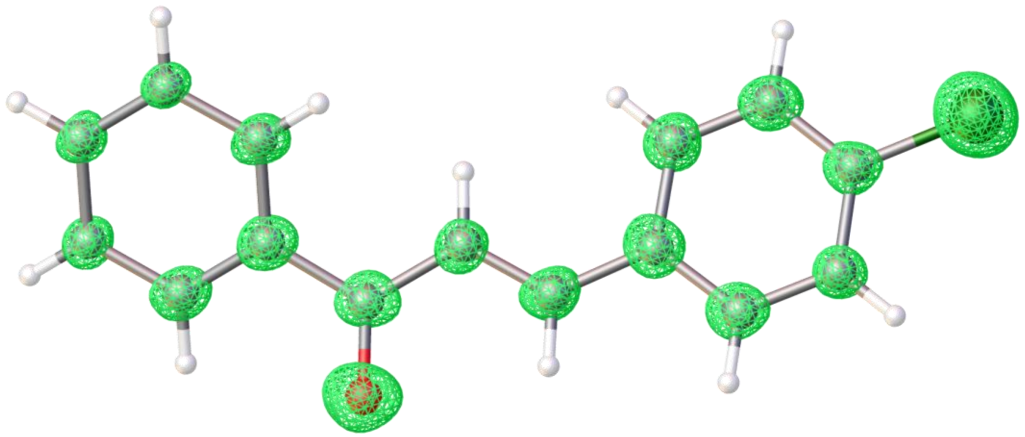

Figure S237. F_{obs} (contour: 0.95) electron density map superimposed on the structure of **73** in the single crystal structure of **Ag_3Pz_3·73**.

Preparation of Ag_3Pz_3·74. 2.28 mg (0.0107 mmol) of 1-(4-bromo-3-methylphenyl)ethanone (**74**) was dissolved in 3 mL of a binary solvent system of DCM and n-Hex (1:1, v/v), followed by the addition of equimolar amounts of Ag_3Pz_3 (10.00 mg, 0.0107 mmol). The resulting mixed solution was filtered and then transferred to a 20 mL screw-capped sample vial. The cap of the sample vial was loosely closed to allow the solvent to slowly evaporate at room temperature. The entire co-crystal incubation process was protected from light using aluminum foil. After the designated evaporation period, typically 1-3 days, high-quality colorless block-shaped crystals suitable for single-crystal X-ray diffraction analysis formed at the bottom of the vial.

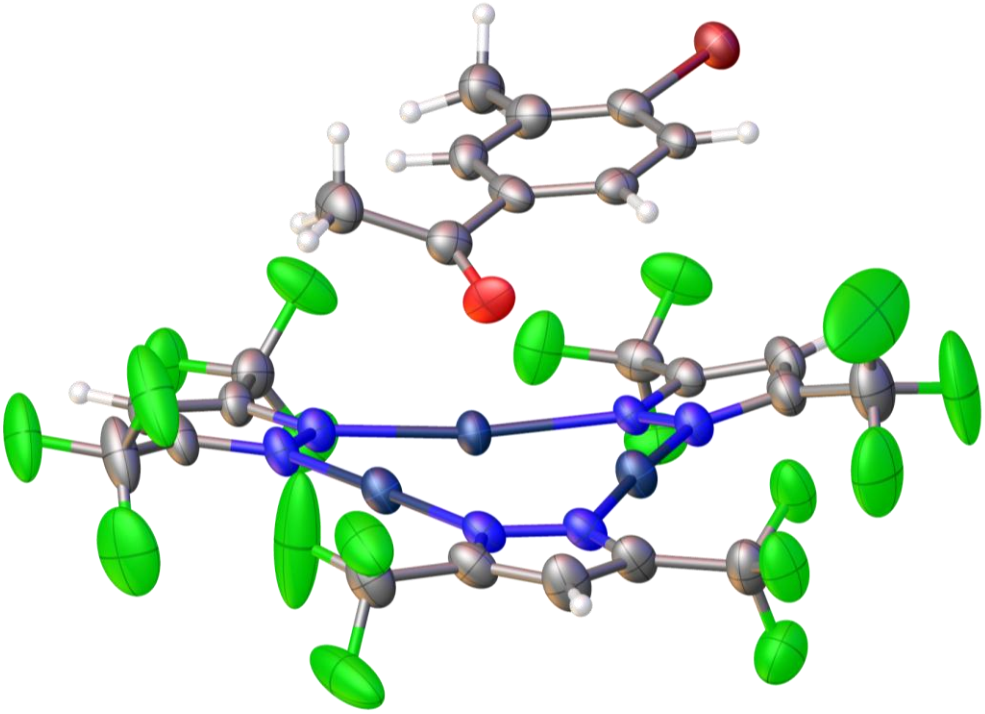

Figure S238. Asymmetric unit of **Ag_3Pz_3·74** (thermal displacement parameters at the 50% probability level).

Table S82. Crystal data and structure refinement for **Ag_3Pz_3·74**

Empirical formula	$C_{24}H_{12}Ag_3BrF_{18}N_6O$
Formula weight	1145.92
Temperature/K	100.00(10)
Crystal system	monoclinic
Space group	$P2_1/c$
a/Å	8.9202(4)
b/Å	18.1007(8)
c/Å	20.1319(9)
α/°	90
β/°	92.225(4)
γ/°	90
Volume/Å^3	3248.1(3)
Z	4
ρ_{calc}g/cm^3	2.343
μ/mm^{-1}	17.102
F(000)	2176.0
Crystal size/mm^3	0.26 × 0.23 × 0.15
Radiation	Cu Kα (λ = 1.54184)
2θ range for data collection/°	8.792 to 145.746
Index ranges	$-9 \le h \le 10$, $-22 \le k \le 21$, $-24 \le l \le 24$
Reflections collected	25235
Independent reflections	6270 [R_{int} = 0.0360, R_{sigma} = 0.0309]
Data/restraints/parameters	6270/13/480
Goodness-of-fit on F^2	1.069
Final R indexes [I>=2σ (I)]	R_1 = 0.0371, wR_2 = 0.0976
Final R indexes [all data]	R_1 = 0.0451, wR_2 = 0.1020
Largest diff. peak/hole / e Å^{-3}	1.65/-1.43
CCDC-number	2501861

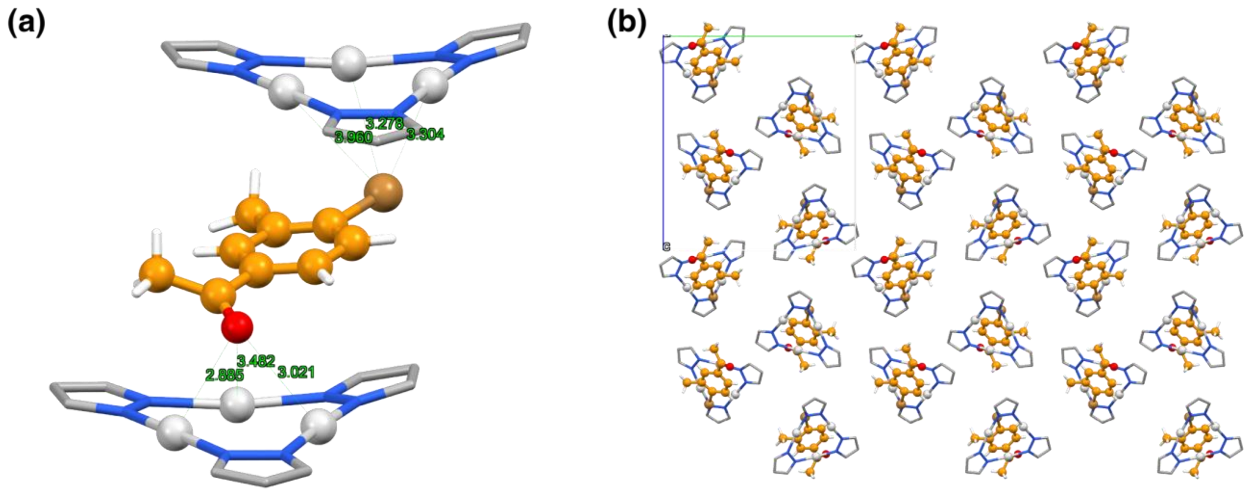

Figure S239. (a) A schematic diagram of the co-crystal structure in the **Ag_3Pz_3·74** single crystal, formed by the guest organic molecule and the surrounding Ag_3Pz_3 units that exhibit significant interactions with it. (b) A 1 × 3 × 2 packing mode in the single crystal structure of **Ag_3Pz_3·74** along the *a* axis. Trifluoromethyl groups and H atoms in Ag_3Pz_3 are omitted for clarity. Ag···O and Ag···Br interactions are indicated with green dotted lines with distances in Å. C, N, and Ag atoms in Ag_3Pz_3 are depicted in dark gray, light blue, and light gray, respectively; C, O, Br, and H atoms in **74** are depicted in orange, red, brown, and white, respectively.

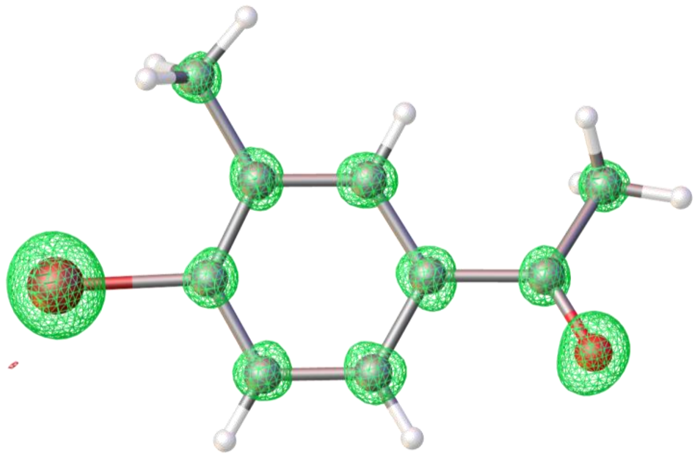

Figure S240. F_{obs} (contour: 0.85) electron density map superimposed on the structure of **74** in the single crystal structure of **Ag_3Pz_3·74**.

Preparation of Ag_3Pz_3·75. 1.45 mg (0.0107 mmol) of 2'-aminoacetophenone (**75**) was dissolved in 3 mL of a binary solvent system of DCM and MeOH (1:1, v/v), followed by the addition of equimolar amounts of Ag_3Pz_3 (10.00 mg, 0.0107 mmol). The resulting mixed solution was filtered and then transferred to a 20 mL screw-capped sample vial. The cap of the sample vial was loosely closed to allow the solvent to slowly evaporate at room temperature. The entire co-crystal incubation process was protected from light using aluminum foil. After the designated evaporation period, typically 1-3 days, high-quality colorless plate-shaped crystals suitable for single-crystal X-ray diffraction analysis formed at the bottom of the vial.

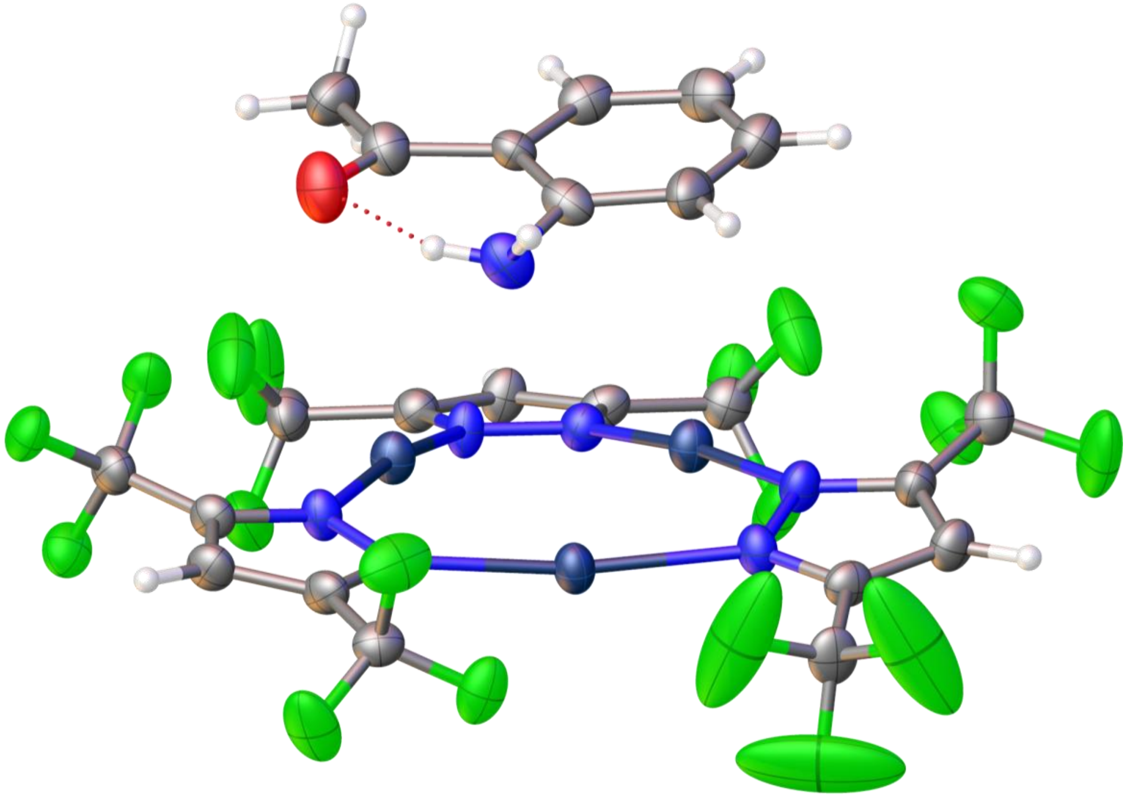

Figure S241. Asymmetric unit of **Ag_3Pz_3·75** (thermal displacement parameters at the 50% probability level).

Table S83. Crystal data and structure refinement for $\mathbf{Ag_3Pz_3{\cdot}75}$

Empirical formula	$C_{23}H_{12}Ag_3F_{18}N_7O$
Formula weight	1068.01
Temperature/K	100.0(3)
Crystal system	triclinic
Space group	$P\bar{1}$
a/Å	7.9092(2)
b/Å	9.6128(2)
c/Å	19.9883(4)
α/°	85.900(2)
β/°	88.992(2)
γ/°	89.601(2)
Volume/Å^3	1515.55(6)
Z	2
ρ_{calc}g/cm^3	2.340
μ/mm^{-1}	16.780
F(000)	1020.0
Crystal size/mm^3	0.2 × 0.2 × 0.1
Radiation	Cu Kα (λ = 1.54184)
2θ range for data collection/°	8.872 to 157.136
Index ranges	$-9 \leq h \leq 10$, $-12 \leq k \leq 11$, $-25 \leq l \leq 22$
Reflections collected	15894
Independent reflections	6191 [R_{int} = 0.0426, R_{sigma} = 0.0480]
Data/restraints/parameters	6191/0/471
Goodness-of-fit on F^2	1.050
Final R indexes [I>=2σ (I)]	R_1 = 0.0713, wR_2 = 0.1978
Final R indexes [all data]	R_1 = 0.0775, wR_2 = 0.2014
Largest diff. peak/hole / e Å^{-3}	2.25/-1.54
CCDC-number	2501862

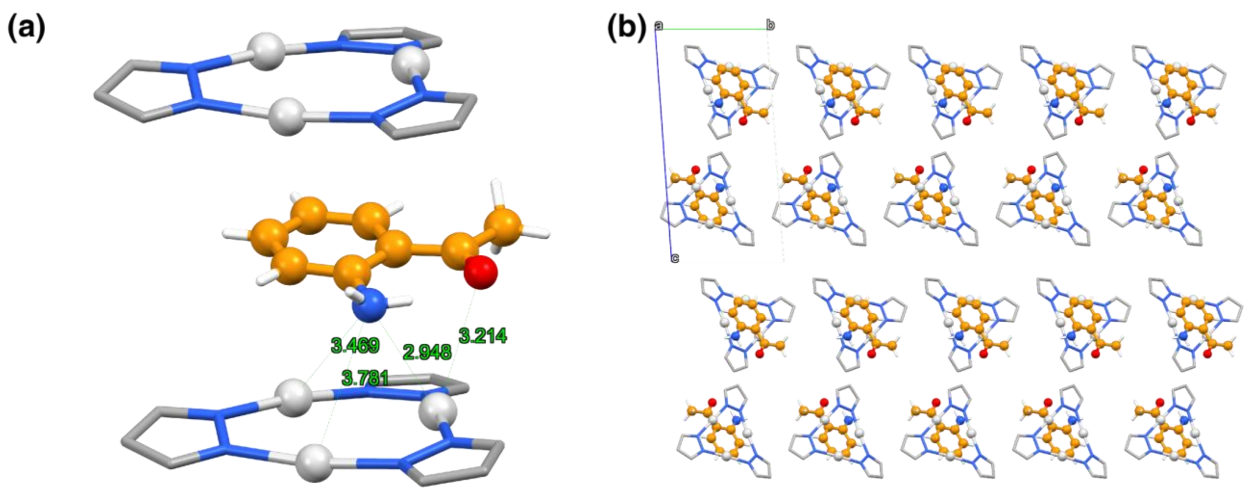

Figure S242. (a) A schematic diagram of the co-crystal structure in the $\mathbf{Ag_3Pz_3{\cdot}75}$ single crystal, formed by the guest organic molecule and the surrounding Ag_3Pz_3 units that exhibit significant interactions with it. (b) A 1 × 5 × 2 packing mode in the single crystal structure of $\mathbf{Ag_3Pz_3{\cdot}75}$ along the *a* axis. Trifluoromethyl groups and H atoms in Ag_3Pz_3 are omitted for clarity. Ag···O and Ag···N interactions are indicated with green dotted lines with distances in Å. C, N, and Ag atoms in Ag_3Pz_3 are depicted in dark gray, light blue, and light gray, respectively; C, O, N, and H atoms in **75** are depicted in orange, red, light blue, and white, respectively.

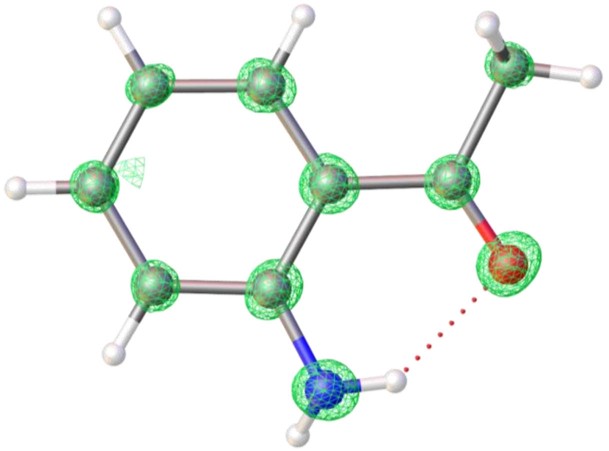

Figure S243. F_{obs} (contour: 1.35) electron density map superimposed on the structure of **75** in the single crystal structure of $\mathbf{Ag_3Pz_3{\cdot}75}$.

Preparation of Ag_3Pz_3·76. 1.45 mg (0.0107 mmol) of 4'-aminoacetophenone (**76**) was dissolved in 3 mL of a binary solvent system of DCM and n-Hex (1:1, v/v), followed by the addition of equimolar amounts of Ag_3Pz_3 (10.00 mg, 0.0107 mmol). The resulting mixed solution was filtered and then transferred to a 20 mL screw-capped sample vial. The cap of the sample vial was loosely closed to allow the solvent to slowly evaporate at room temperature. The entire co-crystal incubation process was protected from light using aluminum foil. After the designated evaporation period, typically 1-3 days, high-quality colorless needle-shaped crystals suitable for single-crystal X-ray diffraction analysis formed at the bottom of the vial.

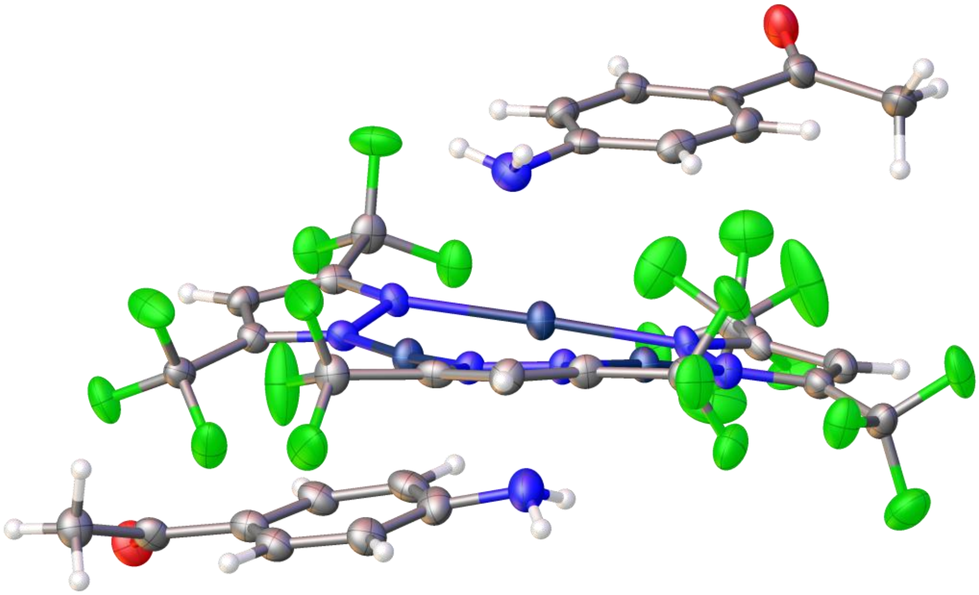

Figure S244. Asymmetric unit of **Ag_3Pz_3·76** (thermal displacement parameters at the 50% probability level).

Table S84. Crystal data and structure refinement for **Ag_3Pz_3·76**

Empirical formula	$C_{31}H_{21}Ag_3F_{18}N_8O_2$
Formula weight	1203.17
Temperature/K	100.01(15)
Crystal system	triclinic
Space group	$P\bar{1}$
a/Å	8.69270(10)
b/Å	12.2745(2)
c/Å	18.6304(2)
α/°	102.5280(10)
β/°	95.4840(10)
γ/°	103.6060(10)
Volume/Å^3	1863.40(4)
Z	2
ρ_{calc}g/cm^3	2.144
μ/mm^{-1}	13.777
F(000)	1164.0
Crystal size/mm^3	0.3 × 0.2 × 0.1
Radiation	Cu Kα (λ = 1.54184)
2θ range for data collection/°	4.918 to 155.838
Index ranges	$-10 \leq h \leq 8$, $-14 \leq k \leq 15$, $-21 \leq l \leq 23$
Reflections collected	17145
Independent reflections	7548 [R_{int} = 0.0539, R_{sigma} = 0.0625]
Data/restraints/parameters	7548/21/591
Goodness-of-fit on F^2	1.079
Final R indexes [I>=2σ (I)]	R_1 = 0.0506, wR_2 = 0.1292
Final R indexes [all data]	R_1 = 0.0636, wR_2 = 0.1337
Largest diff. peak/hole / e Å^{-3}	1.03/-1.33
CCDC-number	2501863

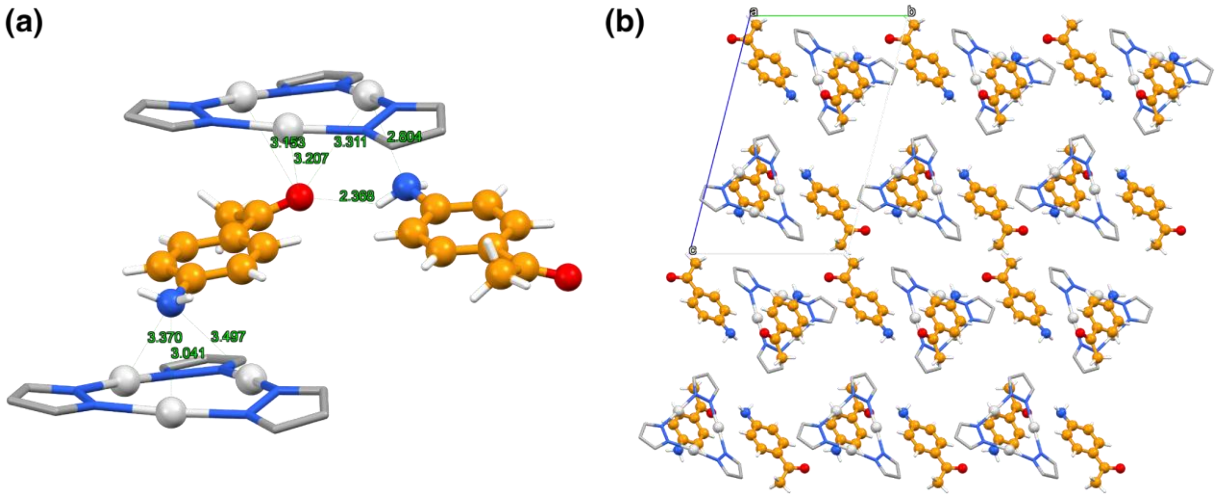

Figure S245. (a) A schematic diagram of the co-crystal structure in the **Ag_3Pz_3·76** single crystal, formed by the guest organic molecule and the surrounding Ag_3Pz_3 units that exhibit significant interactions with it. (b) A 1 × 5 × 2 packing mode in the single crystal structure of **Ag_3Pz_3·76** along the *a* axis. Trifluoromethyl groups and H atoms in Ag_3Pz_3 are omitted for clarity. Ag···O, Ag···N, and N-H···O interactions are indicated with green dotted lines with distances in Å. C, N, and Ag atoms in Ag_3Pz_3 are depicted in dark gray, light blue, and light gray, respectively; C, O, N, and H atoms in **76** are depicted in orange, red, light blue, and white, respectively.

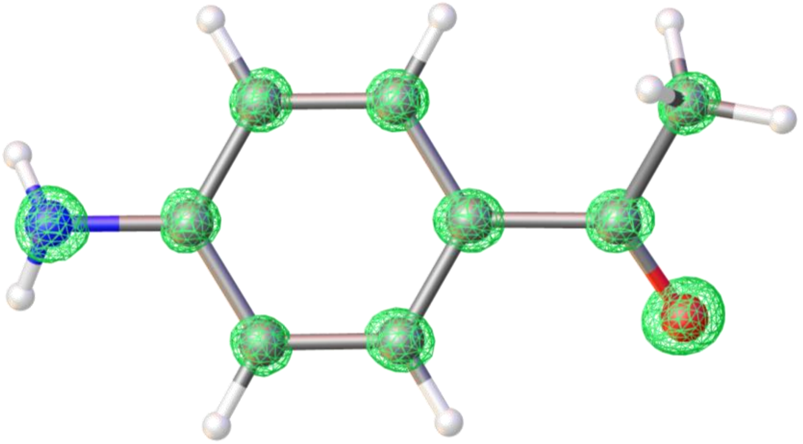

Figure S246. F_{obs} (contour: 1.55) electron density map superimposed on the structure of **76** in the single crystal structure of **Ag_3Pz_3·76**.

Preparation of Ag_3Pz_3·77. 1.25 mg (0.0107 mmol) of indole (**77**) was dissolved in 3 mL of a binary solvent system of DCM and n-Hex (1:1, v/v), followed by the addition of equimolar amounts of Ag_3Pz_3 (10.00 mg, 0.0107 mmol). The resulting mixed solution was filtered and then transferred to a 20 mL screw-capped sample vial. The cap of the sample vial was loosely closed to allow the solvent to slowly evaporate at room temperature. The entire co-crystal incubation process was protected from light using aluminum foil. After the designated evaporation period, typically 1-3 days, high-quality colorless needle-shaped crystals suitable for single-crystal X-ray diffraction analysis formed at the bottom of the vial.

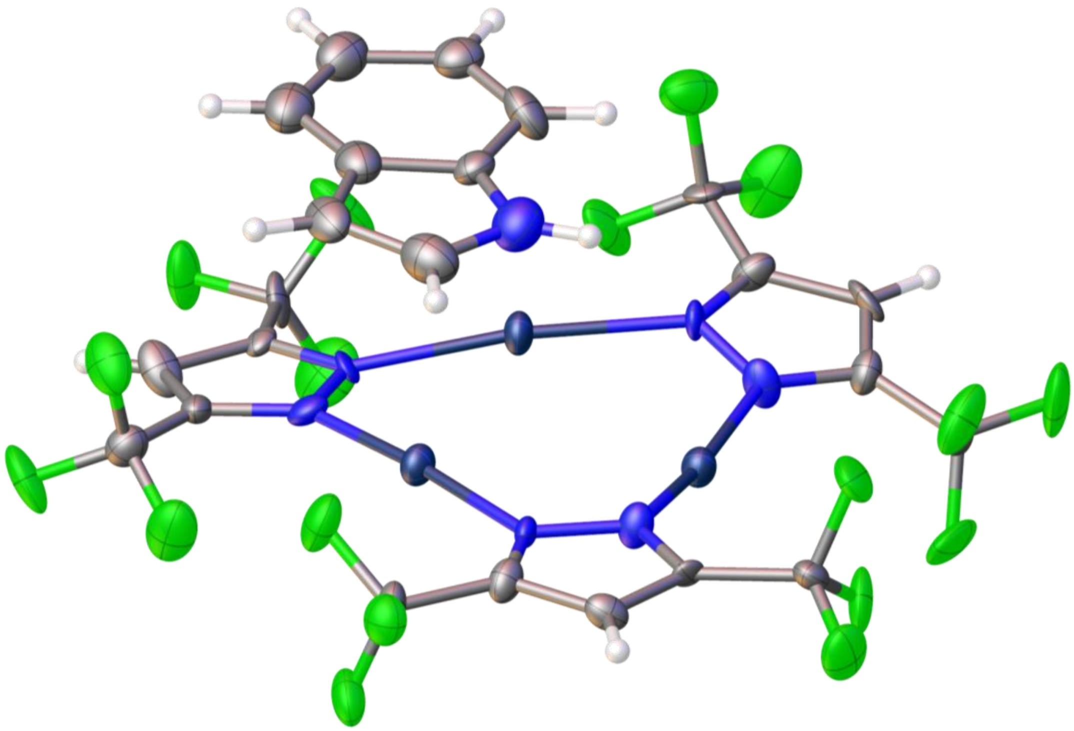

Figure S247. Asymmetric unit of **Ag_3Pz_3·77** (thermal displacement parameters at the 50% probability level).

Table S85. Crystal data and structure refinement for $\mathbf{Ag_3Pz_3{\cdot}77}$

Empirical formula	$C_{23}H_{10}Ag_3F_{18}N_7$
Formula weight	1049.99
Temperature/K	100.01(11)
Crystal system	monoclinic
Space group	$P2_1/c$
a/Å	7.8391(4)
b/Å	9.6805(4)
c/Å	39.0136(19)
α/°	90
β/°	91.604(4)
γ/°	90
Volume/Å^3	2959.4(2)
Z	4
ρ_{calc}g/cm^3	2.357
μ/mm^{-1}	17.145
F(000)	2000.0
Crystal size/mm^3	0.2 × 0.1 × 0.1
Radiation	Cu Kα (λ = 1.54184)
2θ range for data collection/°	9.414 to 157.09
Index ranges	$-9 \le h \le 7$, $-7 \le k \le 11$, $-49 \le l \le 49$
Reflections collected	11640
Independent reflections	5920 [R_{int} = 0.0373, R_{sigma} = 0.0404]
Data/restraints/parameters	5920/104/460
Goodness-of-fit on F^2	1.172
Final R indexes [I>=2σ (I)]	R_1 = 0.1150, wR_2 = 0.2938
Final R indexes [all data]	R_1 = 0.1187, wR_2 = 0.2956
Largest diff. peak/hole / e Å^{-3}	4.41/-2.85
CCDC-number	2501864

Responses to CheckCIF alerts for Ag_3Pz_3·77 crystal structure:

A-level alerts:

“Check Calcd Resid. Dens. 0.93Ang From Ag3 5.02 eA-3”

This Alert is due to presence of residual density in the presence of heavy metal atom (Ag).

“Check Calcd Resid. Dens. 0.80Ang From Ag1 4.53 eA-3”

This Alert is due to presence of residual density in the presence of heavy metal atom (Ag).

“Check Calcd Resid. Dens. 0.72Ang From Ag3 4.37 eA-3”

This Alert is due to presence of residual density in the presence of heavy metal atom (Ag).

“Check Calcd Resid. Dens. 1.06Ang From Ag2 4.19 eA-3”

This Alert is due to presence of residual density in the presence of heavy metal atom (Ag).

“Check Calcd Resid. Dens. 1.01Ang From Ag1 3.94 eA-3”

This Alert is due to presence of residual density in the presence of heavy metal atom (Ag).

“Check Calcd Resid. Dens. 0.76Ang From Ag2 3.84 eA-3”

This Alert is due to presence of residual density in the presence of heavy metal atom (Ag).

B-level alerts:

“Low Bond Precision on C-C Bonds .... 0.026 Ang.”

Disordered structure.

“Check Calcd Resid. Dens. 0.91Ang From Ag3 2.54 eA-3”

This Alert is due to presence of residual density in the presence of heavy metal atom (Ag).

“Check Calcd Resid. Dens. 0.74Ang From Ag1 -2.88 eA-3”

This Alert is due to presence of residual density in the presence of heavy metal atom (Ag).

“Check Calcd Resid. Dens. 0.76Ang From Ag3 -2.86 eA-3”

This Alert is due to presence of residual density in the presence of heavy metal atom (Ag).

“Check Calcd Resid. Dens. 0.85Ang From Ag2 -2.83 eA-3”

This Alert is due to presence of residual density in the presence of heavy metal atom (Ag).

“Check Calcd Resid. Dens. 1.39Ang From F009   -2.66 eA-3”

This Alert is due to presence of residual density in the presence of heavy metal atom (Ag).

“Check Calcd Resid. Dens. 0.79Ang From Ag3   -2.60 eA-3”

This Alert is due to presence of residual density in the presence of heavy metal atom (Ag).

“Check Calcd Resid. Dens. 0.90Ang From Ag1   -2.51 eA-3”

This Alert is due to presence of residual density in the presence of heavy metal atom (Ag).

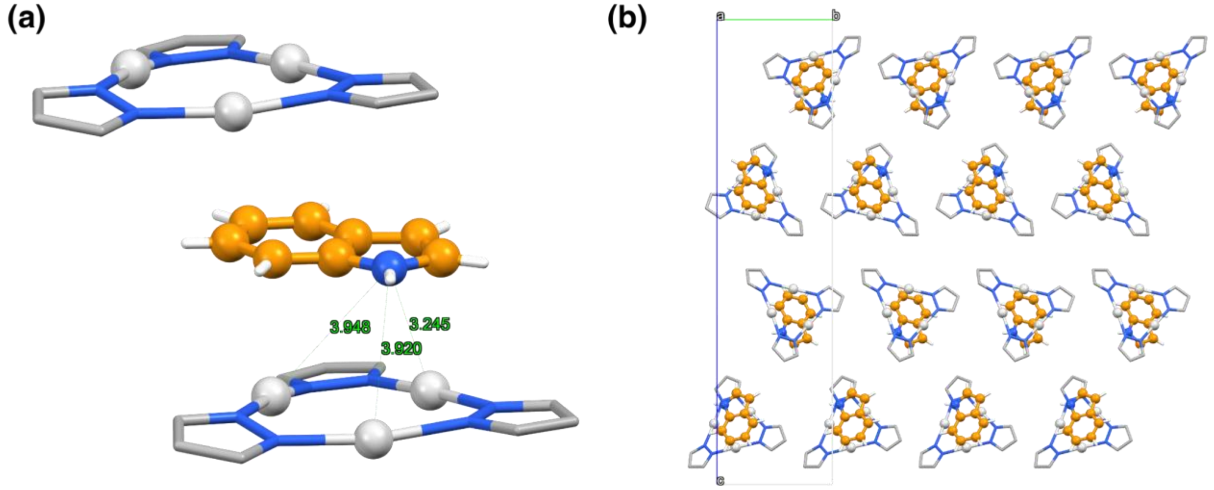

Figure S248. (a) A schematic diagram of the co-crystal structure in the **Ag$_3$Pz$_3$·77** single crystal, formed by the guest organic molecule and the surrounding Ag$_3$Pz$_3$ units that exhibit significant interactions with it. (b) A 1 × 4 × 1 packing mode in the single crystal structure of **Ag$_3$Pz$_3$·77** along the *a* axis. Trifluoromethyl groups and H atoms in Ag$_3$Pz$_3$ are omitted for clarity. Ag···N interactions are indicated with green dotted lines with distances in Å. C, N, and Ag atoms in Ag$_3$Pz$_3$ are depicted in dark gray, light blue, and light gray, respectively; C, N, and H atoms in **77** are depicted in orange, light blue, and white, respectively.

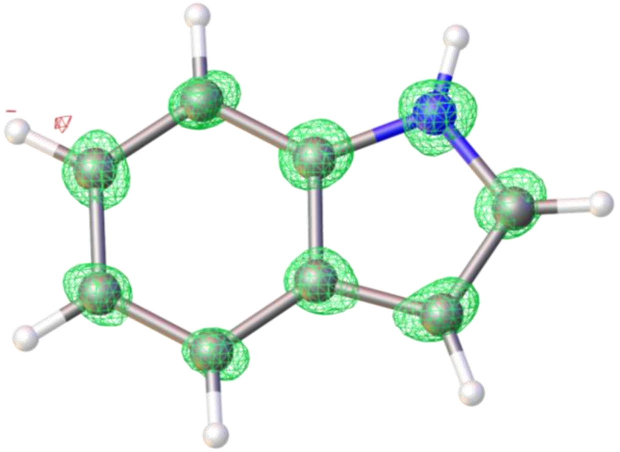

Figure S249. F_{obs} (contour: 0.85) electron density map superimposed on the structure of **77** in the single crystal structure of **Ag$_3$Pz$_3$·77**.

Preparation of Ag_3Pz_3·78. 1.70 mg (0.0107 mmol) of 3-acetylindole (**78**) was dissolved in 3 mL of a binary solvent system of DCM and n-Hex (1:1, v/v), followed by the addition of equimolar amounts of Ag_3Pz_3 (10.00 mg, 0.0107 mmol). The resulting mixed solution was filtered and then transferred to a 20 mL screw-capped sample vial. The cap of the sample vial was loosely closed to allow the solvent to slowly evaporate at room temperature. The entire co-crystal incubation process was protected from light using aluminum foil. After the designated evaporation period, typically 1-3 days, high-quality colorless block-shaped crystals suitable for single-crystal X-ray diffraction analysis formed at the bottom of the vial.

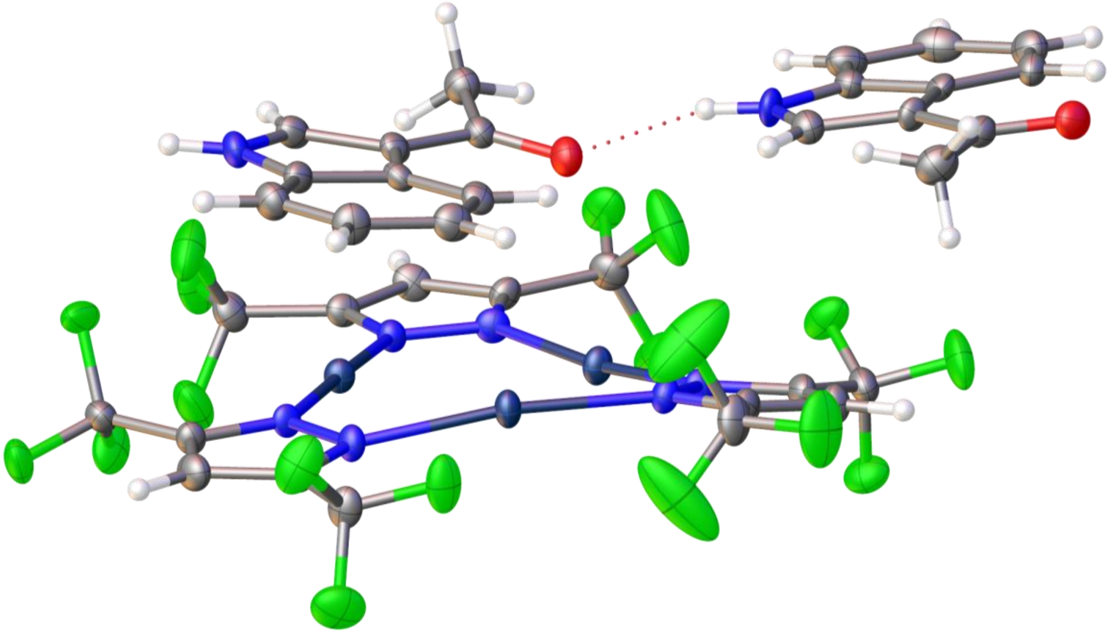

Figure S250. Asymmetric unit of **Ag_3Pz_3·78** (thermal displacement parameters at the 50% probability level).

Table S86. Crystal data and structure refinement for $\mathbf{Ag_3Pz_3 \cdot 78}$

Empirical formula	$C_{35}H_{21}Ag_3F_{18}N_8O_2$
Formula weight	1251.21
Temperature/K	100.0(2)
Crystal system	triclinic
Space group	$P\bar{1}$
a/Å	8.7193(2)
b/Å	10.7952(2)
c/Å	21.1971(4)
α/°	88.455(2)
β/°	86.642(2)
γ/°	88.502(2)
Volume/Å^3	1990.46(7)
Z	2
ρ_{calc}g/cm^3	2.088
μ/mm^{-1}	12.933
F(000)	1212.0
Crystal size/mm^3	0.16 × 0.15 × 0.14
Radiation	Cu Kα (λ = 1.54184)
2θ range for data collection/°	8.196 to 156.518
Index ranges	$-11 \leq h \leq 11$, $-13 \leq k \leq 13$, $-26 \leq l \leq 26$
Reflections collected	22525
Independent reflections	8191 [R_{int} = 0.0418, R_{sigma} = 0.0487]
Data/restraints/parameters	8191/0/597
Goodness-of-fit on F^2	1.074
Final R indexes [I>=2σ (I)]	R_1 = 0.0448, wR_2 = 0.1108
Final R indexes [all data]	R_1 = 0.0577, wR_2 = 0.1147
Largest diff. peak/hole / e Å^{-3}	0.93/-0.88
CCDC-number	2501865

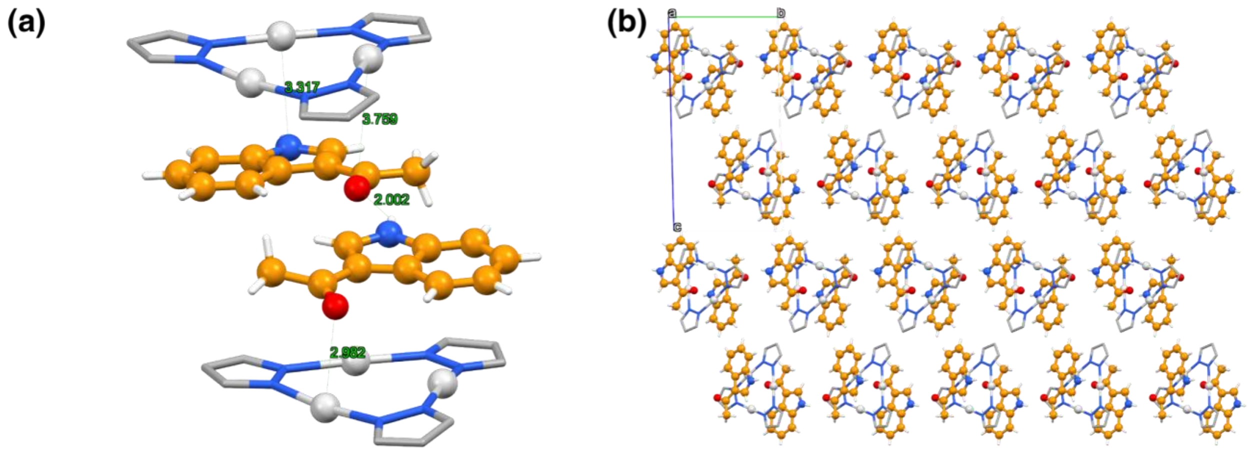

Figure S251. (a) A schematic diagram of the co-crystal structure in the **Ag_3Pz_3·78** single crystal, formed by the guest organic molecule and the surrounding Ag_3Pz_3 units that exhibit significant interactions with it. (b) A 1 × 5 × 2 packing mode in the single crystal structure of **Ag_3Pz_3·78** along the *a* axis. Trifluoromethyl groups and H atoms in Ag_3Pz_3 are omitted for clarity. Ag···N, Ag···O, and N-H···O interactions are indicated with green dotted lines with distances in Å. C, N, and Ag atoms in Ag_3Pz_3 are depicted in dark gray, light blue, and light gray, respectively; C, O, N, and H atoms in **78** are depicted in orange, red, light blue, and white, respectively.

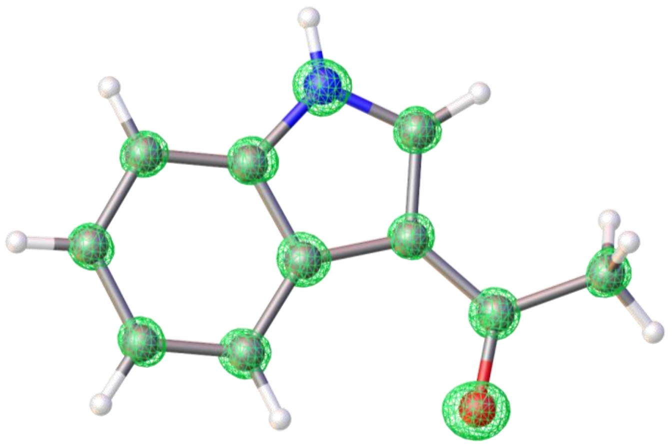

Figure S252. F_{obs} (contour: 1.90) electron density map superimposed on the structure of **78** in the single crystal structure of **Ag_3Pz_3·78**.

Preparation of Ag_3Pz_3·79. 2.07 mg (0.0107 mmol) of diacetamate (**79**) was dissolved in 3 mL of a binary solvent system of DCM and c-Hex (1:1, v/v), followed by the addition of equimolar amounts of Ag_3Pz_3 (10.00 mg, 0.0107 mmol). The resulting mixed solution was filtered and then transferred to a 20 mL screw-capped sample vial. The cap of the sample vial was loosely closed to allow the solvent to slowly evaporate at room temperature. The entire co-crystal incubation process was protected from light using aluminum foil. After the designated evaporation period, typically 1-3 days, high-quality colorless needle-shaped crystals suitable for single-crystal X-ray diffraction analysis formed at the bottom of the vial.

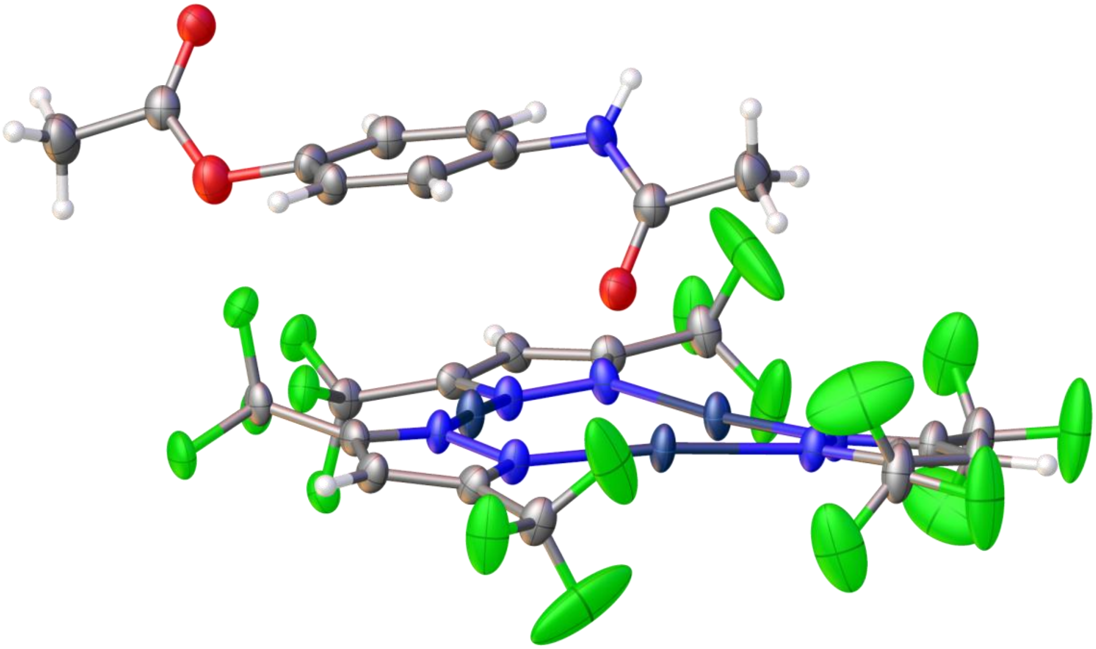

Figure S253. Asymmetric unit of **Ag_3Pz_3·79** (thermal displacement parameters at the 50% probability level).

Table S87. Crystal data and structure refinement for **Ag_3Pz_3·79**

Empirical formula	$C_{25}H_{14}Ag_3F_{18}N_7O_3$
Formula weight	1126.04
Temperature/K	100.00(10)
Crystal system	monoclinic
Space group	$P2_1/c$
a/Å	9.3891(2)
b/Å	25.8241(6)
c/Å	13.8681(3)
α/°	90
β/°	90.059(2)
γ/°	90
Volume/Å^3	3362.53(13)
Z	4
ρ_{calc}g/cm^3	2.224
μ/mm^{-1}	15.220
F(000)	2160.0
Crystal size/mm^3	0.23 × 0.21 × 0.12
Radiation	Cu Kα (λ = 1.54184)
2θ range for data collection/°	6.846 to 149.944
Index ranges	$-11 \leq h \leq 11$, $-30 \leq k \leq 32$, $-17 \leq l \leq 15$
Reflections collected	39272
Independent reflections	6728 [R_{int} = 0.0606, R_{sigma} = 0.0290]
Data/restraints/parameters	6728/0/507
Goodness-of-fit on F^2	1.081
Final R indexes [I>=2σ (I)]	R_1 = 0.0568, wR_2 = 0.1320
Final R indexes [all data]	R_1 = 0.0746, wR_2 = 0.1394
Largest diff. peak/hole / e Å^{-3}	1.50/-0.97
CCDC-number	2501866

Responses to CheckCIF alert for Ag_3Pz_3·79 crystal structure:

(There is no A-level alert)

B-level alert:

"ADDSYM Detects New (Pseudo) Symm. Elem C 96 %Fit"

The host has higher symmetry, but the co-crystallized molecules prevent the possible additional symmetry. The space group was checked with Platon, which indicated that there was pseudo-translation present, but that the correct $P2_1/c$.

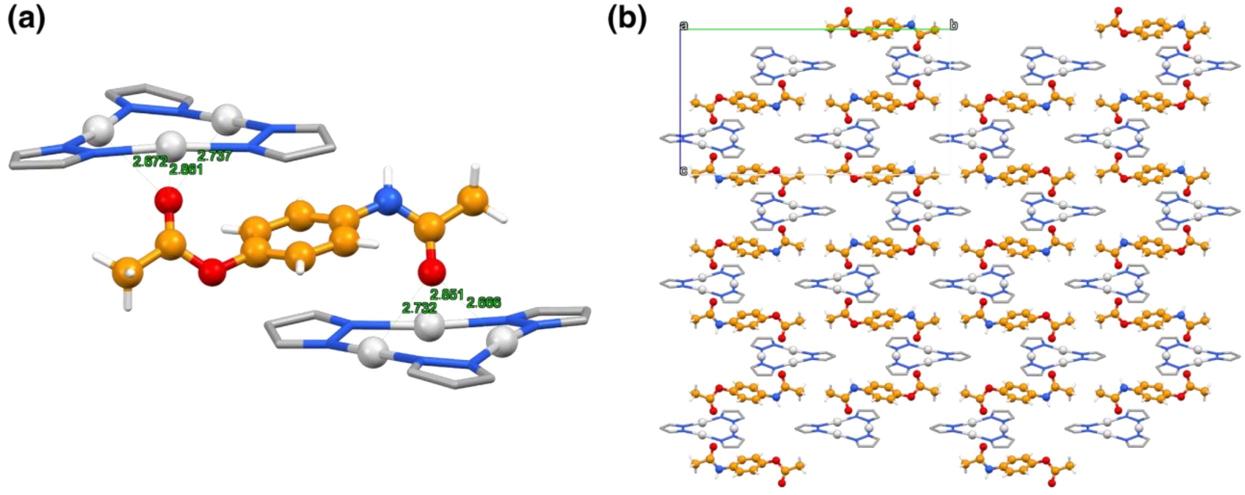

Figure S254. (a) A schematic diagram of the co-crystal structure in the **Ag_3Pz_3·79** single crystal, formed by the guest organic molecule and the surrounding Ag_3Pz_3 units that exhibit significant interactions with it. (b) A 1 × 2 × 3 packing mode in the single crystal structure of **Ag_3Pz_3·79** along the *a* axis. Trifluoromethyl groups and H atoms in Ag_3Pz_3 are omitted for clarity. Ag···O interactions are indicated with green dotted lines with distances in Å. C, N, and Ag atoms in Ag_3Pz_3 are depicted in dark gray, light blue, and light gray, respectively; C, O, N, and H atoms in **79** are depicted in orange, red, light blue, and white, respectively.

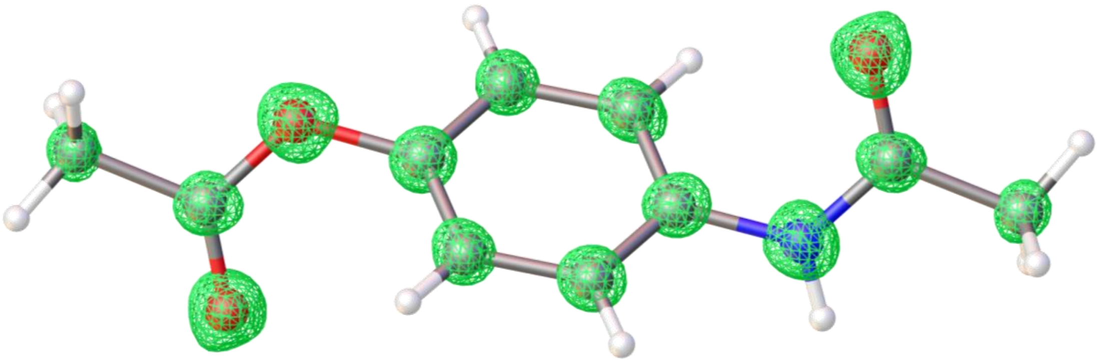

Figure S255. F_{obs} (contour: 0.85) electron density map superimposed on the structure of **79** in the single crystal structure of **Ag_3Pz_3·79**.

Preparation of Ag_3Pz_3·80. 1.85 mg (0.0107 mmol) of N-phenylmaleimide (**80**) was dissolved in 3 mL of n-Hex, followed by the addition of equimolar amounts of Ag_3Pz_3 (10.00 mg, 0.0107 mmol). The resulting mixed solution was filtered and then transferred to a 20 mL screw-capped sample vial. The cap of the sample vial was loosely closed to allow the solvent to slowly evaporate at room temperature. The entire co-crystal incubation process was protected from light using aluminum foil. After the designated evaporation period, typically 1-3 days, high-quality yellow block-shaped crystals suitable for single-crystal X-ray diffraction analysis formed at the bottom of the vial.

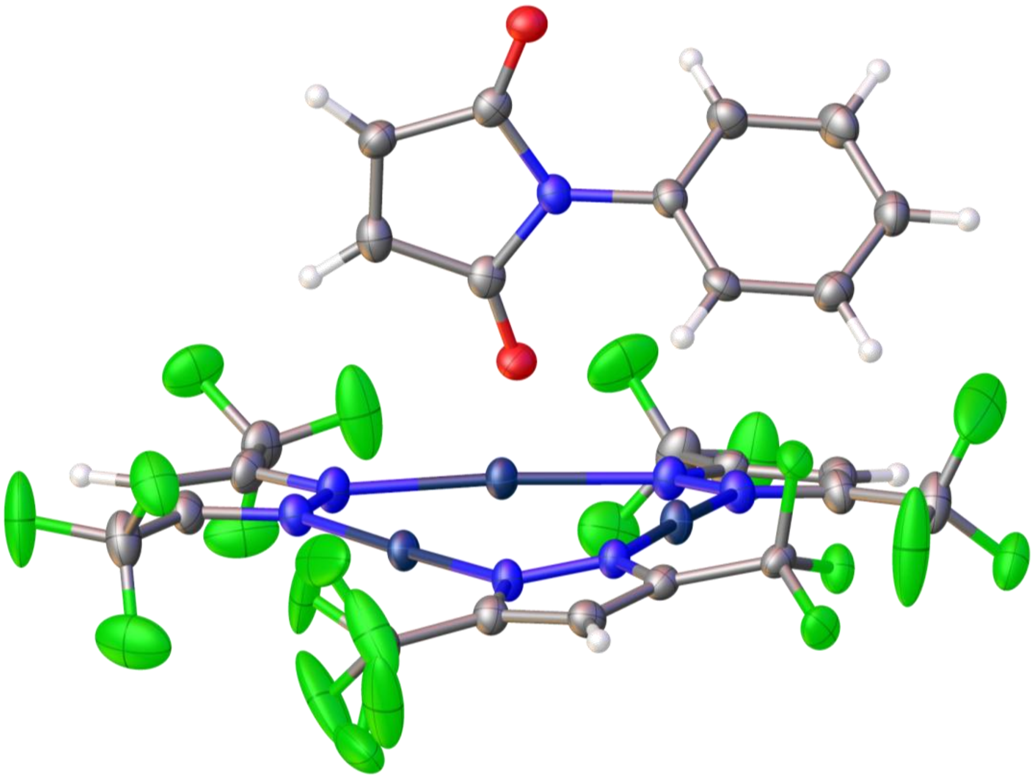

Figure S256. Asymmetric unit of **Ag_3Pz_3·80** (thermal displacement parameters at the 50% probability level).

Table S88. Crystal data and structure refinement for **Ag_3Pz_3·80**

Empirical formula	$C_{25}H_{10}Ag_3F_{18}N_7O_2$
Formula weight	1106.01
Temperature/K	100.01(18)
Crystal system	triclinic
Space group	$P\bar{1}$
a/Å	8.6364(2)
b/Å	13.0948(2)
c/Å	14.9126(4)
α/°	103.145(2)
β/°	96.009(2)
γ/°	98.251(2)
Volume/Å^3	1608.72(6)
Z	2
ρ_{calc}g/cm^3	2.283
μ/mm^{-1}	15.868
F(000)	1056.0
Crystal size/mm^3	0.2 × 0.1 × 0.1
Radiation	Cu Kα (λ = 1.54184)
2θ range for data collection/°	6.148 to 155.76
Index ranges	$-9 \le h \le 10$, $-16 \le k \le 16$, $-16 \le l \le 18$
Reflections collected	13963
Independent reflections	6513 [R_{int} = 0.0346, R_{sigma} = 0.0367]
Data/restraints/parameters	6513/3/524
Goodness-of-fit on F^2	1.054
Final R indexes [I>=2σ (I)]	R_1 = 0.0389, wR_2 = 0.1091
Final R indexes [all data]	R_1 = 0.0414, wR_2 = 0.1104
Largest diff. peak/hole / e Å^{-3}	1.16/-1.11
CCDC-number	2501867

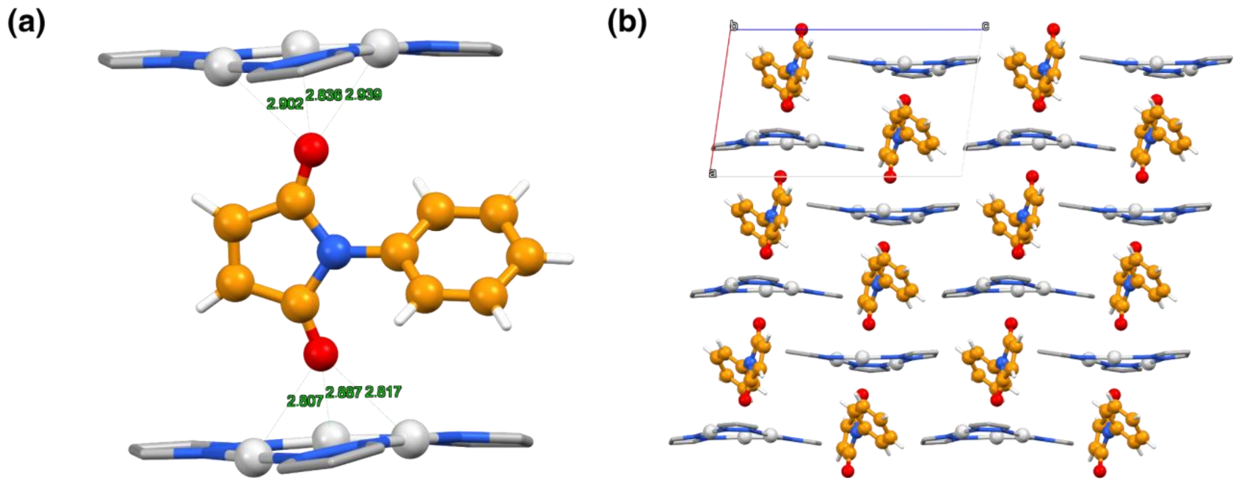

Figure S257. (a) A schematic diagram of the co-crystal structure in the **Ag$_3$Pz$_3$·80** single crystal, formed by the guest organic molecule and the surrounding Ag$_3$Pz$_3$ units that exhibit significant interactions with it. (b) A 3 × 1 × 2 packing mode in the single crystal structure of **Ag$_3$Pz$_3$·80** along the *b* axis. Trifluoromethyl groups and H atoms in Ag$_3$Pz$_3$ are omitted for clarity. Ag···O interactions are indicated with green dotted lines with distances in Å. C, N, and Ag atoms in Ag$_3$Pz$_3$ are depicted in dark gray, light blue, and light gray, respectively; C, O, N, and H atoms in **80** are depicted in orange, red, light blue, and white, respectively.

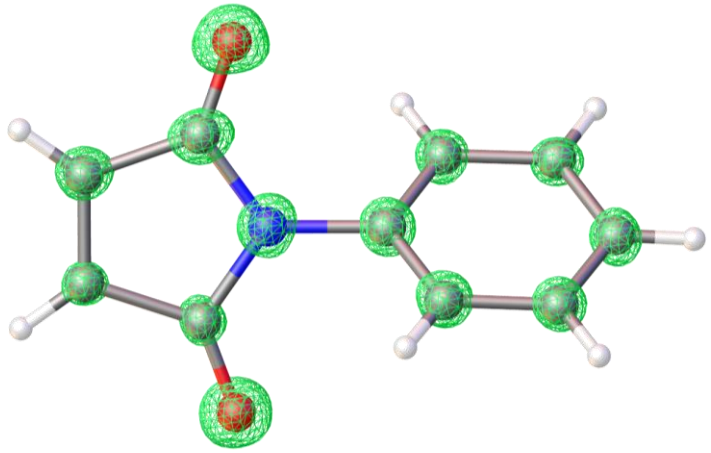

Figure S258. F_{obs} (contour: 2.05) electron density map superimposed on the structure of **80** in the single crystal structure of **Ag$_3$Pz$_3$·80**.

Preparation of Ag_3Pz_3·81. 1.53 mg (0.0107 mmol) of 1-phenylpyrrole (**81**) was dissolved in 3 mL of n-Hex, followed by the addition of equimolar amounts of Ag_3Pz_3 (10.00 mg, 0.0107 mmol). The resulting mixed solution was filtered and then transferred to a 20 mL screw-capped sample vial. The cap of the sample vial was loosely closed to allow the solvent to slowly evaporate at room temperature. The entire co-crystal incubation process was protected from light using aluminum foil. After the designated evaporation period, typically 1-3 days, high-quality colorless plate-shaped crystals suitable for single-crystal X-ray diffraction analysis formed at the bottom of the vial.

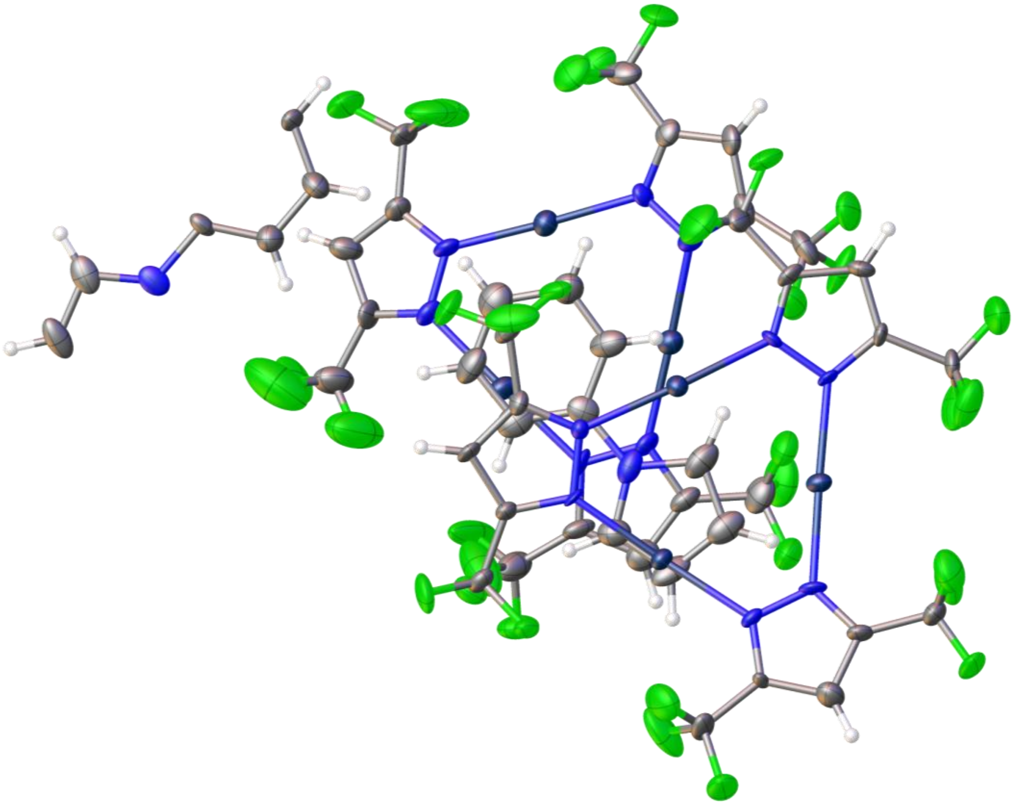

Figure S259. Asymmetric unit of **Ag_3Pz_3·81** (thermal displacement parameters at the 50% probability level).

Table S89. Crystal data and structure refinement for **Ag_3Pz_3·81**

Empirical formula	$C_{45}H_{19.5}Ag_6F_{36}N_{13.5}$
Formula weight	2080.46
Temperature/K	100.00(18)
Crystal system	orthorhombic
Space group	$C222_1$
a/Å	13.2473(2)
b/Å	22.9690(4)
c/Å	42.2825(9)
α/°	90
β/°	90
γ/°	90
Volume/Å^3	12865.6(4)
Z	8
ρ_{calc}g/cm^3	2.148
μ/mm^{-1}	15.764
F(000)	7920.0
Crystal size/mm^3	0.25 × 0.24 × 0.14
Radiation	Cu Kα (λ = 1.54184)
2θ range for data collection/°	7.698 to 157.112
Index ranges	$-16 \leq h \leq 16$, $-29 \leq k \leq 28$, $-52 \leq l \leq 52$
Reflections collected	36253
Independent reflections	13160 [R_{int} = 0.0550, R_{sigma} = 0.0559]
Data/restraints/parameters	13160/55/910
Goodness-of-fit on F^2	1.064
Final R indexes [I>=2σ (I)]	R_1 = 0.0644, wR_2 = 0.1592
Final R indexes [all data]	R_1 = 0.0691, wR_2 = 0.1615
Largest diff. peak/hole / e Å^{-3}	1.71/-0.98
Flack parameter	0.011(7)
CCDC-number	2501868

Responses to CheckCIF alert for Ag_3Pz_3·81 crystal structure:

(There is no A-level alert)

B-level alert:

"Low Bond Precision on C-C Bonds .... 0.0232 Ang."

Disordered structure.

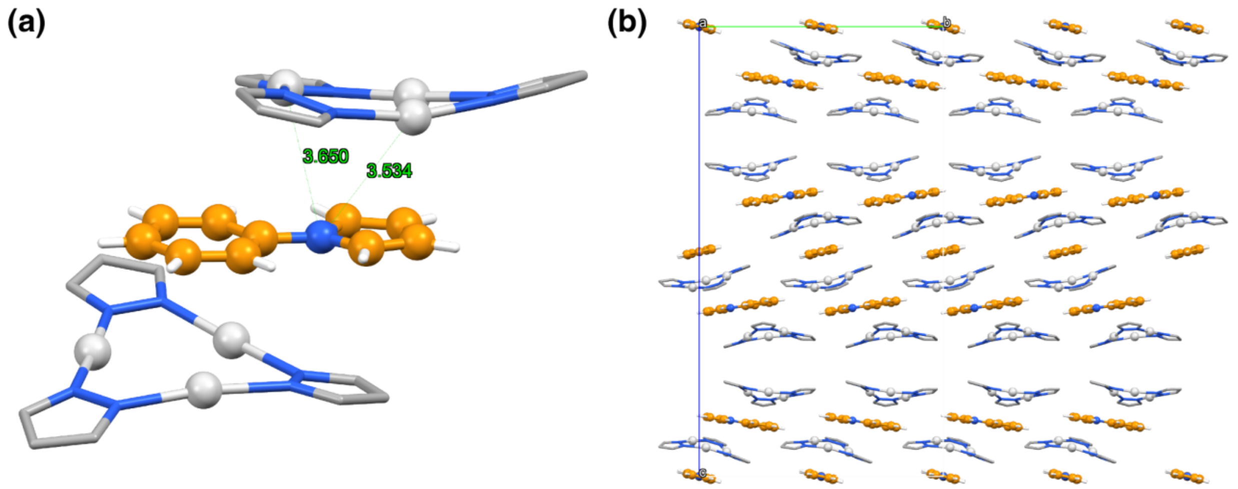

Figure S260. (a) A schematic diagram of the co-crystal structure in the **Ag_3Pz_3·81** single crystal, formed by the guest organic molecule and the surrounding Ag_3Pz_3 units that exhibit significant interactions with it. (b) A 1 × 2 × 1 packing mode in the single crystal structure of **Ag_3Pz_3·81** along the *a* axis. Trifluoromethyl groups and H atoms in Ag_3Pz_3 are omitted for clarity. Ag···N interactions are indicated with green dotted lines with distances in Å. C, N, and Ag atoms in Ag_3Pz_3 are depicted in dark gray, light blue, and light gray, respectively; C, N, and H atoms in **81** are depicted in orange, light blue, and white, respectively.

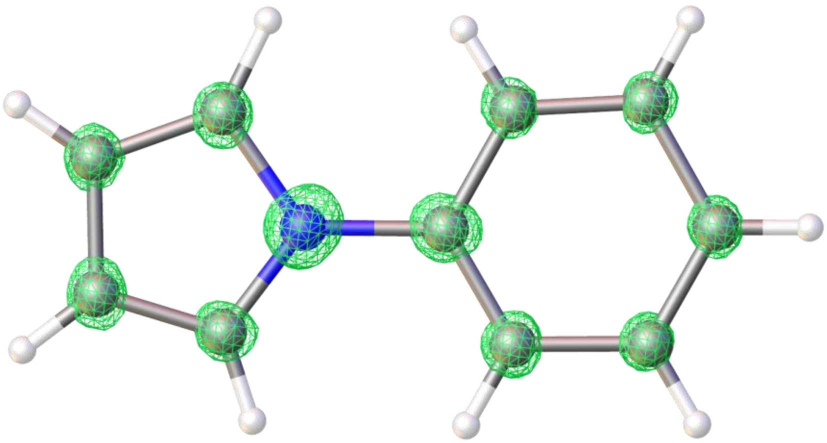

Figure S261. F_{obs} (contour: 0.20) electron density map superimposed on the structure of **81** in the single crystal structure of **Ag_3Pz_3·81**.

Preparation of Ag_3Pz_3·82. 1.79 mg (0.0107 mmol) of carbazole (**82**) was dissolved in 3 mL of n-Hex, followed by the addition of equimolar amounts of Ag_3Pz_3 (10.00 mg, 0.0107 mmol). The resulting mixed solution was filtered and then transferred to a 20 mL screw-capped sample vial. The cap of the sample vial was loosely closed to allow the solvent to slowly evaporate at room temperature. The entire co-crystal incubation process was protected from light using aluminum foil. After the designated evaporation period, typically 1-3 days, high-quality colorless needle-shaped crystals suitable for single-crystal X-ray diffraction analysis formed at the bottom of the vial.

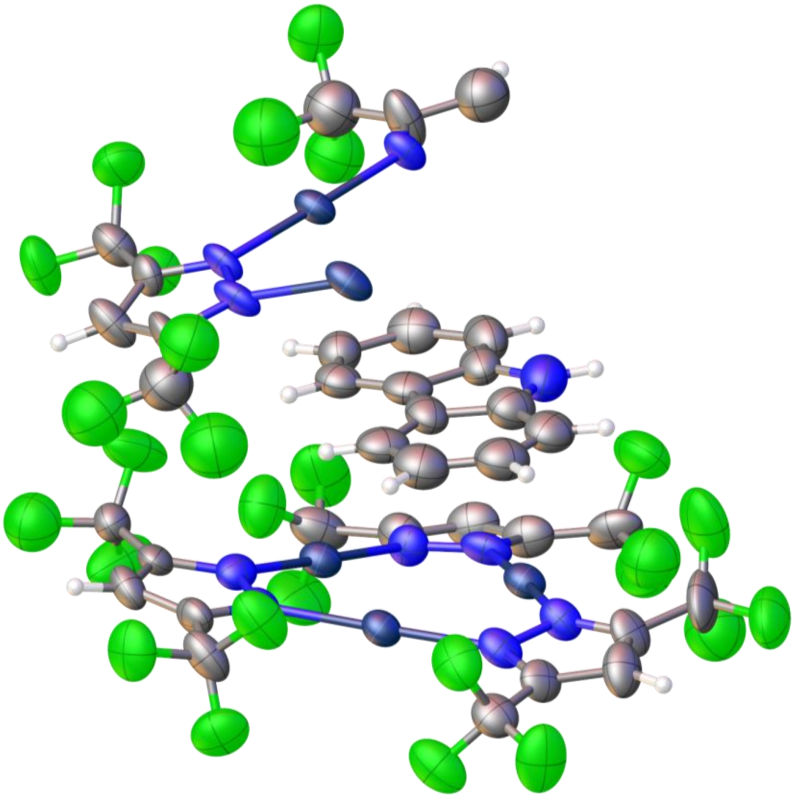

Figure S262. Asymmetric unit of **Ag_3Pz_3·82** (thermal displacement parameters at the 50% probability level).

Table S90. Crystal data and structure refinement for **Ag_3Pz_3·82**

Empirical formula	$C_{23}H_9Ag_3F_{18}N_{6.67}$
Formula weight	1044.32
Temperature/K	100.15
Crystal system	monoclinic
Space group	*C*2/*c*
a/Å	27.4648(11)
b/Å	12.8992(5)
c/Å	28.1210(15)
α/°	90
β/°	97.239(4)
γ/°	90
Volume/Å^3	9883.1(8)
Z	12
ρ_{calc}g/cm^3	2.106
μ/mm^{-1}	15.395
F(000)	5960.0
Crystal size/mm^3	0.2 × 0.2 × 0.1
Radiation	Cu Kα (λ = 1.54184)
2θ range for data collection/°	8.06 to 156.268
Index ranges	$-33 \leq h \leq 33$, $-12 \leq k \leq 16$, $-20 \leq l \leq 33$
Reflections collected	22138
Independent reflections	9929 [R_{int} = 0.0557, R_{sigma} = 0.0538]
Data/restraints/parameters	9929/148/677
Goodness-of-fit on F^2	1.026
Final *R* indexes [I>=2σ (I)]	R_1 = 0.1062, wR_2 = 0.2673
Final *R* indexes [all data]	R_1 = 0.1149, wR_2 = 0.2734
Largest diff. peak/hole / e Å^{-3}	2.39/-2.48
CCDC-number	2501869

Responses to CheckCIF alerts for Ag_3Pz_3·82 crystal structure:

(There is no A-level alert)

B-level alerts:

"Low Bond Precision on C-C Bonds .... 0.02453 Ang."

Disordered structure.

"Check Calcd Resid. Dens. 0.81Ang From Ag5 -2.94 eA-3"

This Alert is due to presence of residual density in the presence of heavy metal atom (Ag).

"Check Calcd Resid. Dens. 0.70Ang From Ag4 -2.56 eA-3"

This Alert is due to presence of residual density in the presence of heavy metal atom (Ag).

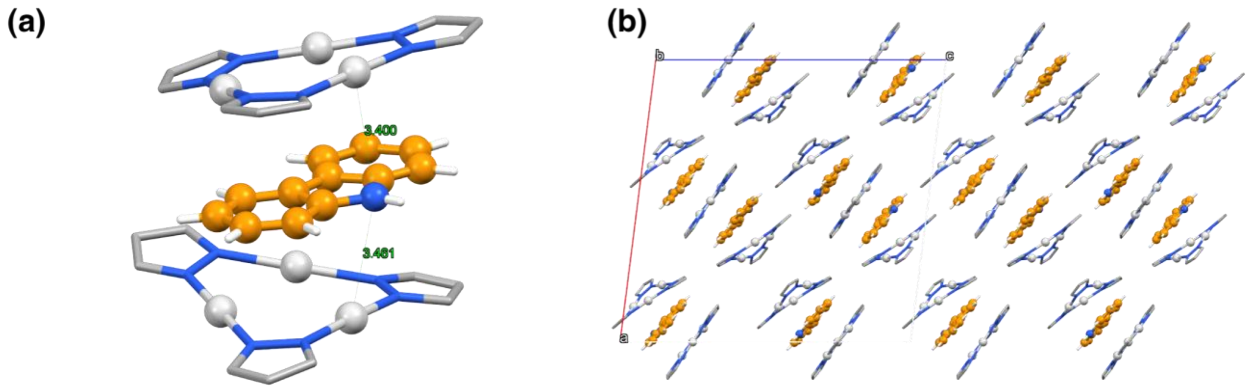

Figure S263. (a) A schematic diagram of the co-crystal structure in the $\mathbf{Ag_3Pz_3 \cdot 82}$ single crystal, formed by the guest organic molecule and the surrounding Ag_3Pz_3 units that exhibit significant interactions with it. (b) A 1 × 1 × 2 packing mode in the single crystal structure of $\mathbf{Ag_3Pz_3 \cdot 82}$ along the *b* axis. Trifluoromethyl groups and H atoms in Ag_3Pz_3 are omitted for clarity. Ag···N interactions are indicated with green dotted lines with distances in Å. C, N, and Ag atoms in Ag_3Pz_3 are depicted in dark gray, light blue, and light gray, respectively; C, N, and H atoms in **82** are depicted in orange, light blue, and white, respectively.

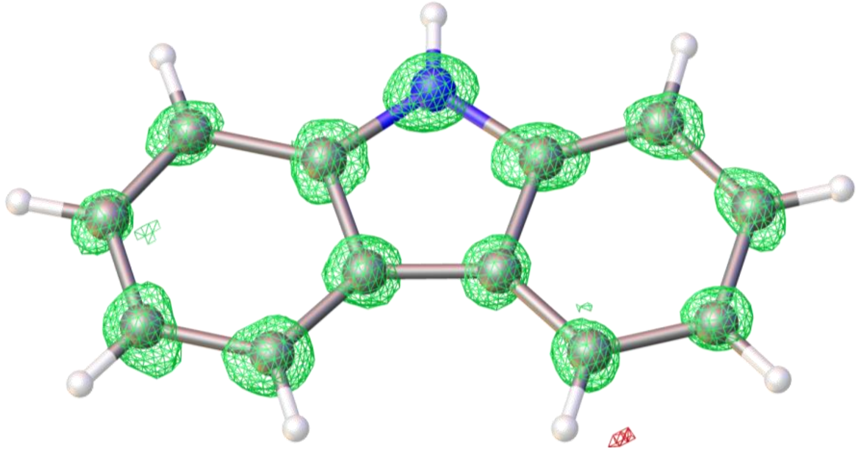

Figure S264. F_{obs} (contour: 0.14) electron density map superimposed on the structure of **82** in the single crystal structure of $\mathbf{Ag_3Pz_3 \cdot 82}$.

Preparation of Ag_3Pz_3·83. 2.55 mg (0.0107 mmol) of 1,4-diaminoanthraquinone (**83**) was dissolved in 3 mL of a binary solvent system of DCM and c-Hex (1:1, v/v), followed by the addition of equimolar amounts of Ag_3Pz_3 (10.00 mg, 0.0107 mmol). The resulting mixed solution was filtered and then transferred to a 20 mL screw-capped sample vial. The cap of the sample vial was loosely closed to allow the solvent to slowly evaporate at room temperature. The entire co-crystal incubation process was protected from light using aluminum foil. After the designated evaporation period, typically 1-3 days, high-quality violet block-shaped crystals suitable for single-crystal X-ray diffraction analysis formed at the bottom of the vial.

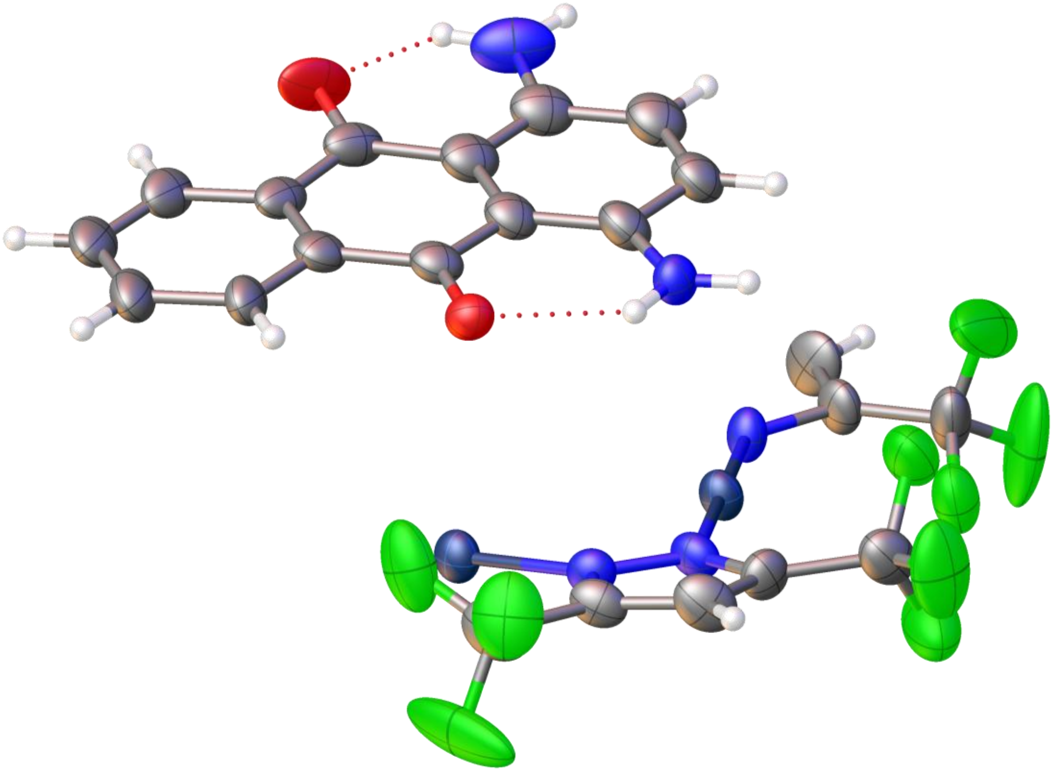

Figure S265. Asymmetric unit of **Ag_3Pz_3·83** (thermal displacement parameters at the 50% probability level).

Table S91. Crystal data and structure refinement for **Ag_3Pz_3·83**

Empirical formula	$C_{29}H_{13}Ag_3F_{18}N_8O_2$
Formula weight	1171.08
Temperature/K	100.15
Crystal system	monoclinic
Space group	*I*2/*a*
a/Å	16.3878(15)
b/Å	13.2075(13)
c/Å	16.0469(17)
α/°	90
β/°	91.059(9)
γ/°	90
Volume/Å^3	3472.6(6)
Z	4
ρ_{calc}g/cm^3	2.240
μ/mm^{-1}	14.764
F(000)	2248.0
Crystal size/mm^3	0.26 × 0.19 × 0.16
Radiation	Cu Kα (λ = 1.54184)
2θ range for data collection/°	8.6 to 146.868
Index ranges	$-18 \le h \le 20$, $-15 \le k \le 16$, $-18 \le l \le 19$
Reflections collected	12222
Independent reflections	3324 [R_{int} = 0.0561, R_{sigma} = 0.0444]
Data/restraints/parameters	3324/126/329
Goodness-of-fit on F^2	1.069
Final *R* indexes [I>=2σ (I)]	R_1 = 0.0533, wR_2 = 0.1353
Final *R* indexes [all data]	R_1 = 0.0642, wR_2 = 0.1403
Largest diff. peak/hole / e Å^{-3}	1.02/-1.64
CCDC-number	2501870

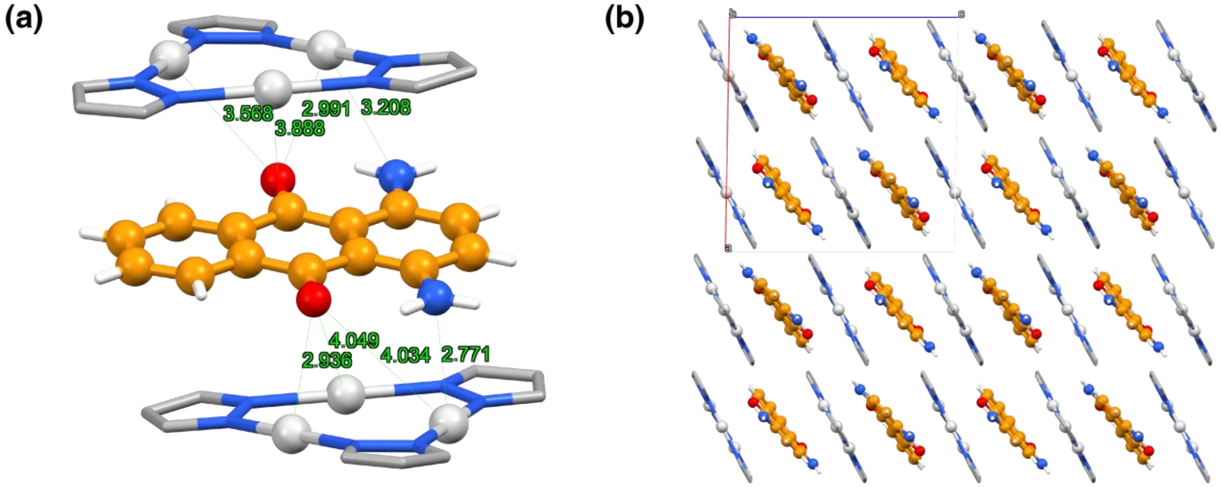

Figure S266. (a) A schematic diagram of the co-crystal structure in the **Ag_3Pz_3·83** single crystal, formed by the guest organic molecule and the surrounding Ag_3Pz_3 units that exhibit significant interactions with it. (b) A 2 × 1 × 2 packing mode in the single crystal structure of **Ag_3Pz_3·83** along the *b* axis. Trifluoromethyl groups and H atoms in Ag_3Pz_3 are omitted for clarity. Ag···N and Ag···O interactions are indicated with green dotted lines with distances in Å. C, N, and Ag atoms in Ag_3Pz_3 are depicted in dark gray, light blue, and light gray, respectively; C, O, N, and H atoms in **83** are depicted in orange, red, light blue, and white, respectively.

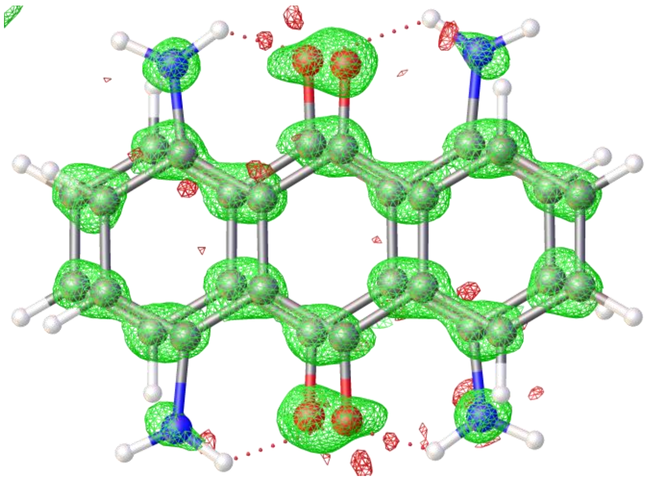

Figure S267. F_{obs} (contour: 0.20) electron density map superimposed on the structure of **83** in the single crystal structure of **Ag_3Pz_3·83**. We believe that the unassigned electron density is attributable to the Ag_3Pz_3 units. Please note that there is a twofold disorder in the molecule **83**, likely due to molecular symmetry.

Preparation of Ag_3Pz_3·84. 1.33 mg (0.0107 mmol) of methylphenylsulfide (**84**) was dissolved in 3 mL of a binary solvent system of DCM and MeOH (1:1, v/v), followed by the addition of equimolar amounts of Ag_3Pz_3 (10.00 mg, 0.0107 mmol). The resulting mixed solution was filtered and then transferred to a 20 mL screw-capped sample vial. The cap of the sample vial was loosely closed to allow the solvent to slowly evaporate at room temperature. The entire co-crystal incubation process was protected from light using aluminum foil. After the designated evaporation period, typically 1-3 days, high-quality colorless block-shaped crystals suitable for single-crystal X-ray diffraction analysis formed at the bottom of the vial.

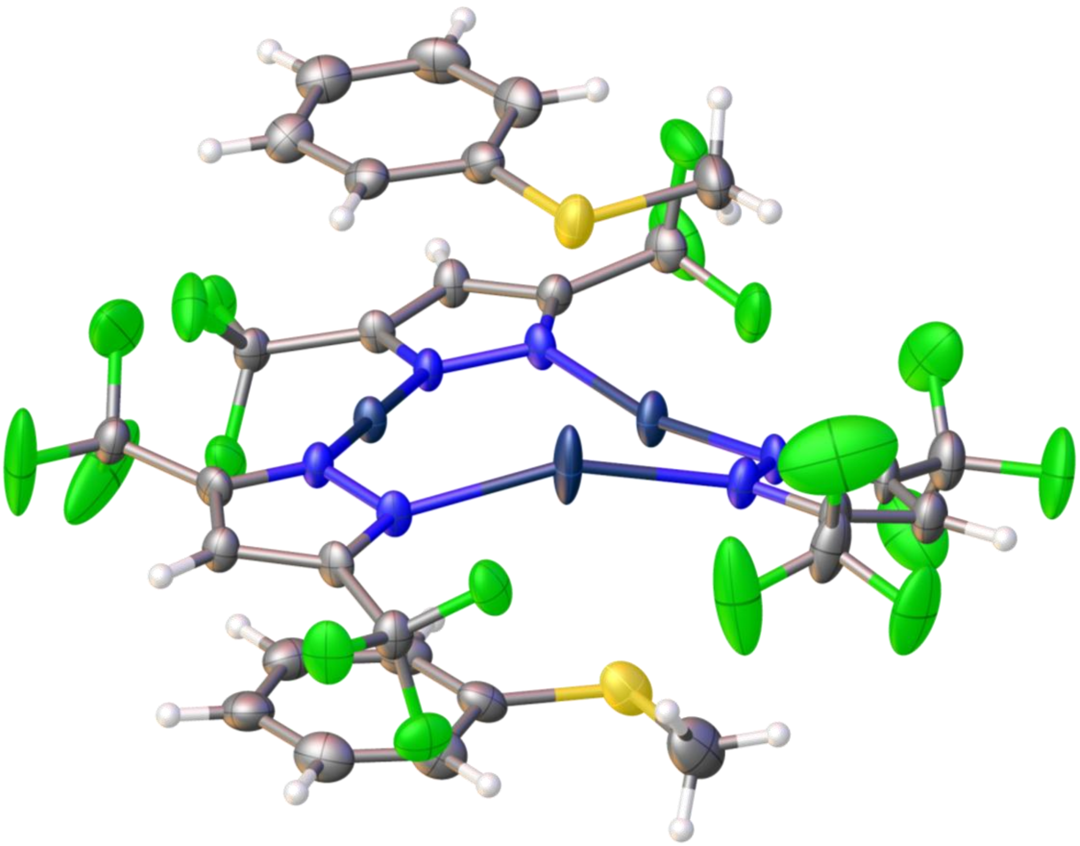

Figure S268. Asymmetric unit of **Ag_3Pz_3·84** (thermal displacement parameters at the 50% probability level).

Table S92. Crystal data and structure refinement for **Ag_3Pz_3·84**

Empirical formula	$C_{29}H_{19}Ag_3F_{17.99}N_6S_2$
Formula weight	1181.09
Temperature/K	99.99(16)
Crystal system	monoclinic
Space group	$P2_1/n$
a/Å	7.55370(10)
b/Å	24.6709(2)
c/Å	19.7226(2)
α/°	90
β/°	93.4520(10)
γ/°	90
Volume/Å^3	3668.77(7)
Z	4
ρ_{calc}g/cm^3	2.138
μ/mm^{-1}	14.954
F(000)	2280.0
Crystal size/mm^3	0.3 × 0.2 × 0.1
Radiation	Cu Kα (λ = 1.54184)
2θ range for data collection/°	5.744 to 155.71
Index ranges	$-9 \le h \le 9$, $-29 \le k \le 30$, $-24 \le l \le 24$
Reflections collected	25374
Independent reflections	7589 [R_{int} = 0.0652, R_{sigma} = 0.0506]
Data/restraints/parameters	7589/6/526
Goodness-of-fit on F^2	1.061
Final R indexes [I>=2σ (I)]	R_1 = 0.0635, wR_2 = 0.1700
Final R indexes [all data]	R_1 = 0.0678, wR_2 = 0.1748
Largest diff. peak/hole / e Å^{-3}	1.54/-2.23
CCDC-number	2501871

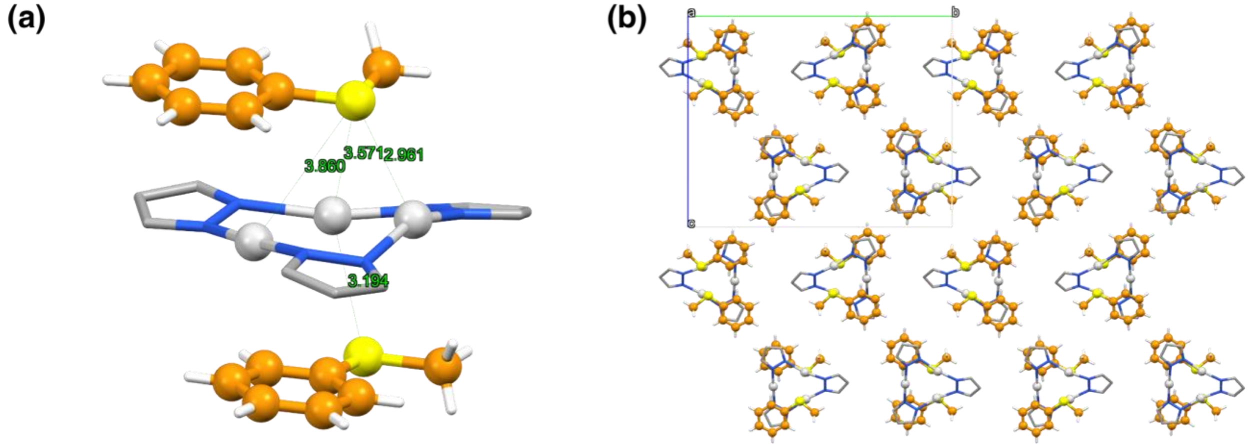

Figure S269. (a) A schematic diagram of the co-crystal structure in the **Ag_3Pz_3·84** single crystal, formed by the guest organic molecule and the surrounding Ag_3Pz_3 units that exhibit significant interactions with it. (b) A 1 × 2 × 2 packing mode in the single crystal structure of **Ag_3Pz_3·84** along the *a* axis. Trifluoromethyl groups and H atoms in Ag_3Pz_3 are omitted for clarity. Ag···S interactions are indicated with green dotted lines with distances in Å. C, N, and Ag atoms in Ag_3Pz_3 are depicted in dark gray, light blue, and light gray, respectively; C, S, and H atoms in **84** are depicted in orange, yellow, and white, respectively.

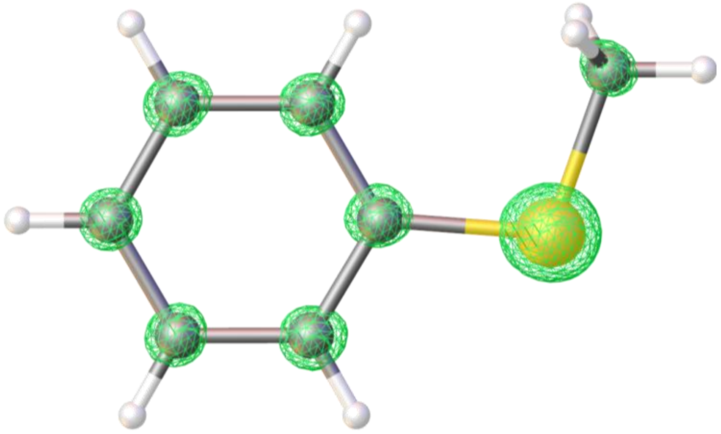

Figure S270. F_{obs} (contour: 0.85) electron density map superimposed on the structure of **84** in the single crystal structure of **Ag_3Pz_3·84**.

Preparation of Ag_3Pz_3·85. 2.17 mg (0.0107 mmol) of 4-bromothioanisole (**85**) was dissolved in 3 mL of n-Hex, followed by the addition of equimolar amounts of Ag_3Pz_3 (10.00 mg, 0.0107 mmol). The resulting mixed solution was filtered and then transferred to a 20 mL screw-capped sample vial. The cap of the sample vial was loosely closed to allow the solvent to slowly evaporate at room temperature. The entire co-crystal incubation process was protected from light using aluminum foil. After the designated evaporation period, typically 1-3 days, high-quality colorless needle-shaped crystals suitable for single-crystal X-ray diffraction analysis formed at the bottom of the vial.

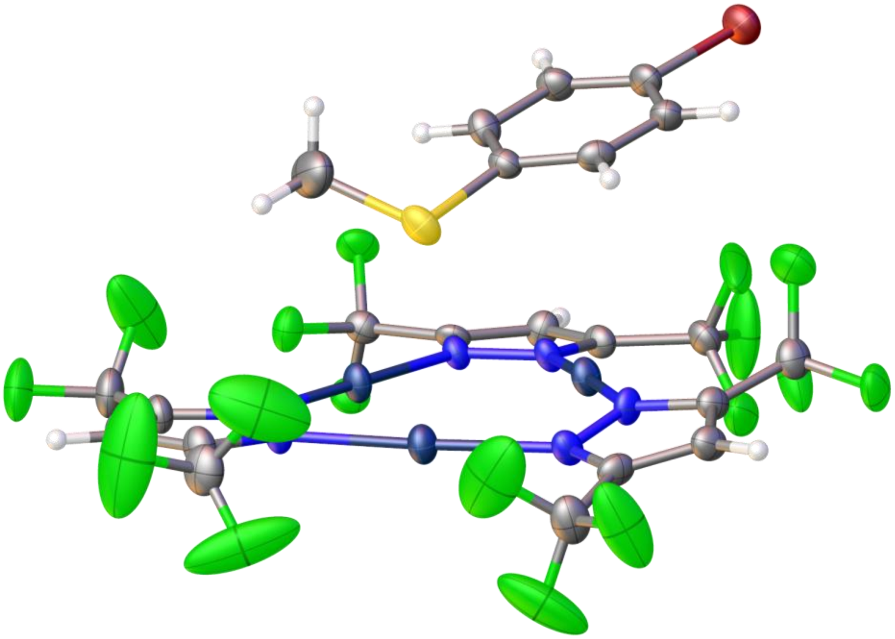

Figure S271. Asymmetric unit of **Ag_3Pz_3·85** (thermal displacement parameters at the 50% probability level).

Table S93. Crystal data and structure refinement for **Ag_3Pz_3·85**

Empirical formula	$C_{22}H_{10}Ag_3BrF_{18}N_6S$
Formula weight	1135.94
Temperature/K	100.01(14)
Crystal system	monoclinic
Space group	$P2_1/c$
a/Å	9.01820(10)
b/Å	18.21110(10)
c/Å	19.55040(10)
α/°	90
β/°	94.2910(10)
γ/°	90
Volume/Å^3	3201.79(4)
Z	4
ρ_{calc}g/cm^3	2.357
μ/mm^{-1}	17.910
F(000)	2152.0
Crystal size/mm^3	0.25 × 0.23 × 0.15
Radiation	Cu Kα (λ = 1.54184)
2θ range for data collection/°	6.642 to 156.75
Index ranges	$-10 \leq h \leq 11$, $-21 \leq k \leq 22$, $-24 \leq l \leq 24$
Reflections collected	19080
Independent reflections	6634 [R_{int} = 0.0280, R_{sigma} = 0.0315]
Data/restraints/parameters	6634/0/461
Goodness-of-fit on F^2	1.050
Final R indexes [I>=2σ (I)]	R_1 = 0.0448, wR_2 = 0.1104
Final R indexes [all data]	R_1 = 0.0504, wR_2 = 0.1136
Largest diff. peak/hole / e Å^{-3}	2.09/-1.17
CCDC-number	2501872

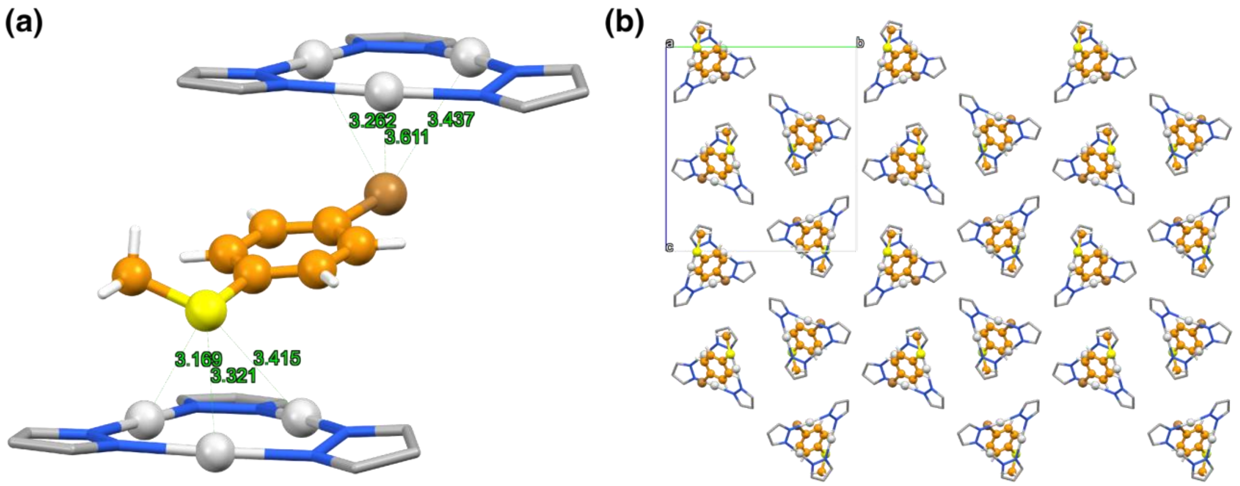

Figure S272. (a) A schematic diagram of the co-crystal structure in the $\mathbf{Ag_3Pz_3}$·**85** single crystal, formed by the guest organic molecule and the surrounding Ag_3Pz_3 units that exhibit significant interactions with it. (b) A 1 × 3 × 2 packing mode in the single crystal structure of $\mathbf{Ag_3Pz_3}$·**85** along the *a* axis. Trifluoromethyl groups and H atoms in Ag_3Pz_3 are omitted for clarity. Ag···S and Ag···Br interactions are indicated with green dotted lines with distances in Å. C, N, and Ag atoms in Ag_3Pz_3 are depicted in dark gray, light blue, and light gray, respectively; C, S, Br, and H atoms in **85** molecules are depicted in orange, yellow, brown, and white, respectively.

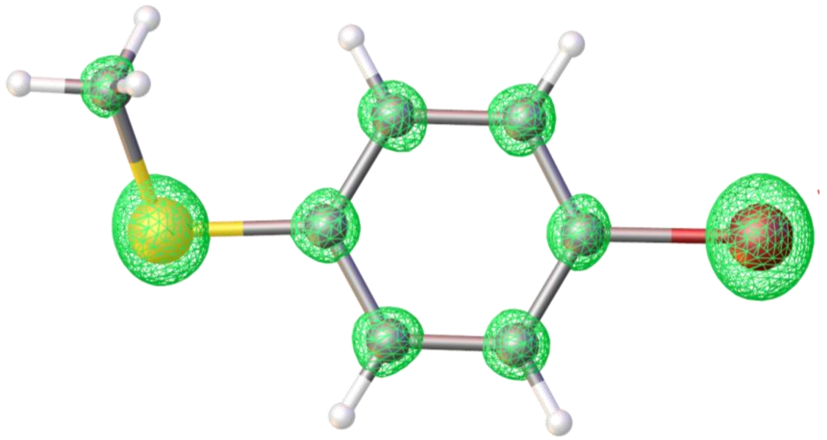

Figure S273. F_{obs} (contour: 0.75) electron density map superimposed on the structure of **85** in the single crystal structure of $\mathbf{Ag_3Pz_3}$·**85**.

Preparation of Ag_3Pz_3·86. 1.35 mg (0.0107 mmol) of 2-acetylthiophene (**86**) was dissolved in 3 mL of n-Hex, followed by the addition of equimolar amounts of Ag_3Pz_3 (10.00 mg, 0.0107 mmol). The resulting mixed solution was filtered and then transferred to a 20 mL screw-capped sample vial. The cap of the sample vial was loosely closed to allow the solvent to slowly evaporate at room temperature. The entire co-crystal incubation process was protected from light using aluminum foil. After the designated evaporation period, typically 1-3 days, high-quality colorless block-shaped crystals suitable for single-crystal X-ray diffraction analysis formed at the bottom of the vial.

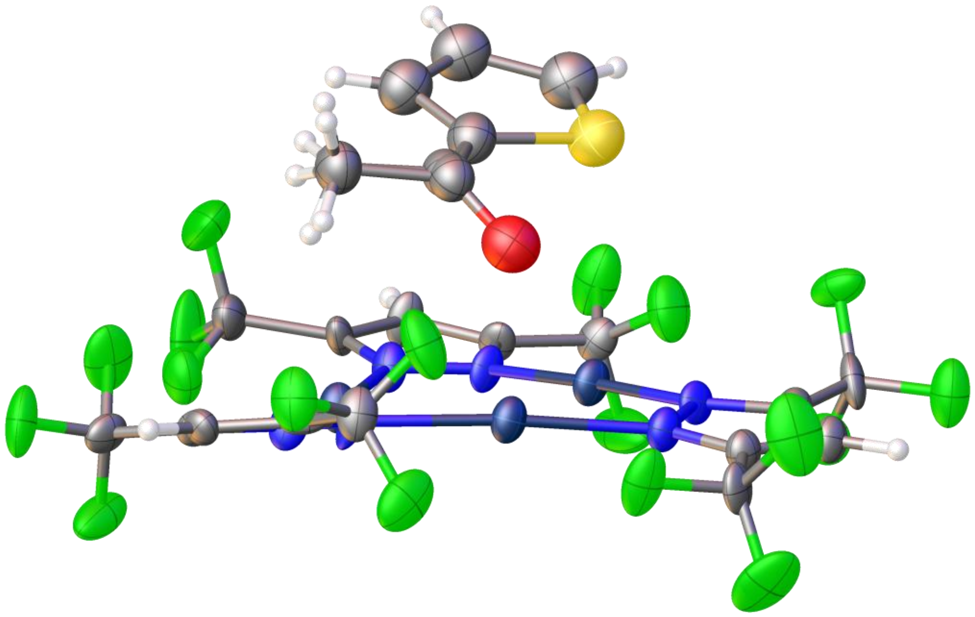

Figure S274. Asymmetric unit of **Ag_3Pz_3·86** (thermal displacement parameters at the 50% probability level).

Table S94. Crystal data and structure refinement for **Ag_3Pz_3·86**

Empirical formula	$C_{21}H_9Ag_3F_{18}N_6OS$
Formula weight	1059.01
Temperature/K	100.02(18)
Crystal system	orthorhombic
Space group	$Pna2_1$
a/Å	12.5401(2)
b/Å	21.2666(2)
c/Å	11.22290(10)
α/°	90
β/°	90
γ/°	90
Volume/Å^3	2992.98(6)
Z	4
ρ_{calc}g/cm^3	2.350
μ/mm^{-1}	17.610
F(000)	2016.0
Crystal size/mm^3	0.17 × 0.16 × 0.16
Radiation	Cu Kα (λ = 1.54184)
2θ range for data collection/°	8.186 to 157.26
Index ranges	$-15 \leq h \leq 15$, $-24 \leq k \leq 26$, $-14 \leq l \leq 11$
Reflections collected	14313
Independent reflections	4675 [R_{int} = 0.0542, R_{sigma} = 0.0548]
Data/restraints/parameters	4675/140/455
Goodness-of-fit on F^2	1.150
Final R indexes [I>=2σ (I)]	R_1 = 0.0770, wR_2 = 0.1883
Final R indexes [all data]	R_1 = 0.0885, wR_2 = 0.1937
Largest diff. peak/hole / e Å^{-3}	3.40/-1.70
Flack parameter	-0.03(2)
CCDC-number	2501873

Responses to CheckCIF alerts for Ag_3Pz_3·86 crystal structure:

(There is no A-level alert)

B-level alerts:

"Low Bond Precision on C-C Bonds .... 0.03133 Ang."

Disordered structure.

"Check Calcd Resid. Dens. 0.23Ang From C1 3.42 eA-3"

Possible low grade disorder/absorption effects.

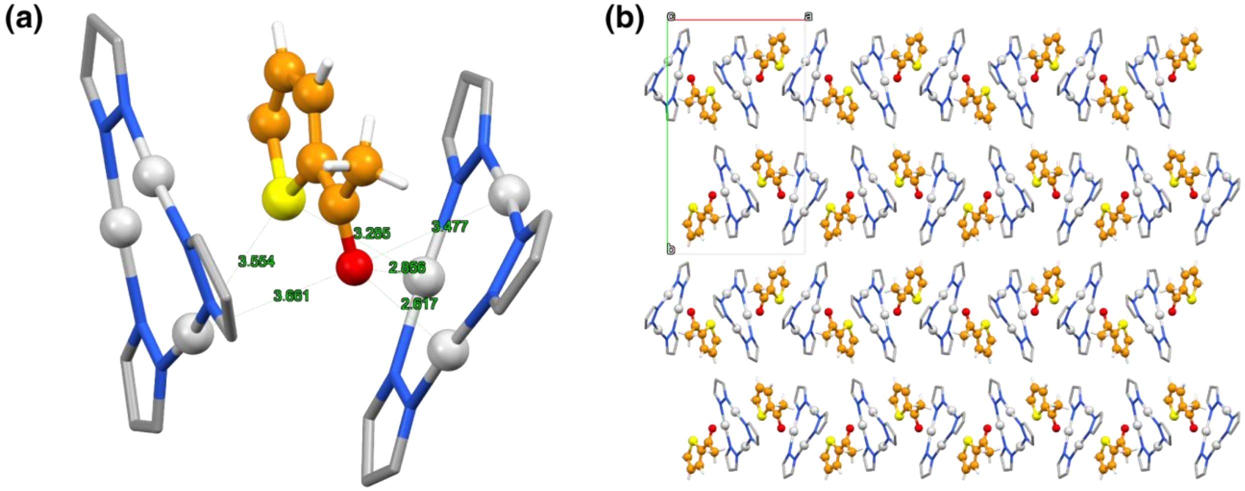

Figure S275. (a) A schematic diagram of the co-crystal structure in the **Ag_3Pz_3·86** single crystal, formed by the guest organic molecule and the surrounding Ag_3Pz_3 units that exhibit significant interactions with it. (b) A 4 × 2 × 1 packing mode in the single crystal structure of **Ag_3Pz_3·86** along the *c* axis. Trifluoromethyl groups and H atoms in Ag_3Pz_3 are omitted for clarity. Ag···S and Ag···O interactions are indicated with green dotted lines with distances in Å. C, N, and Ag atoms in Ag_3Pz_3 are depicted in dark gray, light blue, and light gray, respectively; C, S, O, and H atoms in **86** are depicted in orange, yellow, red, and white, respectively.

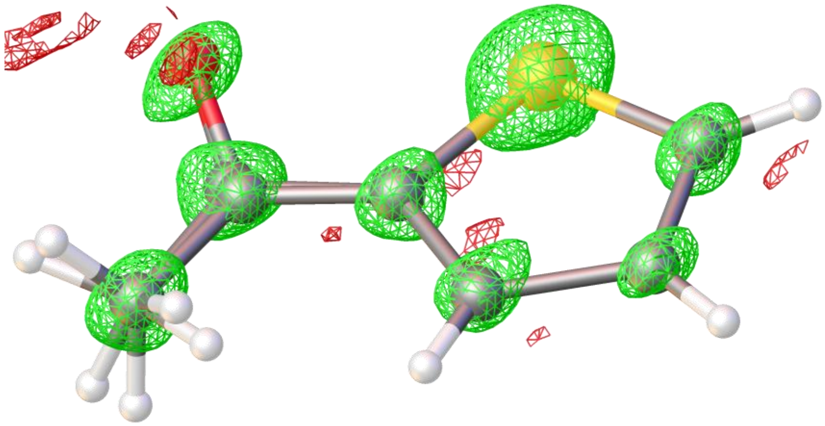

Figure S276. F_{obs} (contour: 0.60) electron density map superimposed on the structure of **86** in the single crystal structure of **Ag_3Pz_3·86**. Please note that there is a twofold disorder in the molecule **86** which is due to molecular vibrations.

Preparation of Ag_3Pz_3·87. 2.13 mg (0.0107 mmol) of phenothiazine (**87**) was dissolved in 3 mL of a binary solvent system of DCM and n-Hex (1:1, v/v), followed by the addition of equimolar amounts of Ag_3Pz_3 (10.00 mg, 0.0107 mmol). The resulting mixed solution was filtered and then transferred to a 20 mL screw-capped sample vial. The cap of the sample vial was loosely closed to allow the solvent to slowly evaporate at room temperature. The entire co-crystal incubation process was protected from light using aluminum foil. After the designated evaporation period, typically 1-3 days, high-quality colorless needle-shaped crystals suitable for single-crystal X-ray diffraction analysis formed at the bottom of the vial.

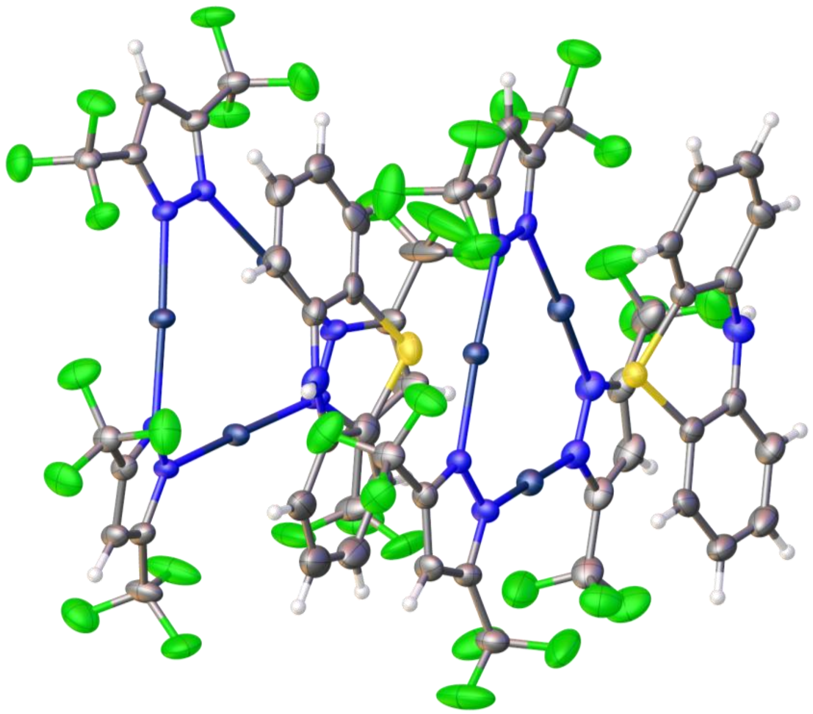

Figure S277. Asymmetric unit of **Ag_3Pz_3·87** (thermal displacement parameters at the 50% probability level).

Table S95. Crystal data and structure refinement for $\mathbf{Ag_3Pz_3 \cdot 87}$

Empirical formula	$C_{27}H_{12}Ag_3F_{18}N_7S$
Formula weight	1132.11
Temperature/K	100.01(16)
Crystal system	monoclinic
Space group	$P2_1/c$
a/Å	21.9920(2)
b/Å	12.75940(10)
c/Å	24.7580(3)
α/°	90
β/°	103.8390(10)
γ/°	90
Volume/Å^3	6745.54(12)
Z	8
ρ_{calc}g/cm^3	2.230
μ/mm^{-1}	15.676
F(000)	4336.0
Crystal size/mm^3	0.2 × 0.2 × 0.2
Radiation	Cu Kα (λ = 1.54184)
2θ range for data collection/°	7.354 to 156.804
Index ranges	$-27 \leq h \leq 27$, $-11 \leq k \leq 16$, $-30 \leq l \leq 22$
Reflections collected	32037
Independent reflections	13687 [R_{int} = 0.0423, R_{sigma} = 0.0518]
Data/restraints/parameters	13687/0/1009
Goodness-of-fit on F^2	1.049
Final R indexes [I>=2σ (I)]	R_1 = 0.0495, wR_2 = 0.1384
Final R indexes [all data]	R_1 = 0.0579, wR_2 = 0.1435
Largest diff. peak/hole / e Å^{-3}	1.28/-1.36
CDCC-number	2501875

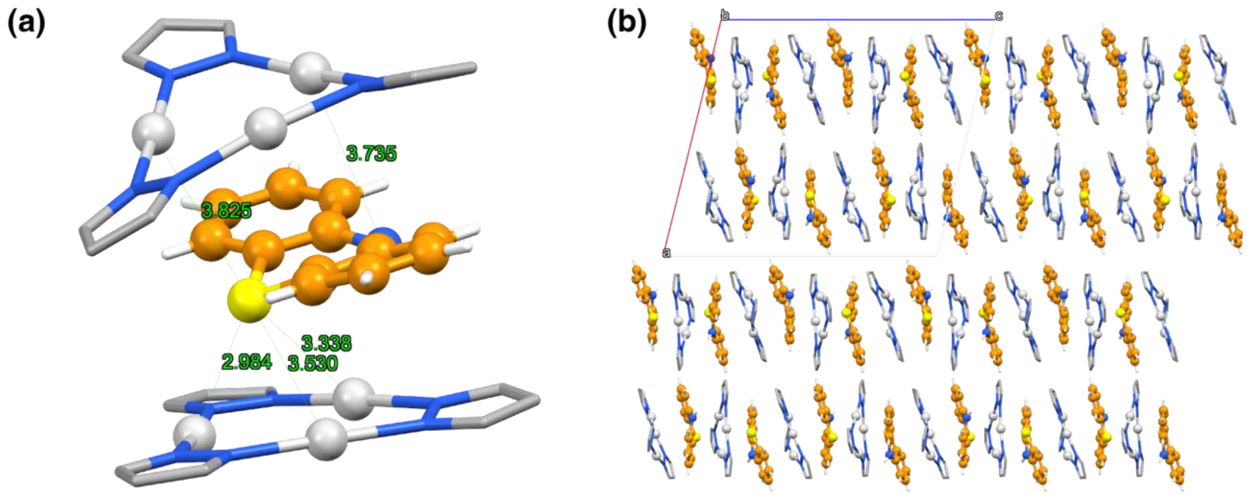

Figure S278. (a) A schematic diagram of the co-crystal structure in the $\mathbf{Ag_3Pz_3}$**·87** single crystal, formed by the guest organic molecule and the surrounding Ag_3Pz_3 units that exhibit significant interactions with it. (b) A 2 × 1 × 2 packing mode in the single crystal structure of $\mathbf{Ag_3Pz_3}$**·87** along the *b* axis. Trifluoromethyl groups and H atoms in Ag_3Pz_3 are omitted for clarity. Ag···S and Ag···N interactions are indicated with green dotted lines with distances in Å. C, N, and Ag atoms in Ag_3Pz_3 are depicted in dark gray, light blue, and light gray, respectively; C, S, N, and H atoms in **87** are depicted in orange, yellow, light blue, and white, respectively.

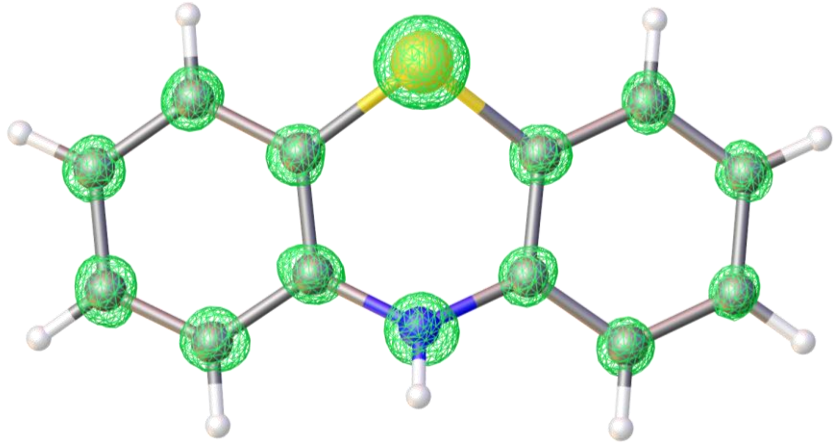

Figure S279. F_{obs} (contour: 0.40) electron density map superimposed on the structure of **87** in the single crystal structure of $\mathbf{Ag_3Pz_3}$**·87**.

Preparation of Ag_3Pz_3·88. 2.50 mg (0.0107 mmol) of 2-chlorophenothiazine (**88**) was dissolved in 3 mL of c-Hex, followed by the addition of equimolar amounts of Ag_3Pz_3 (10.00 mg, 0.0107 mmol). The resulting mixed solution was filtered and then transferred to a 20 mL screw-capped sample vial. The cap of the sample vial was loosely closed to allow the solvent to slowly evaporate at room temperature. The entire co-crystal incubation process was protected from light using aluminum foil. After the designated evaporation period, typically 1-3 days, high-quality colorless needle-shaped crystals suitable for single-crystal X-ray diffraction analysis formed at the bottom of the vial.

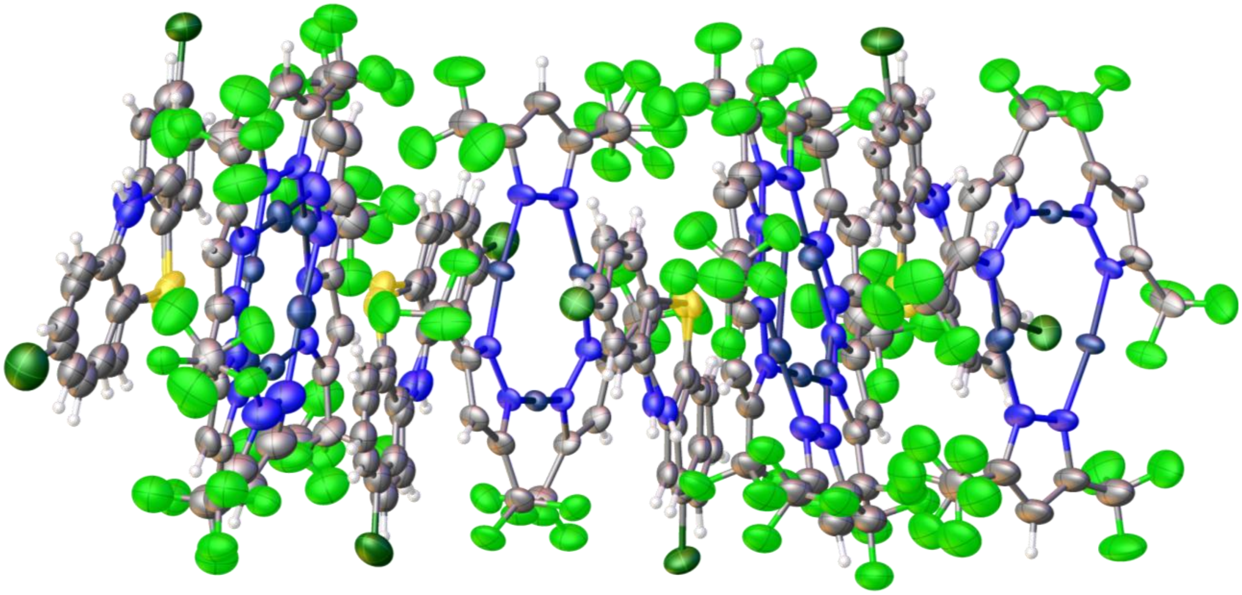

Figure S280. Asymmetric unit of **Ag_3Pz_3·88** (thermal displacement parameters at the 50% probability level).

Table S96. Crystal data and structure refinement for **Ag_3Pz_3·88**

Empirical formula	$C_{108}H_{44}Ag_{12}Cl_4F_{72}N_{28}S_4$
Formula weight	4666.19
Temperature/K	100.00(17)
Crystal system	triclinic
Space group	$P\bar{1}$
a/Å	13.0986(2)
b/Å	22.5585(3)
c/Å	25.0454(4)
α/°	109.3260(10)
β/°	92.4980(10)
γ/°	90.3520(10)
Volume/Å^3	6975.22(18)
Z	2
ρ_{calc}g/cm^3	2.222
μ/mm^{-1}	15.876
F(000)	4464.0
Crystal size/mm^3	0.27 × 0.26 × 0.16
Radiation	Cu Kα (λ = 1.54184)
2θ range for data collection/°	4.576 to 157.206
Index ranges	$-16 \le h \le 14$, $-28 \le k \le 21$, $-31 \le l \le 31$
Reflections collected	64839
Independent reflections	28369 [R_{int} = 0.0648, R_{sigma} = 0.0838]
Data/restraints/parameters	28369/9142/3060
Goodness-of-fit on F^2	1.048
Final R indexes [I>=2σ (I)]	R_1 = 0.1102, wR_2 = 0.2547
Final R indexes [all data]	R_1 = 0.1473, wR_2 = 0.2671
Largest diff. peak/hole / e Å^{-3}	2.10/-1.83
CCDC-number	2501877

Responses to CheckCIF alerts for Ag_3Pz_3·88 crystal structure:

(There is no A-level alert)

B-level alerts:

"Low Bond Precision on C-C Bonds .... 0.02165 Ang."

Disordered structure.

"Check Calcd Resid. Dens. 0.93Ang From Ag2 2.61 eA-3"

This Alert is due to presence of residual density in the presence of heavy metal atom (Ag).

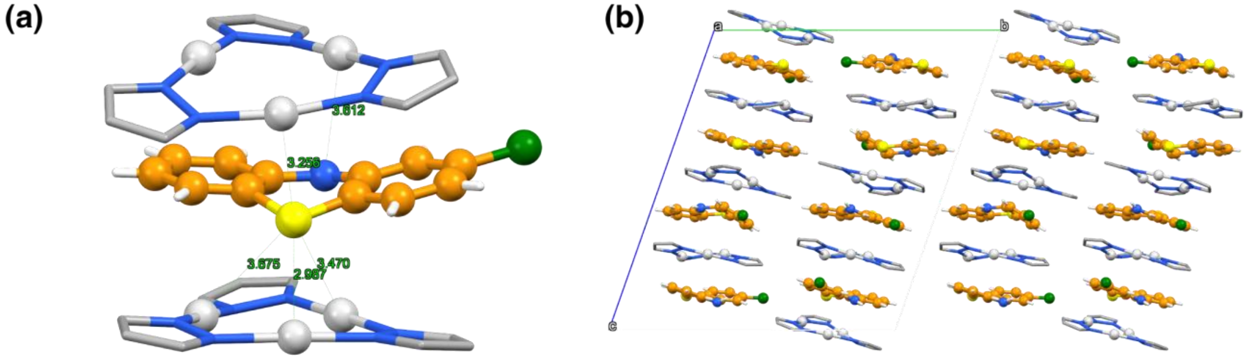

Figure S281. (a) A schematic diagram of the co-crystal structure in the **Ag_3Pz_3·88** single crystal, formed by the guest organic molecule and the surrounding Ag_3Pz_3 units that exhibit significant interactions with it. (b) A 1 × 2 × 1 packing mode in the single crystal structure of **Ag_3Pz_3·88** along the *a* axis. Trifluoromethyl groups and H atoms in Ag_3Pz_3 are omitted for clarity. Ag···S and Ag···N interactions are indicated with green dotted lines with distances in Å. C, N, and Ag atoms in Ag_3Pz_3 are depicted in dark gray, light blue, and light gray, respectively; C, S, N, Cl, and H atoms in **88** are depicted in orange, yellow, light blue, green, and white, respectively.

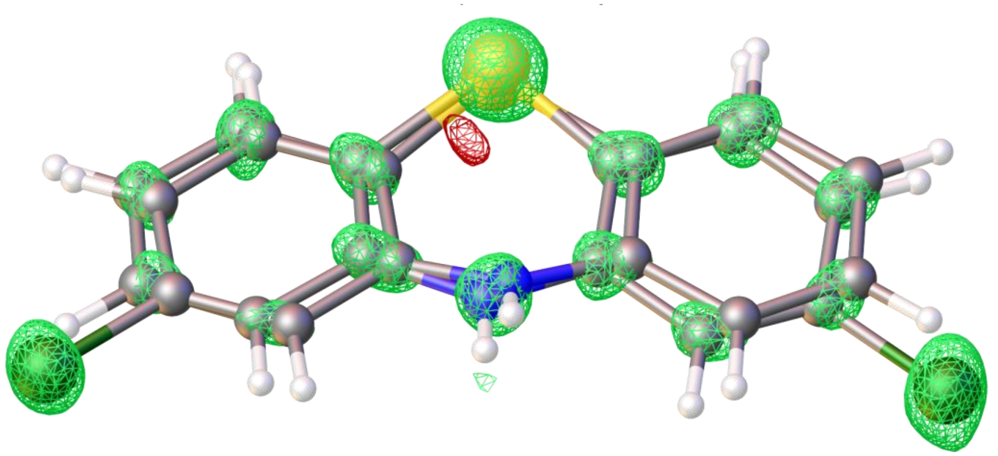

Figure S282. F_{obs} (contour: 0.35) electron density map superimposed on the structure of **88** in the single crystal structure of **Ag_3Pz_3·88**. Please note that there is a twofold disorder in the molecule **88** which is due to molecular vibrations.

Preparation of Ag_3Pz_3·89. 2.58 mg (0.0107 mmol) of 2-acetylphenothiazine (**89**) was dissolved in 3 mL of DCM, followed by the addition of equimolar amounts of Ag_3Pz_3 (10.00 mg, 0.0107 mmol). The resulting mixed solution was filtered and then transferred to a 20 mL screw-capped sample vial. The cap of the sample vial was loosely closed to allow the solvent to slowly evaporate at room temperature. The entire co-crystal incubation process was protected from light using aluminum foil. After the designated evaporation period, typically 1-3 days, high-quality colorless needle-shaped crystals suitable for single-crystal X-ray diffraction analysis formed at the bottom of the vial.

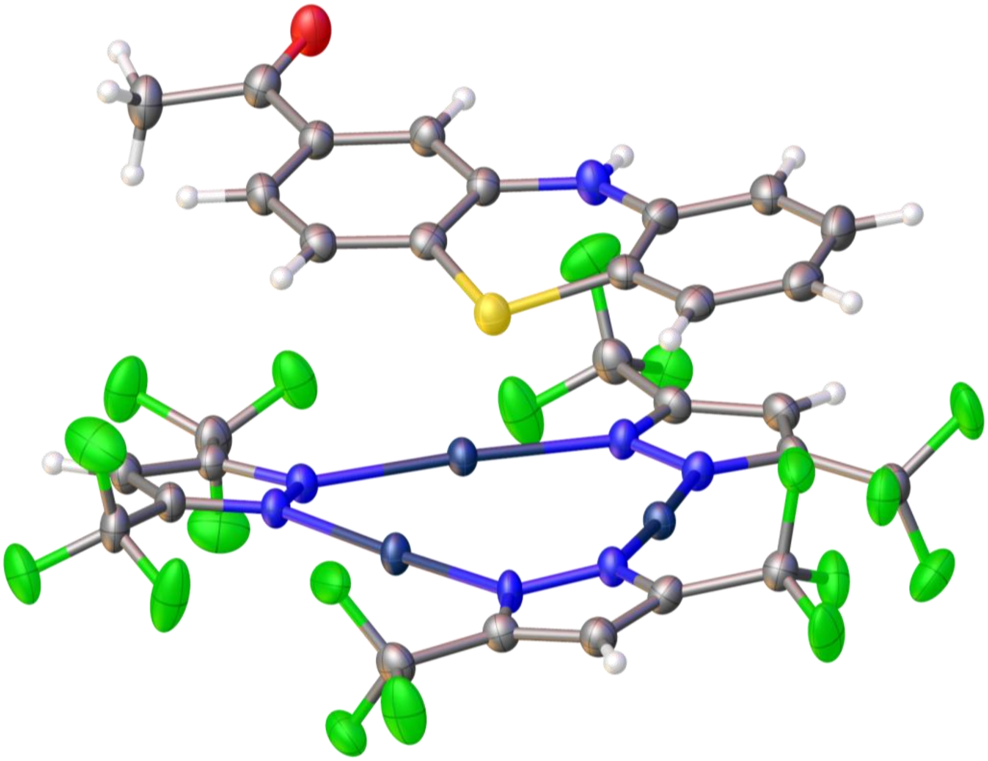

Figure S283. Asymmetric unit of **Ag_3Pz_3·89** (thermal displacement parameters at the 50% probability level).

Table S97. Crystal data and structure refinement for **Ag_3Pz_3·89**

Empirical formula	$C_{29}H_{14}Ag_3F_{18}N_7OS$
Formula weight	1174.14
Temperature/K	100.01(19)
Crystal system	monoclinic
Space group	$P2_1/n$
a/Å	13.56300(10)
b/Å	11.35740(10)
c/Å	22.58400(10)
α/°	90
β/°	92.5570(10)
γ/°	90
Volume/Å^3	3475.38(4)
Z	4
ρ_{calc}g/cm^3	2.244
μ/mm^{-1}	15.269
F(000)	2256.0
Crystal size/mm^3	0.3 × 0.2 × 0.1
Radiation	Cu Kα (λ = 1.54184)
2θ range for data collection/°	7.76 to 155.566
Index ranges	$-16 \leq h \leq 16$, $-14 \leq k \leq 14$, $-28 \leq l \leq 24$
Reflections collected	22216
Independent reflections	7167 [R_{int} = 0.0410, R_{sigma} = 0.0345]
Data/restraints/parameters	7167/0/533
Goodness-of-fit on F^2	1.056
Final R indexes [I>=2σ (I)]	R_1 = 0.0400, wR_2 = 0.1134
Final R indexes [all data]	R_1 = 0.0414, wR_2 = 0.1148
Largest diff. peak/hole / e Å^{-3}	1.69/-1.08
CCDC-number	2501878

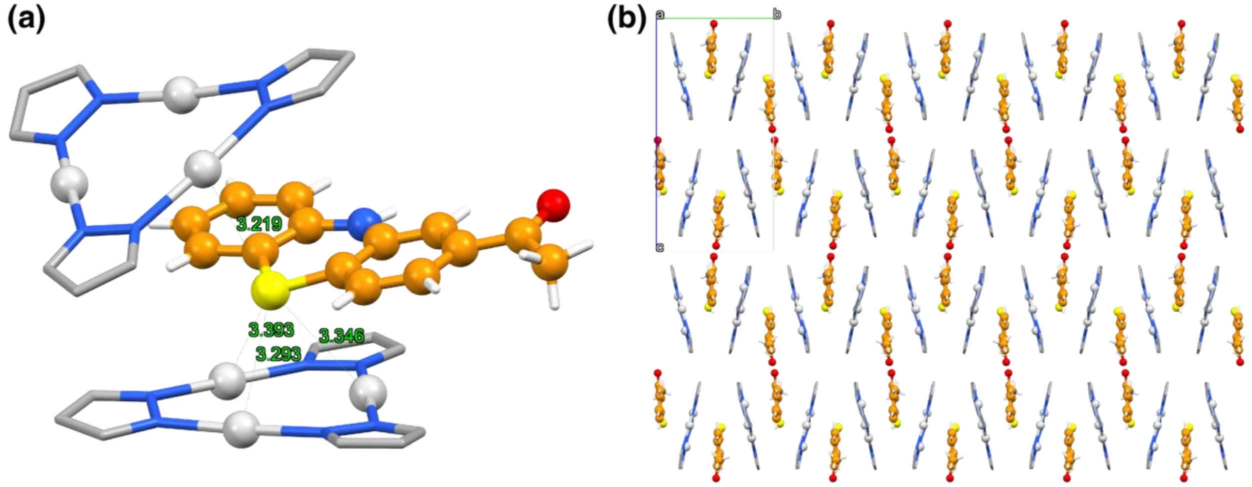

Figure S284. (a) A schematic diagram of the co-crystal structure in the **Ag_3Pz_3·89** single crystal, formed by the guest organic molecule and the surrounding Ag_3Pz_3 units that exhibit significant interactions with it. (b) A 1 × 5 × 2 packing mode in the single crystal structure of **Ag_3Pz_3·89** along the *a* axis. Trifluoromethyl groups and H atoms in Ag_3Pz_3 are omitted for clarity. Ag···S interactions are indicated with green dotted lines with distances in Å. C, N, and Ag atoms in Ag_3Pz_3 are depicted in dark gray, light blue, and light gray, respectively; C, S, N, O, and H atoms in **89** are depicted in orange, yellow, light blue, red, and white, respectively.

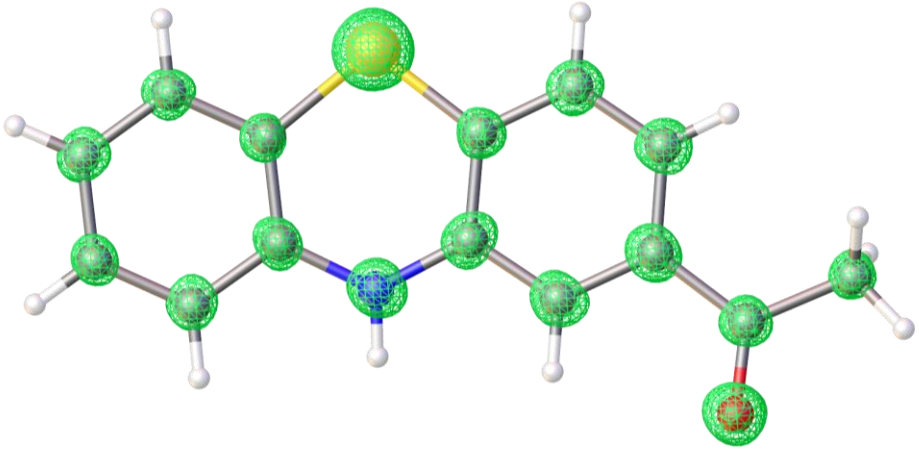

Figure S285. F_{obs} (contour: 0.90) electron density map superimposed on the structure of **89** in the single crystal structure of **Ag_3Pz_3·89**.

Preparation of Ag_3Pz_3·90. 2.95 mg (0.0107 mmol) of 10-phenylphenothiazine (**90**) was dissolved in 3 mL of a binary solvent system of DCM and n-Hex (1:1, v/v), followed by the addition of equimolar amounts of Ag_3Pz_3 (10.00 mg, 0.0107 mmol). The resulting mixed solution was filtered and then transferred to a 20 mL screw-capped sample vial. The cap of the sample vial was loosely closed to allow the solvent to slowly evaporate at room temperature. The entire co-crystal incubation process was protected from light using aluminum foil. After the designated evaporation period, typically 1-3 days, high-quality colorless needle-shaped crystals suitable for single-crystal X-ray diffraction analysis formed at the bottom of the vial.

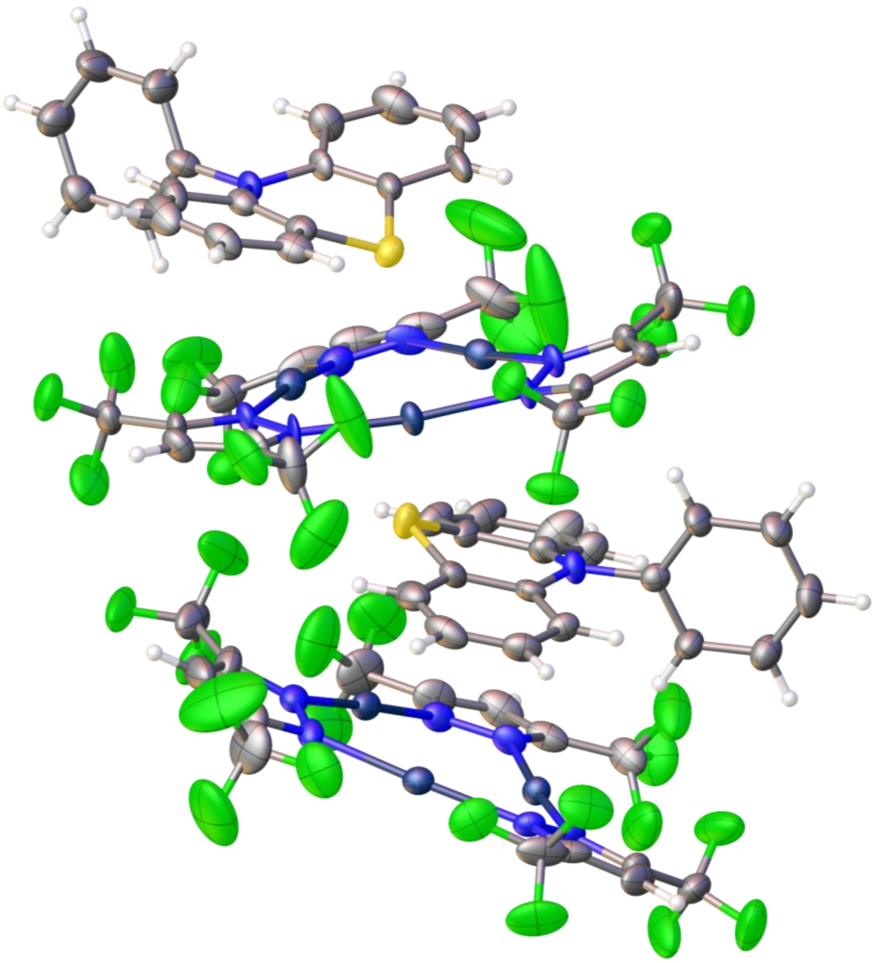

Figure S286. Asymmetric unit of **Ag_3Pz_3·90** (thermal displacement parameters at the 50% probability level).

Table S98. Crystal data and structure refinement for **Ag_3Pz_3·90**

Empirical formula	$C_{33}H_{16}Ag_3F_{18}N_7S$
Formula weight	1208.20
Temperature/K	100.0(2)
Crystal system	monoclinic
Space group	*Cc*
a/Å	14.0984(2)
b/Å	21.3196(3)
c/Å	25.4948(3)
α/°	90
β/°	98.1380(10)
γ/°	90
Volume/Å^3	7585.86(18)
Z	8
ρ_{calc}g/cm^3	2.116
μ/mm^{-1}	13.997
F(000)	4656.0
Crystal size/mm^3	0.2 × 0.2 × 0.2
Radiation	Cu Kα (λ = 1.54184)
2θ range for data collection/°	7.004 to 156.776
Index ranges	$-17 \le h \le 17$, $-22 \le k \le 26$, $-32 \le l \le 20$
Reflections collected	19103
Independent reflections	9807 [R_{int} = 0.0321, R_{sigma} = 0.0351]
Data/restraints/parameters	9807/8/1118
Goodness-of-fit on F^2	1.053
Final R indexes [I>=2σ (I)]	R_1 = 0.0371, wR_2 = 0.1036
Final R indexes [all data]	R_1 = 0.0379, wR_2 = 0.1044
Largest diff. peak/hole / e Å^{-3}	1.33/-0.97
Flack parameter	0.004(10)
CCDC-number	2501879

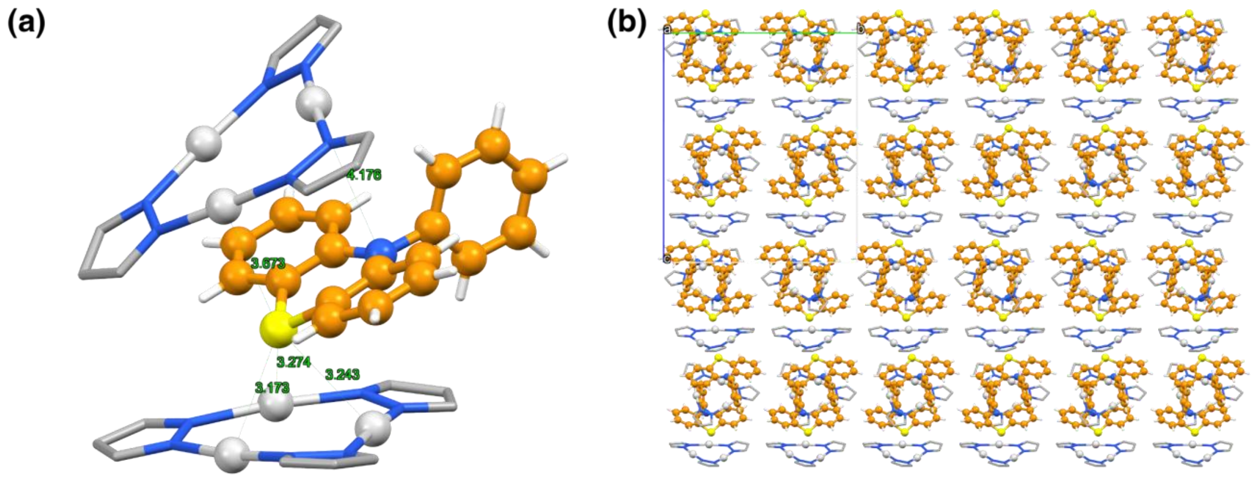

Figure S287. (a) A schematic diagram of the co-crystal structure in the **Ag_3Pz_3·90** single crystal, formed by the guest organic molecule and the surrounding Ag_3Pz_3 units that exhibit significant interactions with it. (b) A 1 × 3 × 2 packing mode in the single crystal structure of **Ag_3Pz_3·90** along the *a* axis. Trifluoromethyl groups and H atoms in Ag_3Pz_3 are omitted for clarity. Ag···S and Ag···N interactions are indicated with green dotted lines with distances in Å. C, N, and Ag atoms in Ag_3Pz_3 are depicted in dark gray, light blue, and light gray, respectively; C, S, N, and H atoms in **90** molecules are depicted in orange, yellow, light blue, and white, respectively.

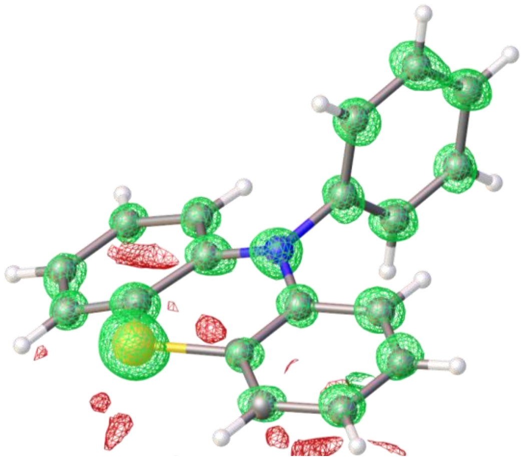

Figure S288. F_{obs} (contour: 0.25) electron density map superimposed on the structure of **90** in the single crystal structure of **Ag_3Pz_3·90**. We believe that the unassigned electron density is attributable to the Ag_3Pz_3 units.

Preparation of Ag_3Pz_3·91. 2.63 mg (0.0107 mmol) of 2-methylthiophenothiazine (**91**) was dissolved in 3 mL of DCM, followed by the addition of equimolar amounts of Ag_3Pz_3 (10.00 mg, 0.0107 mmol). The resulting mixed solution was filtered and then transferred to a 20 mL screw-capped sample vial. The cap of the sample vial was loosely closed to allow the solvent to slowly evaporate at room temperature. The entire co-crystal incubation process was protected from light using aluminum foil. After the designated evaporation period, typically 1-3 days, high-quality colorless needle-shaped crystals suitable for single-crystal X-ray diffraction analysis formed at the bottom of the vial.

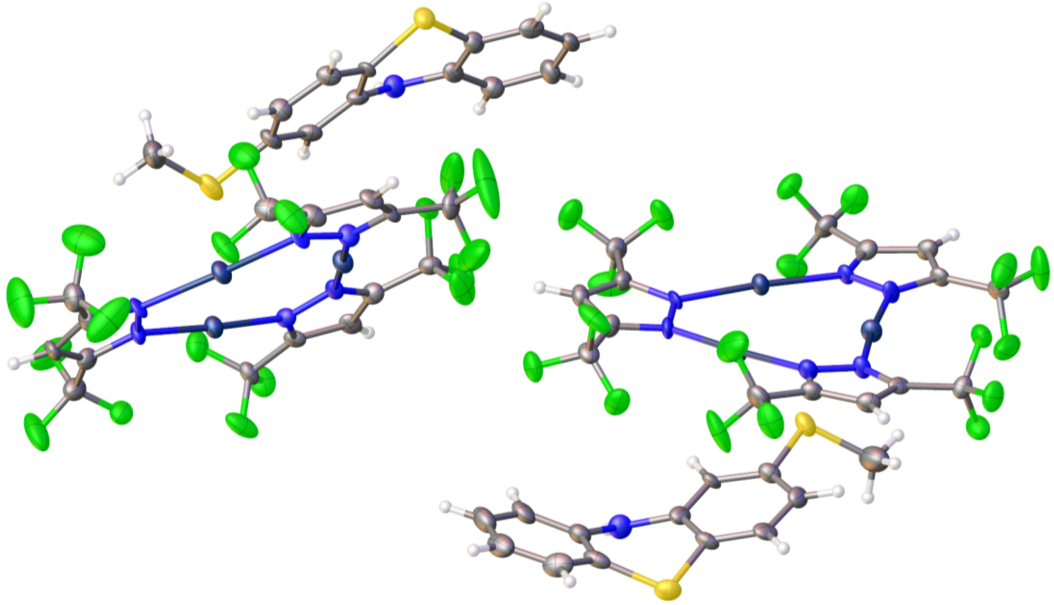

Figure S289. Asymmetric unit of **Ag_3Pz_3·91** (thermal displacement parameters at the 50% probability level).

Table S99. Crystal data and structure refinement for **Ag_3Pz_3·91**

Empirical formula	$C_{28}H_{14}Ag_3F_{18}N_7S_2$
Formula weight	1178.19
Temperature/K	100.00(10)
Crystal system	monoclinic
Space group	$P2_1$
a/Å	9.08510(10)
b/Å	18.7880(2)
c/Å	20.9519(2)
α/°	90
β/°	93.2820(10)
γ/°	90
Volume/Å^3	3570.43(6)
Z	4
ρ_{calc}g/cm^3	2.192
μ/mm^{-1}	15.375
F(000)	2264.0
Crystal size/mm^3	0.2 × 0.1 × 0.1
Radiation	Cu Kα (λ = 1.54184)
2θ range for data collection/°	6.324 to 156.916
Index ranges	$-9 \leq h \leq 11$, $-23 \leq k \leq 18$, $-26 \leq l \leq 24$
Reflections collected	19349
Independent reflections	11667 [R_{int} = 0.0400, R_{sigma} = 0.0534]
Data/restraints/parameters	11667/79/1048
Goodness-of-fit on F^2	1.096
Final R indexes [I>=2σ (I)]	R_1 = 0.0462, wR_2 = 0.1105
Final R indexes [all data]	R_1 = 0.0503, wR_2 = 0.1123
Largest diff. peak/hole / e Å^{-3}	0.97/-1.03
Flack parameter	0.157(11)
CCDC-number	2501880

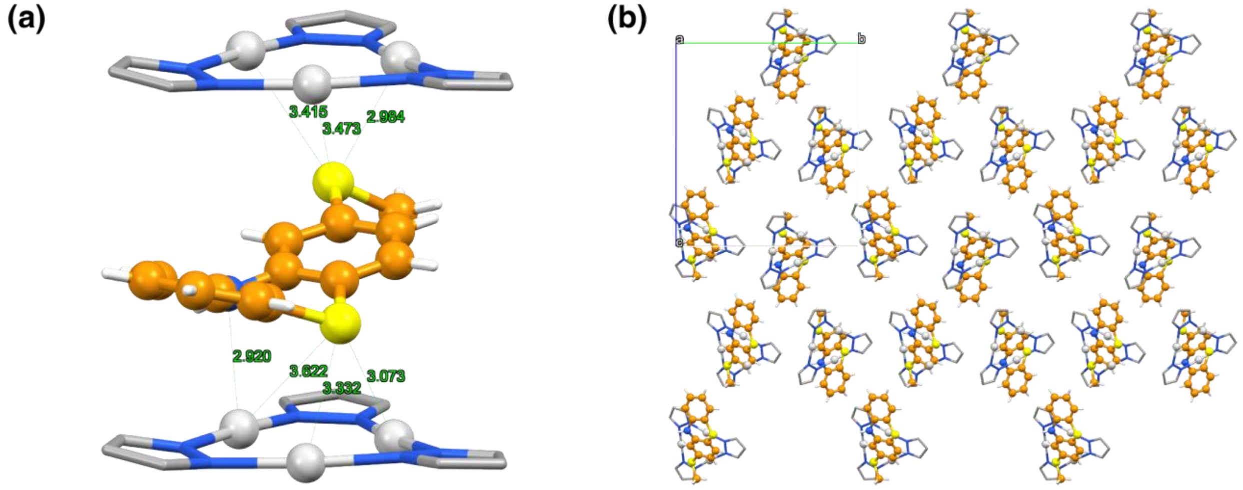

Figure S290. (a) A schematic diagram of the co-crystal structure in the **Ag_3Pz_3·91** single crystal, formed by the guest organic molecule and the surrounding Ag_3Pz_3 units that exhibit significant interactions with it. (b) A 1 × 3 × 2 packing mode in the single crystal structure of **Ag_3Pz_3·91** along the *a* axis. Trifluoromethyl groups and H atoms in Ag_3Pz_3 are omitted for clarity. Ag···S and Ag···N interactions are indicated with green dotted lines with distances in Å. C, N, and Ag atoms in Ag_3Pz_3 are depicted in dark gray, light blue, and light gray, respectively; C, S, N, and H atoms in **91** are depicted in orange, yellow, light blue, and white, respectively.

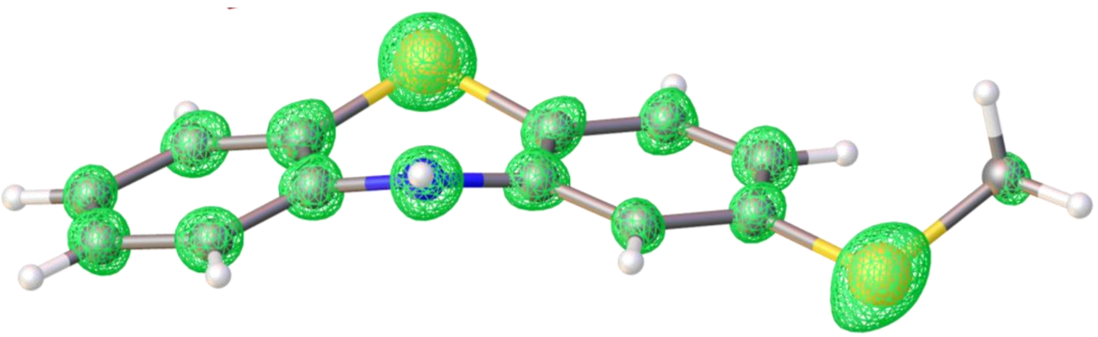

Figure S291. F_{obs} (contour: 0.75) electron density map superimposed on the structure of **91** in the single crystal structure of **Ag_3Pz_3·91**.

Preparation of Ag_3Pz_3·92. 2.31 mg (0.0107 mmol) of thianthrene (**92**) was dissolved in 3 mL of a binary solvent system of DCM and MeOH (1:1, v/v), followed by the addition of equimolar amounts of Ag_3Pz_3 (10.00 mg, 0.0107 mmol). The resulting mixed solution was filtered and then transferred to a 20 mL screw-capped sample vial. The cap of the sample vial was loosely closed to allow the solvent to slowly evaporate at room temperature. The entire co-crystal incubation process was protected from light using aluminum foil. After the designated evaporation period, typically 1-3 days, high-quality colorless block-shaped crystals suitable for single-crystal X-ray diffraction analysis formed at the bottom of the vial.

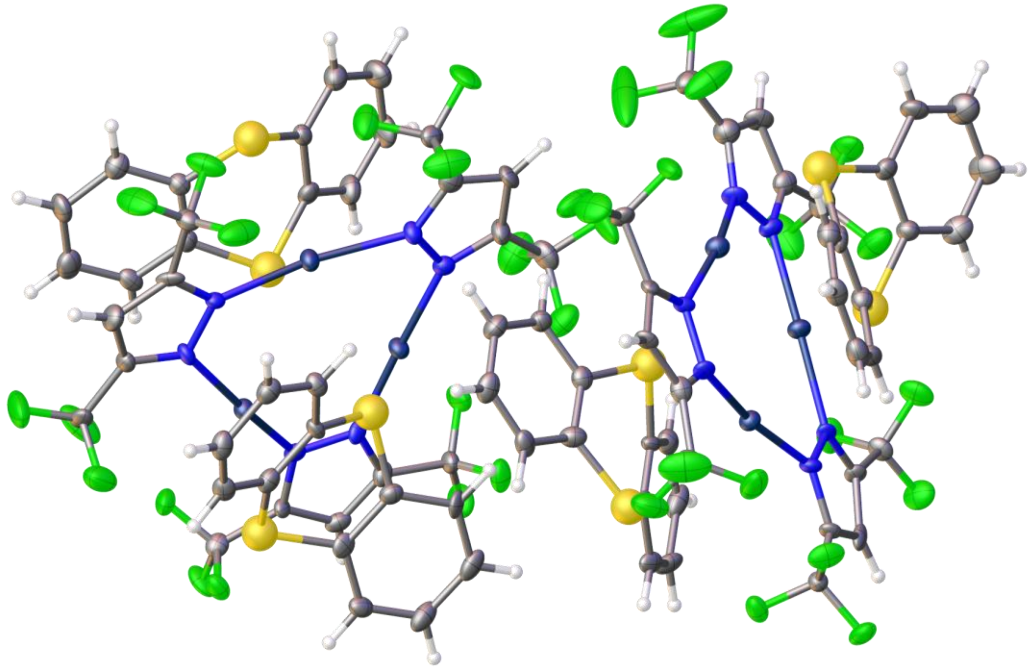

Figure S292. Asymmetric unit of **Ag_3Pz_3·92** (thermal displacement parameters at the 50% probability level).

Table S100. Crystal data and structure refinement for **Ag_3Pz_3·92**

Empirical formula	$C_{39}H_{19}Ag_3F_{18}N_6S_4$
Formula weight	1365.45
Temperature/K	100.15
Crystal system	triclinic
Space group	$P\bar{1}$
a/Å	12.2200(2)
b/Å	12.4005(2)
c/Å	33.2147(4)
α/°	81.7500(10)
β/°	81.5090(10)
γ/°	63.477(2)
Volume/Å^3	4436.95(14)
Z	4
ρ_{calc}g/cm^3	2.044
μ/mm^{-1}	13.343
F(000)	2648.0
Crystal size/mm^3	0.3 × 0.2 × 0.1
Radiation	Cu Kα (λ = 1.54184)
2θ range for data collection/°	8 to 133.198
Index ranges	$-14 \le h \le 14$, $-14 \le k \le 14$, $-39 \le l \le 39$
Reflections collected	74815
Independent reflections	15181 [R_{int} = 0.0775, R_{sigma} = 0.0372]
Data/restraints/parameters	15181/6/1261
Goodness-of-fit on F^2	1.116
Final R indexes [I>=2σ (I)]	R_1 = 0.0950, wR_2 = 0.2893
Final R indexes [all data]	R_1 = 0.0975, wR_2 = 0.2906
Largest diff. peak/hole / e Å^{-3}	5.32/-2.60
CCDC-number	2501881

Responses to CheckCIF alerts for Ag_3Pz_3·92 crystal structure:

A-level alerts:

“Check Calcd Resid. Dens. 1.11Ang From Ag03 5.72 eA-3”

This Alert is due to presence of residual density in the presence of heavy metal atom.

“Check Calcd Resid. Dens. 1.14Ang From Ag06 5.48 eA-3”

This Alert is due to presence of residual density in the presence of heavy metal atom.

“Check Calcd Resid. Dens. 1.18Ang From Ag04 5.10 eA-3”

This Alert is due to presence of residual density in the presence of heavy metal atom.

“Check Calcd Resid. Dens. 1.11Ang From Ag05 4.83 eA-3”

This Alert is due to presence of residual density in the presence of heavy metal atom.

“Check Calcd Resid. Dens. 0.99Ang From N00W 4.80 eA-3”

This Alert is due to presence of residual density in the presence of heavy metal atom.

“Check Calcd Resid. Dens. 0.99Ang From Ag02 4.80 eA-3”

This Alert is due to presence of residual density in the presence of heavy metal atom.

“Check Calcd Resid. Dens. 1.86Ang From F00Y 4.48 eA-3”

This Alert is due to presence of residual density in the presence of heavy metal atom.

“Check Calcd Resid. Dens. 1.45Ang From F00U 4.40 eA-3”

This Alert is due to presence of residual density in the presence of heavy metal atom.

“Check Calcd Resid. Dens. 1.85Ang From C02L 4.24 eA-3”

This Alert is due to presence of residual density in the presence of heavy metal atom.

“Check Calcd Resid. Dens. 1.59Ang From F00N 4.22 eA-3”

This Alert is due to presence of residual density in the presence of heavy metal atom.

“Check Calcd Resid. Dens. 1.77Ang From F00V 4.12 eA-3”

This Alert is due to presence of residual density in the presence of heavy metal atom.

“Check Calcd Resid. Dens. 1.09Ang From Ag03 4.06 eA-3”

This Alert is due to presence of residual density in the presence of heavy metal atom.

"Check Calcd Resid. Dens. 1.93Ang From F00Z 3.81 eA-3"

This Alert is due to presence of residual density in the presence of heavy metal atom.

"Check Calcd Resid. Dens. 1.04Ang From N017 3.80 eA-3"

This Alert is due to presence of residual density in the presence of heavy metal atom.

"Check Calcd Resid. Dens. 1.39Ang From F00I 3.77 eA-3"

This Alert is due to presence of residual density in the presence of heavy metal atom.

"Check Calcd Resid. Dens. 1.09Ang From Ag06 3.75 eA-3"

This Alert is due to presence of residual density in the presence of heavy metal atom.

"Check Calcd Resid. Dens. 1.57Ang From F00Z 3.56 eA-3"

This Alert is due to presence of residual density in the presence of heavy metal atom.

"Check Calcd Resid. Dens. 1.23Ang From C01P 3.51 eA-3"

This Alert is due to presence of residual density in the presence of heavy metal atom.

B-level alerts:

"Large Reported Max. (Positive) Residual Density 5.32 eA-3"

This Alert is due to presence of residual density in the presence of heavy metal atom.

"Low Bond Precision on C-C Bonds .... 0.02472 Ang."

Disordered structure.

"Check Calcd Resid. Dens. 1.01Ang From Ag02 3.40 eA-3"

This Alert is due to presence of residual density in the presence of heavy metal atom.

"Check Calcd Resid. Dens. 1.37Ang From C01Q 3.28 eA-3"

This Alert is due to presence of residual density in the presence of heavy metal atom.

"Check Calcd Resid. Dens. 1.04Ang From Ag04 3.20 eA-3"

This Alert is due to presence of residual density in the presence of heavy metal atom.

“Check Calcd Resid. Dens. 1.15Ang From Ag05 3.08 eA-3”

This Alert is due to presence of residual density in the presence of heavy metal atom.

“Check Calcd Resid. Dens. 1.51Ang From F00R 2.81 eA-3”

This Alert is due to presence of residual density in the presence of heavy metal atom.

“Check Calcd Resid. Dens. 0.92Ang From Ag05 -3.07 eA-3”

This Alert is due to presence of residual density in the presence of heavy metal atom.

“Check Calcd Resid. Dens. 0.87Ang From Ag02 -2.73 eA-3”

This Alert is due to presence of residual density in the presence of heavy metal atom.

“Check Calcd Resid. Dens. 0.84Ang From Ag04 -2.61 eA-3”

This Alert is due to presence of residual density in the presence of heavy metal atom.

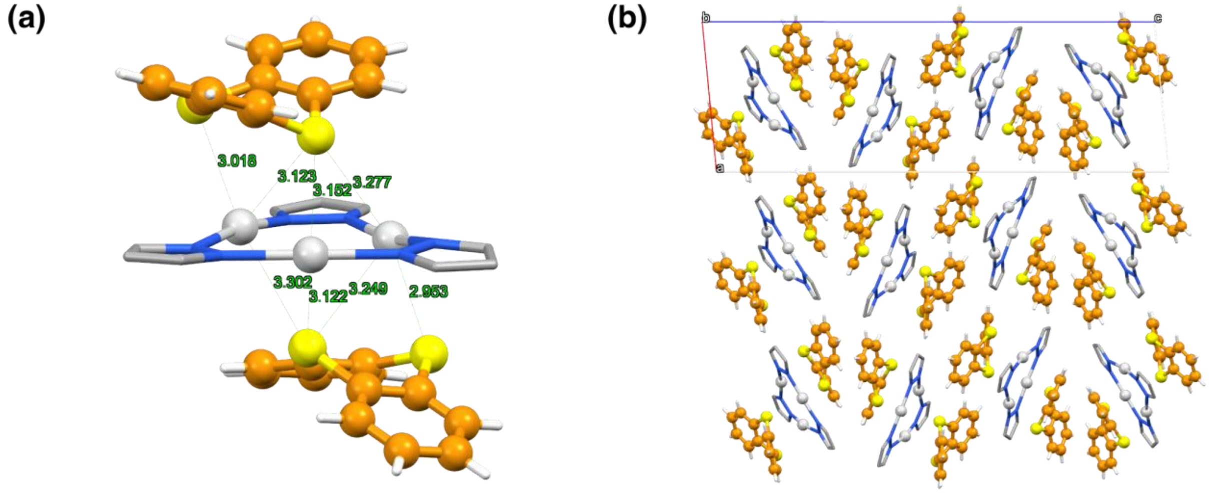

Figure S293. (a) A schematic diagram of the co-crystal structure in the $\mathbf{Ag_3Pz_3{\cdot}92}$ single crystal, formed by the guest organic molecule and the surrounding Ag_3Pz_3 units that exhibit significant interactions with it. (b) A 3 × 1 × 1 packing mode in the single crystal structure of $\mathbf{Ag_3Pz_3{\cdot}92}$ along the *b* axis. Trifluoromethyl groups and H atoms in Ag_3Pz_3 are omitted for clarity. Ag···S interactions are indicated with green dotted lines with distances in Å. C, N, and Ag atoms in Ag_3Pz_3 are depicted in dark gray, light blue, and light gray, respectively; C, S, and H atoms in **92** are depicted in orange, yellow, and white, respectively.

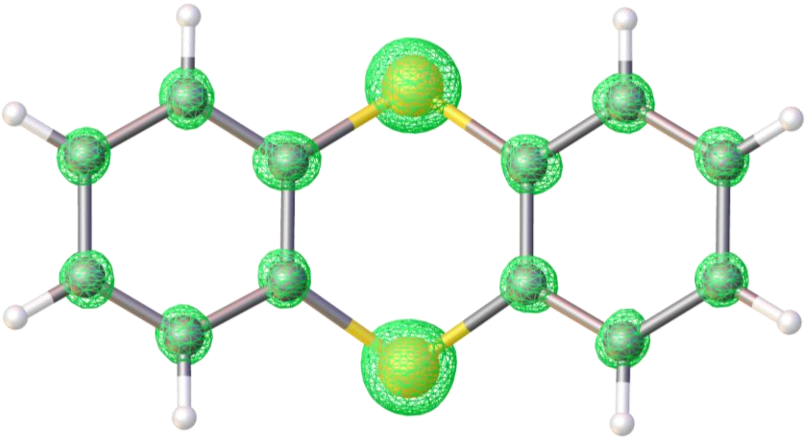

Figure S294. F_{obs} (contour: 0.60) electron density map superimposed on the structure of **92** in the single crystal structure of $\mathbf{Ag_3Pz_3{\cdot}92}$.

Preparation of Ag_3Pz_3·93. 2.10 mg (0.0107 mmol) of 2-(dimethyl (oxo)-Λ6-sulfanylidene)-1-phenylethan-1-one (**93**) was dissolved in 3 mL of n-Hex, followed by the addition of equimolar amounts of Ag_3Pz_3 (10.00 mg, 0.0107 mmol). The resulting mixed solution was filtered and then transferred to a 20 mL screw-capped sample vial. The cap of the sample vial was loosely closed to allow the solvent to slowly evaporate at room temperature. The entire co-crystal incubation process was protected from light using aluminum foil. After the designated evaporation period, typically 1-3 days, high-quality colorless needle-shaped crystals suitable for single-crystal X-ray diffraction analysis formed at the bottom of the vial.

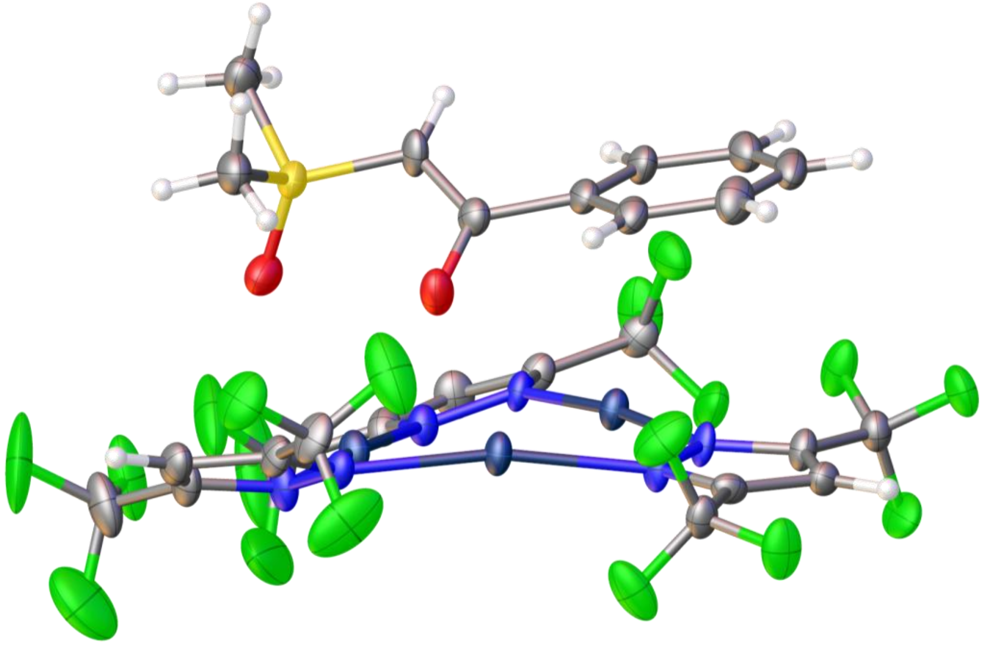

Figure S295. Asymmetric unit of **Ag_3Pz_3·93** (thermal displacement parameters at the 50% probability level).

Table S101. Crystal data and structure refinement for **Ag_3Pz_3·93**

Empirical formula	$C_{25}H_{15}Ag_3F_{18}N_6O_2S$
Formula weight	1129.10
Temperature/K	100.15
Crystal system	monoclinic
Space group	$P2_1/c$
a/Å	12.8384(2)
b/Å	20.2216(3)
c/Å	13.6185(3)
α/°	90
β/°	107.272(2)
γ/°	90
Volume/Å^3	3376.11(11)
Z	4
ρ_{calc}g/cm^3	2.221
μ/mm^{-1}	15.691
F(000)	2168.0
Crystal size/mm^3	0.26 × 0.15 × 0.15
Radiation	Cu Kα (λ = 1.54184)
2θ range for data collection/°	7.21 to 155.598
Index ranges	$-16 \leq h \leq 16$, $-21 \leq k \leq 25$, $-12 \leq l \leq 17$
Reflections collected	16302
Independent reflections	6838 [R_{int} = 0.0483, R_{sigma} = 0.0498]
Data/restraints/parameters	6838/24/498
Goodness-of-fit on F^2	1.081
Final R indexes [I>=2σ (I)]	R_1 = 0.0587, wR_2 = 0.1539
Final R indexes [all data]	R_1 = 0.0733, wR_2 = 0.1596
Largest diff. peak/hole / e Å^{-3}	1.44/-1.56
CCDC-number	2501882

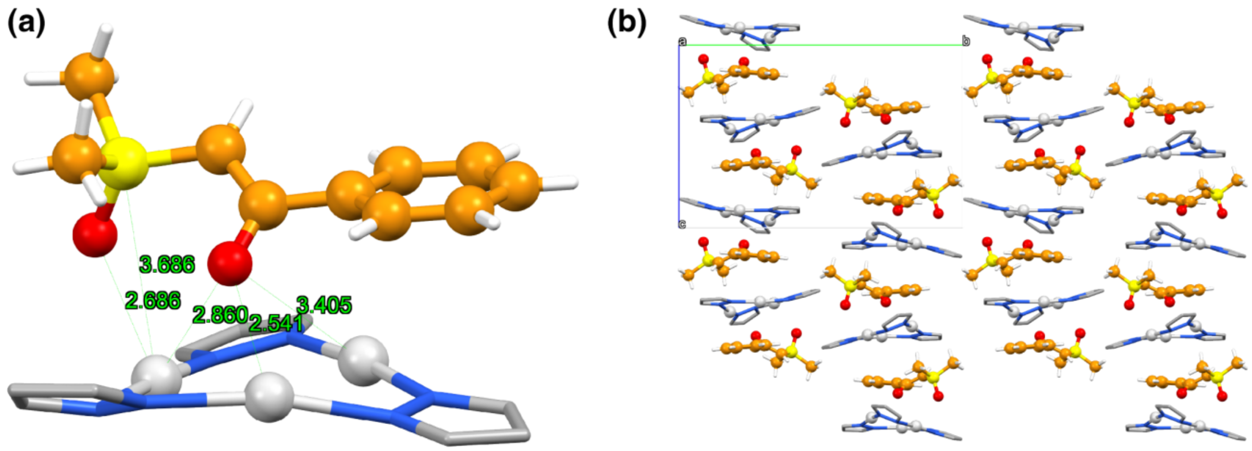

Figure S296. (a) A schematic diagram of the co-crystal structure in the $\mathbf{Ag_3Pz_3 \cdot 93}$ single crystal, formed by the guest organic molecule and the surrounding Ag_3Pz_3 units that exhibit significant interactions with it. (b) A 1 × 2 × 2 packing mode in the single crystal structure of $\mathbf{Ag_3Pz_3 \cdot 93}$ along the *a* axis. Trifluoromethyl groups and H atoms in Ag_3Pz_3 are omitted for clarity. Ag···S and Ag···O interactions are indicated with green dotted lines with distances in Å. C, N, and Ag atoms in Ag_3Pz_3 are depicted in dark gray, light blue, and light gray, respectively; C, S, O, and H atoms in **93** are depicted in orange, yellow, red, and white, respectively.

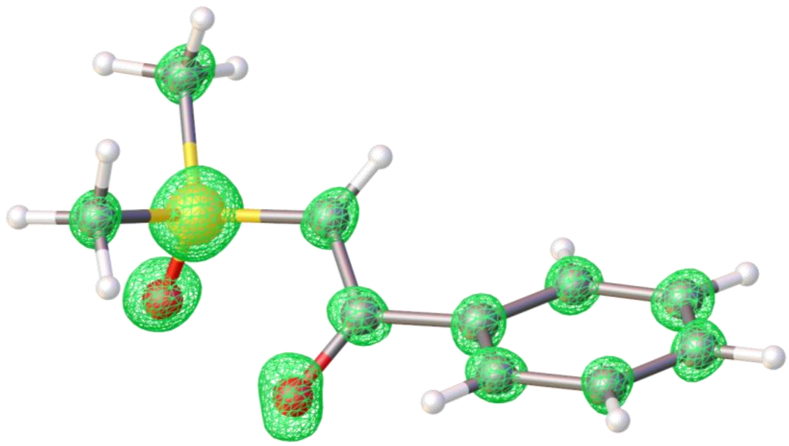

Figure S297. F_{obs} (contour: 0.85) electron density map superimposed on the structure of **93** in the single crystal structure of $\mathbf{Ag_3Pz_3 \cdot 93}$.

Preparation of Ag_3Pz_3·94. 2.98 mg (0.0107 mmol) of N-(4-(2,4-dihydroxyphenyl)-1,3-thiazol-2-yl)-2-methylpropanamide (**94**) was dissolved in 3 mL of a binary solvent system of Me_2CO and c-Hex (1:1, v/v), followed by the addition of equimolar amounts of Ag_3Pz_3 (10.00 mg, 0.0107 mmol). The resulting mixed solution was filtered and then transferred to a 20 mL screw-capped sample vial. The cap of the sample vial was loosely closed to allow the solvent to slowly evaporate at room temperature. The entire co-crystal incubation process was protected from light using aluminum foil. After the designated evaporation period, typically 1-3 days, high-quality colorless plate-shaped crystals suitable for single-crystal X-ray diffraction analysis formed at the bottom of the vial.

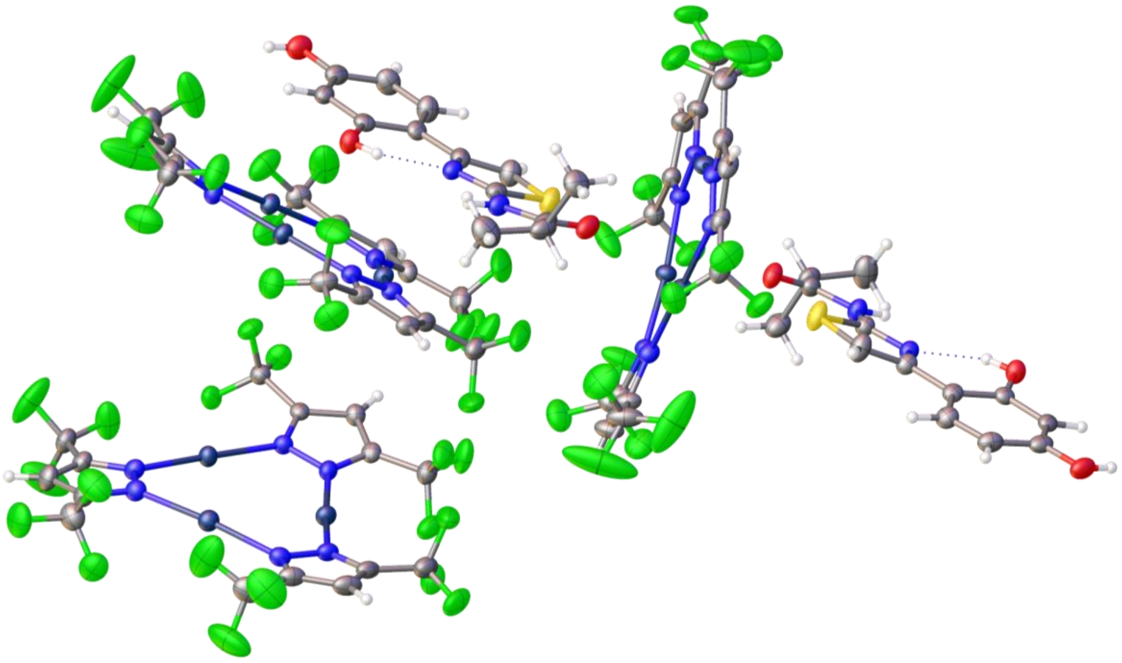

Figure S298. Asymmetric unit of **Ag_3Pz_3·94** (thermal displacement parameters at the 50% probability level).

Table S102. Crystal data and structure refinement for **Ag_3Pz_3·94**

Empirical formula	$C_{71}H_{37}Ag_9F_{54}N_{22}O_6S_2$
Formula weight	3355.17
Temperature/K	100.15
Crystal system	triclinic
Space group	$P\bar{1}$
a/Å	13.1122(2)
b/Å	17.9462(3)
c/Å	22.7626(3)
α/°	70.7840(10)
β/°	79.3120(10)
γ/°	75.974(2)
Volume/Å^3	4874.37(14)
Z	2
ρ_{calc}g/cm^3	2.286
μ/mm^{-1}	16.112
F(000)	3212.0
Crystal size/mm^3	0.25 × 0.24 × 0.13
Radiation	Cu Kα (λ = 1.54184)
2θ range for data collection/°	6.996 to 146.024
Index ranges	$-13 \le h \le 16$, $-22 \le k \le 21$, $-27 \le l \le 28$
Reflections collected	83741
Independent reflections	18309 [R_{int} = 0.0333, R_{sigma} = 0.0203]
Data/restraints/parameters	18309/6/1485
Goodness-of-fit on F^2	1.044
Final R indexes [I>=2σ (I)]	R_1 = 0.0346, wR_2 = 0.0907
Final R indexes [all data]	R_1 = 0.0369, wR_2 = 0.0923
Largest diff. peak/hole / e Å^{-3}	1.42/-1.78
CCDC-number	2501883

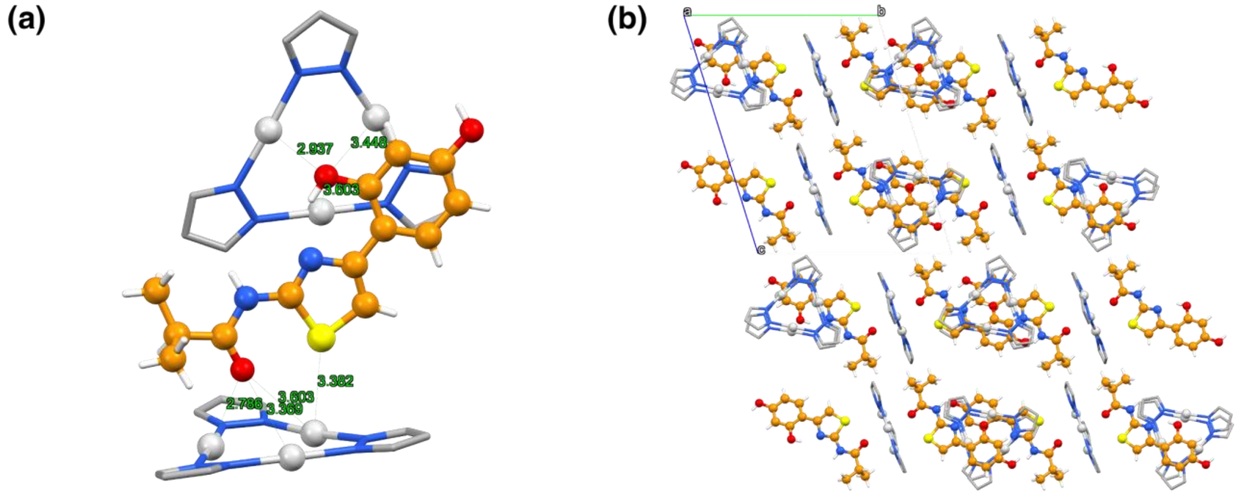

Figure S299. (a) A schematic diagram of the co-crystal structure in the **Ag_3Pz_3·94** single crystal, formed by the guest organic molecule and the surrounding Ag_3Pz_3 units that exhibit significant interactions with it. (b) A 1 × 2 × 2 packing mode in the single crystal structure of **Ag_3Pz_3·94** along the *a* axis. Trifluoromethyl groups and H atoms in Ag_3Pz_3 are omitted for clarity. Ag···S and Ag···O interactions are indicated with green dotted lines with distances in Å. C, N, and Ag atoms in Ag_3Pz_3 are depicted in dark gray, light blue, and light gray, respectively; C, S, O, N, and H atoms in **94** are depicted in orange, yellow, red, light blue, and white, respectively.

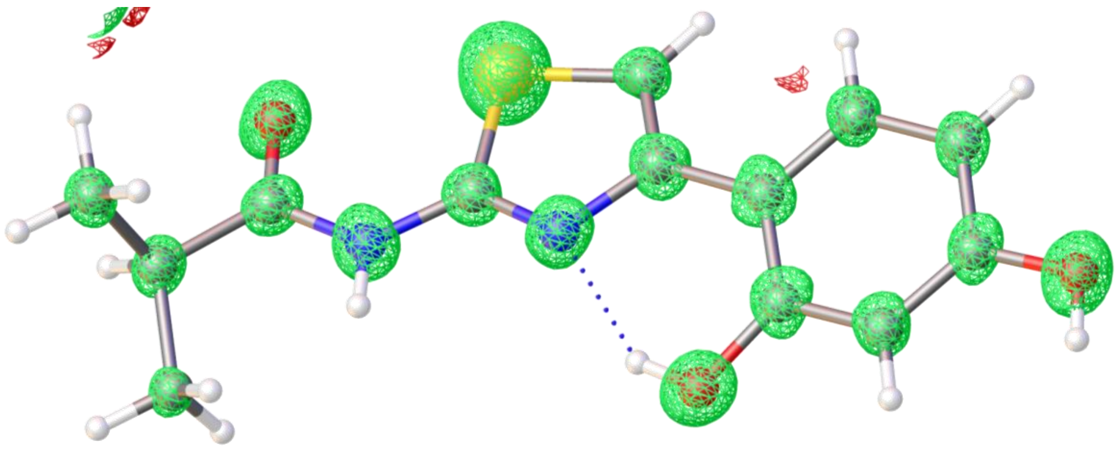

Figure S300. F_{obs} (contour: 0.70) electron density map superimposed on the structure of **94** in the single crystal structure of **Ag_3Pz_3·94**.

Preparation of Ag_3Pz_3·95. 2.23 mg (0.0107 mmol) of (E)-1,2-bis(4-methylphenyl)ethene (**95**) was dissolved in 3 mL of a binary solvent system of DCM and c-Hex (1:1, v/v), followed by the addition of equimolar amounts of Ag_3Pz_3 (10.00 mg, 0.0107 mmol). The resulting mixed solution was filtered and then transferred to a 20 mL screw-capped sample vial. The cap of the sample vial was loosely closed to allow the solvent to slowly evaporate at room temperature. The entire co-crystal incubation process was protected from light using aluminum foil. After the designated evaporation period, typically 1-3 days, high-quality colorless needle-shaped crystals suitable for single-crystal X-ray diffraction analysis formed at the bottom of the vial.

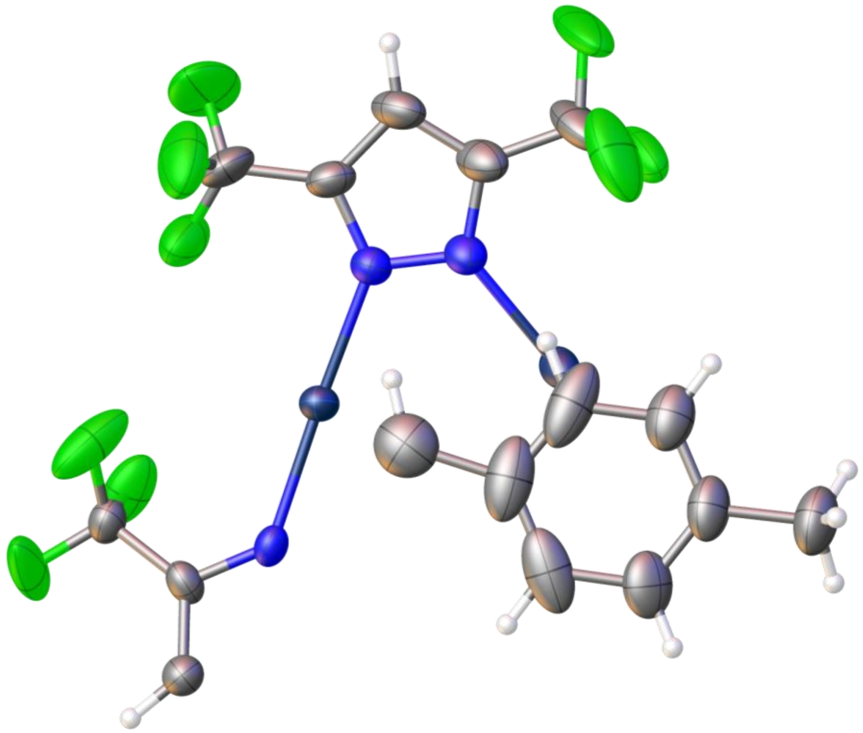

Figure S301. Asymmetric unit of **Ag_3Pz_3·95** (thermal displacement parameters at the 50% probability level).

Table S103. Crystal data and structure refinement for **Ag_3Pz_3·95**

Empirical formula	$C_{31}H_{19}Ag_3F_{18}N_6$
Formula weight	1141.13
Temperature/K	100.1(3)
Crystal system	monoclinic
Space group	$C2/c$
a/Å	23.0121(4)
b/Å	12.9825(2)
c/Å	12.1703(2)
α/°	90
β/°	94.3800(10)
γ/°	90
Volume/Å^3	3625.31(10)
Z	4
ρ_{calc}g/cm^3	2.091
μ/mm^{-1}	14.057
F(000)	2200.0
Crystal size/mm^3	0.19 × 0.16 × 0.14
Radiation	Cu Kα (λ = 1.54184)
2θ range for data collection/°	7.706 to 156.728
Index ranges	$-29 \le h \le 29$, $-11 \le k \le 16$, $-11 \le l \le 15$
Reflections collected	10735
Independent reflections	3742 [R_{int} = 0.0358, R_{sigma} = 0.0392]
Data/restraints/parameters	3742/1/264
Goodness-of-fit on F^2	1.108
Final R indexes [I>=2σ (I)]	R_1 = 0.0550, wR_2 = 0.1643
Final R indexes [all data]	R_1 = 0.0590, wR_2 = 0.1680
Largest diff. peak/hole / e Å^{-3}	2.07/-1.28
CCDC-number	2501884

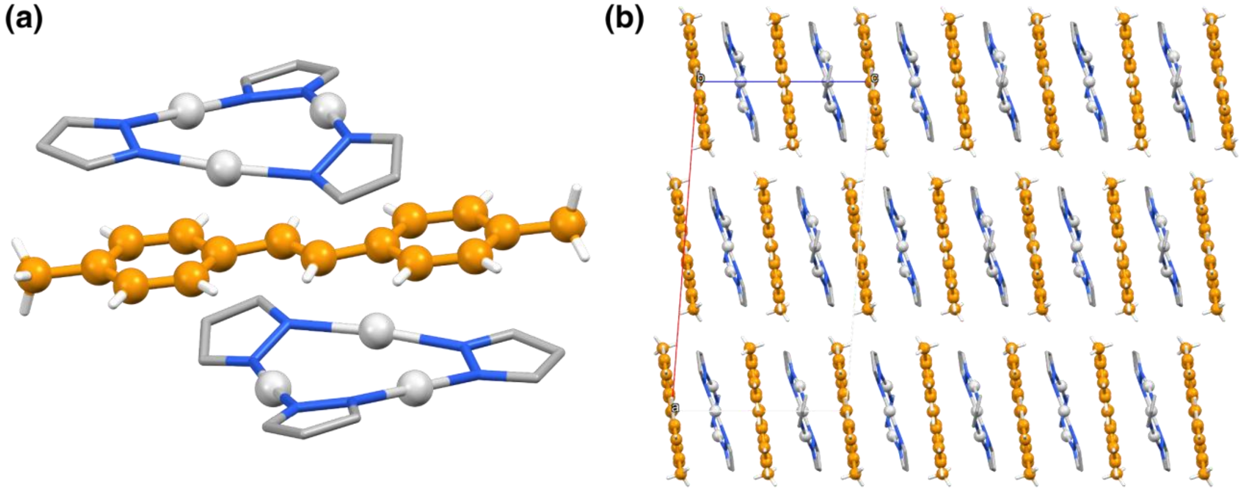

Figure S302. (a) A schematic diagram of the co-crystal structure in the **Ag_3Pz_3·95** single crystal, formed by the guest organic molecule and the surrounding Ag_3Pz_3 units that exhibit significant interactions with it. (b) A 1 × 1 × 3 packing mode in the single crystal structure of **Ag_3Pz_3·95** along the *b* axis. Trifluoromethyl groups and H atoms in Ag_3Pz_3 are omitted for clarity. C, N, and Ag atoms in Ag_3Pz_3 are depicted in dark gray, light blue, and light gray, respectively; C and H atoms in **95** are depicted in orange and white, respectively.

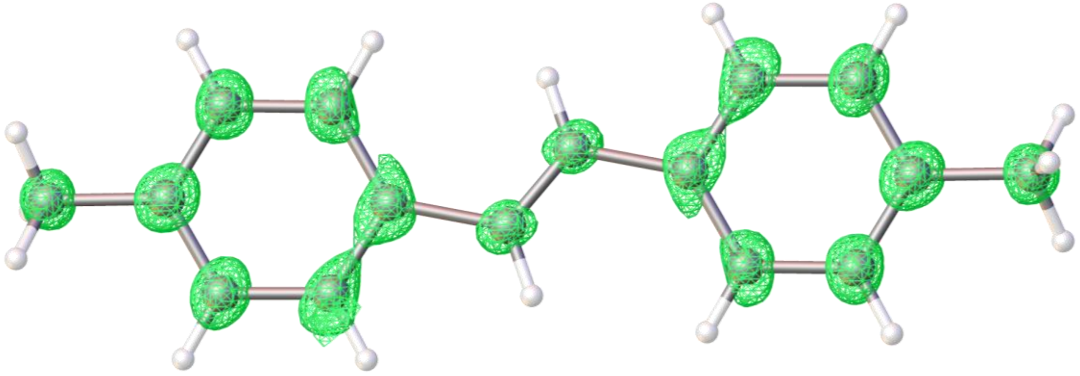

Figure S303. F_{obs} (contour: 0.35) electron density map superimposed on the structure of **95** in the single crystal structure of **Ag_3Pz_3·95**.

Preparation of Ag_3Pz_3·96. 1.93 mg (0.0107 mmol) of trans-1,2-diphenylethylene (**96**) was dissolved in 3 mL of a binary solvent system of DCM and MeOH (1:1, v/v), followed by the addition of equimolar amounts of Ag_3Pz_3 (10.00 mg, 0.0107 mmol). The resulting mixed solution was filtered and then transferred to a 20 mL screw-capped sample vial. The cap of the sample vial was loosely closed to allow the solvent to slowly evaporate at room temperature. The entire co-crystal incubation process was protected from light using aluminum foil. After the designated evaporation period, typically 1-3 days, high-quality colorless needle-shaped crystals suitable for single-crystal X-ray diffraction analysis formed at the bottom of the vial.

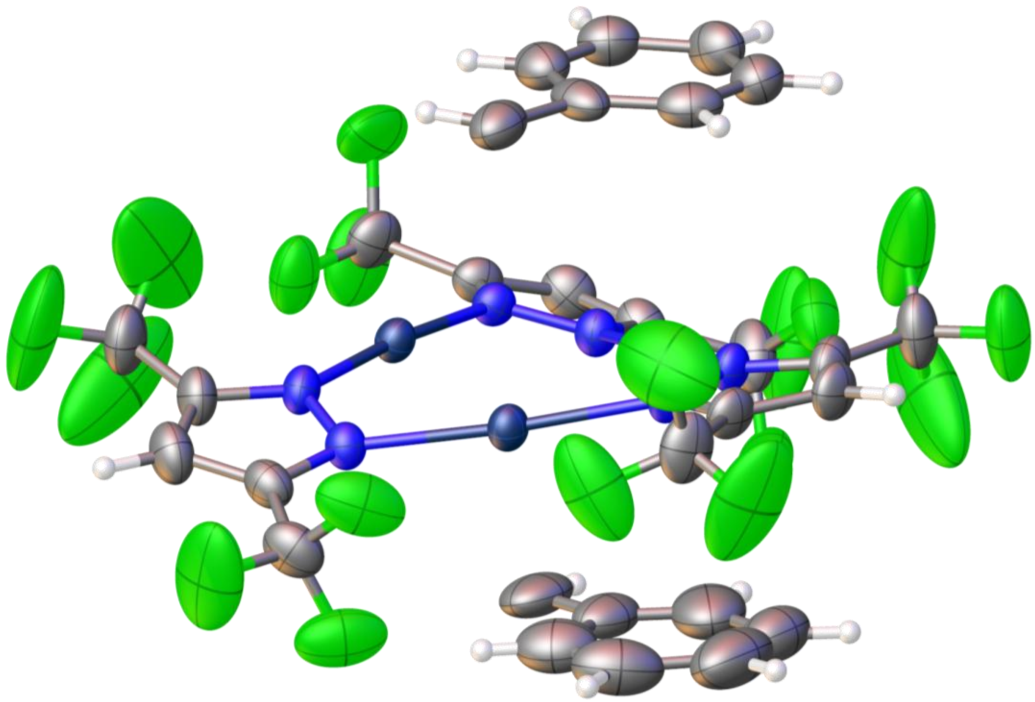

Figure S304. Asymmetric unit of **Ag_3Pz_3·96** (thermal displacement parameters at the 50% probability level).

Table S104. Crystal data and structure refinement for **Ag_3Pz_3·96**

Empirical formula	$C_{58}H_{30}Ag_6F_{36}N_{12}$
Formula weight	2226.16
Temperature/K	150.00(11)
Crystal system	triclinic
Space group	$P\bar{1}$
a/Å	12.4747(3)
b/Å	12.7374(3)
c/Å	12.9647(4)
α/°	118.671(3)
β/°	103.031(2)
γ/°	92.084(2)
Volume/Å^3	1735.54(9)
Z	1
ρ_{calc}g/cm^3	2.130
μ/mm^{-1}	14.660
F(000)	1068.0
Crystal size/mm^3	0.1 × 0.1 × 0.1
Radiation	Cu Kα (λ = 1.54184)
2θ range for data collection/°	7.38 to 155.748
Index ranges	$-15 \leq h \leq 14$, $-16 \leq k \leq 16$, $-16 \leq l \leq 16$
Reflections collected	16318
Independent reflections	7037 [R_{int} = 0.0236, R_{sigma} = 0.0254]
Data/restraints/parameters	7037/24/505
Goodness-of-fit on F^2	1.067
Final R indexes [I>=2σ (I)]	R_1 = 0.0527, wR_2 = 0.1454
Final R indexes [all data]	R_1 = 0.0552, wR_2 = 0.1480
Largest diff. peak/hole / e Å^{-3}	1.76/-1.18
CCDC-number	2501885

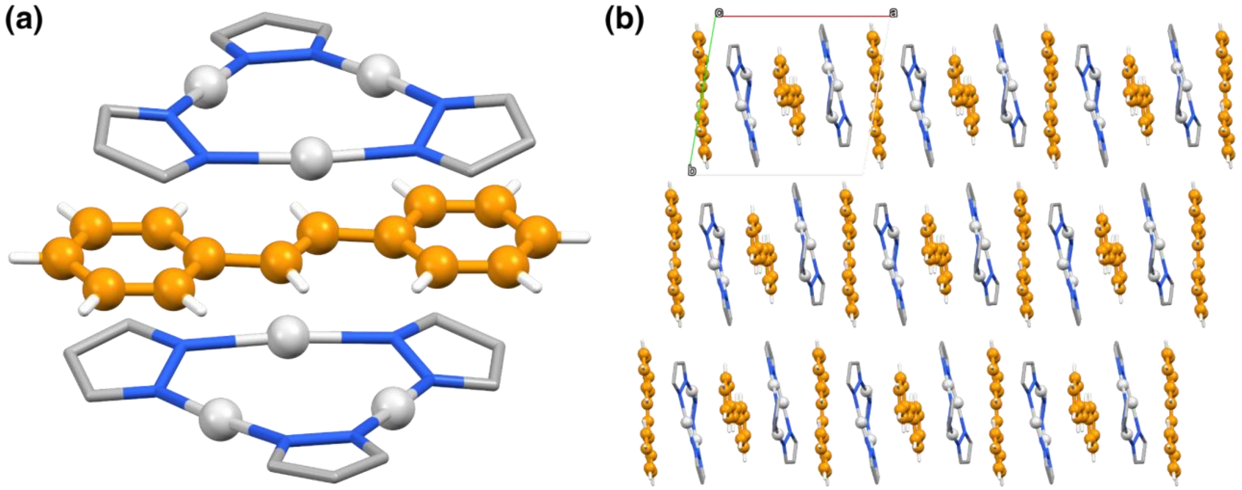

Figure S305. (a) A schematic diagram of the co-crystal structure in the **Ag_3Pz_3·96** single crystal, formed by the guest organic molecule and the surrounding Ag_3Pz_3 units that exhibit significant interactions with it. (b) A 3 × 3 × 1 packing mode in the single crystal structure of **Ag_3Pz_3·96** along the *c* axis. Trifluoromethyl groups and H atoms in Ag_3Pz_3 are omitted for clarity. C, N, and Ag atoms in Ag_3Pz_3 are depicted in dark gray, light blue, and light gray, respectively; C and H atoms in **96** are depicted in orange and white, respectively.

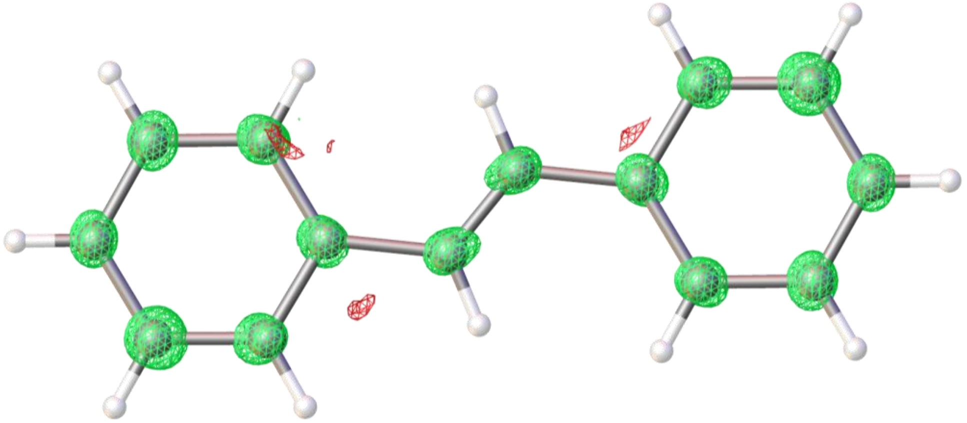

Figure S306. F_{obs} (contour: 1.45) electron density map superimposed on the structure of **96** in the single crystal structure of **Ag_3Pz_3·96**.

Preparation of Ag_3Pz_3·97. 1.65 mg (0.0107 mmol) of acenaphthene (**97**) was dissolved in 3 mL of a binary solvent system of DCM and MeOH (1:1, v/v), followed by the addition of equimolar amounts of Ag_3Pz_3 (10.00 mg, 0.0107 mmol). The resulting mixed solution was filtered and then transferred to a 20 mL screw-capped sample vial. The cap of the sample vial was loosely closed to allow the solvent to slowly evaporate at room temperature. The entire co-crystal incubation process was protected from light using aluminum foil. After the designated evaporation period, typically 1-3 days, high-quality colorless block-shaped crystals suitable for single-crystal X-ray diffraction analysis formed at the bottom of the vial.

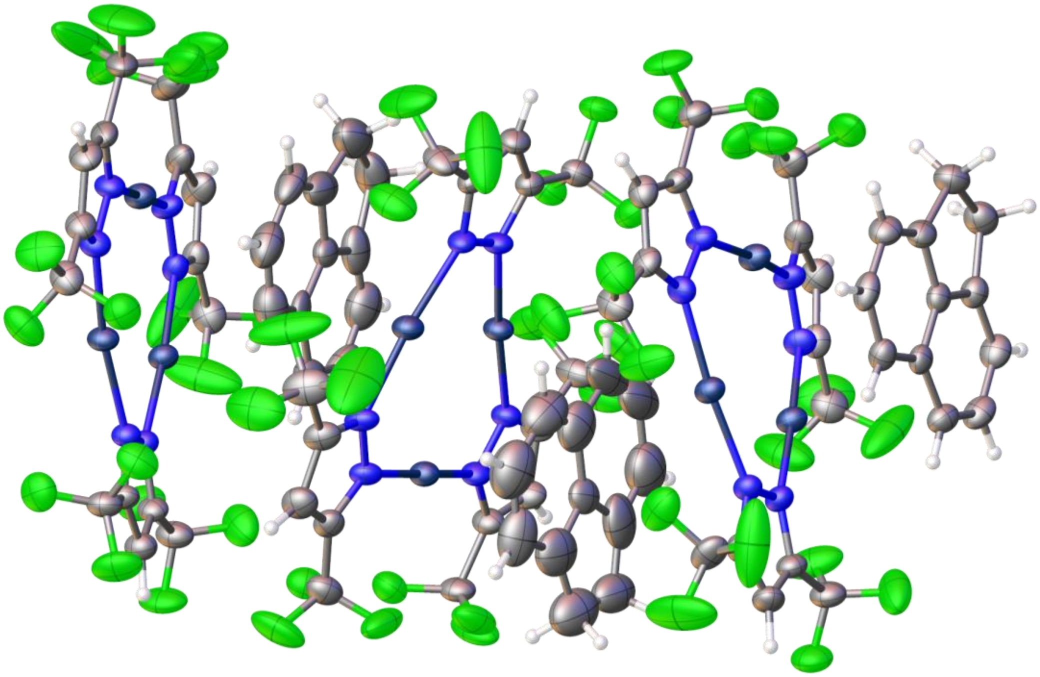

Figure S307. Asymmetric unit of **Ag_3Pz_3·97** (thermal displacement parameters at the 50% probability level).

Table S105. Crystal data and structure refinement for **Ag_3Pz_3·97**

Empirical formula	$C_{27}H_{13}Ag_3F_{18}N_6$
Formula weight	1087.04
Temperature/K	100.0(2)
Crystal system	monoclinic
Space group	$P2_1/n$
a/Å	22.5622(2)
b/Å	12.88500(10)
c/Å	33.9620(3)
α/°	90
β/°	103.2330(10)
γ/°	90
Volume/Å^3	9611.06(15)
Z	12
ρ_{calc}g/cm^3	2.254
μ/mm^{-1}	15.861
F(000)	6240.0
Crystal size/mm^3	0.2 × 0.2 × 0.2
Radiation	Cu Kα (λ = 1.54184)
2θ range for data collection/°	5.316 to 156.56
Index ranges	$-28 \leq h \leq 27$, $-14 \leq k \leq 15$, $-41 \leq l \leq 32$
Reflections collected	54453
Independent reflections	19755 [R_{int} = 0.0438, R_{sigma} = 0.0470]
Data/restraints/parameters	19755/32/1450
Goodness-of-fit on F^2	1.029
Final R indexes [I>=2σ (I)]	R_1 = 0.0589, wR_2 = 0.1537
Final R indexes [all data]	R_1 = 0.0701, wR_2 = 0.1611
Largest diff. peak/hole / e Å^{-3}	1.35/-1.20
CCDC-number	2501886

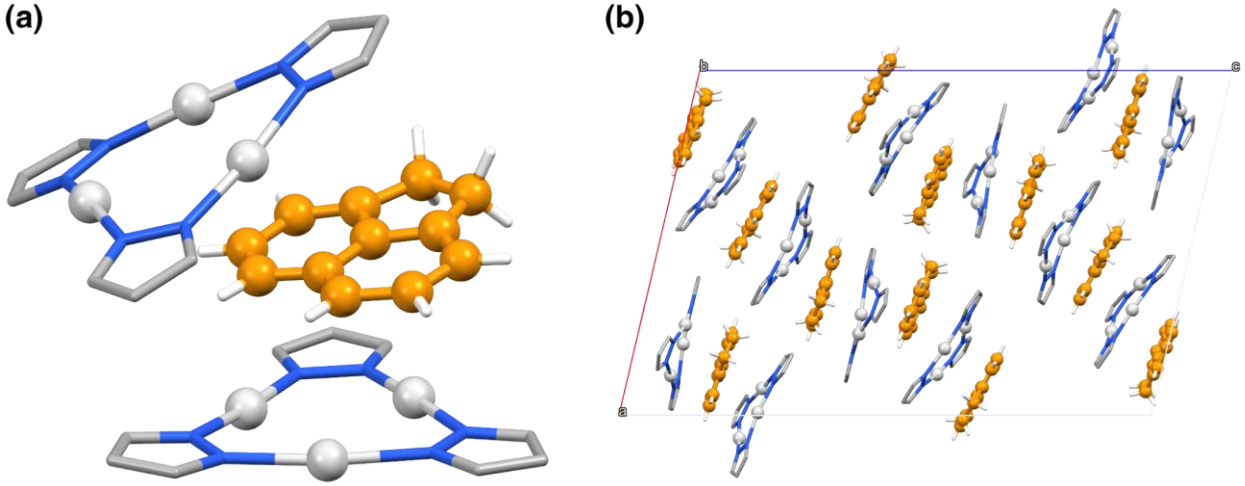

Figure S308. (a) A schematic diagram of the co-crystal structure in the **Ag_3Pz_3·97** single crystal, formed by the guest organic molecule and the surrounding Ag_3Pz_3 units that exhibit significant interactions with it. (b) A 1 × 1 × 1 packing mode in the single crystal structure of **Ag_3Pz_3·97** along the *b* axis. Trifluoromethyl groups and H atoms in Ag_3Pz_3 are omitted for clarity. C, N, and Ag atoms in Ag_3Pz_3 are depicted in dark gray, light blue, and light gray, respectively; C and H atoms in **97** are depicted in orange and white, respectively.

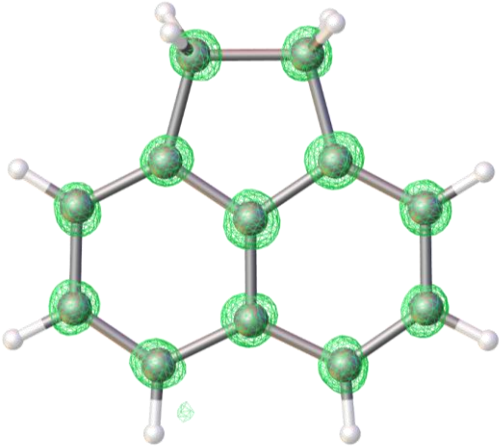

Figure S309. F_{obs} (contour: 0.25) electron density map superimposed on the structure of **97** in the single crystal structure of **Ag_3Pz_3·97**.

Preparation of Ag_3Pz_3·98. 1.63 mg (0.0107 mmol) of acenaphthylene (**98**) was dissolved in 3 mL of DCM, followed by the addition of equimolar amounts of Ag_3Pz_3 (10.00 mg, 0.0107 mmol). The resulting mixed solution was filtered and then transferred to a 20 mL screw-capped sample vial. The cap of the sample vial was loosely closed to allow the solvent to slowly evaporate at room temperature. The entire co-crystal incubation process was protected from light using aluminum foil. After the designated evaporation period, typically 1-3 days, high-quality yellow needle-shaped crystals suitable for single-crystal X-ray diffraction analysis formed at the bottom of the vial.

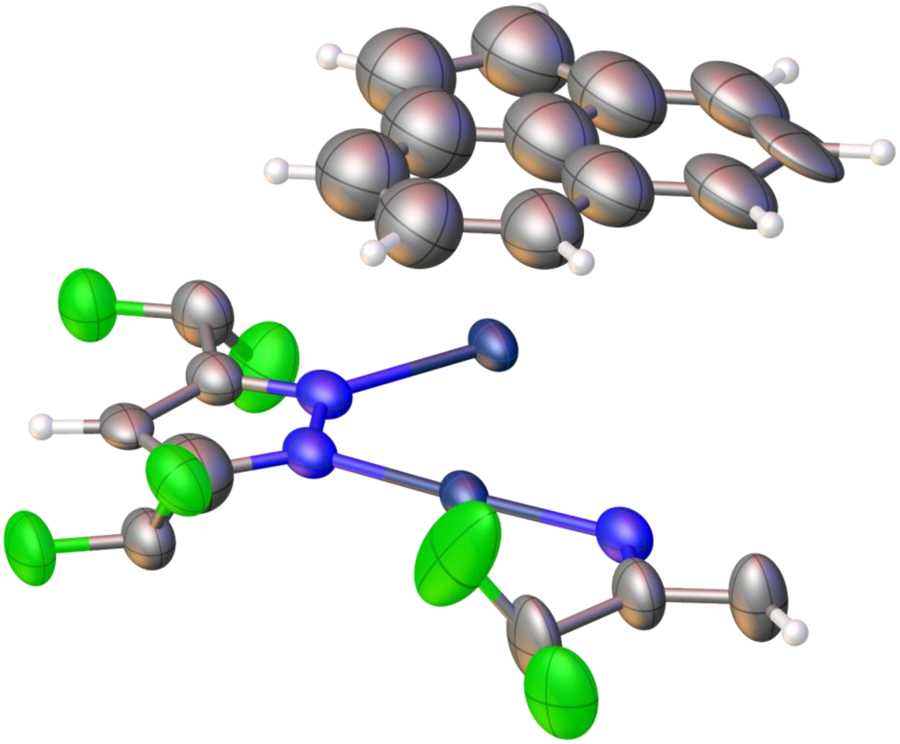

Figure S310. Asymmetric unit of **Ag_3Pz_3·98** (thermal displacement parameters at the 50% probability level).

Table S106. Crystal data and structure refinement for **Ag_3Pz_3·98**

Empirical formula	$C_{38}H_{18}Ag_3F_{18}N_6$
Formula weight	1224.19
Temperature/K	100.15
Crystal system	orthorhombic
Space group	*Cmcm*
a/Å	6.5206(2)
b/Å	23.6115(5)
c/Å	21.1404(5)
α/°	90
β/°	90
γ/°	90
Volume/Å^3	3254.80(14)
Z	4
ρ_{calc}g/cm^3	2.498
μ/mm^{-1}	15.734
F(000)	2364.0
Crystal size/mm^3	0.26 × 0.16 × 0.14
Radiation	Cu Kα (λ = 1.54184)
2θ range for data collection/°	7.488 to 156.02
Index ranges	$-7 \leq h \leq 5$, $-29 \leq k \leq 28$, $-26 \leq l \leq 21$
Reflections collected	5418
Independent reflections	1842 [R_{int} = 0.0484, R_{sigma} = 0.0407]
Data/restraints/parameters	1842/301/199
Goodness-of-fit on F^2	1.257
Final R indexes [I>=2σ (I)]	R_1 = 0.0914, wR_2 = 0.2624
Final R indexes [all data]	R_1 = 0.0977, wR_2 = 0.2725
Largest diff. peak/hole / e Å^{-3}	2.39/-2.17
CCDC-number	2501887

Responses to CheckCIF alert for Ag_3Pz_3·98 crystal structure:

(There is no A-level alert)

B-level alert:

“Low Bond Precision on C-C Bonds ....   0.02117 Ang.”

Disordered structure.

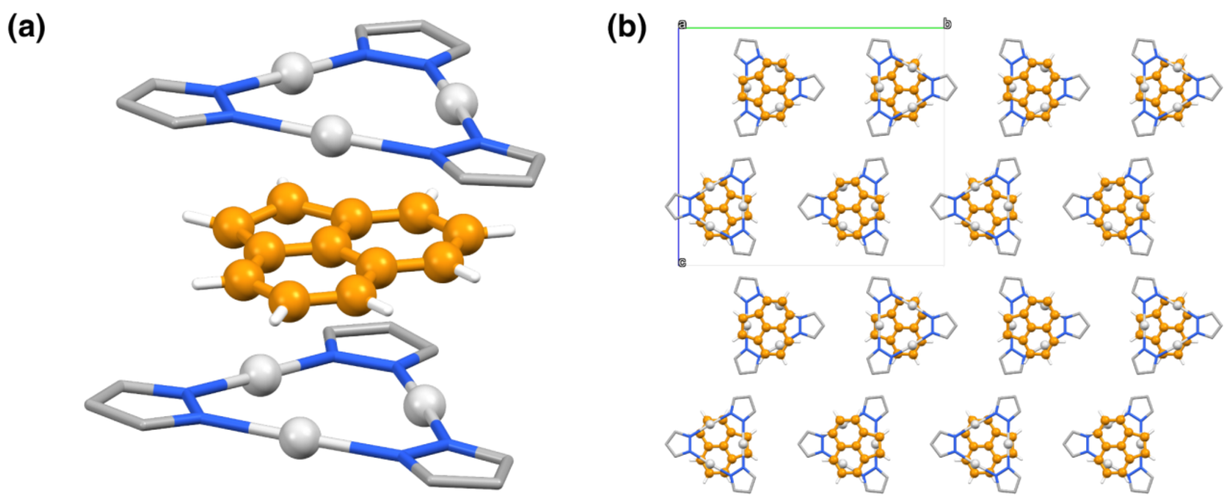

Figure S311. (a) A schematic diagram of the co-crystal structure in the **Ag_3Pz_3·98** single crystal, formed by the guest organic molecule and the surrounding Ag_3Pz_3 units that exhibit significant interactions with it. (b) A 1 × 2 × 2 packing mode in the single crystal structure of **Ag_3Pz_3·98** along the *a* axis. Trifluoromethyl groups and H atoms in Ag_3Pz_3 are omitted for clarity. C, N, and Ag atoms in Ag_3Pz_3 are depicted in dark gray, light blue, and light gray, respectively; C and H atoms in **98** are depicted in orange and white, respectively.

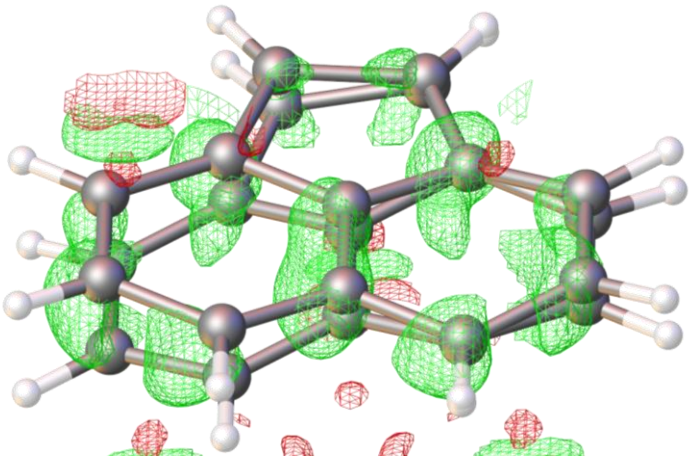

Figure S312. F_{obs} (contour: 0.35) electron density map superimposed on the structure of **98** in the single crystal structure of **Ag_3Pz_3·98**. Please note that there is a twofold disorder in the molecule **98** which is due to vibrations of the molecular plane. We believe that the unassigned electron density is attributable to residual solvent molecules and the Ag_3Pz_3 units.

Preparation of Ag_3Pz_3·99. 3.51 mg (0.0107 mmol) of dibenzo[g,p]chrysene (**99**) was dissolved in 3 mL of a binary solvent system of DCM and n-Hex (1:1, v/v), followed by the addition of equimolar amounts of Ag_3Pz_3 (10.00 mg, 0.0107 mmol). The resulting mixed solution was filtered and then transferred to a 20 mL screw-capped sample vial. The cap of the sample vial was loosely closed to allow the solvent to slowly evaporate at room temperature. The entire co-crystal incubation process was protected from light using aluminum foil. After the designated evaporation period, typically 1-3 days, high-quality colorless needle-shaped crystals suitable for single-crystal X-ray diffraction analysis formed at the bottom of the vial.

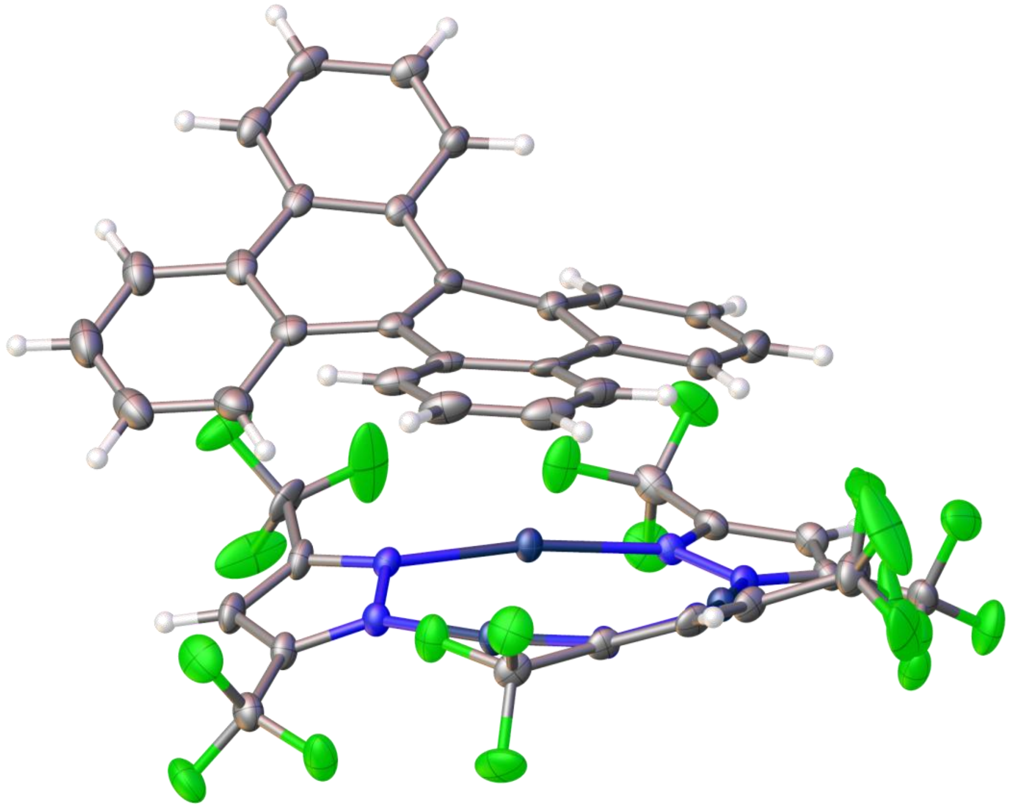

Figure S313. Asymmetric unit of **Ag_3Pz_3·99** (thermal displacement parameters at the 50% probability level).

Table S107. Crystal data and structure refinement for **Ag_3Pz_3·99**

Empirical formula	$C_{41}H_{19}Ag_3F_{18}N_6$
Formula weight	1261.23
Temperature/K	100.15
Crystal system	orthorhombic
Space group	*Pbca*
a/Å	12.60020(10)
b/Å	14.7573(2)
c/Å	42.4666(4)
α/°	90
β/°	90
γ/°	90
Volume/Å^3	7896.45(14)
Z	8
ρ_{calc}g/cm^3	2.122
μ/mm^{-1}	12.998
F(000)	4880.0
Crystal size/mm^3	0.2 × 0.2 × 0.1
Radiation	Cu Kα (λ = 1.54184)
2θ range for data collection/°	8.16 to 157.06
Index ranges	$-8 \le h \le 15$, $-18 \le k \le 15$, $-51 \le l \le 53$
Reflections collected	32106
Independent reflections	8244 [R_{int} = 0.0812, R_{sigma} = 0.0596]
Data/restraints/parameters	8244/69/641
Goodness-of-fit on F^2	1.050
Final R indexes [I>=2σ (I)]	R_1 = 0.0651, wR_2 = 0.1592
Final R indexes [all data]	R_1 = 0.0720, wR_2 = 0.1636
Largest diff. peak/hole / e Å^{-3}	1.33/-2.14
CCDC-number	2501888

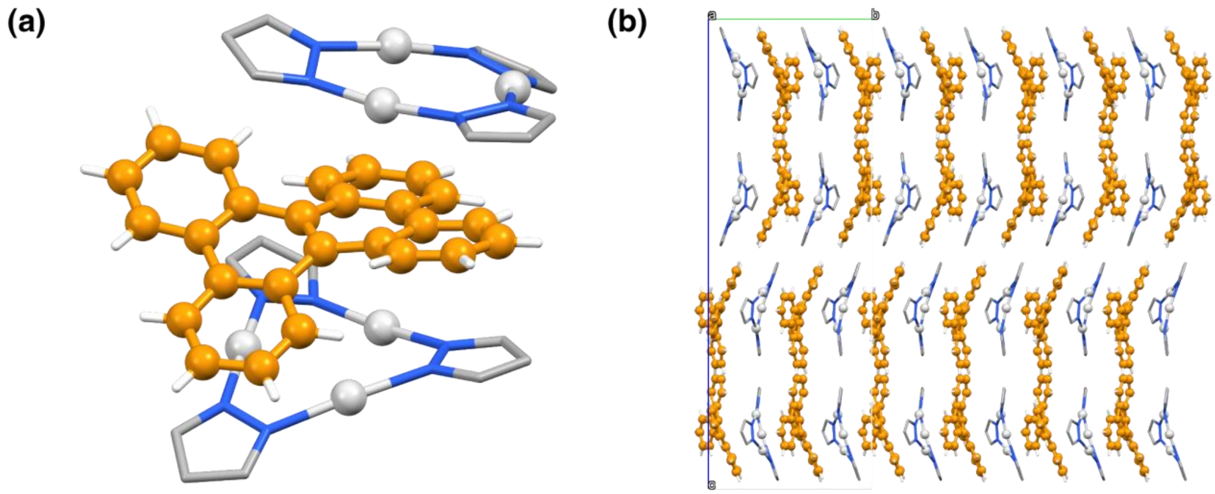

Figure S314. (a) A schematic diagram of the co-crystal structure in the $\mathbf{Ag_3Pz_3{\cdot}99}$ single crystal, formed by the guest organic molecule and the surrounding Ag_3Pz_3 units that exhibit significant interactions with it. (b) A 1 × 3 × 1 packing mode in the single crystal structure of $\mathbf{Ag_3Pz_3{\cdot}99}$ along the *a* axis. Trifluoromethyl groups and H atoms in Ag_3Pz_3 are omitted for clarity. C, N, and Ag atoms in Ag_3Pz_3 are depicted in dark gray, light blue, and light gray, respectively; C and H atoms in **99** are depicted in orange and white, respectively.

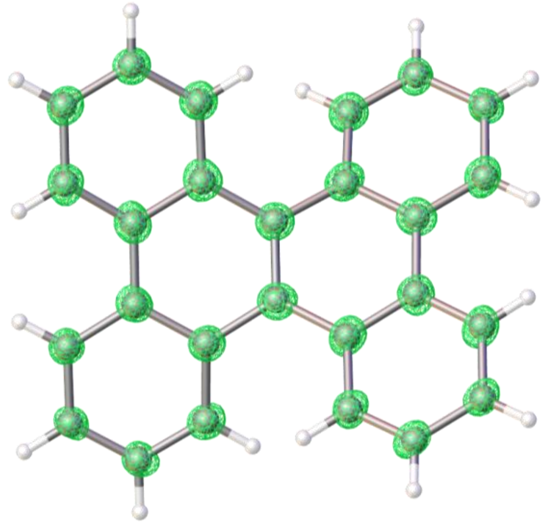

Figure S315. F_{obs} (contour: 0.60) electron density map superimposed on the structure of **99** in the single crystal structure of $\mathbf{Ag_3Pz_3{\cdot}99}$.

Preparation of Ag_3Pz_3·100. 1.65 mg (0.0107 mmol) of biphenyl (**100**) was dissolved in 3 mL of a binary solvent system of DCM and c-Hex (1:1, v/v), followed by the addition of equimolar amounts of Ag_3Pz_3 (10.00 mg, 0.0107 mmol). The resulting mixed solution was filtered and then transferred to a 20 mL screw-capped sample vial. The cap of the sample vial was loosely closed to allow the solvent to slowly evaporate at room temperature. The entire co-crystal incubation process was protected from light using aluminum foil. After the designated evaporation period, typically 1-3 days, high-quality colorless needle-shaped crystals suitable for single-crystal X-ray diffraction analysis formed at the bottom of the vial.

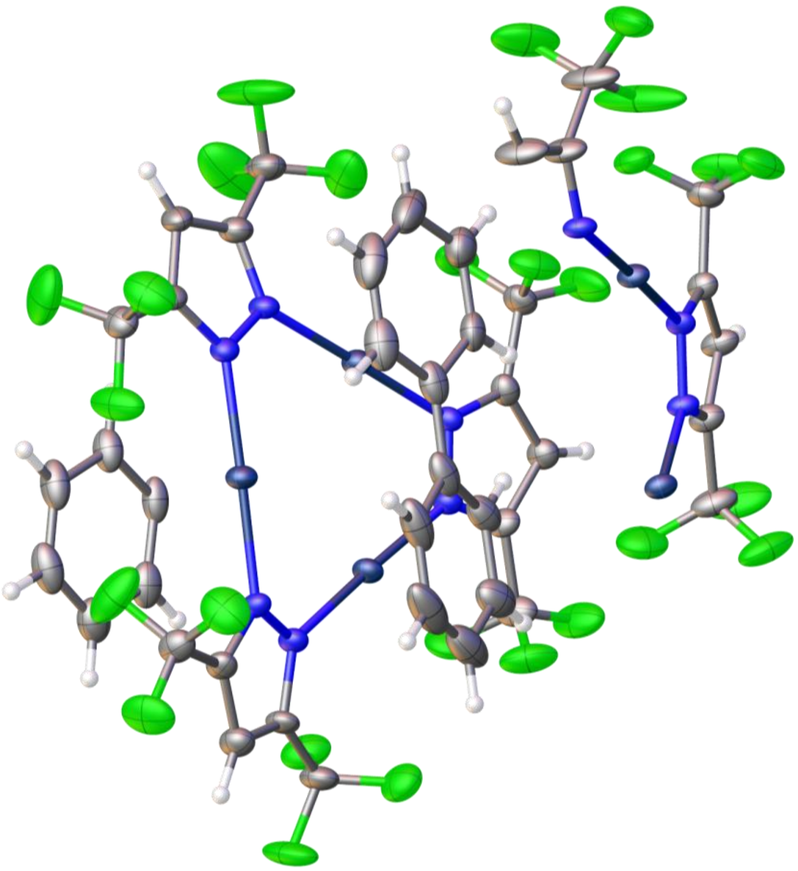

Figure S316. Asymmetric unit of **Ag_3Pz_3·100** (thermal displacement parameters at the 50% probability level).

Table S108. Crystal data and structure refinement for **Ag_3Pz_3·100**

Empirical formula	$C_{27}H_{13}Ag_3F_{18}N_6$
Formula weight	1087.04
Temperature/K	99.98(14)
Crystal system	monoclinic
Space group	*C*2/*c*
a/Å	27.1484(2)
b/Å	12.86880(10)
c/Å	28.3804(2)
α/°	90
β/°	97.3330(10)
γ/°	90
Volume/Å^3	9834.09(13)
Z	12
ρ_{calc}g/cm^3	2.203
μ/mm^{-1}	15.502
F(000)	6240.0
Crystal size/mm^3	0.2 × 0.2 × 0.2
Radiation	Cu K*α* (*λ* = 1.54184)
2*θ* range for data collection/°	6.566 to 155.846
Index ranges	$-34 \leq h \leq 32$, $-8 \leq k \leq 15$, $-34 \leq l \leq 35$
Reflections collected	30435
Independent reflections	10111 [R_{int} = 0.0293, R_{sigma} = 0.0264]
Data/restraints/parameters	10111/0/731
Goodness-of-fit on F^2	1.081
Final *R* indexes [I>=2σ (I)]	R_1 = 0.0382, wR_2 = 0.1041
Final *R* indexes [all data]	R_1 = 0.0399, wR_2 = 0.1054
Largest diff. peak/hole / e Å^{-3}	1.32/-0.66
CCDC-number	2501843

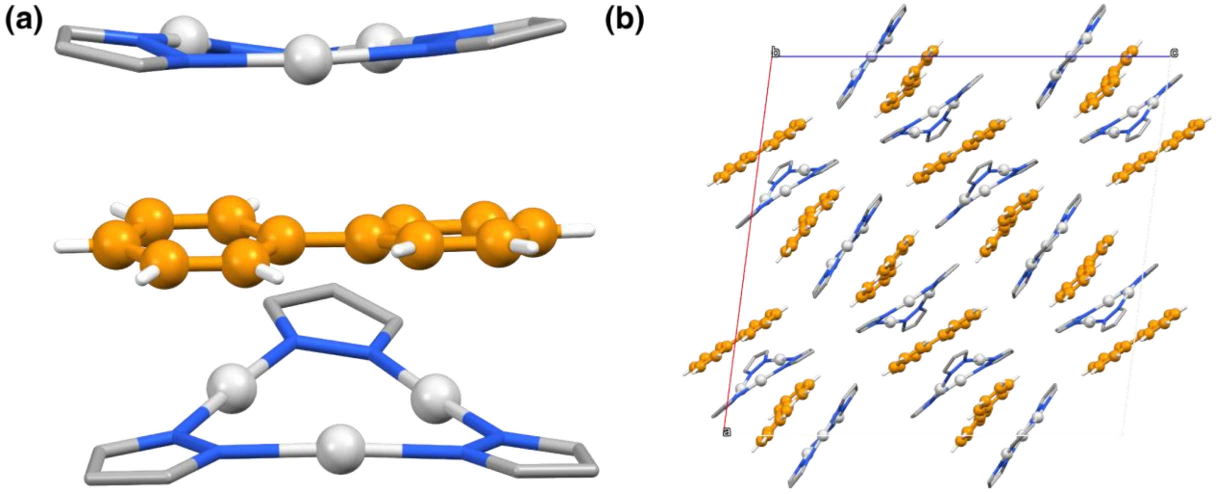

Figure S317. (a) A schematic diagram of the co-crystal structure in the **Ag_3Pz_3·100** single crystal, formed by the guest organic molecule and the surrounding Ag_3Pz_3 units that exhibit significant interactions with it. (b) A 1 × 1 × 1 packing mode in the single crystal structure of **Ag_3Pz_3·100** along the *b* axis. Trifluoromethyl groups and H atoms in Ag_3Pz_3 are omitted for clarity. C, N, and Ag atoms in Ag_3Pz_3 are depicted in dark gray, light blue, and light gray, respectively; C and H atoms in **100** are depicted in orange and white, respectively.

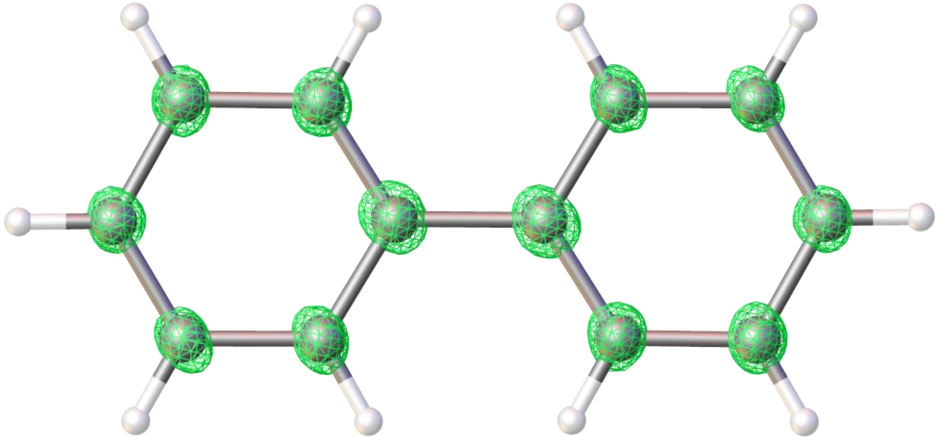

Figure S318. F_{obs} (contour: 0.45) electron density map superimposed on the structure of **100** in the single crystal structure of **Ag_3Pz_3·100**.

Preparation of Ag_3Pz_3·101. 1.80 mg (0.0107 mmol) of 4-methylbiphenyl (**101**) was dissolved in 3 mL of a binary solvent system of DCM and n-Hex (1:1, v/v), followed by the addition of equimolar amounts of Ag_3Pz_3 (10.00 mg, 0.0107 mmol). The resulting mixed solution was filtered and then transferred to a 20 mL screw-capped sample vial. The cap of the sample vial was loosely closed to allow the solvent to slowly evaporate at room temperature. The entire co-crystal incubation process was protected from light using aluminum foil. After the designated evaporation period, typically 1-3 days, high-quality colorless needle-shaped crystals suitable for single-crystal X-ray diffraction analysis formed at the bottom of the vial.

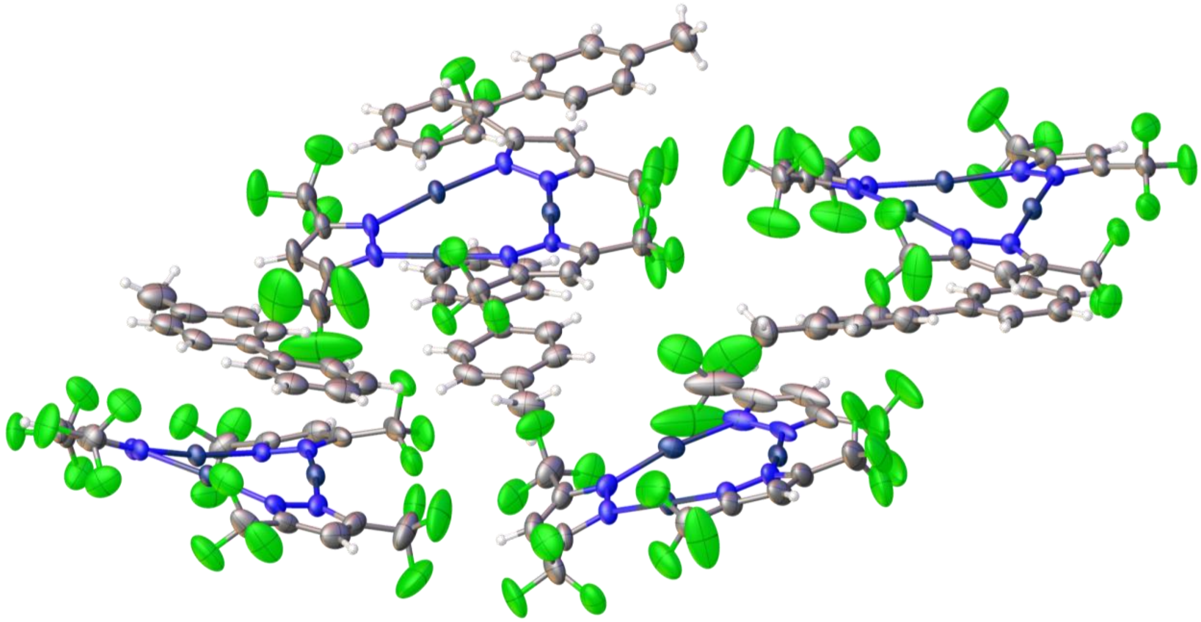

Figure S319. Asymmetric unit of **Ag_3Pz_3·101** (thermal displacement parameters at the 50% probability level).

Table S109. Crystal data and structure refinement for **Ag_3Pz_3·101**

Empirical formula	$C_{112}H_{60}Ag_{12}F_{72}N_{24}$
Formula weight	4404.28
Temperature/K	100.00(10)
Crystal system	monoclinic
Space group	$P2_1/n$
a/Å	21.6879(2)
b/Å	24.5290(2)
c/Å	25.4511(2)
α/°	90
β/°	91.0090(10)
γ/°	90
Volume/Å^3	13537.4(2)
Z	4
ρ_{calc}g/cm^3	2.161
μ/mm^{-1}	15.025
F(000)	8448.0
Crystal size/mm^3	0.16 × 0.13 × 0.115
Radiation	Cu Kα (λ = 1.54184)
2θ range for data collection/°	5.004 to 149.934
Index ranges	$-26 \le h \le 27$, $-30 \le k \le 30$, $-31 \le l \le 24$
Reflections collected	101523
Independent reflections	26482 [R_{int} = 0.0511, R_{sigma} = 0.0396]
Data/restraints/parameters	26482/32/1985
Goodness-of-fit on F^2	1.035
Final R indexes [I>=2σ (I)]	R_1 = 0.0487, wR_2 = 0.1274
Final R indexes [all data]	R_1 = 0.0579, wR_2 = 0.1332
Largest diff. peak/hole / e Å^{-3}	1.96/-1.25
CCDC-number	2501844

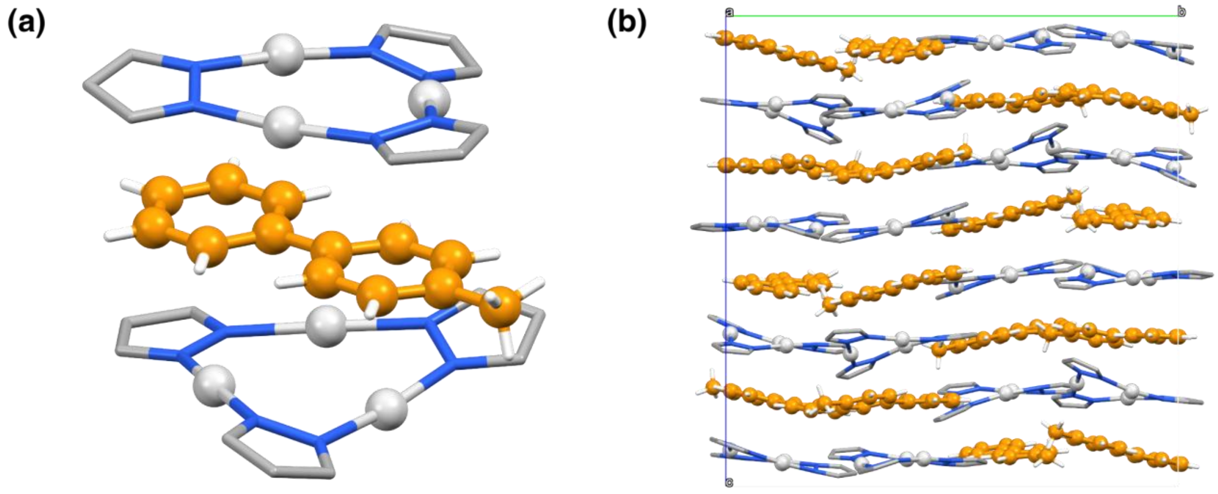

Figure S320. (a) A schematic diagram of the co-crystal structure in the **Ag_3Pz_3·101** single crystal, formed by the guest organic molecule and the surrounding Ag_3Pz_3 units that exhibit significant interactions with it. (b) A 1 × 1 × 1 packing mode in the single crystal structure of **Ag_3Pz_3·101** along the *a* axis. Trifluoromethyl groups and H atoms in Ag_3Pz_3 are omitted for clarity. C, N, and Ag atoms in Ag_3Pz_3 are depicted in dark gray, light blue, and light gray, respectively; C and H atoms in **101** are depicted in orange and white, respectively.

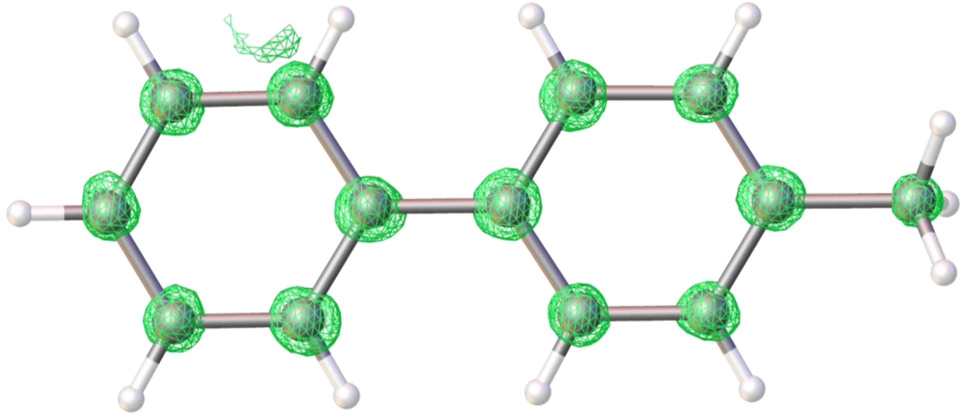

Figure S321. F_{obs} (contour: 0.17) electron density map superimposed on the structure of **101** in the single crystal structure of **Ag_3Pz_3·101**.

Preparation of Ag_3Pz_3·102. 3.84 mg (0.0107 mmol) of 4-bromo-4'-iodobiphenyl (**102**) was dissolved in 3 mL of a binary solvent system of DCM and n-Hex (1:1, v/v), followed by the addition of equimolar amounts of Ag_3Pz_3 (10.00 mg, 0.0107 mmol). The resulting mixed solution was filtered and then transferred to a 20 mL screw-capped sample vial. The cap of the sample vial was loosely closed to allow the solvent to slowly evaporate at room temperature. The entire co-crystal incubation process was protected from light using aluminum foil. After the designated evaporation period, typically 1-3 days, high-quality colorless plate-shaped crystals suitable for single-crystal X-ray diffraction analysis formed at the bottom of the vial.

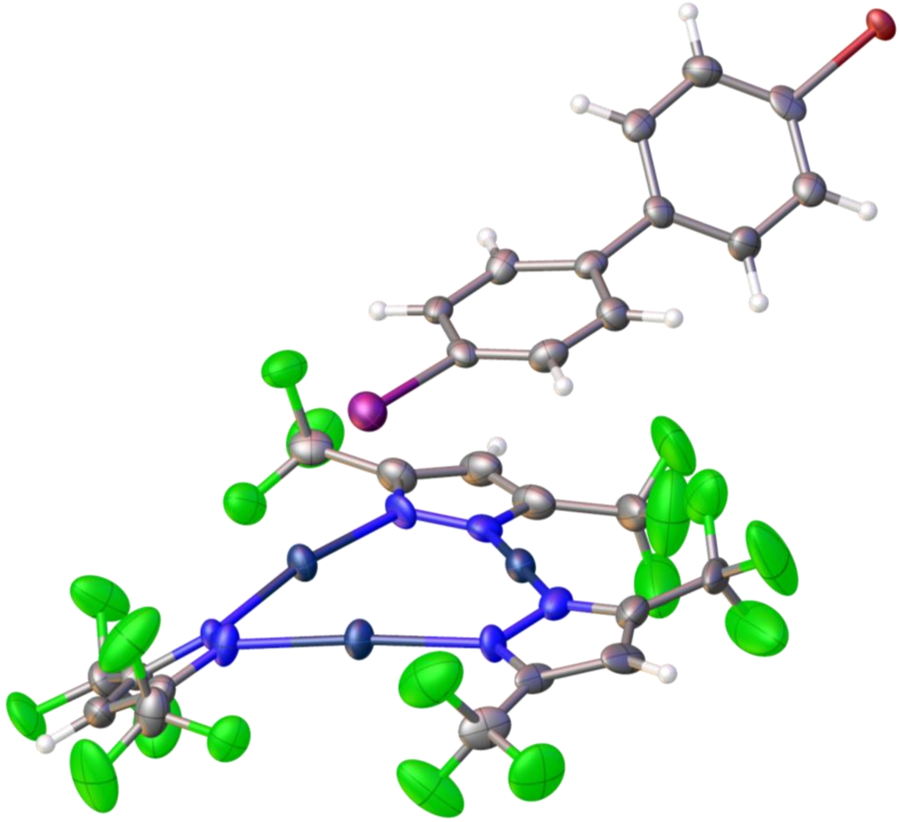

Figure S322. Asymmetric unit of **Ag_3Pz_3·102** (thermal displacement parameters at the 50% probability level).

Table S110. Crystal data and structure refinement for **Ag_3Pz_3·102**

Empirical formula	$C_{27}H_{11}Ag_3BrF_{18}IN_6$
Formula weight	1291.84
Temperature/K	100.0(2)
Crystal system	monoclinic
Space group	$P2_1/c$
a/Å	13.4294(4)
b/Å	18.0985(4)
c/Å	15.4233(4)
α/°	90
β/°	110.490(3)
γ/°	90
Volume/Å^3	3511.50(17)
Z	4
ρ_{calc}g/cm^3	2.444
μ/mm^{-1}	22.730
F(000)	2424.0
Crystal size/mm^3	0.16 × 0.16 × 0.15
Radiation	Cu Kα (λ = 1.54184)
2θ range for data collection/°	7.026 to 157.246
Index ranges	$-16 \leq h \leq 14$, $-21 \leq k \leq 22$, $-19 \leq l \leq 18$
Reflections collected	18276
Independent reflections	7257 [R_{int} = 0.0572, R_{sigma} = 0.0581]
Data/restraints/parameters	7257/51/505
Goodness-of-fit on F^2	1.051
Final R indexes [I>=2σ (I)]	R_1 = 0.0877, wR_2 = 0.2362
Final R indexes [all data]	R_1 = 0.1015, wR_2 = 0.2445
Largest diff. peak/hole / e Å^{-3}	4.79/-2.65
CCDC-number	2501845

Responses to CheckCIF alerts for Ag_3Pz_3·102 crystal structure:

A-level alerts:

"Check Calcd Resid. Dens. 0.20Ang From Br05 5.06 eA-3"

This Alert is due to presence of residual density in the presence of heavy metal atom.

"Check Calcd Resid. Dens. 1.32Ang From F01F 3.81 eA-3"

This Alert is due to presence of residual density in the presence of heavy metal atom.

B-level alerts:

"Low Bond Precision on C-C Bonds .... 0.02116 Ang."

Disordered structure.

"Check Calcd Resid. Dens. 0.99Ang From N00J 3.30 eA-3"

This Alert is due to presence of residual density in the presence of heavy metal atom.

"Check Calcd Resid. Dens. 1.12Ang From F01G 3.05 eA-3"

This Alert is due to presence of residual density in the presence of heavy metal atom.

"Check Calcd Resid. Dens. 1.04Ang From N00B 2.75 eA-3"

This Alert is due to presence of residual density in the presence of heavy metal atom.

"Check Calcd Resid. Dens. 1.01Ang From F01F 2.66 eA-3"

This Alert is due to presence of residual density in the presence of heavy metal atom.

"Check Calcd Resid. Dens. 0.61Ang From I04 -2.82 eA-3"

This Alert is due to presence of residual density in the presence of heavy metal atom.

"Check Calcd Resid. Dens. 0.66Ang From I04 -2.57 eA-3"

This Alert is due to presence of residual density in the presence of heavy metal atom.

"Check Calcd Resid. Dens. 0.55Ang From I04 -2.52 eA-3"

This Alert is due to presence of residual density in the presence of heavy metal atom.

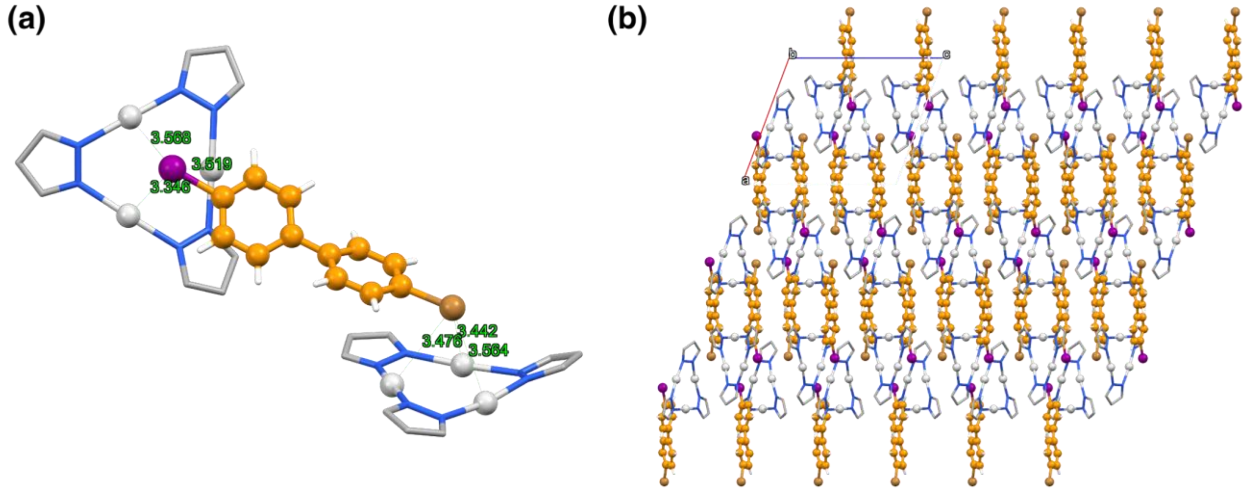

Figure S323. (a) A schematic diagram of the co-crystal structure in the **Ag_3Pz_3·102** single crystal, formed by the guest organic molecule and the surrounding Ag_3Pz_3 units that exhibit significant interactions with it. (b) A 3 × 1 × 3 packing mode in the single crystal structure of **Ag_3Pz_3·102** along the *b* axis. Trifluoromethyl groups and H atoms in Ag_3Pz_3 are omitted for clarity. Ag···I and Ag···Br interactions are indicated with green dotted lines with distances in Å. C, N, and Ag atoms in Ag_3Pz_3 are depicted in dark gray, light blue, and light gray, respectively; C, Br, I, and H atoms in **102** are depicted in orange, brown, purple, and white, respectively.

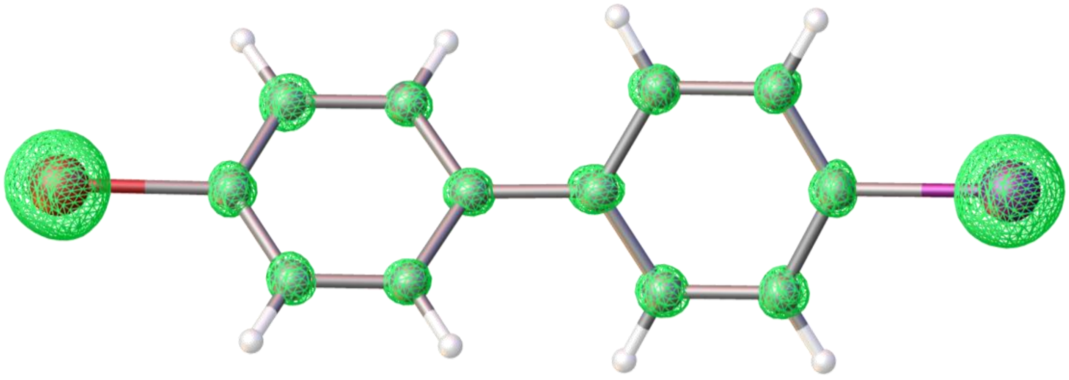

Figure S324. F_{obs} (contour: 1.10) electron density map superimposed on the structure of **102** in the single crystal structure of **Ag_3Pz_3·102**.

Preparation of Ag_3Pz_3·103. 3.62 mg (0.0107 mmol) of idebenone (**103**) was dissolved in 3 mL of c-Hex, followed by the addition of equimolar amounts of Ag_3Pz_3 (10.00 mg, 0.0107 mmol). The resulting mixed solution was filtered and then transferred to a 20 mL screw-capped sample vial. The cap of the sample vial was loosely closed to allow the solvent to slowly evaporate at room temperature. The entire co-crystal incubation process was protected from light using aluminum foil. After the designated evaporation period, typically 1-3 days, high-quality orange prism-shaped crystals suitable for single-crystal X-ray diffraction analysis formed at the bottom of the vial.

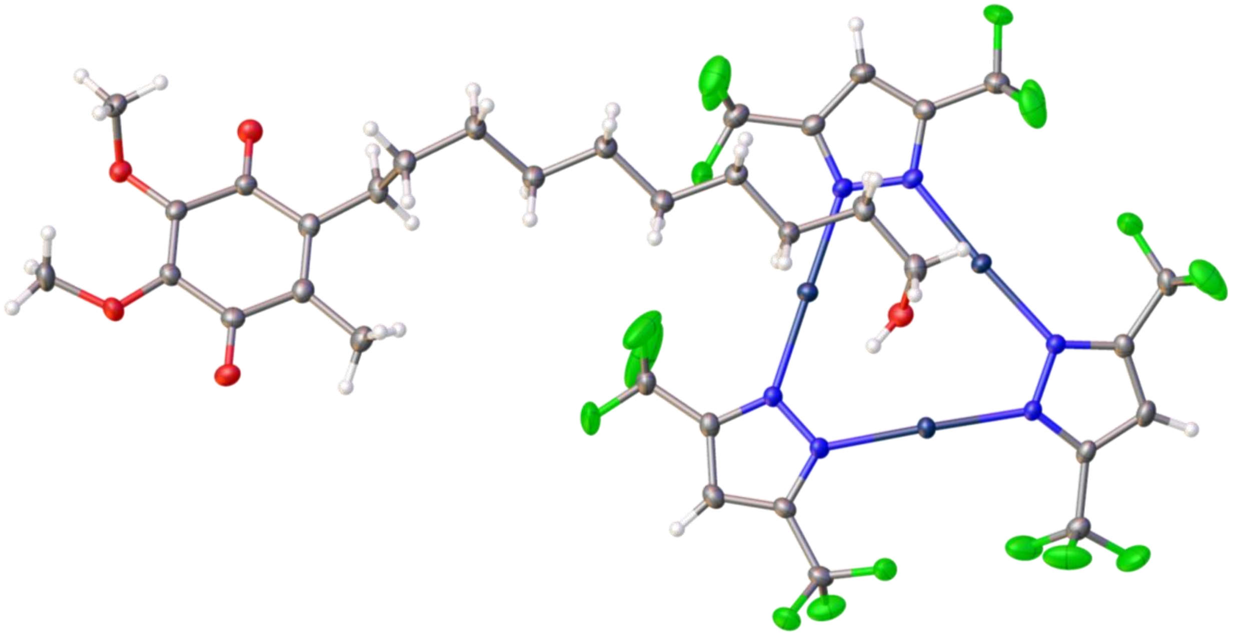

Figure S325. Asymmetric unit of **Ag_3Pz_3·103** (thermal displacement parameters at the 50% probability level).

Table S111. Crystal data and structure refinement for **Ag_3Pz_3·103**

Empirical formula	$C_{34}H_{33}Ag_3F_{18}N_6O_5$
Formula weight	1271.27
Temperature/K	100.00(13)
Crystal system	monoclinic
Space group	$P2_1/c$
a/Å	19.10820(10)
b/Å	13.42820(10)
c/Å	17.36750(10)
α/°	90
β/°	105.4360(10)
γ/°	90
Volume/Å^3	4295.56(5)
Z	4
ρ_{calc}g/cm^3	1.966
μ/mm^{-1}	12.031
F(000)	2488.0
Crystal size/mm^3	0.25 × 0.24 × 0.14
Radiation	Cu Kα (λ = 1.54184)
2θ range for data collection/°	8.148 to 156.882
Index ranges	$-16 \leq h \leq 24$, $-17 \leq k \leq 9$, $-21 \leq l \leq 21$
Reflections collected	23243
Independent reflections	8857 [R_{int} = 0.0261, R_{sigma} = 0.0292]
Data/restraints/parameters	8857/0/599
Goodness-of-fit on F^2	1.049
Final R indexes [I>=2σ (I)]	R_1 = 0.0396, wR_2 = 0.1037
Final R indexes [all data]	R_1 = 0.0418, wR_2 = 0.1052
Largest diff. peak/hole / e Å^{-3}	1.08/-1.02
CCDC-number	2501846

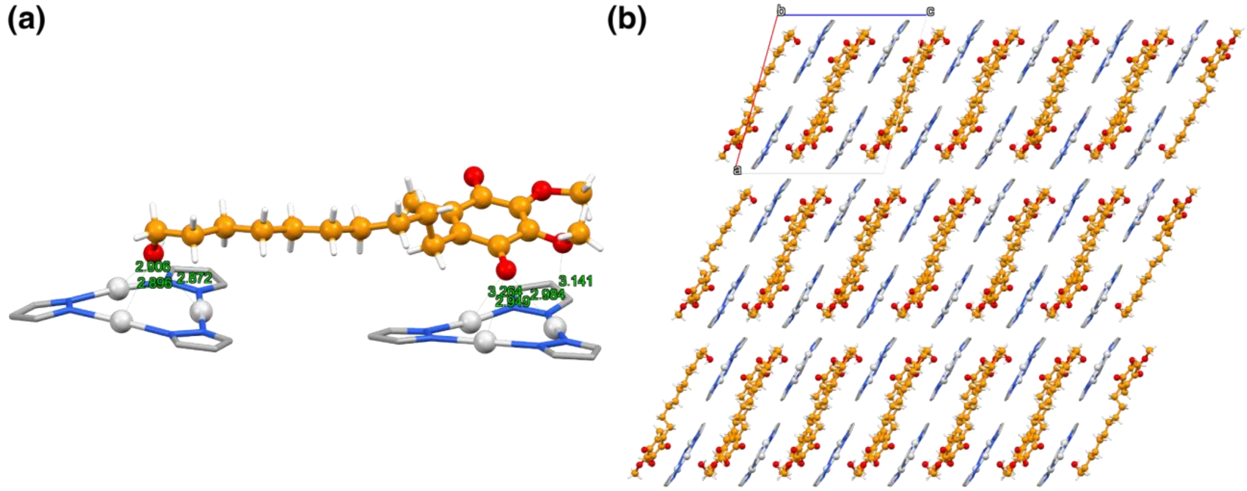

Figure S326. (a) A schematic diagram of the co-crystal structure in the **Ag_3Pz_3·103** single crystal, formed by the guest organic molecule and the surrounding Ag_3Pz_3 units that exhibit significant interactions with it. (b) A 3 × 1 × 3 packing mode in the single crystal structure of **Ag_3Pz_3·103** along the *b* axis. Trifluoromethyl groups and H atoms in Ag_3Pz_3 are omitted for clarity. Ag···O interactions are indicated with green dotted lines with distances in Å. C, N, and Ag atoms in Ag_3Pz_3 are depicted in dark gray, light blue, and light gray, respectively; C, O, and H atoms in **103** are depicted in orange, red, and white, respectively.

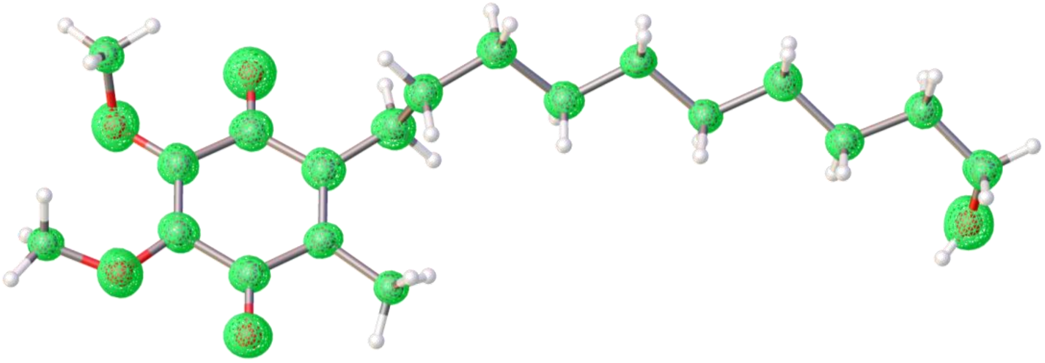

Figure S327. F_{obs} (contour: 1.10) electron density map superimposed on the structure of **103** in the single crystal structure of **Ag_3Pz_3·103**.

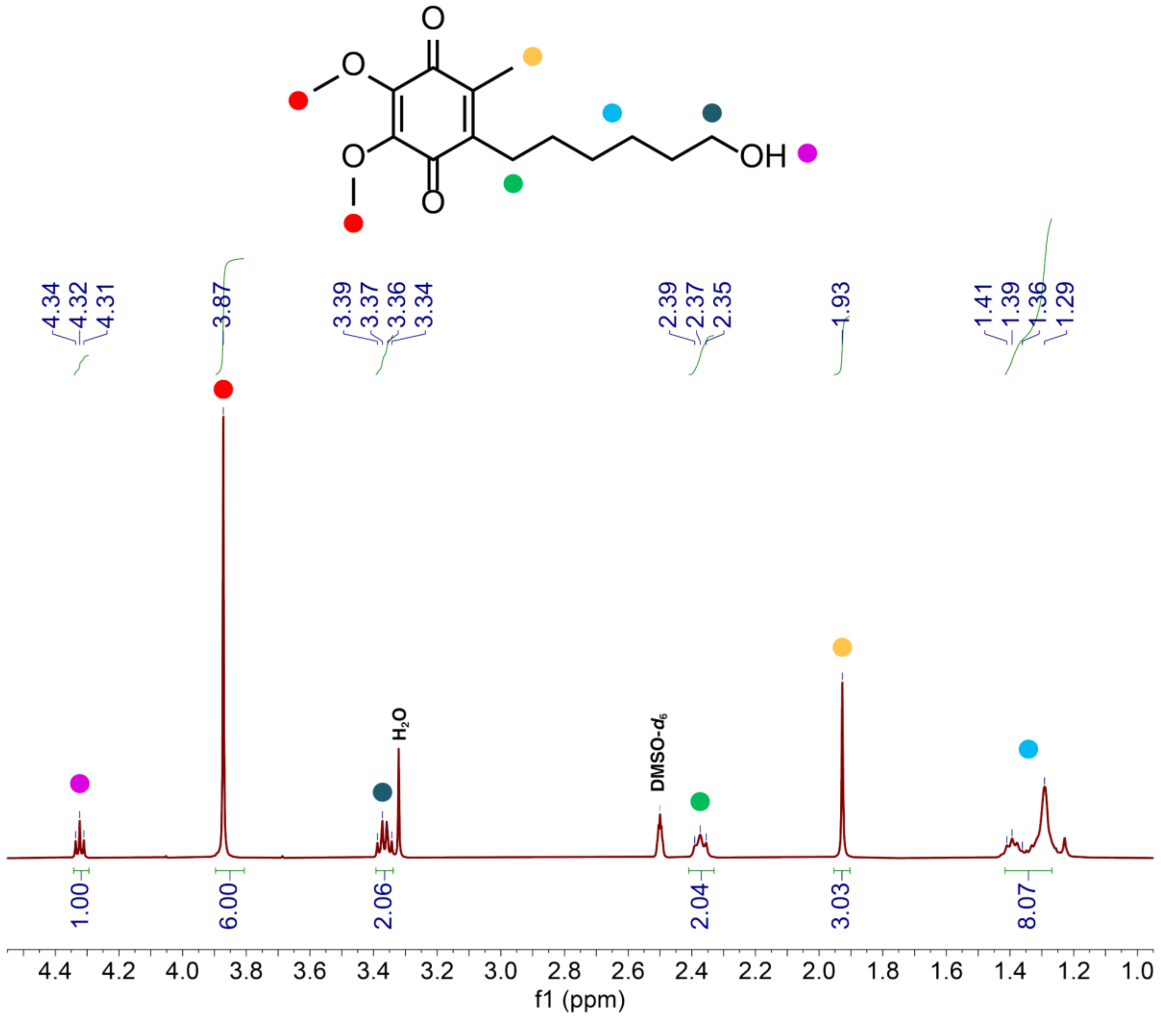

Figure S328. ^{1}H NMR of 6-(6-Hydroxyhexyl)-2,3-dimethoxy-5-methyl-1,4-benzoquinone (**104**) in DMSO-d_6.

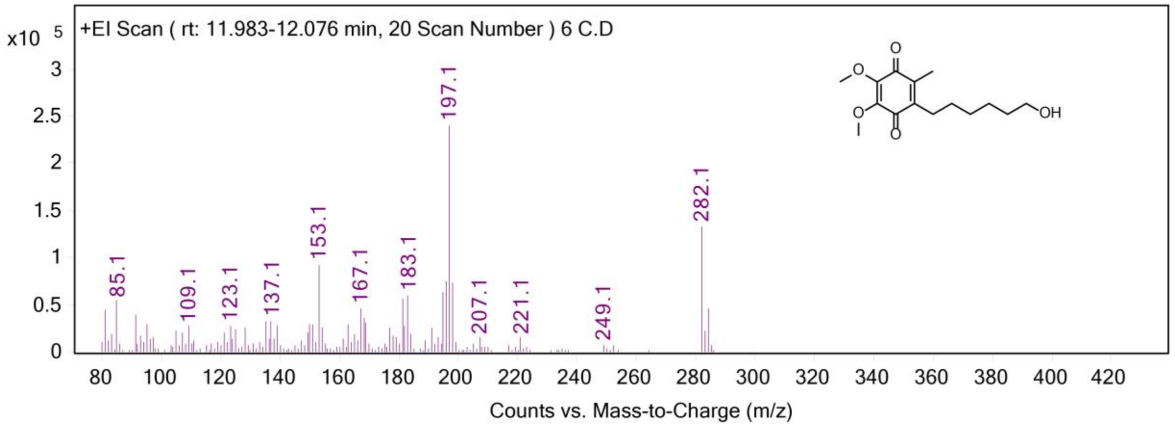

Figure S329. GC–MS EI mass spectrum of **104**.

Preparation of Ag_3Pz_3·104. 3.02 mg (0.0107 mmol) of 6-(6-hydroxyhexyl)-2,3-dimethoxy-5-methyl-1,4-benzoquinone (**104**) was dissolved in 3 mL of a binary solvent system of n-Hex and c-Hex (1:1, v/v), followed by the addition of equimolar amounts of Ag_3Pz_3 (10.00 mg, 0.0107 mmol). The resulting mixed solution was filtered and then transferred to a 20 mL screw-capped sample vial. The cap of the sample vial was loosely closed to allow the solvent to slowly evaporate at room temperature. The entire co-crystal incubation process was protected from light using aluminum foil. After the designated evaporation period, typically 1-3 days, high-quality orange prism-shaped crystals suitable for single-crystal X-ray diffraction analysis formed at the bottom of the vial.

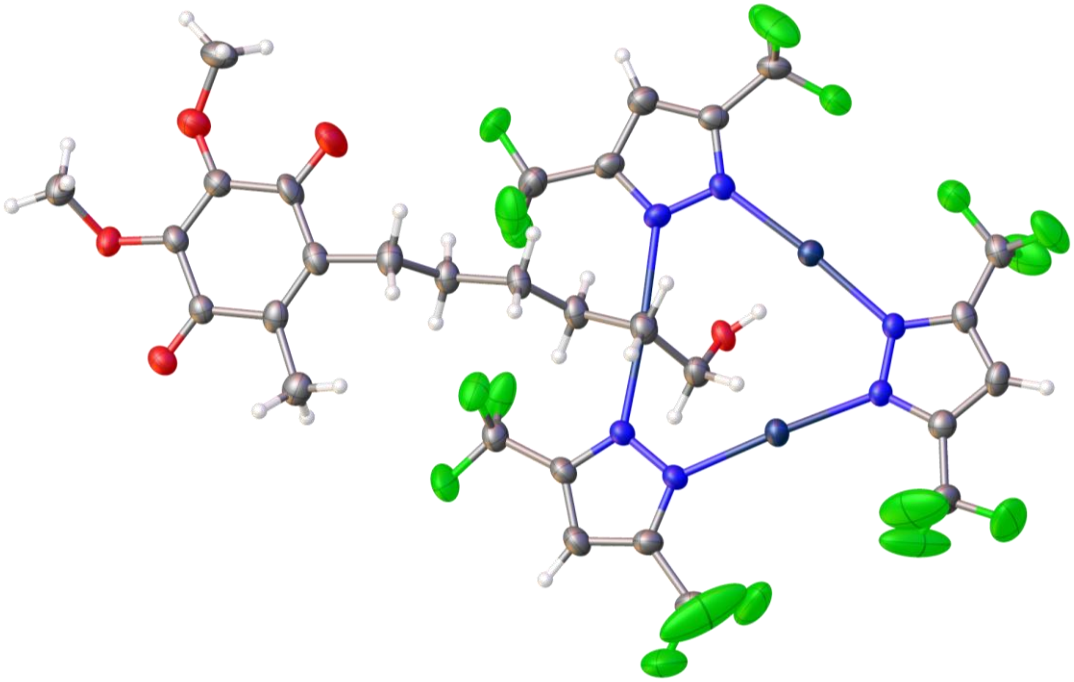

Figure S330. Asymmetric unit of **Ag_3Pz_3·104** (thermal displacement parameters at the 50% probability level).

Table S112. Crystal data and structure refinement for **Ag_3Pz_3·104**

Empirical formula	$C_{30}H_{25}Ag_3F_{18}N_6O_5$
Formula weight	1215.17
Temperature/K	100.0(3)
Crystal system	monoclinic
Space group	$P2_1/c$
a/Å	9.9293(3)
b/Å	37.1724(8)
c/Å	10.6654(3)
α/°	90
β/°	99.098(2)
γ/°	90
Volume/Å^3	3887.03(18)
Z	4
ρ_{calc}g/cm^3	2.076
μ/mm^{-1}	13.258
F(000)	2360.0
Crystal size/mm^3	0.14 × 0.13 × 0.11
Radiation	Cu Kα (λ = 1.54184)
2θ range for data collection/°	8.728 to 155.56
Index ranges	$-12 \le h \le 10$, $-46 \le k \le 34$, $-11 \le l \le 13$
Reflections collected	19680
Independent reflections	7971 [R_{int} = 0.0478, R_{sigma} = 0.0477]
Data/restraints/parameters	7971/24/563
Goodness-of-fit on F^2	1.070
Final R indexes [I>=2σ (I)]	R_1 = 0.0593, wR_2 = 0.1551
Final R indexes [all data]	R_1 = 0.0669, wR_2 = 0.1597
Largest diff. peak/hole / e Å^{-3}	1.58/-1.04
CCDC-number	2501847

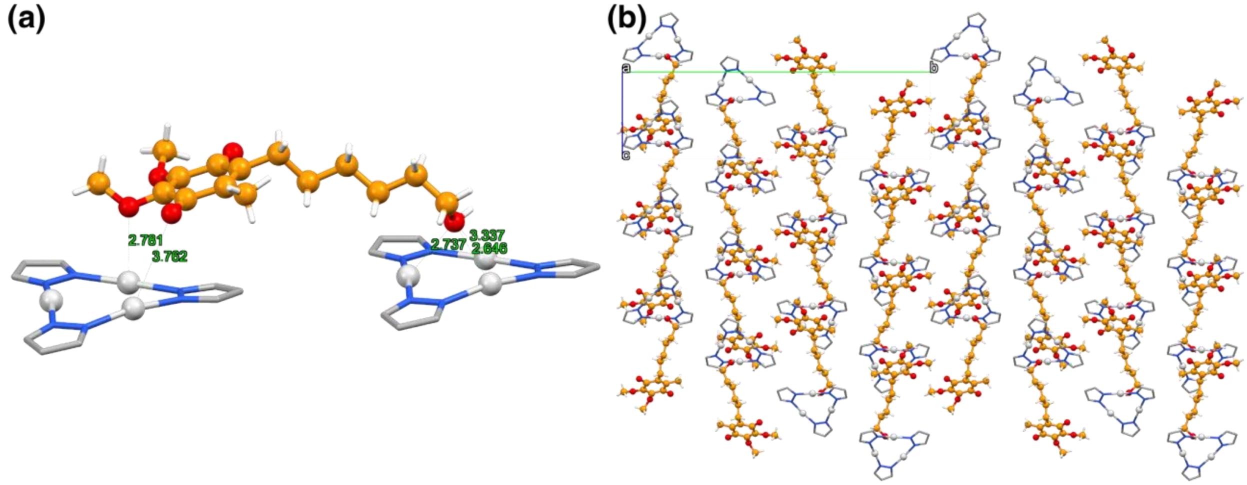

Figure S331. (a) A schematic diagram of the co-crystal structure in the $\mathbf{Ag_3Pz_3 \cdot 104}$ single crystal, formed by the guest organic molecule and the surrounding Ag_3Pz_3 units that exhibit significant interactions with it. (b) A 1 × 2 × 4 packing mode in the single crystal structure of $\mathbf{Ag_3Pz_3 \cdot 104}$ along the *a* axis. Trifluoromethyl groups and H atoms in Ag_3Pz_3 are omitted for clarity. Ag···O interactions are indicated with green dotted lines with distances in Å. C, N, and Ag atoms in Ag_3Pz_3 are depicted in dark gray, light blue, and light gray, respectively; C, O, and H atoms in **104** are depicted in orange, red, and white, respectively.

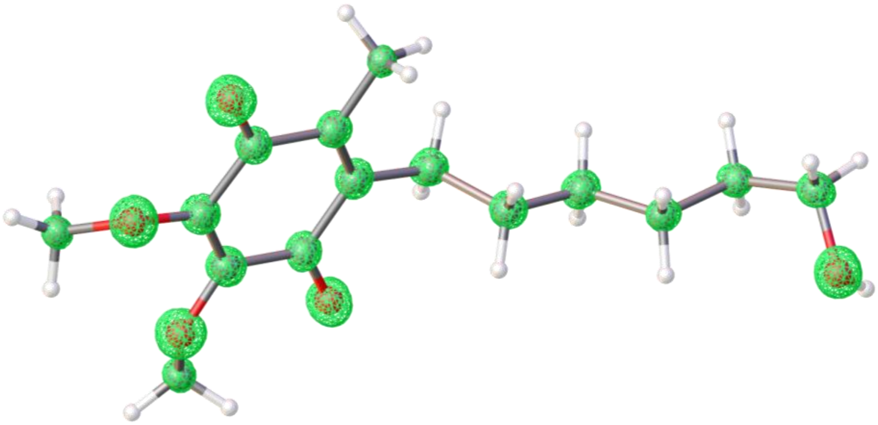

Figure S332. F_{obs} (contour: 1.15) electron density map superimposed on the structure of **104** in the single crystal structure of $\mathbf{Ag_3Pz_3 \cdot 104}$.

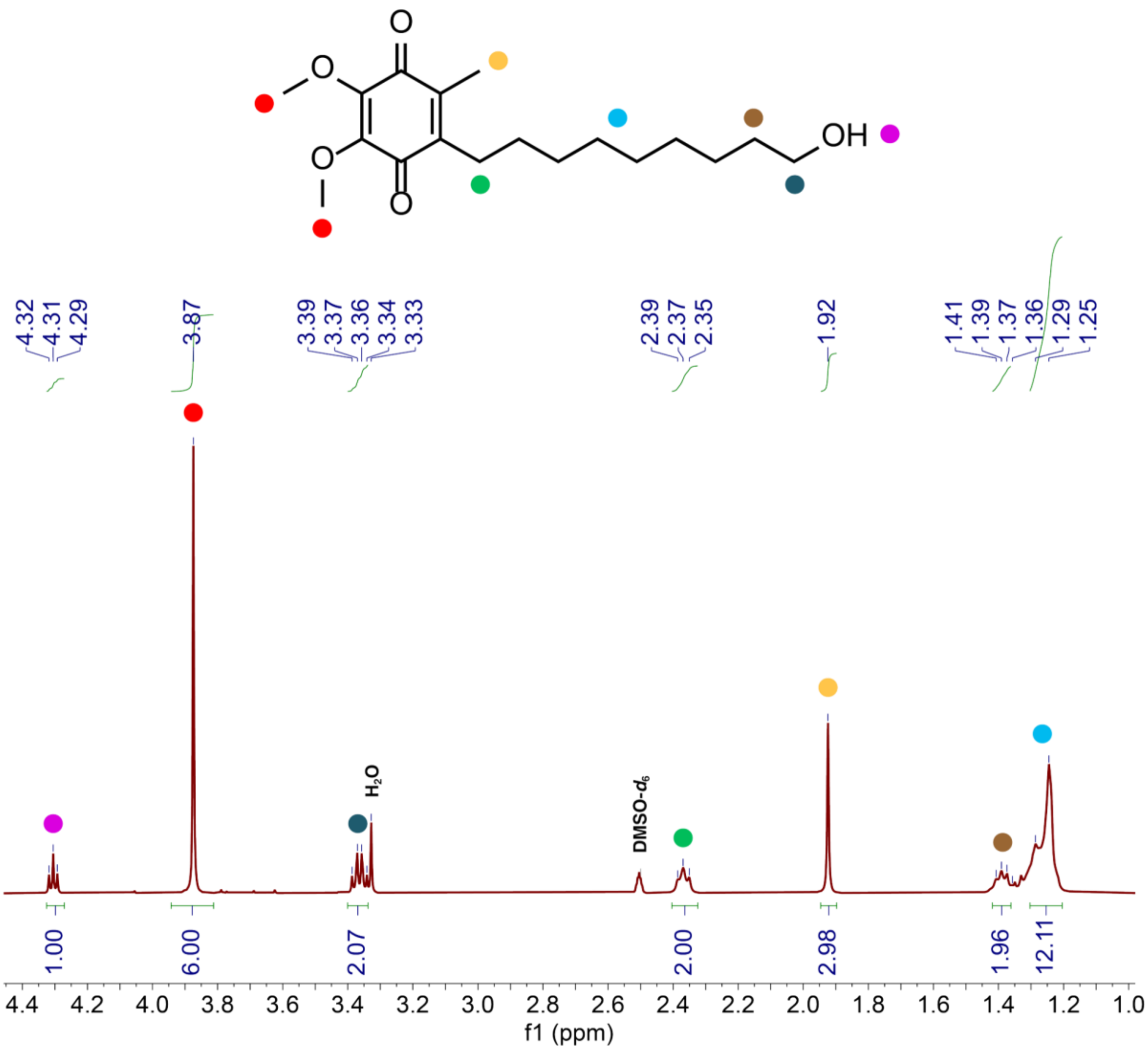

Figure S333. ^{1}H NMR of 6-(9-Hydroxynonyl)-2,3-dimethoxy-5-methyl-1,4-benzoquinone (**105**) in DMSO-d_6.

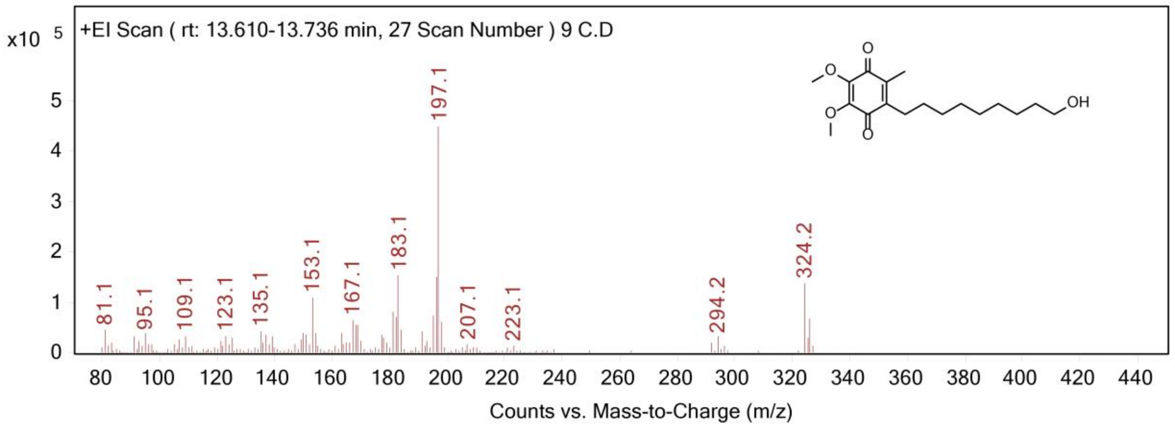

Figure S334. GC – MS EI mass spectrum of **105**.

Preparation of Ag_3Pz_3·105. 3.47 mg (0.0107 mmol) of 6-(9-hydroxynonyl)-2,3-dimethoxy-5-methyl-1,4-benzoquinone (**105**) was dissolved in 3 mL of c-Hex, followed by the addition of equimolar amounts of Ag_3Pz_3 (10.00 mg, 0.0107 mmol). The resulting mixed solution was filtered and then transferred to a 20 mL screw-capped sample vial. The cap of the sample vial was loosely closed to allow the solvent to slowly evaporate at room temperature. The entire co-crystal incubation process was protected from light using aluminum foil. After the designated evaporation period, typically 1-3 days, high-quality orange prism-shaped crystals suitable for single-crystal X-ray diffraction analysis formed at the bottom of the vial.

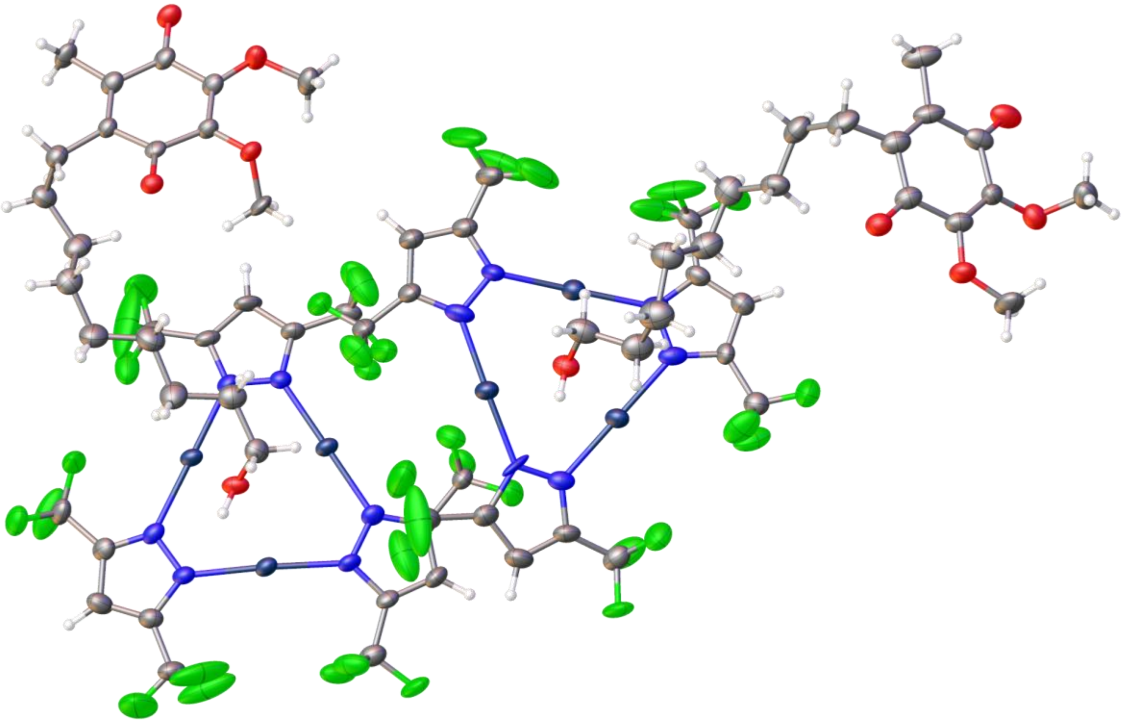

Figure S335. Asymmetric unit of **Ag_3Pz_3·105** (thermal displacement parameters at the 50% probability level).

Table S113. Crystal data and structure refinement for $\mathbf{Ag_3Pz_3 \cdot 105}$

Empirical formula	$C_{33}H_{31}Ag_3F_{18}N_6O_5$
Formula weight	1257.25
Temperature/K	100.00(10)
Crystal system	triclinic
Space group	$P\bar{1}$
a/Å	10.4806(2)
b/Å	10.8443(3)
c/Å	38.8707(6)
α/°	96.016(2)
β/°	95.9560(10)
γ/°	103.337(2)
Volume/Å^3	4237.49(16)
Z	4
ρ_{calc}g/cm^3	1.971
μ/mm^{-1}	12.187
F(000)	2456.0
Crystal size/mm^3	0.26 × 0.26 × 0.15
Radiation	Cu Kα (λ = 1.54184)
2θ range for data collection/°	6.92 to 133.2
Index ranges	$-12 \leq h \leq 12$, $-12 \leq k \leq 12$, $-46 \leq l \leq 37$
Reflections collected	61848
Independent reflections	14491 [R_{int} = 0.0566, R_{sigma} = 0.0548]
Data/restraints/parameters	14491/112/1181
Goodness-of-fit on F^2	1.048
Final R indexes [I>=2σ (I)]	R_1 = 0.0640, wR_2 = 0.1675
Final R indexes [all data]	R_1 = 0.0860, wR_2 = 0.1832
Largest diff. peak/hole / e Å^{-3}	4.41/-1.30
CCDC-number	2501848

Responses to CheckCIF alerts for Ag_3Pz_3·105 crystal structure:

A-level alerts:

"Check Calcd Resid. Dens. 1.24Ang From N012 4.56 eA-3"

This Alert is due to presence of residual density in the presence of heavy metal atom (Ag).

"Check Calcd Resid. Dens. 0.95Ang From N01E 4.14 eA-3"

This Alert is due to presence of residual density in the presence of heavy metal atom (Ag).

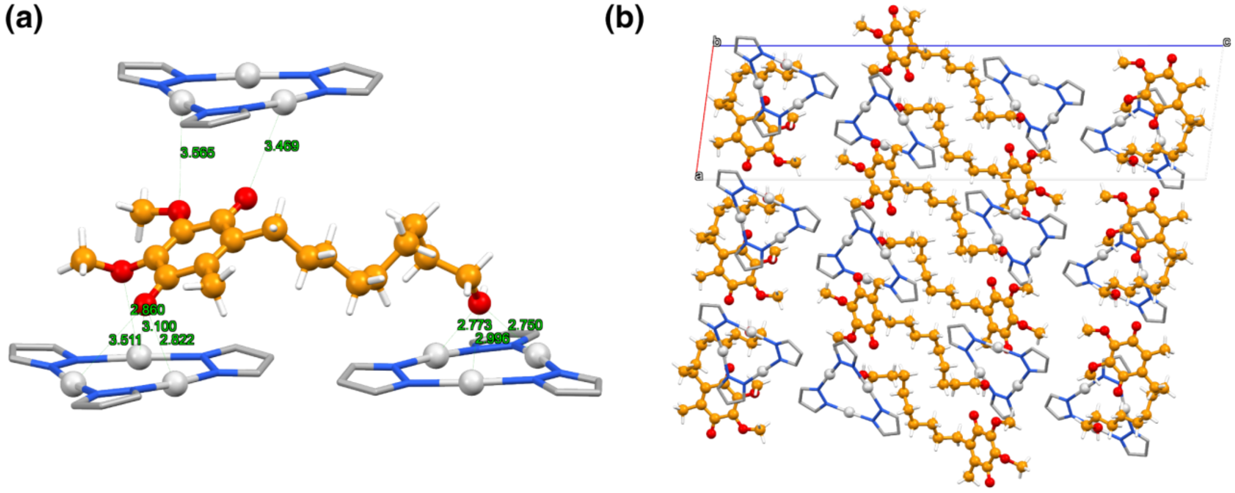

Figure S336. (a) A schematic diagram of the co-crystal structure in the **Ag_3Pz_3·105** single crystal, formed by the guest organic molecule and the surrounding Ag_3Pz_3 units that exhibit significant interactions with it. (b) A 3 × 1 × 1 packing mode in the single crystal structure of **Ag_3Pz_3·105** along the *b* axis. Trifluoromethyl groups and H atoms in Ag_3Pz_3 are omitted for clarity. Ag···O interactions are indicated with green dotted lines with distances in Å. C, N, and Ag atoms in Ag_3Pz_3 are depicted in dark gray, light blue, and light gray, respectively; C, O, and H atoms in **105** are depicted in orange, red, and white, respectively.

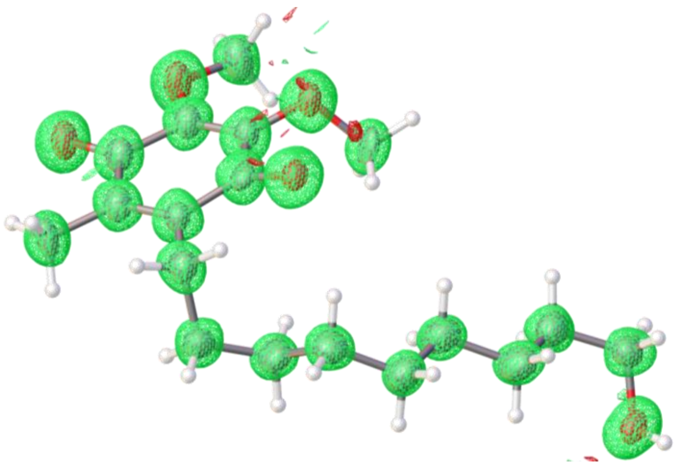

Figure S337. F_{obs} (contour: 0.30) electron density map superimposed on the structure of **105** in the single crystal structure of **Ag_3Pz_3·105**.

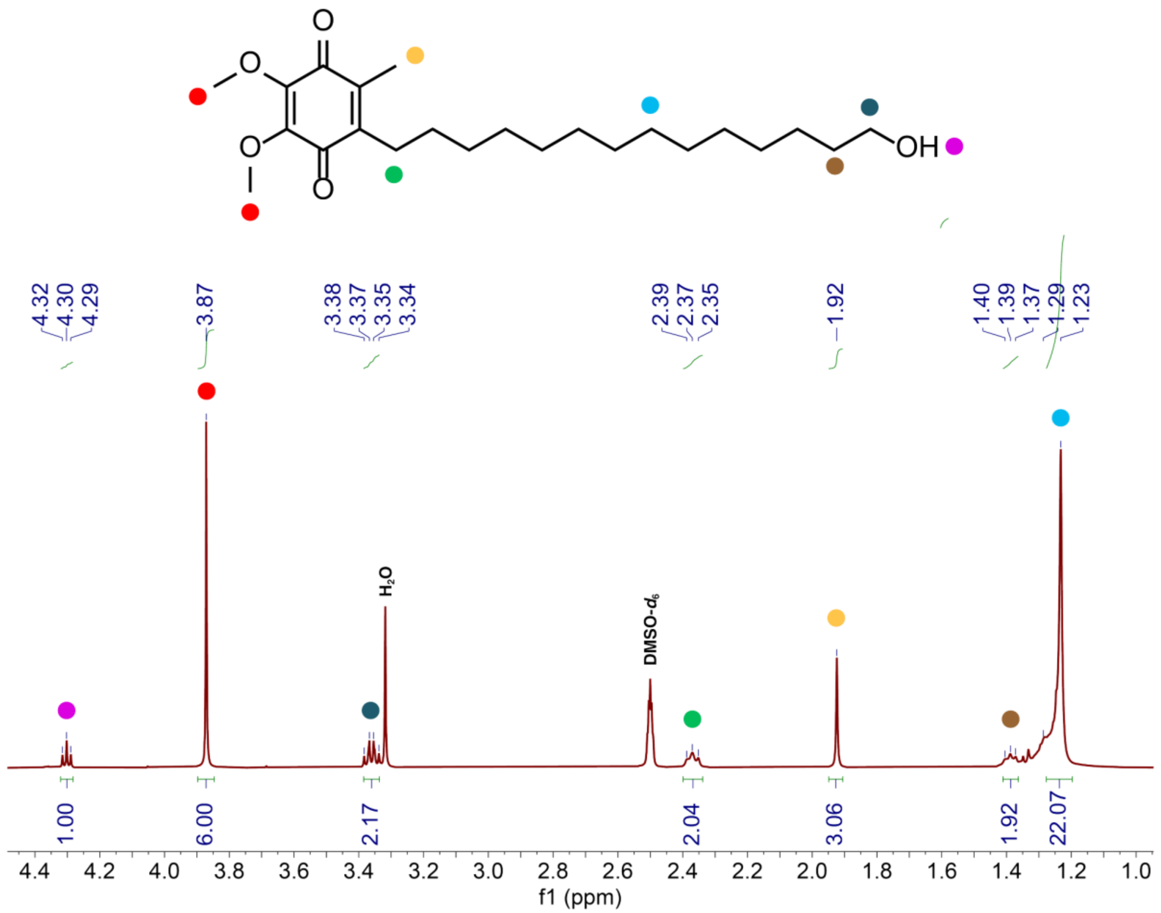

Figure S338. ^{1}H NMR of 6-(14-Hydroxytetradecyl)-2,3-dimethoxy-5-methyl-1,4-benzoquinone (**106**) in DMSO-d_6.

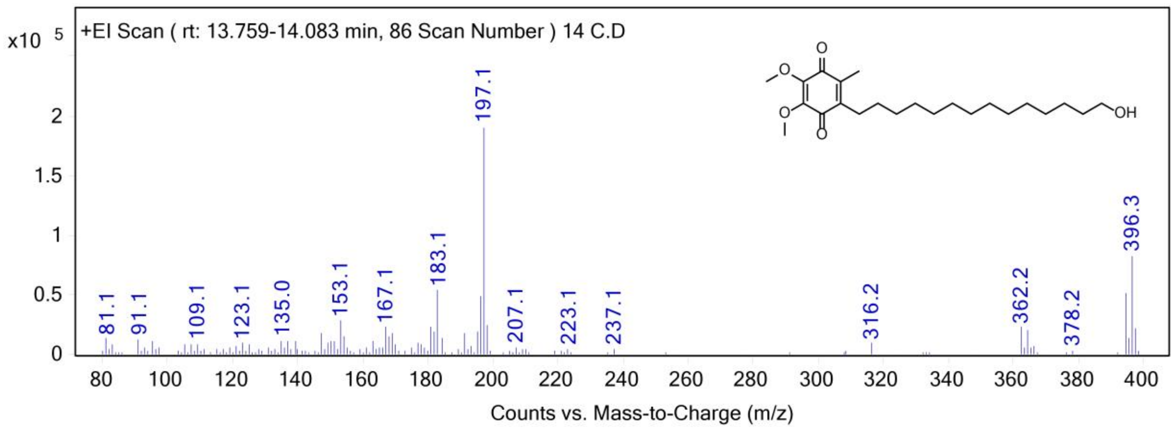

Figure S339. GC – MS EI mass spectrum of **106**.

Preparation of Ag_3Pz_3·106. 4.22 mg (0.0107 mmol) of 6-(14-hydroxytetradecyl)-2,3-dimethoxy-5-methyl-1,4-benzoquinone (**106**) was dissolved in 3 mL of n-Hex, followed by the addition of equimolar amounts of Ag_3Pz_3 (10.00 mg, 0.0107 mmol). The resulting mixed solution was filtered and then transferred to a 20 mL screw-capped sample vial. The cap of the sample vial was loosely closed to allow the solvent to slowly evaporate at room temperature. The entire co-crystal incubation process was protected from light using aluminum foil. After the designated evaporation period, typically 1-3 days, high-quality orange prism-shaped crystals suitable for single-crystal X-ray diffraction analysis formed at the bottom of the vial.

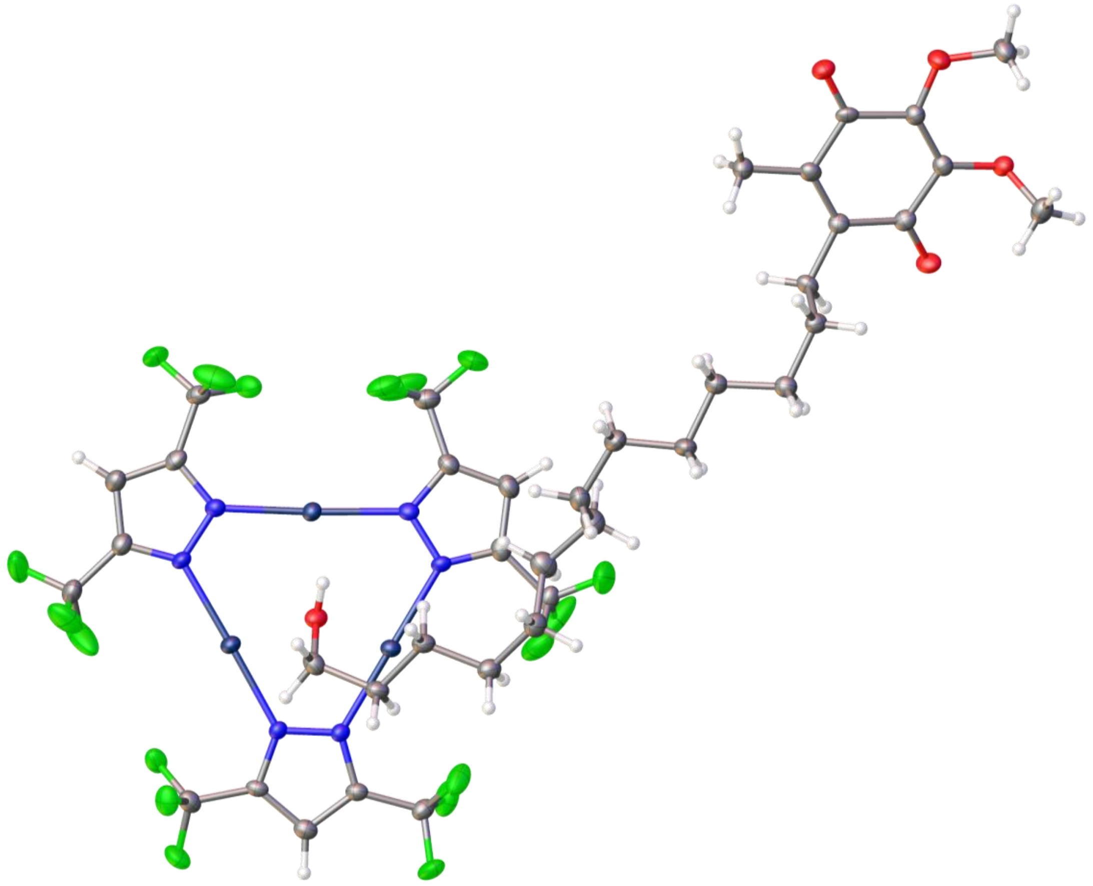

Figure S340. Asymmetric unit of **Ag_3Pz_3·106** (thermal displacement parameters at the 50% probability level).

Table S114. Crystal data and structure refinement for $\mathbf{Ag_3Pz_3 \cdot 106}$

Empirical formula	$C_{38}H_{41}Ag_3F_{18}N_6O_5$
Formula weight	1327.38
Temperature/K	100.00(10)
Crystal system	triclinic
Space group	$P\bar{1}$
a/Å	9.99070(10)
b/Å	13.2568(2)
c/Å	18.8672(2)
α/°	79.9710(10)
β/°	75.5510(10)
γ/°	80.5680(10)
Volume/Å^3	2363.77(5)
Z	2
ρ_{calc}g/cm^3	1.865
μ/mm^{-1}	10.963
F(000)	1308.0
Crystal size/mm^3	0.21 × 0.15 × 0.14
Radiation	Cu Kα (λ = 1.54184)
2θ range for data collection/°	4.884 to 156.376
Index ranges	$-12 \le h \le 12$, $-15 \le k \le 16$, $-23 \le l \le 17$
Reflections collected	24876
Independent reflections	9711 [R_{int} = 0.0334, R_{sigma} = 0.0355]
Data/restraints/parameters	9711/0/635
Goodness-of-fit on F^2	1.056
Final R indexes [I>=2σ (I)]	R_1 = 0.0449, wR_2 = 0.1226
Final R indexes [all data]	R_1 = 0.0510, wR_2 = 0.1253
Largest diff. peak/hole / e Å^{-3}	1.09/-1.17
CCDC-number	2501849

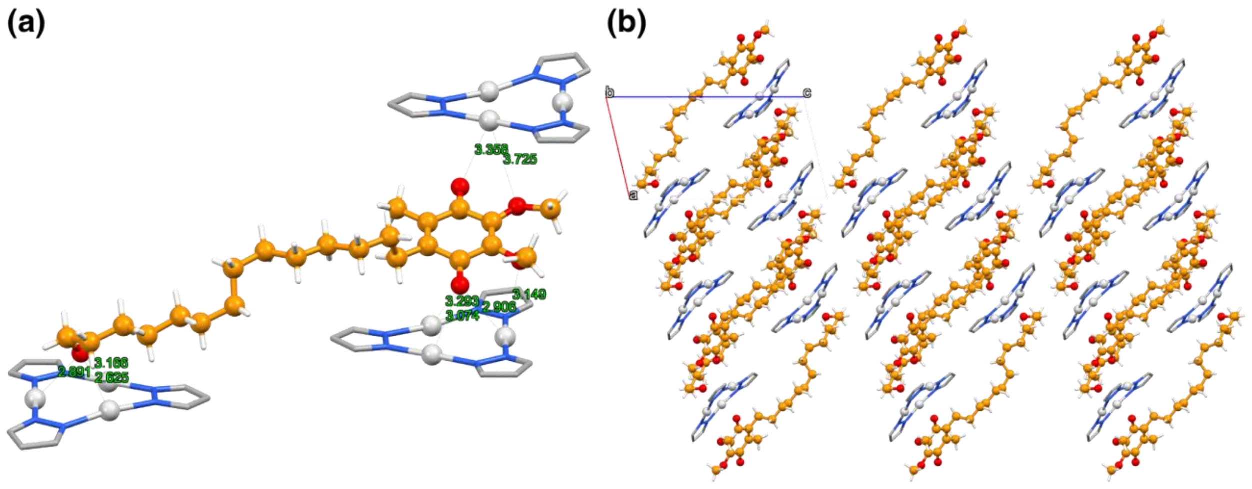

Figure S341. (a) A schematic diagram of the co-crystal structure in the **Ag_3Pz_3·106** single crystal, formed by the guest organic molecule and the surrounding Ag_3Pz_3 units that exhibit significant interactions with it. (b) A 3 × 1 × 3 packing mode in the single crystal structure of **Ag_3Pz_3·106** along the *b* axis. Trifluoromethyl groups and H atoms in Ag_3Pz_3 are omitted for clarity. Ag···O interactions are indicated with green dotted lines with distances in Å. C, N, and Ag atoms in Ag_3Pz_3 are depicted in dark gray, light blue, and light gray, respectively; C, O, and H atoms in **106** are depicted in orange, red, and white, respectively.

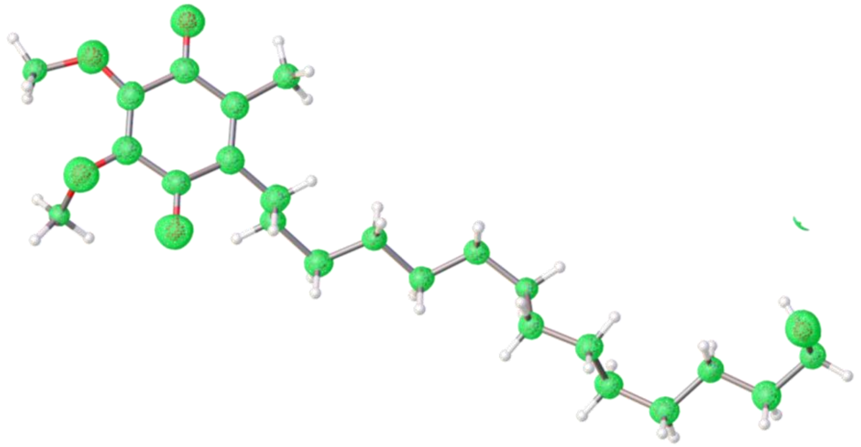

Figure S342. F_{obs} (contour: 1.90) electron density map superimposed on the structure of **106** in the single crystal structure of **Ag_3Pz_3·106**.

Preparation of Ag_3Pz_3·107. 3.62 mg (0.0107 mmol) of 6-(10-hydroxydecanoyl)-2,3-dimethoxy-5-methylphenol (**107**) was dissolved in 3 mL of n-Hex, followed by the addition of equimolar amounts of Ag_3Pz_3 (10.00 mg, 0.0107 mmol). The resulting mixed solution was filtered and then transferred to a 20 mL screw-capped sample vial. The cap of the sample vial was loosely closed to allow the solvent to slowly evaporate at room temperature. The entire co-crystal incubation process was protected from light using aluminum foil. After the designated evaporation period, typically 1-3 days, high-quality colorless prism-shaped crystals suitable for single-crystal X-ray diffraction analysis formed at the bottom of the vial.

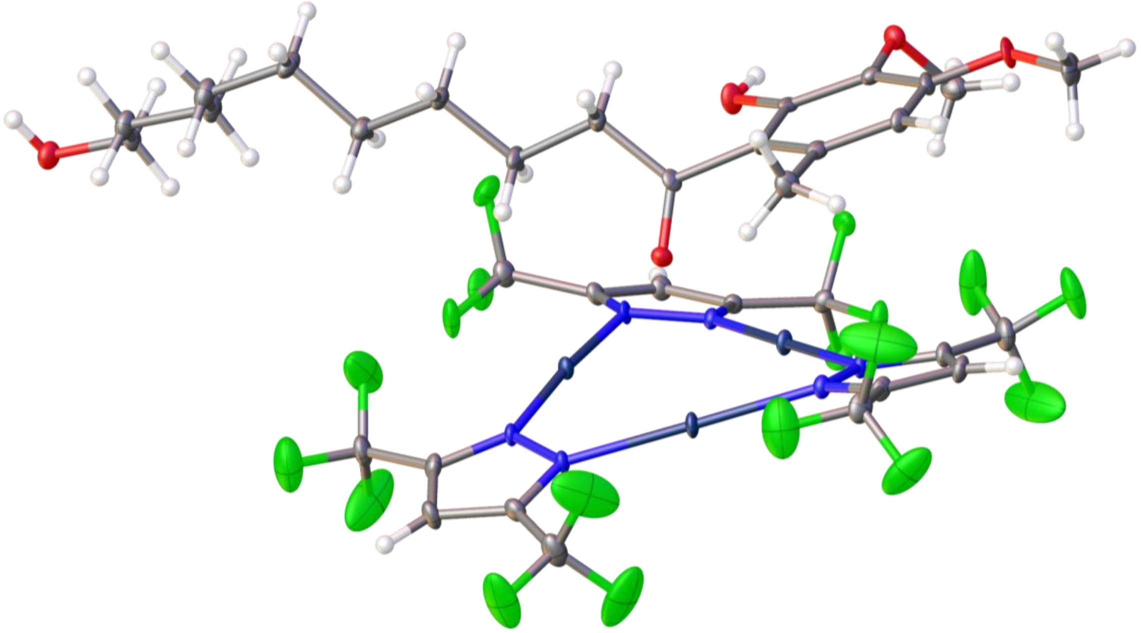

Figure S343. Asymmetric unit of **Ag_3Pz_3·107** (thermal displacement parameters at the 50% probability level).

Table S115. Crystal data and structure refinement for **Ag_3Pz_3·107**

Empirical formula	$C_{34}H_{33}Ag_3F_{18}N_6O_5$
Formula weight	1271.27
Temperature/K	100.01(12)
Crystal system	monoclinic
Space group	$P2_1/n$
a/Å	12.91607(6)
b/Å	14.44842(7)
c/Å	22.98595(11)
α/°	90
β/°	91.3724(4)
γ/°	90
Volume/Å^3	4288.34(3)
Z	4
ρ_{calc}g/cm^3	1.969
μ/mm^{-1}	12.051
F(000)	2488.0
Crystal size/mm^3	0.3 × 0.2 × 0.1
Radiation	Cu Kα (λ = 1.54184)
2θ range for data collection/°	7.228 to 156.922
Index ranges	$-12 \leq h \leq 16$, $-18 \leq k \leq 17$, $-29 \leq l \leq 28$
Reflections collected	24254
Independent reflections	8870 [R_{int} = 0.0270, R_{sigma} = 0.0226]
Data/restraints/parameters	8870/12/600
Goodness-of-fit on F^2	1.062
Final R indexes [I>=2σ (I)]	R_1 = 0.0411, wR_2 = 0.1123
Final R indexes [all data]	R_1 = 0.0415, wR_2 = 0.1128
Largest diff. peak/hole / e Å^{-3}	1.74/-2.06
CCDC-number	2501850

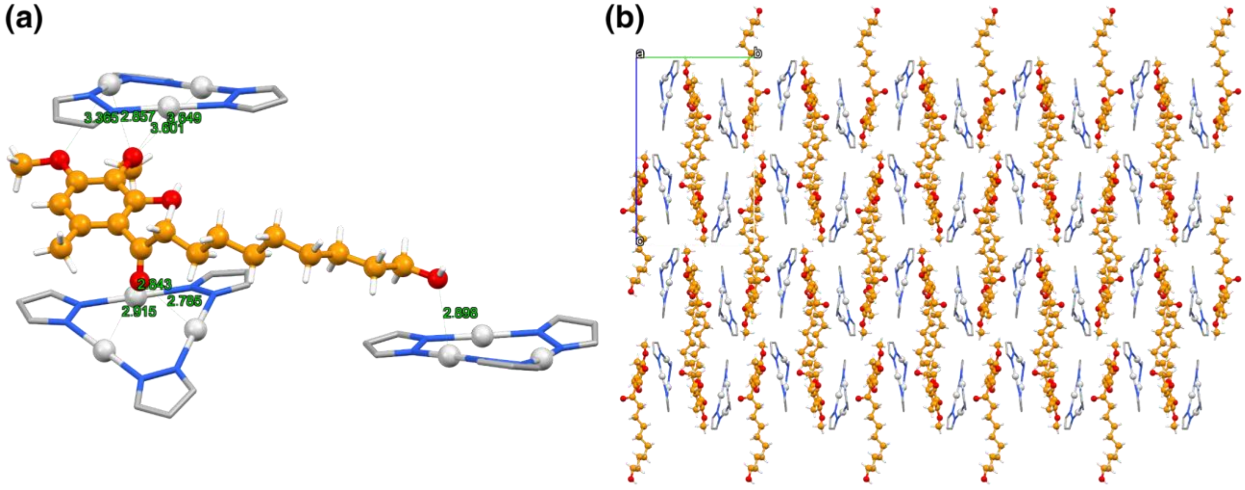

Figure S344. (a) A schematic diagram of the co-crystal structure in the **Ag_3Pz_3·107** single crystal, formed by the guest organic molecule and the surrounding Ag_3Pz_3 units that exhibit significant interactions with it. (b) A 1 × 5 × 2 packing mode in the single crystal structure of **Ag_3Pz_3·107** along the *a* axis. Trifluoromethyl groups and H atoms in Ag_3Pz_3 are omitted for clarity. Ag···O interactions are indicated with green dotted lines with distances in Å. C, N, and Ag atoms in Ag_3Pz_3 are depicted in dark gray, light blue, and light gray, respectively; C, O, and H atoms in **107** are depicted in orange, red, and white, respectively.

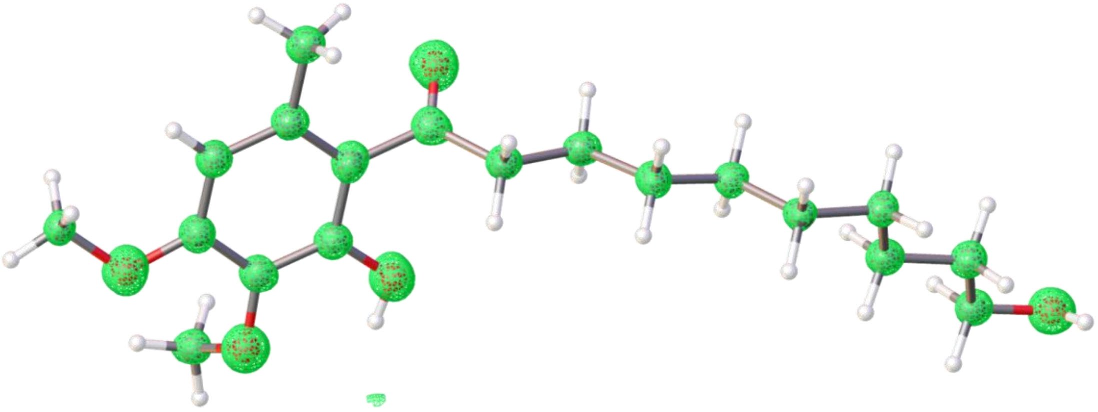

Figure S345. F_{obs} (contour: 1.00) electron density map superimposed on the structure of **107** in the single crystal structure of **Ag_3Pz_3·107**.

Preparation of Ag_3Pz_3·108. 3.41 mg (0.0107 mmol) of zearalenone (**108**) was dissolved in 3 mL of a binary solvent system of DCM and MeOH (1:1, v/v), followed by the addition of equimolar amounts of Ag_3Pz_3 (10.00 mg, 0.0107 mmol). The resulting mixed solution was filtered and then transferred to a 20 mL screw-capped sample vial. The cap of the sample vial was loosely closed to allow the solvent to slowly evaporate at room temperature. The entire co-crystal incubation process was protected from light using aluminum foil. After the designated evaporation period, typically 1-3 days, high-quality colorless block-shaped crystals suitable for single-crystal X-ray diffraction analysis formed at the bottom of the vial.

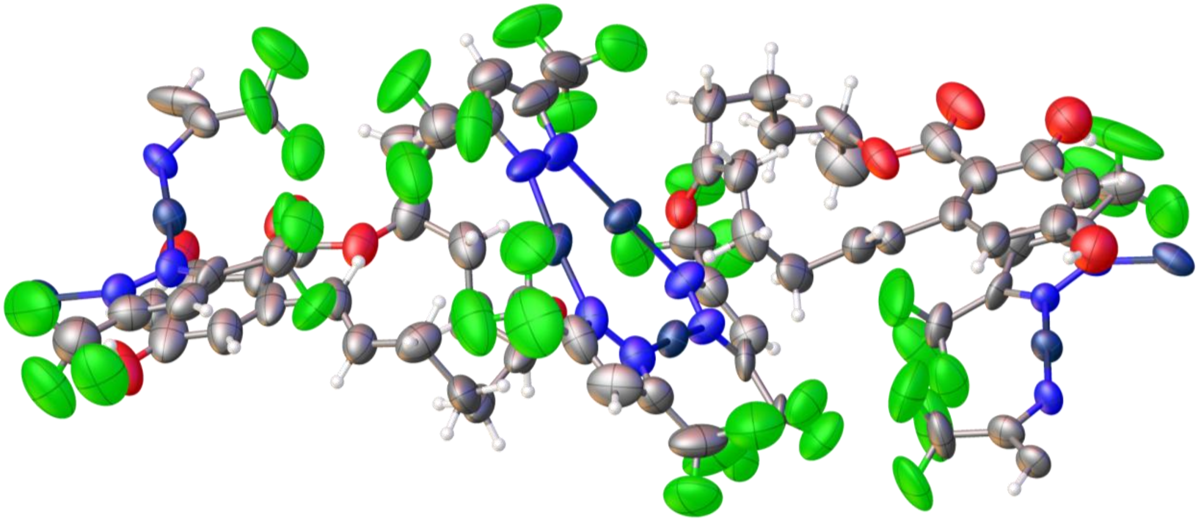

Figure S346. Asymmetric unit of **Ag_3Pz_3·108** (thermal displacement parameters at the 50% probability level).

Table S116. Crystal data and structure refinement for **Ag_3Pz_3·108**

Empirical formula	$C_{33}H_{25}Ag_3F_{18}N_6O_5$
Formula weight	1251.20
Temperature/K	100.2(6)
Crystal system	monoclinic
Space group	*C*2
a/Å	32.3294(8)
b/Å	13.4850(4)
c/Å	21.4483(5)
α/°	90
β/°	99.988(2)
γ/°	90
Volume/Å^3	9208.9(4)
Z	8
ρ_{calc}g/cm^3	1.805
μ/mm^{-1}	11.216
F(000)	4864.0
Crystal size/mm^3	0.15 × 0.15 × 0.14
Radiation	Cu Kα (λ = 1.54184)
2θ range for data collection/°	7.51 to 149.982
Index ranges	$-40 \leq h \leq 39$, $-16 \leq k \leq 12$, $-26 \leq l \leq 24$
Reflections collected	26495
Independent reflections	15114 [R_{int} = 0.0643, R_{sigma} = 0.0609]
Data/restraints/parameters	15114/304/1134
Goodness-of-fit on F^2	1.146
Final *R* indexes [I>=2σ (I)]	R_1 = 0.0820, wR_2 = 0.2380
Final *R* indexes [all data]	R_1 = 0.0904, wR_2 = 0.2499
Largest diff. peak/hole / e Å^{-3}	2.15/-1.71
Flack parameter	0.121(17)
CCDC-number	2501851

Responses to CheckCIF alerts for Ag_3Pz_3·108 crystal structure:

A-level alert:

"Check Calcd Positive Resid. Density on Ag01 2.28 eA-3"

This Alert is due to presence of residual density in the presence of heavy metal atom (Ag).

B-level alerts:

"The absolute value of parameter shift to su ratio > 0.10

Absolute value of the parameter shift to su ratio given 0.150

Additional refinement cycles may be required."

We made several attempts to obtain a higher quality data set for this compound. However, due to solvent disorder and poor crystal quality, we were unable to lower the absolute value of parameter shift. Nevertheless, there is no doubt regarding the framework of the structure.

"Maximum Shift/Error .... 0.15 Why ?"

We have tried to increase the number of fine-tuning rounds to solve this problem, but this is the best result we have achieved so far.

"Large Hirshfeld Difference C01F --C017 . 0.30 Ang."

atoms from a modeled disordered moiety; relevant atomic sites have been checked and they were found to be consistent with the chemistry performed.

"Low Bond Precision on C-C Bonds .... 0.03392 Ang."

Disordered structure.

"D-H Bond Without Acceptor O013 --H013 . Please Check"

The solvent molecules are highly disordered, which supposed to form hydrogen bond with O have been removed by the SQUEEZE routine in the PLATON software package.

"D-H Bond Without Acceptor O01M --H01M . Please Check"

The solvent molecules are highly disordered, which supposed to form hydrogen bond with O have been removed by the SQUEEZE routine in the PLATON software package.

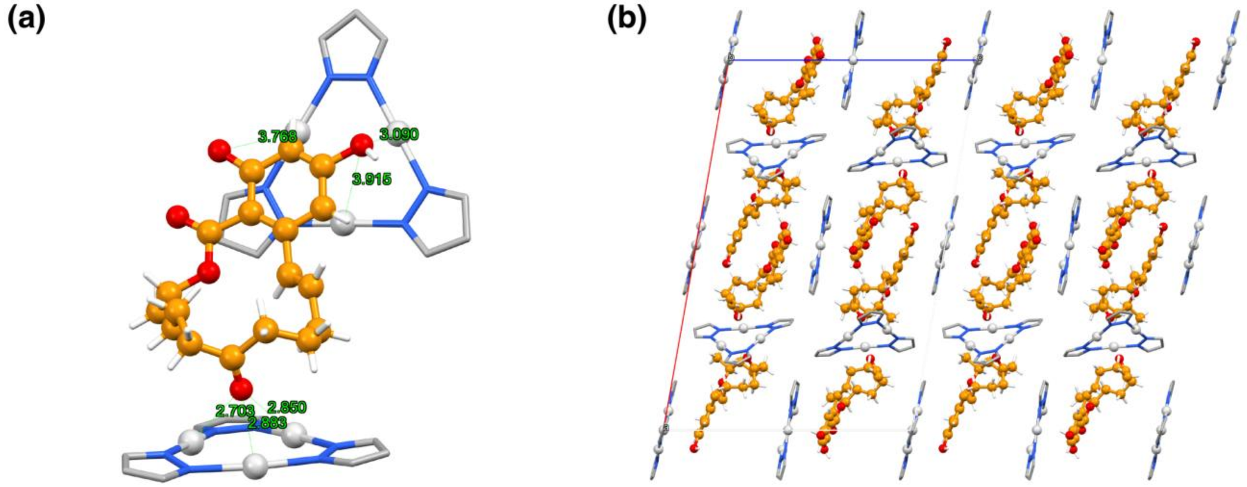

Figure S347. (a) A schematic diagram of the co-crystal structure in the **Ag_3Pz_3·108** single crystal, formed by the guest organic molecule and the surrounding Ag_3Pz_3 units that exhibit significant interactions with it. (b) A 1 × 1 × 2 packing mode in the single crystal structure of **Ag_3Pz_3·108** along the *b* axis. Trifluoromethyl groups and H atoms in Ag_3Pz_3 are omitted for clarity. Ag···O interactions are indicated with green dotted lines with distances in Å. C, N, and Ag atoms in Ag_3Pz_3 are depicted in dark gray, light blue, and light gray, respectively; C, O, and H atoms in **108** are depicted in orange, red, and white, respectively.

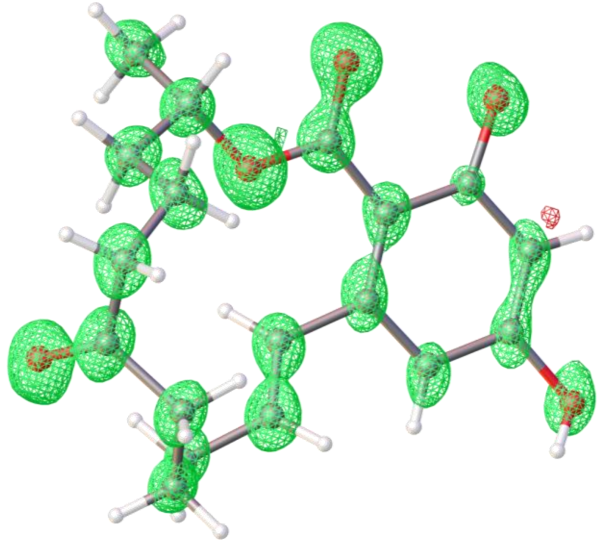

Figure S348. F_{obs} (contour: 0.30) electron density map superimposed on the structure of **108** in the single crystal structure of **Ag_3Pz_3·108**.

Preparation of Ag_3Pz_3·109. 7.45 mg (0.0107 mmol) of rifamycin S (**109**) was dissolved in 3 mL of a binary solvent system of DCM and n-Hex (1:1, v/v), followed by the addition of equimolar amounts of Ag_3Pz_3 (10.00 mg, 0.0107 mmol). The resulting mixed solution was filtered and then transferred to a 20 mL screw-capped sample vial. The cap of the sample vial was loosely closed to allow the solvent to slowly evaporate at room temperature. The entire co-crystal incubation process was protected from light using aluminum foil. After the designated evaporation period, typically 1-3 days, high-quality orange block-shaped crystals suitable for single-crystal X-ray diffraction analysis formed at the bottom of the vial.

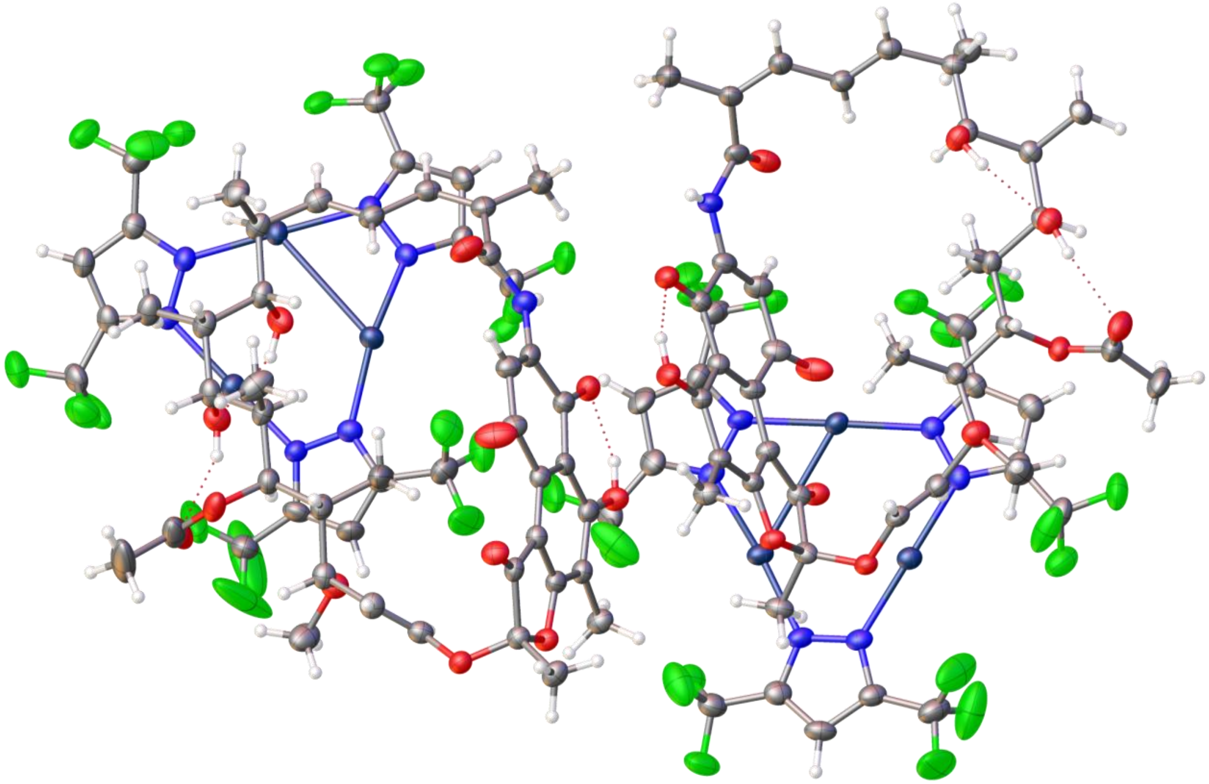

Figure S349. Asymmetric unit of **Ag_3Pz_3·109** (thermal displacement parameters at the 50% probability level).

Table S117. Crystal data and structure refinement for **Ag_3Pz_3·109**

Empirical formula	$C_{52}H_{48}Ag_3F_{18}N_7O_{12}$
Formula weight	1628.58
Temperature/K	100.15
Crystal system	triclinic
Space group	*P*1
a/Å	12.0102(2)
b/Å	12.27470(10)
c/Å	23.4682(3)
α/°	96.2670(10)
β/°	99.3650(10)
γ/°	112.2810(10)
Volume/Å^3	3102.93(7)
Z	2
ρ_{calc}g/cm^3	1.743
μ/mm^{-1}	8.581
F(000)	1616.0
Crystal size/mm^3	0.3 × 0.2 × 0.1
Radiation	Cu Kα (λ = 1.54184)
2θ range for data collection/°	7.774 to 146.184
Index ranges	$-14 \leq h \leq 14$, $-14 \leq k \leq 11$, $-29 \leq l \leq 28$
Reflections collected	35316
Independent reflections	17236 [R_{int} = 0.0349, R_{sigma} = 0.0331]
Data/restraints/parameters	17236/9/1690
Goodness-of-fit on F^2	1.035
Final *R* indexes [*I*>=2σ (*I*)]	R_1 = 0.0356, wR_2 = 0.0940
Final *R* indexes [all data]	R_1 = 0.0363, wR_2 = 0.0946
Largest diff. peak/hole / e Å^{-3}	0.78/-1.27
Flack parameter	-0.017(5)
CCDC-number	2501852

Responses to CheckCIF alert for Ag_3Pz_3·109 crystal structure:

(There is no A-level alert)

B-level alert:

"No Flack x Check Done: Low Friedel Pair Coverage 44 %"

Due to insufficient data. The compound crystallizes in the chiral space group, but no chiral ligand is used, and the complex is no longer chiral.

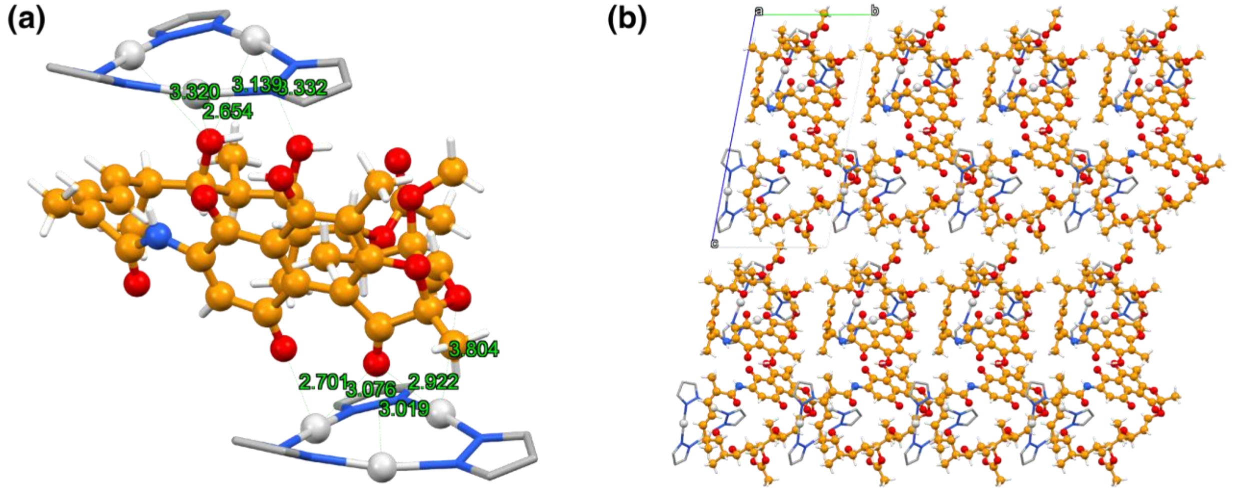

Figure S350. (a) A schematic diagram of the co-crystal structure in the **Ag_3Pz_3·109** single crystal, formed by the guest organic molecule and the surrounding Ag_3Pz_3 units that exhibit significant interactions with it. (b) A 1 × 4 × 2 packing mode in the single crystal structure of **Ag_3Pz_3·109** along the *a* axis. Trifluoromethyl groups and H atoms in Ag_3Pz_3 are omitted for clarity. Ag···O interactions are indicated with green dotted lines with distances in Å. C, N, and Ag atoms in Ag_3Pz_3 are depicted in dark gray, light blue, and light gray, respectively; C, O, N, and H atoms in **109** are depicted in orange, red, light blue, and white, respectively.

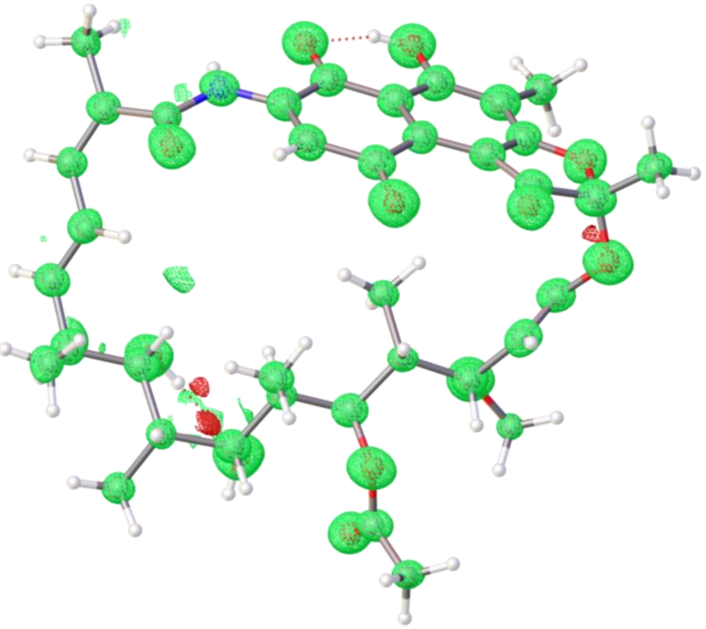

Figure S351. F_{obs} (contour: 0.85) electron density map superimposed on the structure of **109** in the single crystal structure of **Ag_3Pz_3·109**.

Preparation of Ag_3Pz_3·110. 8.07 mg (0.0107 mmol) of rifamycin O (**110**) was dissolved in 3 mL of a binary solvent system of DCM and MeOH (1:1, v/v), followed by the addition of equimolar amounts of Ag_3Pz_3 (10.00 mg, 0.0107 mmol). The resulting mixed solution was filtered and then transferred to a 20 mL screw-capped sample vial. The cap of the sample vial was loosely closed to allow the solvent to slowly evaporate at room temperature. The entire co-crystal incubation process was protected from light using aluminum foil. After the designated evaporation period, typically 1-3 days, high-quality yellow needle-shaped crystals suitable for single-crystal X-ray diffraction analysis formed at the bottom of the vial.

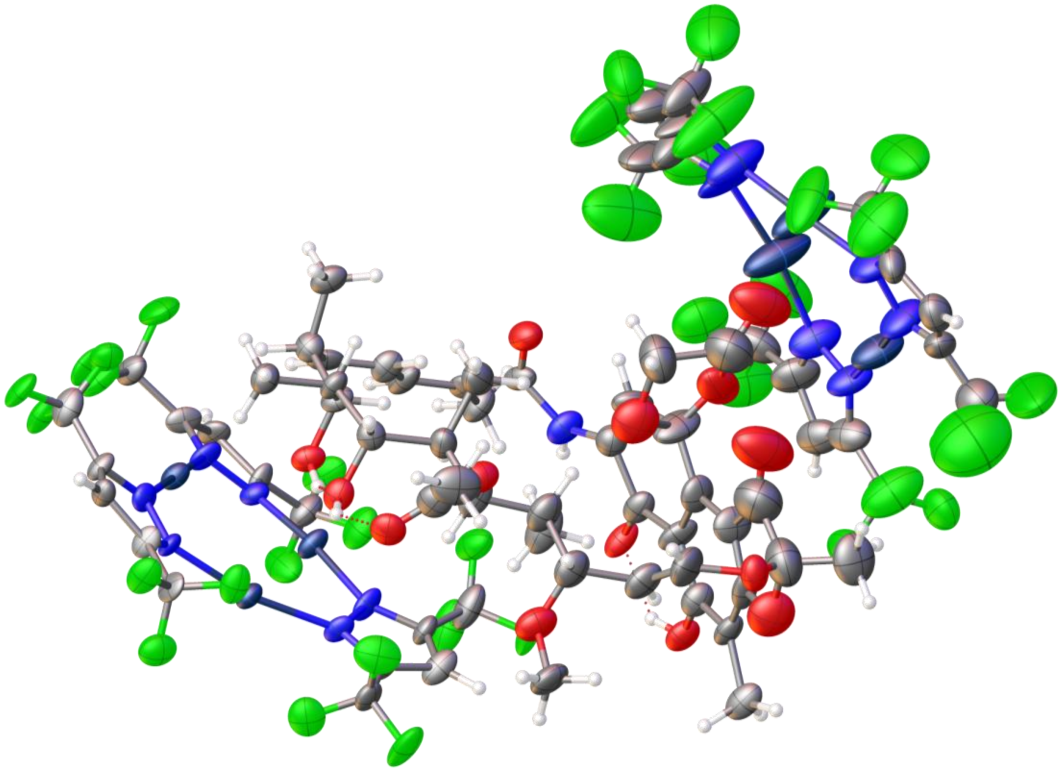

Figure S352. Asymmetric unit of **Ag_3Pz_3·110** (thermal displacement parameters at the 50% probability level).

Table S118. Crystal data and structure refinement for Ag**3Pz3·110**

Empirical formula	$C_{69}H_{53}Ag_6F_{36}N_{13}O_{14}$
Formula weight	2619.46
Temperature/K	100.15
Crystal system	monoclinic
Space group	*C*2
a/Å	23.4213(8)
b/Å	13.5037(3)
c/Å	28.3748(11)
α/°	90
β/°	104.746(4)
γ/°	90
Volume/Å^3	8678.6(5)
Z	4
ρ_{calc}g/cm^3	2.005
μ/mm^{-1}	11.977
F(000)	5104.0
Crystal size/mm^3	0.23 × 0.16 × 0.14
Radiation	Cu Kα (λ = 1.54184)
2θ range for data collection/°	7.622 to 145.312
Index ranges	$-27 \leq h \leq 28, -15 \leq k \leq 16, -34 \leq l \leq 33$
Reflections collected	38441
Independent reflections	14916 [R_{int} = 0.0574, R_{sigma} = 0.0719]
Data/restraints/parameters	14916/39/1231
Goodness-of-fit on F^2	1.042
Final *R* indexes [I>=2σ (I)]	R_1 = 0.0765, wR_2 = 0.1843
Final *R* indexes [all data]	R_1 = 0.0897, wR_2 = 0.1935
Largest diff. peak/hole / e Å^{-3}	1.21/-1.24
Flack parameter	-0.001(8)
CCDC-number	2501853

Responses to CheckCIF alert for Ag_3Pz_3·110 crystal structure:

(There is no A-level alert)

B-level alert:

"Low Bond Precision on C-C Bonds .... 0.03483 Ang."

Disordered structure.

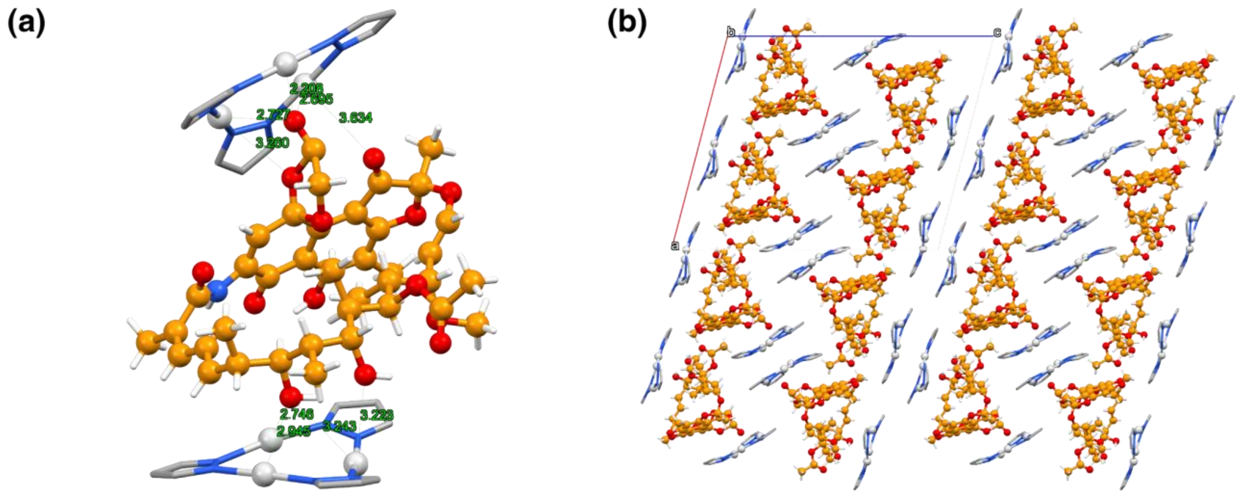

Figure S353. (a) A schematic diagram of the co-crystal structure in the **Ag_3Pz_3·110** single crystal, formed by the guest organic molecule and the surrounding Ag_3Pz_3 units that exhibit significant interactions with it. (b) A 2 × 1 × 2 packing mode in the single crystal structure of **Ag_3Pz_3·110** along the *b* axis. Trifluoromethyl groups and H atoms in Ag_3Pz_3 are omitted for clarity. Ag···O interactions are indicated with green dotted lines with distances in Å. C, N, and Ag atoms in Ag_3Pz_3 are depicted in dark gray, light blue, and light gray, respectively; C, O, N, and H atoms in **110** are depicted in orange, red, light blue, and white, respectively.

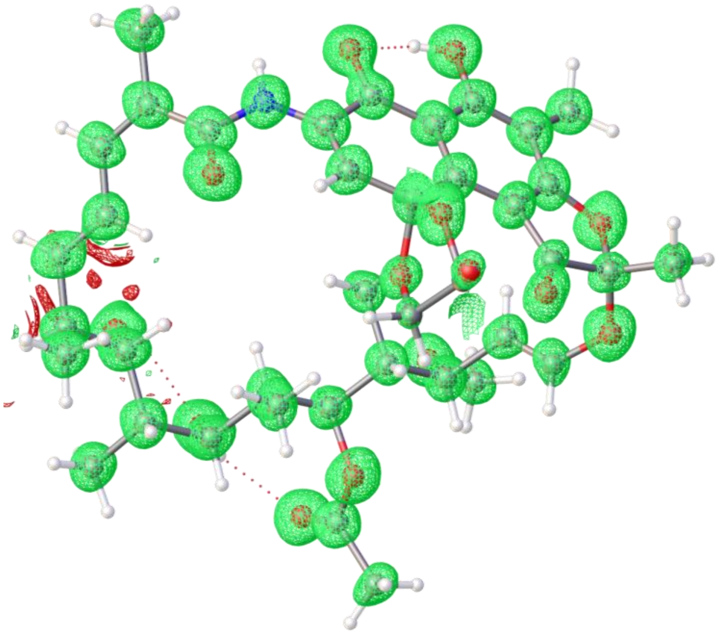

Figure S354. F_{obs} (contour: 0.25) electron density map superimposed on the structure of **110** in the single crystal structure of **Ag_3Pz_3·110**.

Preparation of Ag_3Pz_3·111. 7.85 mg (0.0107 mmol) of erythromycin (**111**) was dissolved in 3 mL of a binary solvent system of DCM and MeOH (1:1, v/v), followed by the addition of equimolar amounts of Ag_3Pz_3 (10.00 mg, 0.0107 mmol). The resulting mixed solution was filtered and then transferred to a 20 mL screw-capped sample vial. The cap of the sample vial was loosely closed to allow the solvent to slowly evaporate at room temperature. The entire co-crystal incubation process was protected from light using aluminum foil. After the designated evaporation period, typically 1-3 days, high-quality colorless needle-shaped crystals suitable for single-crystal X-ray diffraction analysis formed at the bottom of the vial.

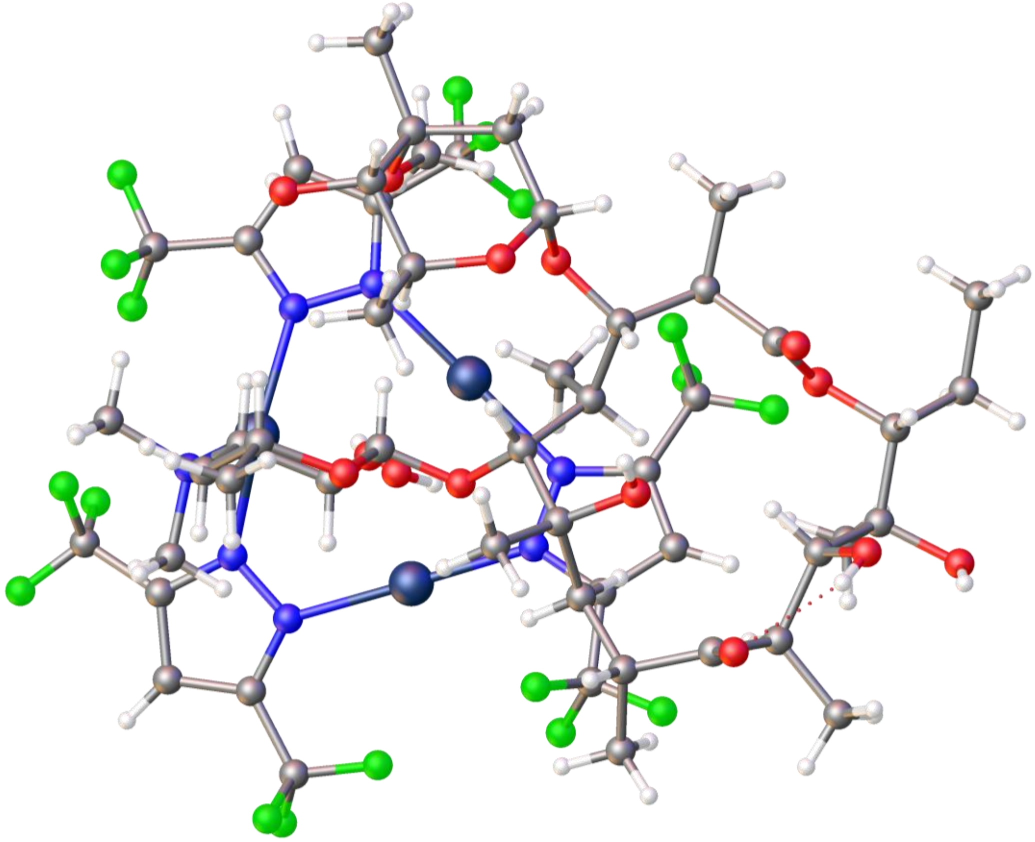

Figure S355. Asymmetric unit of **Ag_3Pz_3·111** (thermal displacement parameters at the 50% probability level).

Table S119. Crystal data and structure refinement for $\mathbf{Ag_3Pz_3{\cdot}111}$

Empirical formula	$C_{52}H_{72}Ag_3F_{18}N_7O_{14}$
Formula weight	1684.77
Temperature/K	100.0(3)
Crystal system	monoclinic
Space group	$P2_1$
a/Å	10.6616(2)
b/Å	12.9514(2)
c/Å	24.6669(4)
α/°	90
β/°	102.471(2)
γ/°	90
Volume/Å^3	3325.71(10)
Z	2
ρ_{calc}g/cm^3	1.682
μ/mm^{-1}	8.044
F(000)	1696.0
Crystal size/mm^3	0.16 × 0.15 × 0.14
Radiation	Cu Kα (λ = 1.54184)
2θ range for data collection/°	7.34 to 156.502
Index ranges	$-10 \le h \le 13$, $-15 \le k \le 16$, $-29 \le l \le 30$
Reflections collected	26075
Independent reflections	12106 [R_{int} = 0.0396, R_{sigma} = 0.0542]
Data/restraints/parameters	12106/1/866
Goodness-of-fit on F^2	1.104
Final R indexes [I>=2σ (I)]	R_1 = 0.0423, wR_2 = 0.1172
Final R indexes [all data]	R_1 = 0.0469, wR_2 = 0.1193
Largest diff. peak/hole / e Å^{-3}	0.71/-0.66
Flack parameter	0.019(9)
CCDC-number	2501854

Responses to CheckCIF alert for Ag_3Pz_3·111 crystal structure:

(There is no A-level alert)

B-level alert:

"D-H Bond Without Acceptor O1 --H1B . Please Check"

The solvent molecules are highly disordered, which supposed to form hydrogen bond with O have been removed by the SQUEEZE routine in the PLATON software package.

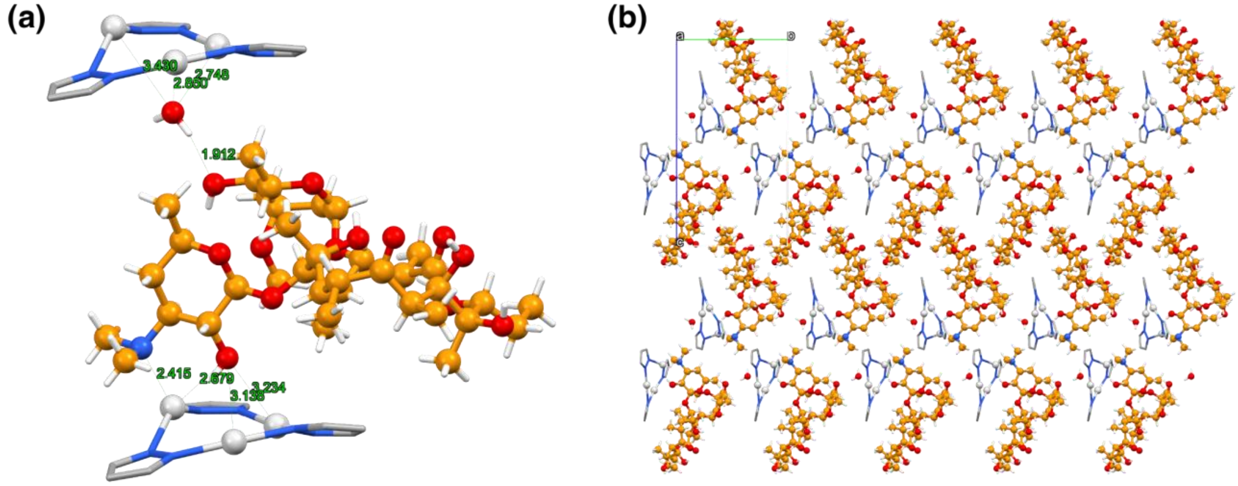

Figure S356. (a) A schematic diagram of the co-crystal structure in the **Ag_3Pz_3·111** single crystal, formed by the guest organic molecule and the surrounding Ag_3Pz_3 units that exhibit significant interactions with it. (b) A 1 × 5 × 2 packing mode in the single crystal structure of **Ag_3Pz_3·111** along the *a* axis. Trifluoromethyl groups and H atoms in Ag_3Pz_3 are omitted for clarity. Ag···O, Ag···N, and O-H···O interactions are indicated with green dotted lines with distances in Å. C, N, and Ag atoms in Ag_3Pz_3 are depicted in dark gray, light blue, and light gray, respectively; C, O, N, and H atoms in **111** are depicted in orange, red, light blue, and white, respectively.

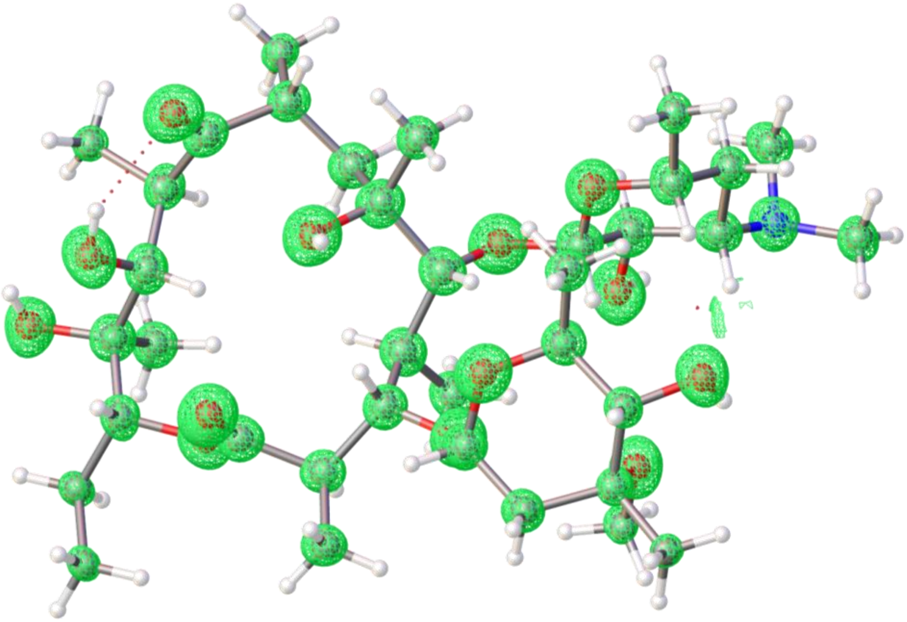

Figure S357. F_{obs} (contour: 1.15) electron density map superimposed on the structure of **111** in the single crystal structure of **Ag_3Pz_3·111**.

Preparation of Ag_3Pz_3·112. 9.22 mg (0.0107 mmol) of erythromycin ethylsuccinate (**112**) was dissolved in 3 mL of a binary solvent system of DCM and n-Hex (1:1, v/v), followed by the addition of equimolar amounts of Ag_3Pz_3 (10.00 mg, 0.0107 mmol). The resulting mixed solution was filtered and then transferred to a 20 mL screw-capped sample vial. The cap of the sample vial was loosely closed to allow the solvent to slowly evaporate at room temperature. The entire co-crystal incubation process was protected from light using aluminum foil. After the designated evaporation period, typically 1-3 days, high-quality colorless block-shaped crystals suitable for single-crystal X-ray diffraction analysis formed at the bottom of the vial.

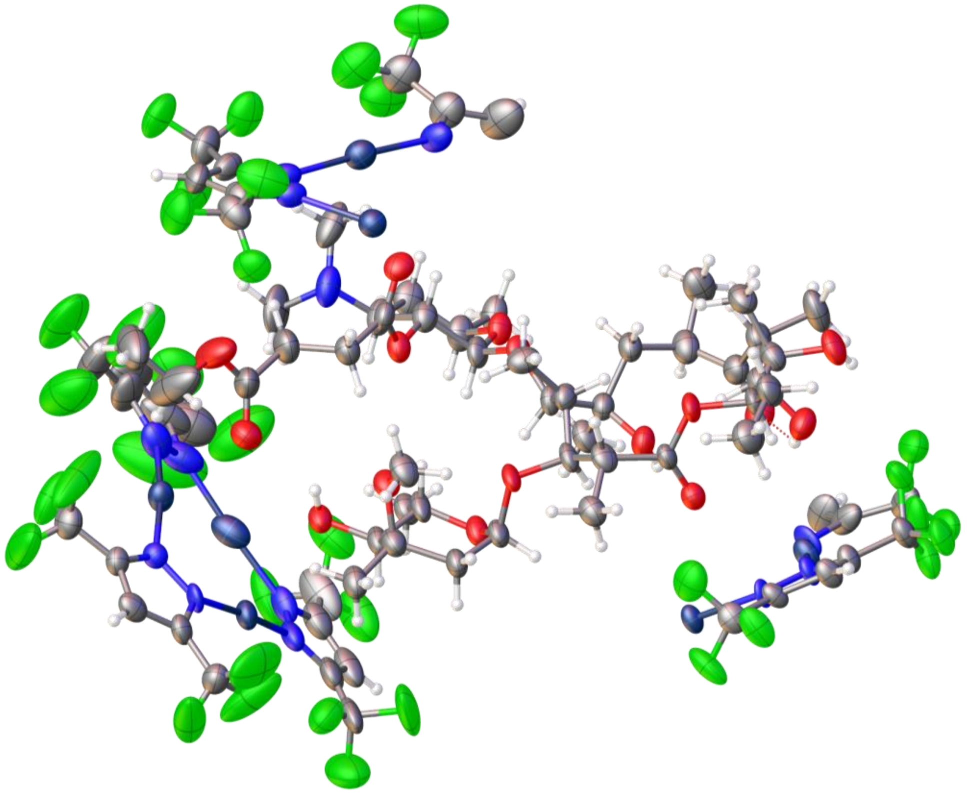

Figure S358. Asymmetric unit of **Ag_3Pz_3·112** (thermal displacement parameters at the 50% probability level).

Table S120. Crystal data and structure refinement for **Ag_3Pz_3·112**

Empirical formula	$C_{73}H_{81}Ag_6F_{36}N_{13}O_{16}$
Formula weight	2727.72
Temperature/K	100.15
Crystal system	monoclinic
Space group	*C*2
a/Å	33.0301(5)
b/Å	12.9921(2)
c/Å	23.7486(4)
α/°	90
β/°	104.652(2)
γ/°	90
Volume/Å^3	9859.8(3)
Z	4
ρ_{calc}g/cm^3	1.838
μ/mm^{-1}	10.583
F(000)	5376.0
Crystal size/mm^3	0.17 × 0.16 × 0.15
Radiation	Cu Kα (λ = 1.54184)
2θ range for data collection/°	5.532 to 156.656
Index ranges	$-41 \le h \le 40$, $-15 \le k \le 15$, $-30 \le l \le 30$
Reflections collected	33993
Independent reflections	17713 [R_{int} = 0.0417, R_{sigma} = 0.0640]
Data/restraints/parameters	17713/86/1317
Goodness-of-fit on F^2	1.075
Final R indexes [I>=2σ (I)]	R_1 = 0.0548, wR_2 = 0.1329
Final R indexes [all data]	R_1 = 0.0642, wR_2 = 0.1382
Largest diff. peak/hole / e Å^{-3}	2.33/-1.86
Flack parameter	-0.013(5)
CCDC-number	2501855

Responses to CheckCIF alert for Ag_3Pz_3·112 crystal structure:

(There is no A-level alert)

B-level alert:

"Low Bond Precision on C-C Bonds .... 0.02076 Ang."

Disordered structure.

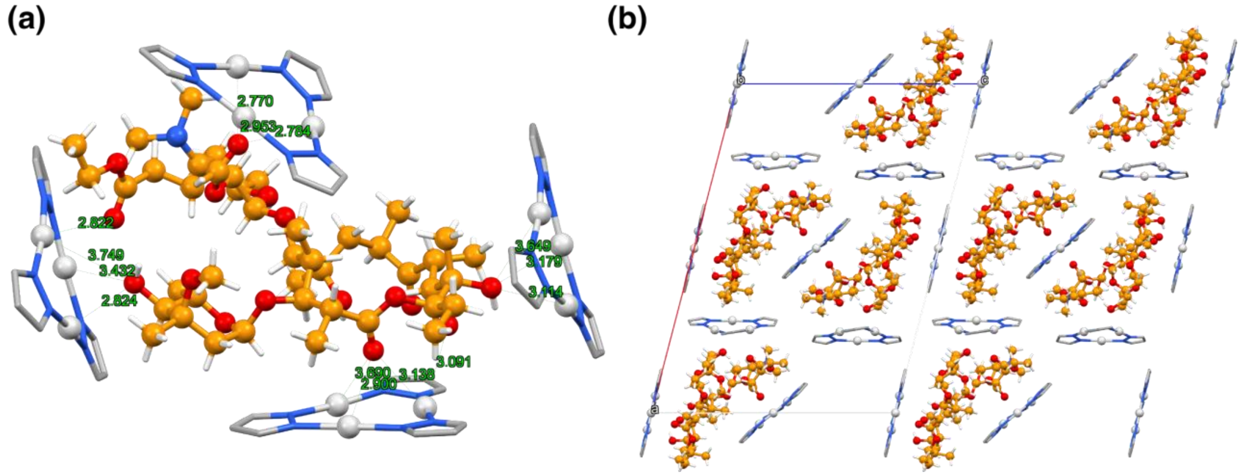

Figure S359. (a) A schematic diagram of the co-crystal structure in the **Ag_3Pz_3·112** single crystal, formed by the guest organic molecule and the surrounding Ag_3Pz_3 units that exhibit significant interactions with it. (b) A 1 × 1 × 2 packing mode in the single crystal structure of **Ag_3Pz_3·112** along the *b* axis. Trifluoromethyl groups and H atoms in Ag_3Pz_3 are omitted for clarity. Ag···O interactions are indicated with green dotted lines with distances in Å. C, N, and Ag atoms in Ag_3Pz_3 are depicted in dark gray, light blue, and light gray, respectively; C, O, N, and H atoms in **112** are depicted in orange, red, light blue, and white, respectively.

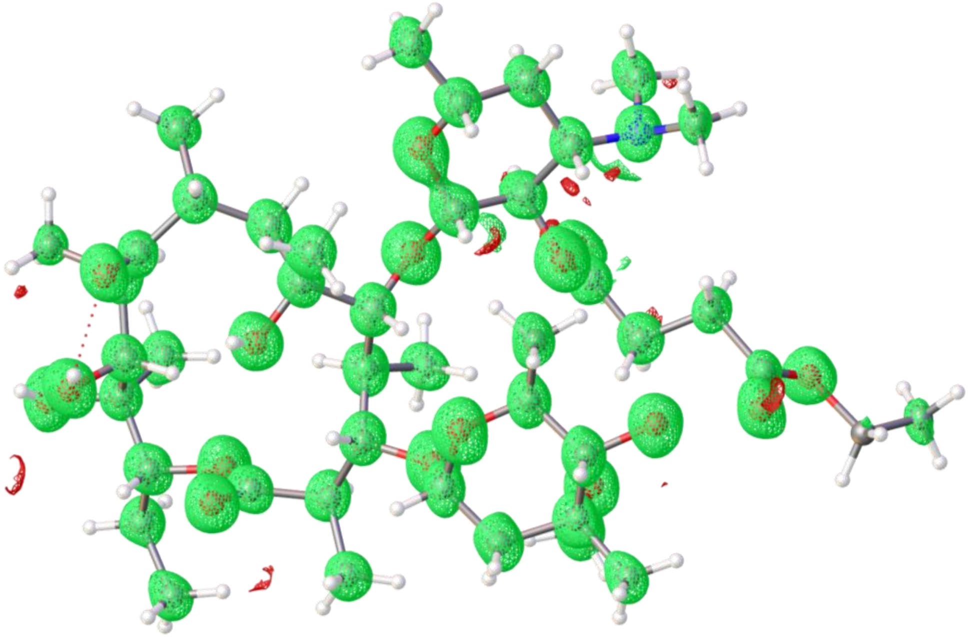

Figure S360. F_{obs} (contour: 0.30) electron density map superimposed on the structure of **112** in the single crystal structure of **Ag_3Pz_3·112**.

Preparation of Ag_3Pz_3·113. 9.36 mg (0.0107 mmol) of ivermectin B1a (**113**) was dissolved in 3 mL of a binary solvent system of DCM and c-Hex (1:1, v/v), followed by the addition of equimolar amounts of Ag_3Pz_3 (10.00 mg, 0.0107 mmol). The resulting mixed solution was filtered and then transferred to a 20 mL screw-capped sample vial. The cap of the sample vial was loosely closed to allow the solvent to slowly evaporate at room temperature. The entire co-crystal incubation process was protected from light using aluminum foil. After the designated evaporation period, typically 1-3 days, high-quality colorless block-shaped crystals suitable for single-crystal X-ray diffraction analysis formed at the bottom of the vial.

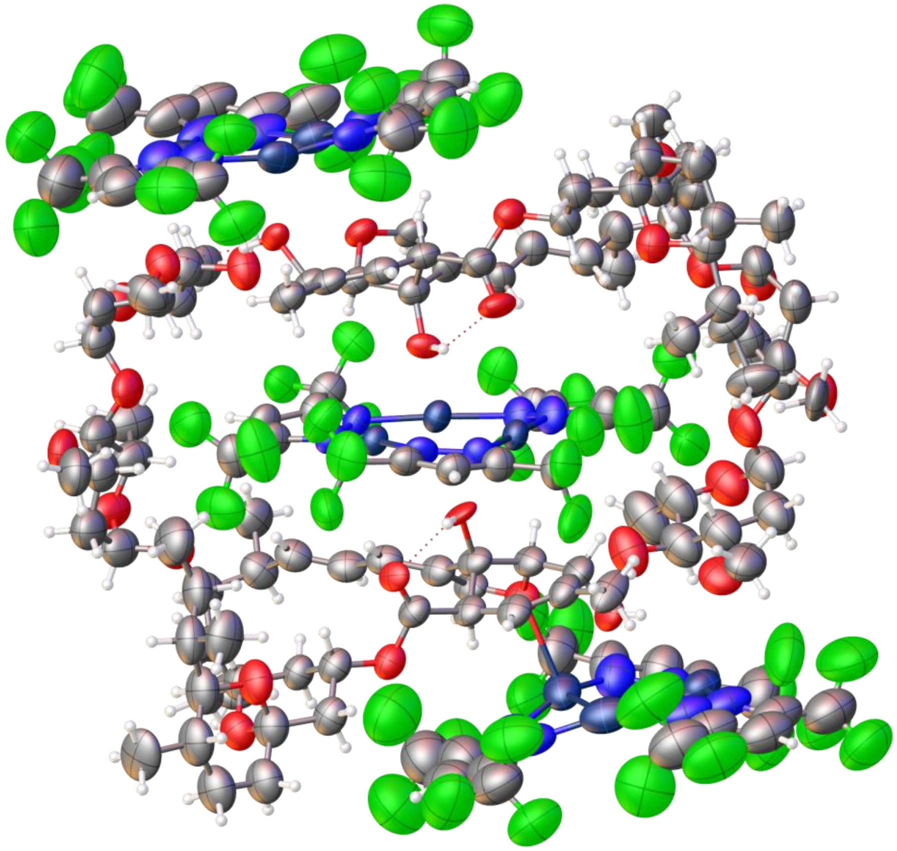

Figure S361. Asymmetric unit of **Ag_3Pz_3·113** (thermal displacement parameters at the 50% probability level).

Table S121. Crystal data and structure refinement for **Ag_3Pz_3·113**

Empirical formula	$C_{141}H_{153}Ag_9F_{54}N_{18}O_{28}$
Formula weight	4544.63
Temperature/K	100.15
Crystal system	monoclinic
Space group	$P2_1$
a/Å	13.6323(4)
b/Å	32.4016(9)
c/Å	24.8156(6)
α/°	90
β/°	95.785(2)
γ/°	90
Volume/Å^3	10905.4(5)
Z	2
ρ_{calc}g/cm^3	1.384
μ/mm^{-1}	7.246
F(000)	4516.0
Crystal size/mm^3	0.17 × 0.16 × 0.14
Radiation	Cu Kα (λ = 1.54184)
2θ range for data collection/°	5.454 to 156.274
Index ranges	$-17 \leq h \leq 16$, $-39 \leq k \leq 32$, $-31 \leq l \leq 29$
Reflections collected	67332
Independent reflections	36007 [R_{int} = 0.0777, R_{sigma} = 0.1471]
Data/restraints/parameters	36007/4414/2196
Goodness-of-fit on F^2	0.940
Final R indexes [I>=2σ (I)]	R_1 = 0.0815, wR_2 = 0.2056
Final R indexes [all data]	R_1 = 0.1459, wR_2 = 0.2394
Largest diff. peak/hole / e Å^{-3}	0.62/-0.67
Flack parameter	0.162(10)
CCDC-number	2501856

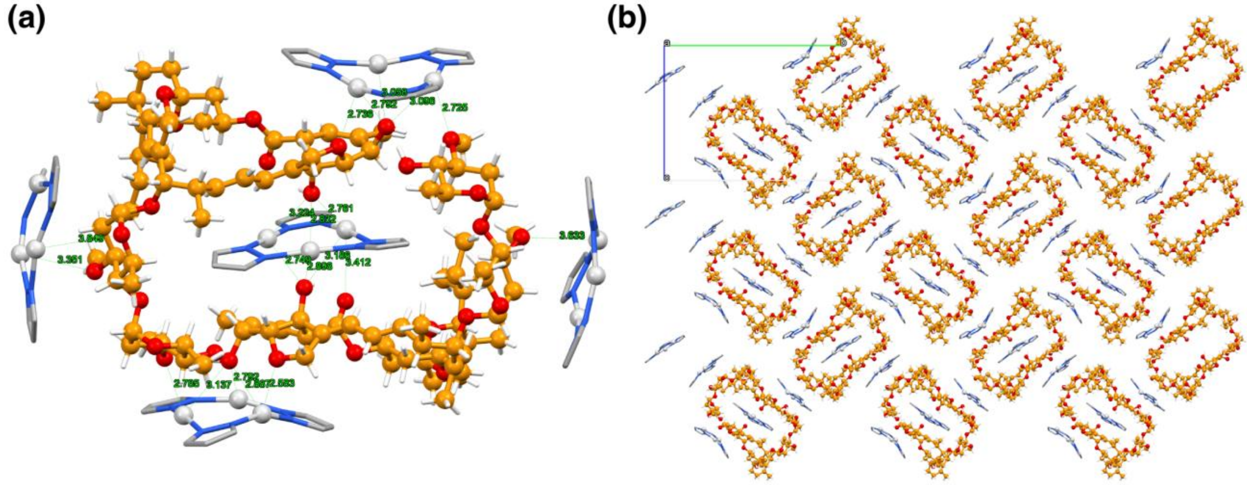

Figure S362. (a) A schematic diagram of the co-crystal structure in the **Ag_3Pz_3·113** single crystal, formed by the guest organic molecule and the surrounding Ag_3Pz_3 units that exhibit significant interactions with it. (b) A 1 × 3 × 3 packing mode in the single crystal structure of **Ag_3Pz_3·113** along the *a* axis. Trifluoromethyl groups and H atoms in Ag_3Pz_3 are omitted for clarity. Ag···O interactions are indicated with green dotted lines with distances in Å. C, N, and Ag atoms in Ag_3Pz_3 are depicted in dark gray, light blue, and light gray, respectively; C, O, N, and H atoms in **113** are depicted in orange, red, light blue, and white, respectively.

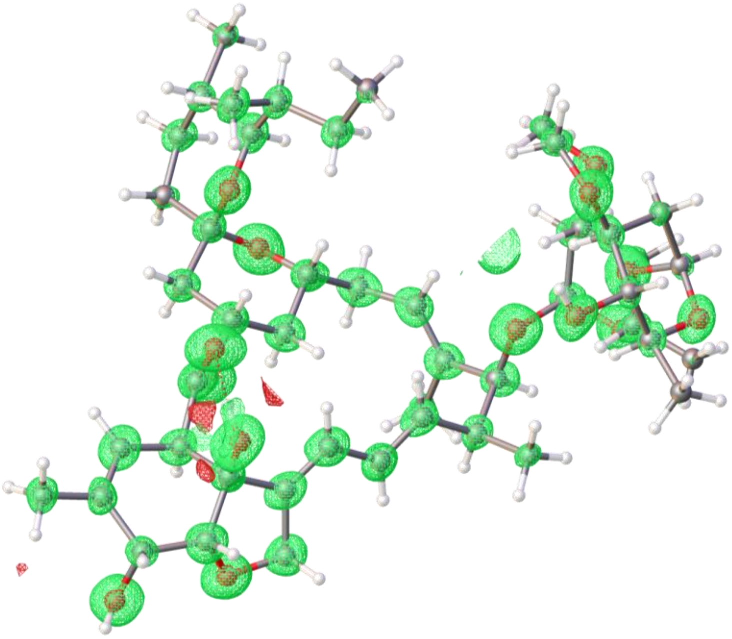

Figure S363. F_{obs} (contour: 0.35) electron density map superimposed on the structure of **113** in the single crystal structure of **Ag_3Pz_3·113**.

Preparation of Ag_3Pz_3·114. 9.62 mg (0.0107 mmol) of Doramectin (**114**) was dissolved in 3 mL of a binary solvent system of DCM and n-Hex (1:1, v/v), followed by the addition of equimolar amounts of Ag_3Pz_3 (10.00 mg, 0.0107 mmol). The resulting mixed solution was filtered and then transferred to a 20 mL screw-capped sample vial. The cap of the sample vial was loosely closed to allow the solvent to slowly evaporate at room temperature. The entire co-crystal incubation process was protected from light using aluminum foil. After the designated evaporation period, typically 1-3 days, high-quality colorless block-shaped crystals suitable for single-crystal X-ray diffraction analysis formed at the bottom of the vial.

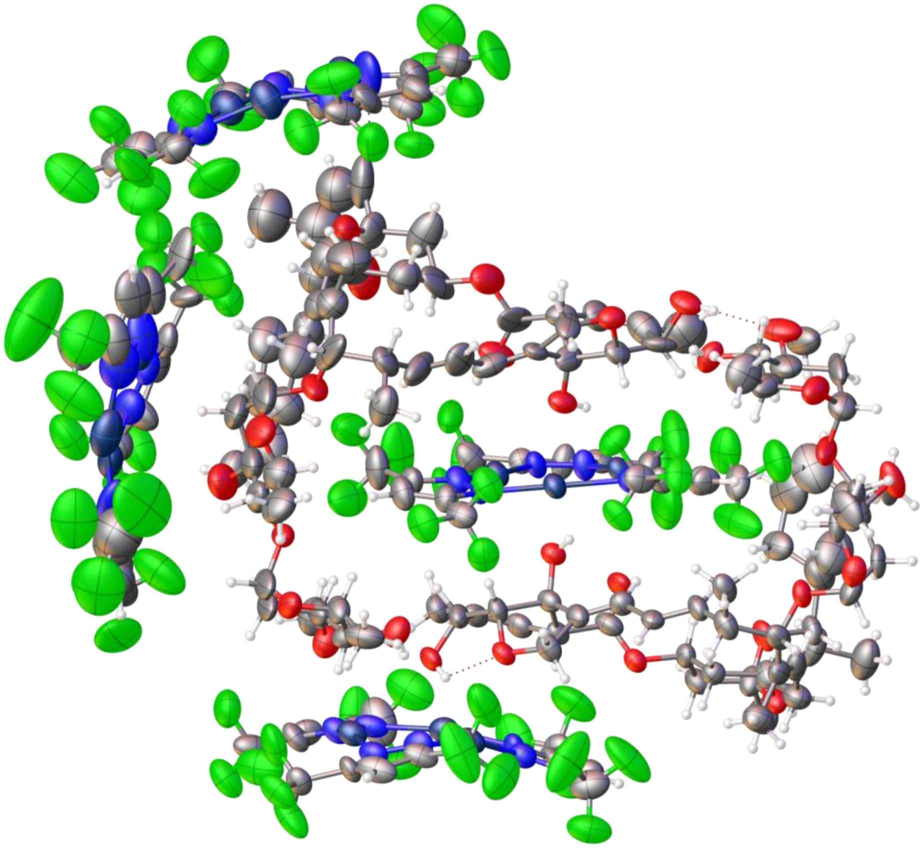

Figure S364. Asymmetric unit of **Ag_3Pz_3·114** (thermal displacement parameters at the 50% probability level).

Table S122. Crystal data and structure refinement for **Ag_3Pz_3·114**

Empirical formula	$C_{80}H_{80}Ag_6F_{36}N_{12}O_{14}$
Formula weight	2764.78
Temperature/K	100.15
Crystal system	monoclinic
Space group	$P2_1$
a/Å	13.5064(2)
b/Å	31.9763(5)
c/Å	24.8145(4)
α/°	90
β/°	94.6340(10)
γ/°	90
Volume/Å^3	10682.0(3)
Z	4
ρ_{calc}g/cm^3	1.719
μ/mm^{-1}	9.762
F(000)	5448.0
Crystal size/mm^3	0.26 × 0.24 × 0.21
Radiation	Cu Kα (λ = 1.54184)
2θ range for data collection/°	6.566 to 155.914
Index ranges	$-15 \le h \le 17$, $-40 \le k \le 32$, $-30 \le l \le 28$
Reflections collected	61019
Independent reflections	33573 [R_{int} = 0.0401, R_{sigma} = 0.0619]
Data/restraints/parameters	33573/733/2579
Goodness-of-fit on F^2	1.021
Final R indexes [I>=2σ (I)]	R_1 = 0.0896, wR_2 = 0.2443
Final R indexes [all data]	R_1 = 0.1016, wR_2 = 0.2595
Largest diff. peak/hole / e Å^{-3}	1.83/-1.86
Flack parameter	0.121(11)
CCDC-number	2501857

Responses to CheckCIF alerts for Ag_3Pz_3·114 crystal structure:

(There is no A-level alert)

B-level alerts:

"Low Bond Precision on C-C Bonds .... 0.03563 Ang."

Disordered structure.

"Short Intra XH3 .. XHn H11B ..H63C . 1.76 Ang.

x,y,z = 1_555 Check"

This Alert is due to close contacts between protons of highly disordered terminal methyl groups with high thermal parameters.

"D-H Bond Without Acceptor O00M --H00M . Please Check"

The solvent molecules are highly disordered, which supposed to form hydrogen bond with O have been removed by the SQUEEZE routine in the PLATON software package.

"Coordinates do not Form a Properly Connected Set Please Do !"

The alert is due to a large number of co-crystallized molecules in the which sometimes do not show as a connected set. This is acceptable from a crystallographic point of view.

"Check Calcd Positive Resid. Density on Ag01 1.74 eA-3"

This Alert is due to presence of residual density in the presence of heavy metal atom (Ag).

"Check Calcd Positive Resid. Density on Ag02 1.61 eA-3"

This Alert is due to presence of residual density in the presence of heavy metal atom (Ag).

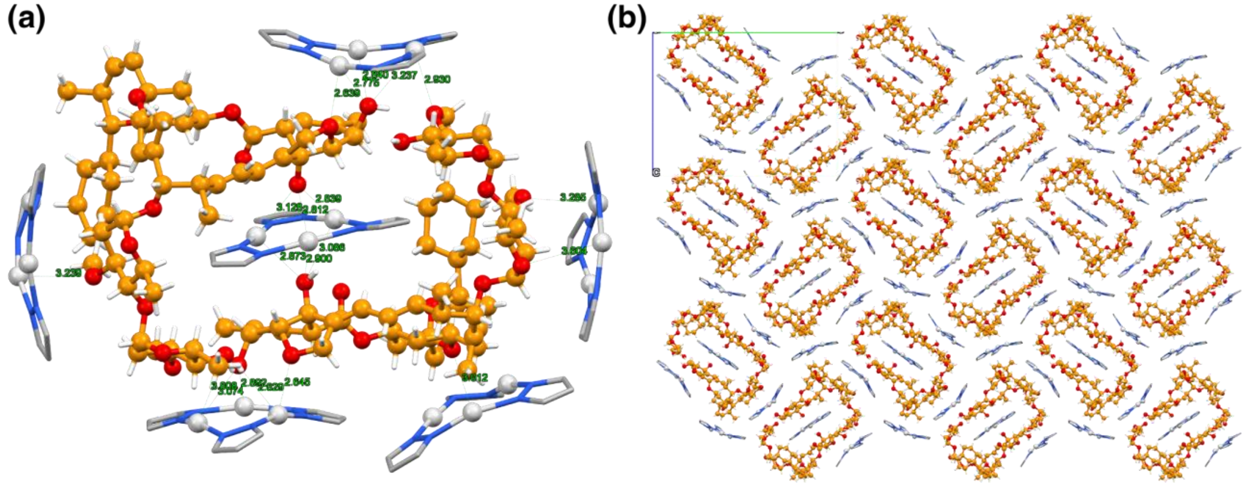

Figure S365. (a) A schematic diagram of the co-crystal structure in the **Ag_3Pz_3·114** single crystal, formed by the guest organic molecule and the surrounding Ag_3Pz_3 units that exhibit significant interactions with it. (b) A 1 × 3 × 3 packing mode in the single crystal structure of **Ag_3Pz_3·114** along the *a* axis. Trifluoromethyl groups and H atoms in Ag_3Pz_3 are omitted for clarity. Ag···O interactions are indicated with green dotted lines with distances in Å. C, N, and Ag atoms in Ag_3Pz_3 are depicted in dark gray, light blue, and light gray, respectively; C, O, and H atoms in **114** are depicted in orange, red, and white, respectively.

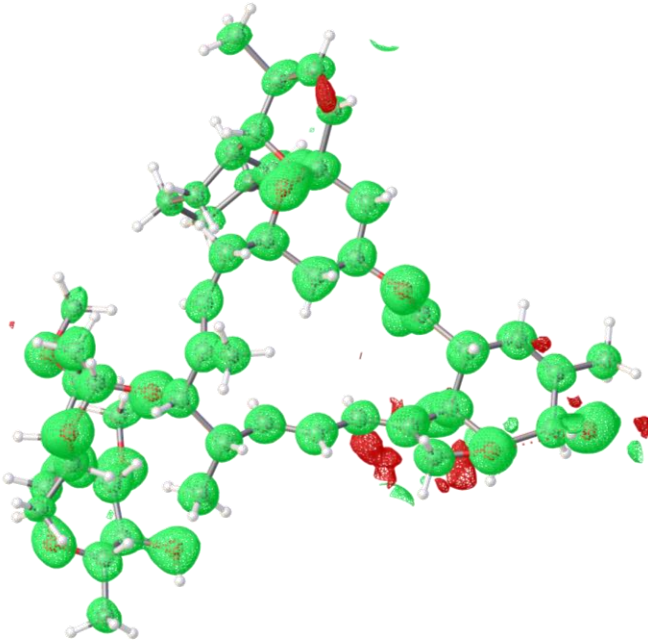

Figure S366. F_{obs} (contour: 0.26) electron density map superimposed on the structure of **114** in the single crystal structure of **Ag_3Pz_3·114**.

One-pot co-crystallization of Ag_3Pz_3. 1.07 mg (0.00535 mmol) of phenothiazine (**87**) and 1.81 mg (0.00535 mmol) of idebenone (**103**) were dissolved in 3 mL of a binary solvent system of n-Hex (1:1, v/v), followed by the addition of equimolar amounts of Ag_3Pz_3 (10.00 mg, 0.0107 mmol). The resulting mixed solution was filtered and then transferred to a 20 mL screw-capped sample vial. The cap of the sample vial was loosely closed to allow the solvent to slowly evaporate at room temperature. The entire co-crystal incubation process was protected from light using aluminum foil. After the designated evaporation period, typically 1-3 days, crystals of various shapes and colors-including orange block-shaped, yellow block-shaped, yellow rod-shaped, and colorless needle-shaped forms-were obtained at the bottom of the vial, some of which were suitable for single-crystal X-ray diffraction analysis.

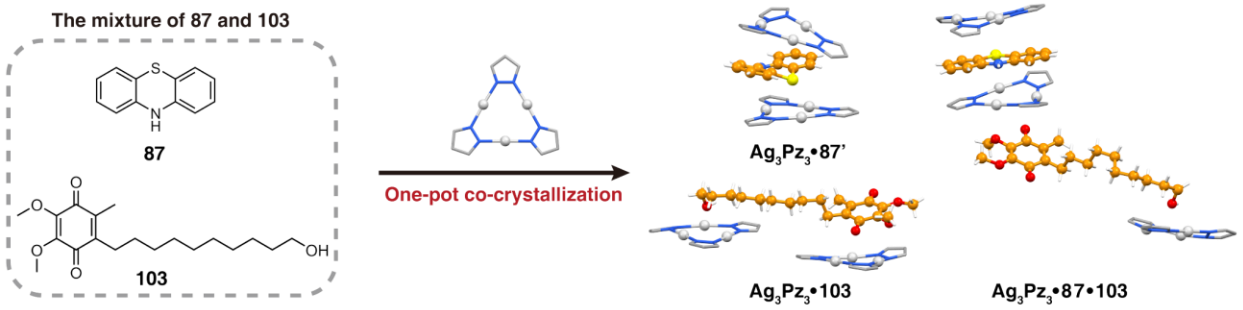

Figure S367. One-pot co-crystallization of Ag_3Pz_3. A mixture of two compounds with distinct chemical structures (compounds **87** and **103**) in an equimolar ratio was used for the co-crystallization experiment.

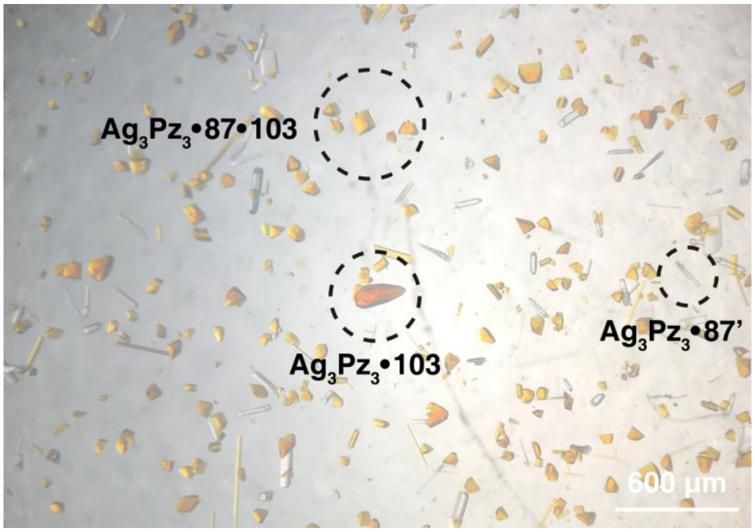

Figure S368. Optical microscopy image of the one-pot co-crystallization products: orange blocks—**Ag_3Pz_3·103**; colorless transparent rods—**Ag_3Pz_3·87’**; yellow blocks—**Ag_3Pz_3·87·103** ternary co-crystal.

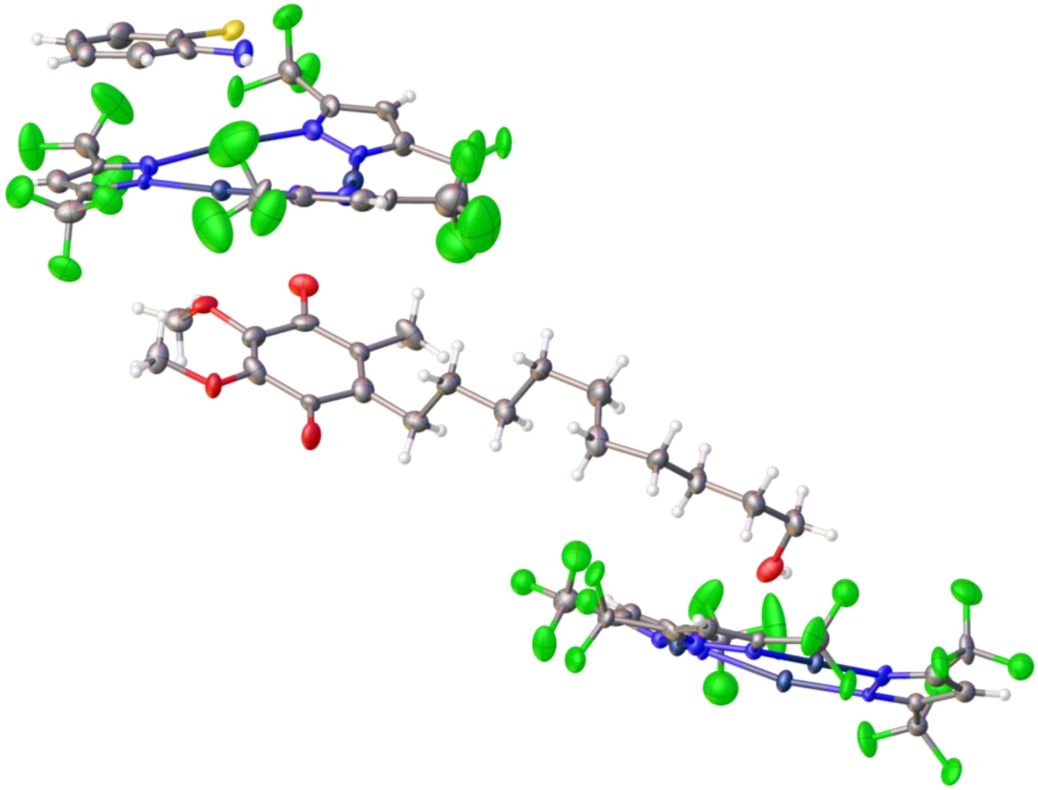

Figure S369. Asymmetric unit of **Ag_3Pz_3·87·103** (thermal displacement parameters at the 50% probability level).

Table S123. Crystal data and structure refinement for **Ag_3Pz_3·87·103**

Empirical formula	$C_{55}H_{40.5}Ag_6F_{36}N_{12.5}O_5S_{0.5}$
Formula weight	2303.75
Temperature/K	100.01(11)
Crystal system	orthorhombic
Space group	$C222_1$
a/Å	13.02260(10)
b/Å	22.6240(2)
c/Å	48.3112(5)
α/°	90
β/°	90
γ/°	90
Volume/Å^3	14233.6(2)
Z	8
ρ_{calc}g/cm^3	2.150
μ/mm^{-1}	14.516
F(000)	8896.0
Crystal size/mm^3	0.15 × 0.13 × 0.12
Radiation	Cu Kα (λ = 1.54184)
2θ range for data collection/°	7.834 to 155.404
Index ranges	$-16 \le h \le 10$, $-26 \le k \le 28$, $-60 \le l \le 60$
Reflections collected	23391
Independent reflections	13243 [R_{int} = 0.0542, R_{sigma} = 0.0621]
Data/restraints/parameters	13243/357/976
Goodness-of-fit on F^2	1.195
Final R indexes [I>=2σ (I)]	R_1 = 0.0914, wR_2 = 0.2177
Final R indexes [all data]	R_1 = 0.0969, wR_2 = 0.2202
Largest diff. peak/hole / e Å^{-3}	1.58/-1.27
Flack parameter	0.08(3)
CCDC-number	2501876

Responses to CheckCIF alert for Ag_3Pz_3·87·103 crystal structure:

(There is no A-level alert)

B-level alert:

"Low Bond Precision on C-C Bonds .... 0.03255 Ang."

Disordered structure.

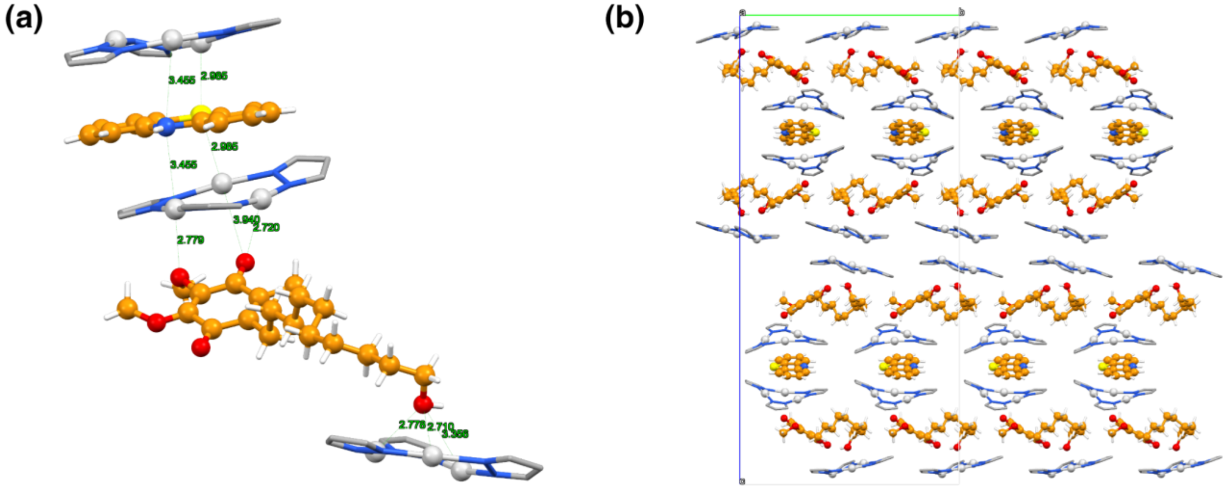

Figure S370. (a) A schematic diagram of the co-crystal structure in the **Ag_3Pz_3·87·103** single crystal, formed by the guest organic molecules and the surrounding Ag_3Pz_3 units that exhibit significant interactions with it. (b) A 1 × 2 × 1 packing mode in the single crystal structure of **Ag_3Pz_3·87·103** along the *a* axis. Trifluoromethyl groups and H atoms in Ag_3Pz_3 are omitted for clarity. Ag···O, Ag···S, and Ag···N interactions are indicated with green dotted lines with distances in Å. C, N, and Ag atoms in Ag_3Pz_3 are depicted in dark gray, light blue, and light gray, respectively; C, S, N, and H atoms in **87** are depicted in orange, yellow, light blue, and white, respectively. C, O, and H atoms in **103** are depicted in orange, red, and white, respectively.

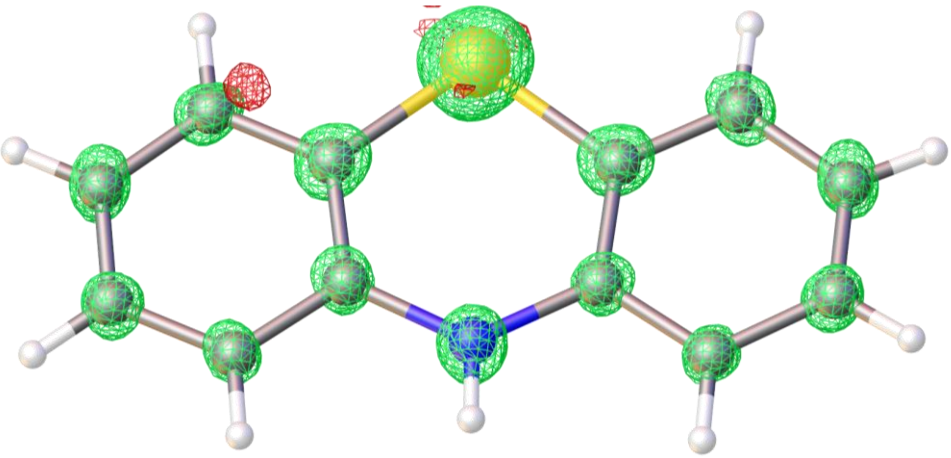

Figure S371. F_{obs} (contour: 0.25) electron density map superimposed on the structure of **87** in the single crystal structure of **Ag_3Pz_3·87·103**.

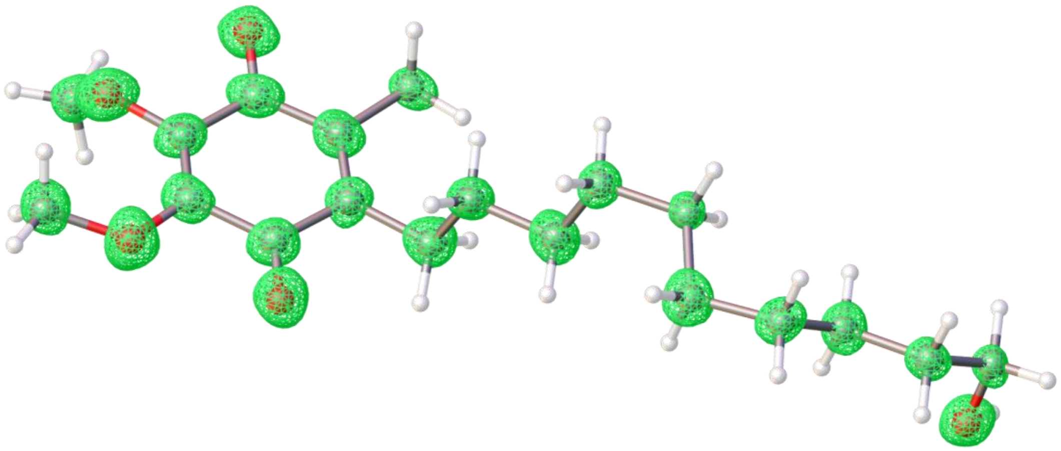

Figure S372. F_{obs} (contour: 0.25) electron density map superimposed on the structure of **103** in the single crystal structure of **Ag$_3$Pz$_3$·87·103**.

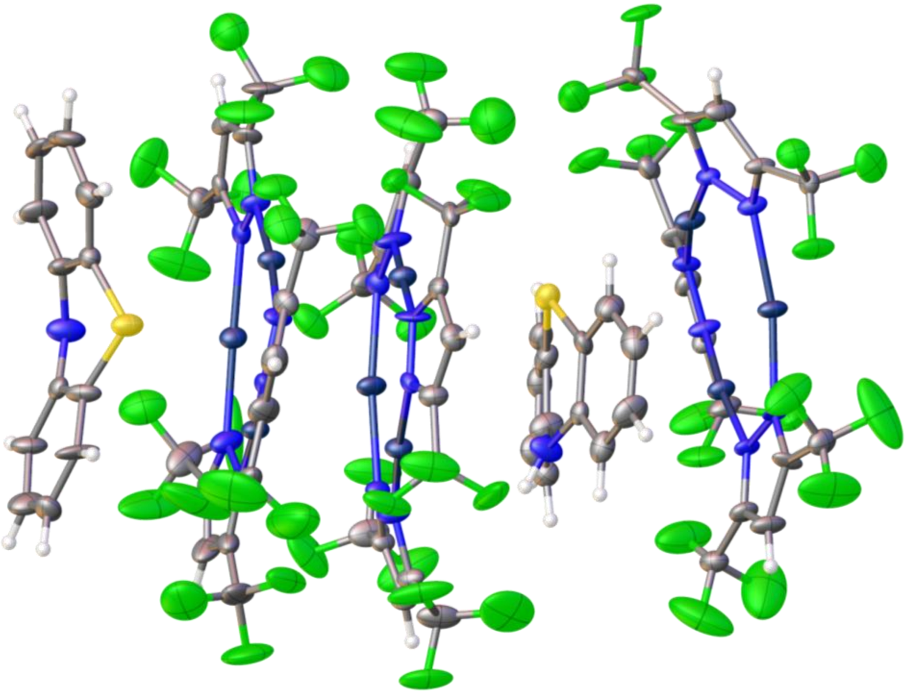

Figure S373. Asymmetric unit of **Ag$_3$Pz$_3$·87'** (thermal displacement parameters at the 50% probability level).

Table S124. Crystal data and structure refinement for **Ag_3Pz_3·87'**

Empirical formula	$C_{69}H_{27}Ag_9F_{54}N_{20}S_2$
Formula weight	3197.05
Temperature/K	100.01(18)
Crystal system	monoclinic
Space group	$P2_1$
a/Å	12.7861(2)
b/Å	30.3212(4)
c/Å	12.9477(2)
α/°	90
β/°	117.261(2)
γ/°	90
Volume/Å^3	4462.15(13)
Z	2
ρ_{calc}g/cm^3	2.379
μ/mm^{-1}	17.494
F(000)	3044.0
Crystal size/mm^3	0.15 × 0.15 × 0.13
Radiation	Cu Kα (λ = 1.54184)
2θ range for data collection/°	5.83 to 155.496
Index ranges	$-16 \leq h \leq 12$, $-25 \leq k \leq 36$, $-16 \leq l \leq 15$
Reflections collected	28959
Independent reflections	13408 [R_{int} = 0.0515, R_{sigma} = 0.0564]
Data/restraints/parameters	13408/200/1388
Goodness-of-fit on F^2	1.095
Final R indexes [I>=2σ (I)]	R_1 = 0.0581, wR_2 = 0.1323
Final R indexes [all data]	R_1 = 0.0667, wR_2 = 0.1349
Largest diff. peak/hole / e Å^{-3}	1.63/-1.22
Flack parameter	-0.009(14)
CCDC-number	2501874

Responses to CheckCIF alerts for Ag_3Pz_3·87' crystal structure:

(There is no A-level alert)

B-level alerts:

"Low Bond Precision on C-C Bonds .... 0.02917 Ang."

Disordered structure.

"No Flack x Check Done: Low Friedel Pair Coverage 44 %"

Due to insufficient data. The compound crystallizes in the chiral space group, but no chiral ligand is used, and the complex is no longer chiral.

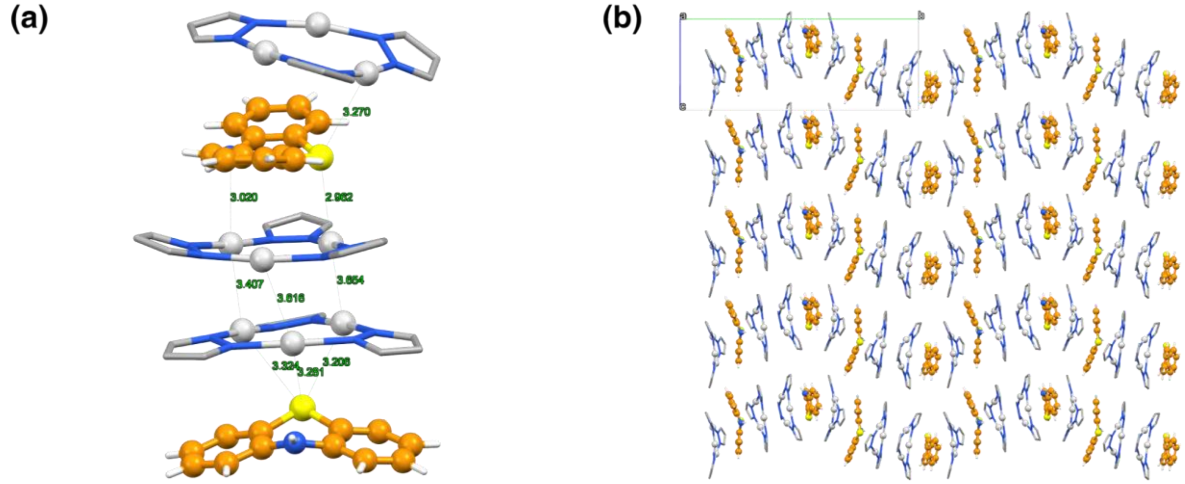

Figure S374. (a) A schematic diagram of the co-crystal structure in the **Ag_3Pz_3·87’** single crystal, formed by the guest organic molecules and the surrounding Ag_3Pz_3 units that exhibit significant interactions with it. (b) A 1 × 2 × 5 packing mode in the single crystal structure of **Ag_3Pz_3·87’** along the *a* axis. Trifluoromethyl groups and H atoms in Ag_3Pz_3 are omitted for clarity. Ag···Ag, Ag···S, and Ag···N interactions are indicated with green dotted lines with distances in Å. C, N, and Ag atoms in Ag_3Pz_3 are depicted in dark gray, light blue, and light gray, respectively; C, S, N, and H atoms in **87** are depicted in orange, yellow, light blue, and white, respectively.

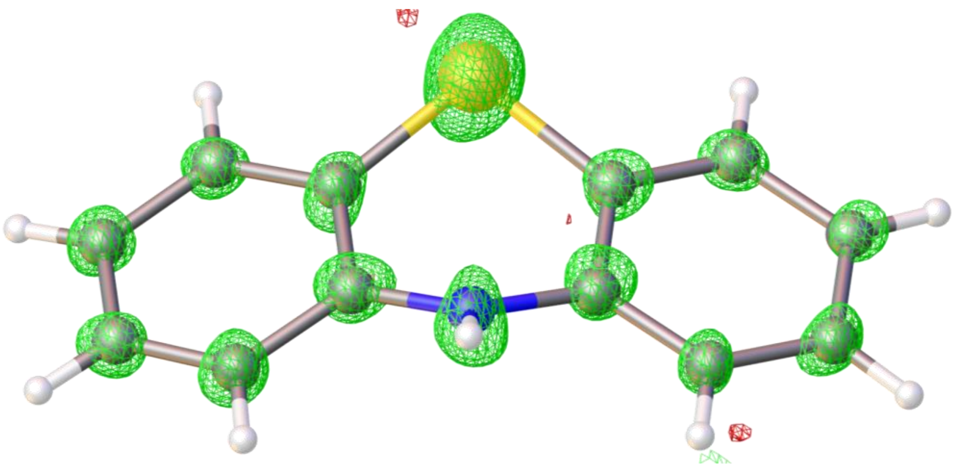

Figure S375. F_{obs} (contour: 0.57) electron density map superimposed on the structure of **87** in the single crystal structure of **Ag_3Pz_3·87’**.

5. Supplementary references

1. Wang, J., Li, S., Yang, T., and Yang, J. (2014). Single-step synthesis of idebenone from Coenzyme Q0 via free-radical alkylation under silver catalysis. Tetrahedron *70*, 9029-9032. 10.1016/j.tet.2014.10.017.
2. Zhou, R.-Y., Li, N., Luo, W.-Y., Wang, L.-L., Zhang, Y.-Y., and Wang, J. (2021). A Simple and Convenient Two-step Synthesis of Idebenone. Org. Prep. Proced. Int. *53*, 397-401. 10.1080/00304948.2021.1917945.
3. Rasika Dias, H.V., Polach, S.A., and Wang, Z. (2000). Coinage metal complexes of 3,5-bis(trifluoromethyl)pyrazolate ligand: Synthesis and characterization of {[3,5-(CF3)2Pz]Cu}3 and {[3,5-(CF3)2Pz]Ag}3. J. Fluorine Chem. *103*, 163-169. 10.1016/S0022-1139(99)00313-9.
4. Sheldrick, G. (2015). SHELXT - Integrated space-group and crystal-structure determination. Acta Crystallogr. Sect. A: Found. Crystallogr. *71*, 3-8. 10.1107/S2053273314026370.
5. Sheldrick, G. (2015). Crystal structure refinement with SHELXL. Acta Crystallogr. Sect. C: Cryst. Struct. Commun. *71*, 3-8. doi:10.1107/S2053229614024218.
6. Dolomanov, O.V., Bourhis, L.J., Gildea, R.J., Howard, J.A.K., and Puschmann, H. (2009). OLEX2: a complete structure solution, refinement and analysis program. J. Appl. Crystallogr. *42*, 339-341. 10.1107/S0021889808042726.
7. Spek, A. (2009). Structure validation in chemical crystallography. Acta Crystallogr. Sect. D. Biol. Crystallogr. *65*, 148-155. 10.1107/S090744490804362X.
8. Omary, M.A., Elbjeirami, O., Gamage, C.S.P., Sherman, K.M., and Dias, H.V.R. (2009). Sensitization of Naphthalene Monomer Phosphorescence in a Sandwich Adduct with an Electron-Poor Trinuclear Silver(I) Pyrazolate Complex. Inorg. Chem. *48*, 1784-1786. 10.1021/ic8021326.
9. Titov, A.A., Filippov, O.A., Bilyachenko, A.N., Smol'yakov, A.F., Dolgushin, F.M., Belsky, V.K., Godovikov, I.A., Epstein, L.M., and Shubina, E.S. (2012). Complexes of Trinuclear Macrocyclic Copper(I) and Silver(I) 3,5-Bis(Trifluoromethyl)Pyrazolates with Ketones. Eur. J. Inorg. Chem. *2012*, 5554-5561. 10.1002/ejic.201200814.
10. Liu, R., Jin, P., Chen, J.-H., Yang, G., and Zhang, W. (2019). A green approach to the preparation of triangular silver(I) 3,5-bis(trifluoromethyl)pyrazolate: crystal structures of two adducts with triethylammonium nitrate or benzoic acid. Transition Met. Chem. *44*, 755-761. 10.1007/s11243-019-00345-z.
11. Liu, R., Zhang, W., Wei, D., Chen, J.-H., Ng, S.W., and Yang, G. (2019). Adducts of triangular silver(i) 3,5-bis(trifluoromethyl)pyrazolate with thiophene derivatives: a weak interaction model of desulfurization. Dalton Trans. *48*, 16162-16166. 10.1039/C9DT03344K.
12. Ghimire, M.M., Simon, O.C., Harris, L.M., Appiah, A., Mitch, R.M., Nesterov, V.N., Macchioni, A., Zuccaccia, C., Rabaâ, H., Galassi, R., and Omary, M.A. (2019). Binary Donor–Acceptor Adducts of Tetrathiafulvalene Donors with Cyclic Trimetallic Monovalent Coinage Metal Acceptors. Inorg. Chem. *58*, 15303-15319. 10.1021/acs.inorgchem.9b02294.
13. Titov, A.A., Smolyakov, A.F., Filippov, O.A., Belkova, N.V., and Shubina, E.S. (2022). Halogen or Arene: Complexation of 4,4'-Dibromobiphenyl with a Trinuclear Silver(I) Macrocycle. Russ. J. Coord. Chem. *48*, 615-621. 10.1134/S1070328422100086.

14. Olbrykh, A., Titov, A., Smol'yakov, A., Filippov, O., and Shubina, E.S. (2023). Exploring the Interaction of Pyridine-Based Chalcones with Trinuclear Silver(I) Pyrazolate Complex. Inorganics *11*, 175. 10.3390/inorganics11040175.
15. Zhan, S.-Z., Ding, F., Liu, X.-W., Zhang, G.-H., Zheng, J., and Li, D. (2019). White Light from Blue Fluorescence and Sensitized Yellow Long-Afterglow Phosphorescence of o-Terphenyl in Its π-Acid⋯Base Adduct with Ag3Pz3. Inorg. Chem. *58*, 12516-12520. 10.1021/acs.inorgchem.9b01911.
16. Song, J.-G., Zheng, J., Wei, R.-J., Huang, Y.-L., Jiang, J., Ning, G.-H., Wang, Y., Lu, W., Ye, W.-C., and Li, D. (2024). Crystalline mate for structure elucidation of organic molecules. Chem *10*, 924-937. 10.1016/j.chempr.2023.12.004.
